# Global, regional, and national epidemiology of ischemic stroke from 1990 to 2021

**DOI:** 10.1111/ene.16481

**Published:** 2024-09-17

**Authors:** Shuai Hou, Yifeng Zhang, Yulei Xia, Yong Liu, Xia Deng, Weihua Wang, Yanqiang Wang, Chunping Wang, Gang Wang

**Affiliations:** ^1^ Emergency Department Affiliated Hospital of Shandong Second Medical University Weifang China; ^2^ Department II of Neurology Affiliated Hospital of Shandong Second Medical University Weifang China; ^3^ School of Public Health Shandong Second Medical University Weifang China

**Keywords:** epidemiology, Global Burden of Disease, ischemic stroke, risk factor

## Abstract

**Background and purpose:**

This study aims to examine the global, regional, and national burden of ischemic stroke from 1990 to 2021.

**Method:**

We used data from the Global Burden of Disease (GBD) 2021 database to comprehensively assess ischemic stroke indicators globally, regionally, and in 204 countries, including incidence, deaths, disability‐adjusted life years (DALYs), estimated annual percentage change (EAPC), and Joinpoint regression analysis.

**Results:**

In 2021, there were a total of 7,804,449 cases of ischemic stroke globally (95% uncertainty interval = 6,719,760–8,943,692), with an age‐standardized incidence rate (ASIR) of 92.39. This represents a declining trend compared to 1990, with an EAPC of −0.67 (95% confidence interval [CI] = −0.76 to −0.58). Mortality and DALY rates also showed a downward trend (EAPC in age‐standardized mortality rate: −1.83, 95% CI = −1.92 to −1.74; EAPC in age‐standardized DALY rate = −1.59, 95% CI = −1.68 to −1.50). The burden of ischemic stroke was inversely correlated with gross domestic product. Regionally, from 2014 to 2021, the Caribbean experienced the fastest increase in ASIR (annual percent change = 0.15, 95% CI = 0.13 to 0.18). Among 204 countries, North Macedonia had the highest incidence, mortality, and DALY rates. In addition to metabolic risks, particulate matter pollution and low temperatures were significant environmental and occupational risk factors for ischemic stroke. Smoking and a diet high in sodium were identified as key behavioral risk factors.

**Conclusions:**

Ischemic stroke remains a serious global health challenge, and our results from this cross‐sectional study suggest that the burden of disease remains high in Eastern Europe, East Asia, Central Asia, and Sub‐Saharan Africa.

## Graphical abstract

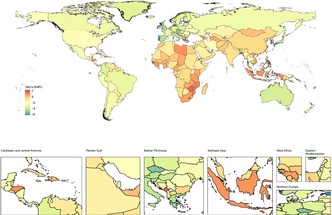

## INTRODUCTION

Ischemic stroke is a significant global health issue and a leading cause of long‐term disability and death [1]. It affects individuals across all age groups, necessitating a comprehensive understanding of its global and regional burden [2, 3, 4]. Historically, ischemic stroke has been a major contributor to the global health burden. Over the past decades, the incidence and impact of ischemic stroke have undergone notable changes. Recent trends indicate that shifts in population demographics and an increase in risk factors have influenced the burden of ischemic stroke in various populations [5, 6]. Despite extensive research, a thorough analysis of the burden of ischemic stroke by region, age group, and gender remains necessary.

Global Burden of Disease (GBD) 2021 provides the latest information on the distribution and burden of diseases and injuries over time by age, gender, and location. This database is regularly updated to estimate levels of exposure to risk factors, the relative health risks associated with these exposures, and the proportion of disease burden attributable to specific risk factors, broadly categorized into environmental and occupational, behavioral, and metabolic risk groups [7, 8]. According to the GBD 2021 database, the burden of ischemic stroke has exhibited significant trends from 1990 to 2021, underscoring the need for ongoing assessment and intervention. Regular reassessment of the disease burden is crucial for updating risk estimates and enhancing prevention and treatment strategies. Early intervention and continuous monitoring can significantly reduce the burden of ischemic stroke, particularly through addressing modifiable risk factors and implementing effective public health policies.

This study provides detailed insights into global and regional trends in ischemic stroke incidence, mortality, and disability‐adjusted life years (DALYs). Utilizing the GBD 2021 database, this study conducts a comprehensive analysis of the global burden of ischemic stroke from 1990 to 2021. Our objectives are to examine trends in the incidence, mortality, and DALYs of ischemic stroke across all age groups. Additionally, we aim to explore the relationship between the burden of ischemic stroke and economic development, as well as the impact of various risk factors. By providing these insights, we aim to assist health care professionals and policymakers in formulating effective strategies to mitigate the health risks and burden of ischemic stroke.

## METHODS

### Availability statement

All data are publicly available from the Global Health Data Exchange query tool (https://vizhub.healthdata.org/gbd‐results/).

### Study design and data collection

This study is a secondary data analysis based on the GBD 2021 database, which reports the incidence, prevalence, mortality, years lived with disability, DALYs, and healthy life expectancy for 371 diseases across 204 countries and regions from 1990 to 2021, along with their corresponding uncertainty intervals (UIs). It also provides data on 288 causes of death [7, 9].

In the GBD database, ischemic stroke is classified as a Level 4 cause, the most specific categorization. The geographical categories are divided into four levels. We analyzed the number of cases, incidence rates, number of deaths, mortality rates, number of DALYs, and age‐standardized DALY rate (ASDR) for ischemic stroke at the global level (Level 1), GBD super regions (Level 2), GBD regions (Level 3), and all 204 countries and regions (Level 4) from 1990 to 2021. We used linear regression to calculate the corresponding estimated annual percentage change (EAPC) and Joinpoint regression analysis.

### Gross domestic product

Gross domestic product (GDP) per capita at purchasing power parity is a useful measure for comparing living standards between nations, as it better reflects differences in cost of living and inflation rates [10]. Previous studies have shown a negative correlation between GDP and the incidence and mortality of many diseases [11, 12]. In this study, we explored the relationship between economic development and the burden of ischemic stroke by fitting GDP to standardized epidemiological indicators of GBD super regions.

### Statistical methods

All data were processed using R Studio software (version 4.4.0).

We primarily used the number of cases, incidence rates, number of deaths, mortality rates, number of DALYs, and ASDR to assess the burden of ischemic stroke. Each rate was reported per 100,000 people, and 95% UIs were determined according to the GBD algorithm. The dynamic trends of ischemic stroke were analyzed by calculating the EAPC to determine time trends in the disease burden. The 95% confidence interval (CI) of the EAPC was determined using a linear model. The EAPC assumes a linear relationship between age‐standardized rates (ASRs) and time, modeled as *y* = *α* + *βx* + *ϵ*, where *y* represents log_10_(ASR), *x* represents the calendar year, and *β* represents the regression coefficient. The EAPC is calculated using the formula EAPC = 100 × (10^*β*−1^). Unlike the 95% UIs used for other estimates, the EAPC is accompanied by a 95% CI [13, 14]. If both the EAPC and its 95% CI lower limit are greater than zero, the ASR is considered to be increasing, and vice versa.

To assess the relationship between GBD super regions and GDP, we adopted a methodology similar to previous studies on the sociodemographic index and regional trends. Additionally, we performed linear regression for each region to fit a line to the data points by minimizing the sum of squared differences between observed and predicted values. The slope represents the direction of the correlation between GDP and regional burden; a positive slope indicates a positive correlation, implying that higher economic levels are associated with a greater burden of stroke, whereas a negative slope indicates the opposite [15].

We also evaluated the risk factors leading to mortality in ischemic stroke patients using population attributable fractions (PAFs) and age‐standardized mortality rates (ASMRs). The PAF estimates the proportion of events attributable to specific risk factors, helping to quantify the preventable disease burden if these risk factors are eliminated [16]. ASMR is a standard indicator in global health assessments, and accurate mortality comparisons can guide resource allocation and funding to high‐mortality regions or groups, ensuring targeted interventions.

Joinpoint regression analysis was used to evaluate global, regional, and national trends in the burden of ischemic stroke and the trends in the burden attributable to various risk factors, following methods described in our previous studies [17].

All *p*‐values were two‐sided, with *p* < 0.05 considered statistically significant.

## RESULTS

### Burden of ischemic stroke: Global trends

#### Incidence

**FIGURE 1:**
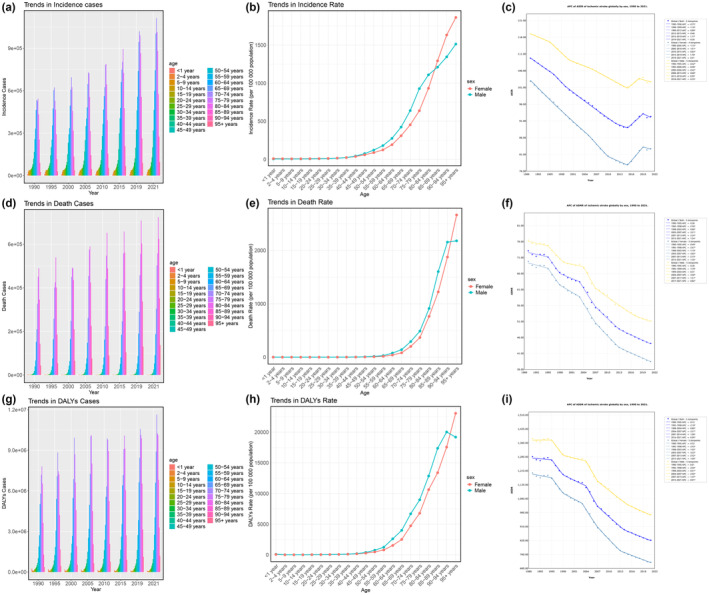
Disease burden of ischemic stroke at the global level from 1990 to 2021. (a) Trends in incidence cases. (b) Trends in incidence rate. (c) Annual percent change (APC) of age‐standardized incidence rate (ASIR) of ischemic stroke globally by sex. (d) Trends in death cases. (e) Trends in death rate. (f) APC of age‐standardized mortality rate (ASMR) of ischemic stroke globally by sex. (g) Trends in disability‐adjusted life years (DALYs) cases. (h) Trends in DALY rate. (i) APC of age‐standardized death rate (ASDR) of ischemic stroke globally by sex. Asterisks indicate *p* < 0.05.

In 2021, there were 7,804,449 cases of ischemic stroke worldwide (95% UI = 6,719,760–8,943,692), with an age‐standardized incidence rate (ASIR) of 92.39 per 100,000 people (95% UI = 79.84–105.82). From 1990 to 2021, the global incident cases of ischemic stroke increased by 87.97% (95% UI = 81.15%–94.54%), with an EAPC of −0.67 (95% CI = −0.76 to −0.58). Joinpoint regression analysis shows that the average annual percent change (AAPC) was −0.57 (95% CI = −0.66 to −0.48) from 1990 to 2021; however, there was no statistically significant difference in the annual percentage change (APC) from 2019 to 2021 (Figure 1, Table 1, and Table S1).

**TABLE 1:** Incidence of ischemic stroke between 1990 and 2021 at the global and regional level.

Location	1990		2021		1990–2021	
	Incident cases (95% UI)	ASIR (95% UI)	Incident cases (95% UI)	ASIR (95% UI)	Cases change (95% UI)	EAPC (95% CI)
Global	4,151,978 (3,536,772 to 4,868,150)	109.79 (93.56 to 127.62)	7,804,449 (6,719,760 to 8,943,692)	92.39 (79.84 to 105.82)	0.88 (0.81 to 0.95)	−0.67 (−0.76 to −0.58)
Regions						
Central Europe, Eastern Europe, and Central Asia	804,315 (677,639 to 941,531)	179.83 (153.44 to 207.12)	831,675 (719,788 to 950,858)	130.31 (113.67 to 147.64)	0.03 (−0.02 to 0.08)	−1.12 (−1.2 to −1.04)
Central Europe	222,510 (193,305 to 251,309)	157.98 (138.35 to 177.71)	238,905 (209,922 to 266,604)	107 (95.27 to 118.4)	0.07 (0.02 to 0.13)	−1.35 (−1.4 to −1.3)
Eastern Europe	515,846 (425,258 to 623,601)	197.9 (164.89 to 232.39)	490,197 (415,356 to 571,451)	142.57 (122.12 to 164.67)	−0.05 (−0.11 to 0.01)	−1.13 (−1.24 to −1.02)
Central Asia	65,959 (58,344 to 73,949)	141.86 (124.6 to 159.13)	102,573 (91,018 to 114,463)	132.92 (118.17 to 148.1)	0.56 (0.46 to 0.64)	−0.2 (−0.29 to −0.11)
High‐income	1,229,268 (1,055,194 to 1,418,850)	103.66 (89.77 to 119)	1,266,828 (1,118,369 to 1,425,018)	58.95 (52.28 to 66.1)	0.03 (−0.02 to 0.08)	−2.07 (−2.2 to −1.94)
Australasia	20,989 (19,362 to 22,541)	91.09 (84.12 to 97.82)	28,160 (25,188 to 31,109)	52.76 (47.35 to 58.52)	0.34 (0.24 to 0.43)	−2.05 (−2.2 to −1.91)
High‐income Asia Pacific	234,946 (193,848 to 280,991)	120.98 (100.7 to 143.98)	284,445 (249,283 to 322,678)	64.59 (56.36 to 73.72)	0.21 (0.10 to 0.33)	−2.53 (−2.73 to −2.33)
High‐income North America	315,394 (259,208 to 383,545)	89.95 (74.41 to 108.35)	352,963 (299,100 to 413,294)	56.86 (48.63 to 66.11)	0.12 (0.05 to 0.20)	−1.58 (−1.73 to −1.43)
Southern Latin America	44,172 (38,720 to 49,910)	97.84 (86.31 to 109.85)	52,070 (46,169 to 58,185)	60.35 (53.46 to 67.61)	0.18 (0.10 to 0.26)	−1.77 (−1.93 to −1.62)
Western Europe	613,766 (538,423 to 692,798)	105.97 (93.99 to 117.98)	549,191 (496,802 to 601,606)	58.14 (52.88 to 63.67)	−0.11 (−0.16 to −0.05)	−2.12 (−2.21 to −2.03)
Latin America and Caribbean	207,783 (177,268 to 241,693)	94.1 (79.97 to 110.27)	357,281 (308,111 to 408,879)	58.8 (50.86 to 67.09)	0.72 (0.64 to 0.80)	−1.7 (−1.82 to −1.58)
Andean Latin America	13,563 (11,785 to 15,387)	63.46 (55.42 to 71.89)	27,415 (24,042 to 31,116)	46.29 (40.6 to 52.41)	1.02 (0.92 to 1.12)	−1.13 (−1.24 to −1.02)
Caribbean	19,577 (17,216 to 21,953)	76.19 (67.54 to 85.71)	36,042 (32,142 to 39,961)	67.34 (59.96 to 74.74)	0.84 (0.76 to 0.93)	−0.42 (−0.46 to −0.37)
Central Latin America	68,643 (58,879 to 78,851)	80.45 (69.05 to 93.22)	127,075 (110,155 to 144,196)	51.96 (45.09 to 58.83)	0.85 (0.76 to 0.95)	−1.67 (−1.82 to −1.51)
Tropical Latin America	106,000 (88,156 to 125,886)	119.16 (98.34 to 142.27)	166,749 (140,998 to 194,698)	66.42 (55.99 to 77.52)	0.57 (0.47 to 0.68)	−2.04 (−2.16 to −1.92)
North Africa and Middle East	199,912 (175,528 to 229,003)	117.53 (102.61 to 133.52)	461,146 (409,815 to 516,837)	103.58 (91.57 to 116.06)	1.31 (1.20 to 1.43)	−0.43 (−0.47 to −0.39)
South Asia	398,076 (331,672 to 474,038)	72.63 (60.6 to 86.45)	853,370 (727,638 to 990,209)	61.16 (52.33 to 70.75)	1.14 (1.03 to 1.26)	−0.74 (−0.82 to −0.66)
Southeast Asia, East Asia, and Oceania	1,068,452 (886,219 to 1,295,199)	102.86 (85.2 to 123.06)	3,522,664 (2,935,605 to 4,169,755)	129.38 (108.76 to 151.13)	2.30 (2.11 to 2.50)	0.69 (0.64 to 0.74)
East Asia	800,330 (656,281 to 982,073)	101.31 (82.99 to 121.88)	2,850,090 (2,363,158 to 3,405,882)	134.77 (112.62 to 158.4)	2.56 (2.34 to 2.81)	0.86 (0.8 to 0.92)
Southeast Asia	265,779 (228,020 to 310,151)	107.32 (91.5 to 125.56)	667,263 (582,281 to 763,035)	107.31 (93.68 to 122.69)	1.51 (1.40 to 1.63)	−0.04 (−0.07 to 0)
Oceania	2343 (2017 to 2728)	81.5 (70.05 to 94.27)	5311 (4625 to 6003)	73.2 (63.72 to 82.91)	1.27 (1.15 to 1.40)	−0.43 (−0.47 to −0.39)
Sub‐Saharan Africa	244,171 (208,494 to 284,034)	110.84 (93.78 to 130.18)	511,485 (444,646 to 580,614)	103.15 (88.81 to 117.81)	1.09 (1.02 to 1.18)	−0.25 (−0.3 to −0.2)
Central Sub‐Saharan Africa	27,144 (23,222 to 32,005)	122.67 (104.25 to 142.58)	59,173 (51,496 to 68,041)	108.99 (94.95 to 124.84)	1.18 (1.05 to 1.33)	−0.41 (−0.45 to −0.38)
Eastern Sub‐Saharan Africa	85,162 (72,719 to 99,207)	110.88 (94.18 to 130.12)	179,318 (154,966 to 204,334)	103.55 (89.22 to 118.4)	1.11 (1.01 to 1.20)	−0.21 (−0.26 to −0.17)
Southern Sub‐Saharan Africa	33,295 (28,141 to 39,225)	122.29 (101.93 to 146.66)	64,729 (54,456 to 74,991)	121.86 (102.46 to 142.18)	0.94 (0.86 to 1.05)	−0.11 (−0.36 to 0.15)
Western Sub‐Saharan Africa	98,570 (83,977 to 114,607)	104.07 (88.24 to 122.05)	208,265 (181,391 to 236,454)	94.74 (82.14 to 106.92)	1.11 (1.02 to 1.20)	−0.29 (−0.33 to −0.25)

Abbreviations: ASIR, age‐standardized incidence rate; EAPC, estimated annual percentage change.

In 2021, the incident cases of ischemic stroke were higher in men than in women. Men had 4,022,421 cases (95% UI = 3,444,603–4,666,933) compared to women's 3,782,028 cases (95% UI = 3,253,626–4,336,193). Similarly, the ASIR for men was 102.77 per 100,000 (95% UI = 88.65–118.80), higher than the rate for women at 82.85 per 100,000 (95% UI = 71.31–94.81). From 1990 to 2021, the incidence in men increased by 100.02% (95% UI = 91.88%–108.36%), whereas in women it increased by 76.65% (95% UI = 70.64%–83.24%). Joinpoint regression analysis indicated no significant APC for women from 2019 to 2021, whereas men had an APC of −0.349 (95% CI = −0.657 to −0.041) from 2018 to 2021 (Figure 1).

As shown in Figure 1, in 2021, the incidence rates for men and women were similar from ages 0 to 40 years. However, from ages 40 to 84 years, men had higher incidence rates than women, and for those 85 years and older, women had higher incidence rates than men. The incidence peaked for both genders at ages 95 years and older. From 1990 to 2021, the highest growth rate in incidence was observed in men aged 95 years and older at 312.38% (95% UI = 281.52%–348.53%) and in women at 273.86% (95% UI = 246.01%–306.84%).

#### Mortality

In 2021, there were 3,591,499 deaths due to ischemic stroke globally (95% UI = 3,213,281–3,888,327), with a growth rate of 55.00% from 1990 to 2021 (95% UI = 43.20%–66.76%). The ASMR was 44.18 per 100,000 people (95% UI = 39.29–47.81), with an EAPC of −1.83 (95% CI = −1.92 to −1.74). Joinpoint regression analysis indicated an APC of −1.24 (95% CI = −1.45 to −1.03) from 2013 to 2021 and AAPC of −1.60 (−1.81 to −1.39) from 1990 to 2021 (Figure 1 and Table S2).

In 2021, more women (1,812,764, 95% UI = 1,573,025–2,001,279) than men (1,778,735, 95% UI = 1,611,777–1,962,926) died from ischemic stroke. However, the ASMR was higher for men at 51.16 per 100,000 (95% UI = 46.21–56.22) compared to 38.54 per 100,000 (95% UI = 33.46–42.53) for women. From 1990 to 2021, the change in death cases was 76.56% (95% UI = 56.08%–99.55%) for men, higher than the 38.41% (95% UI = 27.12%–50.26%) for women. Joinpoint regression analysis showed an APC of −1.35 (95% CI = −1.59 to −1.11) for women from 2013 to 2021, and an APC of −0.82 (95% CI = −1.44 to −0.19) for men from 2017 to 2021 (Figure 1).

As shown in Figure 1, in 2021, mortality rates were similar for both genders from ages 0 to 50 years. However, from ages 50 to 94 years, men had higher mortality rates than women, whereas for those 85 years and older, women had higher mortality rates than men. The mortality rate peaked for both genders at ages 95 years and older. From 1990 to 2021, the highest growth rate in mortality cases was observed in men aged 95 years and older at 242.57% (95% UI = 222.67%–261.30%) and in women at 207.24% (95% UI = 187.95%–222.95%).

#### Disability‐adjusted life years

In 2021, the global number of DALYs due to ischemic stroke was 70,357,912 (95% UI = 64,329,576–76,007,063), with a growth rate of 223.42% from 1990 to 2021 (95% UI = 207.82%–236.20%). The ASDR was 837.36 per 100,000 people (95% UI = 763.73–904.98), with an EAPC of −1.59 (95% CI = −1.68 to −1.5). Joinpoint regression analysis indicated an APC of −0.95 (95% CI = −1.14 to −0.76) from 2014 to 2021, and AAPC of −1.37 (95% CI = −1.53 to −1.20) from 1990 to 2021 (Figure 1 and Table S3).

In 2021, more men (37,007,430, 95% UI = 33,581,055–40,659,723) than women (33,350,482, 95% UI = 29,775,875–36,653,245) experienced DALYs due to ischemic stroke. The ASDR was also higher for men at 975.3 per 100,000 (95% UI = 885.61–1069.81) compared to 719.52 per 100,000 (95% UI = 642.82–791.64) for women. From 1990 to 2021, the change in DALYs was 223.42% (95% UI = 207.82%–236.20%) for men, higher than the 214.80% (95% UI = 198.86%–228.89%) for women. Joinpoint regression analysis showed an APC of −1.05 (95% CI = −1.27 to −0.83) for women from 2013 to 2021, and an APC of −0.91 (95% CI = −1.14 to −0.68) for men from 2015 to 2021 (Figure 1).

As shown in Figure 1, in 2021, ASDRs were similar for both genders from ages 0 to 40 years. However, from ages 40 to 94 years, men had a higher ASDR than women, whereas for those 95 years and older, women had a higher ASDR than men. The ASDR peaked for men at ages 90–94 years and for women at ages 95 years and older. From 1990 to 2021, the highest growth rate in DALYs was observed in men aged 95 years and older at 253.75% (95% UI = 272.33%–235.78%) and in women at 214.80% (95% UI = 198.86%–228.89%).

### Burden of ischemic stroke: Geographic regional trends

#### Incidence

**FIGURE 2:**
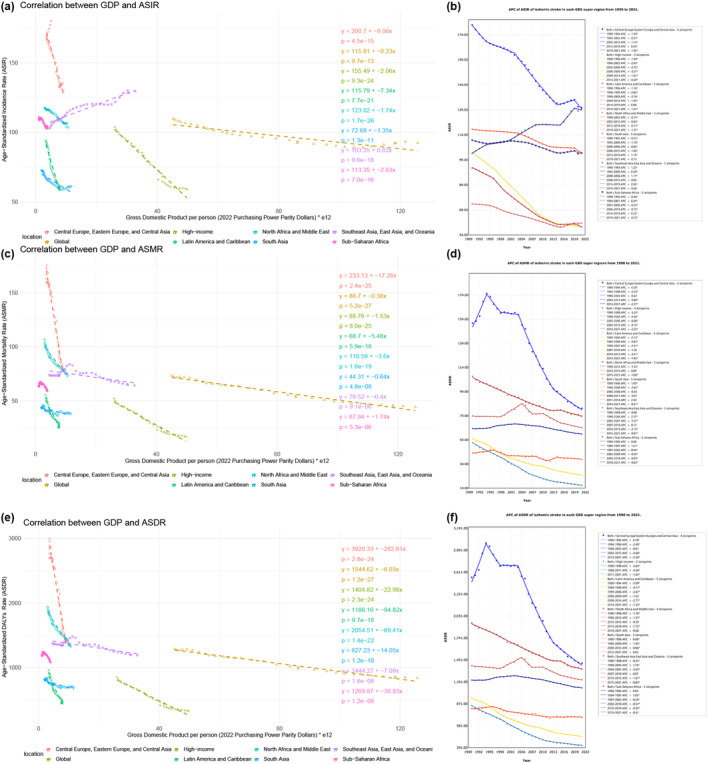
Disease burden of ischemic stroke by Global Burden of Disease (GBD) super region. (a) Correlation graph between gross domestic product (GDP) and age‐standardized incidence rate (ASIR). (b) Annual percentage change (APC) of ASIR of ischemic stroke in each GBD super region from 1990 to 2021. (c) Correlation graph between GDP and age‐standardized mortality rate (ASMR). (d) APC of ASMR of ischemic stroke in each GBD super region from 1990 to 2021. (e) Correlation graph between GDP and age‐standardized disability‐adjusted life year rate (ASDR). (f) APC of ASDR of ischemic stroke in each GBD super region from 1990 to 2021.

The GBD database divides the world into seven super geographical regions and 21 smaller subregions. In 2021, the South Asia region had the highest number of cases at 853,370 (95% UI = 727,638–990,209), whereas Central Europe, Eastern Europe, and Central Asia had the highest ASIR at 130.31 per 100,000 (95% UI = 113.67–147.64). From 1990 to 2021, only Southeast Asia, East Asia, and Oceania had a positive EAPC at 0.69 (95% CI = 0.64 to 0.74), whereas high‐income regions had the lowest EAPC at −2.07 (95% CI = −2.2 to −1.94; Table 1). As shown in Figure 2, GDP and ASIR were generally negatively correlated across regions.

Among the 21 subregions, 11 had ASIRs lower than the global average, such as Andean Latin America and Central Latin America, whereas 10 regions, including Eastern Europe and East Asia, had higher rates. In 2021, East Asia had the highest number of ischemic stroke cases (2,850,090, 95% UI = 2,363,158–3,405,882), whereas Oceania had the fewest cases (5311, 95% UI = 4625–6003). The highest ASIR was in Eastern Europe (142.57 per 100,000, 95% UI = 122.12–164.67), whereas Andean Latin America had the lowest (46.29 per 100,000, 95% UI = 40.6–52.41). From 1990 to 2021, the incidence rate of ischemic stroke decreased in all regions, with the largest decrease in high‐income Asia Pacific (EAPC = −2.53, 95% CI = −2.73 to −2.33). However, the Caribbean showed an increasing trend in incidence from 2014 to 2021 (APC = 0.15, 95% CI = 0.13 to 0.18), as did Southeast Asia from 2015 to 2021 (APC = 0.13, 95% CI = 0.02 to 0.24; Table 1 and Figure S1).

#### Mortality

In 2021, Southeast Asia, East Asia, and Oceania had the highest number of deaths at 1,545,602 (95% UI = 1,326,302–1,757,131), whereas Central Europe, Eastern Europe, and Central Asia had the highest ASMR at 80.11 per 100,000 (95% UI = 73.16–85.14). From 1990 to 2021, all regions showed a decreasing trend in mortality rates. Sub‐Saharan Africa had the slowest decline (EAPC = −0.41, 95% CI = −0.46 to −0.36), whereas high‐income regions had the fastest decline (EAPC = −3.94, 95% CI = −4.1 to −3.78; Table S2). As shown in Figure 2, GDP and ASMR were negatively correlated across regions.

Among the 21 subregions, 11 had ASMRs lower than the global average, whereas 10 regions had higher rates. East Asia had the highest number of deaths due to ischemic stroke (1,202,218, 95% UI = 1,010,916–1,397,915), whereas Oceania had the fewest deaths (1843, 95% UI = 1488–2389). Eastern Europe had the highest ASMR (90.99 per 100,000, 95% UI = 82.79–98.48), whereas Australasia had the lowest (14.11 per 100,000, 95% UI = 11.44–15.55). Eastern Sub‐Saharan Africa showed the largest increase in ASMR (EAPC = 0.94, 95% CI = 0.47 to 1.42) and was the only region with an increasing ASMR. High‐income Asia Pacific had the largest decrease (EAPC = −4.76, 95% CI = −4.91 to −4.6). From 2015 to 2021, the Caribbean showed an increasing trend in mortality (APC = 0.67, 95% CI = 0.45 to 0.89), whereas Southern Latin America had the largest decrease, from 2016 to 2021 (APC = −4.12, 95% CI = −5.15 to −3.07; Table S2 and Figure S2).

#### Disability‐adjusted life years

In 2021, Central Europe, Eastern Europe, and Central Asia had the highest number of DALYs at 9,292,425 (95% UI = 8,664,271–9,856,616) and the highest ASDR at 1407.63 per 100,000 (95% UI = 1311.67–1494.26). From 1990 to 2021, all regions showed a decreasing trend in ASDR. Sub‐Saharan Africa had the slowest decline (EAPC = −0.31, 95% CI = −0.42 to −0.20), whereas high‐income regions had the fastest decline (EAPC = −3.36, 95% CI = −3.52 to −3.2; Table S3) As shown in Figure 2, GDP and ASDR were negatively correlated across regions.

In 2021, the global ASDR for ischemic stroke was 837.36 per 100,000. Eleven regions, including Australasia and Western Europe, had ASDRs lower than the global average, whereas 10 regions, including Eastern Europe and Central Asia, had higher rates. Among the 21 subregions, East Asia had the highest number of DALYs due to ischemic stroke (24,021,156, 95% UI = 20,420,316–27,562,229), whereas Oceania had the fewest (48,691, 95% UI = 40,675–59,299). Eastern Europe had the highest ASDR (1601.2 per 100,000, 95% UI = 1483.51–1723.12), whereas Australasia had the lowest (249.45 per 100,000, 95% UI = 216.51–278.22). From 1990 to 2021, southern Sub‐Saharan Africa showed the largest increase in ASDR (EAPC = 0.64, 95% CI = 0.22 to 1.06) and was the only region with an increasing ASDR. High‐income Asia Pacific had the largest decrease (EAPC = −3.99, 95% CI = −4.14 to −3.83). In our joinpoint regression model, Central Sub‐Saharan Africa was the only region showing an increasing trend from 2016 to 2021 (APC = 0.55, 95% CI = 0.25 to 0.84; Table S3 and Figure S3).

### Burden of ischemic stroke: National trends

#### Incidence

**FIGURE 3:**
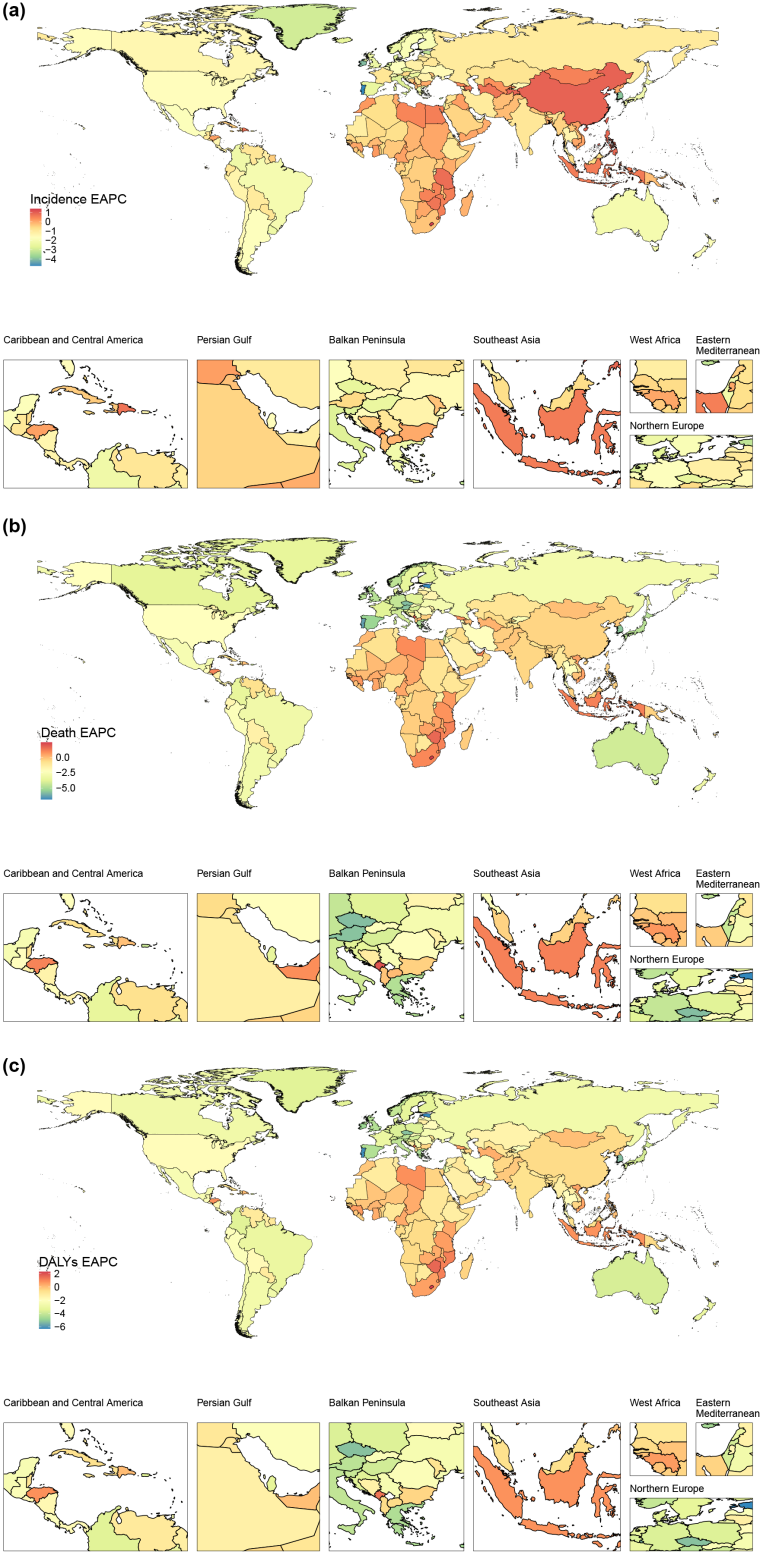
The national burden of ischemic stroke in 204 countries and territories (1990 to 2021). (a) Estimated annual percentage change (EAPC) for age‐standardized incidence rate. (b) EAPC for age‐standardized mortality rate. (c) EAPC for age‐standardized disability‐adjusted life‐year (DALY) rate.

In 2021, the global ASIR of ischemic stroke was 92.39 per 100,000. Among the 204 countries, 97 had ASIRs higher than the global average, whereas 107 had lower rates. China had the highest number of cases (2,772,053, 95% UI = 2,295,713–3,319,150), and Tokelau had the fewest (1, 95% UI = 1–1). North Macedonia had the highest ASIR (214.36 per 100,000, 95% UI = 190.48–239.92), whereas Malta had the lowest (38.89 per 100,000, 95% UI = 33.9–44.52). Lesotho showed the largest increase in ASIR (EAPC = 1.39, 95% CI = 1.23 to 1.55), whereas Singapore had the largest decrease (EAPC = −4.09, 95% CI = −4.37 to −3.81). In recent years, 41 countries showed an increasing trend in ASIR, with Cyprus having the fastest increase from 2014 to 2021 (APC = 1.73, 95% CI = 1.35 to 2.12). Most countries showed a decreasing trend, with Israel having the fastest decrease from 2019 to 2021 (APC = −8.79, 95% CI = −9.49 to −8.092; Figures 3, S4,and S5 and Table S4).

#### Mortality

In 2021, the global ASMR of ischemic stroke was 44.18 per 100,000. Among the 204 countries, 113 had ASMRs higher than the global average, whereas 91 had lower rates. China had the highest number of deaths (1,176,952, 95% UI = 986,876–1,372,707), whereas Niue and Tokelau had the fewest (1, 95% UI = 1–1). North Macedonia had the highest ASMR (216.89 per 100,000, 95% CI = 184.01 to 249.37), whereas Singapore had the lowest (6.97 per 100,000, 95% UI = 5.77–7.78). Lesotho showed the largest increase in ASMR (EAPC = 2.39, 95% CI = 1.91 to 2.86), whereas Estonia had the largest decrease (EAPC = −6.83, 95% CI = −7.44 to −6.22). In recent years, 26 countries showed an increasing trend in ASMR, with Iraq having the fastest increase from 2017 to 2021 (APC = 6.48, 95% CI = 4.98 to 8.01). Most countries showed a decreasing trend, with San Marino having the fastest decrease from 2019 to 2021 (APC = −24.23, 95% CI = −32.96 to −14.37; Figures 3, S4, and S5 and Table S5).

#### Disability‐adjusted life years

In 2021, the global ASDR for ischemic stroke was 837.36 per 100,000. Among the 204 countries, 108 had ASDRs higher than the global average, whereas 96 had lower rates. China had the highest number of DALYs (23,430,411, 95% UI = 19,918,946–26,933,909), whereas Tokelau had the fewest (13, 95% UI = 10–15). North Macedonia had the highest ASDR (3037.44 per 100,000, 95% UI = 2559.01–3507.51), whereas Puerto Rico had the lowest (180.9 per 100,000, 95% UI = 154.41–208.22). Lesotho showed the largest increase in ASDR (EAPC = 2.3, 95% CI = 1.88 to 2.73), whereas Estonia had the largest decrease (EAPC = −6.3, 95% CI = −6.84 to −5.77). In recent years, 19 countries showed an increasing trend in ASDR, with Iraq having the fastest increase from 2017 to 2021 (APC = 4.38, 95% CI = 3.19 to 5.58). Most countries showed a decreasing trend, with San Marino having the fastest decrease from 2019 to 2021 (APC = −17.53, 95% CI = −23.32 to −11.30; Figures 3, S4, and S5 and Table S6).

### Risk factors

**FIGURE 4:**
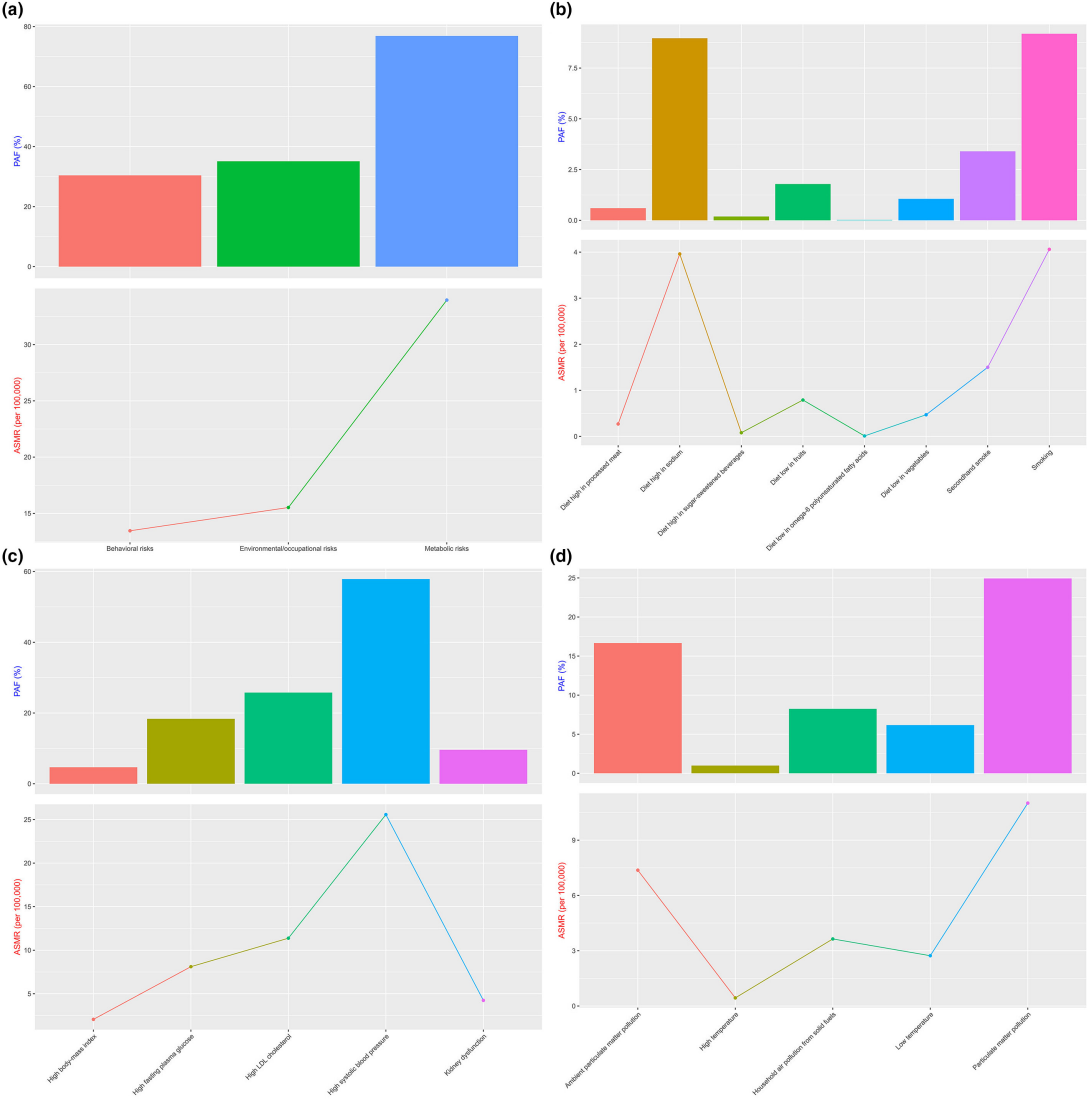
Risk of death from ischemic stroke attributable to each risk factor in 2021. (a) Environmental and occupational risks, behavioral risks, and metabolic risks: age‐standardized mortality rate (ASMR) and population attributable fraction (PAF) of attributable ischemic stroke. (b) ASMR and PAF of ischemic stroke attributable to various environmental and occupational risks. (c) ASMR and PAF of ischemic stroke attributable to various behavioral risks. (d) ASMR and PAF of ischemic stroke attributable to various metabolic risks. LDL, low‐density lipoprotein.

As shown in Table 2 and Figure 4, Environmental and occupational risks collectively contributed to 35.13% of the PAF, with an ASMR of 15.52. Since 2007, there has been a notable APC of −2.32% (95% CI = −2.44 to −2.20), indicating a significant reduction.

**TABLE 2:** Annual percent change and ASMR of ischemic stroke attributable to each risk factor.

Risk factor	PAF, %	ASMR
Environmental/occupational risks	35.13	15.52
Particulate matter pollution	24.93	11.01
Ambient particulate matter pollution	16.67	7.37
Household air pollution from solid fuels	8.25	3.64
High temperature	0.99	0.44
Low temperature	6.17	2.73
Behavioral risks	30.43	13.45
Smoking	9.19	4.06
Secondhand smoke	3.4	1.5
Diet high in sodium	8.97	3.96
Diet low in vegetables	1.06	0.47
Diet low in fruits	1.79	0.79
Diet high in processed meat	0.6	0.27
Diet high in sugar‐sweetened beverages	0.19	0.08
Diet low in omega‐6 polyunsaturated fatty acids	0.02	0.01
Metabolic risks	76.88	33.96
High systolic blood pressure	57.89	25.57
High LDL cholesterol	25.78	11.38
High fasting plasma glucose	18.36	8.11
Kidney dysfunction	9.59	4.24
High body mass index	4.66	2.06

Abbreviations: ASMR, age‐standardized mortality rate; LDL, low‐density lipoprotein; PAF, population attributable fraction.

Particulate matter pollution was identified as a major environmental risk, with a PAF of 24.93% and an ASMR of 11.01. However, from 2019 onward, the APC was −0.86 (95% CI = −3.69 to 2.06), which was not statistically significant. Ambient particulate matter pollution had a PAF of 16.67% and an ASMR of 7.37, with a significant APC of −2.86 (95% CI = −3.68 to −2.03) since 2015. Household air pollution from solid fuels, with a PAF of 8.25% and an ASMR of 3.64, showed an APC of −0.83 (95% CI = −3.78 to 2.22) from 2019, which was also not statistically significant (Figure S6, Table S7).

Behavioral risks accounted for 30.43% of the PAF and an ASMR of 13.45. Since 2015, the APC for behavioral risks showed a significant decrease of −1.15 (95% CI = −1.38 to −0.91). Smoking, a significant behavioral risk factor, had a PAF of 9.19% and an ASMR of 4.06, with an APC of −1.19 (95% CI = −1.95 to −0.43) since 2018. Secondhand smoke contributed to a PAF of 3.4% and an ASMR of 1.5, with a significant APC of −1.24 (95% CI = −1.50 to −0.98) since 2015. A diet high in sodium, with a PAF of 8.97% and an ASMR of 3.96, had an APC of −0.88 (95% CI = −1.11 to −0.65) since 2015.

Metabolic risks were the most significant contributors, with a PAF of 76.88% and an ASMR of 33.96. Since 2013, the APC for metabolic risks indicated a significant reduction of −1.25 (95% CI = −1.43 to −1.06). High systolic blood pressure had the highest PAF among metabolic risks at 57.89%, with an ASMR of 25.57 and an APC of −1.22 (95% CI = −1.44 to −0.99) since 2014. High low‐density lipoprotein (LDL) cholesterol, contributing a PAF of 25.78% and an ASMR of 11.38, showed a significant APC of −1.29 (95% CI = −1.48 to −1.11) since 2013. High fasting plasma glucose had a PAF of 18.36% and an ASMR of 8.11, with an APC of −1.02 (95% CI = −1.11 to −0.92) since 2007. Kidney dysfunction, with a PAF of 9.59% and an ASMR of 4.24, had an APC of −1.45 (95% CI = −1.59 to −1.31) since 2012. High body mass index had a PAF of 4.66% and an ASMR of 2.06, with an APC of 0.08 (95% CI = −0.16 to 0.31) since 2013, which was not statistically significant.

## DISCUSSION

This study utilizes the GBD 2021 database to conduct a comprehensive analysis of the global burden of ischemic stroke from 1990 to 2021. The results indicate significant trends in the incidence, mortality, and DALYs of ischemic stroke. During the study period, the global incidence of ischemic stroke increased by 87.97%, whereas mortality rates showed a declining trend. Additionally, the study highlights a higher burden of ischemic stroke among men compared to women and points out significant regional differences in stroke burden.

The observed global increase in ischemic stroke incidence, which rose by 87.97% from 1990 to 2021, reflects broader demographic changes, including population aging and increased risk factors. This aligns with Pu et al.'s report predicting that by 2030, the global age‐standardized incidence of ischemic stroke will rise to 89.32 per 100,000 people [18]. Similarly, from 1990 to 2016, the absolute number of strokes, deaths, and disabilities nearly doubled [19], consistent with our findings. Furthermore, the declining trend in stroke mortality observed in our study is consistent with national reports, such as the significant reduction in stroke mortality rates in the United States, where ischemic stroke mortality has decreased more than hemorrhagic stroke mortality [20], and the declining stroke mortality rates in China, particularly among women [21].

Our study found that men have a higher incidence and burden of ischemic stroke compared to women, corroborating numerous research findings. A 10‐year analysis of the German statewide registry reported an approximate 4% increase in ischemic stroke incidence among men, whereas this decreased from 220/100,000 to 208/100,000 among women [22]. Similarly, women aged 45–64 years have a lower stroke risk than men [23]. Dimitrov et al. also found that men had higher age‐adjusted incidence rates of ischemic stroke across most age groups. This gender difference is often attributed to higher rates of hypertension, smoking, and alcohol consumption among men [24].

Significant regional differences in stroke burden were observed in our study, consistent with previous research highlighting the impact of socioeconomic factors on stroke outcomes. Incidence, case fatality, and mortality rates of stroke show variations between countries, with shifting burdens between high‐income and low‐ and middle‐income countries [25]. We found the highest incidence rates in Central Europe, Eastern Europe, and Central Asia, consistent with the GBD 2019 report. East Asia had the highest age‐standardized incidence rate of ischemic stroke in 2019 and the largest increase in ASIR from 1990 to 2019 [26]. Our study observed a negative correlation between GDP and age‐standardized rates of stroke incidence, mortality, and DALYs. Additionally, research indicates that long‐term poststroke mortality is inversely proportional to income, highlighting social inequalities in new disease mortality rather than poststroke survival [27].

Metabolic, behavioral, and environmental risks were identified as significant contributors to the burden of ischemic stroke. The substantial increase in ischemic stroke burden is not solely due to rapid population growth and aging but also to exposure to several key risk factors, such as smoking, a high‐sodium diet, high systolic blood pressure, high LDL cholesterol, particulate matter pollution, ambient particulate matter pollution, high fasting plasma glucose, and low temperature. In addition to metabolic factors, GBD 2019 Viewpoint Collaborators found that nonoptimal temperatures, especially low temperatures, significantly impact ischemic stroke burden, further supporting a systematic analysis of temperature effects on ischemic stroke [28]. Our study confirmed and extended previous findings on the impact of nonoptimal temperatures, with PAF and ASMR for low temperatures being 6.17% and 2.73%, respectively, whereas high temperatures had a PAF of 0.99% and an ASMR of 0.44%. These findings align with prior research on environmental temperature changes and stroke occurrence [26]. Countries or regions should develop ischemic stroke policies related to regional and environmental temperatures, especially to reduce the burden in extremely cold areas. Particulate matter pollution was also emphasized in Verhoeven et al.'s study, linking air pollution to increased stroke risk [29]. Our findings reinforce the need for policies to improve air quality and reduce exposure to harmful pollutants. Behavioral risks, including smoking and high sodium intake, are significant as well. Research has shown that smokers have a higher overall risk of stroke compared to nonsmokers, with a combined odds ratio of 1.61 [30]. Our study observed a significant annual percentage change decline in these risk factors in recent years, indicating progress in public health interventions aimed at reducing these behaviors.

The strength of this study lies in the use of comprehensive, globally representative data from the GBD 2021 database, allowing robust analysis of time and regional trends. The use of standardized indicators and advanced statistical methods, such as Joinpoint regression analysis, enhances the reliability of the findings. However, some limitations should be noted. Reliance on secondary data may introduce biases related to data accuracy and completeness [31]. Additionally, due to the limitations inherent in utilizing public databases, this study did not account for the epidemiology of subtypes of ischemic stroke [32]. Future research should refine the epidemiology of each subtype, providing detailed insights into the incidence, mortality, and disability associated with each subtype.

In conclusion, this study provides crucial insights into the global burden of ischemic stroke, highlighting significant temporal trends and regional differences. The findings underscore the importance of ongoing efforts to manage and reduce stroke risk factors, particularly in low‐ and middle‐income regions. Strengthening health care infrastructure, improving access to preventive measures, and addressing the social determinants of health are essential for mitigating the global stroke burden. By addressing these challenges, we can move toward more equitable and effective stroke prevention and management strategies, ultimately reducing the global burden of ischemic stroke.

## AUTHOR CONTRIBUTIONS

**Shuai Hou:** Conceptualization; investigation; methodology; validation; visualization; writing – original draft; writing – review and editing; software; formal analysis; project administration; data curation; supervision; resources. **Yifeng Zhang:** Investigation; writing – original draft; writing – review and editing; methodology; software; resources; data curation; visualization. **Yulei Xia:** Writing – original draft; writing – review and editing; investigation; methodology; software; data curation. **Yong Liu:** Writing – original draft; writing – review and editing; methodology; software; data curation; resources. **Xia Deng:** Writing – original draft; writing – review and editing; validation; conceptualization; supervision; formal analysis. **Weihua Wang:** Methodology; software; data curation; writing – review and editing; writing – original draft; formal analysis. **Yanqiang Wang:** Conceptualization; writing – original draft; writing – review and editing; methodology; software. **Chunping Wang:** Validation; funding acquisition; writing – original draft; writing – review and editing; visualization; conceptualization; supervision. **Gang Wang:** Conceptualization; investigation; funding acquisition; writing – original draft; writing – review and editing; validation; formal analysis; project administration; resources; supervision; data curation; methodology.

## FUNDING INFORMATION

This work was supported by Science and Technology Development Project of Shandong Second Medical University (2023FYM001, 2023FYM006), Health China–BuChang ZhiYuan Public Welfare Projects for Heart and Brain Health (HIGHER2023072), Joint Scientific Research Plan Project of China (LH2022GG03), Clinical Research Center of Affiliated Hospital of Shandong Second Medical University (wyfy‐2022‐ky‐148), Medical and Health Science and Technology Project of Shandong Province (202310001434), Yuan Du Scholars, and Weifang Key Laboratory.

## CONFLICT OF INTEREST STATEMENT

The authors declare that the research was conducted in the absence of any commercial or financial relationships that could be construed as a potential conflict of interest.

## ETHICS STATEMENT

Our study was approved by the ethics committee of the Affiliated Hospital of Shandong Second Medical University (approval no. wyfy‐2024‐qt‐034). Additionally, informed consent was not required for this study, as the analysis did not involve any identifiable personal information.

## Supporting information


APPENDIX S1.

**TABLE S1.** The AAPC of incidence, death, and DALYs of ischemic stroke at global, regional, and national levels. AAPC, average annual percent change; DALY, disability‐adjusted life year.

**TABLE S2.** Deaths by ischemic stroke between 1990 and 2021 at the global and regional levels. ASMR, age‐standardized mortality rate; EAPC, estimated annual percentage change.

**TABLE S3.** DALYs due to ischemic stroke between 1990 and 2021 at the global and regional levels. ASDR, age‐standardized DALY rate; DALY, disability‐adjusted life years; EAPC, estimated annual percentage change.

**TABLE S4.** Incidence of ischemic stroke at the national level. ASIR, age‐standardized incidence rate; EAPC, estimated annual percentage change.

**TABLE S5.** Deaths by ischemic stroke at the national level. ASMR, age‐standardized mortality rate; EAPC, estimated annual percentage change.

**TABLE S6.** DALYs of ischemic stroke at the national level. ASDR, age‐standardized DALY rate; DALY, disability‐adjusted life year; EAPC, estimated annual percentage change.

**TABLE S7.** APC of incidence, death, and DALYs for ischemic stroke in 204 countries and territories. APC, annual percent change; DALY, disability‐adjusted life year.

## Data Availability

The original contributions presented in the study are included in the supplementary material; further inquiries can be directed to the corresponding authors.
