# Supplementary material for: Global, regional, and national epidemiology of ischemic stroke from 1990 to 2021
Source: Eur J Neurol. 2024 Sep 17;31(12):e16481. doi: 10.1111/ene.16481 (PMC11555022; doi:10.1111/ene.16481)
Supplement: Supplementary file 1 — APPENDIX S1. [file ENE-31-e16481-s006.pdf]

Supplementary Figure Legends:

Supplementary Figure 1. APC of ASIR of ischemic stroke in each GBD region from 1990 to 2021.

Supplementary Figure 2. APC of ASMR of ischemic stroke in each GBD region from 1990 to 2021.

Supplementary Figure 3. APC of ASDR of ischemic stroke in each GBD region from 1990 to 2021.

Supplementary Figure 4. Incident, Death, and Disability-Adjusted Life-Years (DALYs) Cases of Ischemic Stroke in 204 Countries and Territories.

Supplementary Figure 5. The ASIR, ASMR, and ASDR of Ischemic Stroke in 204 countries and territories.

Supplementary Figure 6. APC of ASMR for ischemic stroke attributable to each risk factor, 1990-2021.

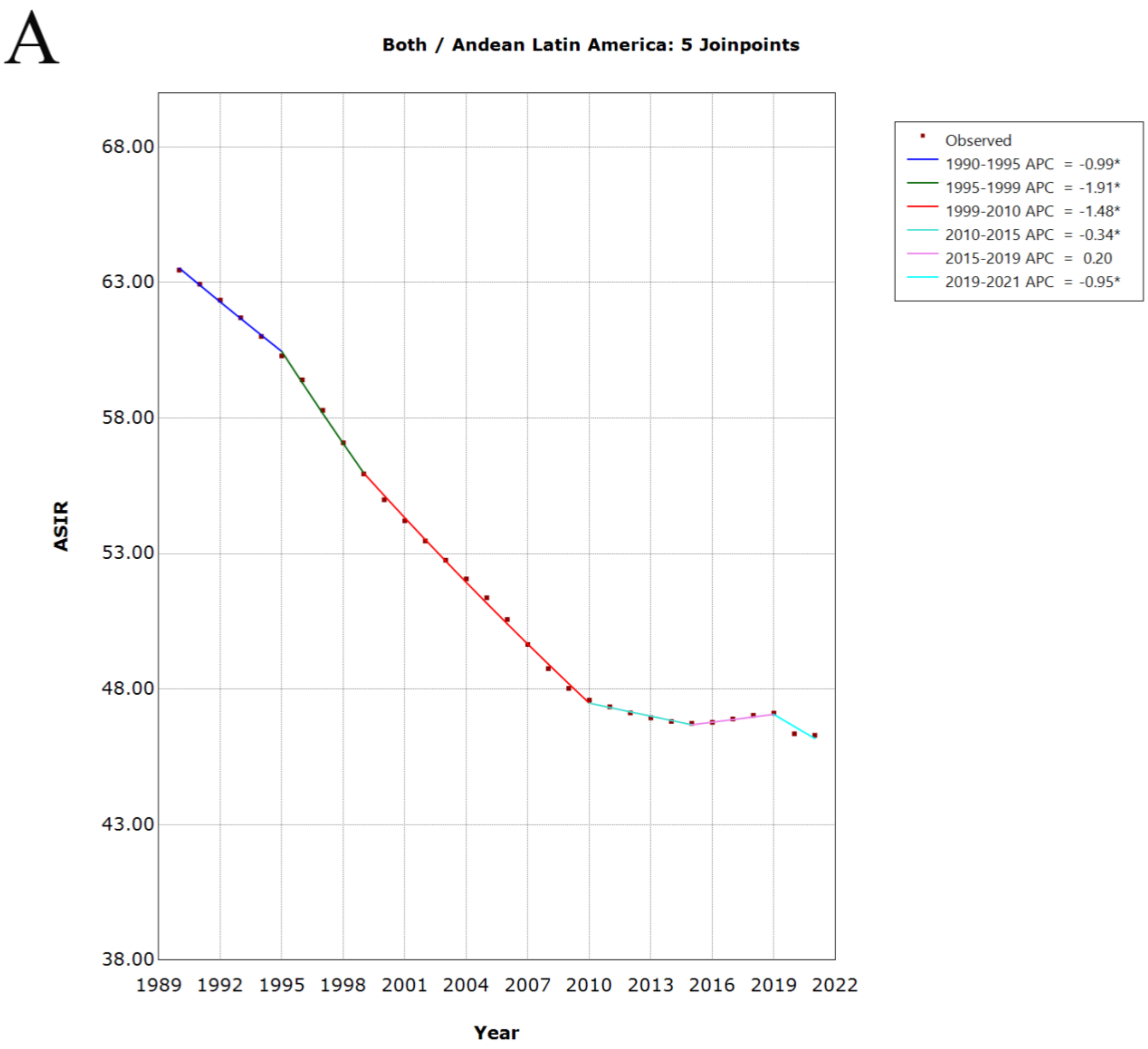

* Indicates that the Annual Percent Change (APC) is significantly different from zero at the alpha = 0.05 level
Final Selected Model: 5 Joinpoints.

B

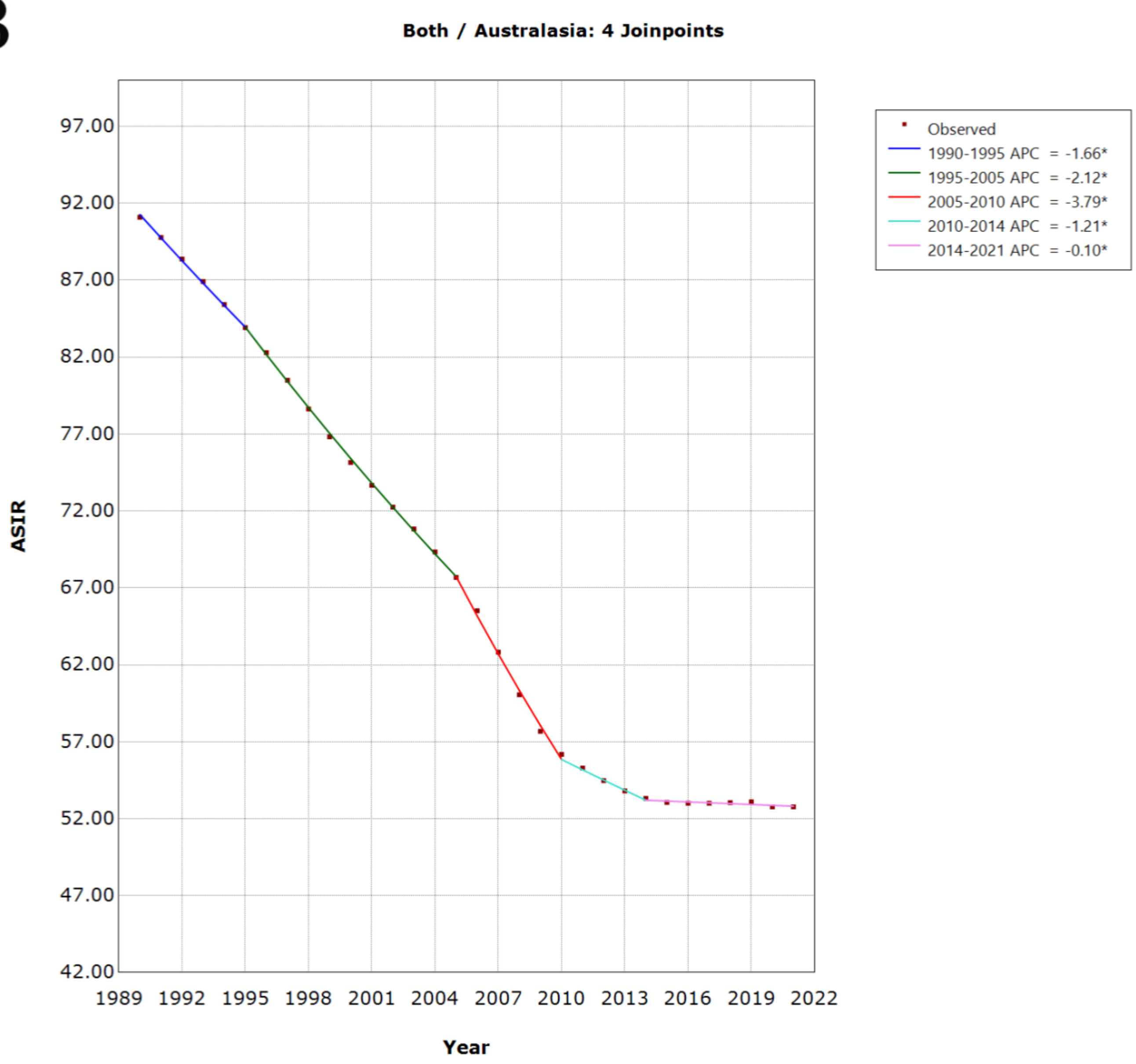

* Indicates that the Annual Percent Change (APC) is significantly different from zero at the alpha = 0.05 level
Final Selected Model: 4 Joinpoints.

C

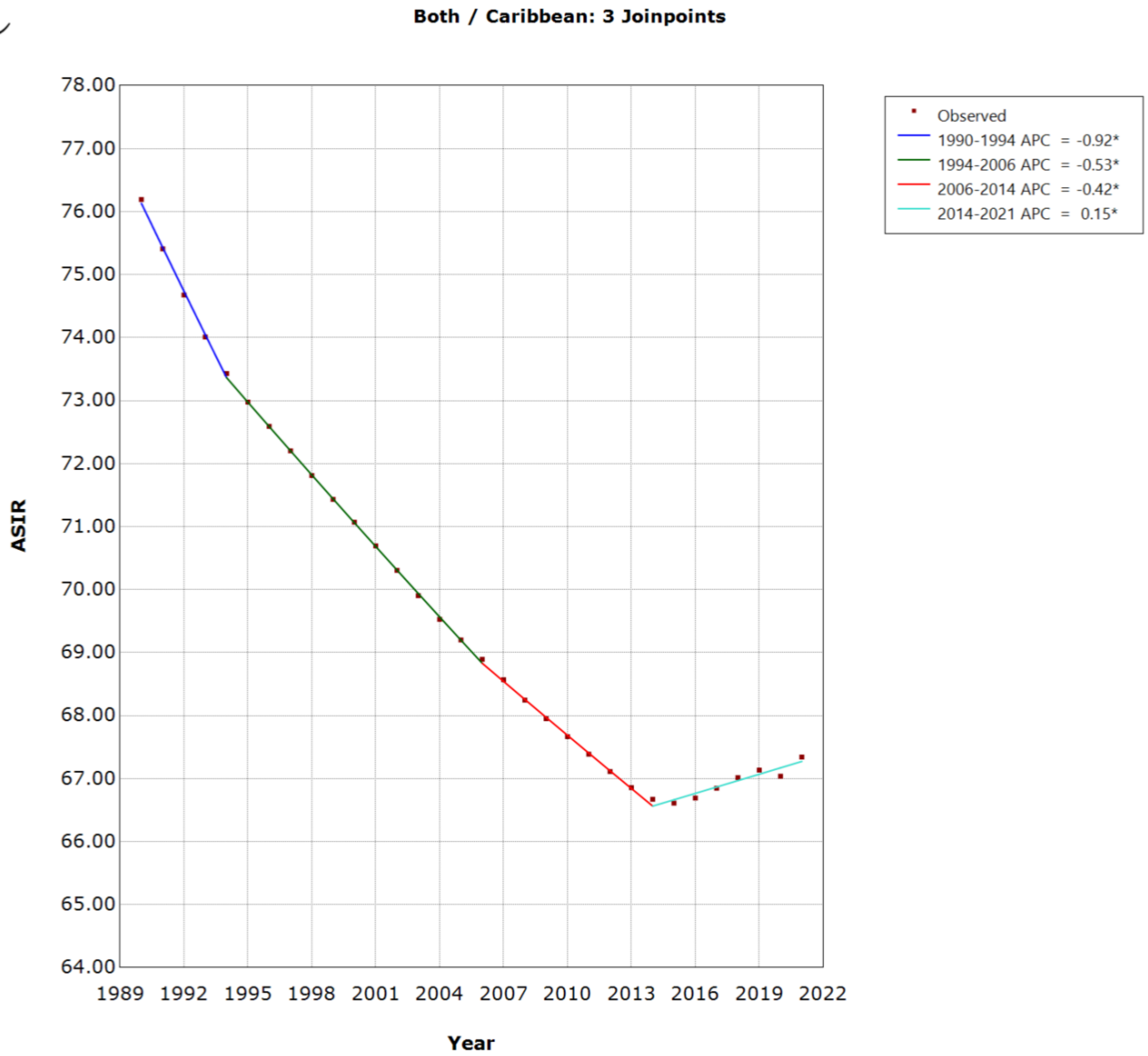

* Indicates that the Annual Percent Change (APC) is significantly different from zero at the alpha = 0.05 level
Final Selected Model: 3 Joinpoints.

D

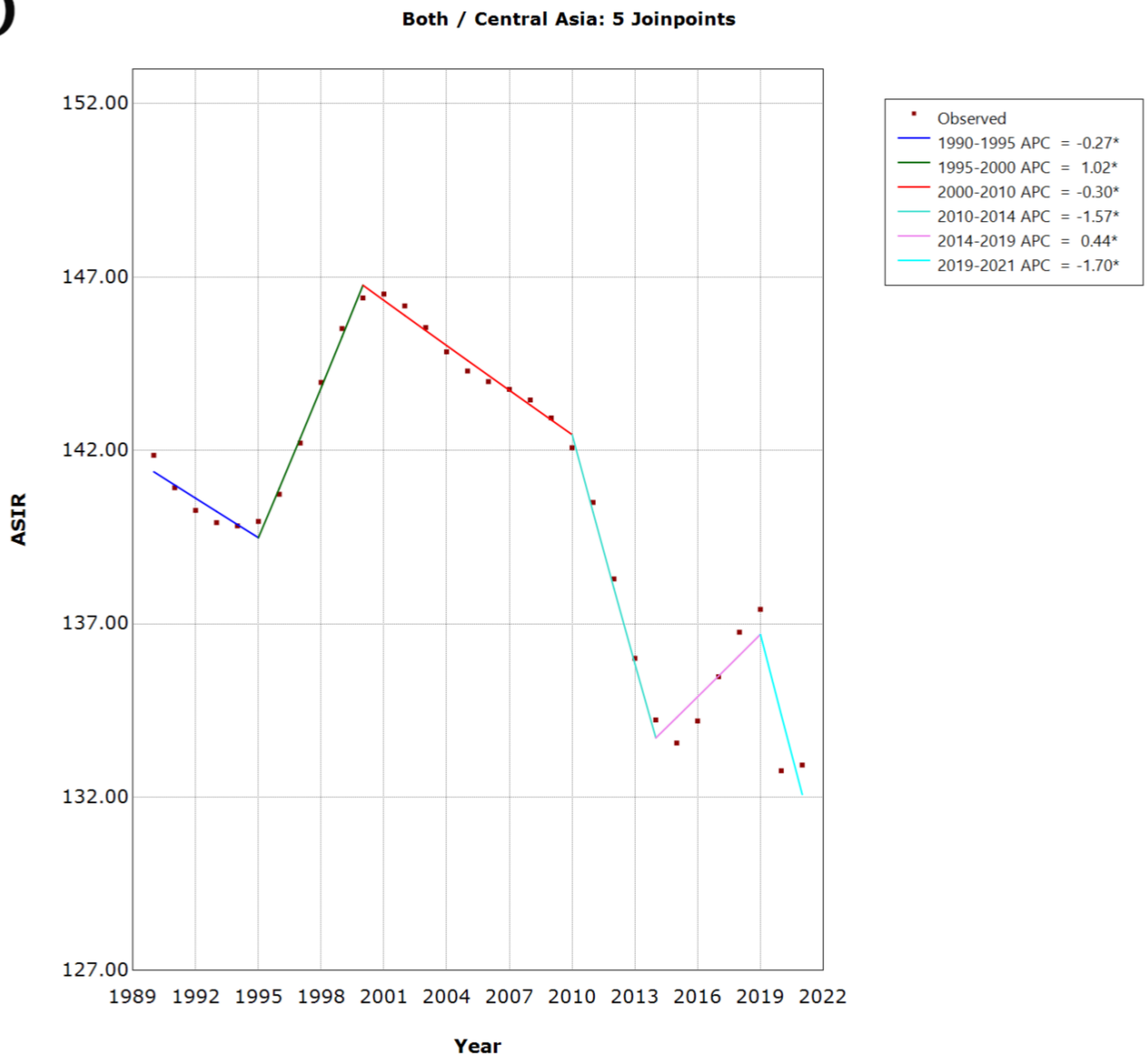

* Indicates that the Annual Percent Change (APC) is significantly different from zero at the alpha = 0.05 level
Final Selected Model: 5 Joinpoints.

E

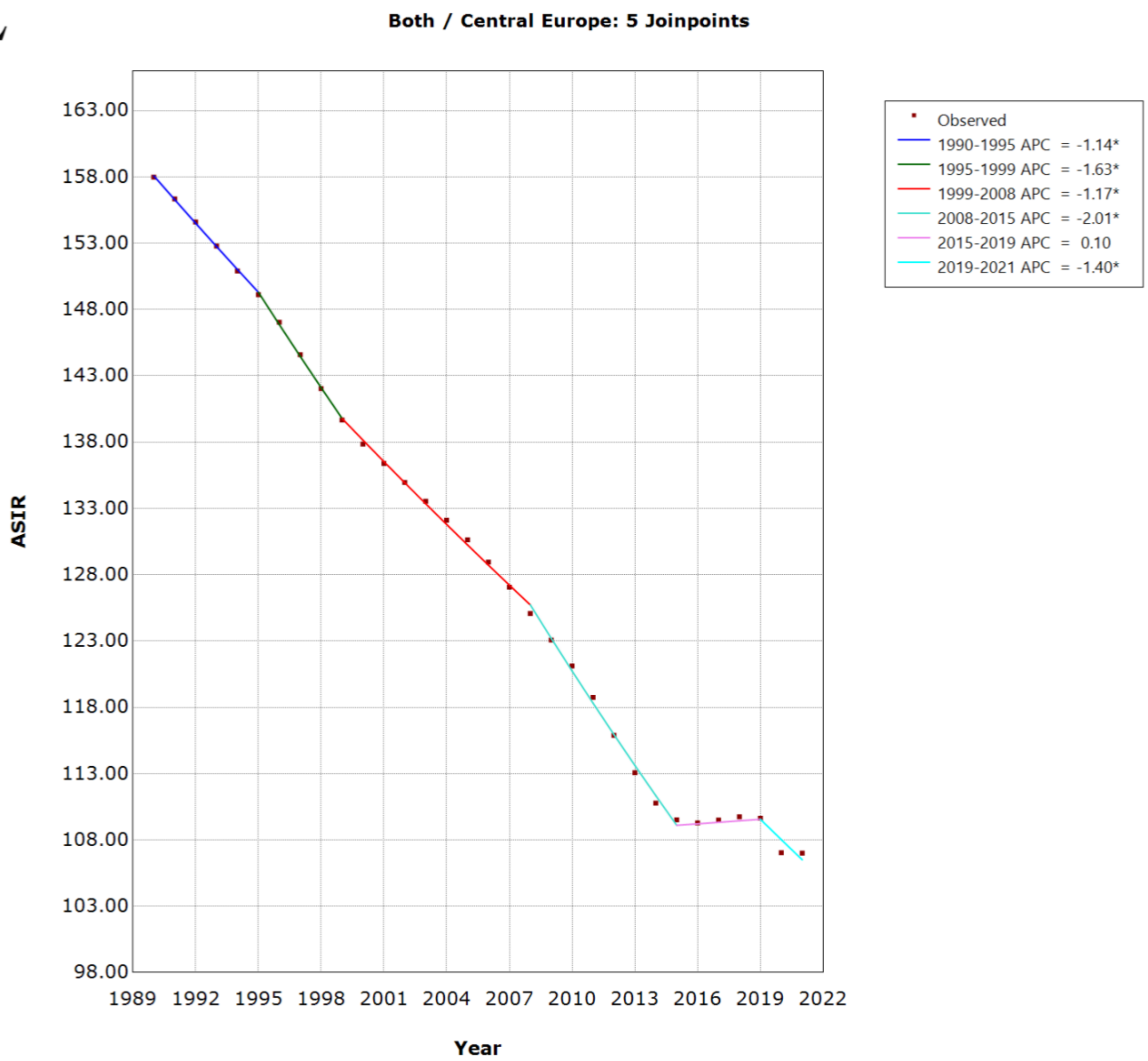

* Indicates that the Annual Percent Change (APC) is significantly different from zero at the alpha = 0.05 level
Final Selected Model: 5 Joinpoints.

F

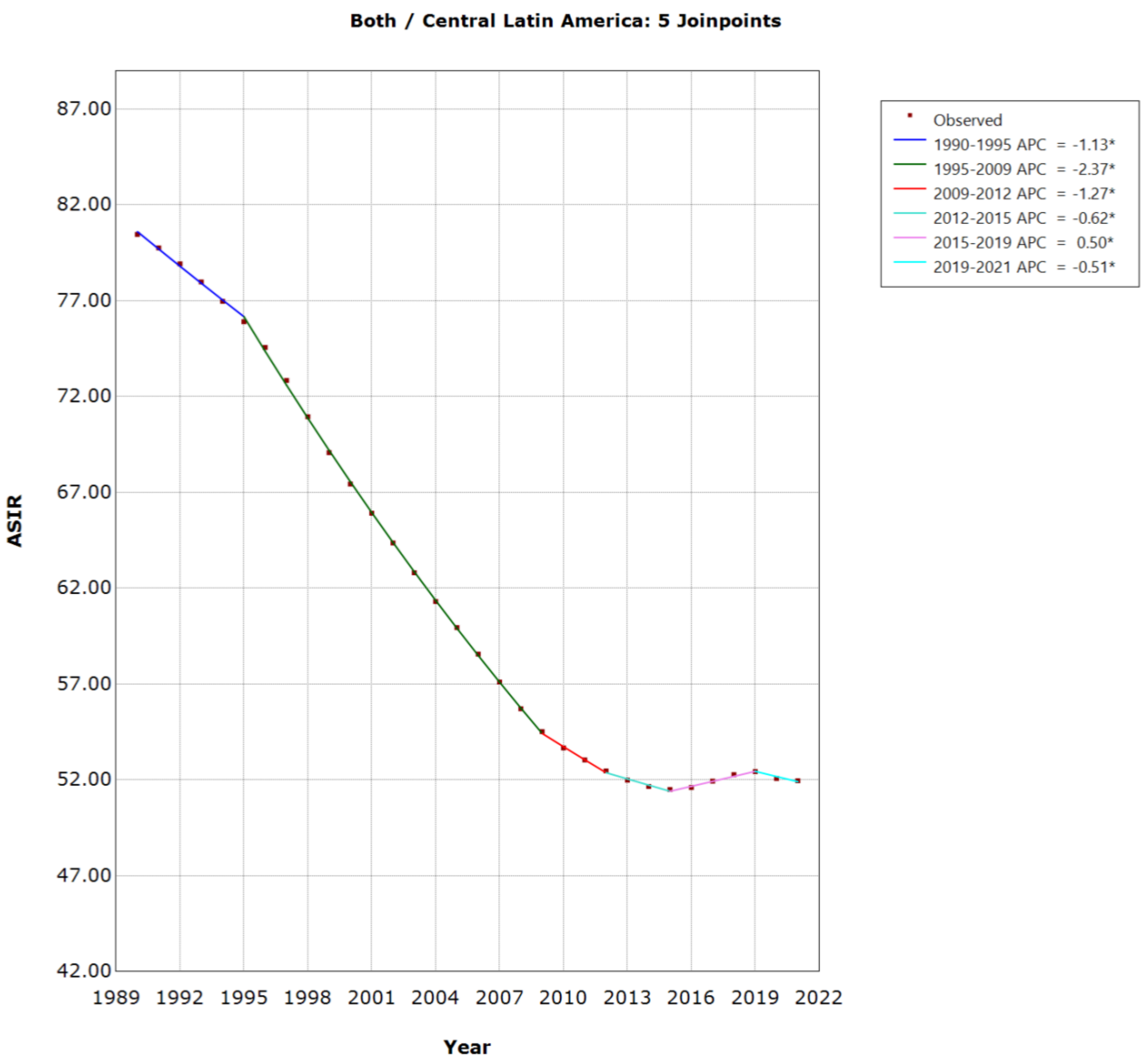

* Indicates that the Annual Percent Change (APC) is significantly different from zero at the alpha = 0.05 level
Final Selected Model: 5 Joinpoints.

G

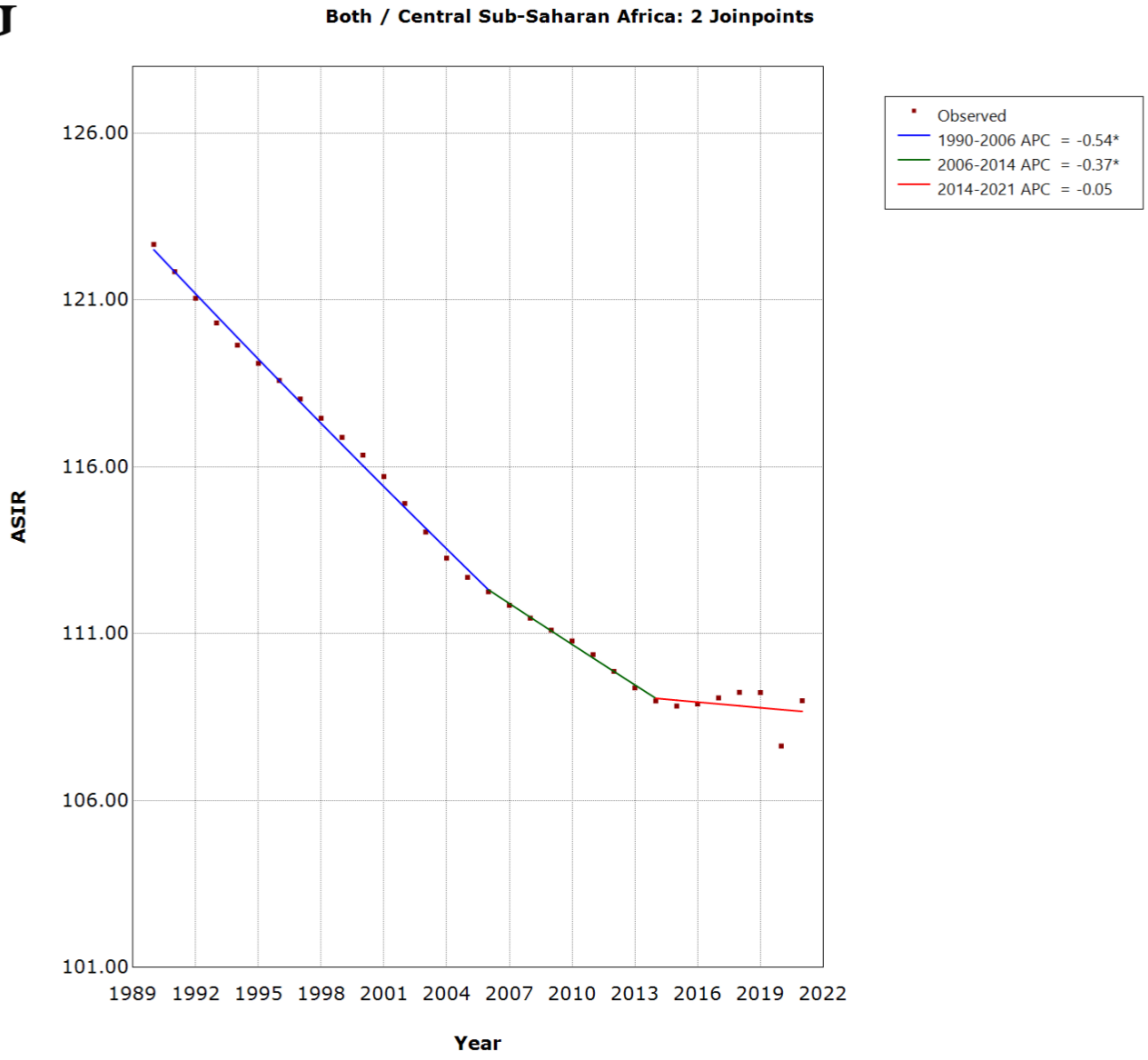

* Indicates that the Annual Percent Change (APC) is significantly different from zero at the alpha = 0.05 level
Final Selected Model: 2 Joinpoints.

H

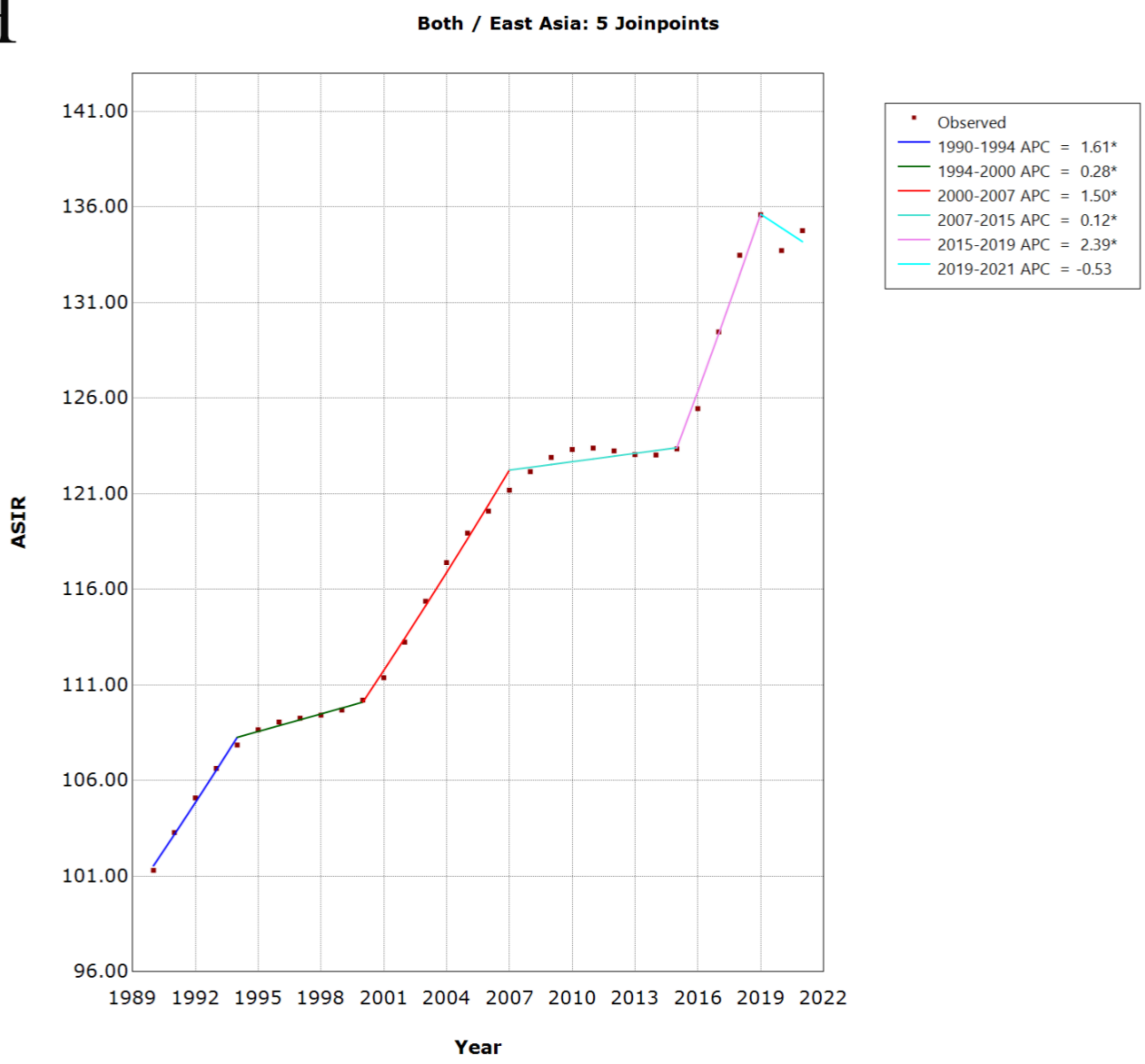

* Indicates that the Annual Percent Change (APC) is significantly different from zero at the alpha = 0.05 level
Final Selected Model: 5 Joinpoints.

I

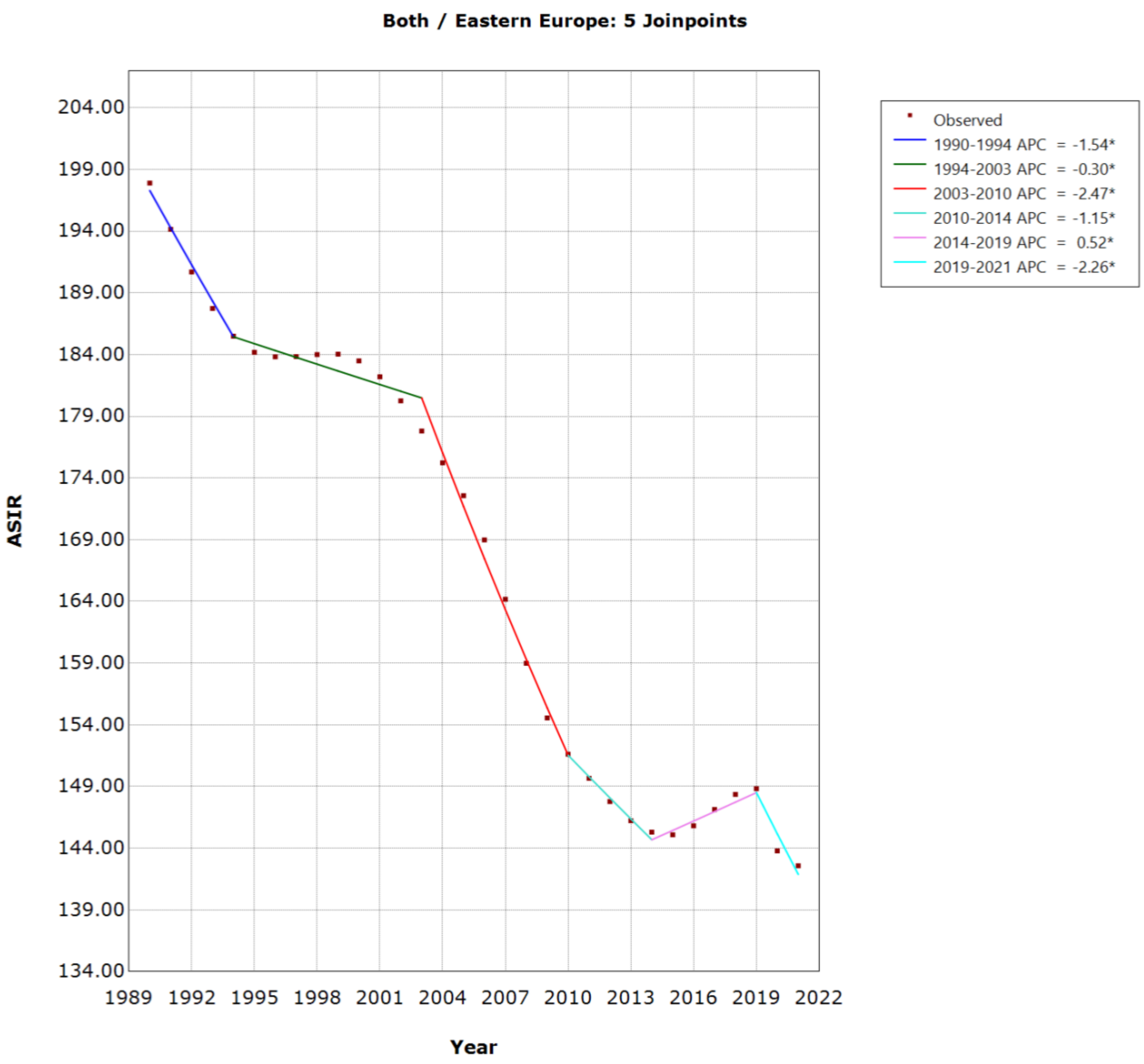

* Indicates that the Annual Percent Change (APC) is significantly different from zero at the alpha = 0.05 level
Final Selected Model: 5 Joinpoints.

J

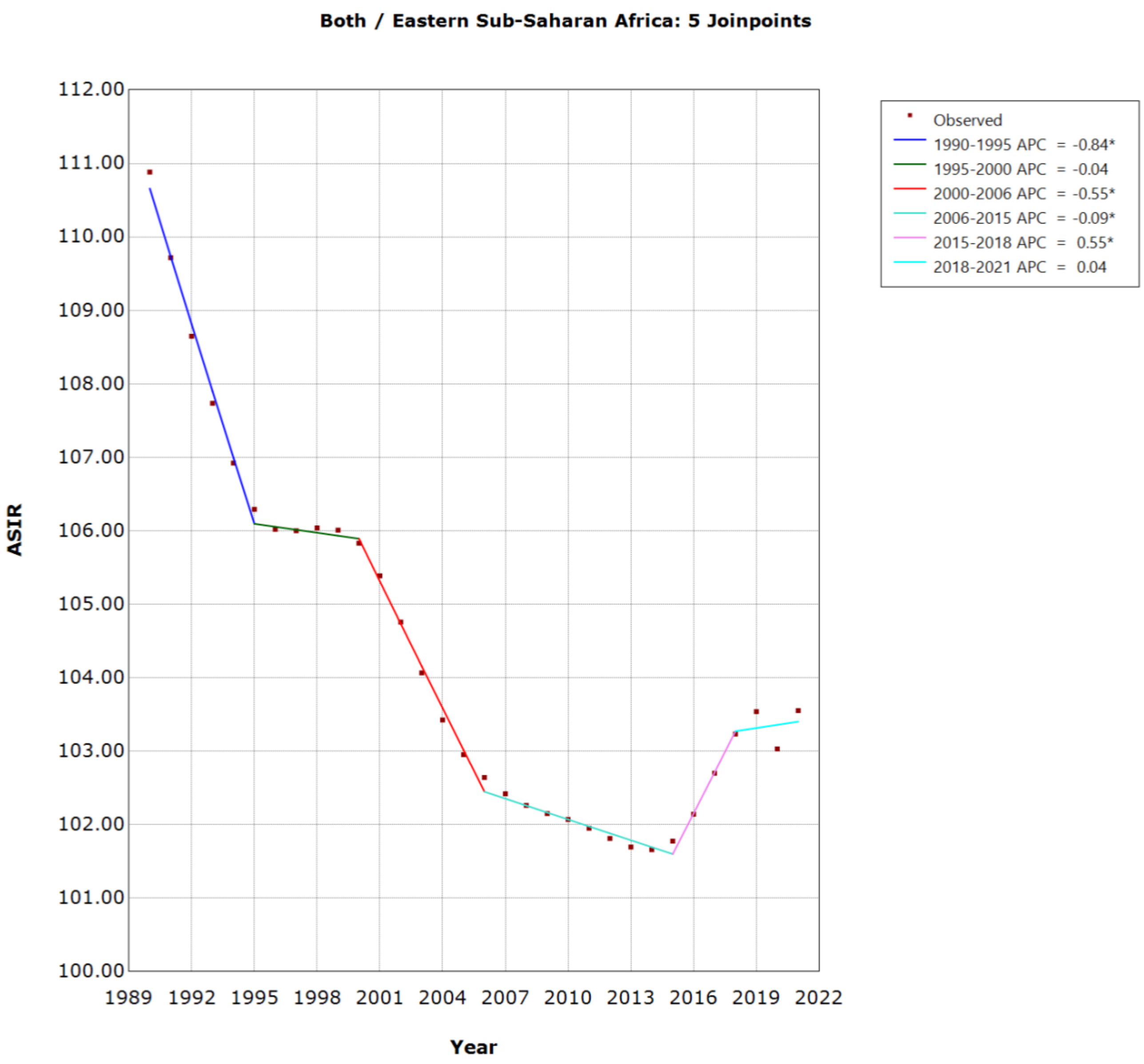

* Indicates that the Annual Percent Change (APC) is significantly different from zero at the alpha = 0.05 level
Final Selected Model: 5 Joinpoints.

K

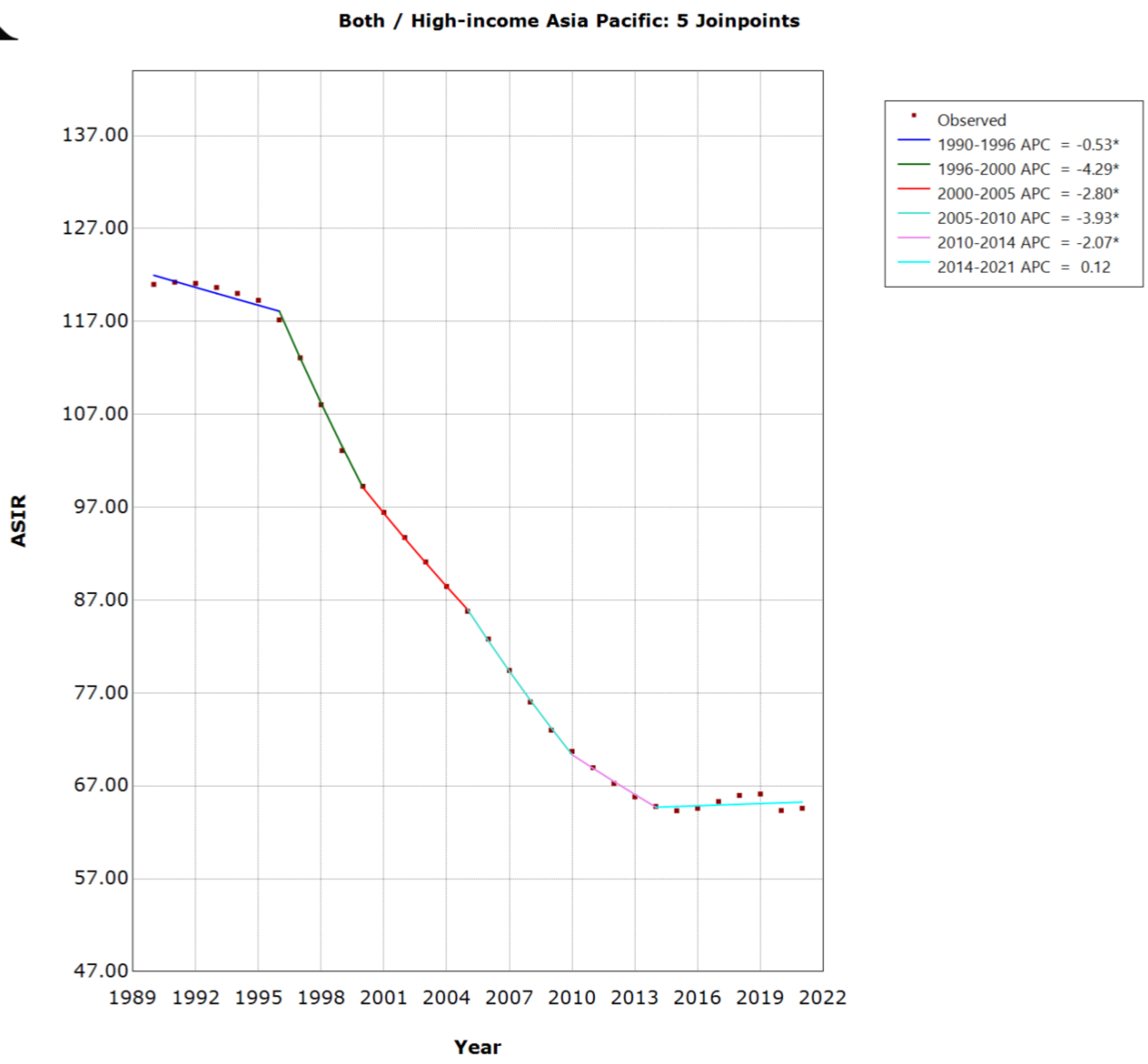

* Indicates that the Annual Percent Change (APC) is significantly different from zero at the alpha = 0.05 level
Final Selected Model: 5 Joinpoints.

L

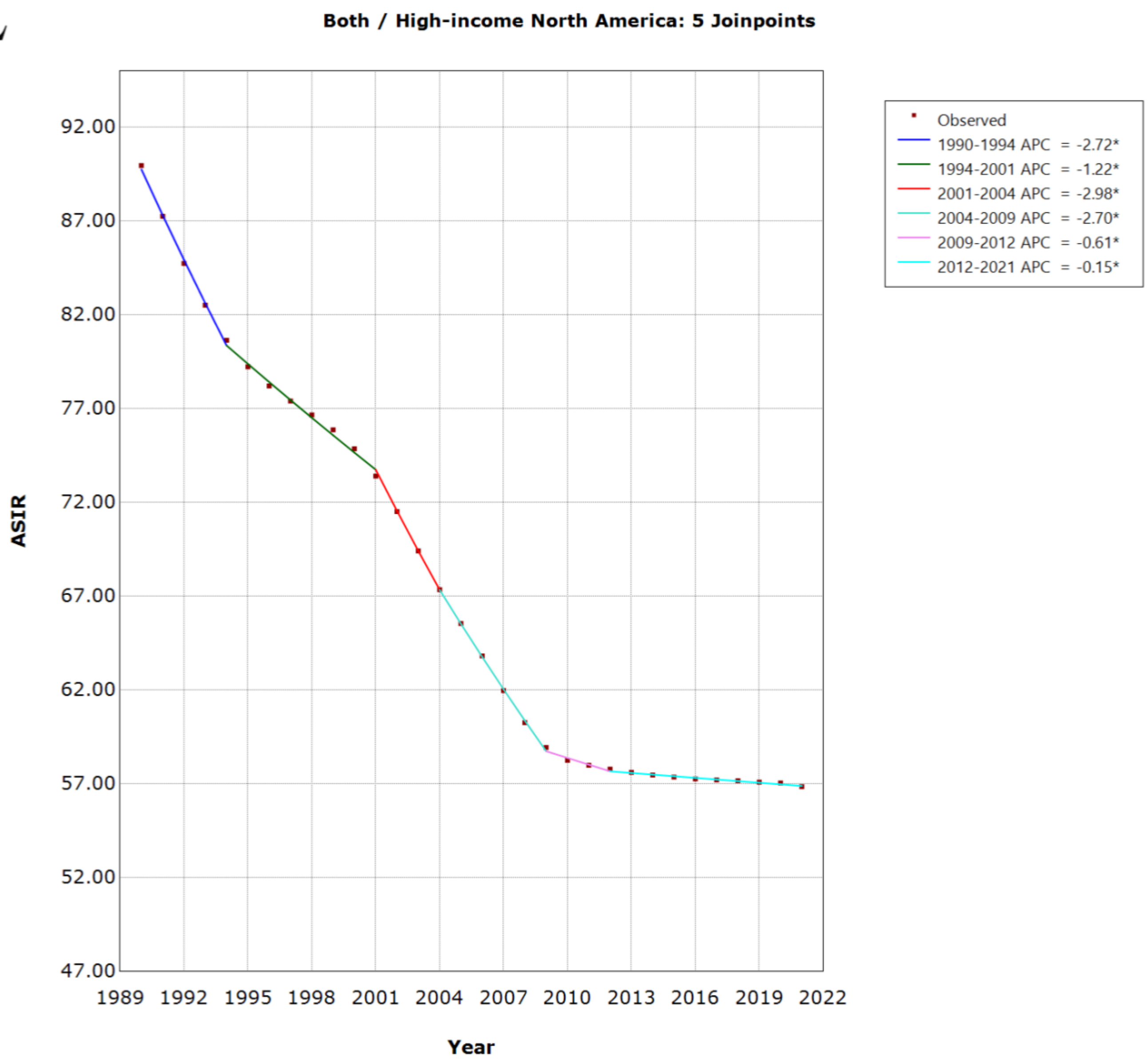

* Indicates that the Annual Percent Change (APC) is significantly different from zero at the alpha = 0.05 level
Final Selected Model: 5 Joinpoints.

M

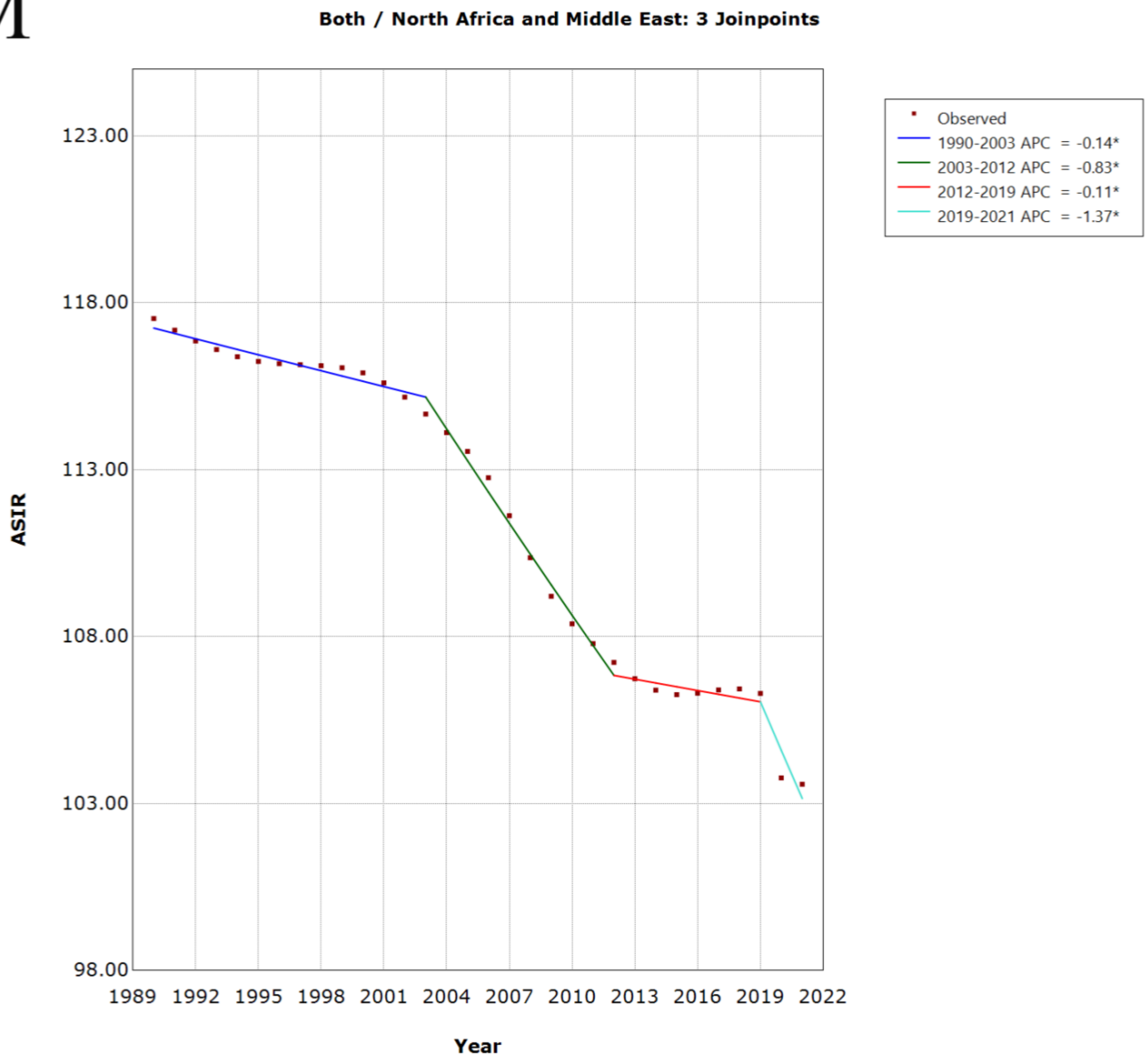

* Indicates that the Annual Percent Change (APC) is significantly different from zero at the alpha = 0.05 level
Final Selected Model: 3 Joinpoints.

N

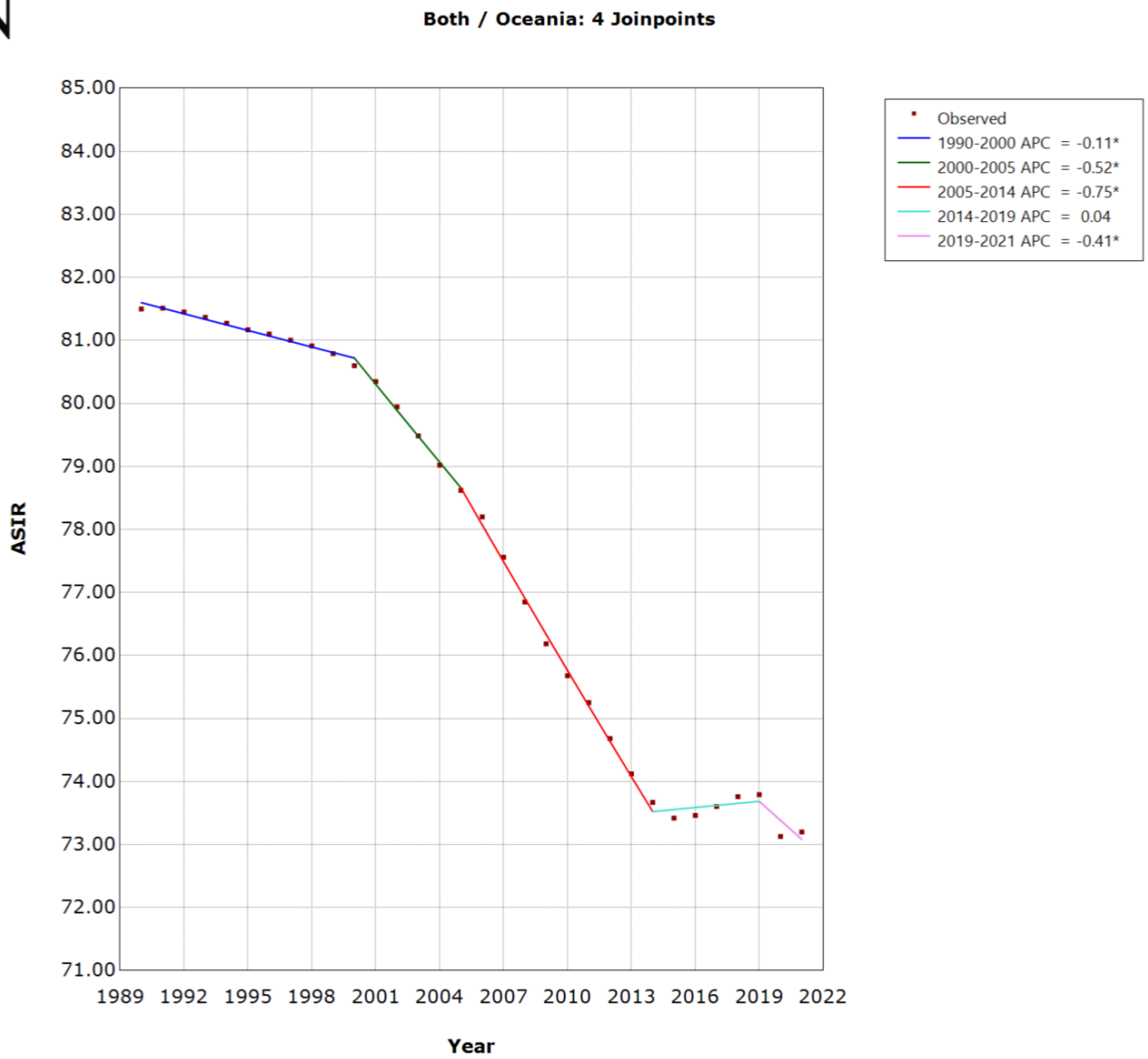

* Indicates that the Annual Percent Change (APC) is significantly different from zero at the alpha = 0.05 level
Final Selected Model: 4 Joinpoints.

O

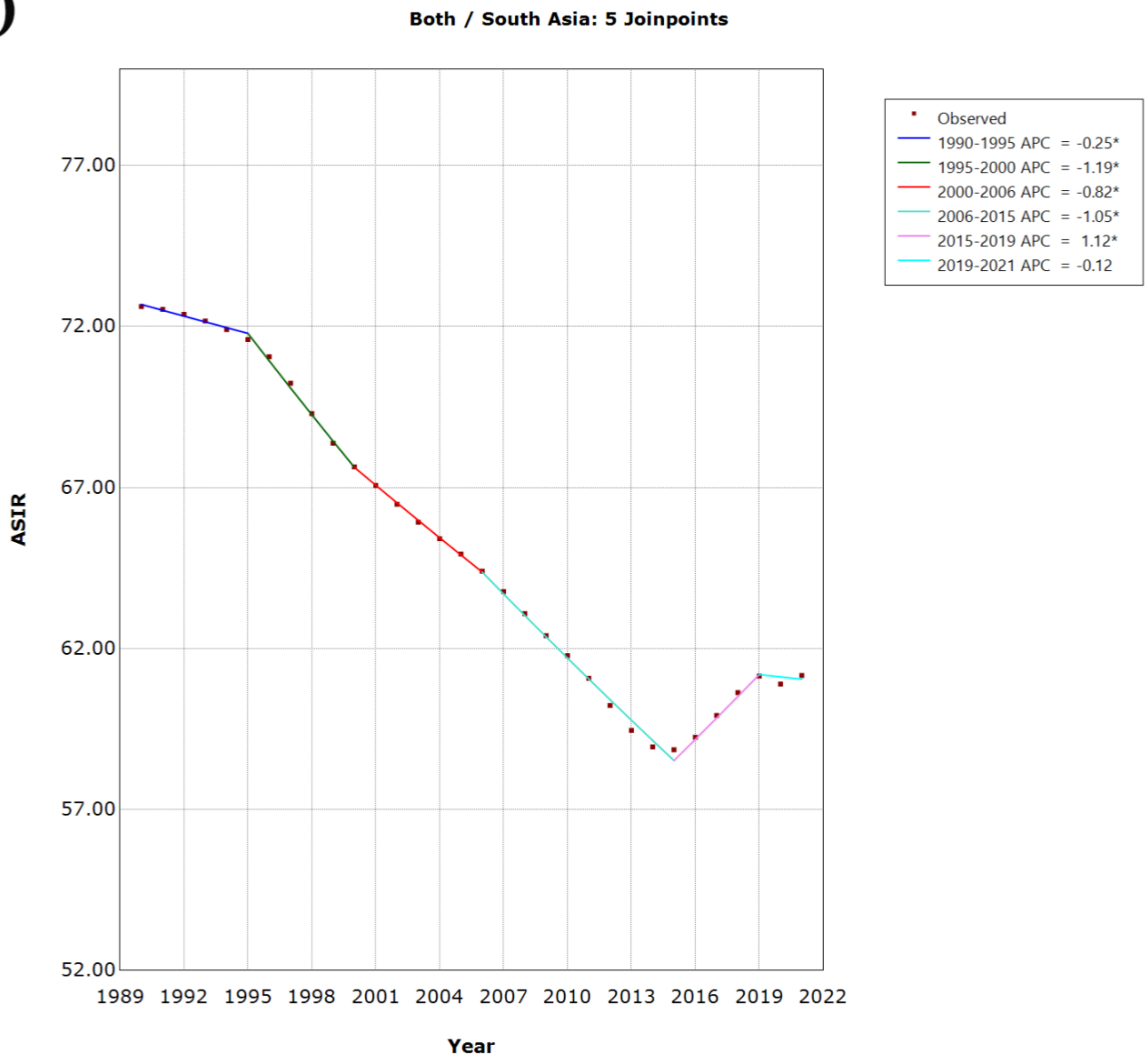

* Indicates that the Annual Percent Change (APC) is significantly different from zero at the alpha = 0.05 level
Final Selected Model: 5 Joinpoints.

P

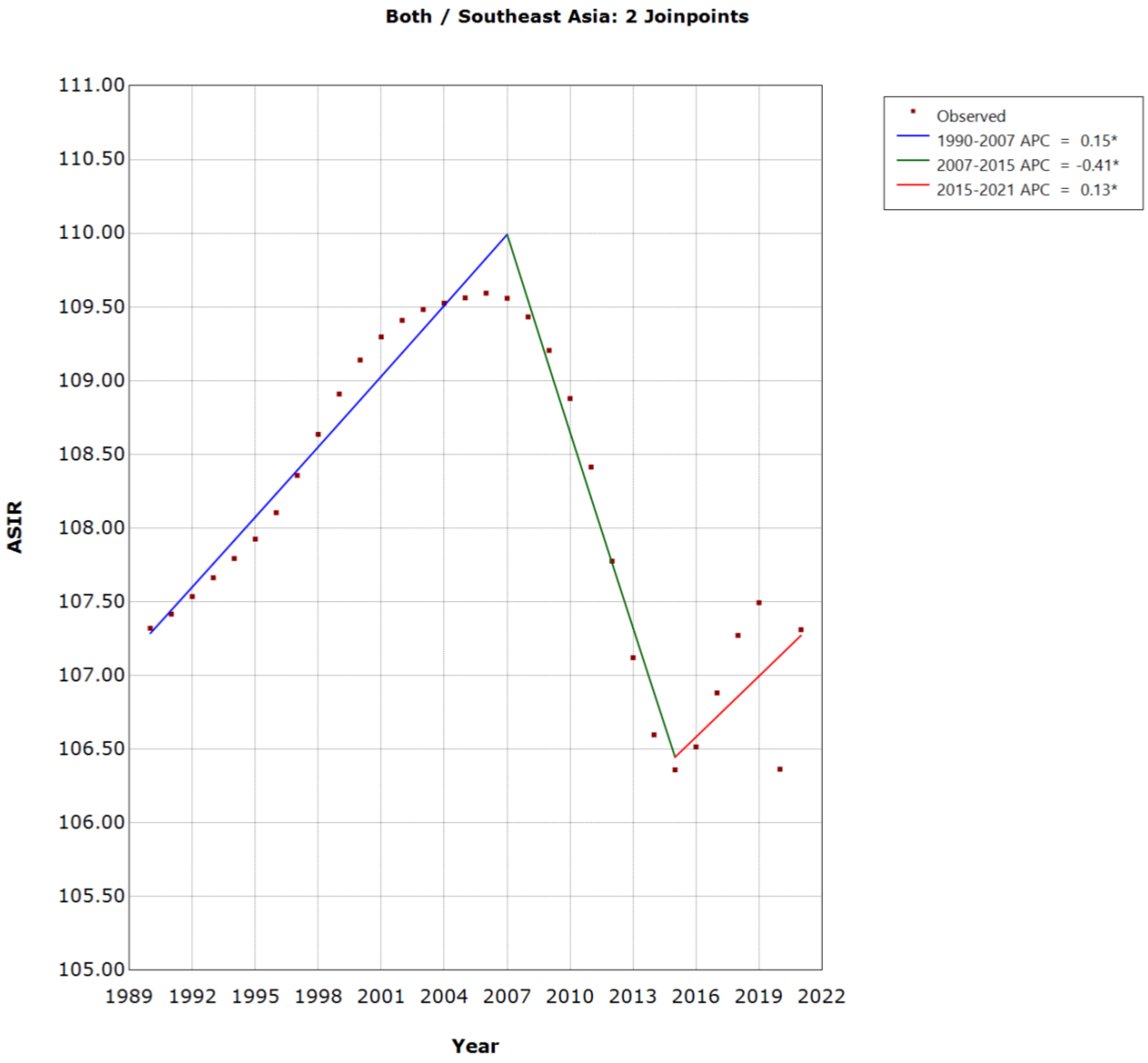

* Indicates that the Annual Percent Change (APC) is significantly different from zero at the alpha = 0.05 level
Final Selected Model: 2 Joinpoints.

Q

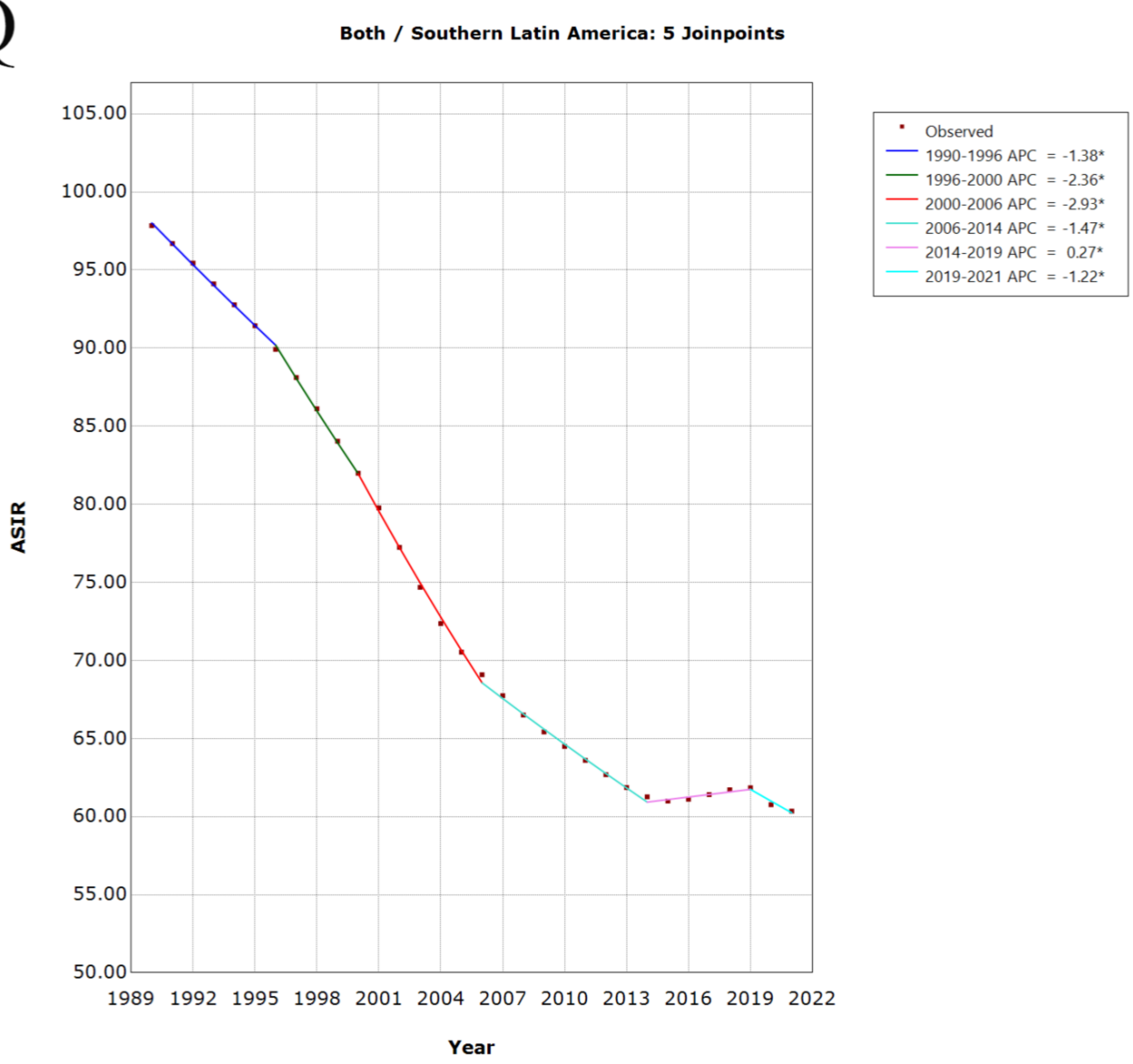

* Indicates that the Annual Percent Change (APC) is significantly different from zero at the alpha = 0.05 level
Final Selected Model: 5 Joinpoints.

R

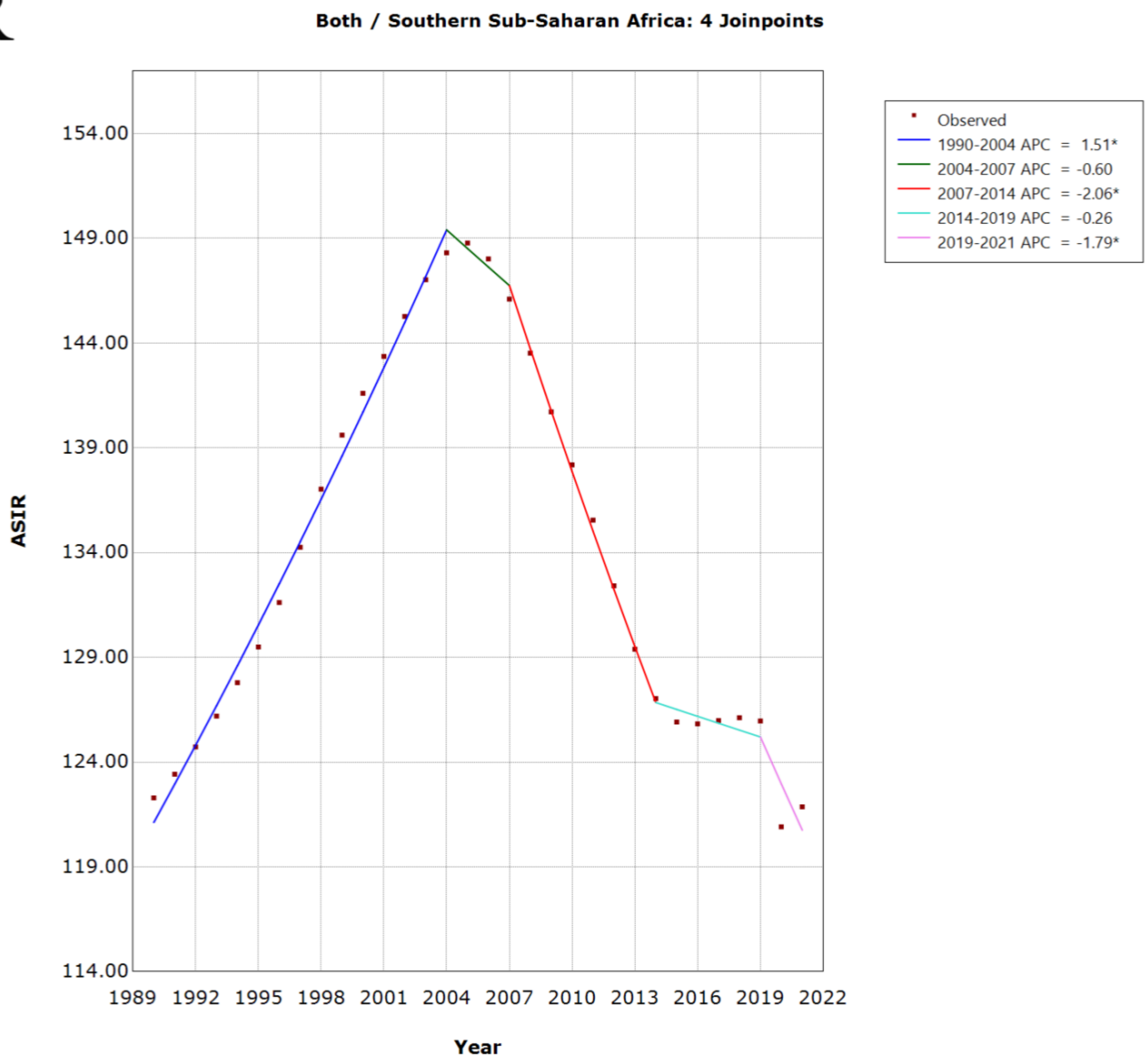

* Indicates that the Annual Percent Change (APC) is significantly different from zero at the alpha = 0.05 level
Final Selected Model: 4 Joinpoints.

S

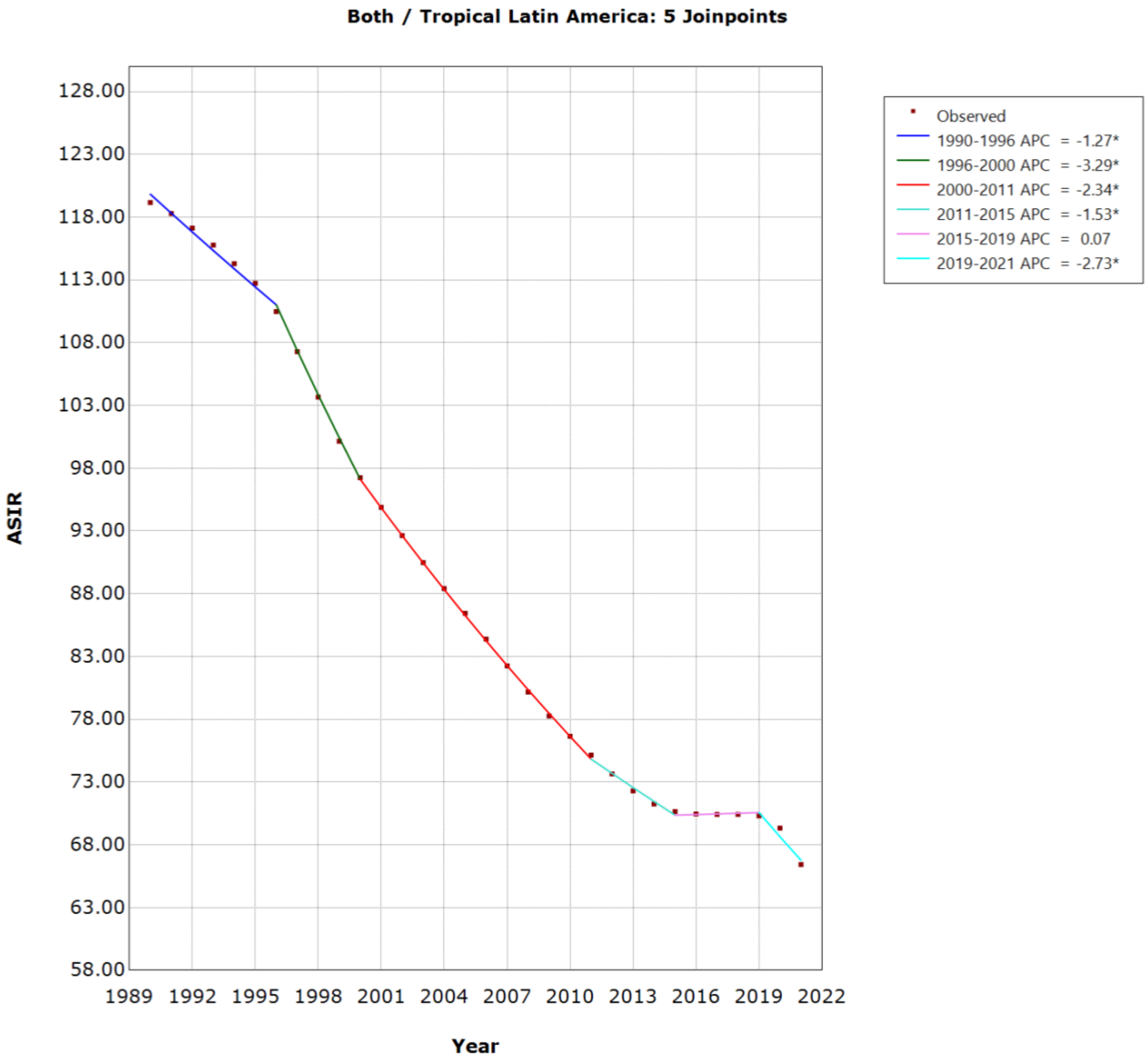

* Indicates that the Annual Percent Change (APC) is significantly different from zero at the alpha = 0.05 level
Final Selected Model: 5 Joinpoints.

T

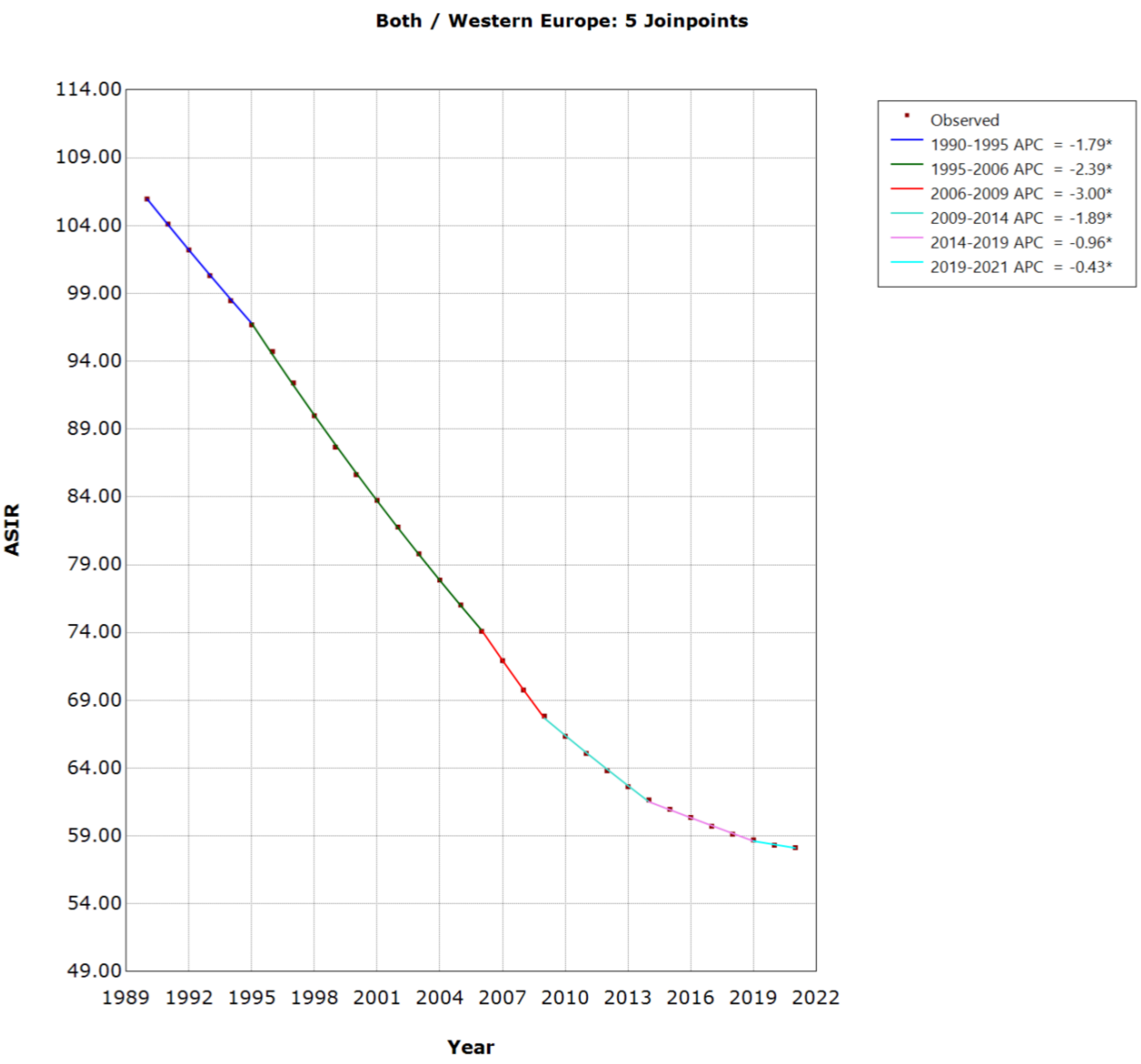

* Indicates that the Annual Percent Change (APC) is significantly different from zero at the alpha = 0.05 level
Final Selected Model: 5 Joinpoints.

U

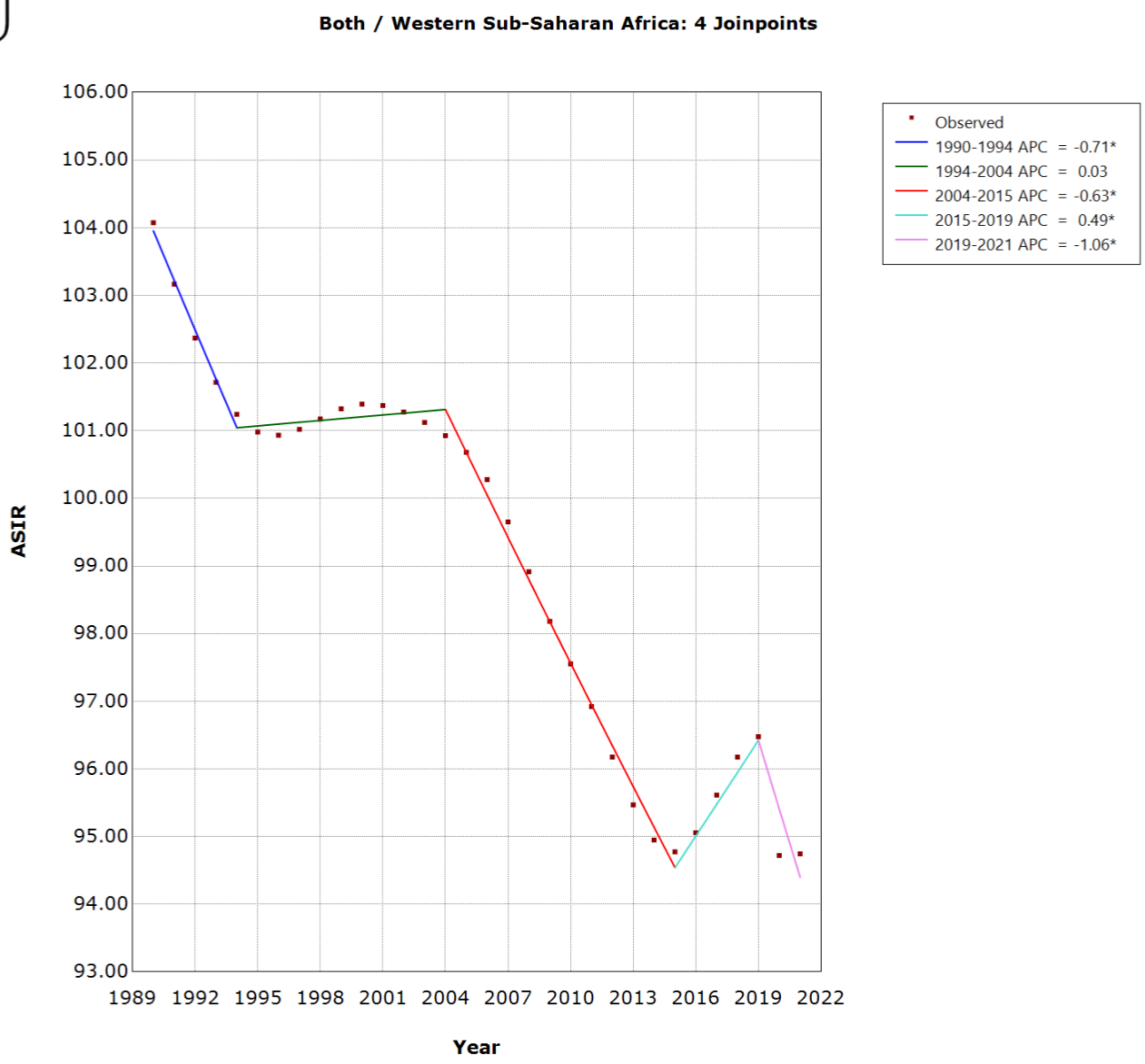

* Indicates that the Annual Percent Change (APC) is significantly different from zero at the alpha = 0.05 level
Final Selected Model: 4 Joinpoints.

Supplementary Figure 1. APC of ASIR of ischemic stroke in each GBD region from 1990 to 2021. A. APC of ASIR of ischemic stroke in Andean Latin America from 1990 to 2021. B. APC of ASIR of ischemic stroke in Australasia from 1990 to 2021. C. APC of ASIR of ischemic stroke in Caribbean from 1990 to 2021. D. APC of ASIR of ischemic stroke in Central Asia from 1990 to 2021. E. APC of ASIR of ischemic stroke in Central Europe from 1990 to 2021. F. APC of ASIR of ischemic stroke in Central Latin America from 1990 to 2021. G. APC of ASIR of ischemic stroke in Central Sub-Saharan Africa from 1990 to 2021. H. APC of ASIR of ischemic stroke in East Asia from 1990 to 2021. I. APC of ASIR of ischemic stroke in Eastern Europe from 1990 to 2021. J. APC of ASIR of ischemic stroke in Eastern Sub-Saharan Africa from 1990 to 2021. K. APC of ASIR of ischemic stroke in High-income Asia Pacific from 1990 to 2021. L. APC of ASIR of ischemic stroke in High-income North America from 1990 to 2021. M. APC of ASIR of ischemic stroke in North Africa and Middle East from 1990 to 2021. N. APC of ASIR of ischemic stroke in Oceania from 1990 to 2021. O. APC of ASIR of ischemic stroke in South Asia from 1990 to 2021. P. APC of ASIR of ischemic stroke in Southeast Asia from 1990 to 2021. Q. APC of ASIR of ischemic stroke in Southern Latin America from 1990 to 2021. R. APC of ASIR of ischemic stroke in Southern Sub-Saharan Africa from 1990 to 2021. S. APC of ASIR of ischemic stroke in Tropical Latin America from 1990 to 2021. T. APC of ASIR of ischemic stroke in each Western Europe 1990 to 2021. U. APC of ASIR of ischemic stroke in each Western Sub-Saharan Africa 1990 to 2021. APC = Annual percent change. ASIR = Age-standardized incidence rate.

A

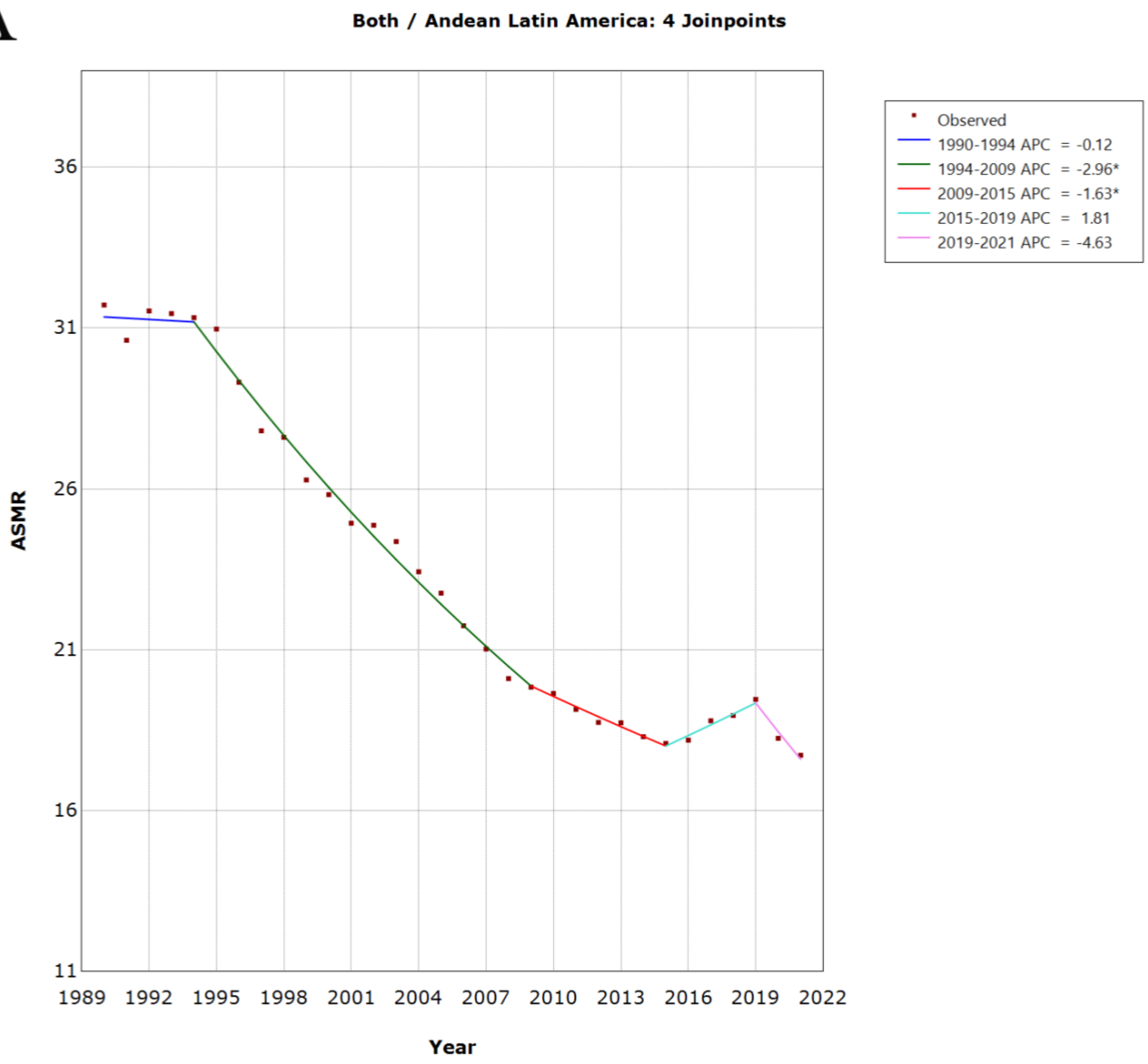

* Indicates that the Annual Percent Change (APC) is significantly different from zero at the alpha = 0.05 level
Final Selected Model: 4 Joinpoints.

B

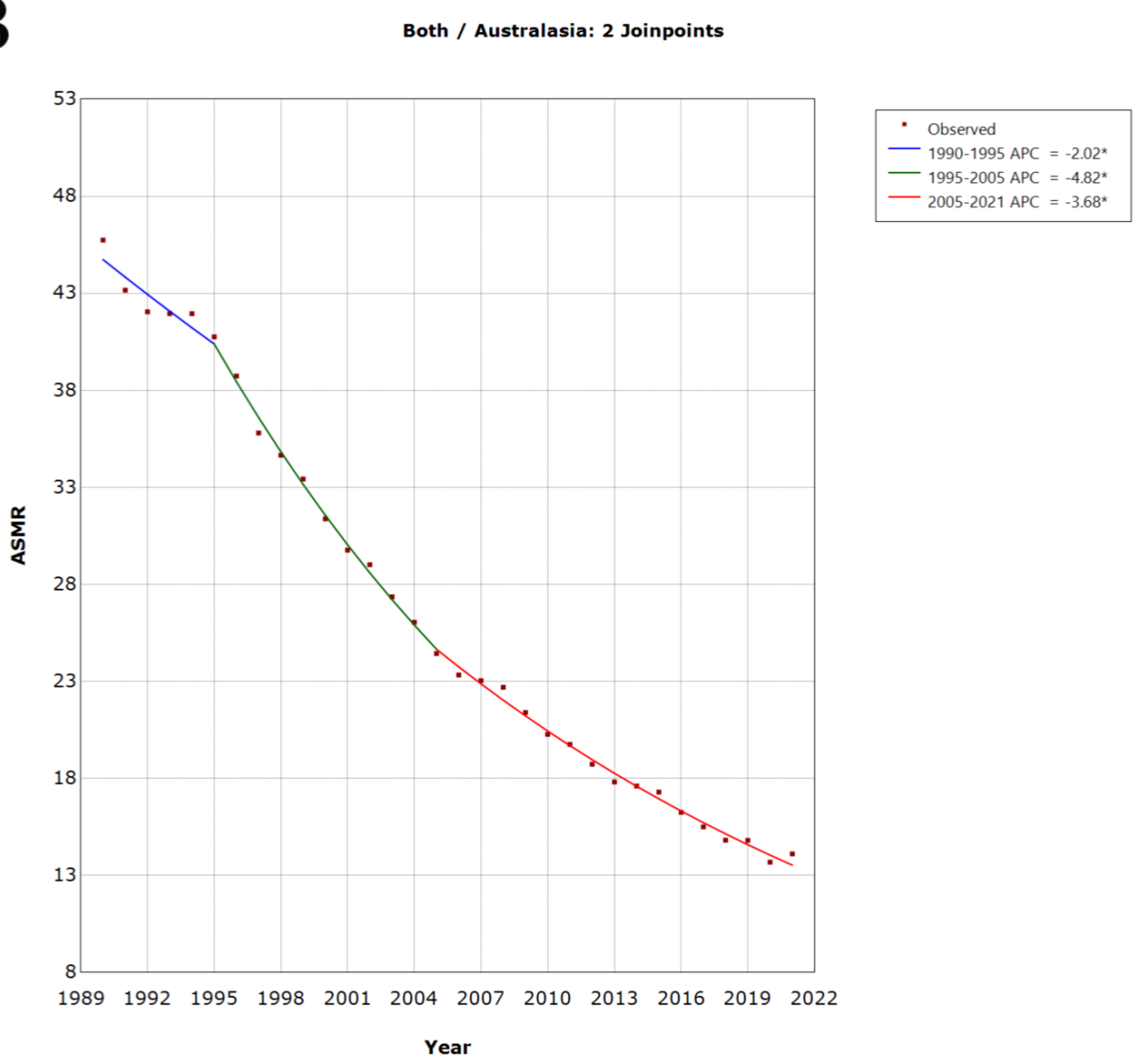

* Indicates that the Annual Percent Change (APC) is significantly different from zero at the alpha = 0.05 level
Final Selected Model: 2 Joinpoints.

C

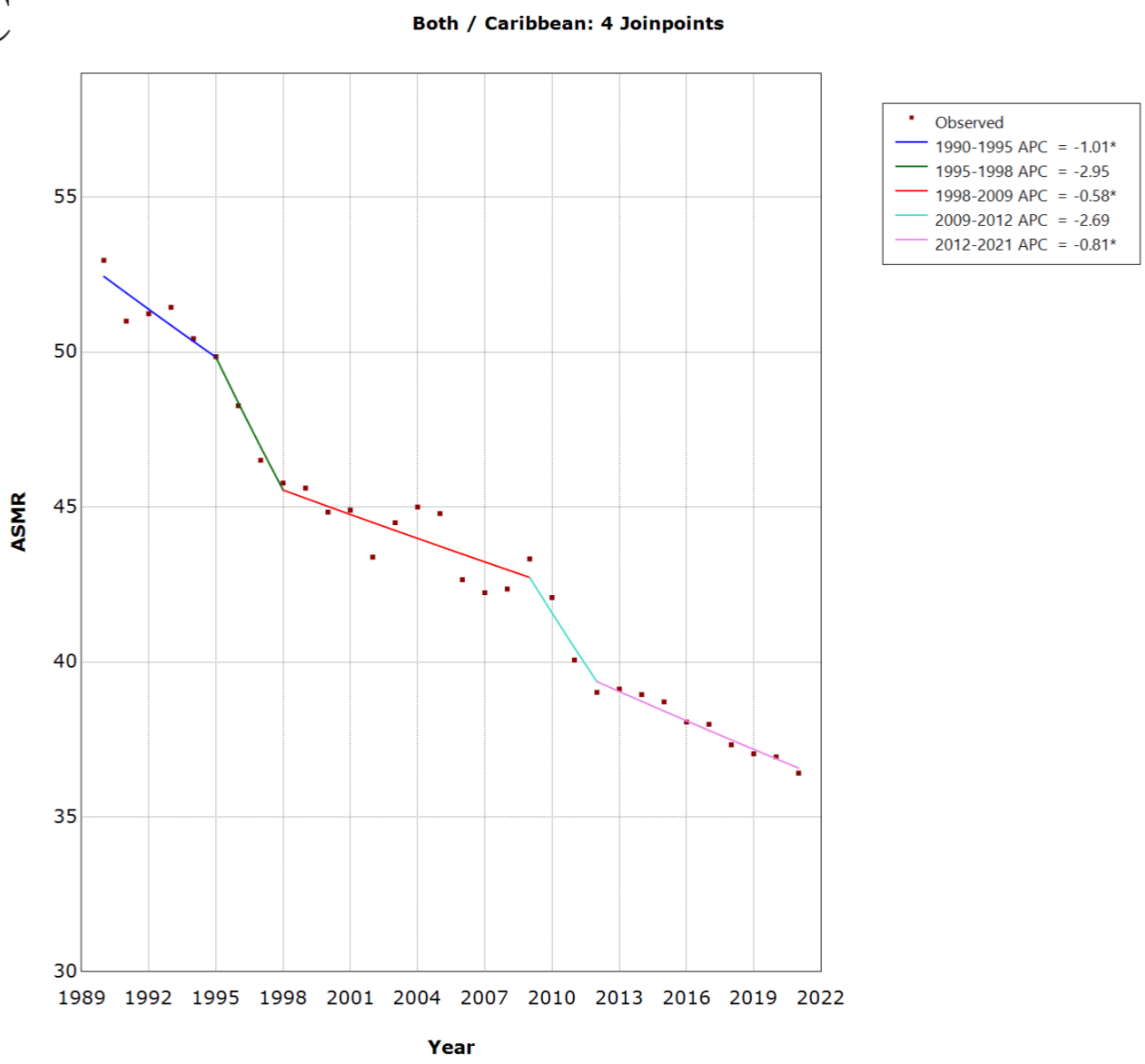

* Indicates that the Annual Percent Change (APC) is significantly different from zero at the alpha = 0.05 level
Final Selected Model: 4 Joinpoints.

D

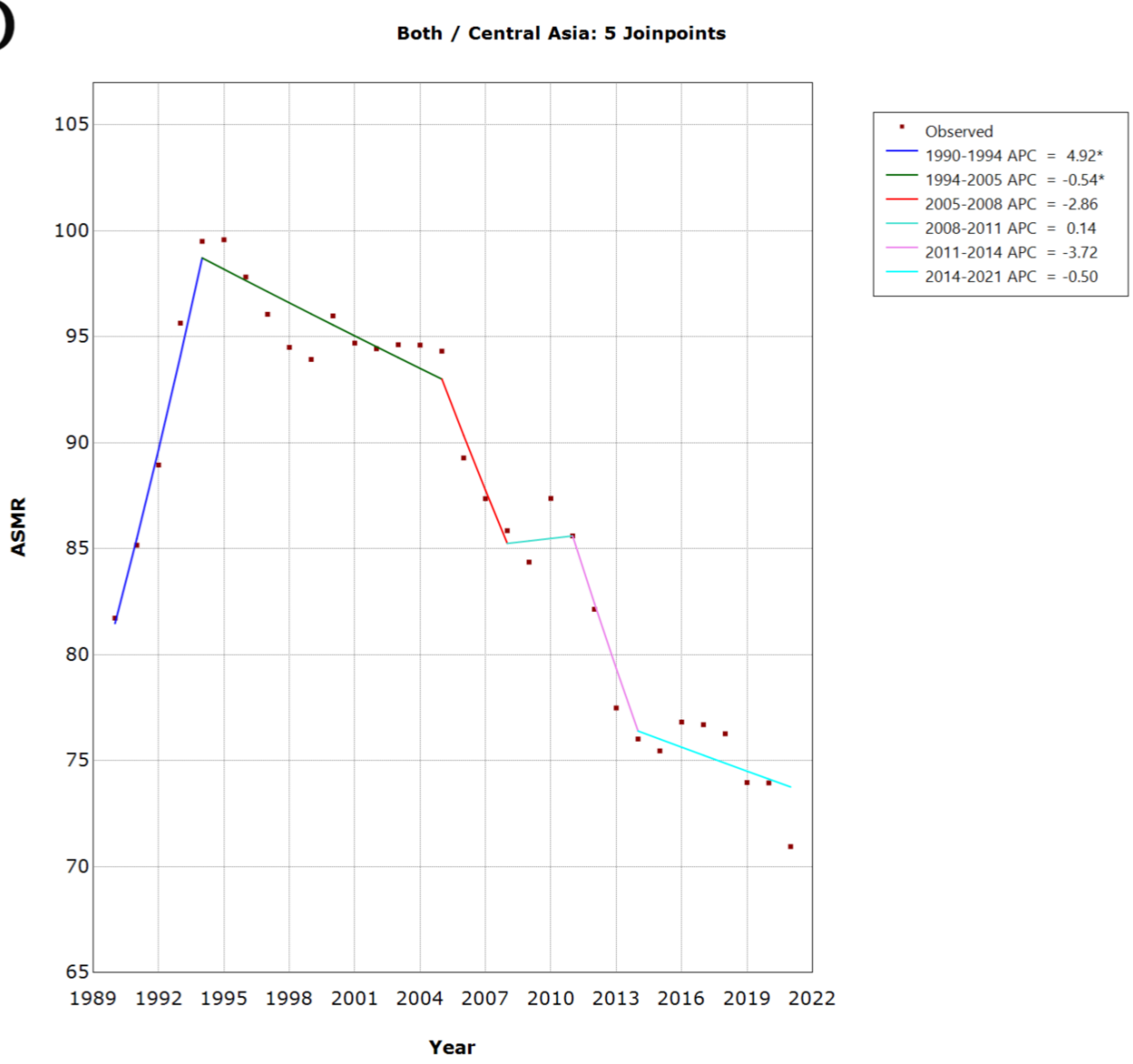

* Indicates that the Annual Percent Change (APC) is significantly different from zero at the alpha = 0.05 level
Final Selected Model: 5 Joinpoints.

E

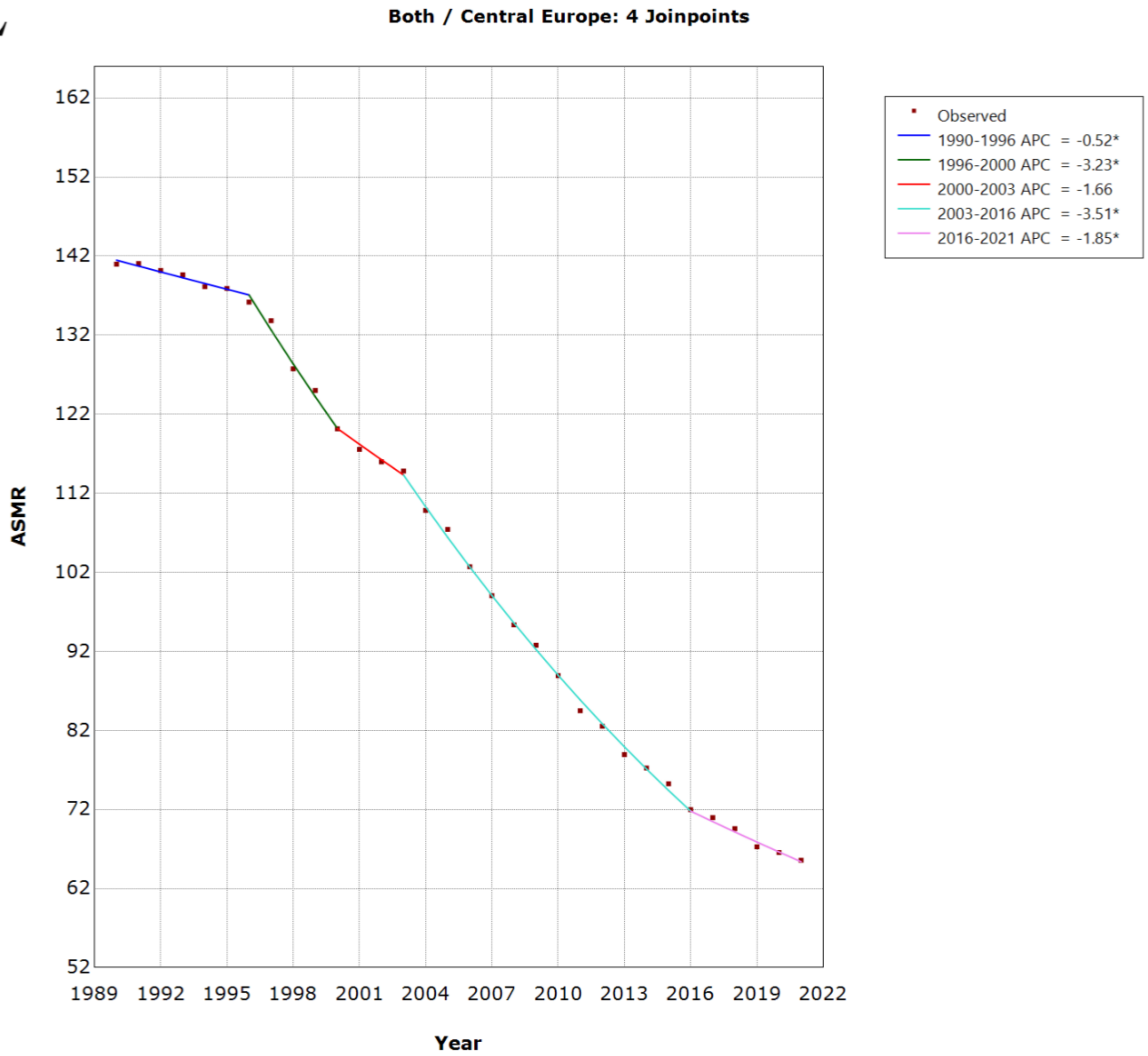

* Indicates that the Annual Percent Change (APC) is significantly different from zero at the alpha = 0.05 level
Final Selected Model: 4 Joinpoints.

F

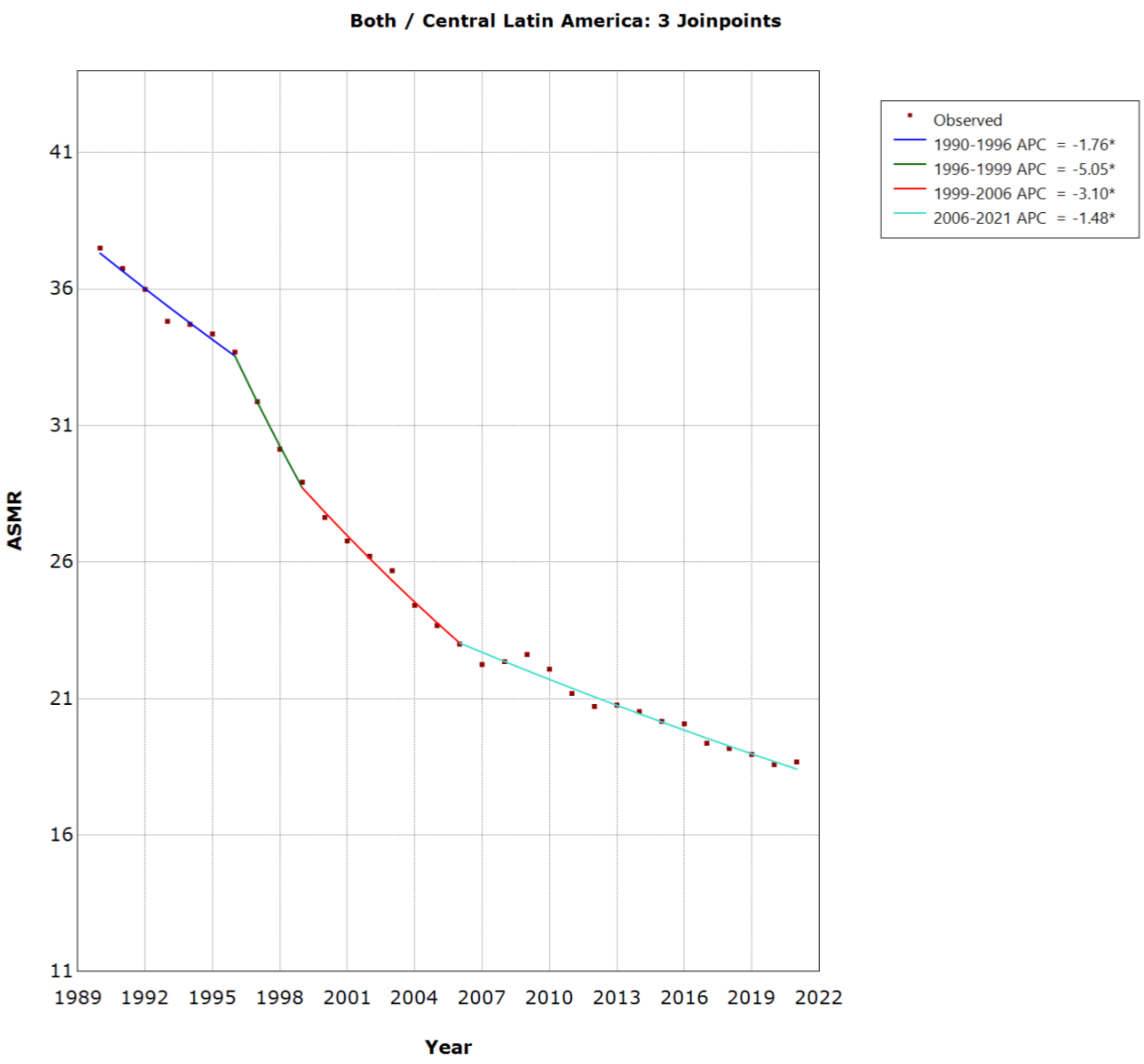

* Indicates that the Annual Percent Change (APC) is significantly different from zero at the alpha = 0.05 level
Final Selected Model: 3 Joinpoints.

G

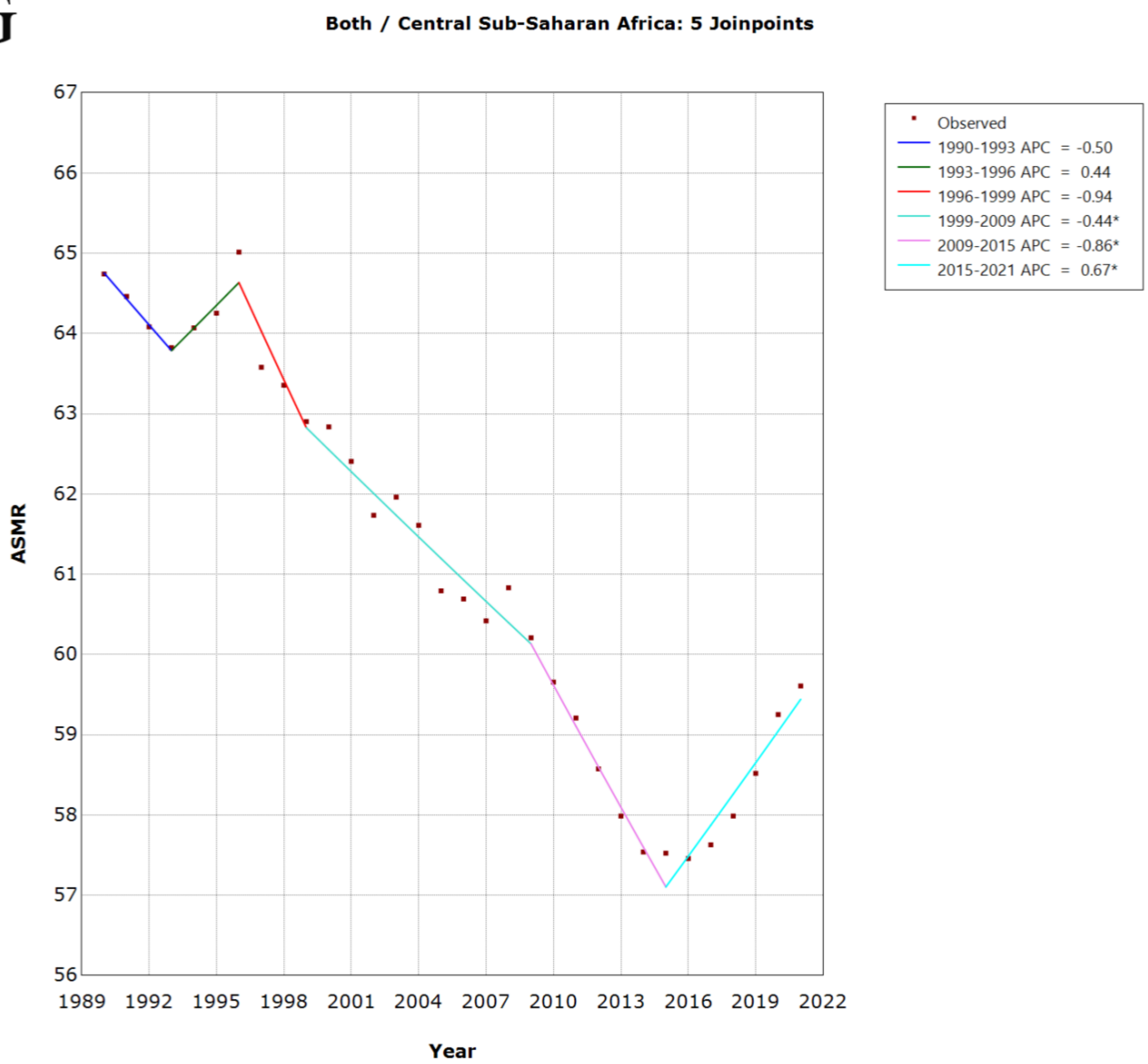

* Indicates that the Annual Percent Change (APC) is significantly different from zero at the alpha = 0.05 level
Final Selected Model: 5 Joinpoints.

H

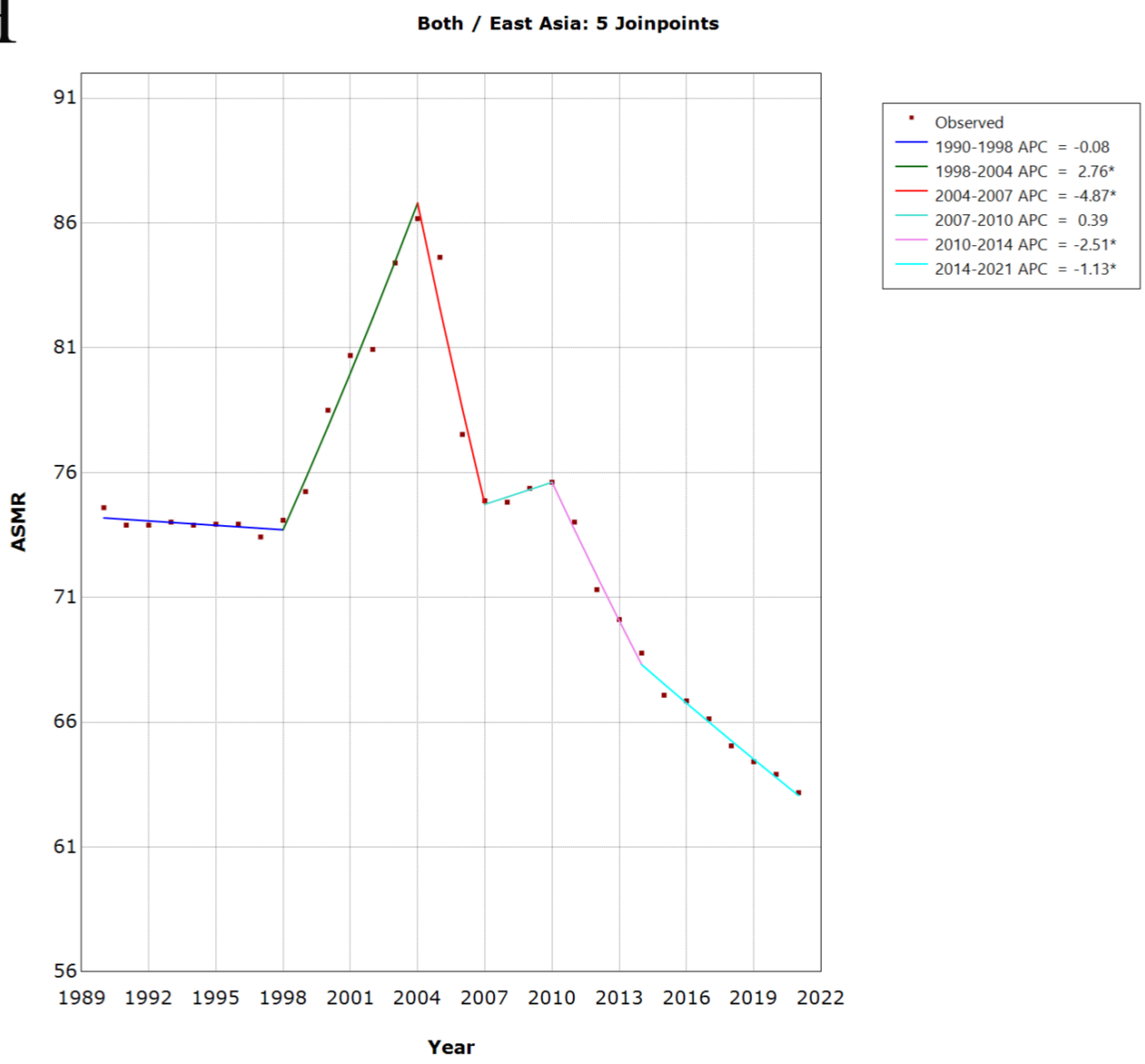

* Indicates that the Annual Percent Change (APC) is significantly different from zero at the alpha = 0.05 level
Final Selected Model: 5 Joinpoints.

I

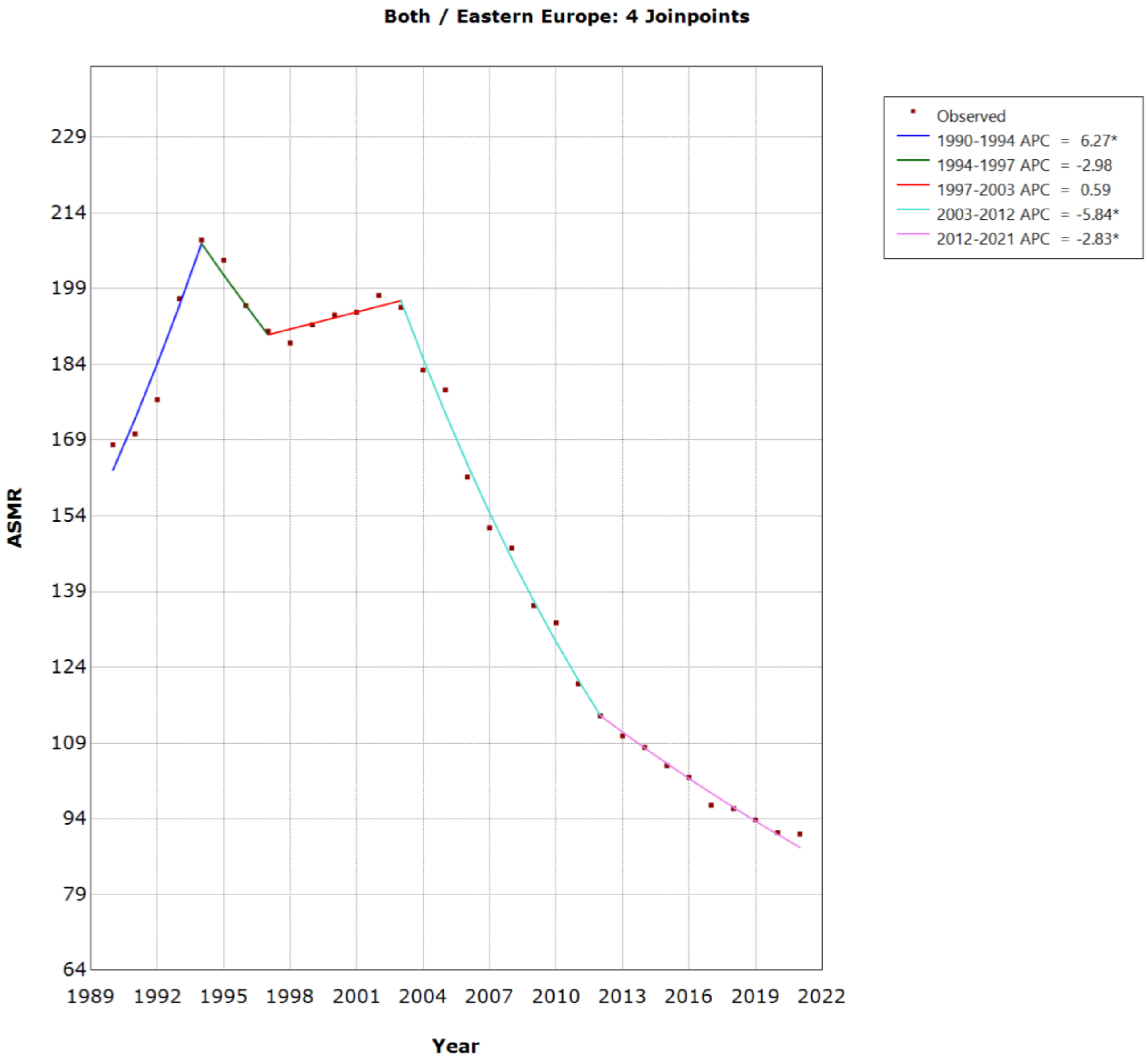

* Indicates that the Annual Percent Change (APC) is significantly different from zero at the alpha = 0.05 level
Final Selected Model: 4 Joinpoints.

J

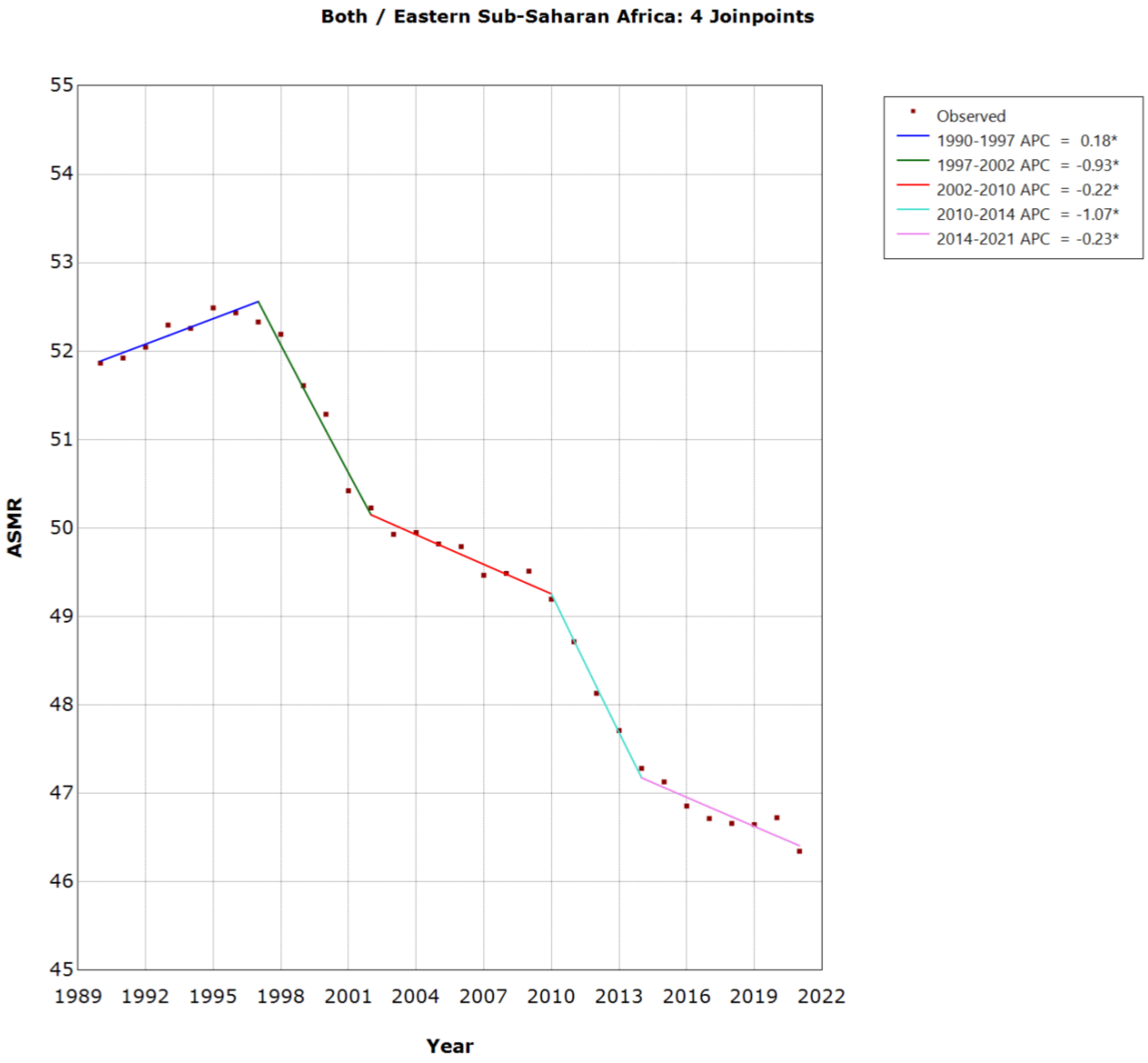

* Indicates that the Annual Percent Change (APC) is significantly different from zero at the alpha = 0.05 level
Final Selected Model: 4 Joinpoints.

K

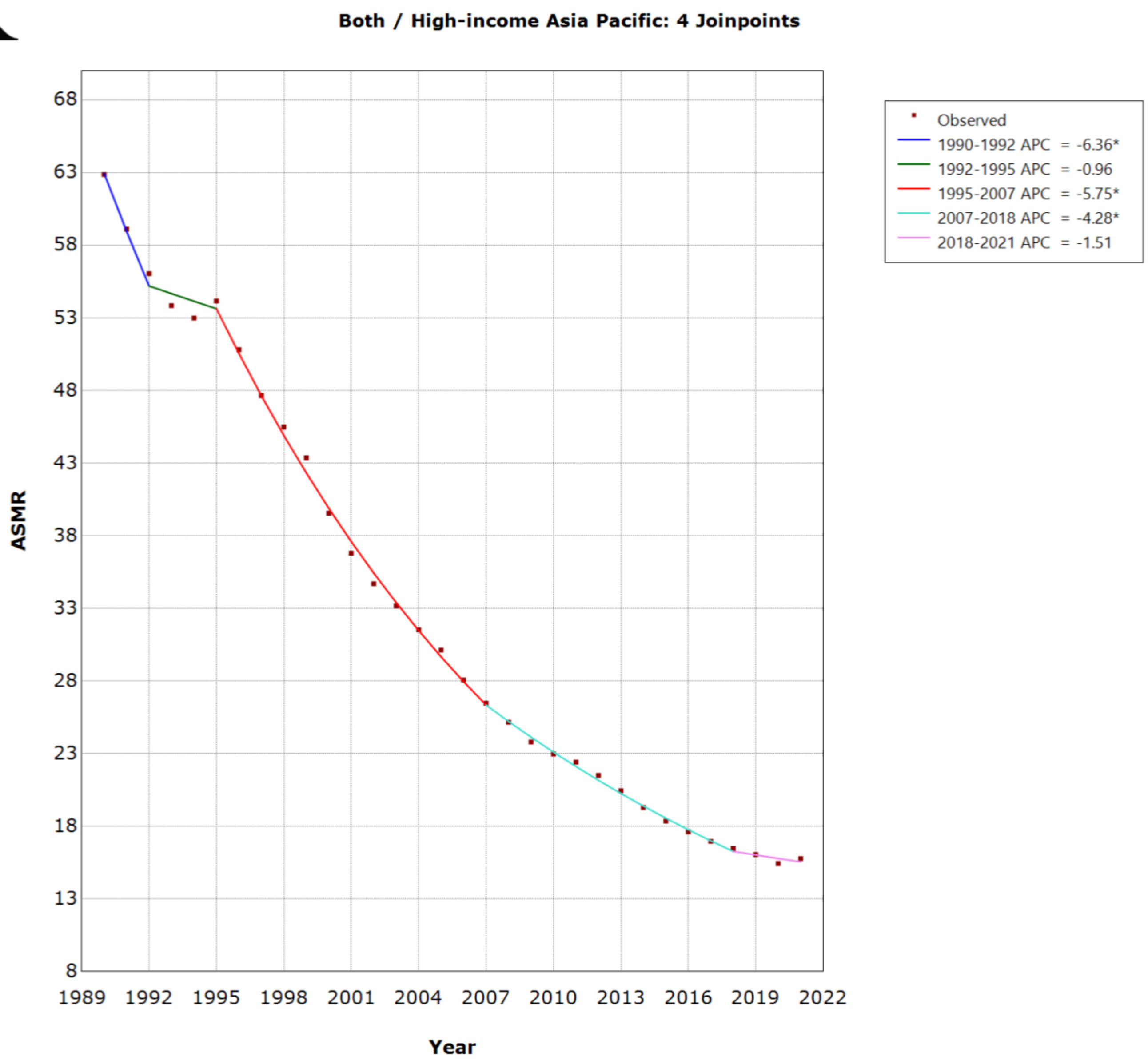

* Indicates that the Annual Percent Change (APC) is significantly different from zero at the alpha = 0.05 level
Final Selected Model: 4 Joinpoints.

L

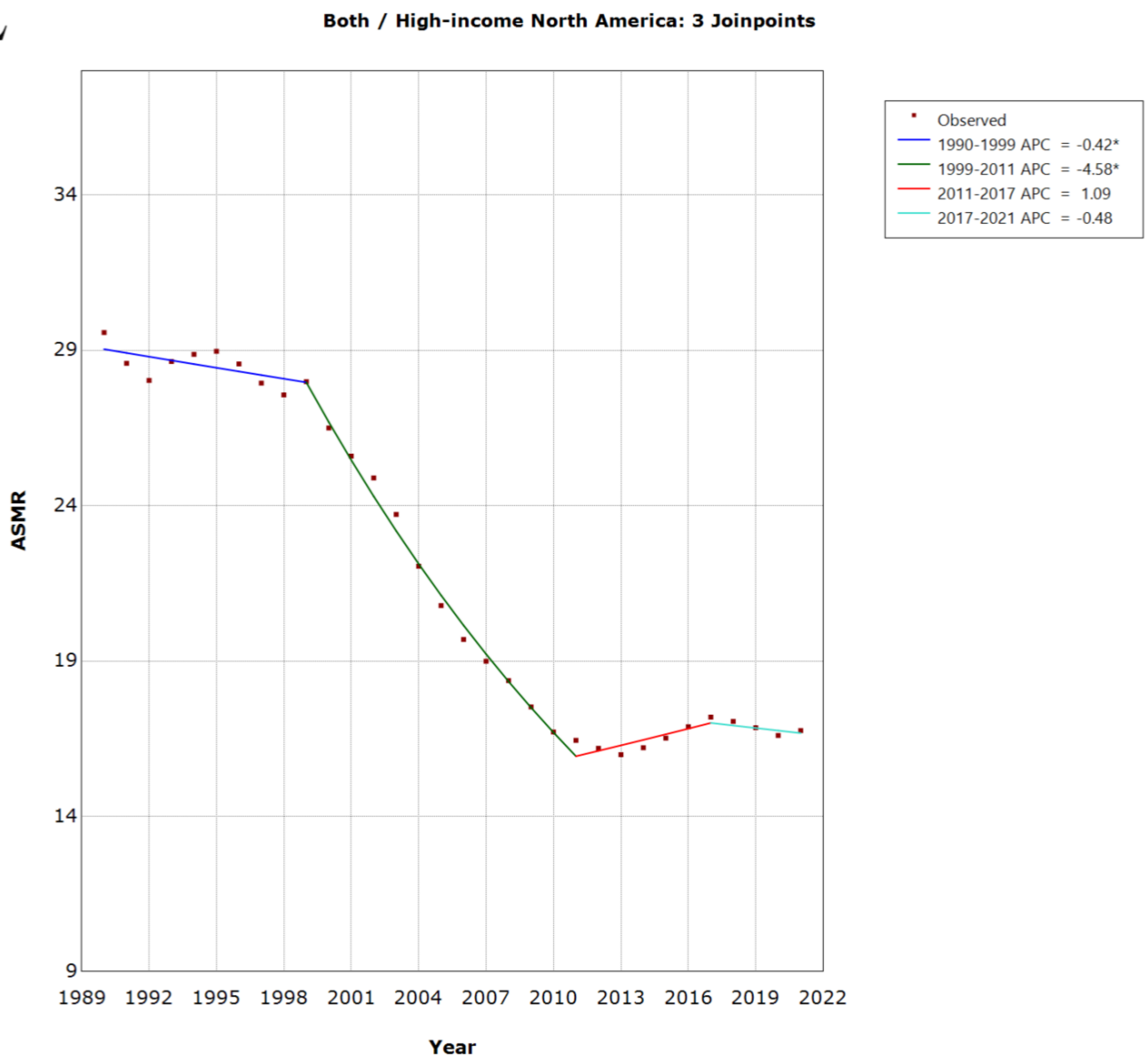

* Indicates that the Annual Percent Change (APC) is significantly different from zero at the alpha = 0.05 level
Final Selected Model: 3 Joinpoints.

M

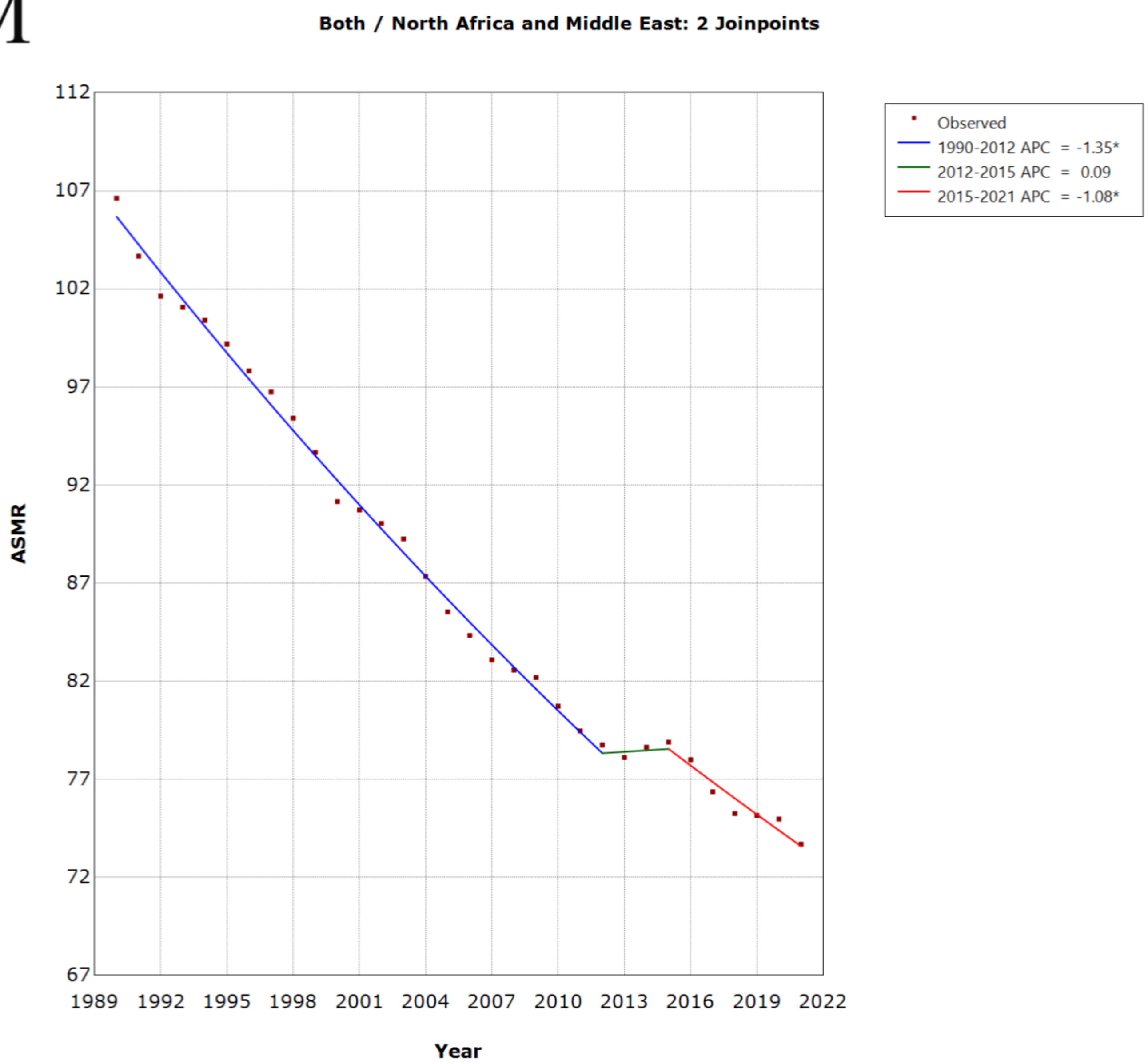

* Indicates that the Annual Percent Change (APC) is significantly different from zero at the alpha = 0.05 level
Final Selected Model: 2 Joinpoints.

N

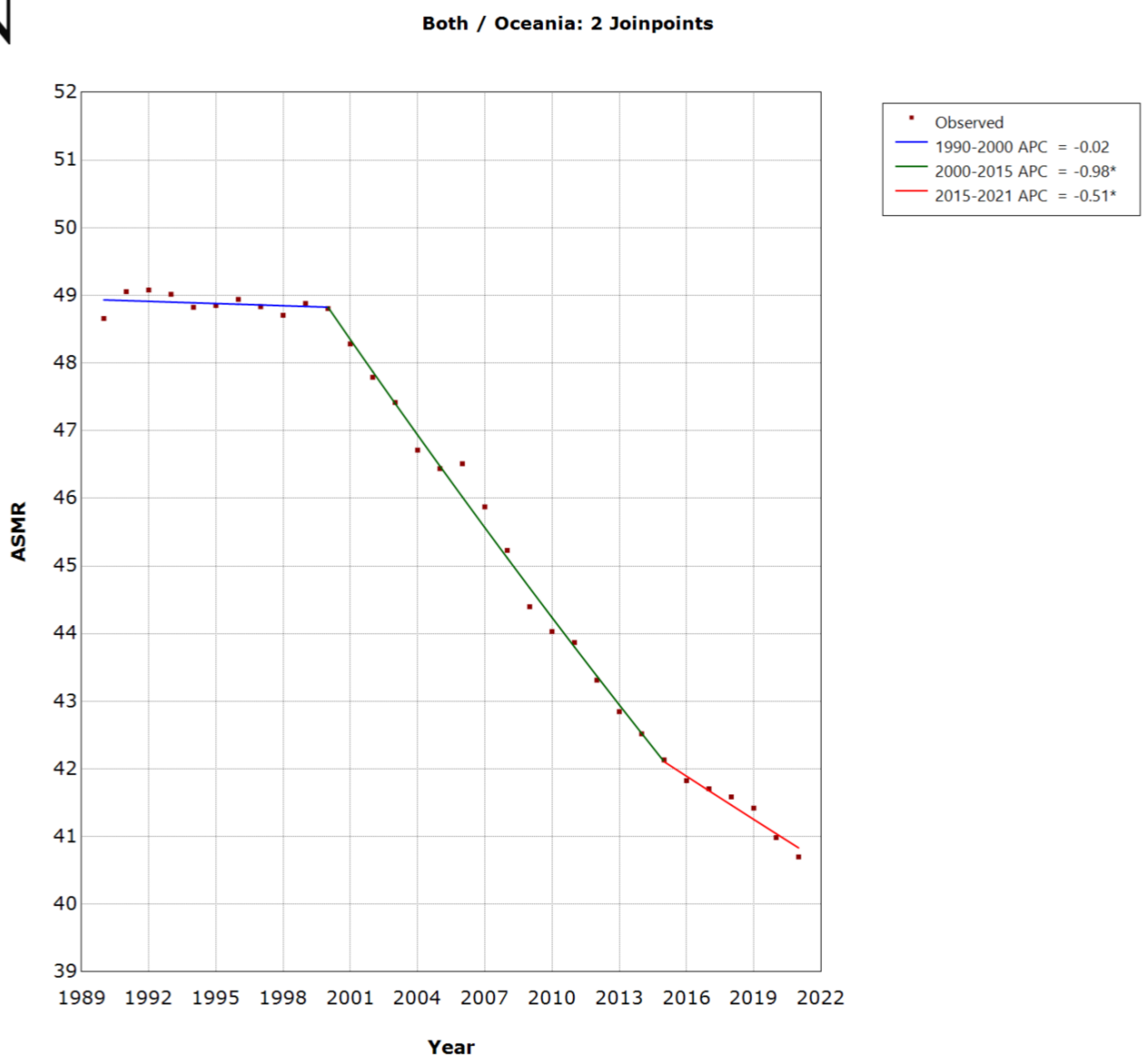

* Indicates that the Annual Percent Change (APC) is significantly different from zero at the alpha = 0.05 level
Final Selected Model: 2 Joinpoints.

O

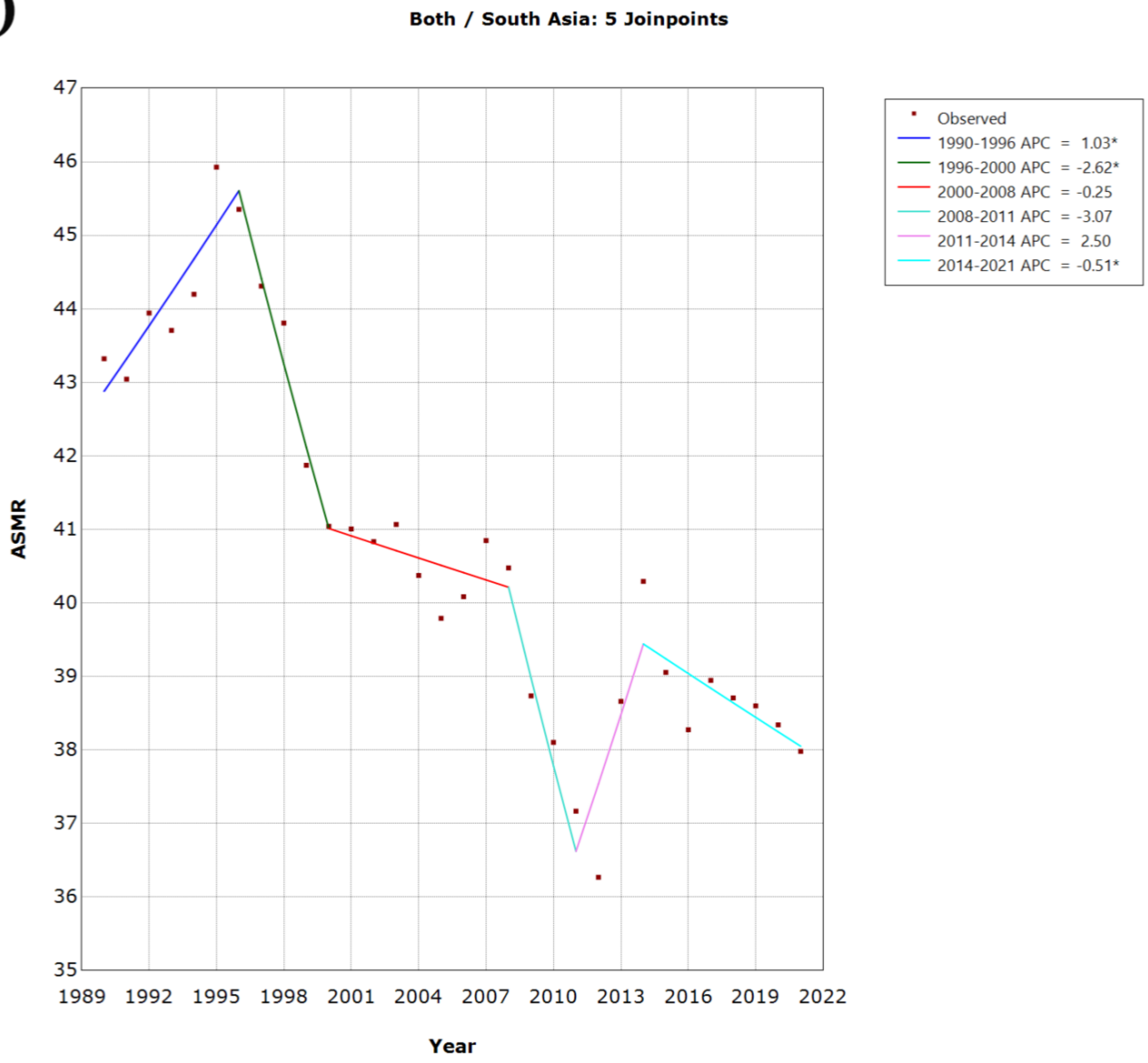

* Indicates that the Annual Percent Change (APC) is significantly different from zero at the alpha = 0.05 level
Final Selected Model: 5 Joinpoints.

P

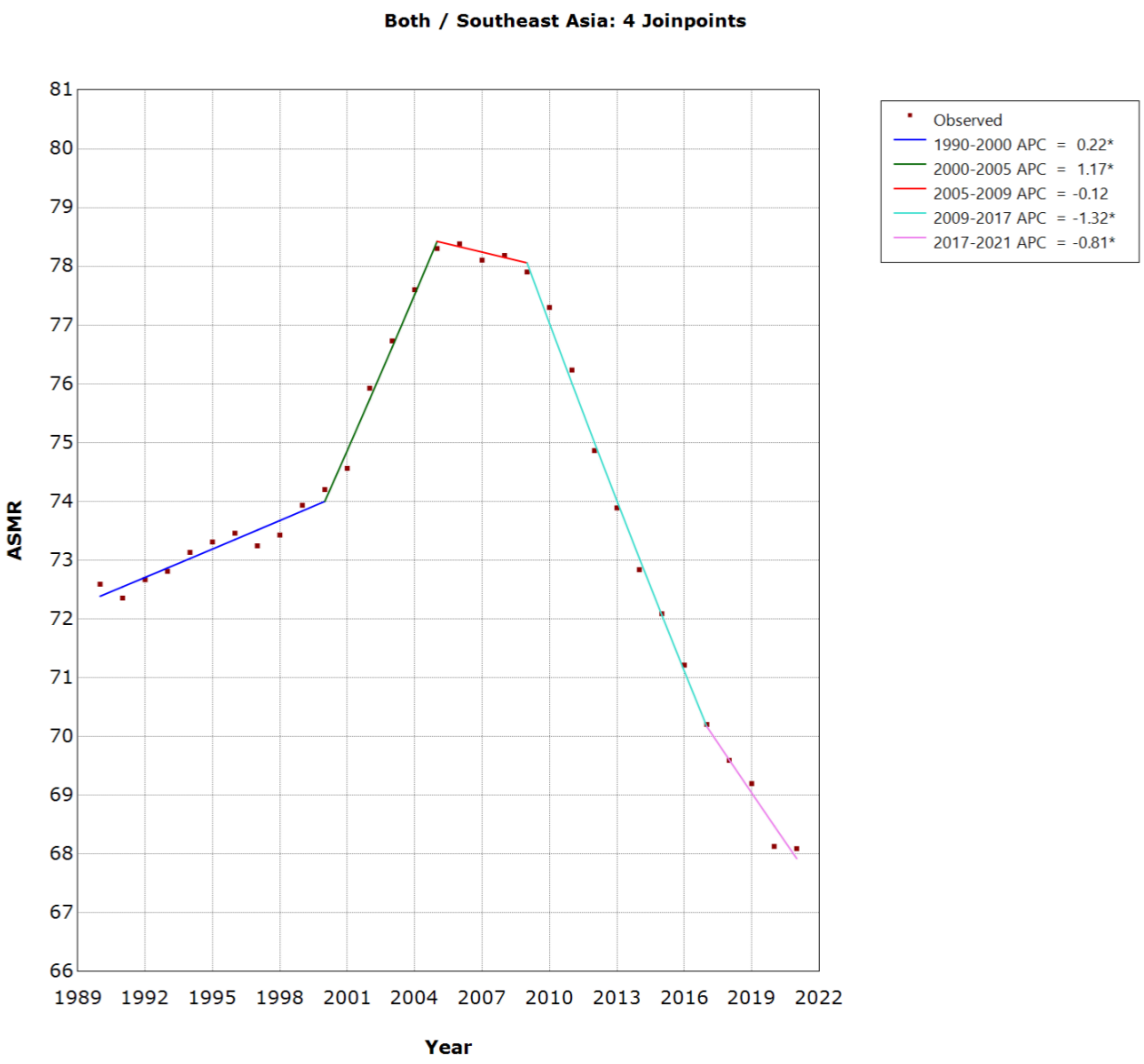

* Indicates that the Annual Percent Change (APC) is significantly different from zero at the alpha = 0.05 level
Final Selected Model: 4 Joinpoints.

Q

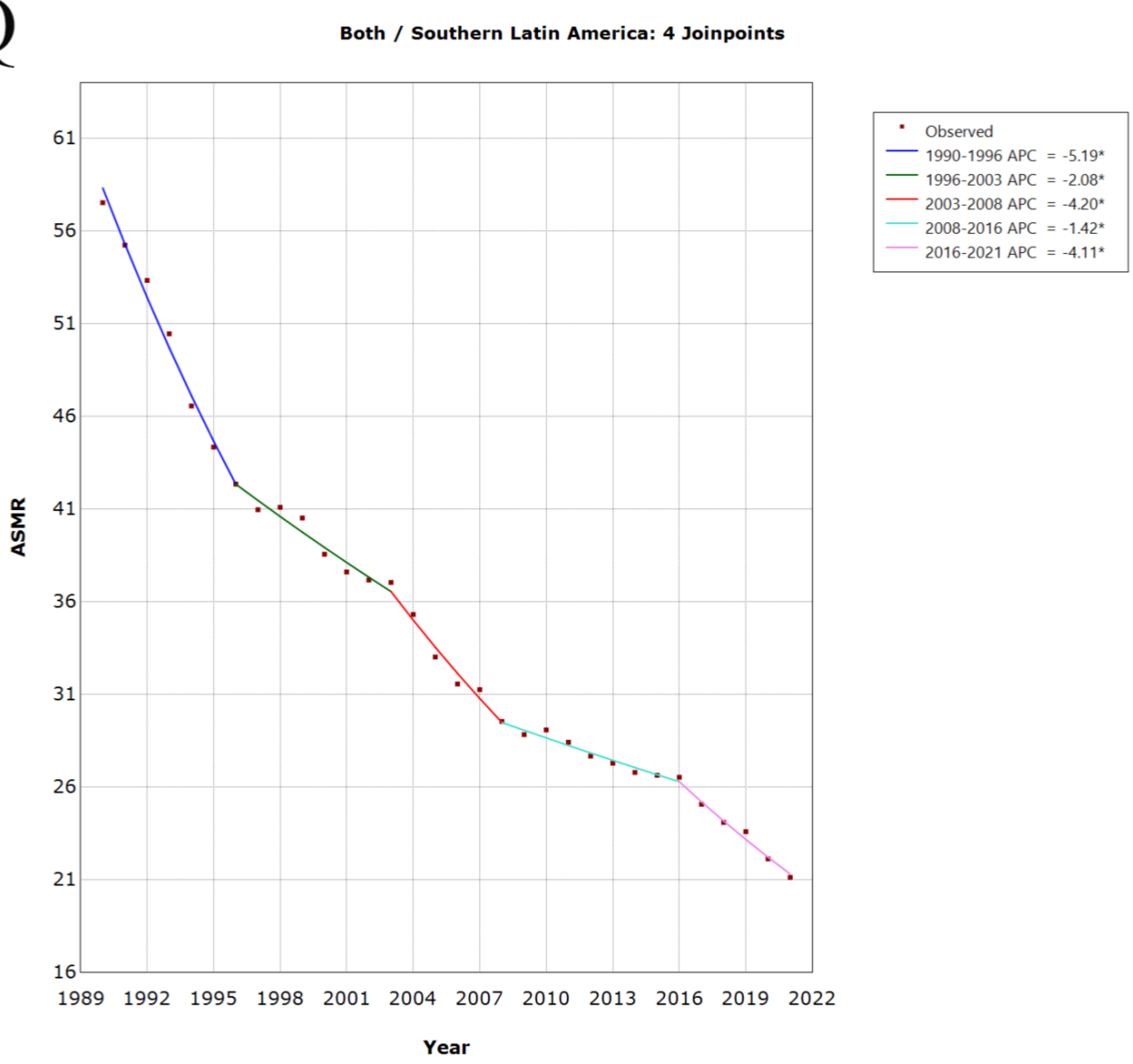

* Indicates that the Annual Percent Change (APC) is significantly different from zero at the alpha = 0.05 level
Final Selected Model: 4 Joinpoints.

R

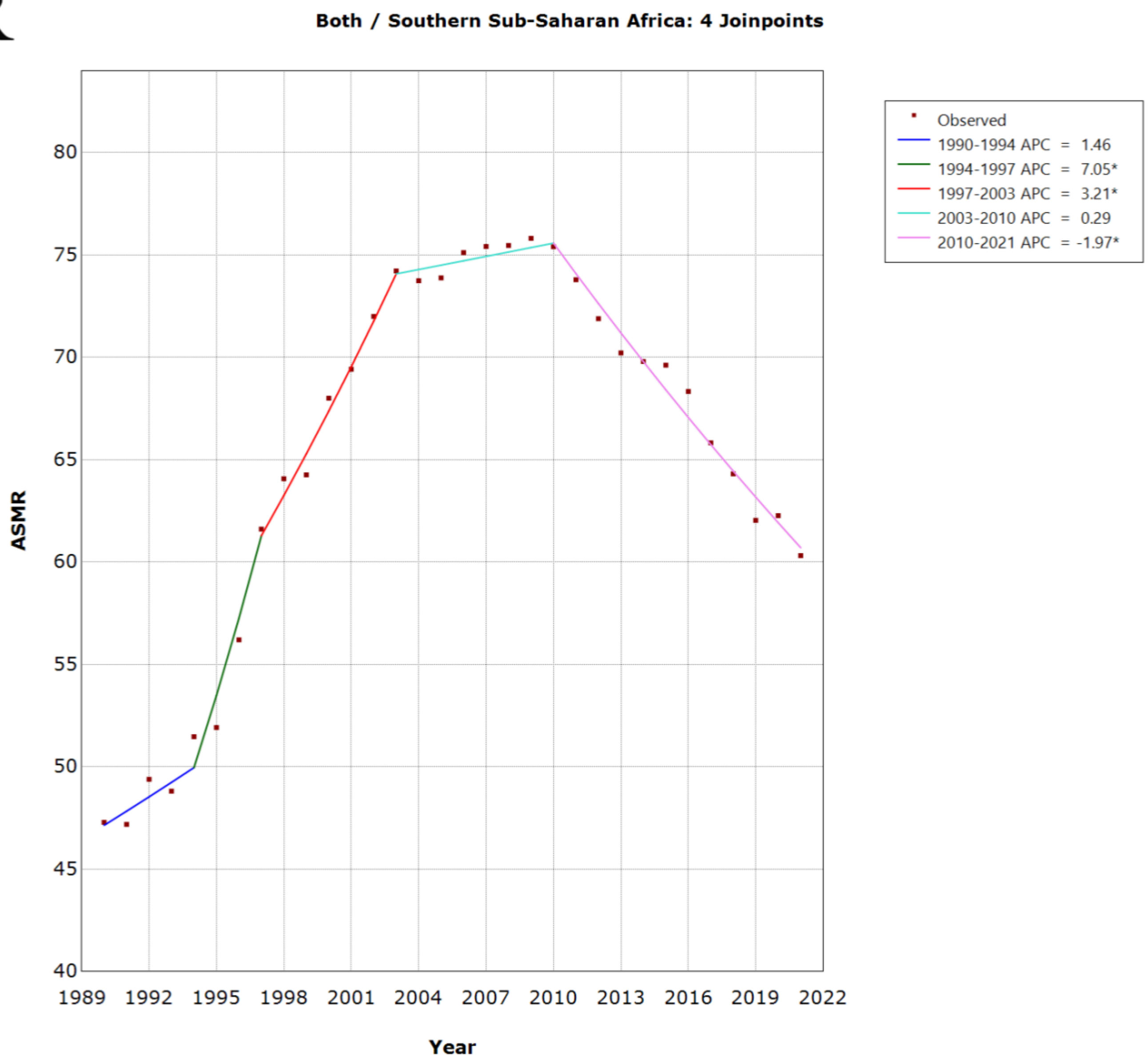

* Indicates that the Annual Percent Change (APC) is significantly different from zero at the alpha = 0.05 level
Final Selected Model: 4 Joinpoints.

S

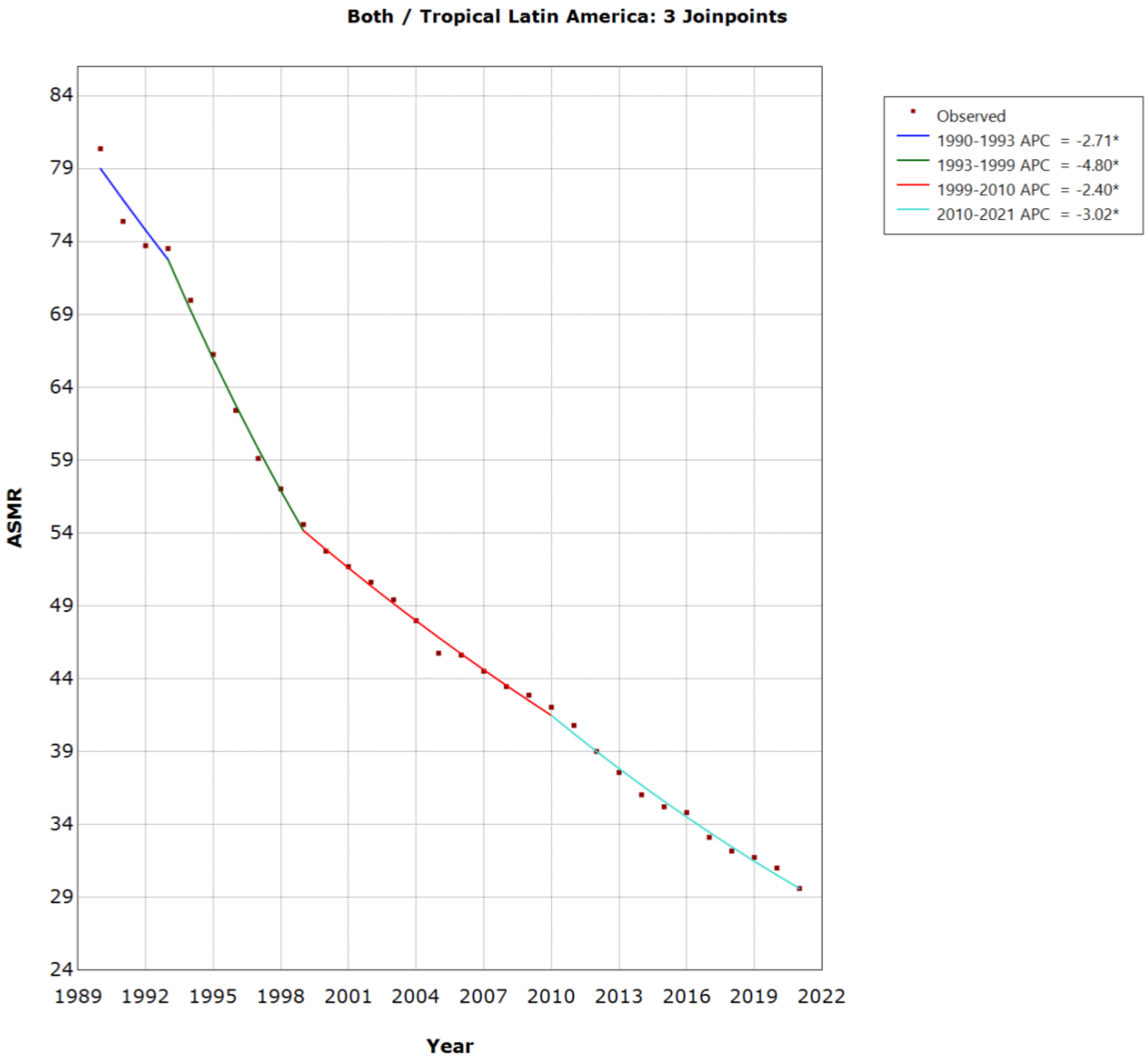

* Indicates that the Annual Percent Change (APC) is significantly different from zero at the alpha = 0.05 level
Final Selected Model: 3 Joinpoints.

T

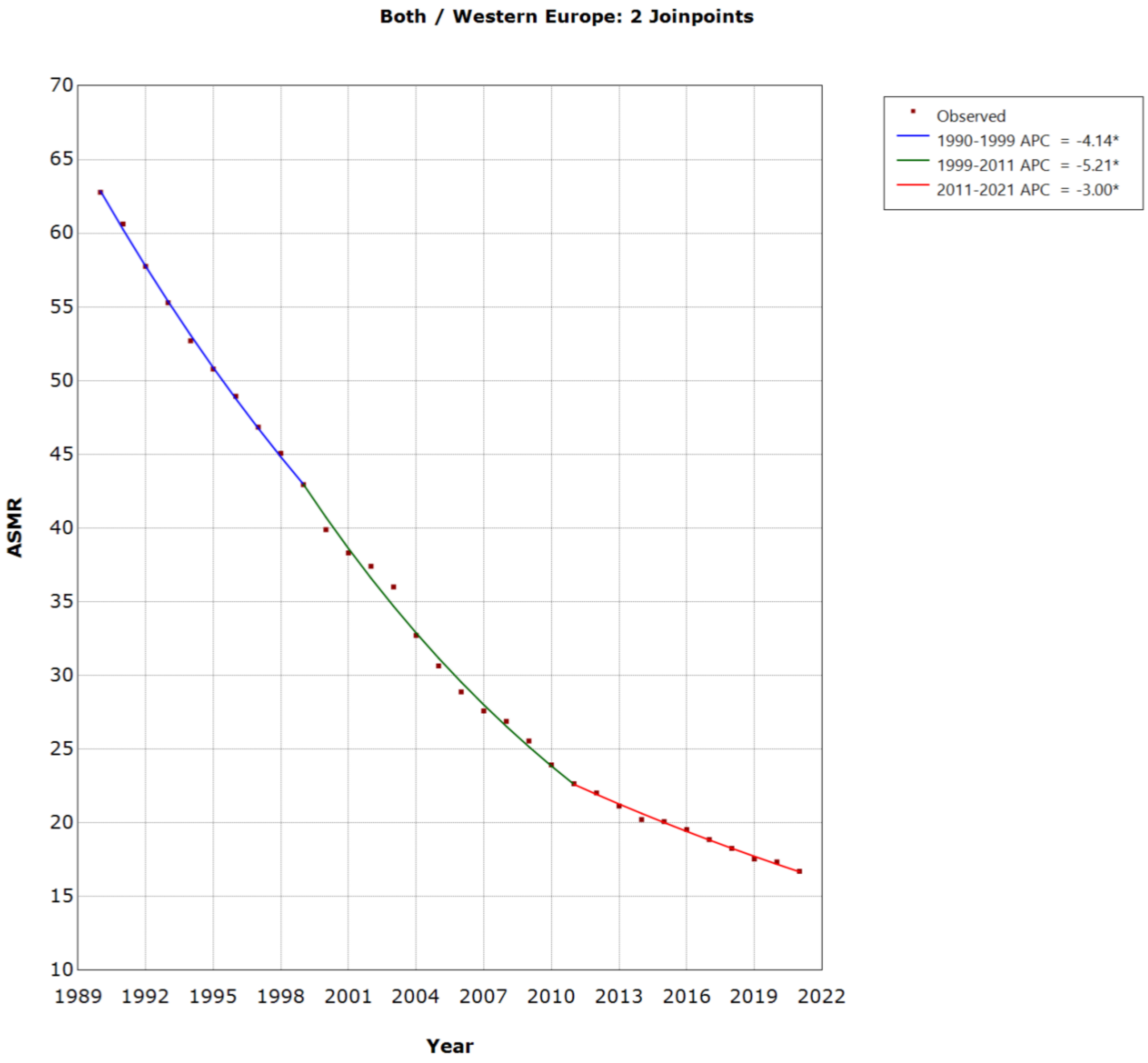

* Indicates that the Annual Percent Change (APC) is significantly different from zero at the alpha = 0.05 level
Final Selected Model: 2 Joinpoints.

U

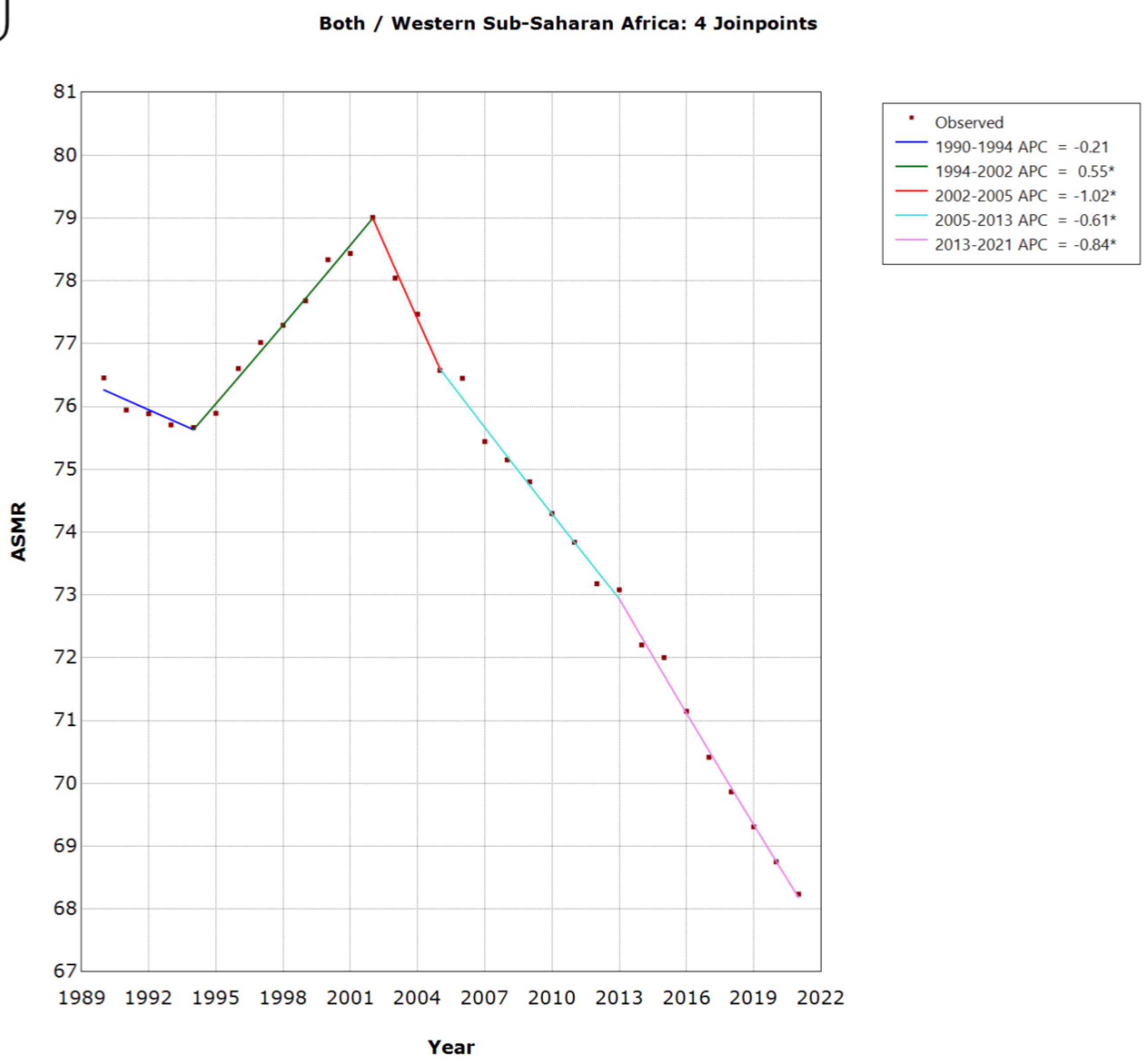

* Indicates that the Annual Percent Change (APC) is significantly different from zero at the alpha = 0.05 level
Final Selected Model: 4 Joinpoints.

Supplementary Figure 2. APC of ASMR of ischemic stroke in each GBD region from 1990 to 2021. A. APC of ASMR of ischemic stroke in Andean Latin America from 1990 to 2021. B. APC of ASMR of ischemic stroke in Australasia from 1990 to 2021. C. APC of ASMR of ischemic stroke in Caribbean from 1990 to 2021. D. APC of ASMR of ischemic stroke in Central Asia from 1990 to 2021. E. APC of ASMR of ischemic stroke in Central Europe from 1990 to 2021. F. APC of ASMR of ischemic stroke in Central Latin America from 1990 to 2021. G. APC of ASMR of ischemic stroke in Central Sub-Saharan Africa from 1990 to 2021. H. APC of ASMR of ischemic stroke in East Asia from 1990 to 2021. I. APC of ASMR of ischemic stroke in Eastern Europe from 1990 to 2021. J. APC of ASMR of ischemic stroke in Eastern Sub-Saharan Africa from 1990 to 2021. K. APC of ASMR of ischemic stroke in High-income Asia Pacific from 1990 to 2021. L. APC of ASMR of ischemic stroke in High-income North America from 1990 to 2021. M. APC of ASMR of ischemic stroke in North Africa and Middle East from 1990 to 2021. N. APC of ASMR of ischemic stroke in Oceania from 1990 to 2021. O. APC of ASMR of ischemic stroke in South Asia from 1990 to 2021. P. APC of ASMR of ischemic stroke in Southeast Asia from 1990 to 2021. Q. APC of ASMR of ischemic stroke in Southern Latin America from 1990 to 2021. R. APC of ASMR of ischemic stroke in Southern Sub-Saharan Africa from 1990 to 2021. S. APC of ASMR of ischemic stroke in Tropical Latin America from 1990 to 2021. T. APC of ASMR of ischemic stroke in each Western Europe 1990 to 2021. U. APC of ASMR of ischemic stroke in each Western Sub-Saharan Africa 1990 to 2021. APC = Annual percent change. ASMR = Age-standardized mortality rate.

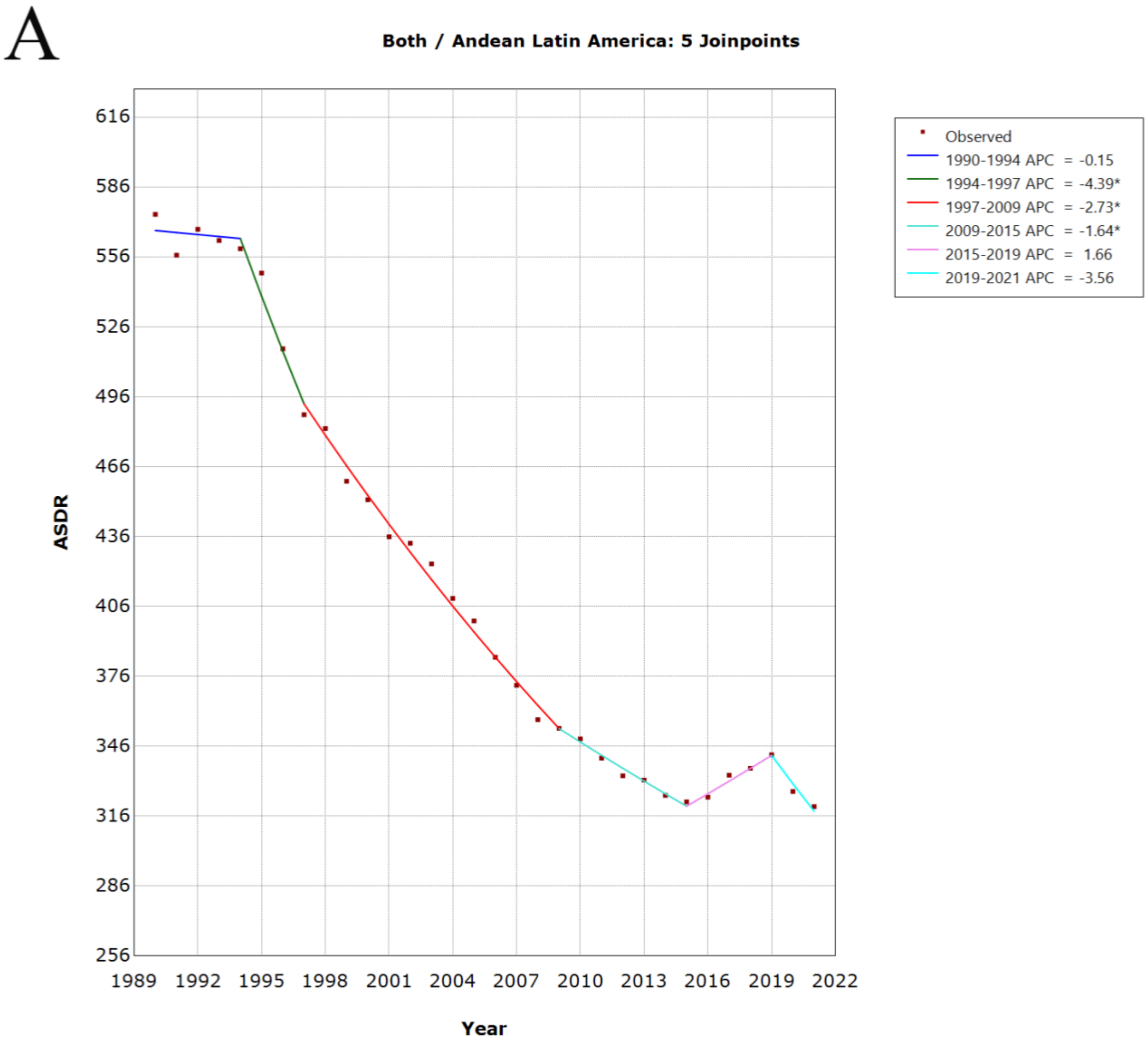

* Indicates that the Annual Percent Change (APC) is significantly different from zero at the alpha = 0.05 level
Final Selected Model: 5 Joinpoints.

B

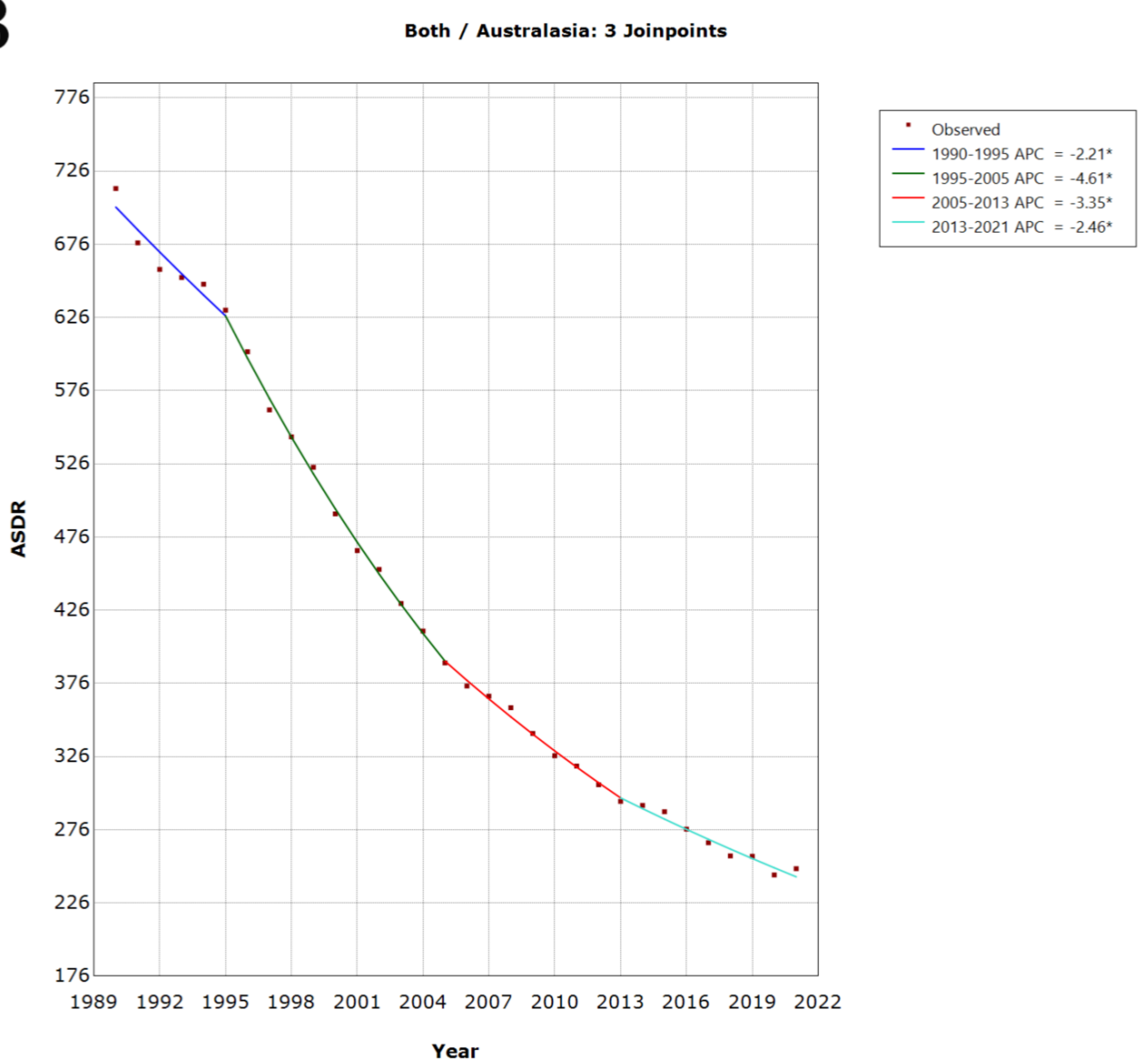

* Indicates that the Annual Percent Change (APC) is significantly different from zero at the alpha = 0.05 level
Final Selected Model: 3 Joinpoints.

C

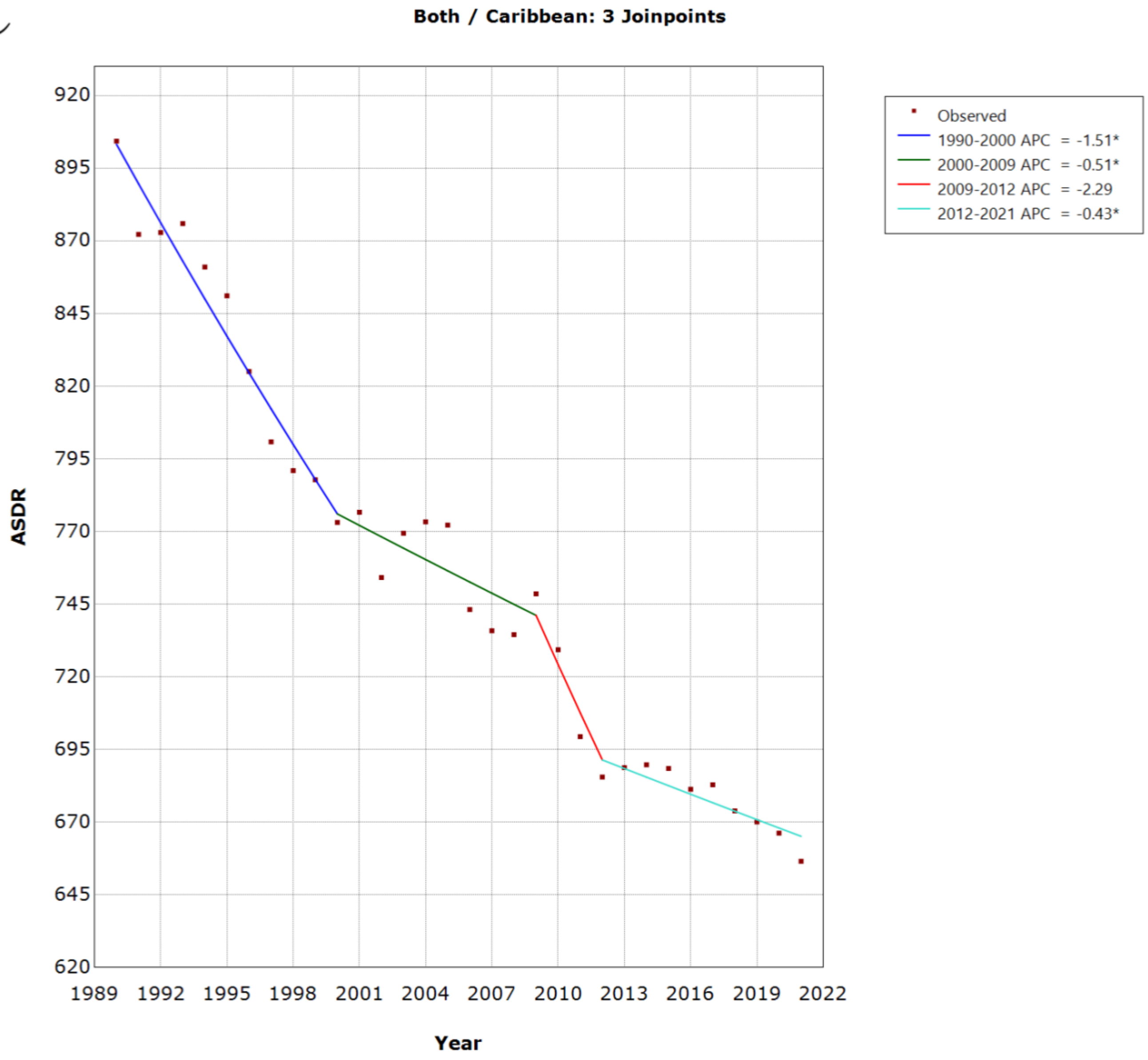

* Indicates that the Annual Percent Change (APC) is significantly different from zero at the alpha = 0.05 level
Final Selected Model: 3 Joinpoints.

D

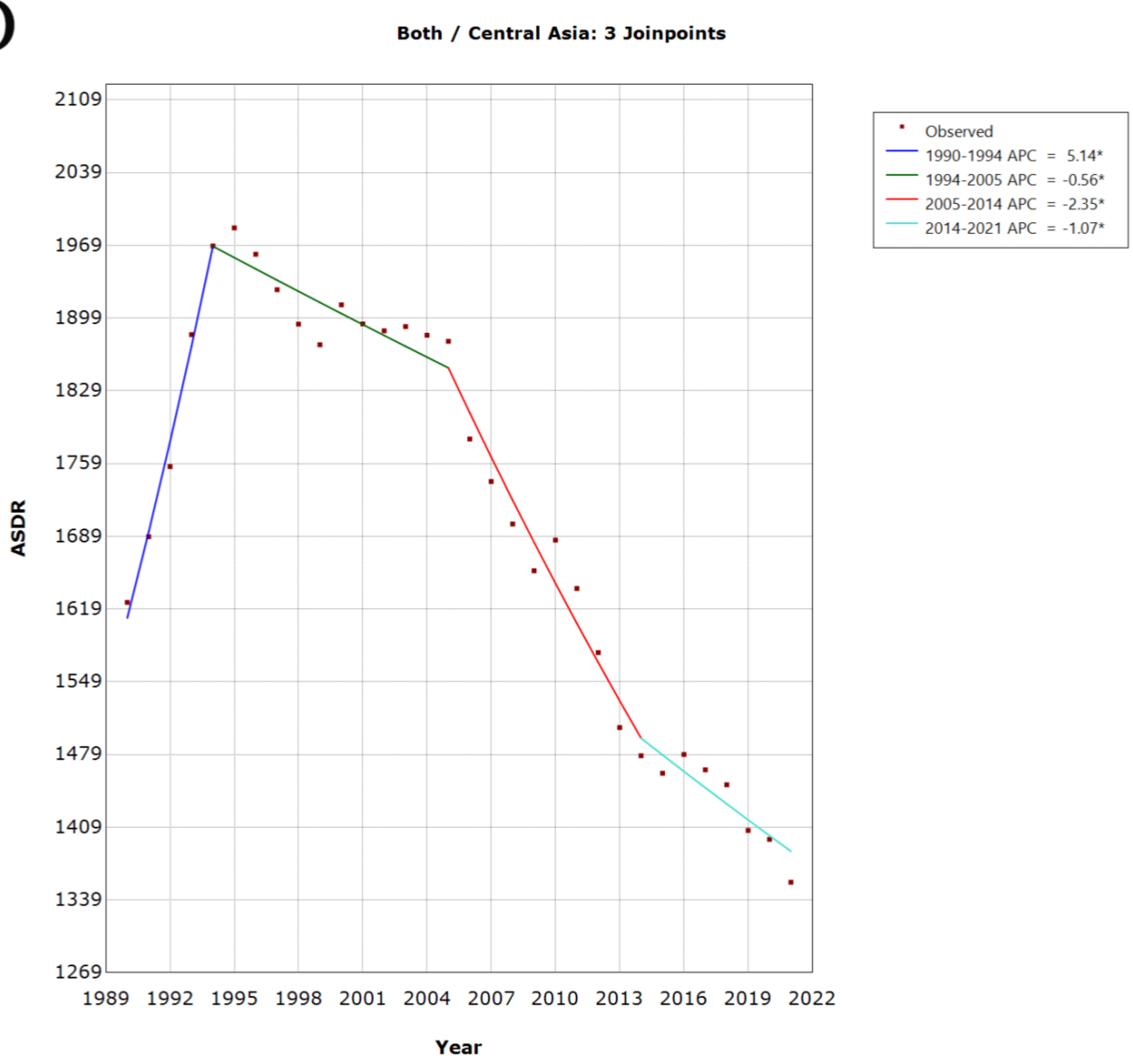

* Indicates that the Annual Percent Change (APC) is significantly different from zero at the alpha = 0.05 level
Final Selected Model: 3 Joinpoints.

E

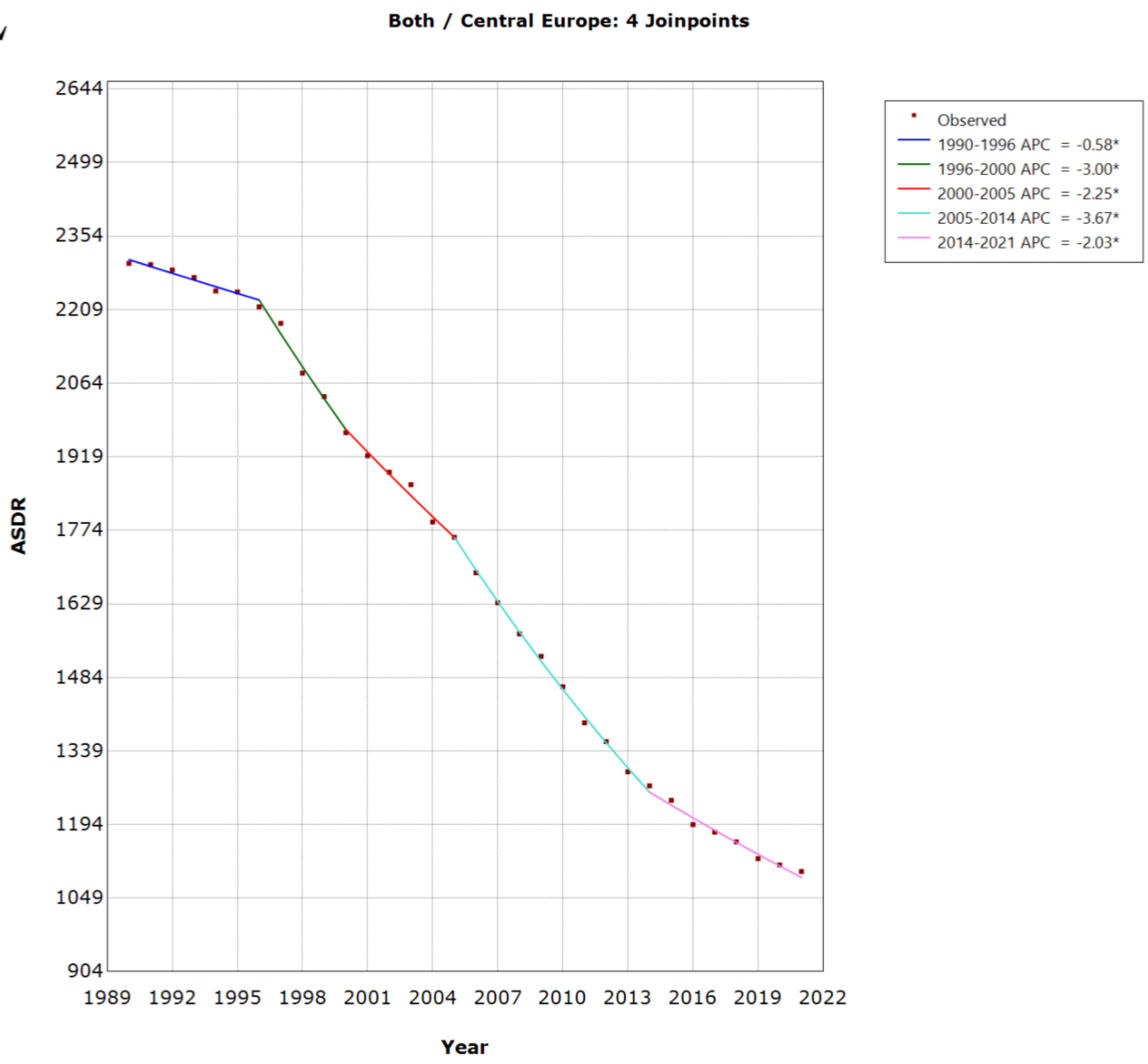

* Indicates that the Annual Percent Change (APC) is significantly different from zero at the alpha = 0.05 level
Final Selected Model: 4 Joinpoints.

F

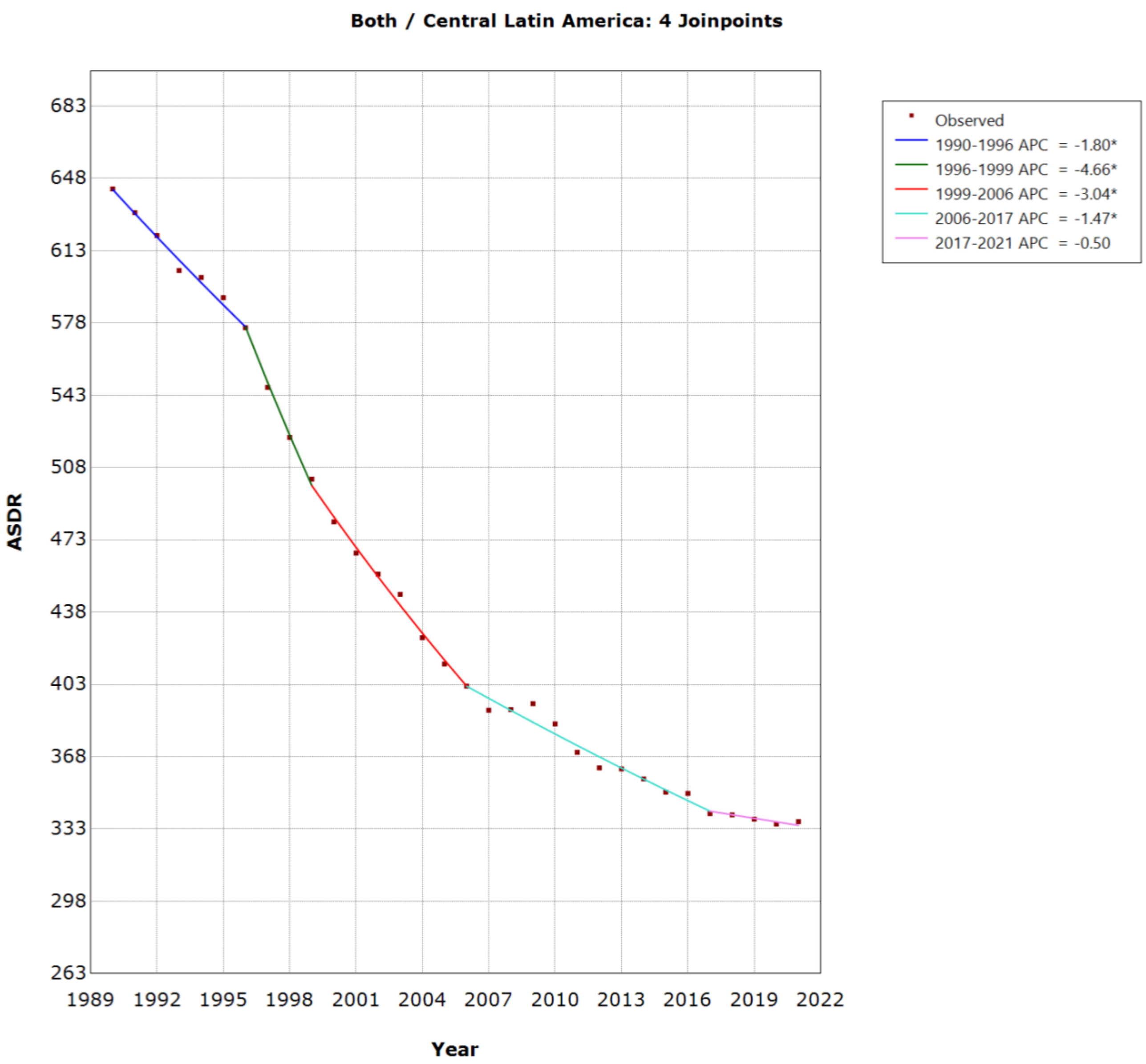

* Indicates that the Annual Percent Change (APC) is significantly different from zero at the alpha = 0.05 level
Final Selected Model: 4 Joinpoints.

G

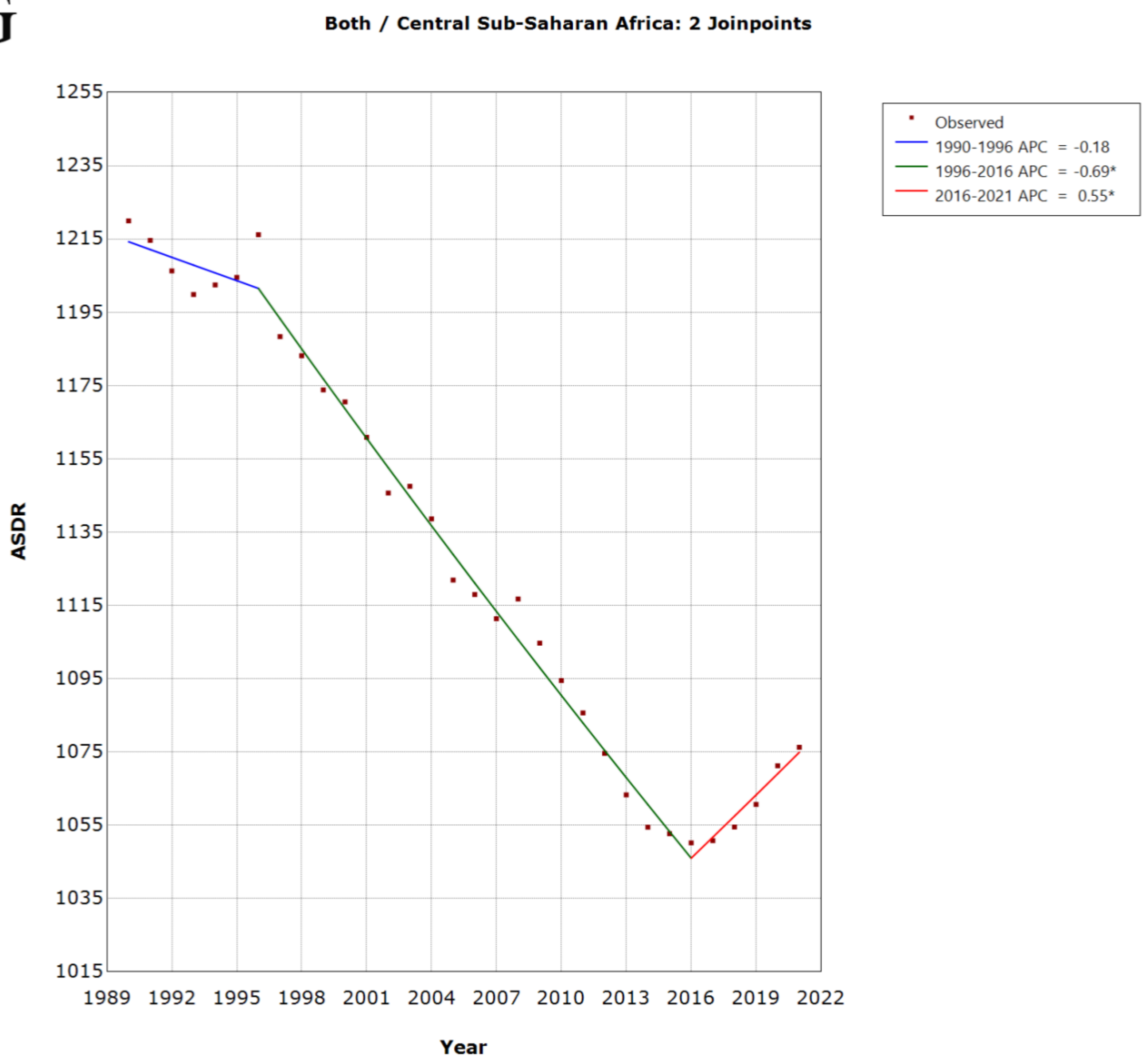

* Indicates that the Annual Percent Change (APC) is significantly different from zero at the alpha = 0.05 level
Final Selected Model: 2 Joinpoints.

H

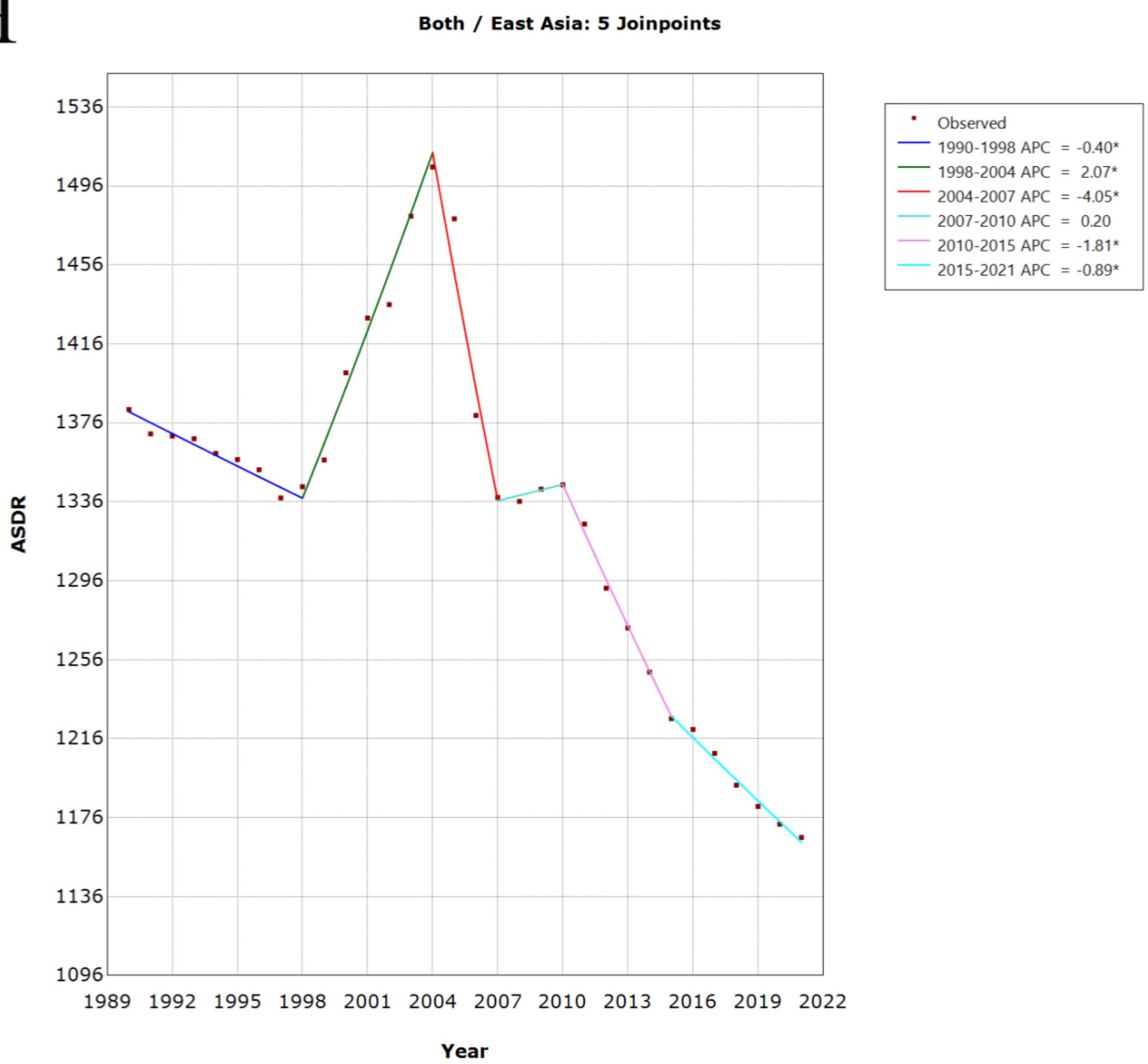

* Indicates that the Annual Percent Change (APC) is significantly different from zero at the alpha = 0.05 level
Final Selected Model: 5 Joinpoints.

I

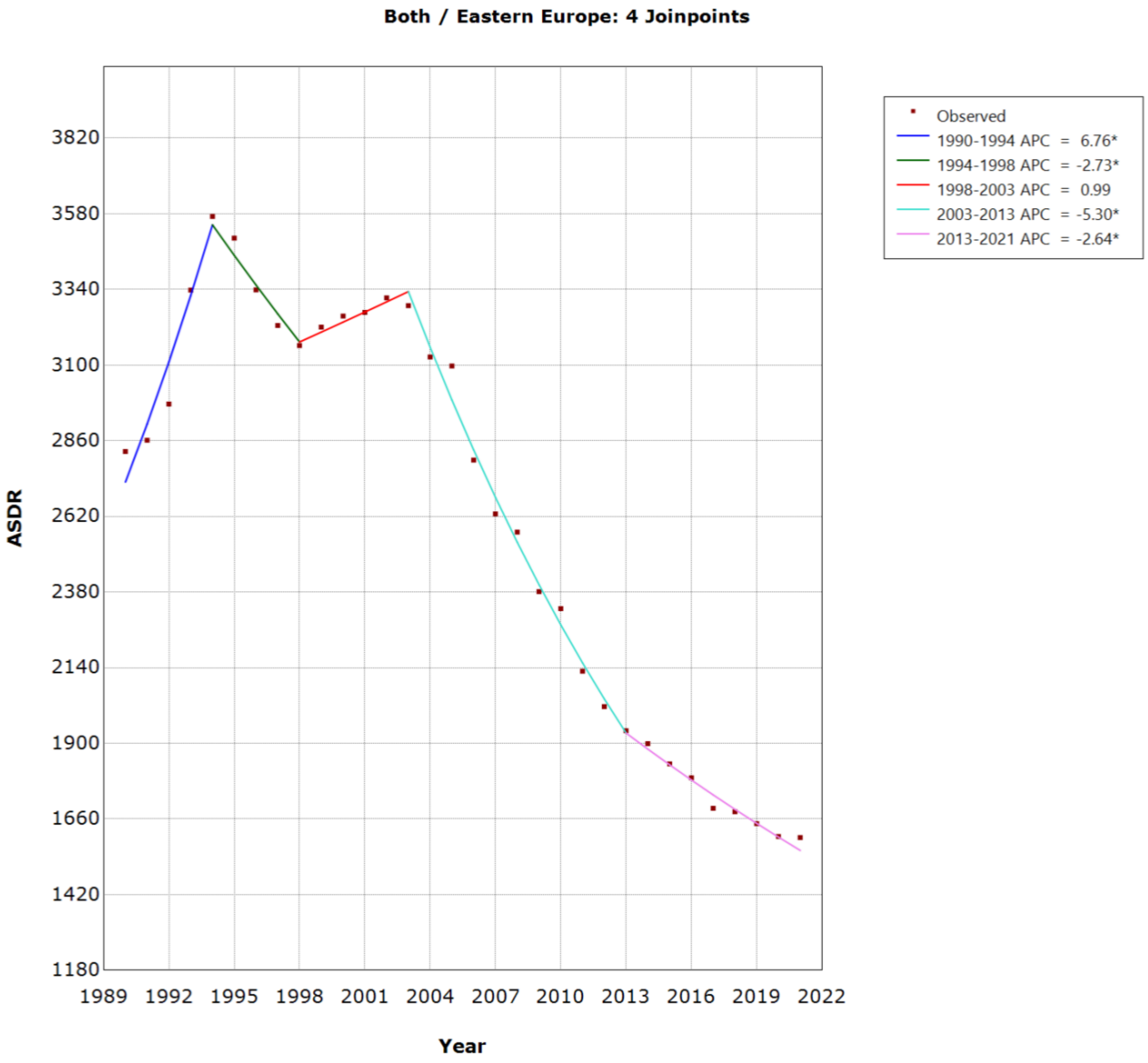

* Indicates that the Annual Percent Change (APC) is significantly different from zero at the alpha = 0.05 level
Final Selected Model: 4 Joinpoints.

J

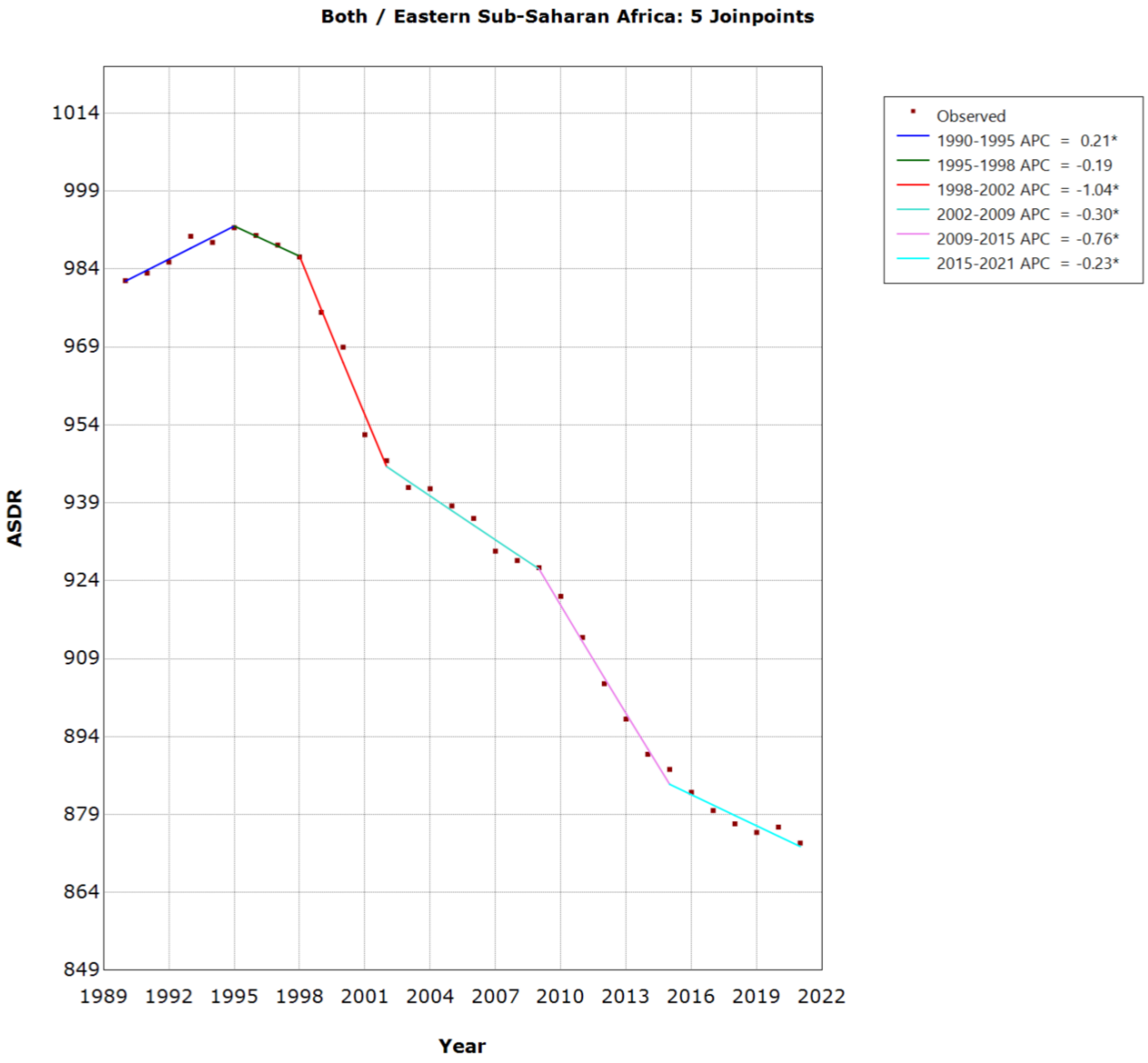

* Indicates that the Annual Percent Change (APC) is significantly different from zero at the alpha = 0.05 level
Final Selected Model: 5 Joinpoints.

K

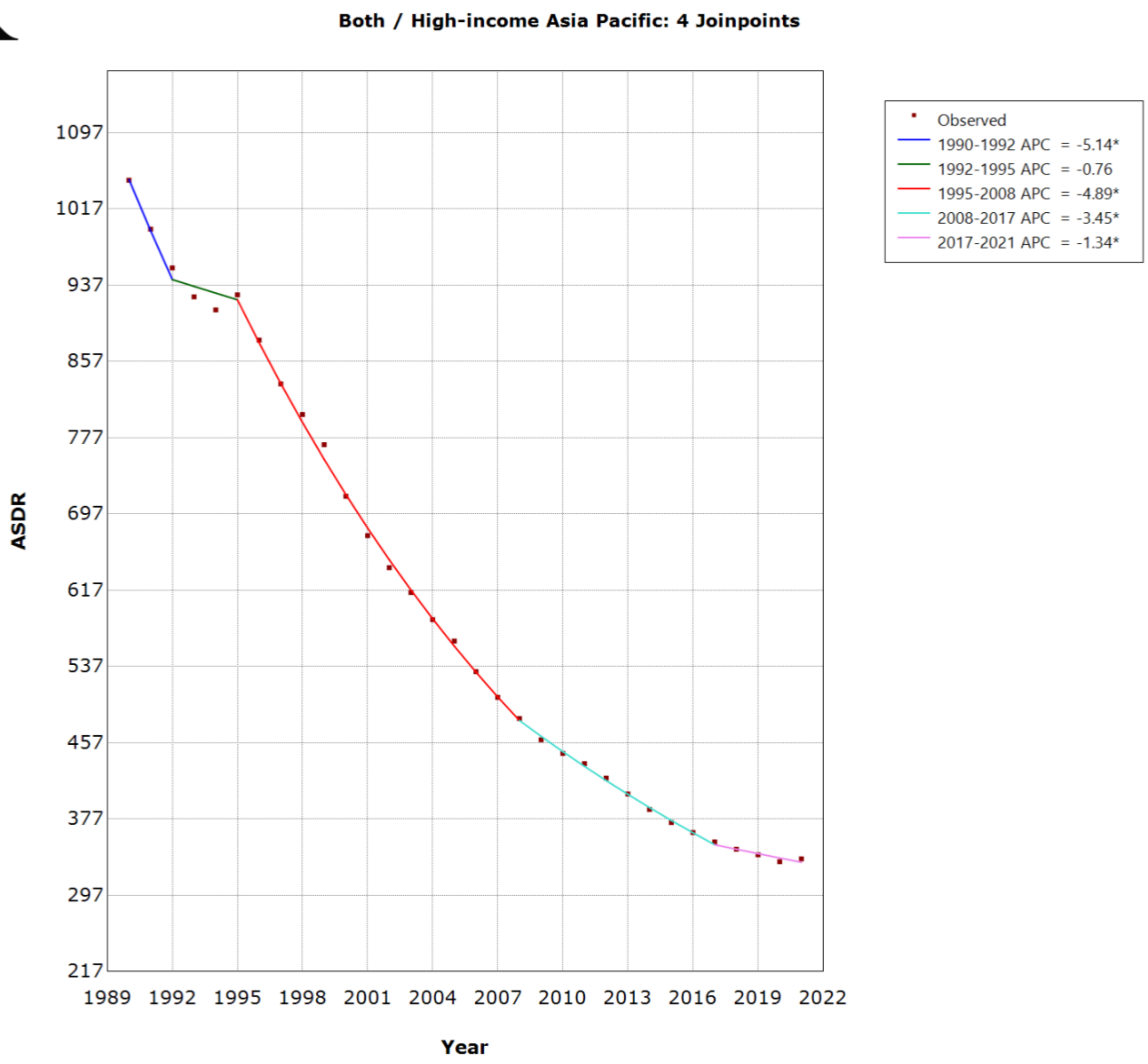

* Indicates that the Annual Percent Change (APC) is significantly different from zero at the alpha = 0.05 level
Final Selected Model: 4 Joinpoints.

L

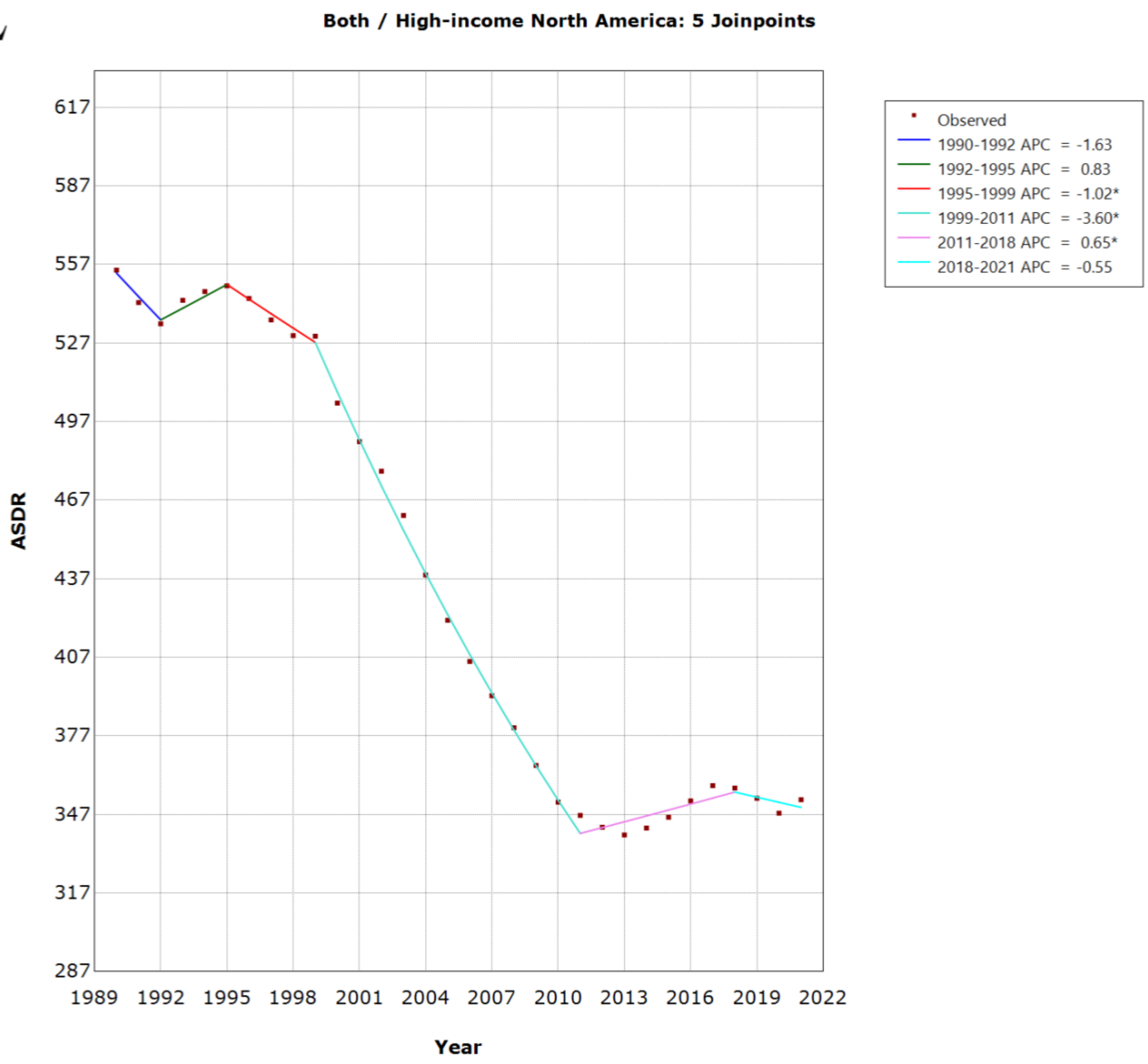

* Indicates that the Annual Percent Change (APC) is significantly different from zero at the alpha = 0.05 level
Final Selected Model: 5 Joinpoints.

M

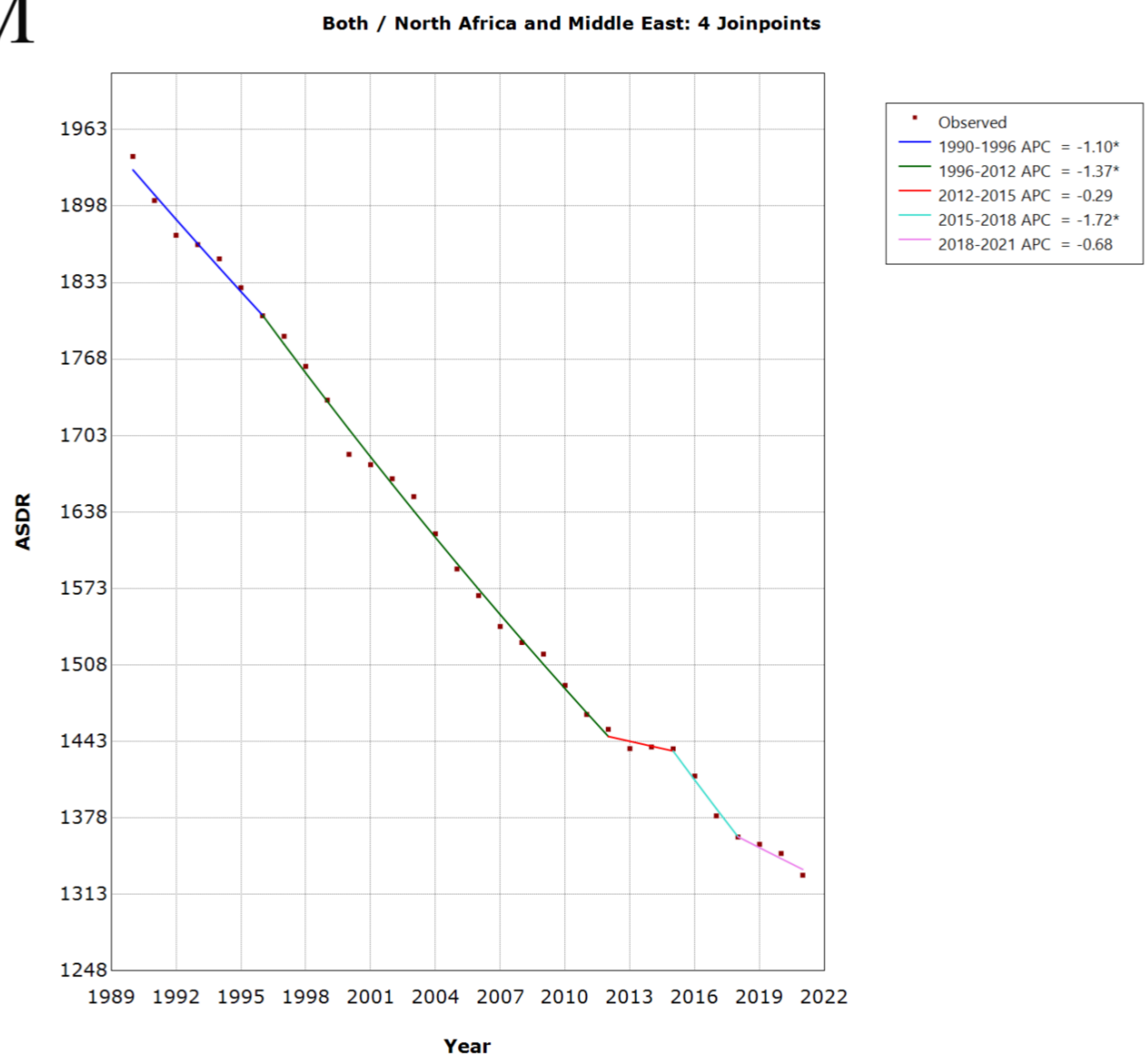

* Indicates that the Annual Percent Change (APC) is significantly different from zero at the alpha = 0.05 level
Final Selected Model: 4 Joinpoints.

N

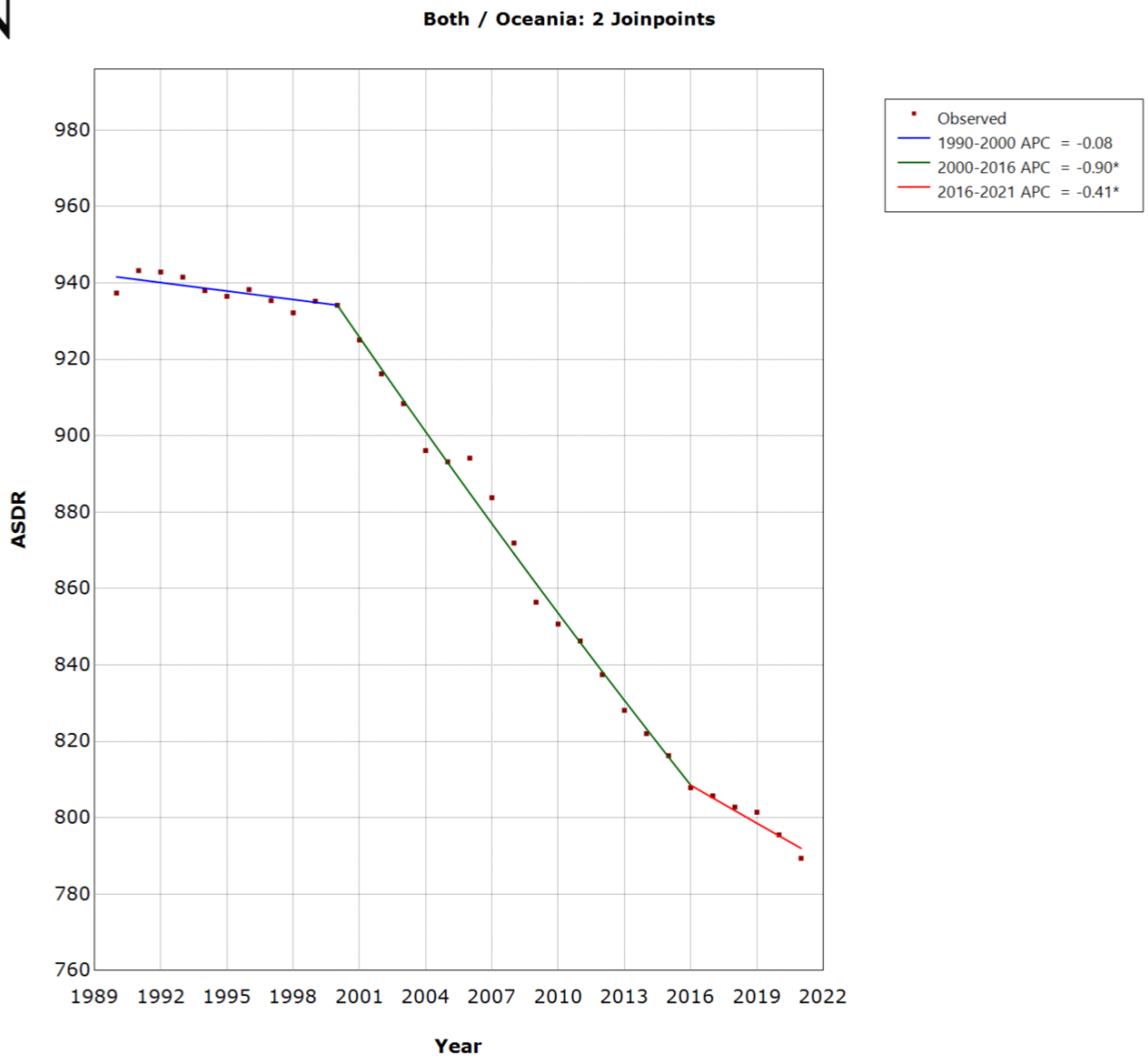

* Indicates that the Annual Percent Change (APC) is significantly different from zero at the alpha = 0.05 level
Final Selected Model: 2 Joinpoints.

O

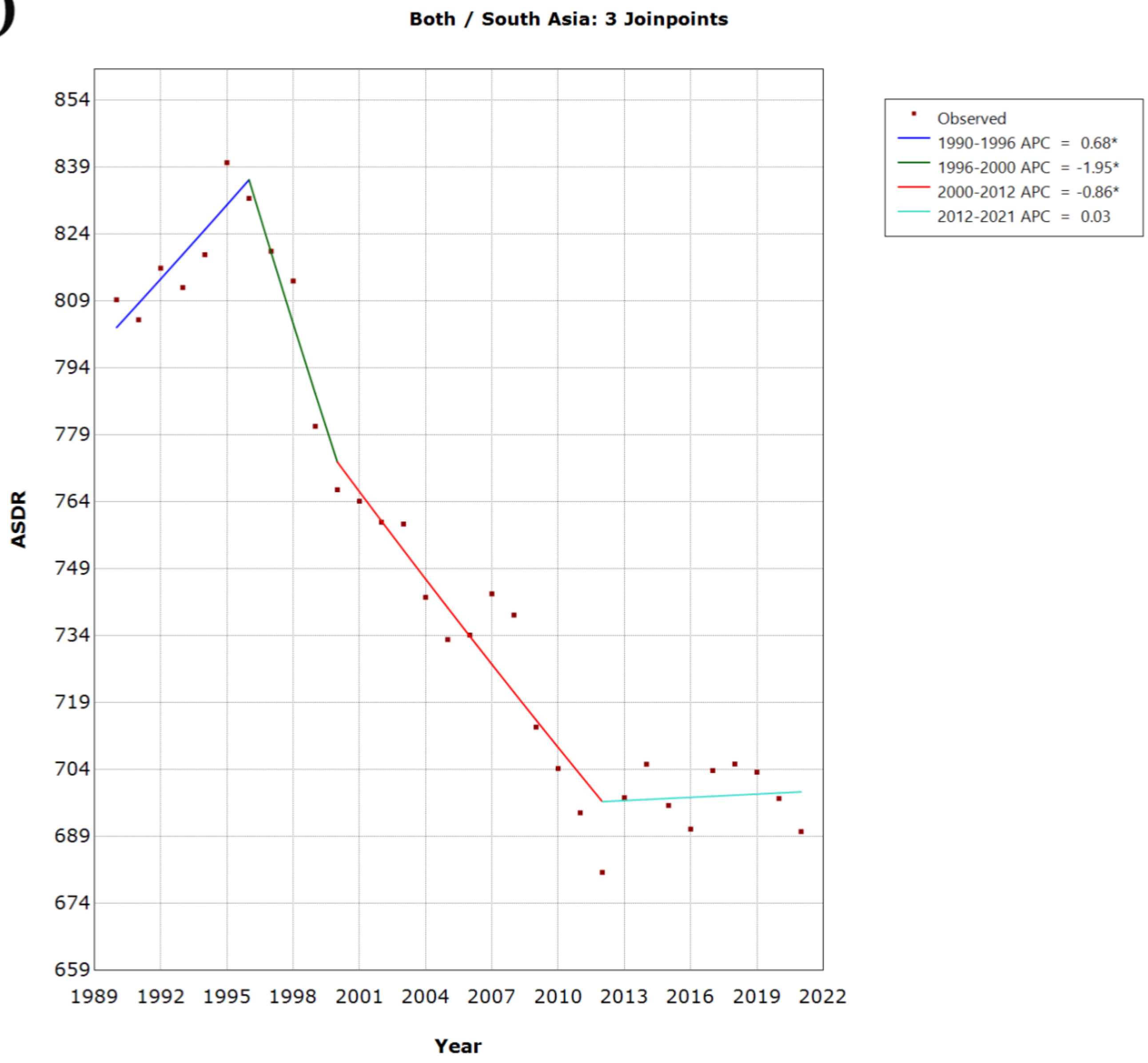

* Indicates that the Annual Percent Change (APC) is significantly different from zero at the alpha = 0.05 level
Final Selected Model: 3 Joinpoints.

P

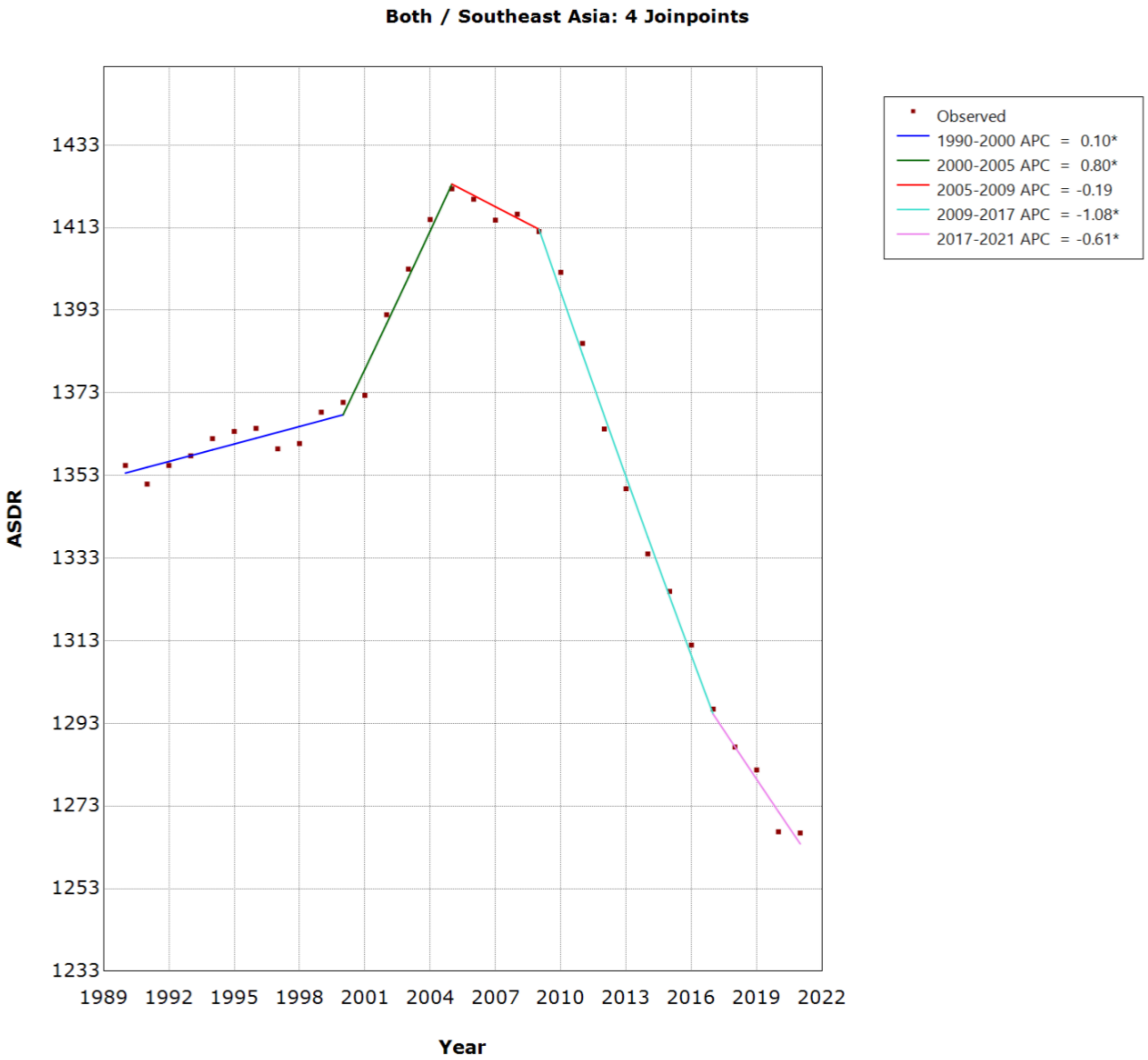

* Indicates that the Annual Percent Change (APC) is significantly different from zero at the alpha = 0.05 level
Final Selected Model: 4 Joinpoints.

Q

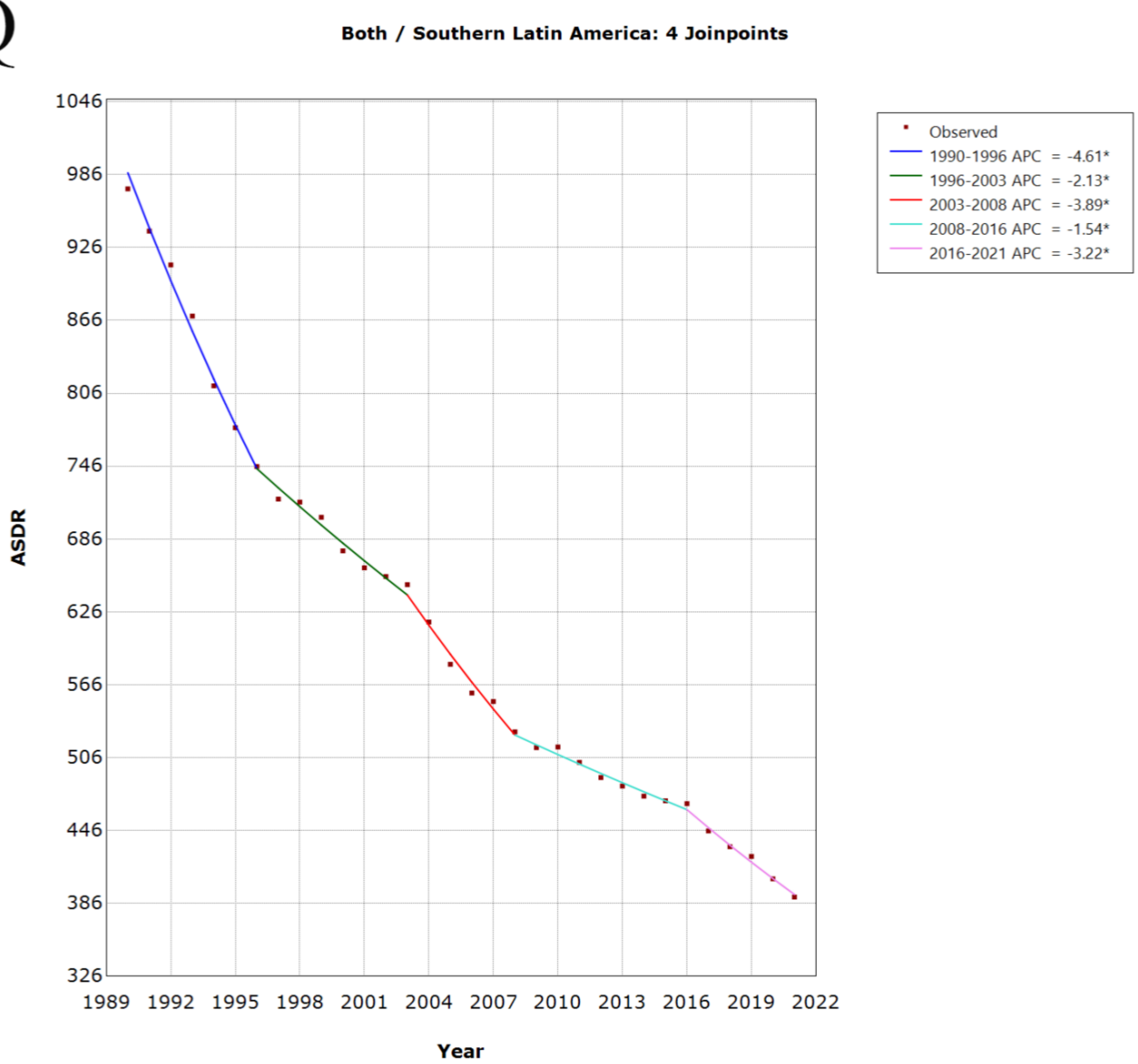

* Indicates that the Annual Percent Change (APC) is significantly different from zero at the alpha = 0.05 level
Final Selected Model: 4 Joinpoints.

R

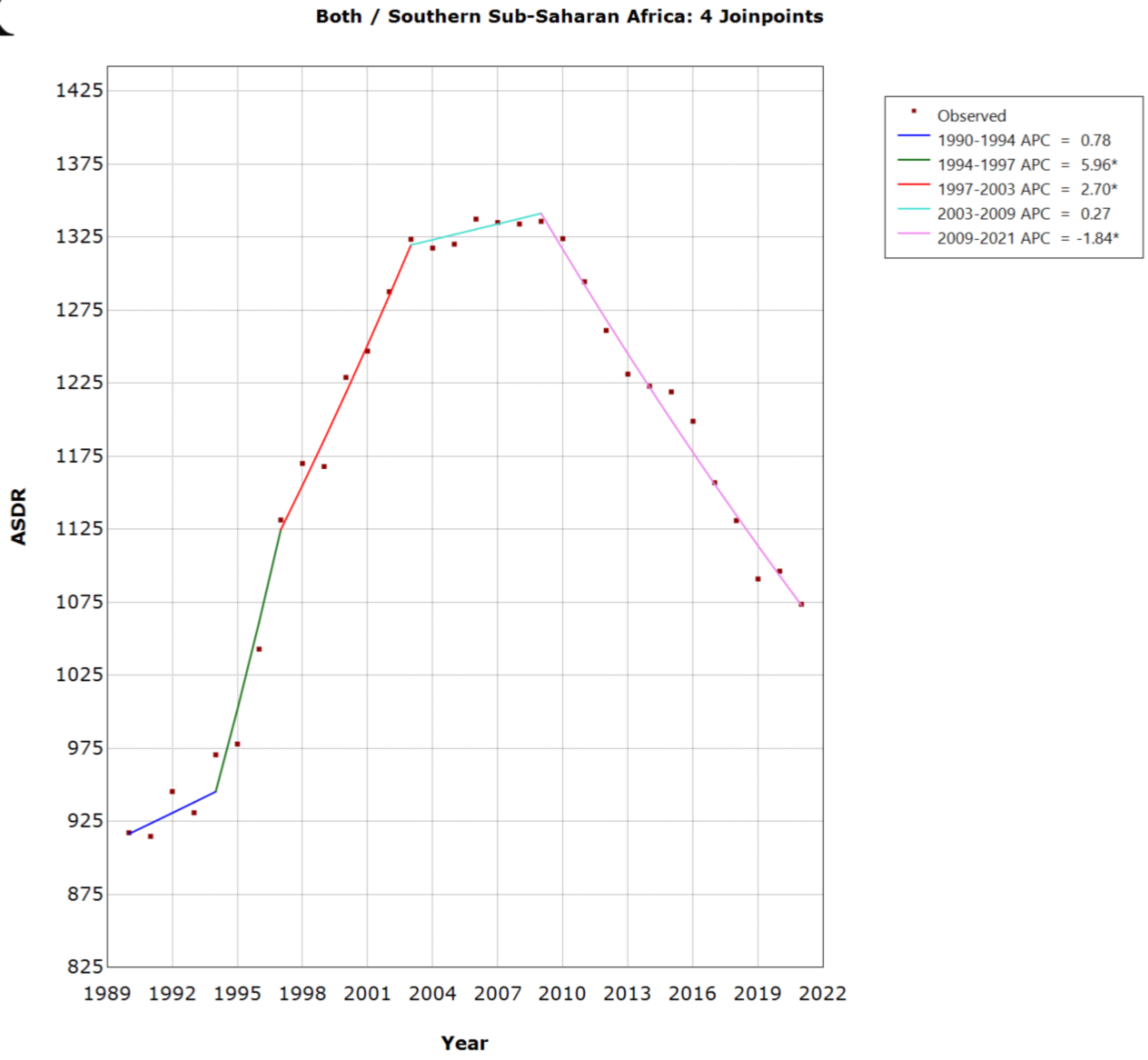

* Indicates that the Annual Percent Change (APC) is significantly different from zero at the alpha = 0.05 level
Final Selected Model: 4 Joinpoints.

S

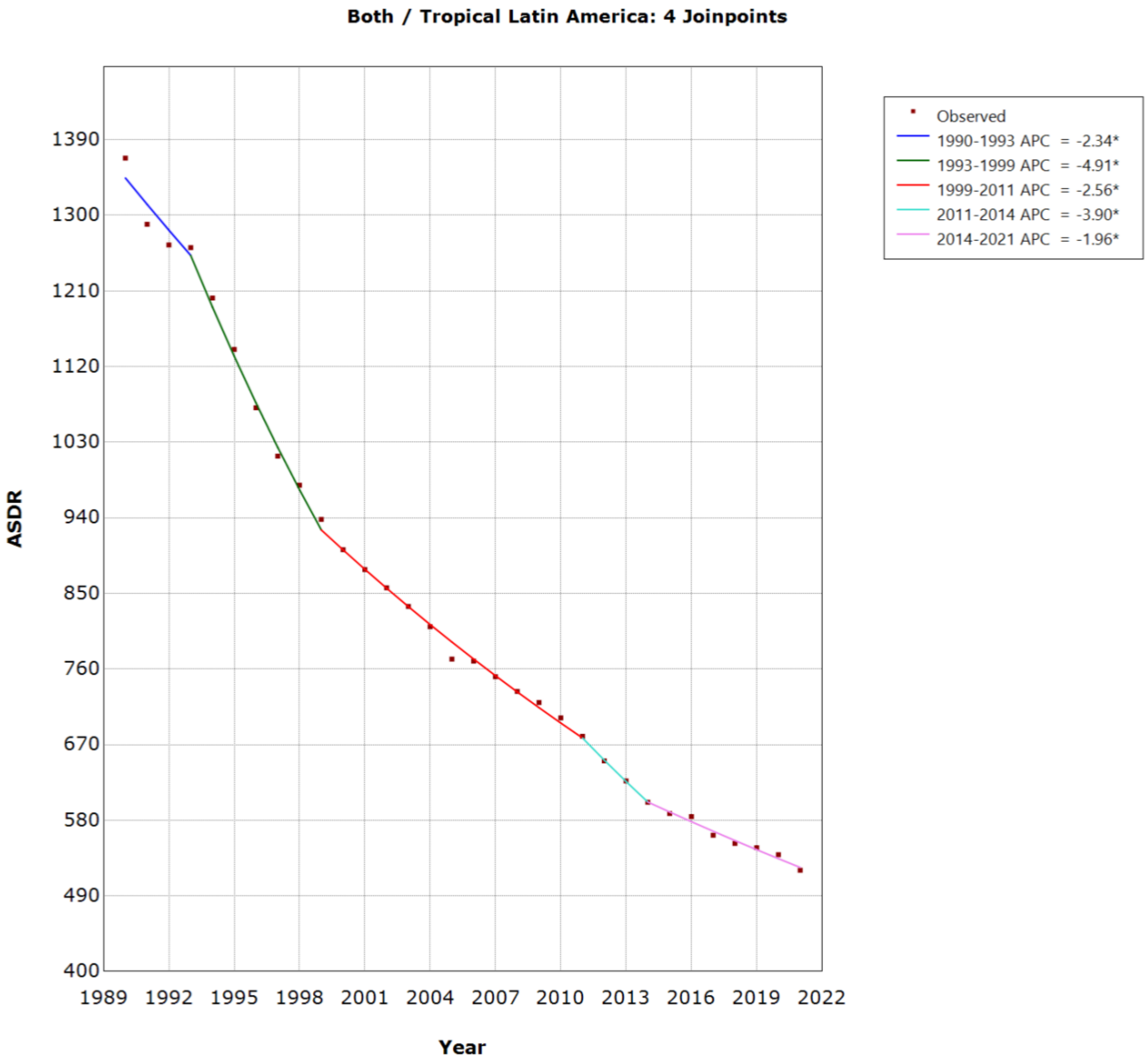

* Indicates that the Annual Percent Change (APC) is significantly different from zero at the alpha = 0.05 level
Final Selected Model: 4 Joinpoints.

T

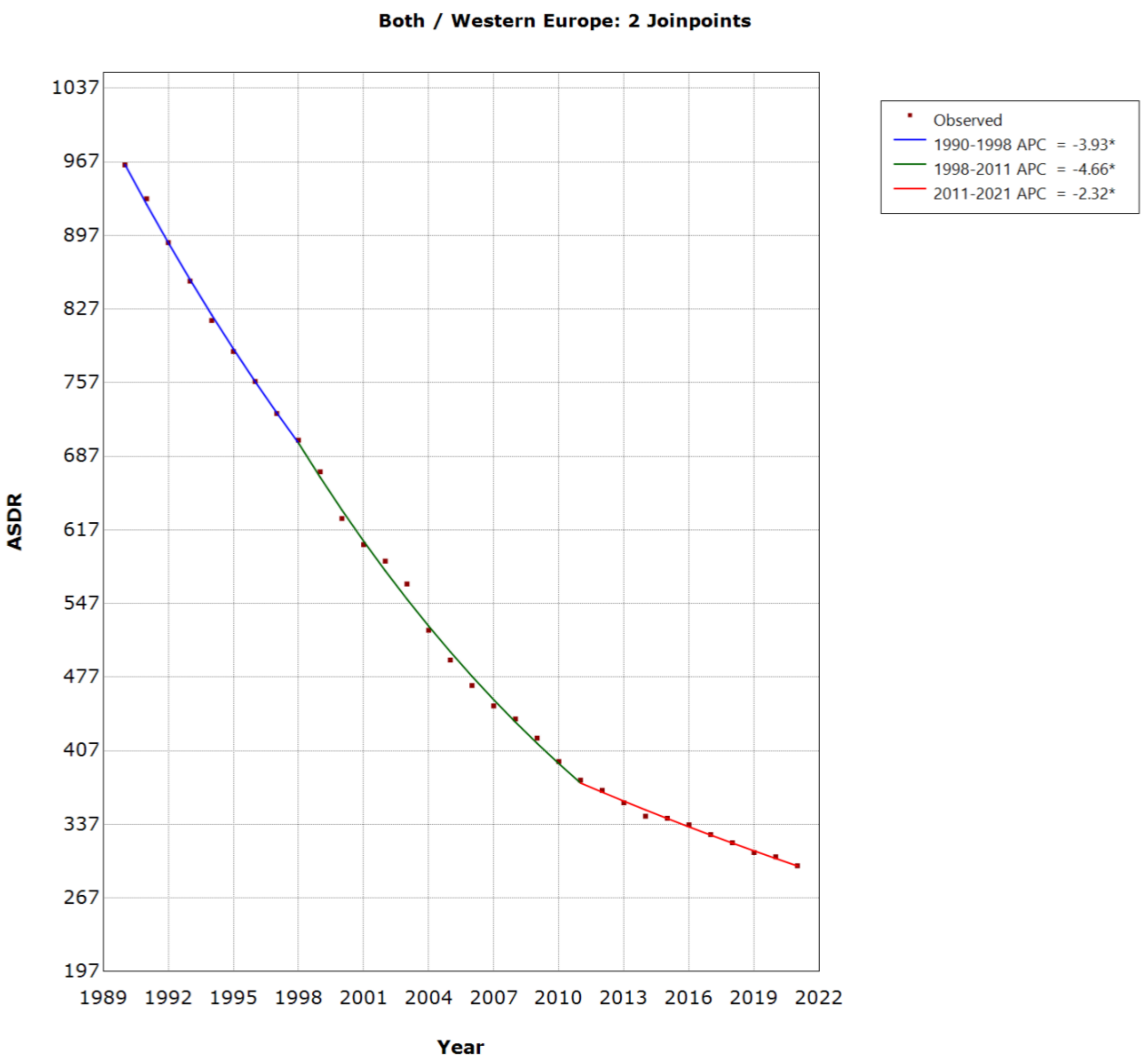

* Indicates that the Annual Percent Change (APC) is significantly different from zero at the alpha = 0.05 level
Final Selected Model: 2 Joinpoints.

U

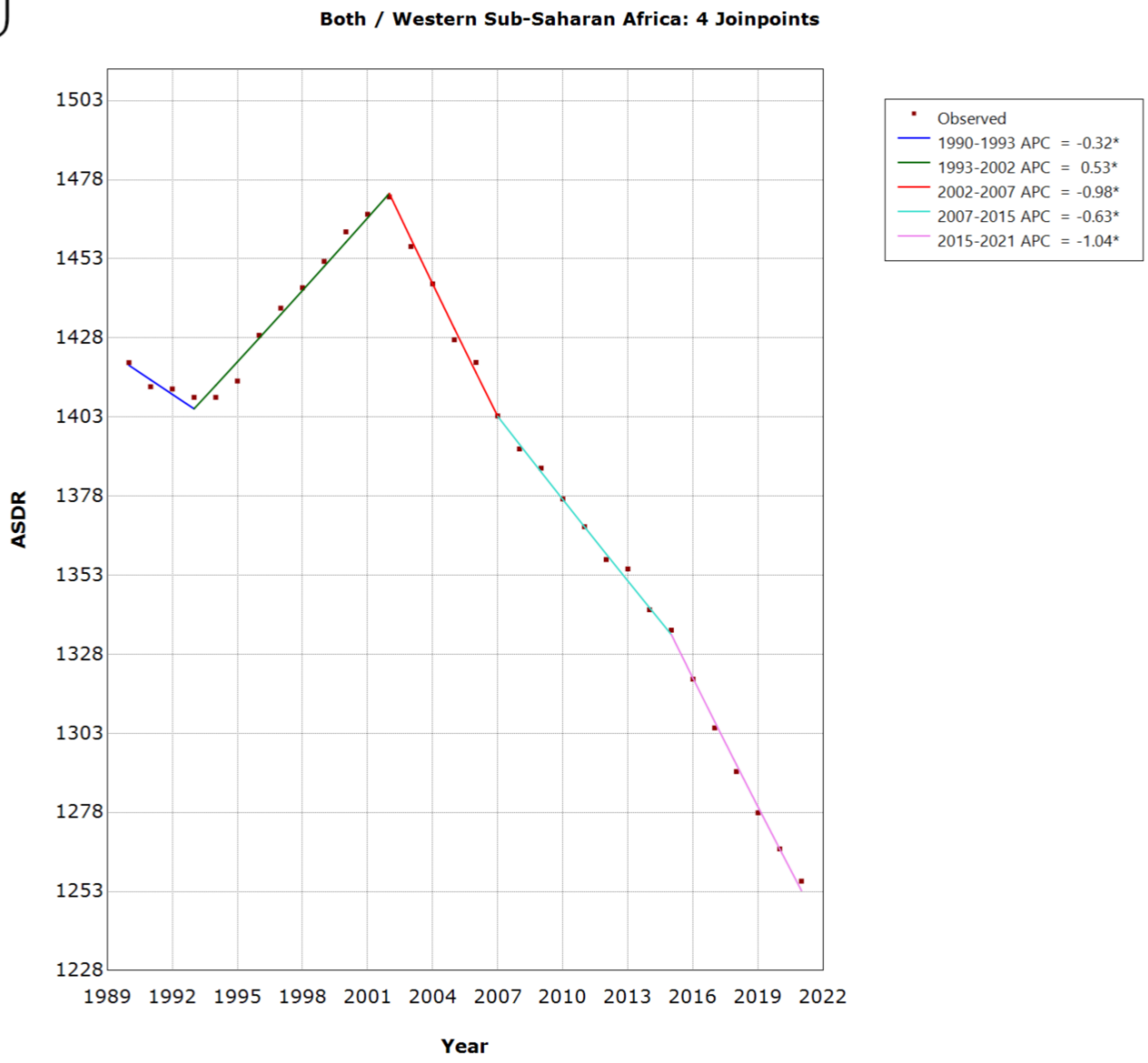

* Indicates that the Annual Percent Change (APC) is significantly different from zero at the alpha = 0.05 level
Final Selected Model: 4 Joinpoints.

Supplementary Figure 3. APC of ASDR of ischemic stroke in each GBD region from 1990 to 2021. A. APC of ASDR of ischemic stroke in Andean Latin America from 1990 to 2021. B. APC of ASDR of ischemic stroke in Australasia from 1990 to 2021. C. APC of ASDR of ischemic stroke in Caribbean from 1990 to 2021. D. APC of ASDR of ischemic stroke in Central Asia from 1990 to 2021. E. APC of ASDR of ischemic stroke in Central Europe from 1990 to 2021. F. APC of ASDR of ischemic stroke in Central Latin America from 1990 to 2021. G. APC of ASDR of ischemic stroke in Central Sub-Saharan Africa from 1990 to 2021. H. APC of ASDR of ischemic stroke in East Asia from 1990 to 2021. I. APC of ASDR of ischemic stroke in Eastern Europe from 1990 to 2021. J. APC of ASDR of ischemic stroke in Eastern Sub-Saharan Africa from 1990 to 2021. K. APC of ASDR of ischemic stroke in High-income Asia Pacific from 1990 to 2021. L. APC of ASDR of ischemic stroke in High-income North America from 1990 to 2021. M. APC of ASDR of ischemic stroke in North Africa and Middle East from 1990 to 2021. N. APC of ASDR of ischemic stroke in Oceania from 1990 to 2021. O. APC of ASDR of ischemic stroke in South Asia from 1990 to 2021. P. APC of ASDR of ischemic stroke in Southeast Asia from 1990 to 2021. Q. APC of ASDR of ischemic stroke in Southern Latin America from 1990 to 2021. R. APC of ASDR of ischemic stroke in Southern Sub-Saharan Africa from 1990 to 2021. S. APC of ASDR of ischemic stroke in Tropical Latin America from 1990 to 2021. T. APC of ASDR of ischemic stroke in each Western Europe 1990 to 2021. U. APC of ASDR of ischemic stroke in each Western Sub-Saharan Africa 1990 to 2021. APC = Annual percent change. ASDR = Age-standardized DALYs rate.

A

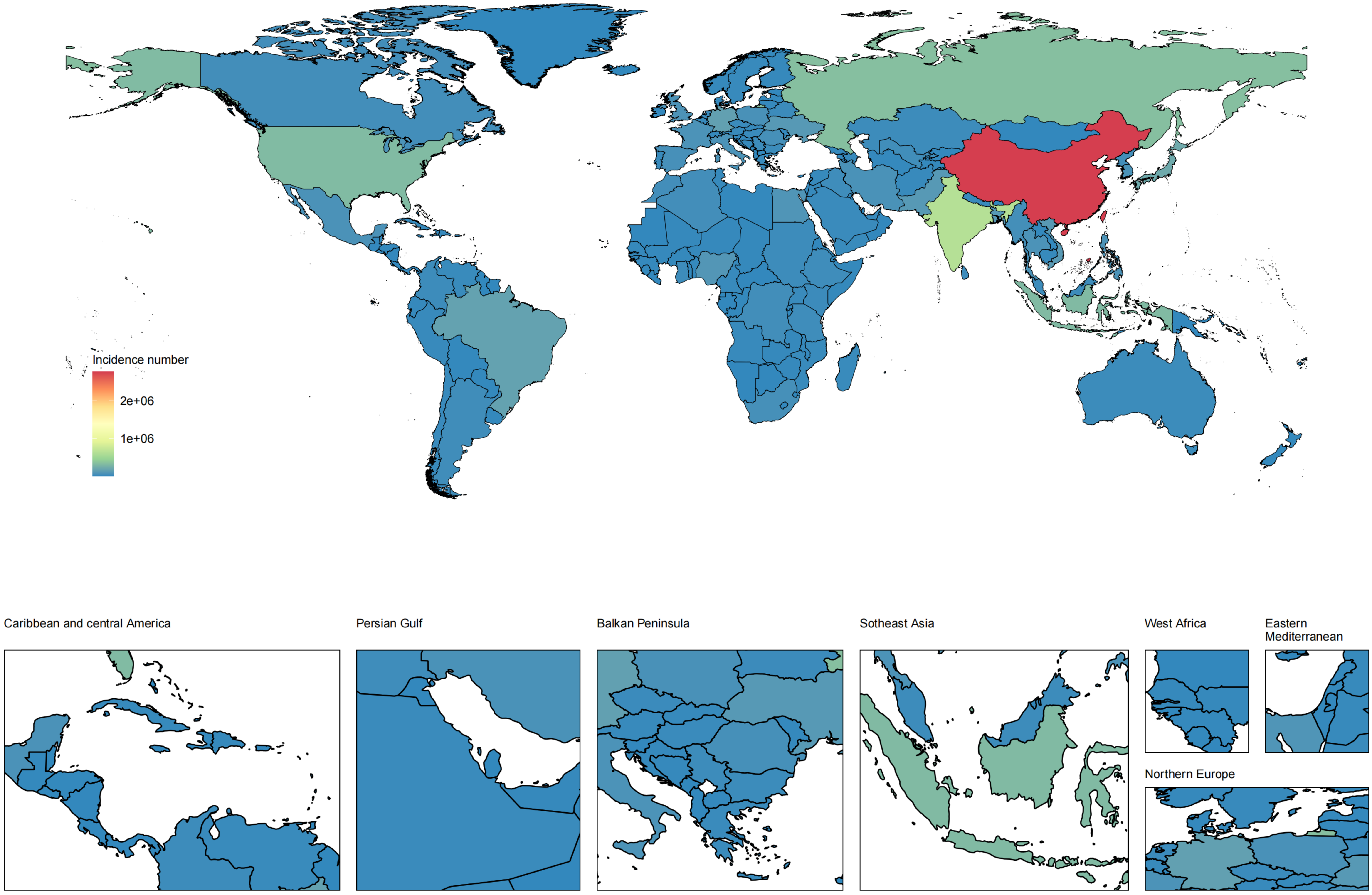
Incidence number
2e+06
1e+06
Caribbean and central America
Persian Gulf
Balkan Peninsula
Sotheast Asia
West Africa
Eastern Mediterranean
Northern Europe

B

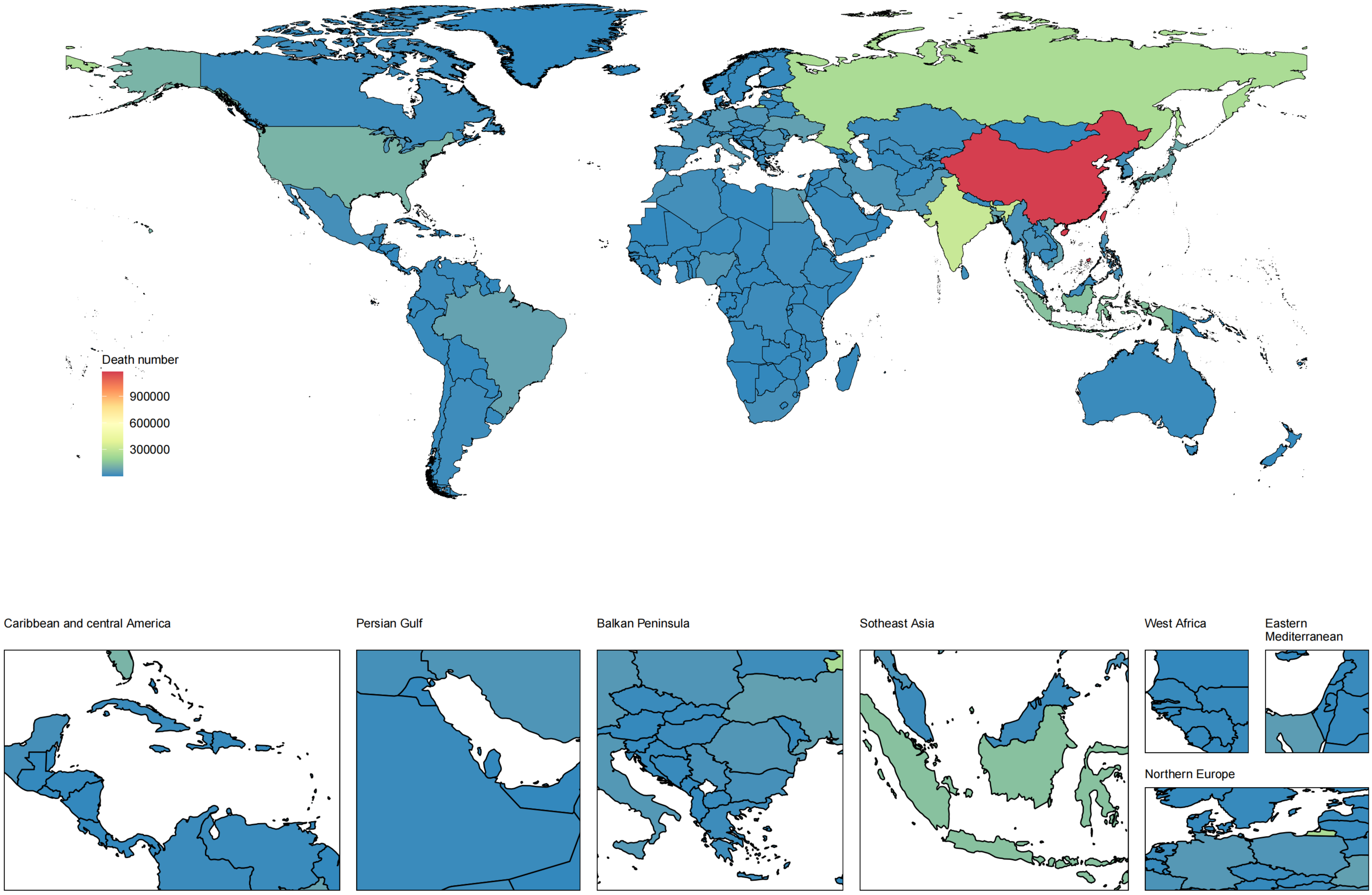

Death number
900000
600000
300000
Caribbean and central America
Persian Gulf
Balkan Peninsula
Sotheast Asia
West Africa
Eastern Mediterranean
Northern Europe

C

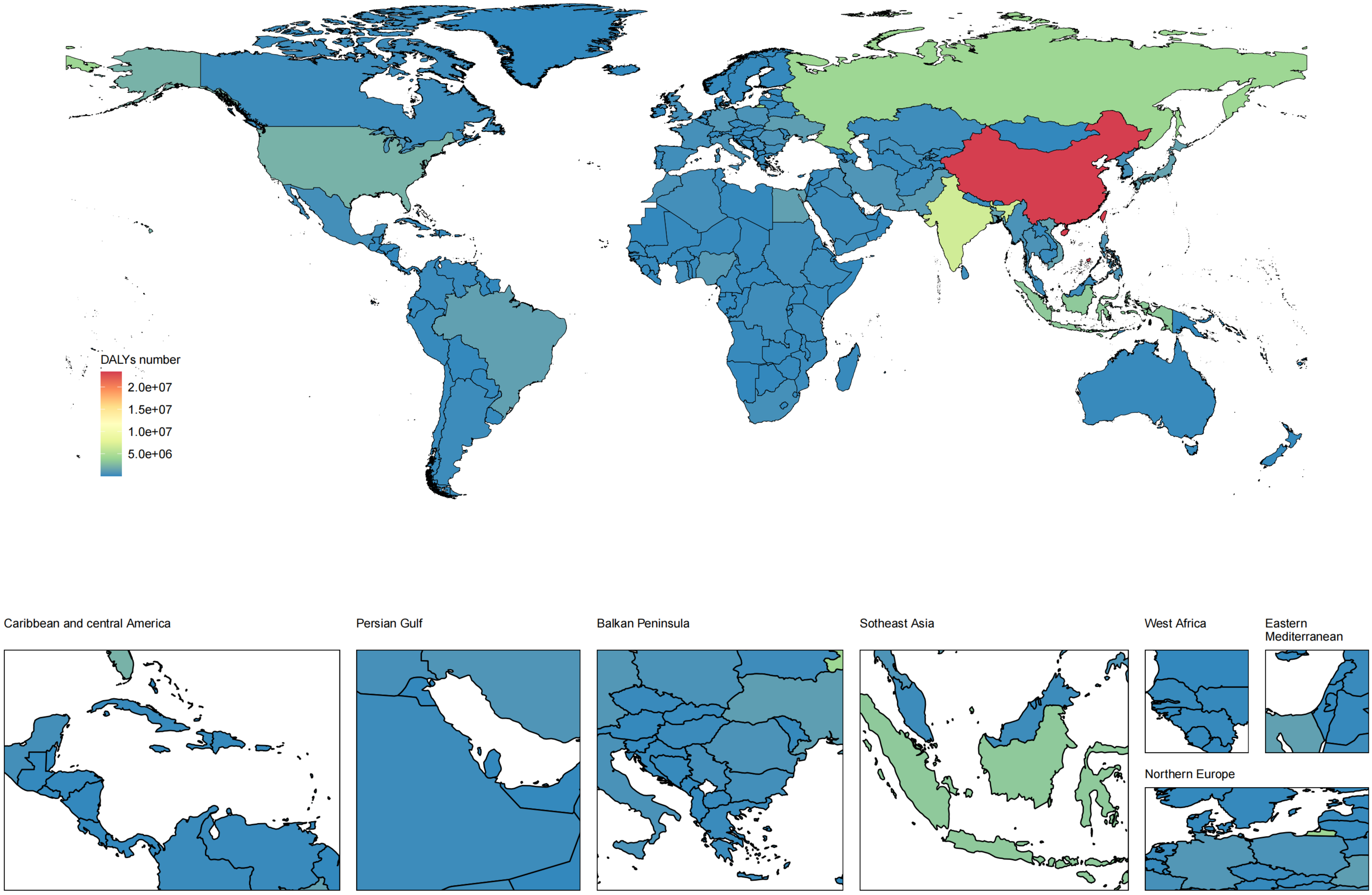
DALYs number
2.0e+07
1.5e+07
1.0e+07
5.0e+06
Caribbean and central America
Persian Gulf
Balkan Peninsula
Sotheast Asia
West Africa
Eastern Mediterranean
Northern Europe

Supplementary Figure 4. Incident, Death, and Disability-Adjusted Life-Years (DALYs) Cases of Ischemic Stroke in 204 Countries and Territories. A. Incident cases. B. Death cases. C. DALYs cases.

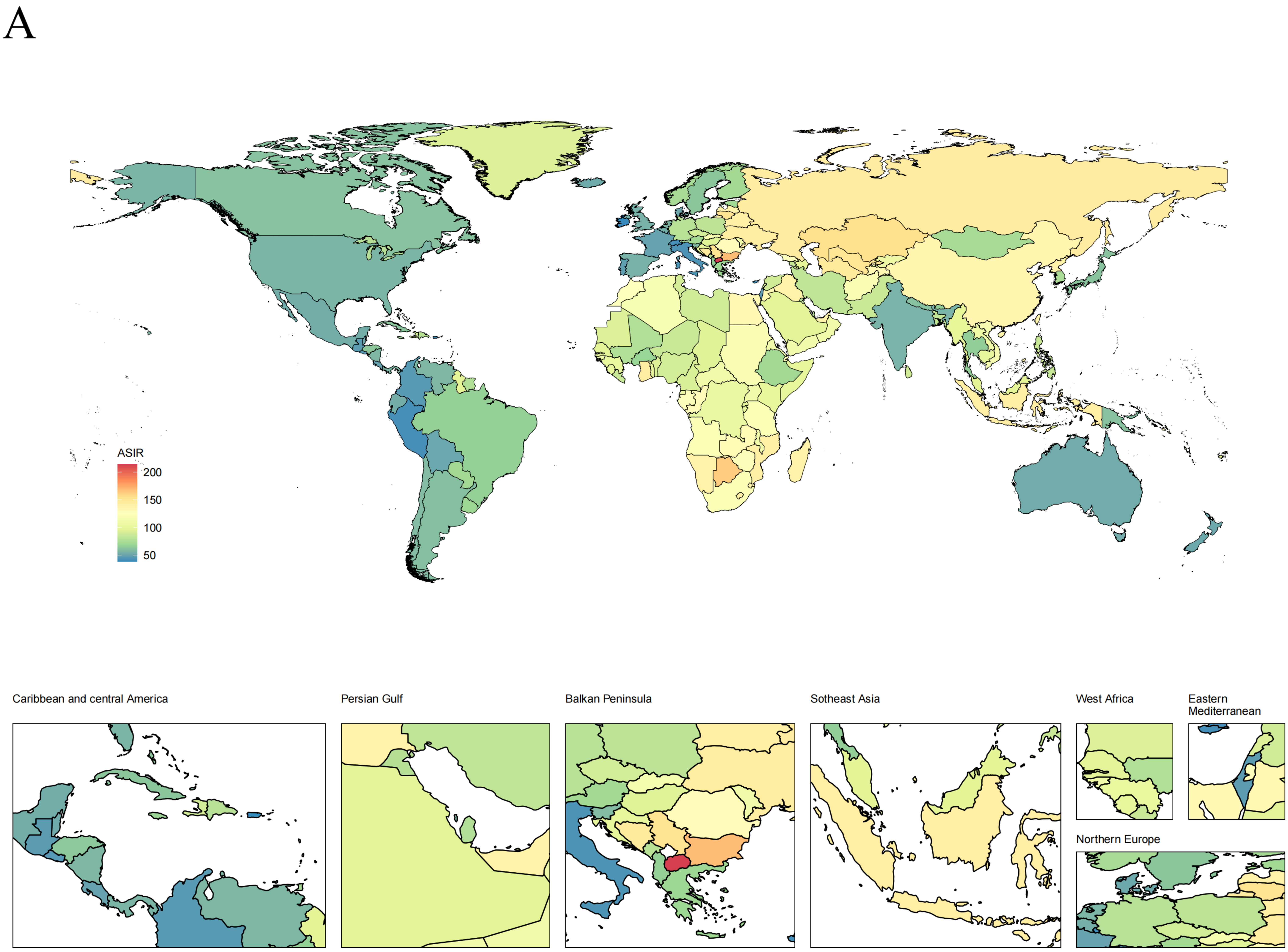
A
ASIR
200
150
100
50
Caribbean and central America
Persian Gulf
Balkan Peninsula
Sotheast Asia
West Africa
Eastern Mediterranean
Northern Europe

B

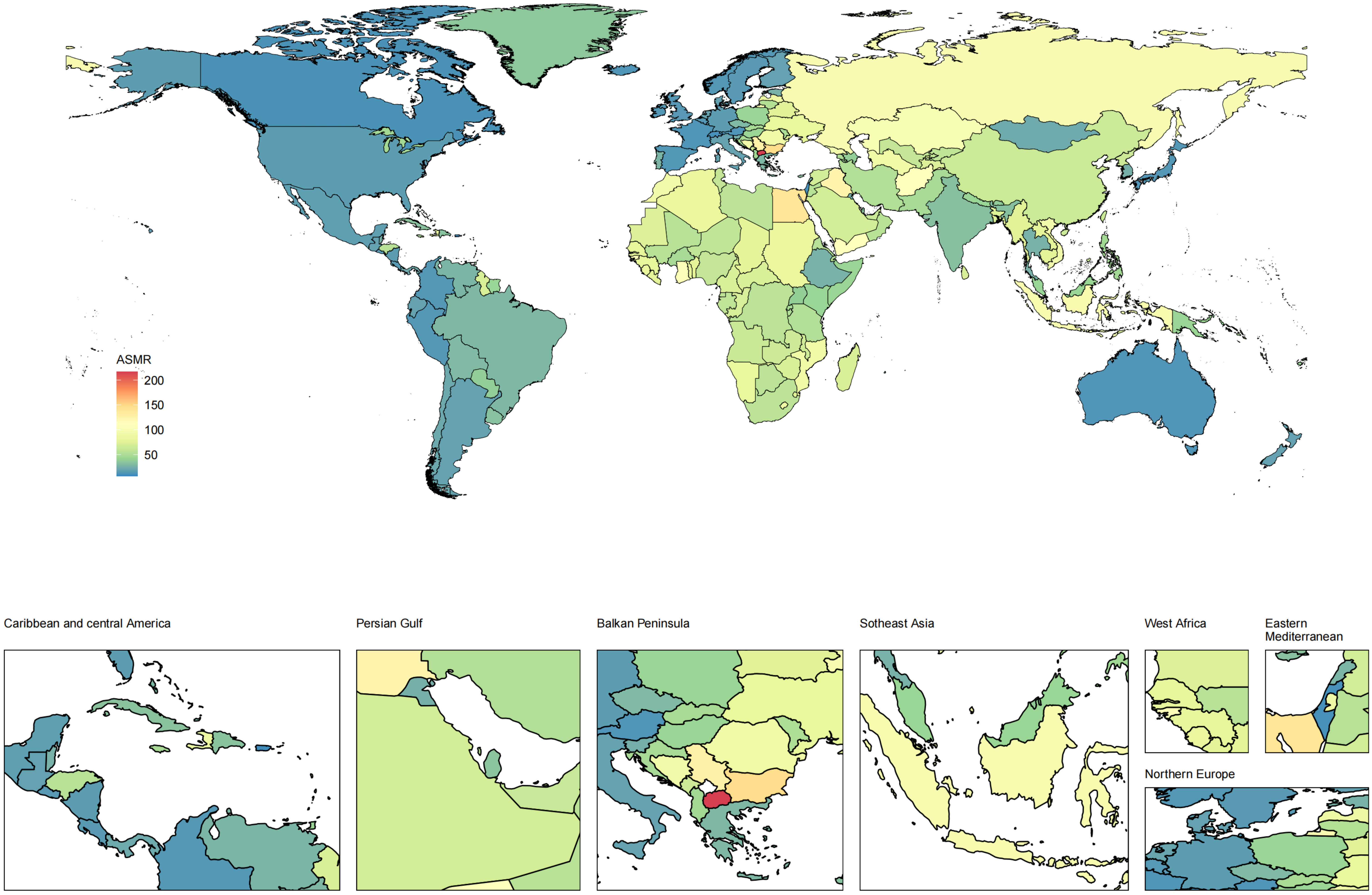
ASMR
200
150
100
50
Caribbean and central America
Persian Gulf
Balkan Peninsula
Sotheast Asia
West Africa
Eastern Mediterranean
Northern Europe

C

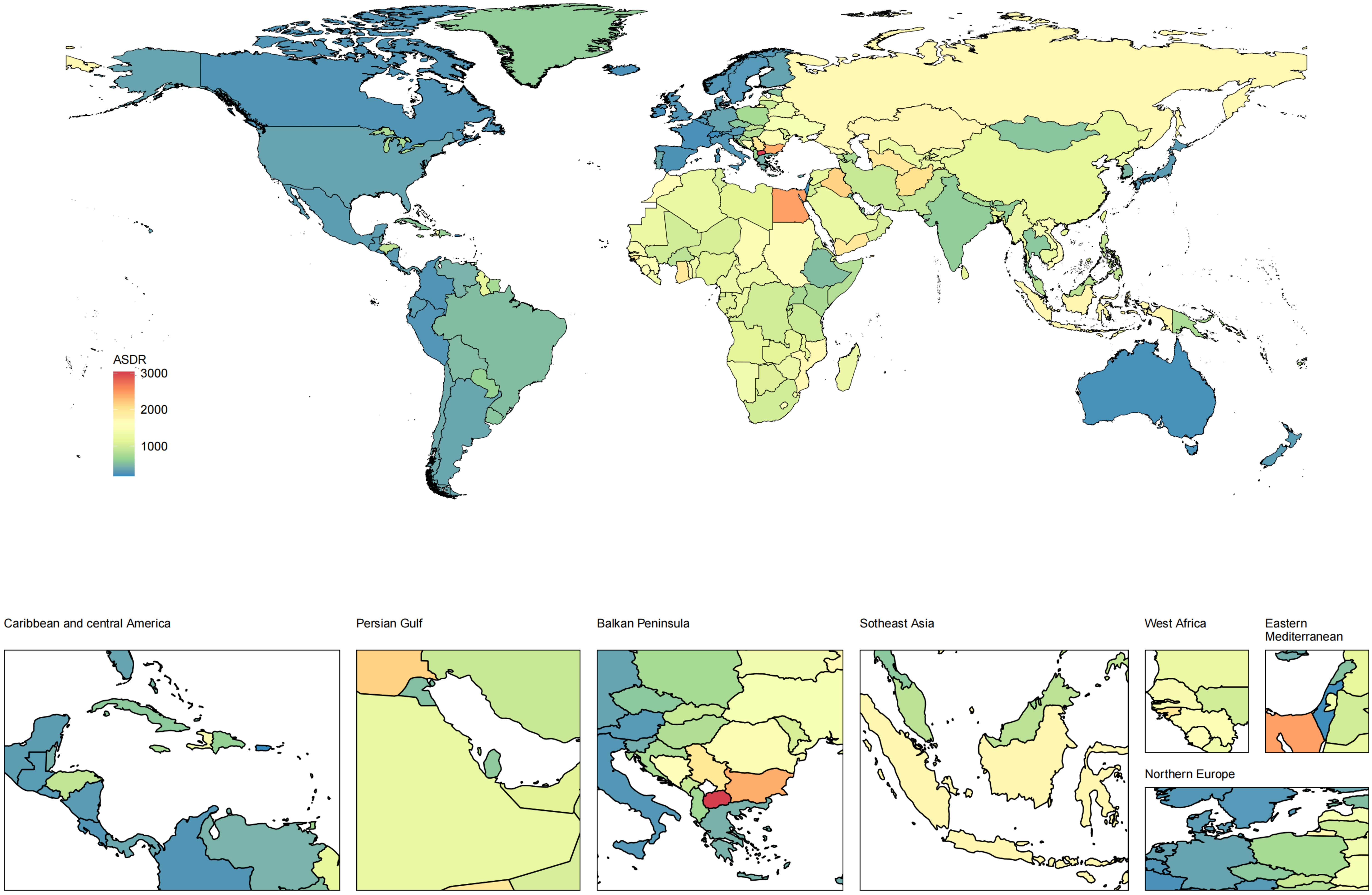
ASDR
3000
2000
1000
Caribbean and central America
Persian Gulf
Balkan Peninsula
Sotheast Asia
West Africa
Eastern Mediterranean
Northern Europe

Supplementary Figure 5. The ASIR, ASMR, and ASDR of Ischemic Stroke in 204 countries and territories. (A) Disease burden of incidence rate. (B) Disease burden of deaths rate. (C) Disease burden of DALYs rate. ASIR = Age-standardized incidence rate. ASMR = Age-standardized mortality rate. ASDR = Age-standardized DALYs rate.

A

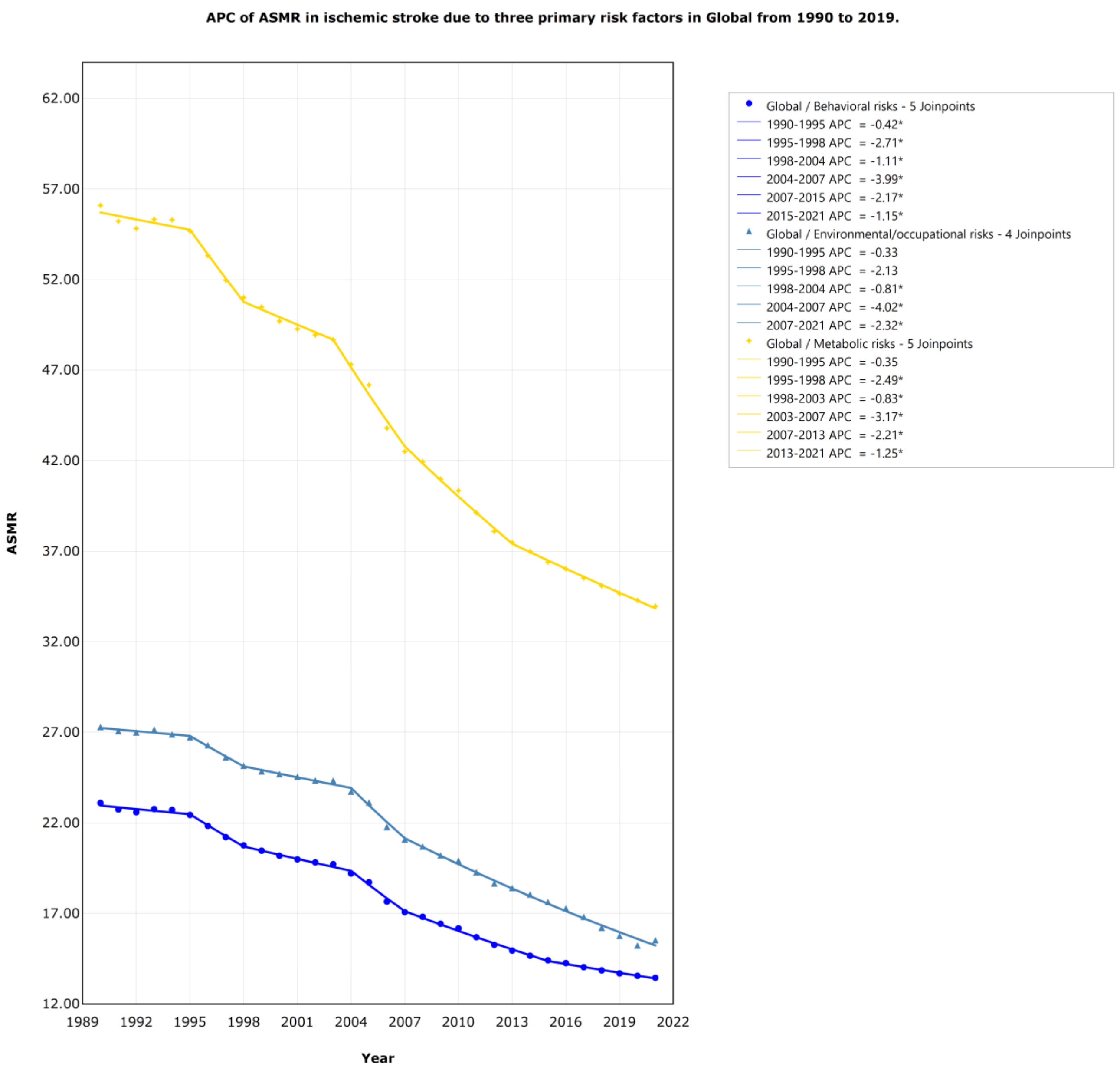
APC of ASMR in ischemic stroke due to three primary risk factors in Global from 1990 to 2019.
ASMR
Year
62.00
57.00
52.00
47.00
42.00
37.00
32.00
27.00
22.00
17.00
12.00
1989
1992
1995
1998
2001
2004
2007
2010
2013
2016
2019
2022
Global / Behavioral risks - 5 Joinpoints
1990-1995 APC = -0.42*
1995-1998 APC = -2.71*
1998-2004 APC = -1.11*
2004-2007 APC = -3.99*
2007-2015 APC = -2.17*
2015-2021 APC = -1.15*
Global / Environmental/occupational risks - 4 Joinpoints
1990-1995 APC = -0.33
1995-1998 APC = -2.13
1998-2004 APC = -0.81*
2004-2007 APC = -4.02*
2007-2021 APC = -2.32*
Global / Metabolic risks - 5 Joinpoints
1990-1995 APC = -0.35
1995-1998 APC = -2.49*
1998-2003 APC = -0.83*
2003-2007 APC = -3.17*
2007-2013 APC = -2.21*
2013-2021 APC = -1.25*

B

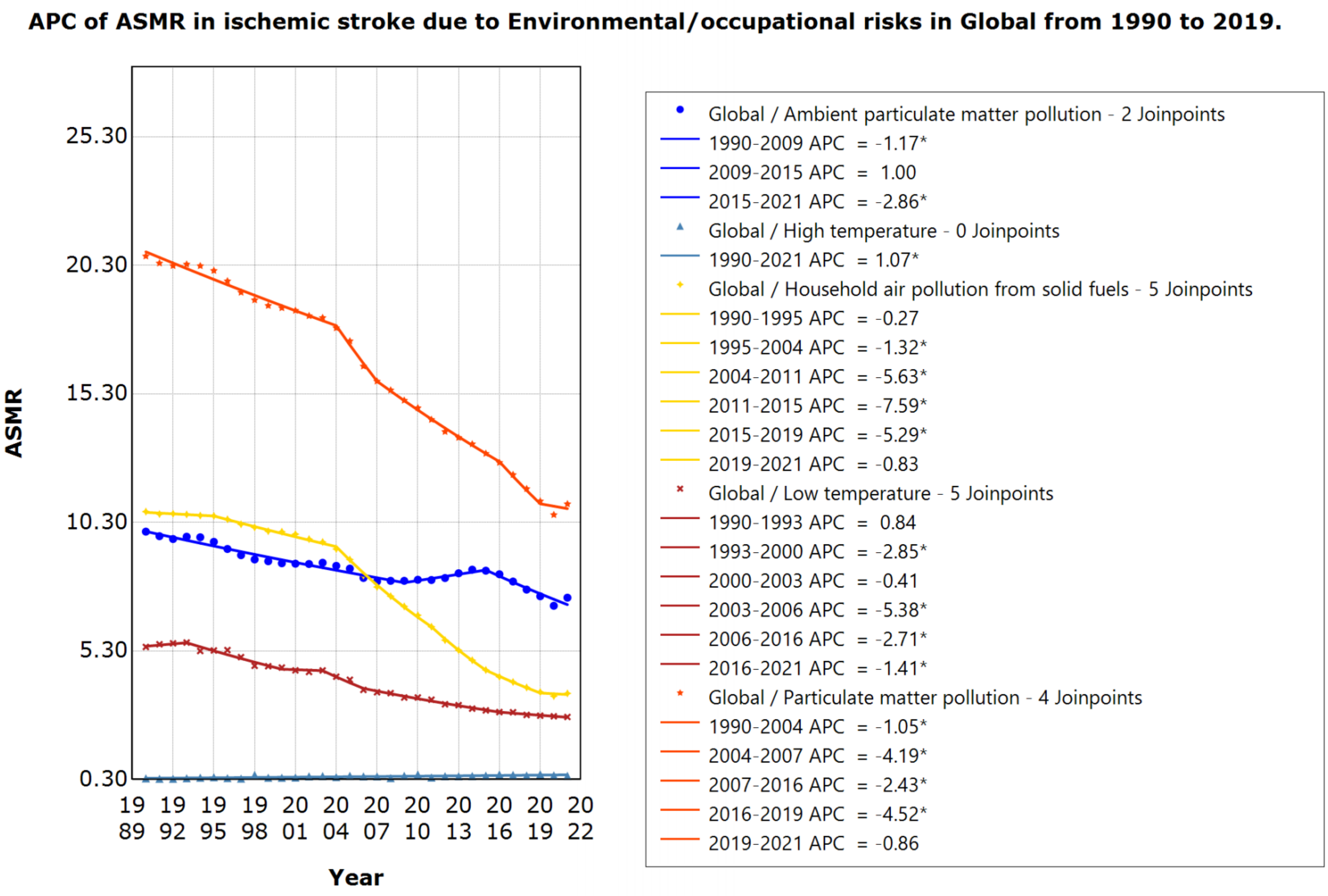
APC of ASMR in ischemic stroke due to Environmental/occupational risks in Global from 1990 to 2019.
ASMR
25.30
20.30
15.30
10.30
5.30
0.30
19 89
19 92
19 95
19 98
20 01
20 04
20 07
20 10
20 13
20 16
20 19
20 22
Year
Global / Ambient particulate matter pollution - 2 Joinpoints
1990-2009 APC = -1.17*
2009-2015 APC = 1.00
2015-2021 APC = -2.86*
Global / High temperature - 0 Joinpoints
1990-2021 APC = 1.07*
Global / Household air pollution from solid fuels - 5 Joinpoints
1990-1995 APC = -0.27
1995-2004 APC = -1.32*
2004-2011 APC = -5.63*
2011-2015 APC = -7.59*
2015-2019 APC = -5.29*
2019-2021 APC = -0.83
Global / Low temperature - 5 Joinpoints
1990-1993 APC = 0.84
1993-2000 APC = -2.85*
2000-2003 APC = -0.41
2003-2006 APC = -5.38*
2006-2016 APC = -2.71*
2016-2021 APC = -1.41*
Global / Particulate matter pollution - 4 Joinpoints
1990-2004 APC = -1.05*
2004-2007 APC = -4.19*
2007-2016 APC = -2.43*
2016-2019 APC = -4.52*
2019-2021 APC = -0.86

C

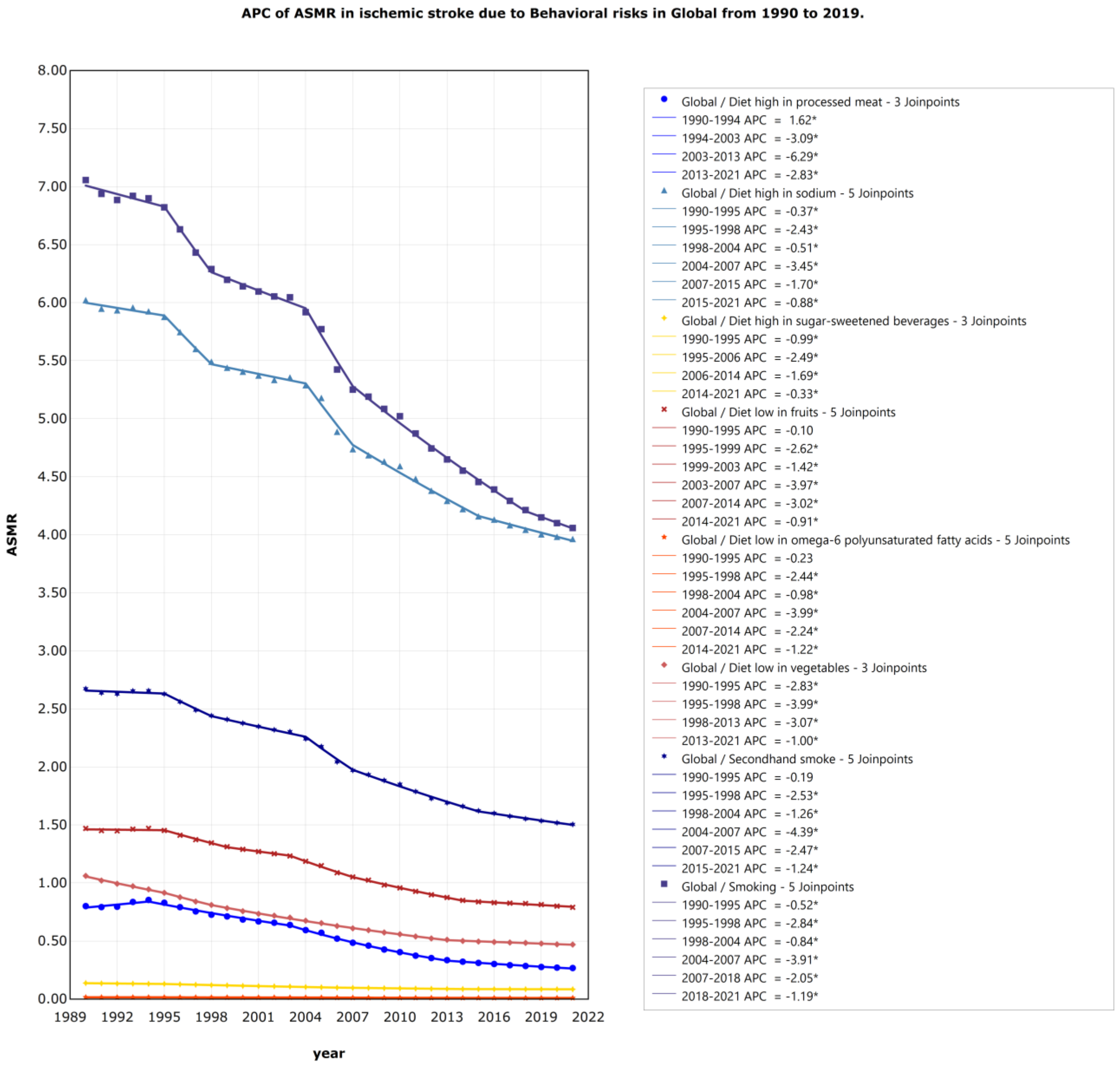

APC of ASMR in ischemic stroke due to Behavioral risks in Global from 1990 to 2019.
ASMR
8.00
7.50
7.00
6.50
6.00
5.50
5.00
4.50
4.00
3.50
3.00
2.50
2.00
1.50
1.00
0.50
0.00
1989
1992
1995
1998
2001
2004
2007
2010
2013
2016
2019
2022
year
Global / Diet high in processed meat - 3 Joinpoints
1990-1994 APC = 1.62*
1994-2003 APC = -3.09*
2003-2013 APC = -6.29*
2013-2021 APC = -2.83*
Global / Diet high in sodium - 5 Joinpoints
1990-1995 APC = -0.37*
1995-1998 APC = -2.43*
1998-2004 APC = -0.51*
2004-2007 APC = -3.45*
2007-2015 APC = -1.70*
2015-2021 APC = -0.88*
Global / Diet high in sugar-sweetened beverages - 3 Joinpoints
1990-1995 APC = -0.99*
1995-2006 APC = -2.49*
2006-2014 APC = -1.69*
2014-2021 APC = -0.33*
Global / Diet low in fruits - 5 Joinpoints
1990-1995 APC = -0.10
1995-1999 APC = -2.62*
1999-2003 APC = -1.42*
2003-2007 APC = -3.97*
2007-2014 APC = -3.02*
2014-2021 APC = -0.91*
Global / Diet low in omega-6 polyunsaturated fatty acids - 5 Joinpoints
1990-1995 APC = -0.23
1995-1998 APC = -2.44*
1998-2004 APC = -0.98*
2004-2007 APC = -3.99*
2007-2014 APC = -2.24*
2014-2021 APC = -1.22*
Global / Diet low in vegetables - 3 Joinpoints
1990-1995 APC = -2.83*
1995-1998 APC = -3.99*
1998-2013 APC = -3.07*
2013-2021 APC = -1.00*
Global / Secondhand smoke - 5 Joinpoints
1990-1995 APC = -0.19
1995-1998 APC = -2.53*
1998-2004 APC = -1.26*
2004-2007 APC = -4.39*
2007-2015 APC = -2.47*
2015-2021 APC = -1.24*
Global / Smoking - 5 Joinpoints
1990-1995 APC = -0.52*
1995-1998 APC = -2.84*
1998-2004 APC = -0.84*
2004-2007 APC = -3.91*
2007-2018 APC = -2.05*
2018-2021 APC = -1.19*

D

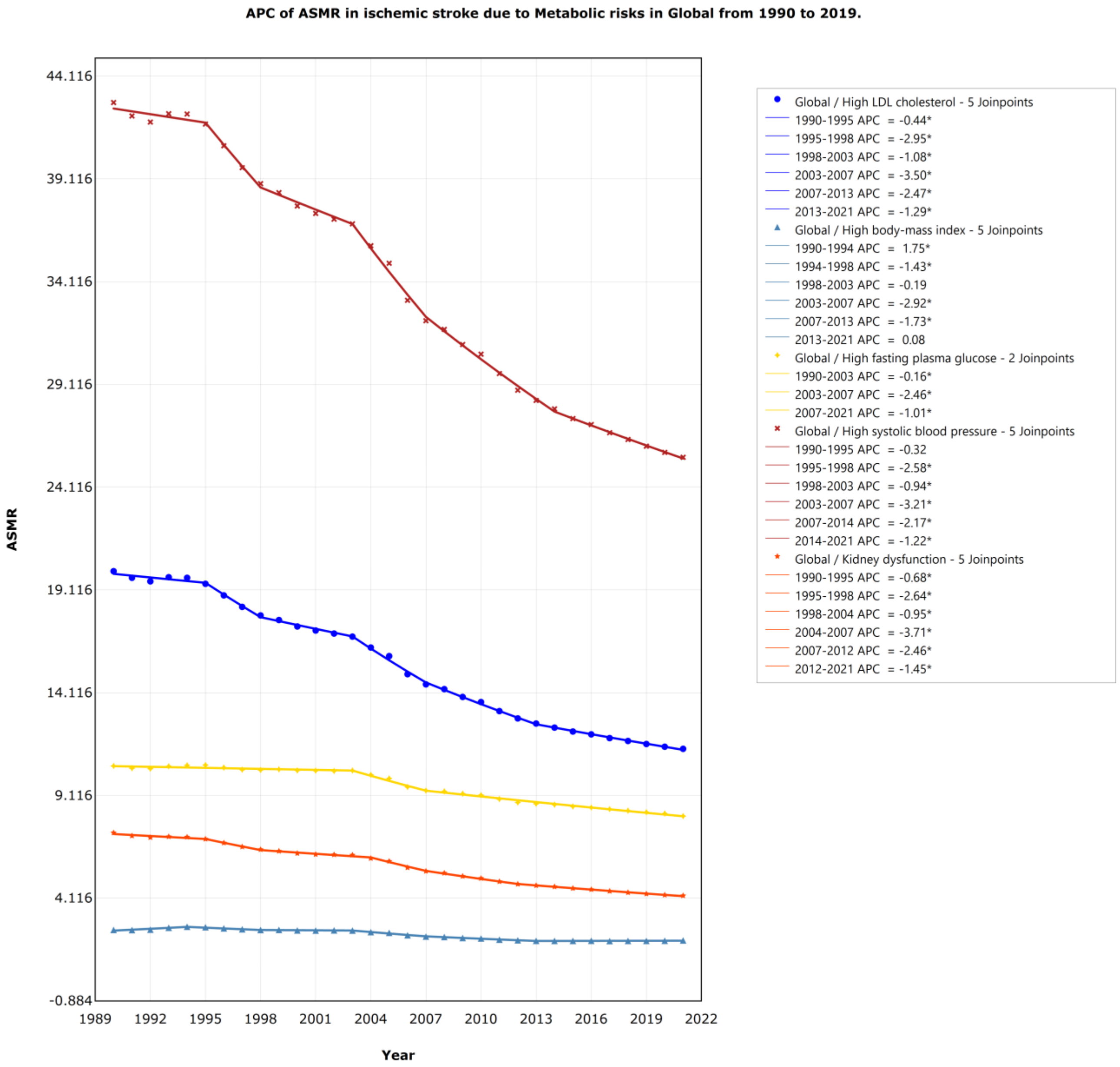
APC of ASMR in ischemic stroke due to Metabolic risks in Global from 1990 to 2019.
ASMR
Year
44.116
39.116
34.116
29.116
24.116
19.116
14.116
9.116
4.116
-0.884
1989
1992
1995
1998
2001
2004
2007
2010
2013
2016
2019
2022
Global / High LDL cholesterol - 5 Joinpoints
1990-1995 APC = -0.44*
1995-1998 APC = -2.95*
1998-2003 APC = -1.08*
2003-2007 APC = -3.50*
2007-2013 APC = -2.47*
2013-2021 APC = -1.29*
Global / High body-mass index - 5 Joinpoints
1990-1994 APC = 1.75*
1994-1998 APC = -1.43*
1998-2003 APC = -0.19
2003-2007 APC = -2.92*
2007-2013 APC = -1.73*
2013-2021 APC = 0.08
Global / High fasting plasma glucose - 2 Joinpoints
1990-2003 APC = -0.16*
2003-2007 APC = -2.46*
2007-2021 APC = -1.01*
Global / High systolic blood pressure - 5 Joinpoints
1990-1995 APC = -0.32
1995-1998 APC = -2.58*
1998-2003 APC = -0.94*
2003-2007 APC = -3.21*
2007-2014 APC = -2.17*
2014-2021 APC = -1.22*
Global / Kidney dysfunction - 5 Joinpoints
1990-1995 APC = -0.68*
1995-1998 APC = -2.64*
1998-2004 APC = -0.95*
2004-2007 APC = -3.71*
2007-2012 APC = -2.46*
2012-2021 APC = -1.45*

Supplementary Figure 6. APC of ASMR for ischemic stroke attributable to each risk factor, 1990-2021. A. APC of ASMR for ischemic stroke attributable to Environmental and occupational risks, behavioral risks, and metabolic risks, 1990-2021. B. APC of ASMR for ischemic stroke attributable to various Environmental and occupational risks, 1990-2021. C. APC of ASMR for ischemic stroke attributable to various behavioral risks, 1990-2021. D. APC of ASMR for ischemic stroke attributable to various metabolic risks, 1990-2021.
