# Supplementary material for: Global, regional, and national epidemiology of ischemic stroke from 1990 to 2021
Source: Eur J Neurol. 2024 Sep 17;31(12):e16481. doi: 10.1111/ene.16481 (PMC11555022; doi:10.1111/ene.16481)
Supplement: Supplementary file 2 — TABLE S1. The AAPC of incidence, death, and DALYs of ischemic stroke at global, regional, and national levels. AAPC, average annual percent change; DALY, disability‐adjusted life year. [file ENE-31-e16481-s008.docx]

Supplementary Table 1. The AAPC of incidence, death, and DALYs of Ischemic Stroke in global, Regional, and National levels. AAPC = Average Annual percent change. DALYs = Disability-Adjusted Life Years.

| **measure** | **location** | **sex** | **AAPC (95% CI)** | **Test Statistic** | **P-Value** |
| --- | --- | --- | --- | --- | --- |
| Incidence | Global | Both | -0.57 (-0.66 to -0.48) | -12.703 | <0.001 |
| Incidence | Global | Female | -0.72 (-0.78 to -0.65) | -21.943 | <0.001 |
| Incidence | Global | Male | -0.42 (-0.50 to -0.35) | -10.928 | <0.001 |
| Incidence | Central Europe Eastern Europe and Central Asia | Both | -1.05 (-1.14 to -0.95) | -20.628 | <0.001 |
| Incidence | Global | Both | -0.57 (-0.66 to -0.48) | -12.703 | <0.001 |
| Incidence | High-income | Both | -1.80 (-1.85 to -1.74) | -64.656 | <0.001 |
| Incidence | Latin America and Caribbean | Both | -1.51 (-1.56 to -1.46) | -60.274 | <0.001 |
| Incidence | North Africa and Middle East | Both | -0.41 (-0.46 to -0.37) | -18.917 | <0.001 |
| Incidence | South Asia | Both | -0.56 (-0.60 to -0.53) | -30.239 | <0.001 |
| Incidence | Southeast Asia East Asia and Oceania | Both | 0.72 (0.65 to 0.80) | 19.341 | <0.001 |
| Incidence | Sub-Saharan Africa | Both | -0.24 (-0.26 to -0.22) | -23.964 | <0.001 |
| Incidence | Andean Latin America | Both | -1.02 (-1.10 to -0.95) | -25.161 | <0.001 |
| Incidence | Australasia | Both | -1.75 (-1.80 to -1.70) | -69.269 | <0.001 |
| Incidence | Caribbean | Both | -0.40 (-0.41 to -0.39) | -65.442 | <0.001 |
| Incidence | Central Asia | Both | -0.22 (-0.27 to -0.17) | -8.244 | <0.001 |
| Incidence | Central Europe | Both | -1.27 (-1.34 to -1.19) | -33.731 | <0.001 |
| Incidence | Central Latin America | Both | -1.41 (-1.48 to -1.34) | -41.599 | <0.001 |
| Incidence | Central Sub-Saharan Africa | Both | -0.39 (-0.42 to -0.35) | -19.444 | <0.001 |
| Incidence | East Asia | Both | 0.90 (0.82 to 0.99) | 20.426 | <0.001 |
| Incidence | Eastern Europe | Both | -1.06 (-1.21 to -0.90) | -13.448 | <0.001 |
| Incidence | Eastern Sub-Saharan Africa | Both | -0.22 (-0.26 to -0.17) | -9.501 | <0.001 |
| Incidence | High-income Asia Pacific | Both | -2.00 (-2.27 to -1.73) | -14.464 | <0.001 |
| Incidence | High-income North America | Both | -1.46 (-1.50 to -1.42) | -67.593 | <0.001 |
| Incidence | North Africa and Middle East | Both | -0.41 (-0.46 to -0.37) | -18.917 | <0.001 |
| Incidence | Oceania | Both | -0.36 (-0.38 to -0.33) | -28.501 | <0.001 |
| Incidence | South Asia | Both | -0.56 (-0.60 to -0.53) | -30.239 | <0.001 |
| Incidence | Southeast Asia | Both | 0.00 (-0.03 to 0.03) | -0.025 | 0.98 |
| Incidence | Southern Latin America | Both | -1.56 (-1.62 to -1.49) | -47.507 | <0.001 |
| Incidence | Southern Sub-Saharan Africa | Both | -0.01 (-0.18 to 0.16) | -0.112 | 0.911 |
| Incidence | Tropical Latin America | Both | -1.87 (-1.93 to -1.81) | -62.097 | <0.001 |
| Incidence | Western Europe | Both | -1.92 (-1.96 to -1.88) | -90.796 | <0.001 |
| Incidence | Western Sub-Saharan Africa | Both | -0.31 (-0.34 to -0.28) | -19.412 | <0.001 |
| Incidence | Afghanistan | Both | -0.23 (-0.26 to -0.21) | -18.494 | <0.001 |
| Incidence | Albania | Both | -0.37 (-0.46 to -0.29) | -8.883 | <0.001 |
| Incidence | Algeria | Both | -0.48 (-0.62 to -0.34) | -6.59 | <0.001 |
| Incidence | American Samoa | Both | -0.62 (-0.67 to -0.57) | -23.683 | <0.001 |
| Incidence | Andorra | Both | -1.19 (-1.26 to -1.11) | -30.596 | <0.001 |
| Incidence | Angola | Both | -0.38 (-0.44 to -0.33) | -13.025 | <0.001 |
| Incidence | Antigua and Barbuda | Both | -0.71 (-0.72 to -0.69) | -98.726 | <0.001 |
| Incidence | Argentina | Both | -1.49 (-1.54 to -1.45) | -64.401 | <0.001 |
| Incidence | Armenia | Both | -1.44 (-1.54 to -1.33) | -26.758 | <0.001 |
| Incidence | Australia | Both | -1.75 (-1.82 to -1.69) | -52.407 | <0.001 |
| Incidence | Austria | Both | -0.94 (-1.20 to -0.68) | -7.016 | <0.001 |
| Incidence | Azerbaijan | Both | 0.26 (0.18 to 0.33) | 6.579 | <0.001 |
| Incidence | Bahamas | Both | -0.61 (-0.63 to -0.58) | -44.531 | <0.001 |
| Incidence | Bahrain | Both | -1.11 (-1.14 to -1.08) | -67.655 | <0.001 |
| Incidence | Bangladesh | Both | -0.04 (-0.09 to 0.00) | -2.059 | 0.039 |
| Incidence | Barbados | Both | -0.82 (-0.85 to -0.78) | -47.553 | <0.001 |
| Incidence | Belarus | Both | -0.86 (-0.92 to -0.79) | -26.595 | <0.001 |
| Incidence | Belgium | Both | -1.93 (-2.01 to -1.86) | -49.48 | <0.001 |
| Incidence | Belize | Both | -0.32 (-0.37 to -0.26) | -11.475 | <0.001 |
| Incidence | Benin | Both | -0.58 (-0.61 to -0.55) | -37.146 | <0.001 |
| Incidence | Bermuda | Both | -1.49 (-1.54 to -1.44) | -59.01 | <0.001 |
| Incidence | Bhutan | Both | -0.30 (-0.36 to -0.24) | -10.036 | <0.001 |
| Incidence | Bolivia (Plurinational State of) | Both | -0.83 (-0.90 to -0.76) | -22.844 | <0.001 |
| Incidence | Bosnia and Herzegovina | Both | -0.47 (-0.62 to -0.32) | -6.143 | <0.001 |
| Incidence | Botswana | Both | 0.15 (-0.02 to 0.32) | 1.736 | 0.083 |
| Incidence | Brazil | Both | -1.89 (-1.95 to -1.84) | -61.598 | <0.001 |
| Incidence | Brunei Darussalam | Both | -1.87 (-1.98 to -1.76) | -33.171 | <0.001 |
| Incidence | Bulgaria | Both | -0.11 (-0.15 to -0.06) | -4.783 | <0.001 |
| Incidence | Burkina Faso | Both | -0.22 (-0.26 to -0.19) | -14.283 | <0.001 |
| Incidence | Burundi | Both | -1.08 (-1.12 to -1.03) | -50.12 | <0.001 |
| Incidence | Cabo Verde | Both | 0.27 (0.21 to 0.33) | 8.68 | <0.001 |
| Incidence | Cambodia | Both | 0.07 (0.05 to 0.09) | 6.114 | <0.001 |
| Incidence | Cameroon | Both | 0.12 (0.04 to 0.19) | 3.047 | 0.002 |
| Incidence | Canada | Both | -1.62 (-1.69 to -1.54) | -43.291 | <0.001 |
| Incidence | Central African Republic | Both | -0.23 (-0.29 to -0.17) | -7.255 | <0.001 |
| Incidence | Chad | Both | -0.15 (-0.17 to -0.12) | -11.257 | <0.001 |
| Incidence | Chile | Both | -1.50 (-1.54 to -1.46) | -73.228 | <0.001 |
| Incidence | China | Both | 0.97 (0.88 to 1.06) | 20.856 | <0.001 |
| Incidence | Colombia | Both | -1.96 (-2.01 to -1.91) | -82.832 | <0.001 |
| Incidence | Comoros | Both | -0.61 (-0.69 to -0.54) | -15.413 | <0.001 |
| Incidence | Congo | Both | -0.51 (-0.57 to -0.45) | -17.031 | <0.001 |
| Incidence | Cook Islands | Both | -0.24 (-0.26 to -0.21) | -18.426 | <0.001 |
| Incidence | Costa Rica | Both | -1.07 (-1.12 to -1.02) | -41.587 | <0.001 |
| Incidence | C?te d'Ivoire | Both | -0.51 (-0.56 to -0.46) | -19.167 | <0.001 |
| Incidence | Croatia | Both | -1.57 (-1.59 to -1.54) | -102.214 | <0.001 |
| Incidence | Cuba | Both | -0.45 (-0.51 to -0.39) | -14.46 | <0.001 |
| Incidence | Cyprus | Both | -2.29 (-2.72 to -1.86) | -10.318 | <0.001 |
| Incidence | Czechia | Both | -2.40 (-2.49 to -2.32) | -55.178 | <0.001 |
| Incidence | Democratic People's Republic of Korea | Both | 0.12 (0.09 to 0.14) | 8.521 | <0.001 |
| Incidence | Democratic Republic of the Congo | Both | -0.40 (-0.44 to -0.36) | -21.397 | <0.001 |
| Incidence | Denmark | Both | -2.34 (-2.40 to -2.28) | -77.633 | <0.001 |
| Incidence | Djibouti | Both | -0.06 (-0.13 to 0.00) | -1.917 | 0.055 |
| Incidence | Dominica | Both | -0.27 (-0.28 to -0.26) | -49.43 | <0.001 |
| Incidence | Dominican Republic | Both | 0.66 (0.62 to 0.70) | 29.688 | <0.001 |
| Incidence | Ecuador | Both | -0.82 (-0.87 to -0.76) | -30.392 | <0.001 |
| Incidence | Egypt | Both | 0.47 (0.44 to 0.49) | 39.763 | <0.001 |
| Incidence | El Salvador | Both | -0.90 (-0.99 to -0.82) | -21.752 | <0.001 |
| Incidence | Equatorial Guinea | Both | -0.82 (-0.86 to -0.79) | -42.522 | <0.001 |
| Incidence | Eritrea | Both | -0.53 (-0.63 to -0.43) | -10.195 | <0.001 |
| Incidence | Estonia | Both | -2.78 (-2.85 to -2.71) | -75.633 | <0.001 |
| Incidence | Eswatini | Both | 0.34 (0.21 to 0.47) | 5.186 | <0.001 |
| Incidence | Ethiopia | Both | -0.83 (-0.88 to -0.77) | -29.719 | <0.001 |
| Incidence | Fiji | Both | -0.49 (-0.55 to -0.43) | -17.132 | <0.001 |
| Incidence | Finland | Both | -1.89 (-1.93 to -1.84) | -77.023 | <0.001 |
| Incidence | France | Both | -0.81 (-0.84 to -0.78) | -54.874 | <0.001 |
| Incidence | Gabon | Both | -0.36 (-0.39 to -0.32) | -18.653 | <0.001 |
| Incidence | Gambia | Both | -0.16 (-0.19 to -0.14) | -11.296 | <0.001 |
| Incidence | Georgia | Both | 0.17 (0.13 to 0.20) | 8.846 | <0.001 |
| Incidence | Germany | Both | -1.60 (-1.63 to -1.56) | -96.305 | <0.001 |
| Incidence | Ghana | Both | 0.06 (0.00 to 0.13) | 1.934 | 0.053 |
| Incidence | Greece | Both | -1.80 (-2.04 to -1.56) | -14.582 | <0.001 |
| Incidence | Greenland | Both | -2.46 (-2.52 to -2.40) | -77.33 | <0.001 |
| Incidence | Grenada | Both | -0.66 (-0.72 to -0.61) | -23.259 | <0.001 |
| Incidence | Guam | Both | -0.64 (-0.70 to -0.59) | -23.411 | <0.001 |
| Incidence | Guatemala | Both | -0.73 (-0.79 to -0.67) | -24.196 | <0.001 |
| Incidence | Guinea | Both | 0.00 (-0.05 to 0.04) | -0.057 | 0.955 |
| Incidence | Guinea-Bissau | Both | -0.22 (-0.27 to -0.16) | -8.142 | <0.001 |
| Incidence | Guyana | Both | -1.05 (-1.16 to -0.93) | -17.886 | <0.001 |
| Incidence | Haiti | Both | -0.37 (-0.39 to -0.36) | -52.031 | <0.001 |
| Incidence | Honduras | Both | 0.17 (0.14 to 0.20) | 9.955 | <0.001 |
| Incidence | Hungary | Both | -2.20 (-2.24 to -2.16) | -107.113 | <0.001 |
| Incidence | Iceland | Both | -2.27 (-2.36 to -2.17) | -45.724 | <0.001 |
| Incidence | India | Both | -0.68 (-0.72 to -0.64) | -35.47 | <0.001 |
| Incidence | Indonesia | Both | 0.51 (0.48 to 0.55) | 30.473 | <0.001 |
| Incidence | Iran (Islamic Republic of) | Both | -1.14 (-1.24 to -1.05) | -23.171 | <0.001 |
| Incidence | Iraq | Both | 0.07 (0.03 to 0.12) | 2.989 | 0.003 |
| Incidence | Ireland | Both | -3.15 (-3.36 to -2.95) | -29.803 | <0.001 |
| Incidence | Israel | Both | -2.44 (-2.50 to -2.37) | -73.574 | <0.001 |
| Incidence | Italy | Both | -2.64 (-2.69 to -2.59) | -94.184 | <0.001 |
| Incidence | Jamaica | Both | -0.55 (-0.62 to -0.48) | -15.142 | <0.001 |
| Incidence | Japan | Both | -1.69 (-1.96 to -1.42) | -12.268 | <0.001 |
| Incidence | Jordan | Both | -0.64 (-0.72 to -0.56) | -15.553 | <0.001 |
| Incidence | Kazakhstan | Both | -0.73 (-0.81 to -0.65) | -17.107 | <0.001 |
| Incidence | Kenya | Both | -0.02 (-0.09 to 0.05) | -0.611 | 0.542 |
| Incidence | Kiribati | Both | -0.23 (-0.26 to -0.21) | -17.818 | <0.001 |
| Incidence | Kuwait | Both | -0.34 (-0.61 to -0.07) | -2.43 | 0.015 |
| Incidence | Kyrgyzstan | Both | -1.31 (-1.40 to -1.22) | -27.266 | <0.001 |
| Incidence | Lao People's Democratic Republic | Both | -0.26 (-0.29 to -0.23) | -16.47 | <0.001 |
| Incidence | Latvia | Both | -1.31 (-1.42 to -1.21) | -25.052 | <0.001 |
| Incidence | Lebanon | Both | -0.52 (-0.54 to -0.50) | -56.775 | <0.001 |
| Incidence | Lesotho | Both | 1.13 (1.08 to 1.19) | 43.008 | <0.001 |
| Incidence | Liberia | Both | -0.69 (-0.71 to -0.66) | -56.337 | <0.001 |
| Incidence | Libya | Both | 0.45 (0.42 to 0.48) | 28.542 | <0.001 |
| Incidence | Lithuania | Both | -1.18 (-1.38 to -0.97) | -11.296 | <0.001 |
| Incidence | Luxembourg | Both | -2.85 (-2.91 to -2.79) | -90.091 | <0.001 |
| Incidence | Madagascar | Both | -0.05 (-0.11 to 0.02) | -1.269 | 0.205 |
| Incidence | Malawi | Both | -0.17 (-0.20 to -0.15) | -13.958 | <0.001 |
| Incidence | Malaysia | Both | -0.72 (-0.73 to -0.70) | -101.56 | <0.001 |
| Incidence | Maldives | Both | -1.64 (-1.69 to -1.58) | -59.341 | <0.001 |
| Incidence | Mali | Both | -0.58 (-0.62 to -0.54) | -29.906 | <0.001 |
| Incidence | Malta | Both | -2.92 (-3.24 to -2.60) | -17.445 | <0.001 |
| Incidence | Marshall Islands | Both | -0.14 (-0.17 to -0.10) | -7.464 | <0.001 |
| Incidence | Mauritania | Both | -1.04 (-1.06 to -1.03) | -124.612 | <0.001 |
| Incidence | Mauritius | Both | -2.15 (-2.30 to -2.00) | -27.643 | <0.001 |
| Incidence | Mexico | Both | -1.49 (-1.56 to -1.42) | -40.562 | <0.001 |
| Incidence | Micronesia (Federated States of) | Both | -0.23 (-0.26 to -0.20) | -15.756 | <0.001 |
| Incidence | Monaco | Both | -1.93 (-2.01 to -1.85) | -46.191 | <0.001 |
| Incidence | Mongolia | Both | 0.43 (0.33 to 0.53) | 8.167 | <0.001 |
| Incidence | Montenegro | Both | 0.10 (0.01 to 0.19) | 2.205 | 0.027 |
| Incidence | Morocco | Both | 0.21 (0.20 to 0.23) | 22.215 | <0.001 |
| Incidence | Mozambique | Both | 0.39 (0.31 to 0.48) | 9.329 | <0.001 |
| Incidence | Myanmar | Both | -0.42 (-0.50 to -0.35) | -11.071 | <0.001 |
| Incidence | Namibia | Both | -0.31 (-0.36 to -0.26) | -11.249 | <0.001 |
| Incidence | Nauru | Both | -0.79 (-0.85 to -0.73) | -26.321 | <0.001 |
| Incidence | Nepal | Both | -0.30 (-0.34 to -0.26) | -13.143 | <0.001 |
| Incidence | Netherlands | Both | -2.20 (-2.29 to -2.11) | -47.788 | <0.001 |
| Incidence | New Zealand | Both | -1.69 (-1.72 to -1.66) | -116.542 | <0.001 |
| Incidence | Nicaragua | Both | -1.17 (-1.20 to -1.13) | -63.247 | <0.001 |
| Incidence | Niger | Both | -0.60 (-0.61 to -0.58) | -72.213 | <0.001 |
| Incidence | Nigeria | Both | -0.37 (-0.39 to -0.36) | -37.948 | <0.001 |
| Incidence | Niue | Both | -0.51 (-0.58 to -0.44) | -13.972 | <0.001 |
| Incidence | North Macedonia | Both | -0.11 (-0.21 to -0.01) | -2.1 | 0.036 |
| Incidence | Northern Mariana Islands | Both | -0.28 (-0.31 to -0.25) | -16.105 | <0.001 |
| Incidence | Norway | Both | -1.94 (-2.08 to -1.80) | -26.526 | <0.001 |
| Incidence | Oman | Both | -0.13 (-0.21 to -0.06) | -3.48 | 0.001 |
| Incidence | Pakistan | Both | -0.10 (-0.15 to -0.06) | -4.524 | <0.001 |
| Incidence | Palau | Both | -0.12 (-0.15 to -0.09) | -7.153 | <0.001 |
| Incidence | Palestine | Both | -0.09 (-0.28 to 0.09) | -0.991 | 0.322 |
| Incidence | Panama | Both | -1.08 (-1.10 to -1.06) | -85.943 | <0.001 |
| Incidence | Papua New Guinea | Both | -0.18 (-0.20 to -0.16) | -15.064 | <0.001 |
| Incidence | Paraguay | Both | -0.74 (-0.81 to -0.67) | -22.043 | <0.001 |
| Incidence | Peru | Both | -1.18 (-1.26 to -1.11) | -29.371 | <0.001 |
| Incidence | Philippines | Both | 0.79 (0.68 to 0.89) | 14.465 | <0.001 |
| Incidence | Poland | Both | -1.08 (-1.16 to -1.00) | -25.852 | <0.001 |
| Incidence | Portugal | Both | -3.88 (-4.08 to -3.68) | -37.433 | <0.001 |
| Incidence | Puerto Rico | Both | -1.17 (-1.23 to -1.11) | -39.459 | <0.001 |
| Incidence | Qatar | Both | -1.45 (-1.59 to -1.30) | -19.07 | <0.001 |
| Incidence | Republic of Korea | Both | -3.27 (-3.48 to -3.06) | -30.008 | <0.001 |
| Incidence | Republic of Moldova | Both | -0.54 (-0.67 to -0.40) | -7.882 | <0.001 |
| Incidence | Romania | Both | -1.12 (-1.18 to -1.07) | -39.389 | <0.001 |
| Incidence | Russian Federation | Both | -1.01 (-1.20 to -0.81) | -10.141 | <0.001 |
| Incidence | Rwanda | Both | -1.16 (-1.23 to -1.08) | -31.952 | <0.001 |
| Incidence | Saint Kitts and Nevis | Both | -1.37 (-1.41 to -1.33) | -66.068 | <0.001 |
| Incidence | Saint Lucia | Both | -1.56 (-1.65 to -1.47) | -33.628 | <0.001 |
| Incidence | Saint Vincent and the Grenadines | Both | -1.08 (-1.14 to -1.03) | -39.797 | <0.001 |
| Incidence | Samoa | Both | -0.29 (-0.35 to -0.23) | -9.099 | <0.001 |
| Incidence | San Marino | Both | -1.65 (-1.68 to -1.61) | -98.544 | <0.001 |
| Incidence | Sao Tome and Principe | Both | 0.10 (0.06 to 0.14) | 4.555 | <0.001 |
| Incidence | Saudi Arabia | Both | -0.49 (-0.57 to -0.40) | -11.327 | <0.001 |
| Incidence | Senegal | Both | -0.57 (-0.62 to -0.52) | -21.164 | <0.001 |
| Incidence | Serbia | Both | -0.74 (-0.79 to -0.70) | -32.792 | <0.001 |
| Incidence | Seychelles | Both | -0.71 (-0.74 to -0.67) | -37.549 | <0.001 |
| Incidence | Sierra Leone | Both | -0.40 (-0.42 to -0.37) | -31.478 | <0.001 |
| Incidence | Singapore | Both | -3.39 (-3.50 to -3.27) | -57.846 | <0.001 |
| Incidence | Slovakia | Both | -1.35 (-1.41 to -1.29) | -44.583 | <0.001 |
| Incidence | Slovenia | Both | -2.70 (-2.76 to -2.64) | -90.242 | <0.001 |
| Incidence | Solomon Islands | Both | 0.07 (0.04 to 0.09) | 5.715 | <0.001 |
| Incidence | Somalia | Both | -0.34 (-0.37 to -0.31) | -22.735 | <0.001 |
| Incidence | South Africa | Both | -0.11 (-0.30 to 0.09) | -1.076 | 0.282 |
| Incidence | South Sudan | Both | -0.35 (-0.36 to -0.33) | -41.093 | <0.001 |
| Incidence | Spain | Both | -2.27 (-2.47 to -2.07) | -21.968 | <0.001 |
| Incidence | Sri Lanka | Both | -0.64 (-0.67 to -0.61) | -41.757 | <0.001 |
| Incidence | Sudan | Both | 0.04 (0.00 to 0.07) | 2.063 | 0.039 |
| Incidence | Suriname | Both | -0.30 (-0.35 to -0.25) | -11.37 | <0.001 |
| Incidence | Sweden | Both | -1.50 (-1.59 to -1.42) | -35.077 | <0.001 |
| Incidence | Switzerland | Both | -1.76 (-1.79 to -1.72) | -95.151 | <0.001 |
| Incidence | Syrian Arab Republic | Both | -0.63 (-0.66 to -0.59) | -36.682 | <0.001 |
| Incidence | Taiwan (Province of China) | Both | -1.57 (-1.64 to -1.49) | -40.511 | <0.001 |
| Incidence | Tajikistan | Both | 0.61 (0.53 to 0.68) | 16.294 | <0.001 |
| Incidence | Thailand | Both | -1.03 (-1.09 to -0.96) | -31.629 | <0.001 |
| Incidence | Timor-Leste | Both | 0.43 (0.34 to 0.52) | 9.188 | <0.001 |
| Incidence | Togo | Both | -0.42 (-0.47 to -0.37) | -16.436 | <0.001 |
| Incidence | Tokelau | Both | -0.57 (-0.63 to -0.51) | -19.355 | <0.001 |
| Incidence | Tonga | Both | -0.09 (-0.12 to -0.05) | -5.029 | <0.001 |
| Incidence | Trinidad and Tobago | Both | -1.46 (-1.49 to -1.43) | -92.718 | <0.001 |
| Incidence | Tunisia | Both | -0.01 (-0.07 to 0.06) | -0.153 | 0.879 |
| Incidence | T rkiye | Both | -1.29 (-1.34 to -1.24) | -50.985 | <0.001 |
| Incidence | Turkmenistan | Both | 0.41 (0.19 to 0.63) | 3.697 | <0.001 |
| Incidence | Tuvalu | Both | -0.11 (-0.14 to -0.09) | -8.191 | <0.001 |
| Incidence | Uganda | Both | -0.21 (-0.30 to -0.13) | -4.987 | <0.001 |
| Incidence | Ukraine | Both | -1.21 (-1.25 to -1.18) | -72.294 | <0.001 |
| Incidence | United Arab Emirates | Both | -0.87 (-1.03 to -0.72) | -11.255 | <0.001 |
| Incidence | United Kingdom | Both | -2.03 (-2.09 to -1.97) | -67.44 | <0.001 |
| Incidence | United Republic of Tanzania | Both | 0.50 (0.42 to 0.57) | 13.07 | <0.001 |
| Incidence | United States of America | Both | -1.45 (-1.50 to -1.41) | -61.243 | <0.001 |
| Incidence | United States Virgin Islands | Both | -0.07 (-0.08 to -0.05) | -8.396 | <0.001 |
| Incidence | Uruguay | Both | -2.05 (-2.17 to -1.94) | -35.611 | <0.001 |
| Incidence | Uzbekistan | Both | 0.35 (0.27 to 0.43) | 8.136 | <0.001 |
| Incidence | Vanuatu | Both | 0.01 (-0.07 to 0.08) | 0.179 | 0.858 |
| Incidence | Venezuela (Bolivarian Republic of) | Both | -0.84 (-0.90 to -0.78) | -26.7 | <0.001 |
| Incidence | Viet Nam | Both | 0.17 (0.03 to 0.30) | 2.392 | 0.017 |
| Incidence | Yemen | Both | 0.00 (-0.02 to 0.02) | -0.023 | 0.981 |
| Incidence | Zambia | Both | 0.41 (0.38 to 0.44) | 26.646 | <0.001 |
| Incidence | Zimbabwe | Both | 0.54 (0.42 to 0.66) | 8.935 | <0.001 |
| Deaths | Global | Both | -1.60 (-1.81 to -1.39) | -14.994 | <0.001 |
| Deaths | Global | Female | -1.88 (-2.10 to -1.66) | -16.429 | <0.001 |
| Deaths | Global | Male | -1.28 (-1.47 to -1.08) | -12.89 | <0.001 |
| Deaths | Central Europe Eastern Europe and Central Asia | Both | -2.00 (-2.33 to -1.67) | -11.695 | <0.001 |
| Deaths | High-income | Both | -3.61 (-3.89 to -3.33) | -24.74 | <0.001 |
| Deaths | Latin America and Caribbean | Both | -2.53 (-2.87 to -2.18) | -14.13 | <0.001 |
| Deaths | North Africa and Middle East | Both | -1.16 (-1.34 to -0.98) | -12.629 | <0.001 |
| Deaths | South Asia | Both | -0.38 (-0.89 to 0.12) | -1.484 | 0.138 |
| Deaths | Southeast Asia East Asia and Oceania | Both | -0.44 (-0.63 to -0.26) | -4.639 | <0.001 |
| Deaths | Sub-Saharan Africa | Both | -0.23 (-0.35 to -0.11) | -3.711 | <0.001 |
| Deaths | Andean Latin America | Both | -1.85 (-2.45 to -1.24) | -5.919 | <0.001 |
| Deaths | Australasia | Both | -3.78 (-4.01 to -3.56) | -32.828 | <0.001 |
| Deaths | Caribbean | Both | -1.16 (-1.71 to -0.60) | -4.071 | <0.001 |
| Deaths | Central Asia | Both | -0.32 (-1.02 to 0.39) | -0.887 | 0.375 |
| Deaths | Central Europe | Both | -2.46 (-2.69 to -2.23) | -20.861 | <0.001 |
| Deaths | Central Latin America | Both | -2.25 (-2.56 to -1.94) | -13.943 | <0.001 |
| Deaths | Central Sub-Saharan Africa | Both | -0.28 (-0.43 to -0.12) | -3.426 | 0.001 |
| Deaths | East Asia | Both | -0.52 (-0.78 to -0.26) | -3.897 | <0.001 |
| Deaths | Eastern Europe | Both | -1.96 (-2.50 to -1.41) | -6.94 | <0.001 |
| Deaths | Eastern Sub-Saharan Africa | Both | -0.36 (-0.42 to -0.30) | -11.779 | <0.001 |
| Deaths | High-income Asia Pacific | Both | -4.41 (-4.84 to -3.97) | -19.443 | <0.001 |
| Deaths | High-income North America | Both | -1.77 (-2.09 to -1.45) | -10.743 | <0.001 |
| Deaths | North Africa and Middle East | Both | -1.16 (-1.34 to -0.98) | -12.627 | <0.001 |
| Deaths | Oceania | Both | -0.58 (-0.64 to -0.53) | -21.331 | <0.001 |
| Deaths | South Asia | Both | -0.39 (-0.89 to 0.12) | -1.486 | 0.137 |
| Deaths | Southeast Asia | Both | -0.21 (-0.28 to -0.13) | -5.408 | <0.001 |
| Deaths | Southern Latin America | Both | -3.19 (-3.53 to -2.86) | -18.234 | <0.001 |
| Deaths | Southern Sub-Saharan Africa | Both | 0.82 (0.25 to 1.39) | 2.813 | 0.005 |
| Deaths | Tropical Latin America | Both | -3.12 (-3.31 to -2.93) | -31.666 | <0.001 |
| Deaths | Western Europe | Both | -4.19 (-4.35 to -4.02) | -48.778 | <0.001 |
| Deaths | Western Sub-Saharan Africa | Both | -0.36 (-0.44 to -0.28) | -9.234 | <0.001 |
| Deaths | Afghanistan | Both | -0.27 (-0.33 to -0.21) | -9.043 | <0.001 |
| Deaths | Albania | Both | -0.70 (-1.36 to -0.04) | -2.083 | 0.037 |
| Deaths | Algeria | Both | -1.09 (-1.25 to -0.94) | -13.857 | <0.001 |
| Deaths | American Samoa | Both | -0.87 (-1.01 to -0.74) | -12.542 | <0.001 |
| Deaths | Andorra | Both | -2.47 (-2.90 to -2.03) | -10.969 | <0.001 |
| Deaths | Angola | Both | -0.09 (-0.17 to -0.01) | -2.103 | 0.035 |
| Deaths | Antigua and Barbuda | Both | -1.30 (-1.90 to -0.69) | -4.163 | <0.001 |
| Deaths | Argentina | Both | -3.32 (-3.93 to -2.69) | -10.29 | <0.001 |
| Deaths | Armenia | Both | -1.00 (-2.13 to 0.14) | -1.72 | 0.085 |
| Deaths | Australia | Both | -3.97 (-4.24 to -3.69) | -27.82 | <0.001 |
| Deaths | Austria | Both | -5.05 (-5.74 to -4.35) | -13.802 | <0.001 |
| Deaths | Azerbaijan | Both | -0.44 (-1.05 to 0.18) | -1.406 | 0.16 |
| Deaths | Bahamas | Both | -1.11 (-1.88 to -0.32) | -2.767 | 0.006 |
| Deaths | Bahrain | Both | -1.75 (-2.48 to -1.01) | -4.601 | <0.001 |
| Deaths | Bangladesh | Both | -0.07 (-0.88 to 0.74) | -0.172 | 0.863 |
| Deaths | Barbados | Both | -1.54 (-2.34 to -0.73) | -3.698 | <0.001 |
| Deaths | Belarus | Both | -1.09 (-1.62 to -0.55) | -3.987 | <0.001 |
| Deaths | Belgium | Both | -4.08 (-4.25 to -3.92) | -47.547 | <0.001 |
| Deaths | Belize | Both | -0.18 (-0.99 to 0.63) | -0.447 | 0.655 |
| Deaths | Benin | Both | -0.40 (-0.57 to -0.23) | -4.688 | <0.001 |
| Deaths | Bermuda | Both | -3.00 (-3.58 to -2.42) | -10.038 | <0.001 |
| Deaths | Bhutan | Both | -0.52 (-0.63 to -0.42) | -9.748 | <0.001 |
| Deaths | Bolivia (Plurinational State of) | Both | -1.55 (-1.66 to -1.44) | -26.566 | <0.001 |
| Deaths | Bosnia and Herzegovina | Both | -0.97 (-1.23 to -0.70) | -7.118 | <0.001 |
| Deaths | Botswana | Both | -1.29 (-2.35 to -0.22) | -2.365 | 0.018 |
| Deaths | Brazil | Both | -3.17 (-3.36 to -2.98) | -31.714 | <0.001 |
| Deaths | Brunei Darussalam | Both | -2.33 (-2.75 to -1.90) | -10.646 | <0.001 |
| Deaths | Bulgaria | Both | -0.67 (-1.46 to 0.12) | -1.671 | 0.095 |
| Deaths | Burkina Faso | Both | 0.05 (-0.08 to 0.19) | 0.772 | 0.44 |
| Deaths | Burundi | Both | -1.54 (-1.73 to -1.36) | -16.088 | <0.001 |
| Deaths | Cabo Verde | Both | 0.96 (0.57 to 1.36) | 4.762 | <0.001 |
| Deaths | Cambodia | Both | -0.05 (-0.11 to 0.02) | -1.335 | 0.182 |
| Deaths | Cameroon | Both | 0.32 (0.20 to 0.45) | 5.202 | <0.001 |
| Deaths | Canada | Both | -3.14 (-3.51 to -2.77) | -16.233 | <0.001 |
| Deaths | Central African Republic | Both | -0.29 (-0.38 to -0.20) | -6.314 | <0.001 |
| Deaths | Chad | Both | 0.46 (0.32 to 0.60) | 6.375 | <0.001 |
| Deaths | Chile | Both | -3.05 (-3.62 to -2.48) | -10.36 | <0.001 |
| Deaths | China | Both | -0.49 (-0.75 to -0.23) | -3.649 | <0.001 |
| Deaths | Colombia | Both | -3.19 (-3.73 to -2.65) | -11.422 | <0.001 |
| Deaths | Comoros | Both | -1.04 (-1.11 to -0.98) | -31.229 | <0.001 |
| Deaths | Congo | Both | -0.58 (-0.75 to -0.42) | -6.835 | <0.001 |
| Deaths | Cook Islands | Both | -1.97 (-2.04 to -1.90) | -53.898 | <0.001 |
| Deaths | Costa Rica | Both | -1.44 (-2.16 to -0.72) | -3.912 | <0.001 |
| Deaths | Croatia | Both | -3.47 (-3.69 to -3.24) | -29.934 | <0.001 |
| Deaths | Cuba | Both | -0.81 (-0.95 to -0.68) | -12.304 | <0.001 |
| Deaths | Cyprus | Both | -4.12 (-4.93 to -3.31) | -9.785 | <0.001 |
| Deaths | Czechia | Both | -5.20 (-5.77 to -4.63) | -17.394 | <0.001 |
| Deaths | Côte d'Ivoire | Both | -0.22 (-0.33 to -0.11) | -3.919 | <0.001 |
| Deaths | Democratic People's Republic of Korea | Both | -0.24 (-0.27 to -0.20) | -12.397 | <0.001 |
| Deaths | Democratic Republic of the Congo | Both | -0.28 (-0.51 to -0.05) | -2.374 | 0.018 |
| Deaths | Denmark | Both | -3.11 (-3.43 to -2.79) | -18.823 | <0.001 |
| Deaths | Djibouti | Both | -0.11 (-0.23 to 0.01) | -1.849 | 0.065 |
| Deaths | Dominica | Both | -0.66 (-0.73 to -0.58) | -16.586 | <0.001 |
| Deaths | Dominican Republic | Both | -0.53 (-1.05 to -0.02) | -2.02 | 0.043 |
| Deaths | Ecuador | Both | -2.07 (-2.92 to -1.21) | -4.674 | <0.001 |
| Deaths | Egypt | Both | -0.87 (-1.36 to -0.38) | -3.459 | 0.001 |
| Deaths | El Salvador | Both | -1.48 (-2.29 to -0.66) | -3.517 | <0.001 |
| Deaths | Equatorial Guinea | Both | -0.64 (-0.83 to -0.45) | -6.702 | <0.001 |
| Deaths | Eritrea | Both | -0.36 (-0.44 to -0.27) | -8.044 | <0.001 |
| Deaths | Estonia | Both | -5.18 (-6.09 to -4.27) | -10.921 | <0.001 |
| Deaths | Eswatini | Both | 0.08 (-0.09 to 0.25) | 0.9 | 0.368 |
| Deaths | Ethiopia | Both | -0.77 (-0.88 to -0.66) | -13.133 | <0.001 |
| Deaths | Fiji | Both | -0.17 (-0.41 to 0.08) | -1.31 | 0.19 |
| Deaths | Finland | Both | -3.65 (-4.04 to -3.26) | -17.985 | <0.001 |
| Deaths | France | Both | -3.82 (-4.14 to -3.49) | -22.423 | <0.001 |
| Deaths | Gabon | Both | -0.24 (-0.44 to -0.03) | -2.271 | 0.023 |
| Deaths | Gambia | Both | 0.48 (0.16 to 0.80) | 2.931 | 0.003 |
| Deaths | Georgia | Both | 0.81 (-0.85 to 2.51) | 0.953 | 0.34 |
| Deaths | Germany | Both | -4.29 (-4.54 to -4.03) | -32.298 | <0.001 |
| Deaths | Ghana | Both | 0.16 (0.09 to 0.23) | 4.642 | <0.001 |
| Deaths | Greece | Both | -4.12 (-4.42 to -3.82) | -26.017 | <0.001 |
| Deaths | Greenland | Both | -3.22 (-3.47 to -2.97) | -25.007 | <0.001 |
| Deaths | Grenada | Both | -2.05 (-2.57 to -1.52) | -7.514 | <0.001 |
| Deaths | Guam | Both | -4.42 (-5.61 to -3.23) | -7.113 | <0.001 |
| Deaths | Guatemala | Both | -1.48 (-2.48 to -0.48) | -2.877 | 0.004 |
| Deaths | Guinea | Both | 0.41 (0.33 to 0.48) | 11.073 | <0.001 |
| Deaths | Guinea-Bissau | Both | -0.03 (-0.10 to 0.04) | -0.776 | 0.438 |
| Deaths | Guyana | Both | -1.38 (-2.06 to -0.70) | -3.968 | <0.001 |
| Deaths | Haiti | Both | -0.86 (-0.90 to -0.82) | -37.897 | <0.001 |
| Deaths | Honduras | Both | 0.99 (0.64 to 1.33) | 5.626 | <0.001 |
| Deaths | Hungary | Both | -3.52 (-4.08 to -2.95) | -11.989 | <0.001 |
| Deaths | Iceland | Both | -3.40 (-4.10 to -2.69) | -9.324 | <0.001 |
| Deaths | India | Both | -0.46 (-1.06 to 0.15) | -1.47 | 0.142 |
| Deaths | Indonesia | Both | 1.02 (0.97 to 1.07) | 39.392 | <0.001 |
| Deaths | Iran (Islamic Republic of) | Both | -2.04 (-2.16 to -1.92) | -32.393 | <0.001 |
| Deaths | Iraq | Both | 0.04 (-0.20 to 0.28) | 0.306 | 0.76 |
| Deaths | Ireland | Both | -4.64 (-5.05 to -4.23) | -21.893 | <0.001 |
| Deaths | Israel | Both | -4.00 (-4.84 to -3.16) | -9.105 | <0.001 |
| Deaths | Italy | Both | -3.89 (-4.29 to -3.49) | -18.715 | <0.001 |
| Deaths | Jamaica | Both | -0.90 (-1.53 to -0.26) | -2.758 | 0.006 |
| Deaths | Japan | Both | -4.41 (-4.83 to -3.99) | -20.191 | <0.001 |
| Deaths | Jordan | Both | -2.41 (-3.07 to -1.74) | -7.029 | <0.001 |
| Deaths | Kazakhstan | Both | -0.44 (-1.33 to 0.45) | -0.968 | 0.333 |
| Deaths | Kenya | Both | 0.41 (0.32 to 0.50) | 9.107 | <0.001 |
| Deaths | Kiribati | Both | 0.21 (0.17 to 0.24) | 11.436 | <0.001 |
| Deaths | Kuwait | Both | -0.90 (-3.11 to 1.36) | -0.786 | 0.432 |
| Deaths | Kyrgyzstan | Both | -2.09 (-2.54 to -1.64) | -8.99 | <0.001 |
| Deaths | Lao People's Democratic Republic | Both | -1.02 (-1.08 to -0.97) | -36.232 | <0.001 |
| Deaths | Latvia | Both | -1.41 (-2.38 to -0.43) | -2.808 | 0.005 |
| Deaths | Lebanon | Both | -3.11 (-3.32 to -2.90) | -29.029 | <0.001 |
| Deaths | Lesotho | Both | 1.52 (1.05 to 2.00) | 6.327 | <0.001 |
| Deaths | Liberia | Both | -0.13 (-0.30 to 0.04) | -1.515 | 0.13 |
| Deaths | Libya | Both | 0.36 (-0.12 to 0.84) | 1.465 | 0.143 |
| Deaths | Lithuania | Both | -1.07 (-1.53 to -0.61) | -4.525 | <0.001 |
| Deaths | Luxembourg | Both | -5.59 (-6.29 to -4.88) | -15.162 | <0.001 |
| Deaths | Madagascar | Both | -0.22 (-0.36 to -0.08) | -3.104 | 0.002 |
| Deaths | Malawi | Both | 0.40 (0.22 to 0.59) | 4.21 | <0.001 |
| Deaths | Malaysia | Both | -0.64 (-1.07 to -0.21) | -2.925 | 0.003 |
| Deaths | Maldives | Both | -2.33 (-2.49 to -2.16) | -27.432 | <0.001 |
| Deaths | Mali | Both | -0.17 (-0.37 to 0.03) | -1.655 | 0.098 |
| Deaths | Malta | Both | -4.81 (-5.20 to -4.42) | -23.797 | <0.001 |
| Deaths | Marshall Islands | Both | -0.49 (-0.63 to -0.36) | -7.2 | <0.001 |
| Deaths | Mauritania | Both | -0.66 (-0.82 to -0.50) | -8.077 | <0.001 |
| Deaths | Mauritius | Both | -3.26 (-4.14 to -2.37) | -7.113 | <0.001 |
| Deaths | Mexico | Both | -2.78 (-3.36 to -2.20) | -9.342 | <0.001 |
| Deaths | Micronesia (Federated States of) | Both | -0.58 (-0.62 to -0.55) | -34.255 | <0.001 |
| Deaths | Monaco | Both | -2.93 (-3.05 to -2.80) | -44.655 | <0.001 |
| Deaths | Mongolia | Both | -0.12 (-0.61 to 0.38) | -0.464 | 0.643 |
| Deaths | Montenegro | Both | 1.78 (0.97 to 2.59) | 4.337 | <0.001 |
| Deaths | Morocco | Both | -0.11 (-0.22 to 0.00) | -1.924 | 0.054 |
| Deaths | Mozambique | Both | 0.69 (0.59 to 0.79) | 13.718 | <0.001 |
| Deaths | Myanmar | Both | -0.99 (-1.05 to -0.92) | -30.74 | <0.001 |
| Deaths | Namibia | Both | -0.26 (-0.50 to -0.02) | -2.133 | 0.033 |
| Deaths | Nauru | Both | -0.37 (-0.43 to -0.31) | -12.252 | <0.001 |
| Deaths | Nepal | Both | -0.84 (-0.93 to -0.76) | -19.92 | <0.001 |
| Deaths | Netherlands | Both | -2.73 (-3.15 to -2.32) | -12.625 | <0.001 |
| Deaths | New Zealand | Both | -2.84 (-3.10 to -2.58) | -21.389 | <0.001 |
| Deaths | Nicaragua | Both | -1.81 (-2.26 to -1.36) | -7.863 | <0.001 |
| Deaths | Niger | Both | 0.03 (-0.07 to 0.13) | 0.561 | 0.575 |
| Deaths | Nigeria | Both | -0.84 (-0.97 to -0.70) | -12.003 | <0.001 |
| Deaths | Niue | Both | -0.64 (-0.72 to -0.55) | -14.211 | <0.001 |
| Deaths | North Macedonia | Both | 0.47 (-0.15 to 1.09) | 1.493 | 0.136 |
| Deaths | Northern Mariana Islands | Both | -1.36 (-2.06 to -0.65) | -3.748 | <0.001 |
| Deaths | Norway | Both | -4.28 (-4.65 to -3.91) | -22.174 | <0.001 |
| Deaths | Oman | Both | -1.12 (-2.07 to -0.17) | -2.298 | 0.022 |
| Deaths | Pakistan | Both | 0.10 (0.03 to 0.17) | 2.844 | 0.004 |
| Deaths | Palau | Both | -0.53 (-0.66 to -0.40) | -8.148 | <0.001 |
| Deaths | Palestine | Both | -1.55 (-1.70 to -1.41) | -20.408 | <0.001 |
| Deaths | Panama | Both | -1.44 (-2.17 to -0.70) | -3.823 | <0.001 |
| Deaths | Papua New Guinea | Both | -0.37 (-0.41 to -0.33) | -18.043 | <0.001 |
| Deaths | Paraguay | Both | -1.19 (-1.79 to -0.59) | -3.895 | <0.001 |
| Deaths | Peru | Both | -1.69 (-3.08 to -0.28) | -2.347 | 0.019 |
| Deaths | Philippines | Both | -0.68 (-1.10 to -0.26) | -3.166 | 0.002 |
| Deaths | Poland | Both | -3.50 (-4.05 to -2.94) | -12.141 | <0.001 |
| Deaths | Portugal | Both | -5.64 (-6.18 to -5.09) | -19.803 | <0.001 |
| Deaths | Puerto Rico | Both | -3.91 (-4.80 to -3.02) | -8.425 | <0.001 |
| Deaths | Qatar | Both | -2.91 (-4.25 to -1.56) | -4.177 | <0.001 |
| Deaths | Republic of Korea | Both | -4.94 (-5.38 to -4.49) | -21.295 | <0.001 |
| Deaths | Republic of Moldova | Both | -1.95 (-2.66 to -1.24) | -5.309 | <0.001 |
| Deaths | Romania | Both | -2.03 (-2.48 to -1.57) | -8.692 | <0.001 |
| Deaths | Russian Federation | Both | -1.94 (-2.68 to -1.20) | -5.109 | <0.001 |
| Deaths | Rwanda | Both | -1.86 (-2.01 to -1.71) | -24.109 | <0.001 |
| Deaths | Saint Kitts and Nevis | Both | -1.92 (-2.84 to -1.00) | -4.049 | <0.001 |
| Deaths | Saint Lucia | Both | -2.80 (-3.70 to -1.89) | -5.957 | <0.001 |
| Deaths | Saint Vincent and the Grenadines | Both | -1.36 (-1.95 to -0.76) | -4.459 | <0.001 |
| Deaths | Samoa | Both | -0.61 (-0.66 to -0.55) | -22.885 | <0.001 |
| Deaths | San Marino | Both | -4.13 (-4.90 to -3.35) | -10.189 | <0.001 |
| Deaths | Sao Tome and Principe | Both | 0.42 (0.16 to 0.69) | 3.159 | 0.002 |
| Deaths | Saudi Arabia | Both | -1.24 (-1.37 to -1.12) | -19.35 | <0.001 |
| Deaths | Senegal | Both | -0.17 (-0.38 to 0.05) | -1.518 | 0.129 |
| Deaths | Serbia | Both | -1.97 (-2.21 to -1.74) | -16.308 | <0.001 |
| Deaths | Seychelles | Both | -1.15 (-1.72 to -0.59) | -3.974 | <0.001 |
| Deaths | Sierra Leone | Both | -0.25 (-0.43 to -0.07) | -2.74 | 0.006 |
| Deaths | Singapore | Both | -6.58 (-7.70 to -5.44) | -11.035 | <0.001 |
| Deaths | Slovakia | Both | -2.25 (-2.69 to -1.81) | -9.953 | <0.001 |
| Deaths | Slovenia | Both | -3.97 (-4.79 to -3.15) | -9.328 | <0.001 |
| Deaths | Solomon Islands | Both | -0.18 (-0.28 to -0.09) | -3.675 | <0.001 |
| Deaths | Somalia | Both | -0.65 (-0.76 to -0.54) | -11.743 | <0.001 |
| Deaths | South Africa | Both | 0.88 (0.18 to 1.59) | 2.45 | 0.014 |
| Deaths | South Sudan | Both | -0.54 (-0.65 to -0.44) | -9.933 | <0.001 |
| Deaths | Spain | Both | -5.04 (-5.22 to -4.86) | -54.19 | <0.001 |
| Deaths | Sri Lanka | Both | -1.24 (-1.83 to -0.64) | -4.035 | <0.001 |
| Deaths | Sudan | Both | -0.80 (-0.86 to -0.74) | -25.808 | <0.001 |
| Deaths | Suriname | Both | -0.53 (-1.13 to 0.07) | -1.742 | 0.081 |
| Deaths | Sweden | Both | -3.58 (-4.11 to -3.05) | -13.076 | <0.001 |
| Deaths | Switzerland | Both | -4.23 (-4.37 to -4.09) | -57.981 | <0.001 |
| Deaths | Syrian Arab Republic | Both | -0.65 (-0.93 to -0.36) | -4.473 | <0.001 |
| Deaths | Taiwan (Province of China) | Both | -4.31 (-5.17 to -3.45) | -9.62 | <0.001 |
| Deaths | Tajikistan | Both | -0.49 (-0.93 to -0.05) | -2.203 | 0.028 |
| Deaths | Thailand | Both | -1.67 (-2.13 to -1.21) | -7.016 | <0.001 |
| Deaths | Timor-Leste | Both | 0.33 (0.22 to 0.44) | 5.865 | <0.001 |
| Deaths | Togo | Both | -0.02 (-0.13 to 0.08) | -0.411 | 0.681 |
| Deaths | Tokelau | Both | -1.20 (-1.23 to -1.16) | -62.861 | <0.001 |
| Deaths | Tonga | Both | -0.14 (-0.37 to 0.09) | -1.205 | 0.228 |
| Deaths | Trinidad and Tobago | Both | -2.21 (-2.50 to -1.92) | -14.571 | <0.001 |
| Deaths | Tunisia | Both | -1.03 (-1.34 to -0.72) | -6.413 | <0.001 |
| Deaths | Turkmenistan | Both | 0.72 (-0.14 to 1.59) | 1.643 | 0.1 |
| Deaths | Tuvalu | Both | -0.77 (-0.81 to -0.74) | -45.021 | <0.001 |
| Deaths | Türkiye | Both | -1.71 (-2.11 to -1.31) | -8.276 | <0.001 |
| Deaths | Uganda | Both | -0.88 (-0.99 to -0.77) | -16.141 | <0.001 |
| Deaths | Ukraine | Both | -2.07 (-2.73 to -1.40) | -6.054 | <0.001 |
| Deaths | United Arab Emirates | Both | -1.13 (-3.98 to 1.81) | -0.757 | 0.449 |
| Deaths | United Kingdom | Both | -4.40 (-4.87 to -3.93) | -18.066 | <0.001 |
| Deaths | United Republic of Tanzania | Both | 0.76 (0.61 to 0.91) | 9.852 | <0.001 |
| Deaths | United States of America | Both | -1.58 (-1.80 to -1.35) | -13.88 | <0.001 |
| Deaths | United States Virgin Islands | Both | -2.65 (-2.97 to -2.34) | -16.305 | <0.001 |
| Deaths | Uruguay | Both | -2.74 (-3.10 to -2.37) | -14.444 | <0.001 |
| Deaths | Uzbekistan | Both | 0.04 (-0.78 to 0.87) | 0.106 | 0.916 |
| Deaths | Vanuatu | Both | -0.67 (-0.76 to -0.58) | -14.42 | <0.001 |
| Deaths | Venezuela (Bolivarian Republic of) | Both | -0.52 (-0.87 to -0.16) | -2.863 | 0.004 |
| Deaths | Viet Nam | Both | 0.22 (0.15 to 0.29) | 6.292 | <0.001 |
| Deaths | Yemen | Both | -0.19 (-0.28 to -0.10) | -4.113 | <0.001 |
| Deaths | Zambia | Both | 0.52 (0.37 to 0.66) | 7.074 | <0.001 |
| Deaths | Zimbabwe | Both | 1.19 (0.76 to 1.63) | 5.424 | <0.001 |
| DALYs | Global | Both | -1.37 (-1.53 to -1.20) | -16.284 | <0.001 |
| DALYs | Global | Female | -1.63 (-1.82 to -1.44) | -16.398 | <0.001 |
| DALYs | Global | Male | -1.12 (-1.27 to -0.96) | -14.318 | <0.001 |
| DALYs | Central Europe Eastern Europe and Central Asia | Both | -1.87 (-2.23 to -1.51) | -10.072 | <0.001 |
| DALYs | High-income | Both | -3.06 (-3.15 to -2.96) | -61.595 | <0.001 |
| DALYs | Latin America and Caribbean | Both | -2.44 (-2.73 to -2.16) | -16.828 | <0.001 |
| DALYs | North Africa and Middle East | Both | -1.18 (-1.38 to -0.99) | -11.695 | <0.001 |
| DALYs | South Asia | Both | -0.45 (-0.70 to -0.20) | -3.509 | <0.001 |
| DALYs | Southeast Asia East Asia and Oceania | Both | -0.47 (-0.61 to -0.34) | -6.802 | <0.001 |
| DALYs | Sub-Saharan Africa | Both | -0.29 (-0.38 to -0.19) | -6.015 | <0.001 |
| DALYs | Andean Latin America | Both | -1.85 (-2.37 to -1.33) | -6.915 | <0.001 |
| DALYs | Australasia | Both | -3.35 (-3.58 to -3.12) | -27.68 | <0.001 |
| DALYs | Caribbean | Both | -0.98 (-1.38 to -0.59) | -4.84 | <0.001 |
| DALYs | Central Asia | Both | -0.48 (-0.76 to -0.21) | -3.463 | 0.001 |
| DALYs | Central Europe | Both | -2.39 (-2.56 to -2.23) | -28.089 | <0.001 |
| DALYs | Central Latin America | Both | -2.08 (-2.39 to -1.77) | -13.06 | <0.001 |
| DALYs | Central Sub-Saharan Africa | Both | -0.39 (-0.45 to -0.33) | -12.636 | <0.001 |
| DALYs | East Asia | Both | -0.55 (-0.73 to -0.38) | -6.107 | <0.001 |
| DALYs | Eastern Europe | Both | -1.79 (-2.30 to -1.27) | -6.753 | <0.001 |
| DALYs | Eastern Sub-Saharan Africa | Both | -0.38 (-0.45 to -0.31) | -10.087 | <0.001 |
| DALYs | Global | Both | -1.37 (-1.53 to -1.20) | -16.284 | <0.001 |
| DALYs | High-income Asia Pacific | Both | -3.64 (-3.91 to -3.38) | -26.168 | <0.001 |
| DALYs | High-income North America | Both | -1.47 (-1.76 to -1.18) | -9.976 | <0.001 |
| DALYs | North Africa and Middle East | Both | -1.18 (-1.38 to -0.99) | -11.695 | <0.001 |
| DALYs | Oceania | Both | -0.56 (-0.61 to -0.50) | -19.839 | <0.001 |
| DALYs | South Asia | Both | -0.45 (-0.70 to -0.20) | -3.51 | <0.001 |
| DALYs | Southeast Asia | Both | -0.22 (-0.29 to -0.15) | -5.9 | <0.001 |
| DALYs | Southern Latin America | Both | -2.93 (-3.18 to -2.67) | -22.007 | <0.001 |
| DALYs | Southern Sub-Saharan Africa | Both | 0.51 (0.02 to 1.00) | 2.059 | 0.04 |
| DALYs | Tropical Latin America | Both | -3.00 (-3.32 to -2.67) | -17.562 | <0.001 |
| DALYs | Western Europe | Both | -3.72 (-3.85 to -3.60) | -56.585 | <0.001 |
| DALYs | Western Sub-Saharan Africa | Both | -0.40 (-0.45 to -0.36) | -17.652 | <0.001 |
| DALYs | Afghanistan | Both | -0.30 (-0.37 to -0.22) | -7.256 | <0.001 |
| DALYs | Albania | Both | -0.95 (-1.47 to -0.42) | -3.532 | <0.001 |
| DALYs | Algeria | Both | -1.17 (-1.27 to -1.07) | -21.809 | <0.001 |
| DALYs | American Samoa | Both | -0.84 (-0.96 to -0.72) | -13.809 | <0.001 |
| DALYs | Andorra | Both | -2.27 (-2.75 to -1.79) | -9.131 | <0.001 |
| DALYs | Angola | Both | -0.28 (-0.35 to -0.20) | -7.085 | <0.001 |
| DALYs | Antigua and Barbuda | Both | -1.43 (-2.12 to -0.73) | -4.003 | <0.001 |
| DALYs | Argentina | Both | -2.91 (-3.38 to -2.43) | -11.852 | <0.001 |
| DALYs | Armenia | Both | -1.02 (-1.93 to -0.11) | -2.19 | 0.028 |
| DALYs | Australia | Both | -3.41 (-3.72 to -3.09) | -20.712 | <0.001 |
| DALYs | Austria | Both | -3.96 (-4.36 to -3.55) | -18.865 | <0.001 |
| DALYs | Azerbaijan | Both | -0.57 (-0.97 to -0.17) | -2.768 | 0.006 |
| DALYs | Bahamas | Both | -1.26 (-1.77 to -0.74) | -4.778 | <0.001 |
| DALYs | Bahrain | Both | -1.95 (-2.54 to -1.37) | -6.488 | <0.001 |
| DALYs | Bangladesh | Both | -0.33 (-0.95 to 0.30) | -1.028 | 0.304 |
| DALYs | Barbados | Both | -1.52 (-2.24 to -0.80) | -4.101 | <0.001 |
| DALYs | Belarus | Both | -1.22 (-1.70 to -0.74) | -4.92 | <0.001 |
| DALYs | Belgium | Both | -3.58 (-3.69 to -3.48) | -65.95 | <0.001 |
| DALYs | Belize | Both | -0.29 (-1.04 to 0.46) | -0.768 | 0.442 |
| DALYs | Benin | Both | -0.47 (-0.65 to -0.28) | -4.907 | <0.001 |
| DALYs | Bermuda | Both | -2.90 (-3.29 to -2.52) | -14.59 | <0.001 |
| DALYs | Bhutan | Both | -0.67 (-0.74 to -0.60) | -17.601 | <0.001 |
| DALYs | Bolivia (Plurinational State of) | Both | -1.74 (-1.83 to -1.65) | -38.157 | <0.001 |
| DALYs | Bosnia and Herzegovina | Both | -1.09 (-1.41 to -0.76) | -6.444 | <0.001 |
| DALYs | Botswana | Both | -1.15 (-1.90 to -0.38) | -2.941 | 0.003 |
| DALYs | Brazil | Both | -3.04 (-3.37 to -2.71) | -17.752 | <0.001 |
| DALYs | Brunei Darussalam | Both | -2.45 (-2.99 to -1.90) | -8.735 | <0.001 |
| DALYs | Bulgaria | Both | -0.59 (-1.29 to 0.13) | -1.616 | 0.106 |
| DALYs | Burkina Faso | Both | -0.05 (-0.15 to 0.06) | -0.914 | 0.361 |
| DALYs | Burundi | Both | -1.66 (-1.85 to -1.48) | -17.317 | <0.001 |
| DALYs | Cabo Verde | Both | 0.61 (0.24 to 0.99) | 3.228 | 0.001 |
| DALYs | Cambodia | Both | -0.29 (-0.35 to -0.23) | -9.33 | <0.001 |
| DALYs | Cameroon | Both | 0.33 (0.18 to 0.47) | 4.428 | <0.001 |
| DALYs | Canada | Both | -2.40 (-2.55 to -2.25) | -30.292 | <0.001 |
| DALYs | Central African Republic | Both | -0.38 (-0.45 to -0.30) | -9.854 | <0.001 |
| DALYs | Chad | Both | 0.41 (0.25 to 0.57) | 5.034 | <0.001 |
| DALYs | Chile | Both | -2.98 (-3.44 to -2.51) | -12.371 | <0.001 |
| DALYs | China | Both | -0.53 (-0.70 to -0.35) | -5.771 | <0.001 |
| DALYs | Colombia | Both | -3.01 (-3.40 to -2.61) | -14.659 | <0.001 |
| DALYs | Comoros | Both | -1.10 (-1.18 to -1.01) | -25.068 | <0.001 |
| DALYs | Congo | Both | -0.67 (-0.85 to -0.50) | -7.574 | <0.001 |
| DALYs | Cook Islands | Both | -1.55 (-1.60 to -1.51) | -64.603 | <0.001 |
| DALYs | Costa Rica | Both | -1.49 (-2.12 to -0.86) | -4.573 | <0.001 |
| DALYs | C?te d'Ivoire | Both | -0.29 (-0.47 to -0.12) | -3.244 | 0.001 |
| DALYs | Croatia | Both | -3.39 (-3.61 to -3.16) | -29.315 | <0.001 |
| DALYs | Cuba | Both | -0.66 (-0.78 to -0.54) | -11.197 | <0.001 |
| DALYs | Cyprus | Both | -4.20 (-4.96 to -3.44) | -10.624 | <0.001 |
| DALYs | Czechia | Both | -4.79 (-5.27 to -4.31) | -18.972 | <0.001 |
| DALYs | Democratic People's Republic of Korea | Both | -0.12 (-0.17 to -0.08) | -5.425 | <0.001 |
| DALYs | Democratic Republic of the Congo | Both | -0.43 (-0.54 to -0.33) | -8.108 | <0.001 |
| DALYs | Denmark | Both | -3.09 (-3.41 to -2.76) | -18.295 | <0.001 |
| DALYs | Djibouti | Both | -0.14 (-0.28 to -0.01) | -2.121 | 0.034 |
| DALYs | Dominica | Both | -0.66 (-0.75 to -0.57) | -13.793 | <0.001 |
| DALYs | Dominican Republic | Both | -0.20 (-0.63 to 0.23) | -0.906 | 0.365 |
| DALYs | Ecuador | Both | -2.28 (-2.99 to -1.57) | -6.228 | <0.001 |
| DALYs | Egypt | Both | -0.67 (-1.12 to -0.22) | -2.909 | 0.004 |
| DALYs | El Salvador | Both | -1.56 (-2.30 to -0.82) | -4.103 | <0.001 |
| DALYs | Equatorial Guinea | Both | -0.78 (-0.93 to -0.62) | -9.831 | <0.001 |
| DALYs | Eritrea | Both | -0.53 (-0.61 to -0.45) | -13.295 | <0.001 |
| DALYs | Estonia | Both | -4.80 (-5.58 to -4.01) | -11.752 | <0.001 |
| DALYs | Eswatini | Both | 0.14 (-0.02 to 0.30) | 1.702 | 0.089 |
| DALYs | Ethiopia | Both | -0.96 (-1.05 to -0.86) | -19.102 | <0.001 |
| DALYs | Fiji | Both | -0.38 (-0.56 to -0.21) | -4.238 | <0.001 |
| DALYs | Finland | Both | -3.44 (-3.65 to -3.22) | -30.564 | <0.001 |
| DALYs | France | Both | -3.09 (-3.27 to -2.91) | -32.831 | <0.001 |
| DALYs | Gabon | Both | -0.34 (-0.51 to -0.18) | -4.14 | <0.001 |
| DALYs | Gambia | Both | 0.33 (0.05 to 0.61) | 2.336 | 0.02 |
| DALYs | Georgia | Both | 0.60 (-0.66 to 1.88) | 0.93 | 0.353 |
| DALYs | Germany | Both | -3.49 (-3.82 to -3.17) | -20.726 | <0.001 |
| DALYs | Ghana | Both | 0.06 (-0.02 to 0.13) | 1.53 | 0.126 |
| DALYs | Greece | Both | -3.84 (-4.15 to -3.52) | -23.269 | <0.001 |
| DALYs | Greenland | Both | -3.15 (-3.38 to -2.92) | -26.17 | <0.001 |
| DALYs | Grenada | Both | -2.34 (-2.84 to -1.83) | -9.004 | <0.001 |
| DALYs | Guam | Both | -2.40 (-2.99 to -1.81) | -7.865 | <0.001 |
| DALYs | Guatemala | Both | -1.62 (-2.45 to -0.78) | -3.773 | <0.001 |
| DALYs | Guinea | Both | 0.36 (0.27 to 0.45) | 7.793 | <0.001 |
| DALYs | Guinea-Bissau | Both | -0.21 (-0.29 to -0.12) | -4.731 | <0.001 |
| DALYs | Guyana | Both | -1.69 (-2.25 to -1.13) | -5.872 | <0.001 |
| DALYs | Haiti | Both | -0.93 (-1.00 to -0.85) | -23.957 | <0.001 |
| DALYs | Honduras | Both | 0.64 (0.28 to 1.00) | 3.493 | <0.001 |
| DALYs | Hungary | Both | -3.31 (-3.79 to -2.84) | -13.493 | <0.001 |
| DALYs | Iceland | Both | -3.25 (-3.68 to -2.82) | -14.49 | <0.001 |
| DALYs | India | Both | -0.53 (-0.85 to -0.21) | -3.224 | 0.001 |
| DALYs | Indonesia | Both | 0.72 (0.67 to 0.77) | 26.871 | <0.001 |
| DALYs | Iran (Islamic Republic of) | Both | -2.08 (-2.19 to -1.97) | -36.685 | <0.001 |
| DALYs | Iraq | Both | -0.37 (-0.56 to -0.19) | -3.976 | <0.001 |
| DALYs | Ireland | Both | -4.52 (-4.85 to -4.19) | -25.977 | <0.001 |
| DALYs | Israel | Both | -3.37 (-4.21 to -2.52) | -7.66 | <0.001 |
| DALYs | Italy | Both | -3.82 (-4.13 to -3.50) | -22.994 | <0.001 |
| DALYs | Jamaica | Both | -0.87 (-1.59 to -0.15) | -2.358 | 0.018 |
| DALYs | Japan | Both | -3.47 (-3.79 to -3.15) | -20.671 | <0.001 |
| DALYs | Jordan | Both | -2.36 (-2.88 to -1.83) | -8.757 | <0.001 |
| DALYs | Kazakhstan | Both | -0.76 (-1.34 to -0.17) | -2.531 | 0.011 |
| DALYs | Kenya | Both | 0.25 (0.21 to 0.30) | 10.812 | <0.001 |
| DALYs | Kiribati | Both | -0.02 (-0.05 to 0.01) | -1.178 | 0.239 |
| DALYs | Kuwait | Both | -1.47 (-3.23 to 0.31) | -1.622 | 0.105 |
| DALYs | Kyrgyzstan | Both | -1.57 (-2.04 to -1.09) | -6.357 | <0.001 |
| DALYs | Lao People's Democratic Republic | Both | -1.18 (-1.21 to -1.15) | -73.248 | <0.001 |
| DALYs | Latvia | Both | -1.49 (-2.29 to -0.68) | -3.609 | <0.001 |
| DALYs | Lebanon | Both | -2.91 (-3.07 to -2.75) | -34.644 | <0.001 |
| DALYs | Lesotho | Both | 1.61 (1.23 to 1.98) | 8.37 | <0.001 |
| DALYs | Liberia | Both | -0.24 (-0.42 to -0.06) | -2.556 | 0.011 |
| DALYs | Libya | Both | 0.48 (-0.01 to 0.98) | 1.93 | 0.054 |
| DALYs | Lithuania | Both | -1.26 (-1.97 to -0.54) | -3.405 | 0.001 |
| DALYs | Luxembourg | Both | -5.33 (-5.94 to -4.72) | -16.587 | <0.001 |
| DALYs | Madagascar | Both | -0.29 (-0.47 to -0.10) | -3.088 | 0.002 |
| DALYs | Malawi | Both | 0.28 (0.17 to 0.40) | 4.843 | <0.001 |
| DALYs | Malaysia | Both | -0.63 (-0.75 to -0.51) | -10.514 | <0.001 |
| DALYs | Maldives | Both | -2.66 (-2.82 to -2.50) | -31.305 | <0.001 |
| DALYs | Mali | Both | -0.22 (-0.36 to -0.07) | -2.881 | 0.004 |
| DALYs | Malta | Both | -4.49 (-4.92 to -4.07) | -20.258 | <0.001 |
| DALYs | Marshall Islands | Both | -0.44 (-0.53 to -0.34) | -8.976 | <0.001 |
| DALYs | Mauritania | Both | -0.89 (-0.99 to -0.78) | -16.852 | <0.001 |
| DALYs | Mauritius | Both | -3.16 (-3.95 to -2.35) | -7.608 | <0.001 |
| DALYs | Mexico | Both | -2.46 (-2.95 to -1.97) | -9.703 | <0.001 |
| DALYs | Micronesia (Federated States of) | Both | -0.58 (-0.61 to -0.55) | -38.641 | <0.001 |
| DALYs | Monaco | Both | -2.77 (-2.87 to -2.67) | -54.629 | <0.001 |
| DALYs | Mongolia | Both | -0.05 (-0.32 to 0.22) | -0.352 | 0.725 |
| DALYs | Montenegro | Both | 1.26 (0.87 to 1.65) | 6.33 | <0.001 |
| DALYs | Morocco | Both | -0.27 (-0.39 to -0.15) | -4.342 | <0.001 |
| DALYs | Mozambique | Both | 0.71 (0.56 to 0.85) | 9.764 | <0.001 |
| DALYs | Myanmar | Both | -1.17 (-1.22 to -1.12) | -45.78 | <0.001 |
| DALYs | Namibia | Both | -0.33 (-0.55 to -0.11) | -2.947 | 0.003 |
| DALYs | Nauru | Both | -0.35 (-0.42 to -0.29) | -10.159 | <0.001 |
| DALYs | Nepal | Both | -1.02 (-1.08 to -0.97) | -36.804 | <0.001 |
| DALYs | Netherlands | Both | -2.74 (-3.10 to -2.38) | -14.763 | <0.001 |
| DALYs | New Zealand | Both | -2.83 (-3.03 to -2.64) | -28.123 | <0.001 |
| DALYs | Nicaragua | Both | -1.83 (-2.23 to -1.43) | -8.843 | <0.001 |
| DALYs | Niger | Both | -0.18 (-0.26 to -0.09) | -3.941 | <0.001 |
| DALYs | Nigeria | Both | -0.83 (-0.95 to -0.71) | -13.942 | <0.001 |
| DALYs | Niue | Both | -0.53 (-0.63 to -0.42) | -9.983 | <0.001 |
| DALYs | North Macedonia | Both | -0.18 (-0.65 to 0.30) | -0.72 | 0.472 |
| DALYs | Northern Mariana Islands | Both | -1.18 (-1.49 to -0.86) | -7.271 | <0.001 |
| DALYs | Norway | Both | -3.79 (-4.10 to -3.49) | -23.927 | <0.001 |
| DALYs | Oman | Both | -1.40 (-2.02 to -0.78) | -4.384 | <0.001 |
| DALYs | Pakistan | Both | 0.10 (0.03 to 0.17) | 2.83 | 0.005 |
| DALYs | Palau | Both | -0.54 (-0.70 to -0.37) | -6.38 | <0.001 |
| DALYs | Palestine | Both | -1.64 (-1.92 to -1.36) | -11.488 | <0.001 |
| DALYs | Panama | Both | -1.54 (-2.11 to -0.95) | -5.146 | <0.001 |
| DALYs | Papua New Guinea | Both | -0.42 (-0.47 to -0.38) | -18.433 | <0.001 |
| DALYs | Paraguay | Both | -1.14 (-1.33 to -0.94) | -11.513 | <0.001 |
| DALYs | Peru | Both | -1.56 (-2.71 to -0.38) | -2.596 | 0.009 |
| DALYs | Philippines | Both | -0.15 (-0.48 to 0.17) | -0.916 | 0.359 |
| DALYs | Poland | Both | -3.22 (-3.57 to -2.88) | -17.807 | <0.001 |
| DALYs | Portugal | Both | -5.58 (-5.98 to -5.17) | -26.044 | <0.001 |
| DALYs | Puerto Rico | Both | -3.20 (-3.69 to -2.71) | -12.654 | <0.001 |
| DALYs | Qatar | Both | -2.87 (-3.95 to -1.78) | -5.092 | <0.001 |
| DALYs | Republic of Korea | Both | -4.62 (-4.95 to -4.28) | -26.443 | <0.001 |
| DALYs | Republic of Moldova | Both | -1.17 (-1.88 to -0.45) | -3.176 | 0.001 |
| DALYs | Romania | Both | -1.84 (-2.26 to -1.41) | -8.444 | <0.001 |
| DALYs | Russian Federation | Both | -1.84 (-2.53 to -1.15) | -5.19 | <0.001 |
| DALYs | Rwanda | Both | -2.03 (-2.20 to -1.86) | -22.833 | <0.001 |
| DALYs | Saint Kitts and Nevis | Both | -1.85 (-2.67 to -1.02) | -4.372 | <0.001 |
| DALYs | Saint Lucia | Both | -2.74 (-3.49 to -1.99) | -7.061 | <0.001 |
| DALYs | Saint Vincent and the Grenadines | Both | -1.48 (-2.14 to -0.82) | -4.364 | <0.001 |
| DALYs | Samoa | Both | -0.50 (-0.54 to -0.46) | -23.466 | <0.001 |
| DALYs | San Marino | Both | -3.46 (-3.93 to -2.98) | -14.095 | <0.001 |
| DALYs | Sao Tome and Principe | Both | 0.36 (0.15 to 0.57) | 3.374 | 0.001 |
| DALYs | Saudi Arabia | Both | -1.19 (-1.29 to -1.08) | -21.881 | <0.001 |
| DALYs | Senegal | Both | -0.50 (-0.56 to -0.44) | -16.549 | <0.001 |
| DALYs | Serbia | Both | -1.90 (-2.18 to -1.63) | -13.286 | <0.001 |
| DALYs | Seychelles | Both | -1.36 (-2.11 to -0.60) | -3.516 | <0.001 |
| DALYs | Sierra Leone | Both | -0.33 (-0.47 to -0.20) | -4.776 | <0.001 |
| DALYs | Singapore | Both | -5.41 (-6.53 to -4.29) | -9.199 | <0.001 |
| DALYs | Slovakia | Both | -2.17 (-2.53 to -1.81) | -11.798 | <0.001 |
| DALYs | Slovenia | Both | -3.90 (-4.26 to -3.55) | -20.993 | <0.001 |
| DALYs | Solomon Islands | Both | -0.18 (-0.35 to -0.02) | -2.137 | 0.033 |
| DALYs | Somalia | Both | -0.64 (-0.74 to -0.55) | -13.482 | <0.001 |
| DALYs | South Africa | Both | 0.46 (-0.17 to 1.10) | 1.443 | 0.149 |
| DALYs | South Sudan | Both | -0.54 (-0.64 to -0.45) | -11.306 | <0.001 |
| DALYs | Spain | Both | -4.29 (-4.55 to -4.02) | -30.913 | <0.001 |
| DALYs | Sri Lanka | Both | -1.29 (-1.96 to -0.61) | -3.718 | <0.001 |
| DALYs | Sudan | Both | -0.84 (-0.88 to -0.79) | -32.799 | <0.001 |
| DALYs | Suriname | Both | -0.62 (-1.12 to -0.11) | -2.375 | 0.018 |
| DALYs | Sweden | Both | -3.19 (-3.61 to -2.77) | -14.653 | <0.001 |
| DALYs | Switzerland | Both | -3.68 (-3.82 to -3.53) | -49.278 | <0.001 |
| DALYs | Syrian Arab Republic | Both | -0.92 (-1.17 to -0.66) | -6.948 | <0.001 |
| DALYs | Taiwan (Province of China) | Both | -3.43 (-3.94 to -2.91) | -12.894 | <0.001 |
| DALYs | Tajikistan | Both | -0.71 (-1.12 to -0.31) | -3.456 | 0.001 |
| DALYs | Thailand | Both | -1.42 (-1.76 to -1.08) | -8.122 | <0.001 |
| DALYs | Timor-Leste | Both | 0.26 (0.14 to 0.37) | 4.46 | <0.001 |
| DALYs | Togo | Both | -0.10 (-0.19 to 0.00) | -1.937 | 0.053 |
| DALYs | Tokelau | Both | -1.00 (-1.03 to -0.96) | -50.409 | <0.001 |
| DALYs | Tonga | Both | -0.25 (-0.49 to -0.01) | -2.002 | 0.045 |
| DALYs | Trinidad and Tobago | Both | -2.02 (-2.35 to -1.69) | -11.774 | <0.001 |
| DALYs | Tunisia | Both | -0.97 (-1.09 to -0.86) | -16.529 | <0.001 |
| DALYs | T rkiye | Both | -2.02 (-2.30 to -1.74) | -13.916 | <0.001 |
| DALYs | Turkmenistan | Both | 0.71 (-0.04 to 1.46) | 1.86 | 0.063 |
| DALYs | Tuvalu | Both | -0.80 (-0.83 to -0.76) | -42.105 | <0.001 |
| DALYs | Uganda | Both | -0.82 (-0.91 to -0.73) | -17.766 | <0.001 |
| DALYs | Ukraine | Both | -1.84 (-2.47 to -1.20) | -5.631 | <0.001 |
| DALYs | United Arab Emirates | Both | -1.48 (-3.44 to 0.51) | -1.464 | 0.143 |
| DALYs | United Kingdom | Both | -4.06 (-4.42 to -3.70) | -21.433 | <0.001 |
| DALYs | United Republic of Tanzania | Both | 0.63 (0.45 to 0.81) | 6.742 | <0.001 |
| DALYs | United States of America | Both | -1.35 (-1.79 to -0.90) | -5.916 | <0.001 |
| DALYs | United States Virgin Islands | Both | -2.48 (-2.81 to -2.15) | -14.562 | <0.001 |
| DALYs | Uruguay | Both | -2.61 (-2.87 to -2.34) | -18.968 | <0.001 |
| DALYs | Uzbekistan | Both | -0.27 (-0.94 to 0.41) | -0.77 | 0.441 |
| DALYs | Vanuatu | Both | -0.59 (-0.70 to -0.48) | -10.365 | <0.001 |
| DALYs | Venezuela (Bolivarian Republic of) | Both | -0.72 (-1.06 to -0.38) | -4.122 | <0.001 |
| DALYs | Viet Nam | Both | 0.19 (0.14 to 0.24) | 7.435 | <0.001 |
| DALYs | Yemen | Both | -0.29 (-0.43 to -0.16) | -4.418 | <0.001 |
| DALYs | Zambia | Both | 0.45 (0.31 to 0.59) | 6.266 | <0.001 |
| DALYs | Zimbabwe | Both | 1.16 (0.52 to 1.80) | 3.547 | <0.001 |
