# Supplementary material for: Global, regional, and national epidemiology of ischemic stroke from 1990 to 2021
Source: Eur J Neurol. 2024 Sep 17;31(12):e16481. doi: 10.1111/ene.16481 (PMC11555022; doi:10.1111/ene.16481)
Supplement: Supplementary file 3 — TABLE S2. Deaths by ischemic stroke between 1990 and 2021 at the global and regional levels. ASMR, age‐standardized mortality rate; EAPC, estimated annual percentage change. [file ENE-31-e16481-s007.docx]

Supplementary Table 2. Deaths of Ischemic Stroke between 1990 and 2021 at the global and regional levels. ASMR = Age-standardized mortality rate. EAPC = estimated annual percentage change.

| **Location** | **1990** | | **2021** | | **1990-2021** | |
| --- | --- | --- | --- | --- | --- | --- |
|  | **Deaths Cases** | **ASMR** | **Deaths Cases** | **ASMR** | **Cases change** | **EAPC** |
| **Global** | 2317112 (2131460 to 2475546) | 73.15 (66.36 to 77.94) | 3591499 (3213281 to 3888327) | 44.18 (39.29 to 47.81) | 0.55 (0.43 to 0.67) | -1.83 (-1.92 to -1.74) |
| **Regions** |  |  |  |  |  |  |
| **Central Europe, Eastern Europe, and Central Asia** | 619601 (587903 to 635796) | 150.75 (141.45 to 155.29) | 534361 (488931 to 567417) | 80.11 (73.16 to 85.14) | -0.14 (-0.18 to -0.09) | -2.69 (-3.04 to -2.34) |
| Central Europe | 181353 (171927 to 186859) | 140.97 (132.21 to 145.88) | 160200 (144922 to 171306) | 65.59 (59.35 to 70.12) | -0.12 (-0.18 to -0.06) | -2.79 (-2.92 to -2.65) |
| Eastern Europe | 405262 (383950 to 415284) | 168.09 (157.84 to 173) | 329291 (299911 to 356035) | 90.99 (82.79 to 98.48) | -0.19 (-0.24 to -0.13) | -2.78 (-3.24 to -2.32) |
| Central Asia | 32986 (30491 to 35153) | 81.73 (74.95 to 87.16) | 44870 (40632 to 48966) | 70.96 (64.29 to 77.28) | 0.36 (0.23 to 0.52) | -0.87 (-1.11 to -0.63) |
| **High-income** | 629164 (562767 to 661354) | 51.96 (46.17 to 54.77) | 483852 (393659 to 531249) | 16.61 (13.7 to 18.11) | -0.23 (-0.31 to -0.19) | -3.94 (-4.1 to -3.78) |
| Australasia | 9883 (8880 to 10633) | 45.74 (40.52 to 49.31) | 9400 (7588 to 10375) | 14.11 (11.44 to 15.55) | -0.05 (-0.14 to 0.02) | -4.01 (-4.11 to -3.9) |
| High-income Asia Pacific | 105659 (93891 to 112114) | 62.85 (54.94 to 67.16) | 112785 (87155 to 127258) | 15.77 (12.54 to 17.52) | 0.07 (-0.08 to 0.16) | -4.76 (-4.91 to -4.6) |
| High-income North America | 110127 (96140 to 117560) | 29.57 (25.75 to 31.61) | 126353 (103455 to 138349) | 16.76 (13.85 to 18.27) | 0.15 (0.07 to 0.19) | -2.38 (-2.68 to -2.08) |
| Southern Latin America | 23300 (21541 to 24644) | 57.53 (52.69 to 60.88) | 19658 (17471 to 21165) | 21.14 (18.83 to 22.77) | -0.16 (-0.22 to -0.10) | -2.87 (-3.02 to -2.73) |
| Western Europe | 380195 (341008 to 399353) | 62.78 (55.96 to 66.13) | 215656 (175955 to 236146) | 16.72 (13.85 to 18.2) | -0.43 (-0.49 to -0.40) | -4.43 (-4.57 to -4.29) |
| **Latin America and Caribbean** | 97123 (90347 to 100519) | 55.49 (50.93 to 57.67) | 144351 (128996 to 155384) | 24.77 (22.11 to 26.65) | 0.49 (0.39 to 0.58) | -2.57 (-2.67 to -2.47) |
| Andean Latin America | 5510 (4930 to 6092) | 31.72 (28.45 to 34.97) | 9794 (8202 to 11614) | 17.73 (14.85 to 21.01) | 0.78 (0.49 to 1.11) | -2.11 (-2.31 to -1.92) |
| Caribbean | 11986 (11099 to 12742) | 52.96 (48.88 to 56.13) | 19948 (17601 to 22403) | 36.42 (32.14 to 40.94) | 0.66 (0.48 to 0.87) | -1.16 (-1.24 to -1.09) |
| Central Latin America | 24285 (22793 to 25098) | 37.5 (34.9 to 38.88) | 43027 (37956 to 47740) | 18.68 (16.48 to 20.72) | 0.77 (0.61 to 0.96) | -2.39 (-2.56 to -2.23) |
| Tropical Latin America | 55341 (50934 to 57541) | 80.36 (72.6 to 84.33) | 71582 (63128 to 76435) | 29.61 (26.03 to 31.67) | 0.29 (0.21 to 0.35) | -3.02 (-3.14 to -2.91) |
| **North Africa and Middle East** | 131135 (114693 to 148487) | 106.62 (92.27 to 120.78) | 253284 (220812 to 283441) | 73.69 (63.97 to 82.06) | 0.93 (0.69 to 1.21) | -1.17 (-1.23 to -1.11) |
| **South Asia** | 172617 (142120 to 218916) | 43.32 (35.65 to 54.1) | 441296 (382997 to 539467) | 37.98 (33.12 to 45.77) | 1.56 (1.20 to 1.97) | -0.57 (-0.68 to -0.45) |
| **Southeast Asia, East Asia, and Oceania** | 574855 (508672 to 658121) | 73.89 (65.19 to 84.57) | 1545602 (1326302 to 1757131) | 64.17 (55.15 to 73.01) | 1.69 (1.19 to 2.22) | -0.42 (-0.63 to -0.2) |
| East Asia | 442486 (376804 to 522374) | 74.59 (64.1 to 87.38) | 1202218 (1010916 to 1397915) | 63.18 (52.91 to 73.15) | 1.72 (1.15 to 2.40) | -0.52 (-0.76 to -0.27) |
| Southeast Asia | 131563 (114808 to 147289) | 72.59 (62.95 to 81.65) | 341541 (293259 to 391953) | 68.09 (58.72 to 77.47) | 1.60 (1.19 to 2.03) | -0.13 (-0.28 to 0.02) |
| Oceania | 806 (627 to 1041) | 48.66 (39.3 to 62.05) | 1843 (1488 to 2389) | 40.7 (33.33 to 52.33) | 1.29 (0.89 to 1.82) | -0.69 (-0.76 to -0.63) |
| **Sub-Saharan Africa** | 92619 (76938 to 115256) | 63.39 (52.71 to 78.1) | 188753 (163454 to 221022) | 58.85 (51.11 to 68.44) | 1.04 (0.76 to 1.40) | -0.41 (-0.46 to -0.36) |
| Central Sub-Saharan Africa | 8561 (6432 to 10968) | 64.74 (49.96 to 81.82) | 18859 (13940 to 25745) | 59.61 (44.16 to 81.52) | 1.20 (0.73 to 1.78) | -0.44 (-0.48 to -0.41) |
| Eastern Sub-Saharan Africa | 24975 (20419 to 31171) | 51.86 (42.85 to 64.28) | 51923 (43067 to 61814) | 46.35 (38.28 to 55.34) | 1.08 (0.69 to 1.55) | 0.94 (0.47 to 1.42) |
| Southern Sub-Saharan Africa | 9795 (8050 to 11101) | 47.29 (38.49 to 53.89) | 24959 (22677 to 27158) | 60.31 (54.56 to 65.54) | 1.55 (1.28 to 2.05) | -0.36 (-0.45 to -0.27) |
| Western Sub-Saharan Africa | 49287 (38585 to 64038) | 76.45 (60.17 to 98.52) | 93012 (77668 to 111241) | 68.24 (58.16 to 80.98) | 0.89 (0.55 to 1.37) | -0.36 (-0.45 to -0.27) |
