# Supplementary material for: Global, regional, and national epidemiology of ischemic stroke from 1990 to 2021
Source: Eur J Neurol. 2024 Sep 17;31(12):e16481. doi: 10.1111/ene.16481 (PMC11555022; doi:10.1111/ene.16481)
Supplement: Supplementary file 4 — TABLE S3. DALYs due to ischemic stroke between 1990 and 2021 at the global and regional levels. ASDR, age‐standardized DALY rate; DALY, disability‐adjusted life years; EAPC, estimated annual percentage change. [file ENE-31-e16481-s002.docx]

Supplementary Table 3. DALYs of Ischemic Stroke due to between 1990 and 2021 at the global and regional levels. DALYs = Disability-Adjusted Life Years. ASDR = Age-standardized DALYs rate. EAPC = estimated annual percentage change.

| **Location** | **1990** | | **2021** | | **1990-2021** | |
| --- | --- | --- | --- | --- | --- | --- |
|  | **DALYs Cases** | **ASDR** | **DALYs Cases** | **ASDR** | **Cases change** | **EAPC** |
| **Global** | 46176240 (42961948 to 49414586) | 1286.31 (1195.19 to 1376.06) | 70357912 (64329576 to 76007063) | 837.36 (763.73 to 904.98) | 0.52 (0.41 to 0.64) | -1.59 (-1.68 to -1.5) |
| **Regions** |  |  |  |  |  |  |
| **Central Europe, Eastern Europe, and Central Asia** | 11346093 (10916909 to 11699970) | 2543.03 (2439.78 to 2625.73) | 9292425 (8664271 to 9856616) | 1407.63 (1311.67 to 1494.26) | -0.18 (-0.22 to -0.14) | -2.55 (-2.9 to -2.21) |
| Central Europe | 3203848 (3074577 to 3318844) | 2300.32 (2201.64 to 2386.78) | 2593398 (2385309 to 2776480) | 1101.6 (1014.18 to 1180.5) | -0.19 (-0.24 to -0.14) | -2.7 (-2.83 to -2.57) |
| Eastern Europe | 7422390 (7152142 to 7643098) | 2825.98 (2711.24 to 2913.4) | 5713718 (5294961 to 6142848) | 1601.2 (1483.51 to 1723.12) | -0.23 (-0.28 to -0.18) | -2.61 (-3.06 to -2.16) |
| Central Asia | 719856 (669626 to 766900) | 1625.39 (1511.24 to 1730.11) | 985309 (892229 to 1076484) | 1356.09 (1234.19 to 1474.78) | 0.37 (0.24 to 0.52) | -1.04 (-1.3 to -0.78) |
| **High-income** | 10385684 (9559453 to 10986485) | 850.43 (781.8 to 902.03) | 8056812 (7030179 to 8868133) | 325.02 (286 to 359.54) | -0.22 (-0.27 to -0.19) | -3.36 (-3.52 to -3.2) |
| Australasia | 162898 (149974 to 174032) | 714.14 (655.92 to 763.6) | 149125 (129769 to 166039) | 249.45 (216.51 to 278.22) | -0.08 (-0.15 to -0.02) | -3.61 (-3.75 to -3.48) |
| High-income Asia Pacific | 1927883 (1766028 to 2055873) | 1047.13 (954.83 to 1117.76) | 1862337 (1582664 to 2083583) | 335.36 (287.81 to 378.86) | -0.03 (-0.11 to 0.03) | -3.99 (-4.14 to -3.83) |
| High-income North America | 2017603 (1818786 to 2187490) | 554.83 (500.04 to 602.52) | 2384648 (2094174 to 2640685) | 352.61 (309.35 to 392.65) | 0.18 (0.14 to 0.22) | -1.94 (-2.17 to -1.7) |
| Southern Latin America | 424811 (394540 to 451984) | 974.15 (900.63 to 1035.83) | 352925 (323074 to 382286) | 391.27 (357.82 to 424.24) | -0.17 (-0.22 to -0.11) | -2.72 (-2.84 to -2.59) |
| Western Europe | 5852489 (5397742 to 6160459) | 963.97 (888.14 to 1017.38) | 3307777 (2903214 to 3626266) | 297.71 (262.76 to 327.93) | -0.43 (-0.47 to -0.40) | -3.95 (-4.1 to -3.79) |
| **Latin America and Caribbean** | 1921392 (1829079 to 1992532) | 958.39 (904.02 to 993.86) | 2634525 (2417154 to 2823446) | 440.05 (403.42 to 471.79) | 0.37 (0.30 to 0.45) | -2.56 (-2.68 to -2.43) |
| Andean Latin America | 113707 (101758 to 126129) | 574.17 (516.58 to 631.29) | 183296 (154621 to 214987) | 320.11 (270.13 to 375.28) | 0.61 (0.36 to 0.89) | -2.11 (-2.31 to -1.91) |
| Caribbean | 224864 (209624 to 241939) | 904.27 (839.81 to 968.96) | 353682 (313629 to 399101) | 656.53 (582.42 to 741.98) | 0.57 (0.40 to 0.76) | -0.97 (-1.04 to -0.9) |
| Central Latin America | 487685 (464998 to 508604) | 642.81 (610.09 to 669.72) | 804658 (727628 to 889436) | 336.61 (304.38 to 372.04) | 0.65 (0.51 to 0.81) | -2.29 (-2.46 to -2.11) |
| Tropical Latin America | 1095136 (1036359 to 1137132) | 1368.14 (1271.03 to 1425.63) | 1292888 (1180666 to 1372019) | 520.41 (473.32 to 553.1) | 0.18 (0.12 to 0.23) | -3.05 (-3.18 to -2.92) |
| **North Africa and Middle East** | 3002344 (2668214 to 3383384) | 1940.2 (1715.24 to 2180.55) | 5405417 (4711954 to 6041794) | 1329.39 (1165.64 to 1483.03) | 0.80 (0.56 to 1.05) | -1.23 (-1.27 to -1.2) |
| **South Asia** | 4009489 (3328354 to 5033258) | 809.31 (676 to 1013.15) | 9193297 (8004944 to 11543775) | 690.13 (604.04 to 851.21) | 1.29 (0.99 to 1.66) | -0.67 (-0.76 to -0.58) |
| **Southeast Asia, East Asia, and Oceania** | 13306405 (11719280 to 15180507) | 1373.93 (1223.29 to 1567.2) | 31388175 (27400608 to 35226001) | 1189.16 (1036.92 to 1332.23) | 1.36 (0.95 to 1.78) | -0.44 (-0.6 to -0.28) |
| East Asia | 10279514 (8853619 to 12005870) | 1382.7 (1191.08 to 1606.73) | 24021156 (20420316 to 27562229) | 1165.93 (998.23 to 1336.56) | 1.34 (0.89 to 1.86) | -0.53 (-0.71 to -0.35) |
| Southeast Asia | 3004177 (2687919 to 3310660) | 1355.44 (1203.59 to 1499.12) | 7318328 (6238954 to 8317300) | 1266.45 (1088.13 to 1430.09) | 1.44 (1.08 to 1.77) | -0.17 (-0.28 to -0.06) |
| Oceania | 22714 (18480 to 28041) | 937.35 (770.88 to 1171.08) | 48691 (40675 to 59299) | 789.38 (663.2 to 979.96) | 1.14 (0.82 to 1.56) | -0.65 (-0.71 to -0.6) |
| **Sub-Saharan Africa** | 2204831 (1883789 to 2675612) | 1185.37 (1009.37 to 1442.31) | 4387261 (3828654 to 5082038) | 1081.51 (952.47 to 1245.72) | 0.99 (0.75 to 1.29) | -0.31 (-0.42 to -0.2) |
| Central Sub-Saharan Africa | 225424 (175337 to 276839) | 1219.92 (970.28 to 1523.56) | 447161 (347102 to 579987) | 1076.29 (830.85 to 1416.65) | 0.98 (0.60 to 1.45) | -0.54 (-0.59 to -0.49) |
| Eastern Sub-Saharan Africa | 616225 (510230 to 758430) | 981.75 (824.06 to 1188.93) | 1231675 (1050099 to 1440686) | 873.5 (749.02 to 1018.61) | 1.00 (0.65 to 1.40) | -0.47 (-0.51 to -0.44) |
| Southern Sub-Saharan Africa | 224900 (194622 to 249787) | 917.26 (782.58 to 1023.35) | 523153 (480110 to 569334) | 1073.71 (979 to 1165.87) | 1.33 (1.11 to 1.66) | 0.64 (0.22 to 1.06) |
| Western Sub-Saharan Africa | 1138282 (925816 to 1442064) | 1420.18 (1161.28 to 1810.07) | 2185272 (1840340 to 2608225) | 1256.23 (1075.86 to 1478.49) | 0.92 (0.59 to 1.35) | -0.39 (-0.49 to -0.29) |
