# Supplementary material for: Global, regional, and national epidemiology of ischemic stroke from 1990 to 2021
Source: Eur J Neurol. 2024 Sep 17;31(12):e16481. doi: 10.1111/ene.16481 (PMC11555022; doi:10.1111/ene.16481)
Supplement: Supplementary file 5 — TABLE S4. Incidence of ischemic stroke at the national level. ASIR, age‐standardized incidence rate; EAPC, estimated annual percentage change. [file ENE-31-e16481-s005.docx]

Supplementary Table 4. Incidence of Ischemic Stroke at the national level. ASIR = Age-standardized incidence rate. EAPC = estimated annual percentage change.

| **Location** | **1990** | | **2021** | | **1990-2021** | |
| --- | --- | --- | --- | --- | --- | --- |
|  | **Incident cases** | **ASIR** | **Incident cases** | **ASIR** | **Cases change** | **EAPC** |
| Afghanistan | 8249 (7194 to 9493) | 127.89 (112.47 to 146.54) | 13465 (11936 to 15296) | 119.1 (107.07 to 135.36) | 0.63 (0.46 to 0.82) | -0.23 (-0.29 to -0.18) |
| Albania | 1560 (1349 to 1790) | 75.15 (65.18 to 86.39) | 2716 (2339 to 3155) | 66.82 (58.59 to 76.71) | 0.74 (0.59 to 0.89) | -0.28 (-0.4 to -0.16) |
| Algeria | 16095 (13920 to 18485) | 135.7 (119.32 to 155.86) | 40525 (34841 to 46589) | 117.69 (100.27 to 135.03) | 1.52 (1.28 to 1.77) | -0.68 (-0.82 to -0.55) |
| American Samoa | 22 (19 to 25) | 96.42 (82.23 to 111.38) | 35 (31 to 41) | 80.06 (69.92 to 92.16) | 0.63 (0.48 to 0.77) | -0.78 (-0.84 to -0.73) |
| Andorra | 36 (30 to 43) | 65.1 (54.95 to 77.16) | 69 (59 to 83) | 44.85 (38.26 to 53.65) | 0.92 (0.74 to 1.09) | -1.44 (-1.58 to -1.31) |
| Angola | 5207 (4457 to 6035) | 134.71 (115.42 to 155.83) | 14134 (12326 to 16327) | 119.56 (103.19 to 137.57) | 1.71 (1.49 to 1.95) | -0.38 (-0.4 to -0.36) |
| Antigua and Barbuda | 46 (40 to 53) | 82.52 (71.8 to 93.79) | 65 (57 to 73) | 66.3 (58.65 to 74.08) | 0.40 (0.27 to 0.53) | -0.83 (-0.92 to -0.73) |
| Argentina | 29885 (25982 to 34220) | 95.14 (82.91 to 108.17) | 33273 (29391 to 37720) | 59.95 (53.23 to 68) | 0.11 (0.02 to 0.21) | -1.74 (-1.89 to -1.59) |
| Armenia | 3518 (3077 to 4026) | 134.75 (117.64 to 152.8) | 3606 (3213 to 4011) | 86.12 (76.87 to 95.04) | 0.03 (-0.06 to 0.12) | -1.64 (-1.77 to -1.52) |
| Australia | 17582 (16334 to 18840) | 91.7 (85.57 to 97.98) | 23830 (21336 to 26510) | 52.97 (47.66 to 58.79) | 0.36 (0.24 to 0.47) | -2.1 (-2.25 to -1.94) |
| Austria | 11608 (10593 to 12695) | 95.82 (87.86 to 104.13) | 13175 (11499 to 15430) | 72.53 (64.01 to 84.2) | 0.13 (0.01 to 0.30) | -0.6 (-0.91 to -0.29) |
| Azerbaijan | 4606 (3982 to 5303) | 91.15 (79.27 to 104.83) | 9015 (7861 to 10288) | 98.67 (86 to 112.65) | 0.96 (0.80 to 1.13) | 0.65 (0.52 to 0.78) |
| Bahamas | 119 (104 to 136) | 76.23 (66.52 to 87.53) | 240 (210 to 269) | 63.17 (55.5 to 70.04) | 1.03 (0.87 to 1.19) | -0.65 (-0.7 to -0.6) |
| Bahrain | 156 (133 to 182) | 79.53 (70.49 to 89.23) | 492 (422 to 574) | 56.54 (49.74 to 63.5) | 2.16 (1.85 to 2.48) | -1.15 (-1.21 to -1.1) |
| Bangladesh | 39040 (33424 to 44847) | 84.21 (72.53 to 96.55) | 107066 (92201 to 123095) | 83.55 (72.26 to 95.65) | 1.74 (1.50 to 2.02) | -0.03 (-0.13 to 0.08) |
| Barbados | 268 (236 to 306) | 90.77 (80.13 to 102.47) | 345 (300 to 389) | 70.35 (61.64 to 79.01) | 0.29 (0.18 to 0.40) | -0.83 (-0.97 to -0.7) |
| Belarus | 23266 (20087 to 26740) | 183.47 (159.18 to 208.59) | 22187 (19277 to 25305) | 140.59 (122.4 to 159.36) | -0.05 (-0.14 to 0.04) | -0.9 (-1.08 to -0.73) |
| Belgium | 15115 (12938 to 17384) | 99.01 (85.66 to 112.61) | 12982 (11652 to 14236) | 53.95 (48.32 to 58.81) | -0.14 (-0.24 to -0.02) | -1.76 (-2.07 to -1.45) |
| Belize | 59 (51 to 67) | 57.97 (50.13 to 65.78) | 157 (138 to 178) | 52.68 (46.27 to 59) | 1.67 (1.43 to 1.95) | -0.63 (-0.74 to -0.51) |
| Benin | 2482 (2130 to 2842) | 111.92 (96.96 to 129.64) | 5459 (4771 to 6227) | 93.79 (81.98 to 108.25) | 1.20 (1.05 to 1.36) | -0.67 (-0.72 to -0.61) |
| Bermuda | 49 (43 to 55) | 81.87 (71.95 to 92.6) | 67 (58 to 77) | 51.48 (44.62 to 57.93) | 0.39 (0.27 to 0.52) | -1.81 (-1.92 to -1.69) |
| Bhutan | 172 (145 to 201) | 71.62 (60.91 to 84.05) | 390 (338 to 446) | 65.49 (56.97 to 74.41) | 1.27 (1.05 to 1.49) | -0.41 (-0.47 to -0.35) |
| Bolivia (Plurinational State of) | 2143 (1845 to 2479) | 66.23 (56.88 to 76.82) | 4368 (3717 to 5067) | 51.01 (43.5 to 58.82) | 1.04 (0.91 to 1.19) | -1.01 (-1.12 to -0.9) |
| Bosnia and Herzegovina | 6506 (5695 to 7536) | 169.11 (149.04 to 193.22) | 8674 (7495 to 9801) | 145.21 (126.84 to 164.01) | 0.33 (0.20 to 0.49) | -0.38 (-0.45 to -0.31) |
| Botswana | 800 (681 to 938) | 154.47 (131.21 to 181.66) | 2135 (1833 to 2452) | 163.07 (140.39 to 187.06) | 1.67 (1.46 to 1.94) | 0.05 (-0.07 to 0.16) |
| Brazil | 104036 (86381 to 123698) | 119.97 (98.78 to 143.42) | 162711 (137407 to 190295) | 66.33 (55.84 to 77.58) | 0.56 (0.45 to 0.67) | -2.07 (-2.19 to -1.95) |
| Brunei Darussalam | 186 (161 to 216) | 170.56 (147.08 to 198.22) | 318 (271 to 368) | 94.65 (82.41 to 108.59) | 0.71 (0.58 to 0.86) | -2.28 (-2.46 to -2.1) |
| Bulgaria | 19356 (16585 to 22494) | 174.32 (153.49 to 197.75) | 23741 (20664 to 26748) | 168.91 (149.17 to 188.26) | 0.23 (0.11 to 0.36) | 0.02 (-0.04 to 0.08) |
| Burkina Faso | 3458 (2938 to 4065) | 72.95 (63.32 to 84.13) | 7184 (6206 to 8258) | 68.11 (59.59 to 77.27) | 1.08 (0.93 to 1.23) | -0.19 (-0.24 to -0.15) |
| Burundi | 3412 (2959 to 3956) | 141.84 (121.93 to 164.7) | 5160 (4468 to 5998) | 101.86 (87.61 to 117.22) | 0.51 (0.38 to 0.65) | -1.32 (-1.42 to -1.23) |
| Cabo Verde | 193 (166 to 220) | 78.22 (67.32 to 89.98) | 386 (337 to 445) | 84.7 (73.91 to 97.74) | 1.00 (0.85 to 1.18) | 0.26 (0.2 to 0.31) |
| Cambodia | 4143 (3547 to 4808) | 98.97 (84.58 to 116.15) | 11185 (9612 to 12804) | 101.04 (86.8 to 115.54) | 1.70 (1.50 to 1.97) | 0.05 (-0.02 to 0.12) |
| Cameroon | 4257 (3658 to 4950) | 89.31 (76.06 to 104.46) | 12998 (11470 to 14823) | 92.83 (81.29 to 105.65) | 2.05 (1.83 to 2.32) | 0.12 (-0.03 to 0.26) |
| Canada | 32537 (28186 to 37205) | 101.9 (88.42 to 116.35) | 41315 (37976 to 44826) | 61.47 (56.61 to 66.41) | 0.27 (0.15 to 0.42) | -1.4 (-1.54 to -1.25) |
| Central African Republic | 1313 (1114 to 1566) | 118.59 (100.28 to 140.89) | 2378 (2033 to 2760) | 110.87 (94.64 to 127.64) | 0.81 (0.68 to 0.98) | -0.24 (-0.26 to -0.22) |
| Chad | 3072 (2655 to 3555) | 99.18 (85.6 to 114.43) | 6548 (5692 to 7465) | 94.75 (82.4 to 106.75) | 1.13 (0.97 to 1.30) | -0.11 (-0.16 to -0.06) |
| Chile | 9364 (8192 to 10710) | 93.94 (82.28 to 107.07) | 14875 (12895 to 17007) | 59.12 (51.46 to 67.41) | 0.59 (0.45 to 0.73) | -1.54 (-1.69 to -1.38) |
| China | 761191 (621354 to 937712) | 100.05 (81.52 to 120.91) | 2772053 (2295713 to 3319150) | 135.79 (113.25 to 159.83) | 2.64 (2.41 to 2.91) | 0.94 (0.88 to 1) |
| Colombia | 15083 (13149 to 17087) | 84.76 (73.82 to 95.32) | 25862 (22399 to 29348) | 46.33 (40.2 to 52.47) | 0.71 (0.58 to 0.88) | -2.28 (-2.45 to -2.1) |
| Comoros | 268 (230 to 310) | 134.39 (115.04 to 156.75) | 531 (460 to 605) | 111.15 (96.17 to 126.97) | 0.98 (0.80 to 1.16) | -0.75 (-0.84 to -0.67) |
| Congo | 1594 (1371 to 1868) | 152.23 (130.39 to 177.36) | 3397 (2934 to 3894) | 129.87 (112.88 to 148.69) | 1.13 (0.95 to 1.32) | -0.61 (-0.66 to -0.56) |
| Cook Islands | 10 (9 to 12) | 84.5 (72.38 to 96.35) | 19 (17 to 22) | 78.6 (69.1 to 89.68) | 0.85 (0.70 to 1.05) | -0.38 (-0.45 to -0.3) |
| Costa Rica | 1258 (1087 to 1446) | 69.18 (59.99 to 79.9) | 2724 (2343 to 3138) | 49.63 (42.45 to 57.12) | 1.16 (0.98 to 1.35) | -1.31 (-1.46 to -1.16) |
| Croatia | 9225 (8492 to 9967) | 162.75 (149.31 to 175.65) | 9245 (8638 to 9821) | 99.91 (93.83 to 105.89) | 0.00 (-0.06 to 0.06) | -1.56 (-1.63 to -1.49) |
| Cuba | 7247 (6352 to 8188) | 72.01 (63.14 to 81.37) | 11976 (10625 to 13408) | 62.68 (55.79 to 69.82) | 0.65 (0.51 to 0.82) | -0.4 (-0.43 to -0.37) |
| Cyprus | 687 (569 to 809) | 86.79 (73.6 to 100.32) | 781 (689 to 879) | 41.79 (37.07 to 46.75) | 0.14 (0.00 to 0.28) | -3.17 (-3.65 to -2.7) |
| Czechia | 25448 (21824 to 29040) | 188.38 (163.22 to 213.49) | 19392 (16684 to 22281) | 89.64 (78.75 to 101.82) | -0.24 (-0.32 to -0.15) | -2.56 (-2.64 to -2.48) |
| Côte d'Ivoire | 5487 (4762 to 6299) | 115.45 (99.88 to 132.78) | 12507 (10917 to 14192) | 99 (85.55 to 112.59) | 1.28 (1.13 to 1.44) | -0.65 (-0.73 to -0.56) |
| Democratic People's Republic of Korea | 19772 (16993 to 23121) | 132.94 (114.58 to 154.45) | 43909 (38065 to 50459) | 138.14 (119.8 to 157.8) | 1.22 (1.01 to 1.46) | 0.06 (-0.01 to 0.12) |
| Democratic Republic of the Congo | 18017 (15203 to 21286) | 116.96 (98.87 to 136.83) | 37504 (32504 to 43159) | 103.65 (89.49 to 121.06) | 1.08 (0.94 to 1.26) | -0.44 (-0.47 to -0.4) |
| Denmark | 8705 (7750 to 9747) | 106.9 (96.16 to 118.02) | 6056 (5212 to 6934) | 51.86 (44.85 to 59.19) | -0.30 (-0.37 to -0.23) | -2.71 (-2.81 to -2.6) |
| Djibouti | 181 (153 to 212) | 127.85 (108.7 to 148.03) | 778 (675 to 898) | 125.3 (107.13 to 144.77) | 3.29 (2.97 to 3.68) | -0.07 (-0.11 to -0.03) |
| Dominica | 41 (35 to 47) | 69.23 (59.86 to 79.45) | 50 (43 to 57) | 63.6 (54.85 to 71.92) | 0.22 (0.11 to 0.34) | -0.27 (-0.3 to -0.23) |
| Dominican Republic | 2531 (2209 to 2897) | 64.88 (56.74 to 73.52) | 7958 (6951 to 9042) | 79.27 (68.91 to 90.14) | 2.14 (1.94 to 2.40) | 0.73 (0.7 to 0.76) |
| Ecuador | 3876 (3379 to 4375) | 69.11 (60.2 to 77.71) | 8683 (7648 to 9804) | 53.81 (47.7 to 60.53) | 1.24 (1.10 to 1.42) | -0.84 (-0.89 to -0.8) |
| Egypt | 30077 (26448 to 34629) | 114.14 (99.47 to 130.62) | 83090 (73098 to 95678) | 132.1 (117.83 to 151.05) | 1.76 (1.58 to 2.02) | 0.56 (0.47 to 0.65) |
| El Salvador | 1898 (1653 to 2173) | 60.46 (52.2 to 69.24) | 3006 (2631 to 3409) | 45.62 (39.98 to 51.93) | 0.58 (0.47 to 0.72) | -1.13 (-1.26 to -1) |
| Equatorial Guinea | 256 (219 to 303) | 135.92 (116.13 to 158.71) | 550 (470 to 637) | 105.37 (89.02 to 120.93) | 1.14 (0.95 to 1.32) | -0.86 (-0.91 to -0.82) |
| Eritrea | 1414 (1207 to 1673) | 129.8 (110.54 to 150.62) | 3074 (2661 to 3597) | 110.54 (94.94 to 128.23) | 1.17 (1.01 to 1.35) | -0.64 (-0.7 to -0.58) |
| Estonia | 3383 (3056 to 3760) | 170.01 (154.2 to 187.83) | 1888 (1640 to 2159) | 71.54 (62.07 to 81.77) | -0.44 (-0.50 to -0.38) | -3.2 (-3.42 to -2.97) |
| Eswatini | 341 (290 to 398) | 124.1 (105.96 to 146.72) | 662 (566 to 761) | 138.02 (118.93 to 157.69) | 0.94 (0.79 to 1.12) | 0.41 (0.27 to 0.55) |
| Ethiopia | 18703 (15513 to 22347) | 92.49 (76.7 to 111.31) | 31926 (26737 to 37066) | 71.34 (59.45 to 83.39) | 0.71 (0.60 to 0.83) | -0.97 (-1.12 to -0.83) |
| Fiji | 407 (353 to 468) | 113.29 (97.46 to 130.01) | 696 (608 to 799) | 97.97 (86.98 to 111.96) | 0.71 (0.58 to 0.87) | -0.68 (-0.76 to -0.6) |
| Finland | 9293 (8364 to 10288) | 130.49 (118.28 to 143.78) | 9251 (8181 to 10433) | 72.56 (64.2 to 81.55) | 0.00 (-0.09 to 0.08) | -1.93 (-2.16 to -1.7) |
| France | 53999 (49850 to 58582) | 63.92 (59.37 to 68.59) | 70227 (64649 to 76089) | 49.92 (46.1 to 54.07) | 0.30 (0.21 to 0.40) | -0.87 (-0.94 to -0.81) |
| Gabon | 757 (647 to 874) | 135.01 (115.32 to 154.93) | 1209 (1037 to 1381) | 121.87 (104.83 to 139.6) | 0.60 (0.46 to 0.75) | -0.44 (-0.5 to -0.38) |
| Gambia | 451 (393 to 520) | 110.44 (96.18 to 127.29) | 1156 (1015 to 1315) | 105 (92.51 to 118.55) | 1.56 (1.39 to 1.78) | -0.14 (-0.17 to -0.1) |
| Georgia | 6935 (6020 to 7997) | 119.16 (104.27 to 136.56) | 7503 (6565 to 8391) | 125.23 (110.17 to 139.49) | 0.08 (-0.03 to 0.20) | 0.43 (0.27 to 0.59) |
| Germany | 164978 (147734 to 182137) | 131 (118.36 to 143.38) | 151528 (134230 to 167831) | 79.35 (70.79 to 87.8) | -0.08 (-0.16 to 0.00) | -1.74 (-1.82 to -1.65) |
| Ghana | 9761 (8574 to 11214) | 139.79 (121.81 to 161.08) | 26184 (23176 to 29266) | 143.07 (125.62 to 160.38) | 1.68 (1.48 to 1.88) | 0.21 (0.15 to 0.27) |
| Greece | 18570 (16315 to 21348) | 126.05 (111.74 to 142.91) | 19139 (16787 to 21384) | 70.3 (61.49 to 79.32) | 0.03 (-0.07 to 0.14) | -2.2 (-2.38 to -2.03) |
| Greenland | 58 (51 to 67) | 200.27 (176.51 to 227.89) | 56 (49 to 66) | 93.8 (82.84 to 106.01) | -0.03 (-0.12 to 0.06) | -2.99 (-3.23 to -2.76) |
| Grenada | 83 (73 to 94) | 108.54 (96.53 to 123.4) | 87 (78 to 98) | 88.41 (79.88 to 97.82) | 0.06 (-0.04 to 0.16) | -0.8 (-0.95 to -0.64) |
| Guam | 67 (58 to 78) | 93.17 (80.75 to 106.75) | 157 (138 to 178) | 76.36 (67.42 to 86.49) | 1.33 (1.14 to 1.52) | -0.87 (-0.99 to -0.74) |
| Guatemala | 2031 (1746 to 2349) | 58.79 (51.24 to 66.73) | 5070 (4441 to 5755) | 46.88 (41.22 to 52.89) | 1.50 (1.29 to 1.73) | -0.91 (-1.03 to -0.8) |
| Guinea | 3513 (3038 to 4051) | 101.91 (88.35 to 117.35) | 6410 (5655 to 7214) | 102.11 (90.22 to 114.89) | 0.82 (0.69 to 0.97) | 0.06 (0.04 to 0.08) |
| Guinea-Bissau | 526 (452 to 614) | 117.69 (101.78 to 136.96) | 935 (811 to 1071) | 110.55 (96.11 to 127.24) | 0.78 (0.63 to 0.93) | -0.21 (-0.25 to -0.16) |
| Guyana | 522 (463 to 592) | 136.15 (120.02 to 153.84) | 588 (517 to 666) | 98.48 (87.06 to 110.23) | 0.13 (0.05 to 0.22) | -1.16 (-1.23 to -1.09) |
| Haiti | 3023 (2625 to 3500) | 100.33 (87.63 to 115.53) | 6038 (5299 to 6899) | 89.58 (78.86 to 102.55) | 1.00 (0.86 to 1.15) | -0.37 (-0.41 to -0.33) |
| Honduras | 1269 (1083 to 1474) | 60.03 (51.7 to 68.77) | 3689 (3174 to 4231) | 63.21 (55 to 72.34) | 1.91 (1.71 to 2.13) | 0.13 (0.07 to 0.18) |
| Hungary | 26163 (23032 to 29288) | 186.23 (166.11 to 208.34) | 18242 (16024 to 20714) | 94.18 (82.08 to 105.78) | -0.30 (-0.36 to -0.22) | -2.48 (-2.6 to -2.36) |
| Iceland | 310 (268 to 361) | 106.62 (92.53 to 123.59) | 305 (263 to 354) | 52.57 (45.14 to 60.93) | -0.02 (-0.12 to 0.10) | -2.56 (-2.69 to -2.44) |
| India | 302344 (249878 to 362047) | 68.83 (56.98 to 82.79) | 626033 (529532 to 730397) | 55.82 (47.5 to 65.01) | 1.07 (0.95 to 1.20) | -0.9 (-0.99 to -0.8) |
| Indonesia | 116746 (97657 to 138207) | 121.53 (100.61 to 146.65) | 306628 (258913 to 363145) | 142.58 (120.42 to 168.44) | 1.63 (1.48 to 1.78) | 0.6 (0.56 to 0.63) |
| Iran (Islamic Republic of) | 31637 (26816 to 37595) | 118.31 (99.48 to 139.54) | 62281 (53414 to 72121) | 82.8 (70.53 to 95.67) | 0.97 (0.82 to 1.12) | -1.01 (-1.12 to -0.9) |
| Iraq | 11863 (10477 to 13410) | 135.9 (119.05 to 155.14) | 32006 (28248 to 36542) | 138.99 (122.08 to 158.55) | 1.70 (1.48 to 1.94) | 0.16 (0.13 to 0.19) |
| Ireland | 4304 (3698 to 4967) | 105.96 (91.81 to 121.89) | 3086 (2688 to 3582) | 39.6 (34.45 to 45.7) | -0.28 (-0.35 to -0.20) | -4.04 (-4.47 to -3.62) |
| Israel | 4730 (4046 to 5491) | 98.8 (84.97 to 114.02) | 5773 (4961 to 6693) | 46.95 (40.36 to 54.8) | 0.22 (0.10 to 0.34) | -2.23 (-2.4 to -2.06) |
| Italy | 88028 (69503 to 109473) | 100.32 (80.48 to 123.22) | 67741 (59294 to 77123) | 43.72 (38.65 to 49.18) | -0.23 (-0.33 to -0.12) | -2.78 (-2.97 to -2.58) |
| Jamaica | 1538 (1351 to 1743) | 81.99 (71.72 to 93.21) | 2230 (1952 to 2466) | 69.15 (60.39 to 76.8) | 0.45 (0.33 to 0.59) | -0.64 (-0.77 to -0.52) |
| Japan | 171958 (137000 to 211473) | 105.25 (85.23 to 127.63) | 212866 (184985 to 243882) | 62.03 (54.48 to 71.46) | 0.24 (0.10 to 0.39) | -2.16 (-2.37 to -1.96) |
| Jordan | 2292 (1994 to 2628) | 163.17 (142.73 to 186.21) | 9791 (8604 to 10979) | 134.18 (115.82 to 150.54) | 3.27 (2.94 to 3.65) | -0.67 (-0.83 to -0.51) |
| Kazakhstan | 23701 (20963 to 26930) | 192.71 (168.88 to 219.04) | 25493 (22256 to 28888) | 154.82 (135.2 to 173.91) | 0.08 (-0.02 to 0.17) | -0.81 (-0.97 to -0.64) |
| Kenya | 9012 (7473 to 10780) | 101.08 (83.22 to 121.71) | 22370 (19009 to 26101) | 100.56 (85.01 to 117.93) | 1.48 (1.36 to 1.62) | -0.02 (-0.06 to 0.03) |
| Kiribati | 50 (44 to 57) | 127.52 (110.82 to 146.48) | 85 (75 to 97) | 119.14 (104.07 to 136.2) | 0.72 (0.59 to 0.86) | -0.31 (-0.35 to -0.27) |
| Kuwait | 643 (558 to 742) | 86.9 (76.24 to 97.24) | 2494 (2188 to 2826) | 78.6 (68.77 to 87.63) | 2.88 (2.58 to 3.22) | -0.28 (-0.56 to 0) |
| Kyrgyzstan | 4721 (4179 to 5352) | 160.56 (141.55 to 182.61) | 5085 (4471 to 5827) | 107.44 (94.01 to 122.19) | 0.08 (-0.03 to 0.19) | -1.52 (-1.65 to -1.39) |
| Lao People's Democratic Republic | 2301 (1971 to 2694) | 117.08 (101.37 to 134.83) | 4719 (4108 to 5383) | 107.99 (93.86 to 123.96) | 1.05 (0.88 to 1.27) | -0.33 (-0.38 to -0.28) |
| Latvia | 7223 (6351 to 8312) | 205.64 (181.2 to 235.84) | 5679 (5047 to 6349) | 137.46 (122.06 to 153.26) | -0.21 (-0.28 to -0.14) | -1.24 (-1.29 to -1.19) |
| Lebanon | 2248 (1958 to 2551) | 108.95 (94.7 to 123.55) | 5676 (4934 to 6390) | 92.97 (80.91 to 104.75) | 1.52 (1.33 to 1.76) | -0.44 (-0.48 to -0.4) |
| Lesotho | 816 (683 to 953) | 100.31 (83.68 to 117.58) | 1311 (1105 to 1530) | 141.05 (118.86 to 163.07) | 0.61 (0.46 to 0.78) | 1.39 (1.23 to 1.55) |
| Liberia | 1323 (1129 to 1529) | 105.82 (90.42 to 122.17) | 2131 (1848 to 2435) | 85.53 (74.74 to 96.67) | 0.61 (0.47 to 0.75) | -0.62 (-0.68 to -0.57) |
| Libya | 1690 (1502 to 1920) | 77.15 (68.08 to 87.17) | 4785 (4256 to 5402) | 88.63 (78.82 to 100.14) | 1.83 (1.57 to 2.11) | 0.48 (0.44 to 0.51) |
| Lithuania | 9460 (8283 to 10533) | 210.95 (184.72 to 235.11) | 8869 (7639 to 10148) | 151.81 (132.64 to 172.16) | -0.06 (-0.16 to 0.03) | -0.94 (-1.24 to -0.64) |
| Luxembourg | 529 (466 to 597) | 99.71 (88.88 to 111.41) | 441 (399 to 483) | 41.12 (36.97 to 45.17) | -0.17 (-0.23 to -0.08) | -3.13 (-3.53 to -2.73) |
| Madagascar | 7272 (6258 to 8310) | 138.77 (118.61 to 160.39) | 16080 (14010 to 18498) | 137.33 (120.09 to 157.64) | 1.21 (1.04 to 1.39) | -0.07 (-0.12 to -0.02) |
| Malawi | 4574 (3915 to 5278) | 117.47 (100.88 to 135.63) | 8415 (7318 to 9673) | 111.18 (96.46 to 125.85) | 0.84 (0.70 to 0.99) | -0.22 (-0.28 to -0.16) |
| Malaysia | 11396 (9844 to 13000) | 121.67 (104.35 to 140.12) | 26669 (23128 to 30696) | 97.67 (84.22 to 111.44) | 1.34 (1.17 to 1.54) | -0.66 (-0.76 to -0.57) |
| Maldives | 115 (99 to 134) | 128.61 (111.24 to 149.03) | 263 (228 to 301) | 77.65 (67.66 to 88.49) | 1.28 (1.07 to 1.50) | -2 (-2.17 to -1.84) |
| Mali | 3817 (3310 to 4404) | 91.8 (80.09 to 105.42) | 7566 (6539 to 8777) | 76.9 (67.32 to 87.1) | 0.98 (0.85 to 1.11) | -0.61 (-0.66 to -0.56) |
| Malta | 400 (343 to 461) | 96.35 (83.25 to 110.54) | 377 (327 to 433) | 38.89 (33.9 to 44.52) | -0.06 (-0.15 to 0.05) | -3.24 (-3.61 to -2.87) |
| Marshall Islands | 19 (16 to 22) | 108.06 (92.94 to 125.2) | 34 (29 to 39) | 104.33 (91.11 to 119.93) | 0.80 (0.66 to 0.95) | -0.2 (-0.29 to -0.11) |
| Mauritania | 1394 (1210 to 1611) | 129.16 (112.57 to 149.28) | 2176 (1905 to 2478) | 93.63 (81.54 to 106.34) | 0.56 (0.45 to 0.67) | -1.12 (-1.24 to -0.99) |
| Mauritius | 1068 (922 to 1243) | 155.43 (134.16 to 178.17) | 1384 (1183 to 1588) | 80.3 (69.23 to 91.47) | 0.30 (0.18 to 0.43) | -2.85 (-3.12 to -2.58) |
| Mexico | 37407 (31440 to 43861) | 84.8 (71.1 to 100.46) | 65181 (55026 to 75805) | 53.4 (45.27 to 62.34) | 0.74 (0.65 to 0.85) | -1.79 (-1.98 to -1.61) |
| Micronesia (Federated States of) | 57 (49 to 66) | 114.43 (98.67 to 132.88) | 72 (62 to 83) | 107.09 (93.23 to 122.29) | 0.27 (0.16 to 0.38) | -0.27 (-0.3 to -0.25) |
| Monaco | 83 (70 to 96) | 113.44 (97.19 to 131.12) | 62 (54 to 72) | 62.63 (54.19 to 72.25) | -0.25 (-0.32 to -0.16) | -2.25 (-2.45 to -2.05) |
| Mongolia | 784 (667 to 916) | 64.51 (55.16 to 74.92) | 1800 (1570 to 2082) | 73.66 (64.21 to 84.35) | 1.30 (1.10 to 1.56) | 0.53 (0.35 to 0.71) |
| Montenegro | 466 (402 to 533) | 78.12 (66.93 to 89.4) | 716 (620 to 825) | 79.84 (70.01 to 90.54) | 0.54 (0.39 to 0.67) | 0.37 (0.24 to 0.5) |
| Morocco | 16785 (14872 to 19318) | 115.79 (101.7 to 132.29) | 40538 (35616 to 46053) | 123.8 (109.52 to 139.51) | 1.42 (1.23 to 1.63) | 0.23 (0.2 to 0.27) |
| Mozambique | 7508 (6468 to 8785) | 124.55 (106.08 to 145.63) | 16509 (14381 to 18823) | 140.75 (121.89 to 161.68) | 1.20 (1.04 to 1.38) | 0.57 (0.48 to 0.66) |
| Myanmar | 24794 (21396 to 28806) | 113.11 (97.61 to 131.43) | 45302 (39623 to 51753) | 99.13 (86.73 to 112.22) | 0.83 (0.67 to 1.01) | -0.49 (-0.59 to -0.4) |
| Namibia | 918 (781 to 1077) | 153.15 (129.38 to 177.12) | 1726 (1481 to 1990) | 139.47 (119.54 to 160.33) | 0.88 (0.72 to 1.06) | -0.45 (-0.51 to -0.39) |
| Nauru | 6 (5 to 7) | 132.77 (114.51 to 152.43) | 7 (6 to 8) | 104.4 (92.17 to 118.47) | 0.12 (0.04 to 0.21) | -1.02 (-1.13 to -0.91) |
| Nepal | 6045 (5122 to 7065) | 66.68 (56.7 to 77.48) | 13138 (11289 to 15164) | 60.73 (52.48 to 69.03) | 1.17 (0.98 to 1.36) | -0.36 (-0.44 to -0.27) |
| Netherlands | 22329 (19821 to 24995) | 112.09 (99.55 to 125.32) | 19396 (16696 to 22566) | 56.78 (49.36 to 65.57) | -0.13 (-0.21 to -0.03) | -2.83 (-3.08 to -2.58) |
| New Zealand | 3407 (2789 to 4110) | 87.96 (72.67 to 105.56) | 4330 (3709 to 4973) | 51.81 (44.48 to 58.88) | 0.27 (0.15 to 0.38) | -1.8 (-1.92 to -1.68) |
| Nicaragua | 1316 (1135 to 1517) | 82.64 (71.75 to 94.16) | 2774 (2409 to 3150) | 57.59 (50.39 to 65.4) | 1.11 (0.96 to 1.27) | -1.37 (-1.49 to -1.26) |
| Niger | 3288 (2848 to 3842) | 102.99 (88.34 to 119.75) | 8182 (6968 to 9516) | 85.68 (73.89 to 98.11) | 1.49 (1.33 to 1.68) | -0.69 (-0.76 to -0.62) |
| Nigeria | 47011 (39275 to 56144) | 101.32 (83.93 to 122.31) | 91564 (77814 to 107150) | 90.34 (76.49 to 105.63) | 0.95 (0.84 to 1.05) | -0.36 (-0.4 to -0.32) |
| Niue | 3 (2 to 3) | 112.42 (97.65 to 129.75) | 2 (2 to 2) | 96.36 (83.3 to 109.95) | -0.23 (-0.30 to -0.15) | -0.65 (-0.72 to -0.58) |
| North Macedonia | 3783 (3325 to 4340) | 223.53 (196.62 to 255.25) | 5666 (4900 to 6428) | 214.36 (190.48 to 239.92) | 0.50 (0.39 to 0.65) | -0.11 (-0.16 to -0.07) |
| Northern Mariana Islands | 15 (13 to 17) | 82.79 (70.3 to 95.07) | 35 (30 to 41) | 75.96 (65.84 to 86.4) | 1.33 (1.07 to 1.64) | -0.41 (-0.45 to -0.36) |
| Norway | 9248 (7268 to 11546) | 134.55 (107.59 to 165.64) | 7597 (6370 to 8923) | 73.76 (62.47 to 86.2) | -0.18 (-0.26 to -0.09) | -2.36 (-2.59 to -2.12) |
| Oman | 876 (761 to 998) | 113.54 (98.83 to 131.61) | 2338 (2058 to 2635) | 110.04 (96.27 to 123.48) | 1.67 (1.44 to 1.90) | -0.02 (-0.11 to 0.08) |
| Pakistan | 50476 (42202 to 60355) | 89.33 (73.88 to 106.67) | 106744 (91891 to 124115) | 86.53 (73.62 to 100.9) | 1.11 (0.97 to 1.27) | -0.2 (-0.27 to -0.12) |
| Palau | 11 (10 to 13) | 114.35 (98.62 to 131.83) | 22 (19 to 26) | 110.75 (96.42 to 126.72) | 1.03 (0.85 to 1.20) | -0.19 (-0.25 to -0.14) |
| Palestine | 1090 (953 to 1244) | 125.75 (110.44 to 143.2) | 2771 (2483 to 3126) | 123.07 (110.98 to 135.92) | 1.54 (1.35 to 1.75) | 0.05 (-0.1 to 0.21) |
| Panama | 1166 (1003 to 1333) | 78.62 (67.58 to 89.69) | 2535 (2175 to 2897) | 56.27 (48.22 to 64.57) | 1.17 (1.00 to 1.34) | -1.26 (-1.35 to -1.17) |
| Papua New Guinea | 1139 (963 to 1354) | 64.68 (54.68 to 75.72) | 3047 (2614 to 3502) | 61.18 (52.87 to 69.73) | 1.68 (1.49 to 1.88) | -0.24 (-0.28 to -0.19) |
| Paraguay | 1964 (1698 to 2251) | 88.44 (76.8 to 100.94) | 4038 (3526 to 4523) | 70.58 (61.49 to 79.28) | 1.06 (0.88 to 1.27) | -0.85 (-0.91 to -0.79) |
| Peru | 7544 (6546 to 8644) | 60.28 (52.26 to 69.06) | 14365 (12519 to 16441) | 41.81 (36.61 to 47.97) | 0.90 (0.78 to 1.05) | -1.31 (-1.46 to -1.16) |
| Philippines | 20012 (16475 to 23885) | 67.46 (54.86 to 81.97) | 68843 (58884 to 80269) | 85.57 (72.79 to 99.83) | 2.44 (2.19 to 2.75) | 0.87 (0.67 to 1.06) |
| Poland | 46955 (38145 to 56295) | 112.98 (93.21 to 134.43) | 58610 (48783 to 69317) | 81.13 (67.94 to 94.74) | 0.25 (0.16 to 0.35) | -1.19 (-1.27 to -1.11) |
| Portugal | 21444 (18753 to 24301) | 161.91 (144.16 to 182) | 12970 (11437 to 14480) | 48.04 (42.25 to 53.45) | -0.40 (-0.45 to -0.34) | -4.74 (-5.12 to -4.37) |
| Puerto Rico | 1959 (1690 to 2264) | 56.49 (49.09 to 64.93) | 2800 (2434 to 3204) | 39.17 (33.55 to 45.45) | 0.43 (0.31 to 0.56) | -1.45 (-1.55 to -1.35) |
| Qatar | 189 (162 to 218) | 118.99 (105.16 to 136.14) | 929 (753 to 1130) | 77.12 (67.18 to 88.51) | 3.92 (3.42 to 4.50) | -1.62 (-1.96 to -1.29) |
| Republic of Korea | 59463 (51464 to 68651) | 207.53 (181.2 to 236.82) | 66827 (56987 to 76617) | 74.88 (64.39 to 85.28) | 0.12 (0.02 to 0.22) | -3.91 (-4.15 to -3.66) |
| Republic of Moldova | 5155 (4481 to 5912) | 128.9 (113.56 to 146.78) | 6405 (5552 to 7300) | 109.67 (95.42 to 124.36) | 0.24 (0.13 to 0.39) | -0.29 (-0.38 to -0.2) |
| Romania | 46842 (40145 to 54374) | 183.6 (160.89 to 209.61) | 49449 (43256 to 55128) | 129.32 (114.49 to 143.38) | 0.06 (-0.05 to 0.17) | -1.28 (-1.37 to -1.19) |
| Russian Federation | 325936 (266420 to 396726) | 196.72 (163.22 to 232.25) | 337709 (285475 to 395210) | 144.18 (122.84 to 167.4) | 0.04 (-0.04 to 0.11) | -1.01 (-1.15 to -0.88) |
| Rwanda | 4103 (3505 to 4824) | 143.97 (123.48 to 170.04) | 6105 (5230 to 7092) | 100.68 (86.12 to 117.56) | 0.49 (0.37 to 0.60) | -1.5 (-1.68 to -1.32) |
| Saint Kitts and Nevis | 53 (46 to 62) | 147.51 (131.4 to 166.9) | 58 (51 to 67) | 96.49 (85.06 to 108.08) | 0.10 (-0.02 to 0.21) | -1.63 (-1.78 to -1.48) |
| Saint Lucia | 98 (86 to 111) | 118.24 (104.22 to 133) | 168 (148 to 188) | 72.88 (64.73 to 81.69) | 0.71 (0.56 to 0.87) | -1.84 (-2 to -1.67) |
| Saint Vincent and the Grenadines | 71 (62 to 82) | 99.87 (88.01 to 114.03) | 96 (85 to 109) | 71.65 (63.53 to 80.44) | 0.36 (0.25 to 0.48) | -1.13 (-1.29 to -0.96) |
| Samoa | 89 (76 to 104) | 103.11 (88.19 to 120.38) | 137 (120 to 155) | 94.33 (82.43 to 107.11) | 0.54 (0.40 to 0.70) | -0.33 (-0.38 to -0.28) |
| San Marino | 32 (27 to 37) | 89.71 (75.84 to 104.85) | 41 (35 to 48) | 53.74 (45.73 to 62.99) | 0.30 (0.20 to 0.42) | -1.94 (-2.06 to -1.82) |
| Sao Tome and Principe | 82 (70 to 94) | 120.54 (103.79 to 137.8) | 152 (134 to 171) | 124.71 (109.84 to 140.11) | 0.86 (0.73 to 1.01) | 0.1 (0.03 to 0.16) |
| Saudi Arabia | 6660 (5852 to 7603) | 110.66 (96.68 to 127.09) | 19021 (16691 to 21461) | 96 (85.29 to 106.79) | 1.86 (1.57 to 2.16) | -0.52 (-0.65 to -0.39) |
| Senegal | 4191 (3635 to 4874) | 114.66 (99.27 to 131.56) | 8115 (7085 to 9236) | 96.07 (84.76 to 109.55) | 0.94 (0.80 to 1.08) | -0.55 (-0.6 to -0.51) |
| Serbia | 19070 (16325 to 22068) | 192.17 (166.71 to 218.27) | 25510 (22730 to 28366) | 153.32 (137.35 to 168.79) | 0.34 (0.20 to 0.48) | -0.88 (-0.98 to -0.77) |
| Seychelles | 61 (53 to 69) | 106.16 (92.26 to 120.84) | 94 (81 to 108) | 85.53 (74.13 to 97.51) | 0.55 (0.42 to 0.69) | -0.82 (-0.91 to -0.74) |
| Sierra Leone | 2577 (2229 to 2949) | 114.31 (99.74 to 131.43) | 4392 (3873 to 5003) | 101.59 (89.01 to 116.14) | 0.70 (0.58 to 0.83) | -0.36 (-0.39 to -0.34) |
| Singapore | 3340 (2880 to 3833) | 151.33 (131.56 to 173.41) | 4434 (3706 to 5272) | 52.9 (44.59 to 61.91) | 0.33 (0.19 to 0.47) | -4.09 (-4.37 to -3.81) |
| Slovakia | 10282 (8728 to 11758) | 173.61 (148.13 to 197.65) | 10730 (9259 to 12349) | 114.65 (99.42 to 130.5) | 0.04 (-0.05 to 0.16) | -1.4 (-1.65 to -1.14) |
| Slovenia | 3295 (2860 to 3768) | 137.33 (119.71 to 156.05) | 2737 (2438 to 3043) | 59.09 (53.01 to 65.46) | -0.17 (-0.25 to -0.09) | -2.76 (-3.08 to -2.43) |
| Solomon Islands | 173 (149 to 202) | 129.58 (110.95 to 152.28) | 446 (390 to 508) | 132.7 (114.86 to 151.63) | 1.58 (1.39 to 1.78) | 0 (-0.04 to 0.04) |
| Somalia | 2977 (2517 to 3520) | 110.6 (93.54 to 130.5) | 6892 (5920 to 8050) | 99.32 (86.56 to 112.93) | 1.32 (1.14 to 1.49) | -0.36 (-0.4 to -0.33) |
| South Africa | 26248 (21881 to 31358) | 125.3 (103.28 to 151.54) | 50938 (42146 to 60032) | 119.42 (99.34 to 141.13) | 0.94 (0.84 to 1.06) | -0.31 (-0.6 to -0.01) |
| South Sudan | 2776 (2355 to 3258) | 106.89 (92.07 to 125.44) | 3722 (3182 to 4284) | 95.8 (82.37 to 110.18) | 0.34 (0.23 to 0.45) | -0.26 (-0.3 to -0.21) |
| Spain | 60528 (52352 to 69468) | 112.18 (97.74 to 127.54) | 53333 (49630 to 56840) | 54.99 (51.29 to 58.91) | -0.12 (-0.21 to -0.01) | -2.49 (-2.71 to -2.28) |
| Sri Lanka | 10481 (9045 to 11979) | 106.78 (92.05 to 121.35) | 21902 (18948 to 25221) | 88.19 (77.37 to 99.81) | 1.09 (0.94 to 1.26) | -0.74 (-0.86 to -0.63) |
| Sudan | 11138 (9735 to 12762) | 116.47 (102.01 to 132.54) | 24581 (21644 to 27806) | 117.97 (104.01 to 132.45) | 1.21 (1.05 to 1.37) | 0.06 (0 to 0.13) |
| Suriname | 215 (189 to 245) | 84.46 (73.79 to 96.16) | 470 (411 to 529) | 77.41 (67.72 to 87.14) | 1.19 (1.02 to 1.38) | -0.44 (-0.55 to -0.34) |
| Sweden | 15774 (12774 to 19035) | 100.55 (83.1 to 120.37) | 14224 (11664 to 17150) | 62.86 (52.29 to 74.25) | -0.10 (-0.17 to -0.01) | -1.91 (-2.08 to -1.73) |
| Switzerland | 7896 (6913 to 8948) | 73.7 (64.95 to 82.9) | 7835 (6796 to 8925) | 42.58 (36.86 to 48.62) | -0.01 (-0.10 to 0.12) | -1.8 (-1.95 to -1.66) |
| Syrian Arab Republic | 6552 (5841 to 7391) | 107.95 (95.08 to 121.42) | 10526 (9257 to 11913) | 88.66 (78.86 to 99.53) | 0.61 (0.48 to 0.74) | -0.82 (-0.92 to -0.71) |
| Taiwan (Province of China) | 19367 (16805 to 22190) | 133.89 (116.43 to 152.2) | 34128 (29303 to 39374) | 82.63 (71.56 to 94.9) | 0.76 (0.56 to 0.94) | -2.12 (-2.31 to -1.94) |
| Tajikistan | 3110 (2698 to 3556) | 110.36 (94.71 to 126.82) | 6863 (5964 to 7812) | 132.66 (115.52 to 150.94) | 1.21 (1.07 to 1.37) | 0.89 (0.78 to 1) |
| Thailand | 31361 (27123 to 36332) | 89.7 (77.13 to 103.78) | 68522 (59605 to 78340) | 65.5 (57.21 to 74.28) | 1.18 (1.00 to 1.38) | -1.32 (-1.43 to -1.2) |
| Timor-Leste | 237 (202 to 278) | 86.28 (73.46 to 100.27) | 811 (695 to 941) | 98.07 (84.7 to 112.92) | 2.42 (2.06 to 2.85) | 0.46 (0.4 to 0.53) |
| Togo | 1684 (1459 to 1941) | 115.32 (99.93 to 131.43) | 4217 (3691 to 4815) | 101.54 (88.14 to 115.39) | 1.50 (1.33 to 1.70) | -0.45 (-0.5 to -0.4) |
| Tokelau | 1 (1 to 1) | 90.48 (77.66 to 105.21) | 1 (1 to 1) | 76.22 (66.18 to 86.82) | -0.05 (-0.14 to 0.04) | -0.78 (-0.87 to -0.69) |
| Tonga | 42 (36 to 49) | 74.85 (64.09 to 86.35) | 59 (52 to 67) | 72.97 (63.71 to 82.41) | 0.41 (0.29 to 0.55) | -0.16 (-0.2 to -0.11) |
| Trinidad and Tobago | 945 (826 to 1074) | 113.84 (99.94 to 129.56) | 1329 (1146 to 1509) | 72.73 (63.29 to 82.02) | 0.41 (0.29 to 0.53) | -1.7 (-1.81 to -1.6) |
| Tunisia | 4344 (3754 to 4973) | 91.1 (78.63 to 103.14) | 11480 (9981 to 13302) | 91.29 (79.82 to 104.82) | 1.64 (1.41 to 1.89) | -0.01 (-0.1 to 0.09) |
| Türkiye | 2516 (2201 to 2865) | 129.88 (113.29 to 148.42) | 5980 (5235 to 6765) | 147.13 (129.67 to 165.86) | 0.72 (0.57 to 0.90) | 0.71 (0.61 to 0.81) |
| Turkmenistan | 6 (5 to 7) | 98.7 (84.76 to 114.11) | 9 (8 to 11) | 95.49 (83.92 to 109.27) | 1.38 (1.17 to 1.58) | -0.25 (-0.32 to -0.18) |
| Tuvalu | 40404 (35164 to 46703) | 116.38 (100.98 to 134.23) | 69523 (60002 to 79849) | 78.5 (68.38 to 90.17) | 0.54 (0.41 to 0.67) | -1.5 (-1.58 to -1.41) |
| Uganda | 7981 (6693 to 9407) | 119 (101.01 to 139.57) | 16918 (14455 to 19391) | 111.07 (94.32 to 127.41) | 1.12 (0.96 to 1.31) | -0.38 (-0.5 to -0.25) |
| Ukraine | 141423 (115123 to 172949) | 209.54 (172.59 to 249.65) | 107459 (89951 to 126619) | 143.47 (121.28 to 166.97) | -0.24 (-0.30 to -0.18) | -1.51 (-1.63 to -1.39) |
| United Arab Emirates | 1042 (899 to 1198) | 182.87 (161.21 to 209.16) | 7842 (6528 to 9343) | 138.76 (123.25 to 156.8) | 6.53 (5.74 to 7.54) | -1.07 (-1.23 to -0.92) |
| United Kingdom | 94638 (78247 to 112106) | 104.15 (87.4 to 121.92) | 72319 (62977 to 82443) | 55.3 (48.33 to 62.96) | -0.24 (-0.29 to -0.17) | -2.36 (-2.47 to -2.25) |
| United Republic of Tanzania | 11539 (9882 to 13441) | 102.53 (87.76 to 119.16) | 31679 (27731 to 36056) | 119.78 (102.6 to 137.03) | 1.75 (1.55 to 1.95) | 0.8 (0.68 to 0.91) |
| United States of America | 282791 (230222 to 345341) | 88.76 (72.46 to 107.66) | 311587 (259142 to 370269) | 56.32 (47.48 to 66.31) | 0.10 (0.04 to 0.18) | -1.62 (-1.78 to -1.45) |
| United States Virgin Islands | 48 (41 to 55) | 60.19 (52.12 to 68.87) | 100 (87 to 116) | 58.96 (51.63 to 67.55) | 1.10 (0.88 to 1.35) | -0.04 (-0.09 to 0.01) |
| Uruguay | 4921 (4315 to 5556) | 130.92 (115.44 to 147.04) | 3919 (3451 to 4436) | 69.78 (61.72 to 79.11) | -0.20 (-0.27 to -0.13) | -2.33 (-2.48 to -2.17) |
| Uzbekistan | 16067 (14134 to 18284) | 134.61 (118.05 to 152.68) | 37228 (32988 to 42472) | 150.7 (133.6 to 170.26) | 1.32 (1.13 to 1.52) | 0.31 (0.1 to 0.53) |
| Vanuatu | 77 (67 to 90) | 117.64 (101.84 to 137.43) | 209 (183 to 237) | 118.2 (104.12 to 133.47) | 1.71 (1.51 to 1.93) | -0.05 (-0.08 to -0.02) |
| Venezuela (Bolivarian Republic of) | 7215 (6210 to 8374) | 74.22 (64.39 to 86.05) | 16235 (14039 to 18535) | 56.94 (49.43 to 64.88) | 1.25 (1.05 to 1.46) | -0.88 (-0.97 to -0.8) |
| Viet Nam | 42679 (36936 to 49115) | 108.86 (93.99 to 125.52) | 110010 (100113 to 120837) | 116.07 (105.63 to 127.36) | 1.58 (1.31 to 1.81) | 0.11 (0.03 to 0.2) |
| Yemen | 5775 (4988 to 6650) | 113.39 (99.31 to 128.16) | 16560 (14635 to 18601) | 113.21 (100.41 to 126.62) | 1.87 (1.65 to 2.10) | 0.07 (0.02 to 0.13) |
| Zambia | 3382 (2877 to 3950) | 116.08 (98.62 to 135.06) | 9002 (7767 to 10263) | 131.62 (112.65 to 150.46) | 1.66 (1.45 to 1.92) | 0.51 (0.44 to 0.57) |
| Zimbabwe | 4172 (3625 to 4808) | 103.66 (89.89 to 119.51) | 7957 (6834 to 9149) | 122.03 (106.47 to 139.59) | 0.91 (0.75 to 1.08) | 0.69 (0.57 to 0.81) |
