# Supplementary material for: Global, regional, and national epidemiology of ischemic stroke from 1990 to 2021
Source: Eur J Neurol. 2024 Sep 17;31(12):e16481. doi: 10.1111/ene.16481 (PMC11555022; doi:10.1111/ene.16481)
Supplement: Supplementary file 6 — TABLE S5. Deaths by ischemic stroke at the national level. ASMR, age‐standardized mortality rate; EAPC, estimated annual percentage change. [file ENE-31-e16481-s004.docx]

Supplementary Table 5. Deaths of Ischemic Stroke at the national level. ASMR = Age-standardized mortality rate. EAPC = estimated annual percentage change.

| **Location** | **1990** | | **2021** | | **1990-2021** | |
| --- | --- | --- | --- | --- | --- | --- |
|  | **Deaths Cases** | **ASMR** | **Deaths Cases** | **ASMR** | **Cases change** | **EAPC** |
| Afghanistan | 6269 (4499 to 8656) | 117.77 (86.38 to 159.08) | 7556 (5695 to 10192) | 108.41 (82.72 to 142.91) | 0.21 (-0.09 to 0.56) | -0.35 (-0.5 to -0.2) |
| Albania | 846 (695 to 1022) | 54.27 (44.65 to 65.3) | 1814 (1353 to 2401) | 44.94 (33.76 to 58.99) | 1.14 (0.50 to 1.96) | -0.15 (-0.43 to 0.13) |
| Algeria | 7870 (6251 to 9801) | 116.09 (93.87 to 141.09) | 19111 (14778 to 23724) | 81.97 (64.4 to 99.85) | 1.43 (0.93 to 2.17) | -0.85 (-0.92 to -0.78) |
| American Samoa | 6 (5 to 7) | 47.22 (40.46 to 53.49) | 12 (10 to 15) | 35.65 (29.89 to 43.01) | 0.98 (0.60 to 1.48) | -1.13 (-1.28 to -0.99) |
| Andorra | 13 (9 to 16) | 28.79 (21.42 to 36.92) | 26 (18 to 33) | 13.8 (9.93 to 18) | 1.04 (0.44 to 1.85) | -2.12 (-2.34 to -1.91) |
| Angola | 1493 (1161 to 1880) | 65.5 (50.73 to 80.47) | 4261 (3365 to 5331) | 63.73 (51.25 to 79.61) | 1.85 (1.06 to 2.79) | -0.34 (-0.43 to -0.24) |
| Antigua and Barbuda | 34 (32 to 37) | 57.53 (52.99 to 61.57) | 32 (30 to 34) | 37.38 (34.65 to 40.36) | -0.08 (-0.15 to 0.01) | -1.57 (-1.81 to -1.32) |
| Argentina | 15576 (14328 to 16629) | 55.05 (50.05 to 58.78) | 11397 (10147 to 12308) | 19.21 (17.13 to 20.75) | -0.27 (-0.33 to -0.20) | -3 (-3.22 to -2.78) |
| Armenia | 1645 (1507 to 1755) | 74.53 (68.16 to 79.56) | 2199 (1927 to 2456) | 50.96 (44.67 to 56.92) | 0.34 (0.20 to 0.52) | -2.2 (-2.56 to -1.83) |
| Australia | 8181 (7337 to 8814) | 45.8 (40.66 to 49.42) | 7568 (6099 to 8378) | 13.28 (10.75 to 14.66) | -0.07 (-0.17 to 0.00) | -4.2 (-4.31 to -4.08) |
| Austria | 8494 (7696 to 9013) | 67.33 (60.71 to 71.6) | 3185 (2617 to 3504) | 13.22 (11.05 to 14.46) | -0.63 (-0.66 to -0.60) | -5.64 (-6 to -5.28) |
| Azerbaijan | 1823 (1442 to 2245) | 45.47 (36.24 to 55.93) | 2873 (2214 to 3658) | 39.25 (30.84 to 49.44) | 0.58 (0.09 to 1.21) | -0.03 (-0.31 to 0.24) |
| Bahamas | 52 (47 to 58) | 40.96 (36.54 to 45.17) | 93 (79 to 111) | 28.87 (24.32 to 33.98) | 0.78 (0.46 to 1.18) | -1.18 (-1.35 to -1.02) |
| Bahrain | 82 (72 to 91) | 97.11 (85.62 to 107.64) | 211 (175 to 249) | 57.1 (49.39 to 66.82) | 1.57 (1.10 to 2.16) | -2.15 (-2.63 to -1.67) |
| Bangladesh | 28711 (22481 to 36681) | 78.02 (61.52 to 98.61) | 78848 (61369 to 101644) | 73.21 (57.42 to 92.42) | 1.75 (1.16 to 2.62) | -0.36 (-0.73 to 0.02) |
| Barbados | 228 (209 to 243) | 73.02 (66.78 to 77.69) | 231 (190 to 274) | 44.41 (36.52 to 52.61) | 0.01 (-0.17 to 0.22) | -1.81 (-2.03 to -1.59) |
| Belarus | 12842 (11796 to 13644) | 104.04 (95.76 to 110.78) | 12332 (10422 to 14334) | 74.42 (63.09 to 86.47) | -0.04 (-0.18 to 0.12) | -1.74 (-2.12 to -1.36) |
| Belgium | 9134 (8132 to 9767) | 57.54 (50.91 to 61.58) | 4775 (3803 to 5321) | 14.99 (12.24 to 16.6) | -0.48 (-0.53 to -0.44) | -4.17 (-4.29 to -4.04) |
| Belize | 27 (24 to 29) | 29.51 (26.64 to 31.9) | 63 (56 to 71) | 26.17 (22.88 to 29.23) | 1.37 (1.11 to 1.68) | -0.79 (-1.34 to -0.24) |
| Benin | 1428 (1173 to 1755) | 86.78 (71.52 to 106.71) | 2829 (2305 to 3544) | 76.6 (63.08 to 95.04) | 0.98 (0.65 to 1.47) | -0.36 (-0.44 to -0.27) |
| Bermuda | 27 (25 to 30) | 50.7 (46.22 to 55.09) | 32 (27 to 39) | 20.21 (17 to 24.2) | 0.18 (0.00 to 0.46) | -3.14 (-3.31 to -2.96) |
| Bhutan | 70 (46 to 96) | 44.93 (28.25 to 61.31) | 200 (156 to 245) | 38.05 (29.76 to 46.31) | 1.86 (1.16 to 2.96) | -0.56 (-0.64 to -0.47) |
| Bolivia (Plurinational State of) | 1210 (875 to 1583) | 49.87 (36.63 to 62.81) | 2142 (1513 to 2965) | 30.57 (21.85 to 41.58) | 0.77 (0.35 to 1.38) | -1.53 (-1.66 to -1.41) |
| Bosnia and Herzegovina | 3819 (3323 to 4351) | 123.9 (108.74 to 140.08) | 5857 (4690 to 6913) | 90.31 (72.38 to 106.72) | 0.53 (0.23 to 0.92) | -1.33 (-1.51 to -1.14) |
| Botswana | 288 (222 to 356) | 84.16 (64.71 to 102.95) | 543 (443 to 676) | 56.9 (46.63 to 70.87) | 0.89 (0.43 to 1.61) | -1.15 (-1.38 to -0.91) |
| Brazil | 54237 (49977 to 56408) | 81.01 (73.23 to 85.04) | 69595 (61559 to 74395) | 29.42 (25.93 to 31.49) | 0.28 (0.20 to 0.34) | -3.07 (-3.19 to -2.96) |
| Brunei Darussalam | 54 (46 to 62) | 76.52 (66.37 to 88.29) | 69 (59 to 81) | 38.53 (32.29 to 44.72) | 0.29 (0.06 to 0.58) | -1.82 (-2.12 to -1.51) |
| Bulgaria | 15460 (14858 to 16068) | 181.53 (173.23 to 187.78) | 21792 (19148 to 24451) | 147.63 (130.31 to 164.65) | 0.41 (0.24 to 0.58) | -0.4 (-0.58 to -0.22) |
| Burkina Faso | 1394 (1043 to 1850) | 47.2 (35.95 to 61.26) | 3120 (2416 to 4062) | 47.64 (37.4 to 61.1) | 1.24 (0.74 to 1.93) | 0.3 (0.17 to 0.42) |
| Burundi | 1479 (1062 to 1969) | 84.08 (61.03 to 110.14) | 1576 (1157 to 2135) | 52.52 (39.33 to 69.99) | 0.07 (-0.21 to 0.46) | -2.05 (-2.28 to -1.82) |
| Cabo Verde | 122 (98 to 149) | 50.05 (40.01 to 61.55) | 268 (219 to 325) | 66.28 (53.89 to 80.3) | 1.20 (0.73 to 1.92) | 0.61 (0.25 to 0.97) |
| Cambodia | 2250 (1839 to 2772) | 76.68 (63.2 to 93.51) | 6007 (4680 to 7254) | 75.23 (60.06 to 88.8) | 1.67 (1.02 to 2.40) | -0.14 (-0.25 to -0.03) |
| Cameroon | 1950 (1459 to 2513) | 64.68 (49.88 to 80.84) | 5974 (4480 to 8270) | 71.3 (55.1 to 95.48) | 2.06 (1.32 to 3.18) | 0.39 (0.01 to 0.77) |
| Canada | 10577 (9378 to 11335) | 33.87 (29.78 to 36.32) | 10781 (8823 to 11889) | 12.24 (10.11 to 13.45) | 0.02 (-0.07 to 0.10) | -3.7 (-3.9 to -3.5) |
| Central African Republic | 503 (352 to 681) | 78.33 (56.63 to 103.51) | 797 (562 to 1142) | 71.38 (50.28 to 101.28) | 0.59 (0.20 to 1.02) | -0.36 (-0.42 to -0.3) |
| Chad | 1579 (1176 to 2193) | 68.2 (51.12 to 94.49) | 3200 (2399 to 4271) | 78.38 (60.3 to 102.79) | 1.03 (0.55 to 1.69) | 0.42 (0.25 to 0.58) |
| Chile | 4860 (4543 to 5118) | 57.55 (53.5 to 60.56) | 5943 (5229 to 6456) | 22.34 (19.67 to 24.26) | 0.22 (0.12 to 0.32) | -2.51 (-2.73 to -2.29) |
| China | 427970 (362335 to 506370) | 75.22 (64.48 to 88.23) | 1176952 (986876 to 1372707) | 64.47 (54.03 to 74.82) | 1.75 (1.16 to 2.47) | -0.47 (-0.73 to -0.22) |
| Colombia | 5393 (5023 to 5651) | 38.39 (35.42 to 40.33) | 8179 (6817 to 9465) | 14.54 (12.12 to 16.81) | 0.52 (0.28 to 0.78) | -3.6 (-3.84 to -3.37) |
| Comoros | 86 (65 to 111) | 66.15 (50.96 to 83.17) | 173 (131 to 225) | 48.3 (37.22 to 62.06) | 1.02 (0.49 to 1.76) | -1.3 (-1.48 to -1.11) |
| Congo | 554 (421 to 689) | 83.6 (65.05 to 102.89) | 1097 (847 to 1345) | 69.59 (54.49 to 85.34) | 0.98 (0.53 to 1.49) | -0.82 (-0.92 to -0.71) |
| Cook Islands | 4 (3 to 5) | 43.76 (37.36 to 51.76) | 6 (5 to 7) | 23.67 (18.53 to 29.29) | 0.49 (0.14 to 0.90) | -2.07 (-2.23 to -1.91) |
| Costa Rica | 419 (376 to 450) | 26.67 (23.81 to 28.61) | 904 (758 to 1017) | 16.01 (13.55 to 17.95) | 1.16 (0.90 to 1.40) | -2.17 (-2.58 to -1.76) |
| Croatia | 6963 (6573 to 7335) | 140.26 (131.38 to 147.84) | 5005 (4355 to 5622) | 48.19 (41.91 to 54.11) | -0.28 (-0.36 to -0.20) | -3.65 (-3.8 to -3.5) |
| Cuba | 4070 (3790 to 4318) | 43.62 (40.37 to 46.22) | 7357 (6482 to 8238) | 34.32 (30.27 to 38.41) | 0.81 (0.57 to 1.07) | -0.81 (-0.93 to -0.69) |
| Cyprus | 553 (468 to 644) | 125.64 (106.25 to 145.63) | 512 (420 to 599) | 33.59 (27.64 to 39.27) | -0.07 (-0.28 to 0.16) | -4.52 (-4.88 to -4.17) |
| Czechia | 21031 (19719 to 22126) | 157.73 (146.34 to 166.12) | 7306 (6383 to 8175) | 30.5 (26.66 to 34.13) | -0.65 (-0.69 to -0.61) | -5.73 (-6.17 to -5.29) |
| Côte d'Ivoire | 2060 (1638 to 2547) | 83.48 (68.35 to 98.69) | 5713 (4348 to 7458) | 77.56 (61.54 to 97.9) | 1.77 (1.15 to 2.66) | -0.28 (-0.45 to -0.12) |
| Democratic People's Republic of Korea | 8004 (6100 to 10383) | 68.95 (51.97 to 88.77) | 18869 (14851 to 24754) | 64.14 (50.56 to 84.17) | 1.36 (0.84 to 2.04) | -0.27 (-0.44 to -0.09) |
| Democratic Republic of the Congo | 5641 (4081 to 7553) | 62.19 (46.11 to 82.51) | 12127 (8087 to 17786) | 57.16 (38.15 to 84.41) | 1.15 (0.55 to 1.91) | -0.38 (-0.42 to -0.33) |
| Denmark | 4399 (3993 to 4678) | 48.7 (44.07 to 51.79) | 2691 (2258 to 2930) | 18.74 (15.78 to 20.36) | -0.39 (-0.44 to -0.34) | -3.43 (-3.63 to -3.23) |
| Djibouti | 40 (29 to 58) | 55.59 (41.21 to 78.86) | 189 (140 to 261) | 53.67 (41.7 to 71.43) | 3.73 (2.34 to 5.81) | -0.25 (-0.29 to -0.2) |
| Dominica | 37 (33 to 42) | 66.38 (59.46 to 73.9) | 40 (35 to 45) | 54.08 (47.18 to 61.61) | 0.06 (-0.10 to 0.24) | -0.72 (-0.8 to -0.65) |
| Dominican Republic | 1146 (982 to 1312) | 41.35 (35.46 to 47.29) | 3264 (2579 to 4128) | 34.43 (27.27 to 43.45) | 1.85 (1.17 to 2.79) | -0.07 (-0.35 to 0.21) |
| Ecuador | 1600 (1451 to 1728) | 36.27 (33 to 39.11) | 2789 (2322 to 3318) | 19.35 (16.25 to 22.83) | 0.74 (0.45 to 1.08) | -1.85 (-2.12 to -1.58) |
| Egypt | 30340 (22710 to 40435) | 182.6 (140.44 to 236.47) | 54667 (42714 to 68604) | 139.76 (113.61 to 167.33) | 0.80 (0.39 to 1.37) | -0.33 (-0.52 to -0.15) |
| El Salvador | 688 (599 to 773) | 24.85 (21.59 to 27.97) | 1085 (870 to 1316) | 15.75 (12.65 to 19.06) | 0.58 (0.25 to 0.92) | -1.67 (-1.96 to -1.37) |
| Equatorial Guinea | 89 (66 to 120) | 71.56 (54.04 to 95.85) | 182 (123 to 255) | 57.32 (39.94 to 78.36) | 1.04 (0.32 to 2.06) | -0.97 (-1.17 to -0.76) |
| Eritrea | 332 (222 to 482) | 61.8 (42.96 to 87.09) | 823 (600 to 1094) | 55.31 (41 to 71.74) | 1.48 (0.87 to 2.38) | -0.42 (-0.48 to -0.36) |
| Estonia | 2703 (2524 to 2846) | 136.94 (127.64 to 144.38) | 886 (754 to 994) | 26.43 (22.6 to 29.58) | -0.67 (-0.71 to -0.63) | -6.83 (-7.44 to -6.22) |
| Eswatini | 133 (103 to 167) | 73.3 (57.72 to 90.11) | 256 (181 to 351) | 74.37 (54.25 to 97.27) | 0.93 (0.36 to 1.71) | 0.55 (0.15 to 0.95) |
| Ethiopia | 4091 (2698 to 6346) | 34.6 (23.82 to 50.91) | 8533 (6624 to 11236) | 27.17 (20.92 to 35.61) | 1.09 (0.28 to 1.95) | -1.04 (-1.12 to -0.95) |
| Fiji | 99 (84 to 118) | 45.94 (38.33 to 54.39) | 200 (159 to 250) | 43.14 (35.16 to 52.83) | 1.02 (0.55 to 1.71) | -0.63 (-0.86 to -0.39) |
| Finland | 4269 (3800 to 4582) | 58.78 (51.97 to 63.28) | 3232 (2600 to 3593) | 18.98 (15.5 to 20.96) | -0.24 (-0.33 to -0.18) | -3.62 (-3.76 to -3.48) |
| France | 40806 (36321 to 43476) | 44.58 (39.68 to 47.43) | 28514 (23424 to 31457) | 13.28 (11.08 to 14.6) | -0.30 (-0.37 to -0.25) | -3.82 (-3.96 to -3.68) |
| Gabon | 281 (218 to 347) | 63.47 (49.58 to 78.39) | 395 (297 to 502) | 58.3 (44.5 to 72.98) | 0.41 (0.09 to 0.83) | -0.4 (-0.54 to -0.26) |
| Gambia | 201 (152 to 264) | 80.55 (61.93 to 104.38) | 674 (497 to 881) | 91.15 (67.48 to 118.58) | 2.35 (1.44 to 3.53) | 0.4 (0.32 to 0.48) |
| Georgia | 4268 (3553 to 4892) | 77.58 (65.19 to 88.22) | 6374 (5661 to 7047) | 95.53 (85.31 to 105.53) | 0.49 (0.27 to 0.84) | 0.76 (0.07 to 1.45) |
| Germany | 92044 (81994 to 98545) | 67.54 (59.56 to 72.21) | 43942 (36060 to 48549) | 17.3 (14.43 to 18.98) | -0.52 (-0.57 to -0.48) | -4.46 (-4.69 to -4.22) |
| Ghana | 4165 (3293 to 5368) | 97.41 (77.92 to 122.91) | 11748 (9003 to 14986) | 102.39 (79.96 to 129.64) | 1.82 (1.10 to 2.76) | 0.4 (0.19 to 0.62) |
| Greece | 14457 (13095 to 15215) | 103.58 (93.25 to 109.3) | 10915 (9136 to 11881) | 29.58 (25 to 32.04) | -0.24 (-0.31 to -0.20) | -4.79 (-5.17 to -4.42) |
| Greenland | 18 (16 to 20) | 101.96 (88.49 to 115.21) | 15 (12 to 18) | 35.8 (29 to 44.23) | -0.18 (-0.34 to 0.03) | -3.62 (-3.79 to -3.44) |
| Grenada | 76 (69 to 84) | 91.13 (81.85 to 99.81) | 45 (40 to 49) | 52.16 (46.06 to 57.24) | -0.41 (-0.49 to -0.33) | -1.76 (-1.99 to -1.53) |
| Guam | 18 (16 to 20) | 43.68 (37.92 to 48.72) | 26 (21 to 30) | 11.94 (10.06 to 14) | 0.43 (0.17 to 0.74) | -3.69 (-4.14 to -3.24) |
| Guatemala | 668 (597 to 731) | 31.43 (28.5 to 34.04) | 1654 (1441 to 1878) | 18.74 (16.47 to 21.08) | 1.48 (1.10 to 1.94) | -2.19 (-2.52 to -1.85) |
| Guinea | 1889 (1368 to 2448) | 70.69 (51.26 to 91.17) | 3477 (2591 to 4566) | 79.69 (61.12 to 103.65) | 0.84 (0.37 to 1.55) | 0.68 (0.55 to 0.82) |
| Guinea-Bissau | 303 (226 to 394) | 106.83 (82.79 to 134.49) | 472 (366 to 612) | 105.62 (81.93 to 131.52) | 0.56 (0.19 to 1.07) | 0.14 (0.08 to 0.2) |
| Guyana | 351 (320 to 386) | 113.85 (104.1 to 125.04) | 344 (281 to 421) | 71.46 (59.03 to 86.12) | -0.02 (-0.23 to 0.21) | -1 (-1.24 to -0.77) |
| Haiti | 2371 (1846 to 2912) | 112.2 (88.74 to 135.08) | 3979 (2871 to 5532) | 85.67 (63.75 to 116.24) | 0.68 (0.26 to 1.21) | -0.76 (-0.82 to -0.71) |
| Honduras | 658 (519 to 799) | 41.45 (32.96 to 50.75) | 2605 (2002 to 3350) | 54.89 (41.9 to 69.77) | 2.96 (2.17 to 4.08) | 1.05 (0.81 to 1.3) |
| Hungary | 18000 (16994 to 18839) | 133.71 (125.81 to 140.22) | 9325 (8066 to 10520) | 42.36 (36.69 to 47.85) | -0.48 (-0.55 to -0.42) | -4.05 (-4.25 to -3.85) |
| Iceland | 124 (110 to 134) | 39.46 (35.03 to 42.5) | 99 (77 to 113) | 13.36 (10.51 to 15.14) | -0.20 (-0.29 to -0.11) | -3.6 (-3.81 to -3.38) |
| India | 119411 (96953 to 151673) | 37.36 (30.37 to 46.68) | 313668 (267425 to 393593) | 32.61 (28.05 to 40.04) | 1.63 (1.20 to 2.10) | -0.52 (-0.67 to -0.37) |
| Indonesia | 47043 (38224 to 55937) | 72.04 (57.55 to 86.44) | 148107 (111694 to 183952) | 98.58 (77.46 to 120.69) | 2.15 (1.38 to 2.99) | 1.1 (0.93 to 1.26) |
| Iran (Islamic Republic of) | 16907 (15367 to 18429) | 99.79 (89.3 to 108.93) | 34027 (30355 to 37132) | 52.97 (47.06 to 57.9) | 1.01 (0.81 to 1.24) | -2.19 (-2.31 to -2.06) |
| Iraq | 8995 (7421 to 10421) | 124.98 (103.2 to 144.24) | 20402 (16163 to 24219) | 123.1 (99.29 to 144.15) | 1.27 (0.74 to 1.87) | -0.65 (-0.87 to -0.43) |
| Ireland | 2383 (2185 to 2513) | 62.3 (56.4 to 65.91) | 1268 (1013 to 1416) | 14.41 (11.53 to 16.09) | -0.47 (-0.54 to -0.42) | -4.65 (-4.86 to -4.44) |
| Israel | 1556 (1413 to 1669) | 35.87 (32.14 to 38.41) | 1592 (1303 to 1766) | 10.83 (8.94 to 11.97) | 0.02 (-0.11 to 0.13) | -4.35 (-4.54 to -4.16) |
| Italy | 57300 (50479 to 60832) | 65.92 (57.39 to 70.33) | 41773 (32901 to 46706) | 19.19 (15.35 to 21.33) | -0.27 (-0.35 to -0.23) | -4.17 (-4.38 to -3.96) |
| Jamaica | 1269 (1153 to 1369) | 66.94 (60.67 to 72.25) | 1624 (1293 to 1988) | 47.44 (38.16 to 58.02) | 0.28 (0.02 to 0.58) | -0.99 (-1.38 to -0.61) |
| Japan | 83151 (72927 to 88236) | 56.45 (48.74 to 60.37) | 88917 (67686 to 100981) | 14.18 (11.28 to 15.84) | 0.07 (-0.08 to 0.16) | -4.66 (-4.82 to -4.5) |
| Jordan | 1025 (862 to 1226) | 110.17 (91.98 to 130.03) | 2630 (2105 to 3182) | 54.38 (43.81 to 66.14) | 1.57 (0.99 to 2.36) | -2.77 (-3.1 to -2.44) |
| Kazakhstan | 12603 (11224 to 13885) | 119.17 (106.42 to 131.3) | 13175 (11609 to 14822) | 101.1 (89.95 to 112.91) | 0.05 (-0.10 to 0.24) | -1 (-1.43 to -0.56) |
| Kenya | 2120 (1561 to 2789) | 36.85 (26.82 to 48.55) | 5802 (4316 to 7372) | 41.75 (30.64 to 53.33) | 1.74 (1.18 to 2.41) | 0.62 (0.53 to 0.72) |
| Kiribati | 12 (10 to 15) | 51.24 (38.94 to 61.8) | 24 (19 to 29) | 54.49 (44.84 to 65.99) | 0.89 (0.50 to 1.54) | 0.16 (0.11 to 0.21) |
| Kuwait | 147 (130 to 160) | 35.82 (31.13 to 39.15) | 539 (445 to 646) | 25.73 (20.95 to 30.85) | 2.68 (2.09 to 3.36) | -0.88 (-1.97 to 0.22) |
| Kyrgyzstan | 2921 (2638 to 3205) | 114.1 (102.59 to 125.02) | 2323 (1976 to 2684) | 59.19 (50.17 to 68.23) | -0.20 (-0.33 to -0.06) | -2.55 (-2.88 to -2.21) |
| Lao People's Democratic Republic | 1547 (1231 to 1931) | 107.33 (86.67 to 133.08) | 2496 (1917 to 3134) | 78.23 (61.17 to 96.66) | 0.61 (0.18 to 1.19) | -1.17 (-1.25 to -1.09) |
| Latvia | 5182 (4873 to 5470) | 148.41 (139.01 to 156.85) | 4304 (3770 to 4748) | 87.79 (77.02 to 96.9) | -0.17 (-0.25 to -0.08) | -2.16 (-2.42 to -1.89) |
| Lebanon | 1252 (994 to 1598) | 75.33 (60.07 to 95.4) | 1910 (1572 to 2245) | 28.21 (23.26 to 33.17) | 0.53 (0.11 to 1.00) | -3.36 (-3.59 to -3.13) |
| Lesotho | 372 (289 to 480) | 57.53 (44.34 to 75.18) | 654 (484 to 855) | 89.61 (69.29 to 113.96) | 0.76 (0.24 to 1.53) | 2.39 (1.91 to 2.86) |
| Liberia | 699 (569 to 849) | 78.66 (65.55 to 93.74) | 1067 (798 to 1404) | 75.42 (57.52 to 97.97) | 0.53 (0.16 to 1.03) | -0.2 (-0.29 to -0.11) |
| Libya | 845 (653 to 1135) | 52.26 (40.22 to 70.44) | 2355 (1727 to 3163) | 57.48 (42.25 to 76.66) | 1.79 (1.10 to 2.68) | 0.87 (0.65 to 1.09) |
| Lithuania | 3411 (3182 to 3612) | 76.26 (71.02 to 80.79) | 3783 (3338 to 4184) | 53.88 (47.72 to 59.62) | 0.11 (-0.02 to 0.22) | -1.39 (-1.7 to -1.07) |
| Luxembourg | 491 (458 to 515) | 93.63 (86.47 to 98.35) | 203 (173 to 225) | 15.74 (13.48 to 17.46) | -0.59 (-0.63 to -0.54) | -5.53 (-5.65 to -5.41) |
| Madagascar | 2749 (2229 to 3297) | 76.74 (61.94 to 91.55) | 4490 (3209 to 5882) | 71.49 (51.6 to 91.83) | 0.63 (0.21 to 1.14) | -0.32 (-0.38 to -0.26) |
| Malawi | 1464 (1153 to 1787) | 59.74 (48.13 to 71.92) | 3219 (2532 to 4042) | 66.66 (52.75 to 82.55) | 1.20 (0.75 to 1.83) | 0.17 (-0.03 to 0.37) |
| Malaysia | 4062 (3553 to 4604) | 52.28 (45.59 to 59.38) | 9487 (8311 to 10673) | 42.51 (36.65 to 48.45) | 1.34 (0.99 to 1.73) | -0.44 (-0.62 to -0.26) |
| Maldives | 43 (37 to 49) | 79.63 (66.76 to 92.59) | 101 (83 to 118) | 40.15 (33.13 to 46.86) | 1.37 (0.84 to 1.92) | -2.51 (-2.6 to -2.42) |
| Mali | 1505 (1102 to 2084) | 58.24 (43.2 to 79.74) | 3249 (2413 to 4520) | 54.78 (42.1 to 75.47) | 1.16 (0.66 to 1.81) | 0.02 (-0.11 to 0.14) |
| Malta | 235 (214 to 250) | 60.99 (55.28 to 65.23) | 179 (146 to 202) | 15.15 (12.37 to 17.02) | -0.24 (-0.33 to -0.15) | -4.53 (-4.8 to -4.26) |
| Marshall Islands | 7 (5 to 9) | 62.74 (48.63 to 81.04) | 10 (7 to 13) | 54.2 (41.93 to 70.69) | 0.46 (0.12 to 0.91) | -0.54 (-0.6 to -0.48) |
| Mauritania | 696 (525 to 916) | 89.28 (67.93 to 117.46) | 1199 (873 to 1680) | 72.64 (53.38 to 101.22) | 0.72 (0.32 to 1.27) | -0.81 (-0.98 to -0.64) |
| Mauritius | 575 (543 to 607) | 100.89 (93.71 to 106.86) | 586 (534 to 622) | 35.76 (32.52 to 38.09) | 0.02 (-0.05 to 0.10) | -4.47 (-4.97 to -3.98) |
| Mexico | 13022 (12404 to 13383) | 41.55 (39.26 to 42.8) | 19544 (17332 to 21720) | 17.7 (15.74 to 19.62) | 0.50 (0.36 to 0.65) | -2.75 (-2.9 to -2.61) |
| Micronesia (Federated States of) | 24 (18 to 30) | 65.73 (50.73 to 84.2) | 24 (19 to 33) | 54.83 (43.18 to 71.04) | 0.04 (-0.20 to 0.36) | -0.67 (-0.72 to -0.61) |
| Monaco | 68 (52 to 81) | 77.85 (59.34 to 93.27) | 42 (32 to 52) | 31.42 (24.08 to 38.53) | -0.38 (-0.53 to -0.15) | -3.07 (-3.26 to -2.87) |
| Mongolia | 195 (150 to 251) | 23.28 (17.62 to 30.05) | 372 (268 to 486) | 23.59 (16.92 to 30.69) | 0.91 (0.28 to 1.74) | -0.05 (-0.45 to 0.36) |
| Montenegro | 214 (173 to 256) | 38.99 (31.43 to 46.65) | 520 (411 to 638) | 64.34 (51.78 to 78.49) | 1.43 (0.80 to 2.22) | 2.01 (1.73 to 2.3) |
| Morocco | 11537 (8800 to 14943) | 96.37 (72.9 to 124.38) | 25825 (20230 to 32471) | 93.19 (73.39 to 115.99) | 1.24 (0.75 to 1.77) | 0.04 (-0.01 to 0.09) |
| Mozambique | 3010 (2531 to 3646) | 73.3 (61.47 to 86.9) | 6743 (4988 to 8656) | 89.19 (65.65 to 113.55) | 1.24 (0.63 to 1.91) | 1.06 (0.91 to 1.21) |
| Myanmar | 16549 (12834 to 20759) | 99.65 (79.82 to 120.54) | 27392 (21971 to 34317) | 73.67 (58.93 to 92.23) | 0.66 (0.24 to 1.26) | -1.15 (-1.23 to -1.08) |
| Namibia | 357 (293 to 422) | 88.57 (72.1 to 104.15) | 735 (567 to 906) | 80.22 (62.73 to 97.6) | 1.06 (0.60 to 1.62) | -0.52 (-0.77 to -0.27) |
| Nauru | 2 (2 to 3) | 88.56 (67.54 to 108.75) | 3 (2 to 4) | 79.11 (63.08 to 97.41) | 0.22 (-0.07 to 0.65) | -0.48 (-0.74 to -0.23) |
| Nepal | 3478 (2392 to 4635) | 54.34 (38.15 to 71.14) | 7429 (5610 to 10154) | 41.82 (32 to 56.1) | 1.14 (0.59 to 1.91) | -0.88 (-1.05 to -0.71) |
| Netherlands | 9714 (8538 to 10443) | 47.09 (41.12 to 50.8) | 8584 (7092 to 9551) | 20.64 (17.11 to 22.94) | -0.12 (-0.19 to -0.05) | -3.19 (-3.48 to -2.89) |
| New Zealand | 1702 (1508 to 1827) | 45.42 (39.81 to 48.81) | 1832 (1501 to 2026) | 18.87 (15.52 to 20.85) | 0.08 (-0.03 to 0.17) | -3.08 (-3.22 to -2.94) |
| Nicaragua | 365 (321 to 413) | 29.72 (25.9 to 33.78) | 683 (564 to 840) | 17.08 (14.06 to 20.78) | 0.87 (0.53 to 1.28) | -1.62 (-1.81 to -1.43) |
| Niger | 1015 (673 to 1473) | 58.97 (41.29 to 84.93) | 3030 (2138 to 4357) | 59.23 (43.19 to 82.57) | 1.98 (1.31 to 2.99) | 0.16 (0.11 to 0.2) |
| Nigeria | 25918 (18599 to 35714) | 78.01 (56.93 to 105.99) | 38019 (30052 to 48057) | 60.25 (48.69 to 73.65) | 0.47 (0.07 to 1.10) | -0.94 (-1.06 to -0.82) |
| Niue | 1 (1 to 2) | 58.36 (48.58 to 69.51) | 1 (1 to 1) | 47.72 (39.54 to 56.53) | -0.36 (-0.48 to -0.19) | -0.88 (-0.94 to -0.81) |
| North Macedonia | 2956 (2666 to 3234) | 194.33 (175.05 to 212.61) | 4879 (4031 to 5734) | 216.89 (184.01 to 249.37) | 0.65 (0.34 to 0.98) | 0.01 (-0.52 to 0.54) |
| Northern Mariana Islands | 4 (3 to 5) | 50.5 (41.78 to 61.61) | 10 (9 to 12) | 33.77 (28.35 to 38.79) | 1.58 (1.01 to 2.32) | -1.77 (-2.05 to -1.49) |
| Norway | 4418 (3941 to 4688) | 55.58 (49.55 to 59.03) | 1847 (1522 to 2027) | 14.37 (12.01 to 15.72) | -0.58 (-0.62 to -0.56) | -4.57 (-4.75 to -4.39) |
| Oman | 405 (308 to 513) | 78.06 (59.61 to 97.75) | 674 (546 to 804) | 55.8 (45.54 to 66.2) | 0.67 (0.22 to 1.35) | -0.49 (-0.84 to -0.13) |
| Pakistan | 20946 (15561 to 27573) | 47.17 (35.1 to 61.56) | 41149 (32090 to 52004) | 48.35 (37.72 to 60.06) | 0.96 (0.59 to 1.52) | -0.19 (-0.34 to -0.04) |
| Palau | 5 (4 to 6) | 65.67 (54.19 to 78.26) | 8 (6 to 9) | 56.75 (47.1 to 68.25) | 0.67 (0.26 to 1.19) | -0.27 (-0.37 to -0.17) |
| Palestine | 965 (796 to 1152) | 143.37 (118.52 to 169.23) | 1436 (1232 to 1634) | 89.12 (76.37 to 101.82) | 0.49 (0.19 to 0.87) | -1.56 (-1.88 to -1.25) |
| Panama | 487 (432 to 522) | 37.13 (32.78 to 39.86) | 1074 (825 to 1274) | 23.04 (17.77 to 27.3) | 1.21 (0.79 to 1.62) | -1.7 (-1.9 to -1.5) |
| Papua New Guinea | 457 (310 to 652) | 47.21 (33.37 to 66.66) | 1193 (876 to 1653) | 42.19 (31.49 to 57.99) | 1.61 (0.93 to 2.62) | -0.43 (-0.54 to -0.33) |
| Paraguay | 1104 (934 to 1237) | 57.74 (48.65 to 64.71) | 1988 (1559 to 2431) | 38.72 (30.43 to 47.29) | 0.80 (0.41 to 1.23) | -0.98 (-1.2 to -0.76) |
| Peru | 2700 (2225 to 3117) | 25.94 (21.39 to 29.94) | 4863 (3742 to 6147) | 14.68 (11.33 to 18.55) | 0.80 (0.36 to 1.30) | -2.43 (-2.87 to -1.99) |
| Philippines | 10259 (9030 to 11250) | 56.05 (50.23 to 60.9) | 28253 (24470 to 32341) | 45.86 (39.86 to 52.13) | 1.75 (1.36 to 2.23) | -0.41 (-0.52 to -0.3) |
| Poland | 49329 (46452 to 51003) | 125.14 (116.56 to 129.85) | 34630 (30084 to 37881) | 43.41 (37.9 to 47.43) | -0.30 (-0.36 to -0.24) | -3.83 (-3.95 to -3.71) |
| Portugal | 19703 (18321 to 20545) | 164.16 (150.23 to 171.87) | 9540 (7857 to 10497) | 27.95 (23.31 to 30.63) | -0.52 (-0.57 to -0.48) | -6.3 (-6.54 to -6.06) |
| Puerto Rico | 998 (929 to 1052) | 31.34 (29.01 to 32.98) | 911 (725 to 1063) | 9.33 (7.56 to 10.84) | -0.09 (-0.24 to 0.06) | -4.29 (-4.53 to -4.05) |
| Qatar | 38 (32 to 45) | 86.88 (73.81 to 101.47) | 113 (88 to 142) | 35.06 (27.98 to 42.46) | 1.95 (1.19 to 2.91) | -3.58 (-4.36 to -2.8) |
| Republic of Korea | 21499 (19240 to 23798) | 117.32 (103.27 to 129.72) | 23232 (18526 to 27079) | 25.68 (20.39 to 29.97) | 0.08 (-0.12 to 0.29) | -5.56 (-5.8 to -5.31) |
| Republic of Moldova | 2749 (2491 to 2984) | 87.03 (78.62 to 94.1) | 3133 (2830 to 3459) | 51.45 (46.56 to 56.79) | 0.14 (0.01 to 0.31) | -1.47 (-1.9 to -1.04) |
| Romania | 34141 (31743 to 36185) | 160.1 (147.95 to 169.54) | 38210 (33943 to 42349) | 89.5 (79.5 to 99.37) | 0.12 (-0.01 to 0.26) | -2.3 (-2.53 to -2.07) |
| Russian Federation | 280039 (265350 to 286551) | 185.67 (174.06 to 191) | 240445 (218161 to 258644) | 99.09 (89.81 to 106.59) | -0.14 (-0.20 to -0.09) | -2.88 (-3.41 to -2.35) |
| Rwanda | 1568 (1233 to 2016) | 86.32 (68.59 to 109.41) | 1874 (1325 to 2504) | 48.5 (34.29 to 64.7) | 0.19 (-0.16 to 0.64) | -2.69 (-3.06 to -2.32) |
| Saint Kitts and Nevis | 48 (43 to 52) | 136.47 (124.26 to 147.33) | 34 (29 to 38) | 73.54 (64.26 to 80.43) | -0.29 (-0.39 to -0.18) | -1.81 (-1.98 to -1.63) |
| Saint Lucia | 81 (76 to 86) | 120.77 (112.26 to 127.35) | 119 (99 to 137) | 53.05 (44.37 to 61.22) | 0.46 (0.23 to 0.70) | -3.5 (-3.94 to -3.06) |
| Saint Vincent and the Grenadines | 49 (45 to 53) | 78.18 (71.1 to 83.87) | 60 (54 to 67) | 49.51 (44.27 to 54.52) | 0.23 (0.10 to 0.38) | -1.33 (-1.58 to -1.08) |
| Samoa | 32 (26 to 39) | 52.48 (42.83 to 64.12) | 47 (39 to 57) | 43.35 (35.87 to 51.89) | 0.50 (0.19 to 0.89) | -0.7 (-0.79 to -0.61) |
| San Marino | 19 (16 to 22) | 49.09 (40.62 to 57.06) | 17 (12 to 23) | 14.7 (10.28 to 20.24) | -0.10 (-0.34 to 0.22) | -3.35 (-3.72 to -2.97) |
| Sao Tome and Principe | 37 (31 to 43) | 67.48 (57.28 to 78.83) | 61 (52 to 74) | 76.75 (65.92 to 91.12) | 0.68 (0.39 to 0.99) | 0.62 (0.52 to 0.72) |
| Saudi Arabia | 4222 (3283 to 5278) | 99.41 (77.79 to 123.17) | 7410 (5956 to 9258) | 68.17 (57.11 to 82.99) | 0.76 (0.31 to 1.39) | -1.45 (-1.59 to -1.31) |
| Senegal | 2096 (1709 to 2601) | 84.34 (69.16 to 102.36) | 4577 (3476 to 6123) | 79.53 (60.88 to 104.74) | 1.18 (0.69 to 1.72) | -0.27 (-0.32 to -0.23) |
| Serbia | 18079 (16178 to 19843) | 236.99 (212.08 to 259.66) | 22405 (18994 to 26072) | 127.06 (107.72 to 148.12) | 0.24 (0.03 to 0.48) | -2.65 (-2.93 to -2.38) |
| Seychelles | 35 (29 to 40) | 62.73 (52.34 to 71.37) | 41 (33 to 47) | 43.4 (35.63 to 49.85) | 0.16 (0.02 to 0.31) | -0.82 (-1.04 to -0.6) |
| Sierra Leone | 1513 (1211 to 1836) | 88.12 (71.06 to 105.56) | 2325 (1787 to 2978) | 81.81 (63.68 to 103.26) | 0.54 (0.20 to 1.01) | -0.04 (-0.2 to 0.12) |
| Singapore | 955 (883 to 1004) | 56.04 (51.12 to 59.17) | 567 (469 to 631) | 6.97 (5.77 to 7.78) | -0.41 (-0.49 to -0.35) | -6.52 (-7.01 to -6.03) |
| Slovakia | 5578 (4965 to 6230) | 98.41 (87.93 to 109.73) | 4682 (3879 to 5563) | 48.73 (40.42 to 57.97) | -0.16 (-0.31 to 0.04) | -2.34 (-2.42 to -2.27) |
| Slovenia | 2036 (1875 to 2163) | 83.81 (76.86 to 89.08) | 1443 (1205 to 1628) | 25.31 (21.3 to 28.5) | -0.29 (-0.38 to -0.22) | -3.84 (-4.06 to -3.62) |
| Solomon Islands | 42 (31 to 57) | 58.25 (44.71 to 77.06) | 112 (81 to 156) | 54.74 (40.85 to 74.16) | 1.68 (1.04 to 2.59) | -0.23 (-0.34 to -0.12) |
| Somalia | 689 (437 to 1046) | 52.48 (34.77 to 78.34) | 1361 (848 to 2070) | 42.45 (27.76 to 61.32) | 0.98 (0.47 to 1.68) | -0.64 (-0.68 to -0.61) |
| South Africa | 7288 (5723 to 8337) | 44.02 (34.43 to 50.63) | 19779 (17764 to 21751) | 57.58 (51.52 to 63.4) | 1.71 (1.41 to 2.31) | 0.95 (0.44 to 1.46) |
| South Sudan | 1003 (733 to 1362) | 52.62 (39.1 to 70.46) | 1067 (765 to 1438) | 44.23 (32.57 to 58.41) | 0.06 (-0.20 to 0.44) | -0.72 (-0.9 to -0.54) |
| Spain | 36782 (32962 to 39167) | 70.2 (62.48 to 74.85) | 19845 (15792 to 22143) | 13.78 (11.15 to 15.24) | -0.46 (-0.52 to -0.42) | -5.23 (-5.43 to -5.02) |
| Sri Lanka | 7243 (6566 to 7929) | 103.75 (94.17 to 113.05) | 15166 (10889 to 19542) | 70.52 (51.23 to 90.17) | 1.09 (0.47 to 1.83) | -0.49 (-0.78 to -0.2) |
| Sudan | 7623 (5553 to 9739) | 104.1 (76.19 to 132.75) | 12090 (8790 to 16044) | 81.26 (60.07 to 106.61) | 0.59 (0.19 to 1.15) | -0.95 (-0.99 to -0.9) |
| Suriname | 125 (111 to 138) | 57.32 (51.19 to 63.66) | 239 (183 to 298) | 42.11 (32.07 to 52.39) | 0.92 (0.45 to 1.47) | -0.92 (-1.18 to -0.66) |
| Sweden | 8224 (7297 to 8743) | 46.94 (41.61 to 49.97) | 4608 (3767 to 5210) | 15.69 (12.88 to 17.68) | -0.44 (-0.50 to -0.39) | -3.65 (-3.91 to -3.39) |
| Switzerland | 5086 (4450 to 5460) | 42.84 (37.5 to 46.03) | 2936 (2297 to 3285) | 11.47 (9.11 to 12.77) | -0.42 (-0.49 to -0.37) | -4.13 (-4.25 to -4.01) |
| Syrian Arab Republic | 3474 (2843 to 4211) | 83.32 (68.9 to 100.52) | 6152 (4810 to 7714) | 67.7 (54.27 to 83.02) | 0.77 (0.31 to 1.45) | -1.14 (-1.34 to -0.94) |
| Taiwan (Province of China) | 6512 (6184 to 6827) | 57.46 (53.42 to 60.7) | 6398 (5400 to 7029) | 14.29 (12.14 to 15.66) | -0.02 (-0.13 to 0.07) | -4.47 (-4.72 to -4.22) |
| Tajikistan | 1990 (1599 to 2351) | 83.72 (67.6 to 98.81) | 2762 (2207 to 3369) | 72.19 (58.25 to 87.6) | 0.39 (0.05 to 0.84) | -0.78 (-1.18 to -0.37) |
| Thailand | 11677 (9553 to 13951) | 44.38 (35.88 to 53) | 28619 (21671 to 35981) | 26.42 (19.94 to 33.24) | 1.45 (0.83 to 2.33) | -2.27 (-2.51 to -2.02) |
| Timor-Leste | 104 (85 to 125) | 63.87 (52.63 to 75.96) | 454 (329 to 599) | 70.52 (51.47 to 92.99) | 3.35 (2.10 to 5.07) | 0.54 (0.38 to 0.7) |
| Togo | 715 (580 to 877) | 83.25 (68.23 to 101.5) | 2009 (1465 to 2611) | 82.94 (62.65 to 107.06) | 1.81 (1.11 to 2.61) | -0.12 (-0.26 to 0.02) |
| Tokelau | 1 (1 to 1) | 62.43 (49.74 to 75.97) | 1 (1 to 1) | 43.03 (34.37 to 53.35) | -0.13 (-0.33 to 0.13) | -1.33 (-1.38 to -1.27) |
| Tonga | 13 (10 to 16) | 31.75 (25.2 to 38.46) | 22 (18 to 27) | 30.11 (23.83 to 36.64) | 0.71 (0.31 to 1.27) | 0 (-0.13 to 0.13) |
| Trinidad and Tobago | 564 (526 to 592) | 86.06 (80.79 to 90.21) | 773 (615 to 949) | 42.79 (34.15 to 52.34) | 0.37 (0.11 to 0.69) | -2.46 (-2.69 to -2.22) |
| Tunisia | 2840 (2244 to 3551) | 80.81 (64.39 to 99.89) | 6787 (4808 to 9147) | 60.14 (42.73 to 80.9) | 1.39 (0.72 to 2.21) | -1.15 (-1.27 to -1.03) |
| Türkiye | 1197 (1003 to 1369) | 76.01 (64.52 to 86.13) | 2920 (2247 to 3687) | 89.6 (70.08 to 111.28) | 0.71 (0.34 to 1.13) | 0.15 (-0.33 to 0.64) |
| Turkmenistan | 3 (2 to 4) | 70.68 (57.56 to 86.66) | 4 (4 to 5) | 55.59 (46.22 to 66.19) | 1.44 (0.95 to 2.03) | -0.82 (-0.86 to -0.77) |
| Tuvalu | 22045 (18758 to 25693) | 83.12 (70.1 to 96.48) | 37640 (30897 to 44916) | 47.22 (38.63 to 56.1) | 0.43 (0.16 to 0.79) | -1.59 (-1.96 to -1.22) |
| Uganda | 2288 (1704 to 3060) | 51.66 (38.35 to 68.72) | 3792 (2911 to 4957) | 39.22 (30.54 to 51.52) | 0.66 (0.22 to 1.29) | -1.49 (-1.76 to -1.23) |
| Ukraine | 98337 (93099 to 102439) | 154.39 (144.51 to 161.04) | 64409 (50727 to 79552) | 80.12 (63.2 to 99.3) | -0.35 (-0.48 to -0.19) | -2.86 (-3.18 to -2.54) |
| United Arab Emirates | 230 (180 to 290) | 89.42 (71.54 to 109.14) | 645 (494 to 790) | 62.67 (50.8 to 75.29) | 1.81 (1.21 to 2.74) | 0.84 (0.19 to 1.5) |
| United Kingdom | 59613 (54333 to 62109) | 61.22 (55.46 to 64.01) | 25142 (21088 to 27152) | 15.25 (12.9 to 16.43) | -0.58 (-0.61 to -0.56) | -4.72 (-4.95 to -4.48) |
| United Republic of Tanzania | 3036 (2350 to 4077) | 41.82 (32.53 to 56.08) | 9495 (7010 to 12617) | 52.82 (39.48 to 69.01) | 2.13 (1.18 to 3.54) | 0.75 (0.56 to 0.94) |
| United States of America | 99529 (86392 to 106294) | 29.2 (25.28 to 31.21) | 115555 (94590 to 126652) | 17.33 (14.33 to 18.92) | 0.16 (0.09 to 0.20) | -2.24 (-2.56 to -1.93) |
| United States Virgin Islands | 25 (20 to 29) | 44.23 (37.01 to 51.51) | 34 (27 to 41) | 19.75 (15.91 to 23.88) | 0.36 (0.04 to 0.74) | -2.39 (-2.54 to -2.24) |
| Uruguay | 2863 (2636 to 3028) | 74.8 (68.32 to 79.33) | 2317 (2020 to 2502) | 33.04 (29.17 to 35.54) | -0.19 (-0.25 to -0.13) | -2.77 (-2.98 to -2.57) |
| Uzbekistan | 6344 (5766 to 6902) | 60.23 (54.62 to 65.42) | 11872 (10308 to 13733) | 61.25 (53.52 to 70.2) | 0.87 (0.58 to 1.23) | -0.62 (-1.01 to -0.24) |
| Vanuatu | 24 (18 to 31) | 66.59 (51.33 to 83) | 57 (43 to 71) | 54.27 (41.18 to 66.49) | 1.36 (0.81 to 2.00) | -0.8 (-0.85 to -0.74) |
| Venezuela (Bolivarian Republic of) | 2585 (2323 to 2801) | 33.35 (29.9 to 36.19) | 7299 (5579 to 9246) | 27.73 (21.33 to 34.86) | 1.82 (1.20 to 2.75) | -0.79 (-1.08 to -0.5) |
| Viet Nam | 29987 (24329 to 37796) | 90.06 (73.32 to 113.23) | 74355 (59508 to 88026) | 95.91 (77.51 to 112.18) | 1.48 (0.86 to 2.13) | 0.47 (0.34 to 0.61) |
| Yemen | 3953 (2893 to 5375) | 114.88 (84.35 to 154.87) | 10868 (7885 to 14995) | 107.9 (79.6 to 147.26) | 1.75 (1.05 to 2.73) | -0.4 (-0.48 to -0.31) |
| Zambia | 1001 (732 to 1397) | 55.36 (40.8 to 76.07) | 2741 (2016 to 3662) | 64.87 (48.27 to 85.09) | 1.74 (1.01 to 2.70) | 0.45 (0.34 to 0.55) |
| Zimbabwe | 1358 (1115 to 1627) | 52.87 (43.67 to 62.6) | 2992 (2425 to 3687) | 70.95 (58.22 to 85.21) | 1.20 (0.70 to 1.93) | 1.62 (1.11 to 2.14) |
