# Supplementary material for: Global, regional, and national epidemiology of ischemic stroke from 1990 to 2021
Source: Eur J Neurol. 2024 Sep 17;31(12):e16481. doi: 10.1111/ene.16481 (PMC11555022; doi:10.1111/ene.16481)
Supplement: Supplementary file 7 — TABLE S6. DALYs of ischemic stroke at the national level. ASDR, age‐standardized DALY rate; DALY, disability‐adjusted life year; EAPC, estimated annual percentage change. [file ENE-31-e16481-s001.docx]

Supplementary Table 6. DALYs of Ischemic Stroke at the national level. DALYs = Disability-Adjusted Life Years. ASDR = Age-standardized DALYs rate. EAPC = estimated annual percentage change.

| **Location** | **1990** | | **2021** | | **1990-2021** | |
| --- | --- | --- | --- | --- | --- | --- |
|  | **DALYs Cases** | **ASDR** | **DALYs Cases** | **ASDR** | **Cases change** | **EAPC** |
| Afghanistan | 143544 (103706 to 197915) | 2251.07 (1647.82 to 3057.17) | 187324 (140596 to 255648) | 2044 (1556.96 to 2733.07) | 0.30 (-0.02 to 0.69) | -0.42 (-0.58 to -0.26) |
| Albania | 16198 (13559 to 19329) | 915.33 (770.1 to 1092.34) | 29324 (22395 to 37641) | 694.75 (537.34 to 886.15) | 0.81 (0.33 to 1.45) | -0.53 (-0.77 to -0.28) |
| Algeria | 174568 (140486 to 211750) | 1828.68 (1475.25 to 2207.33) | 364823 (284814 to 458548) | 1261.07 (998.99 to 1552.31) | 1.09 (0.66 to 1.71) | -1.12 (-1.15 to -1.08) |
| American Samoa | 169 (149 to 189) | 913.97 (811.78 to 1015.45) | 289 (247 to 333) | 704.37 (605.31 to 812.05) | 0.71 (0.45 to 1.07) | -1.06 (-1.19 to -0.93) |
| Andorra | 238 (184 to 303) | 474.03 (368.07 to 596.54) | 397 (310 to 497) | 239.01 (187.95 to 296.78) | 0.67 (0.24 to 1.19) | -2.08 (-2.27 to -1.89) |
| Angola | 41152 (33586 to 50593) | 1273.66 (1036.48 to 1561.79) | 105410 (84803 to 128113) | 1172.42 (958.92 to 1428.14) | 1.56 (0.96 to 2.27) | -0.49 (-0.57 to -0.41) |
| Antigua and Barbuda | 540 (499 to 576) | 927 (858.17 to 988.58) | 552 (515 to 593) | 587.51 (549.19 to 628.77) | 0.02 (-0.05 to 0.11) | -1.7 (-1.92 to -1.48) |
| Argentina | 284463 (262183 to 306682) | 934.07 (854.12 to 1004.98) | 214952 (197251 to 233692) | 371.44 (340.4 to 403.75) | -0.24 (-0.30 to -0.18) | -2.72 (-2.9 to -2.54) |
| Armenia | 34230 (31529 to 36776) | 1401.65 (1292.85 to 1501.21) | 41205 (37054 to 45943) | 957.26 (861.41 to 1066.22) | 0.20 (0.08 to 0.36) | -2.13 (-2.44 to -1.8) |
| Australia | 134423 (123792 to 143942) | 710.89 (652.37 to 762.85) | 121464 (105324 to 135376) | 239.76 (209.12 to 267.98) | -0.10 (-0.16 to -0.03) | -3.73 (-3.87 to -3.59) |
| Austria | 129199 (119470 to 136336) | 1024.12 (945.35 to 1080.68) | 60500 (52694 to 68087) | 290.7 (252.46 to 328.94) | -0.53 (-0.57 to -0.49) | -4.25 (-4.56 to -3.94) |
| Azerbaijan | 42679 (34925 to 51631) | 928.2 (761.22 to 1122.3) | 67046 (53651 to 82737) | 774.55 (623.6 to 951.07) | 0.57 (0.16 to 1.10) | -0.43 (-0.67 to -0.19) |
| Bahamas | 1026 (907 to 1136) | 717.7 (637.67 to 791.74) | 1756 (1496 to 2071) | 490.71 (419.93 to 574.79) | 0.71 (0.41 to 1.09) | -1.24 (-1.36 to -1.12) |
| Bahrain | 2015 (1795 to 2213) | 1578.81 (1409.32 to 1743.75) | 5170 (4380 to 6046) | 884.31 (764.08 to 1022.02) | 1.57 (1.10 to 2.08) | -2.39 (-2.8 to -1.99) |
| Bangladesh | 578132 (469540 to 746048) | 1351.92 (1082.65 to 1719.48) | 1402963 (1086243 to 1841188) | 1155.78 (905.17 to 1492.75) | 1.43 (0.87 to 2.14) | -0.54 (-0.79 to -0.29) |
| Barbados | 3458 (3196 to 3676) | 1101.81 (1019.68 to 1167.38) | 3545 (2900 to 4235) | 681.88 (559.66 to 812.16) | 0.03 (-0.16 to 0.24) | -1.74 (-1.95 to -1.54) |
| Belarus | 253688 (233403 to 270306) | 1987.3 (1825.11 to 2119.29) | 226406 (192635 to 263057) | 1386.51 (1178.06 to 1609.28) | -0.11 (-0.24 to 0.04) | -1.88 (-2.31 to -1.45) |
| Belgium | 138018 (126583 to 146435) | 865.78 (792.33 to 919.04) | 74750 (64855 to 82816) | 272.73 (240.6 to 301.08) | -0.46 (-0.50 to -0.42) | -3.65 (-3.81 to -3.48) |
| Belize | 474 (431 to 512) | 504.7 (457.12 to 545.53) | 1156 (1025 to 1285) | 434.42 (383.59 to 484.05) | 1.44 (1.17 to 1.77) | -0.85 (-1.34 to -0.35) |
| Benin | 29831 (24710 to 36024) | 1578.63 (1316.85 to 1913.43) | 61150 (50505 to 75402) | 1358.53 (1132.66 to 1655.96) | 1.05 (0.69 to 1.52) | -0.45 (-0.55 to -0.36) |
| Bermuda | 478 (433 to 522) | 823.7 (748.2 to 897.37) | 499 (431 to 592) | 337.66 (293.13 to 399.64) | 0.04 (-0.11 to 0.28) | -2.97 (-3.18 to -2.76) |
| Bhutan | 1701 (1239 to 2222) | 833.67 (573.61 to 1107.15) | 3804 (3003 to 4604) | 673.28 (531.09 to 814.3) | 1.24 (0.75 to 1.86) | -0.74 (-0.81 to -0.67) |
| Bolivia (Plurinational State of) | 26367 (19320 to 35084) | 880.24 (652.06 to 1134.26) | 40844 (29094 to 55794) | 510.27 (366.86 to 690.34) | 0.55 (0.20 to 1.08) | -1.77 (-1.9 to -1.63) |
| Bosnia and Herzegovina | 77957 (67477 to 88953) | 2212.88 (1947.07 to 2497.59) | 100394 (82555 to 116938) | 1571.69 (1292.29 to 1836.11) | 0.29 (0.03 to 0.60) | -1.38 (-1.51 to -1.25) |
| Botswana | 6626 (5202 to 8147) | 1507.04 (1181.8 to 1824.47) | 12403 (10368 to 15136) | 1069.67 (901.44 to 1301.7) | 0.87 (0.47 to 1.47) | -1.06 (-1.26 to -0.87) |
| Brazil | 1076191 (1018097 to 1117071) | 1379.82 (1282.44 to 1438.61) | 1258850 (1149139 to 1335368) | 518.03 (471.02 to 550.05) | 0.17 (0.11 to 0.22) | -3.09 (-3.22 to -2.97) |
| Brunei Darussalam | 1248 (1084 to 1440) | 1433.03 (1251.72 to 1636.36) | 1753 (1528 to 2002) | 683.96 (590.67 to 778.98) | 0.40 (0.18 to 0.66) | -2.24 (-2.45 to -2.03) |
| Bulgaria | 295255 (282045 to 309336) | 2833.71 (2709.85 to 2952.67) | 352137 (310468 to 397947) | 2383.57 (2105.14 to 2689.81) | 0.19 (0.05 to 0.35) | -0.39 (-0.54 to -0.23) |
| Burkina Faso | 33969 (26600 to 44132) | 883.28 (692.66 to 1145.41) | 71547 (56989 to 91531) | 867.73 (695.14 to 1103.1) | 1.11 (0.69 to 1.65) | 0.17 (0.07 to 0.26) |
| Burundi | 33958 (25541 to 44801) | 1599.71 (1188.91 to 2079.85) | 37840 (29089 to 48885) | 962.41 (729.7 to 1259.97) | 0.11 (-0.15 to 0.47) | -2.14 (-2.37 to -1.92) |
| Cabo Verde | 2398 (2008 to 2874) | 982.06 (816.1 to 1187.09) | 5030 (4171 to 5937) | 1195.62 (988.47 to 1408.64) | 1.10 (0.68 to 1.68) | 0.33 (0.03 to 0.63) |
| Cambodia | 51084 (42542 to 61440) | 1369.4 (1149.6 to 1641.97) | 122669 (99132 to 146213) | 1249.17 (1004.51 to 1478.07) | 1.40 (0.87 to 1.94) | -0.41 (-0.51 to -0.32) |
| Cameroon | 45828 (35894 to 59057) | 1187.61 (931.55 to 1494.27) | 142484 (108965 to 189763) | 1309.57 (1014.34 to 1735.18) | 2.11 (1.42 to 3.09) | 0.38 (-0.02 to 0.78) |
| Canada | 186887 (171447 to 201161) | 583.9 (533.44 to 628.67) | 206238 (180736 to 231056) | 270.35 (235.02 to 306.54) | 0.10 (0.03 to 0.17) | -2.8 (-2.97 to -2.63) |
| Central African Republic | 13110 (9499 to 17193) | 1460.38 (1075.27 to 1916.61) | 20563 (14922 to 28243) | 1292.86 (949.9 to 1768.66) | 0.57 (0.21 to 0.96) | -0.47 (-0.54 to -0.41) |
| Chad | 35730 (27783 to 48364) | 1295.96 (1004.85 to 1760.83) | 78401 (61185 to 101443) | 1467.28 (1146.02 to 1912.11) | 1.19 (0.73 to 1.82) | 0.35 (0.18 to 0.52) |
| Chile | 90987 (85852 to 95750) | 983.73 (924.54 to 1035.74) | 102550 (92831 to 112233) | 394.06 (356.79 to 432.28) | 0.13 (0.05 to 0.20) | -2.58 (-2.74 to -2.41) |
| China | 9926125 (8510100 to 11656218) | 1387.93 (1188.74 to 1621.4) | 23430411 (19918946 to 26933909) | 1180.98 (1009.7 to 1356.67) | 1.36 (0.90 to 1.91) | -0.5 (-0.69 to -0.32) |
| Colombia | 111122 (104909 to 116858) | 692.82 (651.25 to 728.34) | 150357 (129215 to 173444) | 272.11 (234.31 to 313.71) | 0.35 (0.17 to 0.56) | -3.51 (-3.78 to -3.24) |
| Comoros | 2107 (1627 to 2699) | 1262.94 (990.31 to 1571.91) | 3796 (3015 to 4786) | 905.36 (721.28 to 1134.08) | 0.80 (0.38 to 1.37) | -1.34 (-1.52 to -1.17) |
| Congo | 13714 (10842 to 16974) | 1567.62 (1247.72 to 1897.52) | 26289 (20866 to 31739) | 1272.03 (1008.89 to 1532.14) | 0.92 (0.51 to 1.38) | -0.91 (-1.02 to -0.81) |
| Cook Islands | 94 (81 to 110) | 850.76 (736.93 to 995.78) | 131 (107 to 155) | 524.64 (429.65 to 618.94) | 0.40 (0.14 to 0.69) | -1.64 (-1.78 to -1.5) |
| Costa Rica | 7676 (7043 to 8232) | 456.67 (418.41 to 489.99) | 15108 (13261 to 16627) | 274.4 (242.69 to 301.57) | 0.97 (0.76 to 1.15) | -2.11 (-2.46 to -1.75) |
| Croatia | 117620 (110993 to 124313) | 2217.62 (2090.98 to 2339.36) | 77608 (69180 to 86416) | 781.51 (698 to 867.03) | -0.34 (-0.40 to -0.27) | -3.52 (-3.65 to -3.4) |
| Cuba | 71929 (66977 to 76612) | 724.26 (674.63 to 770.26) | 119030 (105556 to 132769) | 584.52 (519.55 to 653.19) | 0.65 (0.45 to 0.88) | -0.67 (-0.77 to -0.57) |
| Cyprus | 8374 (7174 to 9641) | 1577.38 (1347.88 to 1811.29) | 7164 (6024 to 8303) | 414.76 (350.18 to 480.1) | -0.14 (-0.32 to 0.05) | -4.62 (-4.89 to -4.35) |
| Czechia | 353886 (333415 to 374684) | 2561.6 (2408.29 to 2713.53) | 129117 (114221 to 143143) | 565.82 (502.23 to 629.15) | -0.64 (-0.67 to -0.59) | -5.22 (-5.53 to -4.91) |
| Côte d'Ivoire | 57671 (46744 to 72014) | 1609.14 (1322.33 to 1916.03) | 144264 (110164 to 188375) | 1463.7 (1151.38 to 1853.1) | 1.50 (0.96 to 2.27) | -0.41 (-0.58 to -0.23) |
| Democratic People's Republic of Korea | 198405 (157579 to 250799) | 1412.4 (1130.98 to 1762.26) | 429296 (341946 to 548679) | 1358.81 (1088.57 to 1728.93) | 1.16 (0.71 to 1.75) | -0.15 (-0.31 to 0) |
| Democratic Republic of the Congo | 149154 (111941 to 187131) | 1162.36 (882.73 to 1517.32) | 281741 (206383 to 389246) | 1021.24 (726.4 to 1437.1) | 0.89 (0.43 to 1.52) | -0.52 (-0.57 to -0.48) |
| Denmark | 71525 (66168 to 76068) | 816.94 (759.74 to 869.88) | 41702 (36920 to 45444) | 313.59 (279.04 to 343.32) | -0.42 (-0.46 to -0.38) | -3.41 (-3.59 to -3.24) |
| Djibouti | 1101 (837 to 1523) | 1068.92 (821.31 to 1459.38) | 4947 (3805 to 6550) | 1022.11 (785.92 to 1332.7) | 3.49 (2.37 to 5.00) | -0.26 (-0.31 to -0.22) |
| Dominica | 606 (543 to 673) | 1021.79 (918.96 to 1130.54) | 646 (565 to 743) | 835.78 (735.33 to 957.1) | 0.07 (-0.08 to 0.26) | -0.69 (-0.78 to -0.59) |
| Dominican Republic | 23409 (20358 to 26526) | 696.62 (603.27 to 787.73) | 62109 (49573 to 77698) | 636.5 (507.08 to 797.25) | 1.65 (1.08 to 2.45) | 0.07 (-0.11 to 0.25) |
| Ecuador | 33682 (30063 to 36386) | 658.24 (592.25 to 709.09) | 50780 (42637 to 59967) | 327.98 (276.69 to 385.93) | 0.51 (0.26 to 0.79) | -2.14 (-2.4 to -1.88) |
| Egypt | 705127 (509645 to 966375) | 3107.34 (2358.75 to 4097.47) | 1266972 (984970 to 1597188) | 2462.6 (1969.06 to 3002.37) | 0.80 (0.36 to 1.35) | -0.35 (-0.51 to -0.19) |
| El Salvador | 14035 (12476 to 15625) | 473.15 (420.35 to 528.57) | 18912 (15666 to 22329) | 290.05 (239.86 to 342.52) | 0.35 (0.08 to 0.63) | -1.79 (-2.09 to -1.48) |
| Equatorial Guinea | 2207 (1675 to 2859) | 1365.36 (1048.38 to 1754.28) | 4281 (3089 to 5789) | 1045.28 (761.61 to 1399.72) | 0.94 (0.32 to 1.77) | -1.13 (-1.33 to -0.93) |
| Eritrea | 9728 (6980 to 13599) | 1195.04 (865.33 to 1644.81) | 20981 (16072 to 27166) | 1016.4 (771.39 to 1293.34) | 1.16 (0.67 to 1.82) | -0.58 (-0.64 to -0.52) |
| Estonia | 48300 (45570 to 50921) | 2372.94 (2233.67 to 2496.56) | 14986 (13200 to 16732) | 514.34 (453.8 to 575.43) | -0.69 (-0.73 to -0.66) | -6.3 (-6.84 to -5.77) |
| Eswatini | 2901 (2302 to 3631) | 1286.33 (1023.56 to 1575.79) | 5825 (4257 to 7901) | 1339.34 (1001.75 to 1753.76) | 1.01 (0.48 to 1.75) | 0.52 (0.12 to 0.91) |
| Ethiopia | 112893 (80325 to 166729) | 691.54 (499.2 to 980.76) | 195828 (158179 to 247585) | 515.13 (412.03 to 653.85) | 0.73 (0.13 to 1.26) | -1.22 (-1.31 to -1.12) |
| Fiji | 2942 (2532 to 3438) | 959.89 (820.89 to 1116.6) | 5262 (4286 to 6298) | 848.77 (701.14 to 1011.35) | 0.79 (0.48 to 1.23) | -0.7 (-0.86 to -0.54) |
| Finland | 75211 (68725 to 80426) | 1029.37 (939.53 to 1102.42) | 52985 (46211 to 58598) | 357.84 (314.38 to 397.54) | -0.30 (-0.35 to -0.24) | -3.38 (-3.5 to -3.27) |
| France | 584519 (529676 to 619950) | 651.15 (591.78 to 690.57) | 425563 (370417 to 471781) | 244.49 (214.52 to 270.49) | -0.27 (-0.32 to -0.22) | -3.08 (-3.2 to -2.95) |
| Gabon | 6086 (4936 to 7461) | 1196.96 (970.33 to 1450.33) | 8876 (6884 to 11022) | 1066.86 (830.73 to 1318.52) | 0.46 (0.17 to 0.82) | -0.49 (-0.61 to -0.36) |
| Gambia | 5101 (3959 to 6568) | 1562.04 (1207.95 to 1995.78) | 14913 (11386 to 19175) | 1677.54 (1279.58 to 2144.4) | 1.92 (1.19 to 2.88) | 0.19 (0.09 to 0.29) |
| Georgia | 79352 (65482 to 90903) | 1358.42 (1127.93 to 1549.08) | 103988 (93106 to 114540) | 1653.14 (1472.8 to 1821.49) | 0.31 (0.11 to 0.62) | 0.61 (0.11 to 1.12) |
| Germany | 1470729 (1334668 to 1565305) | 1093.62 (991.66 to 1165.15) | 797270 (701814 to 883712) | 362.86 (322.84 to 402.43) | -0.46 (-0.50 to -0.42) | -3.62 (-3.85 to -3.38) |
| Ghana | 107615 (88498 to 135798) | 1893.8 (1537.04 to 2376.29) | 285112 (226667 to 358224) | 1936.69 (1534.5 to 2411.61) | 1.65 (1.04 to 2.43) | 0.31 (0.12 to 0.51) |
| Greece | 210967 (194877 to 221996) | 1432.33 (1317 to 1509.83) | 141981 (123221 to 154800) | 443.65 (392.59 to 483.95) | -0.33 (-0.37 to -0.29) | -4.45 (-4.75 to -4.15) |
| Greenland | 401 (353 to 450) | 1687.76 (1491.59 to 1885.15) | 319 (269 to 384) | 611.64 (516.48 to 733.1) | -0.20 (-0.33 to -0.04) | -3.51 (-3.65 to -3.36) |
| Grenada | 1261 (1127 to 1381) | 1612.25 (1435.86 to 1768.27) | 817 (729 to 897) | 832.63 (745.76 to 909.91) | -0.35 (-0.44 to -0.26) | -2.08 (-2.26 to -1.89) |
| Guam | 505 (448 to 564) | 837.47 (748.75 to 932.38) | 858 (729 to 996) | 416.48 (353.28 to 484.49) | 0.70 (0.49 to 0.94) | -2.05 (-2.33 to -1.77) |
| Guatemala | 15067 (13376 to 16639) | 520.08 (469.31 to 567.67) | 30378 (26766 to 34395) | 304.01 (269.18 to 342.2) | 1.02 (0.71 to 1.36) | -2.26 (-2.56 to -1.97) |
| Guinea | 42992 (33216 to 54587) | 1340.8 (1026.23 to 1708.22) | 78602 (59721 to 101610) | 1492.23 (1156.19 to 1909.41) | 0.83 (0.39 to 1.45) | 0.62 (0.49 to 0.75) |
| Guinea-Bissau | 7887 (5991 to 10334) | 2116.89 (1641.27 to 2684.76) | 12278 (9557 to 15699) | 1982.17 (1572.41 to 2505.28) | 0.56 (0.20 to 1.02) | -0.07 (-0.12 to -0.01) |
| Guyana | 7359 (6720 to 8142) | 2105.13 (1922.97 to 2320.98) | 6731 (5447 to 8268) | 1213.99 (998.62 to 1477.85) | -0.09 (-0.28 to 0.13) | -1.25 (-1.45 to -1.05) |
| Haiti | 53673 (40968 to 66913) | 1927.17 (1509.39 to 2345.76) | 84370 (60496 to 118305) | 1437.4 (1054.03 to 1973.52) | 0.57 (0.19 to 1.09) | -0.82 (-0.89 to -0.76) |
| Honduras | 14310 (11395 to 17638) | 735.72 (590.55 to 896.37) | 48553 (37815 to 62100) | 887.17 (694.19 to 1123.91) | 2.39 (1.73 to 3.27) | 0.8 (0.61 to 0.98) |
| Hungary | 337458 (320125 to 354229) | 2362.5 (2241.1 to 2481.02) | 165518 (145127 to 185316) | 806.72 (708.21 to 902.1) | -0.51 (-0.57 to -0.45) | -3.84 (-4.03 to -3.65) |
| Iceland | 2045 (1849 to 2196) | 673.2 (610.46 to 721.7) | 1581 (1355 to 1780) | 241.54 (209.83 to 273.72) | -0.23 (-0.29 to -0.16) | -3.41 (-3.55 to -3.27) |
| India | 2888931 (2390413 to 3642144) | 722.08 (597.86 to 903.45) | 6680576 (5770847 to 8536838) | 611.17 (529.23 to 765.99) | 1.31 (0.96 to 1.70) | -0.69 (-0.79 to -0.59) |
| Indonesia | 1173194 (974878 to 1365835) | 1394.23 (1144.13 to 1632.94) | 3339844 (2536606 to 4124779) | 1741.06 (1361.94 to 2109.37) | 1.85 (1.25 to 2.49) | 0.79 (0.65 to 0.93) |
| Iran (Islamic Republic of) | 421889 (389724 to 459410) | 1825.58 (1677.25 to 1988.32) | 683977 (625155 to 745106) | 957.72 (869.79 to 1044.25) | 0.62 (0.46 to 0.79) | -2.2 (-2.31 to -2.09) |
| Iraq | 199205 (168453 to 230315) | 2473.09 (2085.26 to 2855.43) | 443210 (350564 to 534121) | 2163.86 (1728.51 to 2568.46) | 1.22 (0.69 to 1.85) | -0.95 (-1.12 to -0.78) |
| Ireland | 38808 (36353 to 40832) | 952.98 (887.47 to 1002.82) | 18924 (16186 to 20965) | 225.24 (193.18 to 250.1) | -0.51 (-0.56 to -0.47) | -4.77 (-4.98 to -4.57) |
| Israel | 29365 (26855 to 31784) | 622.44 (568.11 to 672.74) | 29592 (25346 to 33191) | 220.98 (189.4 to 248.7) | 0.01 (-0.07 to 0.09) | -3.77 (-3.93 to -3.61) |
| Italy | 859827 (784492 to 909137) | 958.62 (866.53 to 1017.2) | 539857 (447429 to 596628) | 285.83 (244.14 to 315.97) | -0.37 (-0.43 to -0.34) | -4.13 (-4.38 to -3.88) |
| Jamaica | 20279 (18784 to 21992) | 1074.36 (994.11 to 1166.64) | 24038 (19469 to 29433) | 747.03 (603.67 to 912.81) | 0.19 (-0.05 to 0.48) | -1.01 (-1.39 to -0.63) |
| Japan | 1436433 (1312221 to 1539602) | 907.96 (819.32 to 973.83) | 1409376 (1190419 to 1583340) | 307.86 (262.26 to 348.59) | -0.02 (-0.10 to 0.04) | -3.71 (-3.87 to -3.56) |
| Jordan | 25420 (21478 to 29879) | 2049.76 (1743.95 to 2421.73) | 64418 (53929 to 76318) | 1013.71 (842.86 to 1193.24) | 1.53 (0.96 to 2.26) | -2.75 (-3.05 to -2.44) |
| Kazakhstan | 269719 (239032 to 298030) | 2300.84 (2059.55 to 2535.18) | 270137 (235800 to 304369) | 1757.3 (1546.35 to 1964.27) | 0.00 (-0.13 to 0.17) | -1.44 (-1.9 to -0.97) |
| Kenya | 50516 (40278 to 62450) | 701.89 (550.74 to 880.74) | 137041 (110726 to 165865) | 759.48 (599.11 to 929.94) | 1.71 (1.28 to 2.19) | 0.43 (0.34 to 0.52) |
| Kiribati | 387 (327 to 449) | 1130.23 (937.15 to 1324.21) | 690 (568 to 831) | 1121.98 (940.93 to 1327.22) | 0.78 (0.48 to 1.22) | -0.06 (-0.1 to -0.02) |
| Kuwait | 4570 (4173 to 5020) | 753.88 (686.27 to 821.76) | 14657 (12519 to 17060) | 526.33 (444.85 to 612.09) | 2.21 (1.77 to 2.65) | -0.94 (-1.84 to -0.04) |
| Kyrgyzstan | 59532 (53887 to 65051) | 2130.87 (1934.04 to 2328.44) | 58321 (49602 to 66795) | 1274.76 (1095.54 to 1458.2) | -0.02 (-0.17 to 0.16) | -2.17 (-2.52 to -1.82) |
| Lao People's Democratic Republic | 37739 (30544 to 46637) | 2048.2 (1656.27 to 2525.07) | 55537 (43016 to 69493) | 1420.11 (1115.48 to 1751.55) | 0.47 (0.10 to 1.00) | -1.32 (-1.39 to -1.24) |
| Latvia | 89466 (84421 to 94601) | 2504.41 (2362.79 to 2647.56) | 65466 (58444 to 72264) | 1458.59 (1298.88 to 1616.34) | -0.27 (-0.34 to -0.19) | -2.21 (-2.46 to -1.97) |
| Lebanon | 25958 (20949 to 33000) | 1343.46 (1088.61 to 1706.92) | 34130 (29288 to 38742) | 536.13 (461.46 to 610.84) | 0.31 (-0.03 to 0.69) | -3.09 (-3.34 to -2.84) |
| Lesotho | 7307 (5794 to 9143) | 995.7 (791.56 to 1254.1) | 13901 (10484 to 18450) | 1581.93 (1222.1 to 2049.68) | 0.90 (0.36 to 1.68) | 2.3 (1.88 to 2.73) |
| Liberia | 16857 (14025 to 20664) | 1472.46 (1238.03 to 1765.79) | 25079 (19026 to 32952) | 1367.9 (1061.1 to 1754.23) | 0.49 (0.15 to 0.95) | -0.31 (-0.39 to -0.22) |
| Libya | 20085 (15976 to 25597) | 1017.93 (802.31 to 1332.13) | 56023 (42323 to 73821) | 1142.47 (863.13 to 1497.78) | 1.79 (1.12 to 2.60) | 0.82 (0.64 to 1) |
| Lithuania | 65985 (61468 to 70244) | 1466.44 (1364.6 to 1561.54) | 60850 (54067 to 67264) | 969.78 (863.9 to 1072.11) | -0.08 (-0.18 to 0.02) | -1.25 (-1.56 to -0.93) |
| Luxembourg | 7381 (6924 to 7731) | 1354.7 (1268.84 to 1418.94) | 2958 (2603 to 3257) | 251.42 (221.82 to 277.04) | -0.60 (-0.63 to -0.56) | -5.4 (-5.55 to -5.26) |
| Madagascar | 64850 (53795 to 76527) | 1460.03 (1212.49 to 1712.24) | 117563 (89508 to 149247) | 1340.91 (1012.73 to 1714.69) | 0.81 (0.41 to 1.30) | -0.35 (-0.4 to -0.3) |
| Malawi | 36385 (29711 to 44025) | 1115.97 (911.8 to 1321.78) | 74119 (59846 to 91545) | 1209.06 (982.69 to 1480.67) | 1.04 (0.64 to 1.57) | 0.05 (-0.15 to 0.25) |
| Malaysia | 95438 (85152 to 106562) | 1103.51 (983.71 to 1235.82) | 222302 (197108 to 248126) | 862.31 (764.62 to 959.95) | 1.33 (1.05 to 1.63) | -0.65 (-0.76 to -0.53) |
| Maldives | 1154 (1008 to 1311) | 1547.16 (1355.24 to 1764.71) | 2052 (1728 to 2363) | 701.3 (589.06 to 807.15) | 0.78 (0.45 to 1.13) | -2.91 (-3.02 to -2.81) |
| Mali | 37756 (28639 to 51267) | 1086.04 (829.55 to 1465.3) | 79037 (60052 to 106759) | 1006.05 (770.66 to 1361.47) | 1.09 (0.64 to 1.70) | -0.08 (-0.2 to 0.04) |
| Malta | 3958 (3669 to 4197) | 969.61 (895.75 to 1029.04) | 2677 (2300 to 2981) | 245.87 (211.52 to 273.89) | -0.32 (-0.39 to -0.26) | -4.57 (-4.77 to -4.38) |
| Marshall Islands | 171 (137 to 219) | 1194.76 (967.47 to 1514.1) | 283 (219 to 369) | 1047.04 (827.2 to 1329.72) | 0.66 (0.33 to 1.05) | -0.48 (-0.53 to -0.42) |
| Mauritania | 15845 (12428 to 20434) | 1700.46 (1340.46 to 2206.91) | 25194 (19100 to 34546) | 1300.7 (981.01 to 1763.42) | 0.59 (0.25 to 1.03) | -1.02 (-1.18 to -0.86) |
| Mauritius | 13426 (12689 to 14177) | 2004.59 (1893.12 to 2127.16) | 12492 (11539 to 13341) | 723.72 (668.5 to 773.23) | -0.07 (-0.13 to -0.01) | -4.36 (-4.84 to -3.87) |
| Mexico | 257976 (246657 to 268921) | 680.46 (649.86 to 706.95) | 385010 (347021 to 430103) | 325.01 (293.14 to 362.32) | 0.49 (0.36 to 0.64) | -2.46 (-2.59 to -2.32) |
| Micronesia (Federated States of) | 578 (459 to 752) | 1289.07 (1019.64 to 1633.09) | 649 (516 to 830) | 1076.92 (866.14 to 1350.59) | 0.12 (-0.09 to 0.40) | -0.65 (-0.7 to -0.59) |
| Monaco | 962 (759 to 1144) | 1173.11 (929.33 to 1394.43) | 576 (472 to 691) | 492.3 (403.83 to 588.84) | -0.40 (-0.52 to -0.20) | -2.94 (-3.1 to -2.77) |
| Mongolia | 5560 (4553 to 6637) | 539.67 (435.35 to 652.01) | 11492 (9309 to 13966) | 549.66 (436.59 to 677.5) | 1.07 (0.59 to 1.64) | 0.01 (-0.31 to 0.34) |
| Montenegro | 3908 (3302 to 4630) | 673.35 (570.14 to 797.14) | 8382 (6819 to 10309) | 945.49 (772 to 1150.21) | 1.14 (0.63 to 1.78) | 1.33 (1.23 to 1.44) |
| Morocco | 250251 (193436 to 319616) | 1807.59 (1396.62 to 2323.2) | 524384 (408240 to 659417) | 1669.07 (1321.64 to 2087.98) | 1.10 (0.62 to 1.63) | -0.14 (-0.2 to -0.09) |
| Mozambique | 70581 (59832 to 84188) | 1388.11 (1182.33 to 1652.67) | 162429 (120565 to 204982) | 1717.83 (1299.52 to 2153.81) | 1.30 (0.73 to 1.92) | 1.11 (0.95 to 1.27) |
| Myanmar | 392174 (302850 to 495132) | 1909.66 (1498.5 to 2350.47) | 569525 (454554 to 706404) | 1333.73 (1081.53 to 1646.09) | 0.45 (0.12 to 0.93) | -1.32 (-1.4 to -1.24) |
| Namibia | 8031 (6692 to 9372) | 1568.72 (1309.97 to 1832.32) | 15140 (12140 to 18420) | 1386.73 (1104.95 to 1682.41) | 0.89 (0.49 to 1.36) | -0.61 (-0.84 to -0.38) |
| Nauru | 73 (55 to 90) | 1811.83 (1396.02 to 2213.01) | 85 (67 to 106) | 1628.17 (1318.55 to 1972.88) | 0.16 (-0.08 to 0.50) | -0.48 (-0.74 to -0.21) |
| Nepal | 79281 (58058 to 105808) | 995.19 (718.78 to 1303.81) | 149263 (114540 to 204972) | 724.89 (559.95 to 979.19) | 0.88 (0.42 to 1.50) | -1.08 (-1.23 to -0.93) |
| Netherlands | 160933 (146160 to 173070) | 779.6 (707.95 to 840.02) | 129839 (113705 to 142232) | 336.1 (294.77 to 368.88) | -0.19 (-0.25 to -0.15) | -3.17 (-3.42 to -2.93) |
| New Zealand | 28474 (25978 to 30487) | 729.96 (660.85 to 782.78) | 27662 (23868 to 30738) | 304.08 (261.72 to 339.38) | -0.03 (-0.10 to 0.04) | -3.03 (-3.18 to -2.87) |
| Nicaragua | 7764 (6934 to 8651) | 531.08 (469.71 to 596.45) | 13898 (11879 to 16498) | 310.46 (266.01 to 370.98) | 0.79 (0.51 to 1.13) | -1.68 (-1.82 to -1.54) |
| Niger | 27669 (19384 to 39693) | 1110.81 (796.9 to 1552.56) | 72340 (52678 to 100252) | 1045.03 (775.94 to 1440.29) | 1.61 (1.06 to 2.36) | -0.09 (-0.14 to -0.03) |
| Nigeria | 564845 (419295 to 769108) | 1416.71 (1058.67 to 1918.74) | 883707 (706488 to 1104749) | 1097.46 (898.51 to 1349.69) | 0.56 (0.13 to 1.22) | -0.92 (-1.05 to -0.8) |
| Niue | 27 (23 to 32) | 1135.53 (978.1 to 1333.6) | 19 (16 to 23) | 960.96 (811.7 to 1109.74) | -0.28 (-0.40 to -0.13) | -0.79 (-0.86 to -0.72) |
| North Macedonia | 53144 (48360 to 57937) | 3251.15 (2952.09 to 3545.23) | 80990 (66704 to 95496) | 3037.44 (2559.01 to 3507.51) | 0.52 (0.24 to 0.84) | -0.51 (-0.91 to -0.12) |
| Northern Mariana Islands | 125 (103 to 150) | 946.22 (814.21 to 1133.17) | 265 (229 to 299) | 660.04 (572.64 to 743.93) | 1.12 (0.74 to 1.55) | -1.49 (-1.7 to -1.29) |
| Norway | 70072 (64638 to 74785) | 918.3 (844.55 to 981.23) | 32033 (27904 to 35777) | 281.24 (243.31 to 316.54) | -0.54 (-0.57 to -0.52) | -4.06 (-4.16 to -3.96) |
| Oman | 10268 (8067 to 12704) | 1563.84 (1222.75 to 1958.23) | 18108 (15133 to 21167) | 1026.21 (852.71 to 1199.9) | 0.76 (0.33 to 1.38) | -0.87 (-1.1 to -0.65) |
| Pakistan | 461445 (357733 to 588029) | 884.16 (674.23 to 1138.66) | 956691 (779500 to 1193626) | 906.02 (738.53 to 1124.45) | 1.07 (0.71 to 1.59) | -0.18 (-0.32 to -0.03) |
| Palau | 114 (96 to 137) | 1302.81 (1101.78 to 1543.66) | 200 (167 to 238) | 1117.3 (947.12 to 1320.21) | 0.75 (0.39 to 1.21) | -0.36 (-0.43 to -0.29) |
| Palestine | 18962 (15458 to 22737) | 2338.87 (1923.13 to 2806.89) | 29158 (25372 to 33111) | 1423.26 (1235.19 to 1610.32) | 0.54 (0.25 to 0.92) | -1.64 (-1.9 to -1.37) |
| Panama | 8867 (8003 to 9536) | 627.59 (566.25 to 674.31) | 17107 (13802 to 20251) | 378.4 (305.39 to 448.17) | 0.93 (0.57 to 1.27) | -1.81 (-1.99 to -1.63) |
| Papua New Guinea | 12994 (9525 to 17086) | 887.89 (648.65 to 1204.07) | 31503 (25005 to 40327) | 780.11 (605.78 to 1036.05) | 1.42 (0.90 to 2.17) | -0.49 (-0.59 to -0.4) |
| Paraguay | 18945 (16447 to 21340) | 928.38 (801.83 to 1044.16) | 34038 (27270 to 41660) | 634 (509.31 to 775.62) | 0.80 (0.42 to 1.21) | -1.09 (-1.25 to -0.93) |
| Peru | 53658 (46086 to 61370) | 463.28 (393.06 to 532.18) | 91672 (73669 to 112555) | 275.3 (221.18 to 338.34) | 0.71 (0.32 to 1.13) | -2.2 (-2.61 to -1.79) |
| Philippines | 237647 (211199 to 260246) | 965.67 (863.87 to 1051.81) | 671394 (585886 to 758824) | 913.67 (797.64 to 1028.66) | 1.83 (1.46 to 2.26) | -0.02 (-0.11 to 0.07) |
| Poland | 839192 (803734 to 869695) | 2008.95 (1910.01 to 2086.27) | 563558 (506055 to 617924) | 745.11 (672.63 to 817.99) | -0.33 (-0.38 to -0.27) | -3.59 (-3.7 to -3.48) |
| Portugal | 303300 (286121 to 316636) | 2326.9 (2180.79 to 2434.7) | 123071 (106002 to 133863) | 401.95 (353.04 to 437.25) | -0.59 (-0.63 to -0.57) | -6.2 (-6.42 to -5.98) |
| Puerto Rico | 16316 (15353 to 17214) | 476.13 (447.01 to 502.23) | 14711 (12499 to 16895) | 180.9 (154.41 to 208.22) | -0.10 (-0.21 to 0.02) | -3.51 (-3.7 to -3.32) |
| Qatar | 1129 (971 to 1304) | 1458.87 (1255.22 to 1693.32) | 4321 (3482 to 5099) | 592.16 (488.27 to 701.16) | 2.83 (2.10 to 3.75) | -3.33 (-3.98 to -2.68) |
| Republic of Korea | 467894 (420835 to 512530) | 2013.6 (1812.71 to 2204.02) | 434175 (367708 to 495670) | 473.65 (401.6 to 540.18) | -0.07 (-0.20 to 0.07) | -5.29 (-5.52 to -5.05) |
| Republic of Moldova | 52664 (47423 to 57435) | 1418.74 (1286.09 to 1541.33) | 63140 (57401 to 69442) | 1045.92 (950.27 to 1148.48) | 0.20 (0.07 to 0.37) | -0.75 (-1.17 to -0.34) |
| Romania | 605750 (560750 to 646814) | 2492.49 (2311.65 to 2651.92) | 593583 (534628 to 651730) | 1455.3 (1311.71 to 1597.78) | -0.02 (-0.13 to 0.11) | -2.25 (-2.49 to -2) |
| Russian Federation | 5134684 (4942960 to 5276175) | 3081 (2948.28 to 3170.61) | 4128008 (3829112 to 4445427) | 1713.22 (1588.5 to 1846.08) | -0.20 (-0.24 to -0.15) | -2.75 (-3.27 to -2.22) |
| Rwanda | 38128 (30066 to 48575) | 1613.34 (1288.58 to 2017.67) | 42433 (32072 to 54790) | 862.19 (646.35 to 1121.93) | 0.11 (-0.19 to 0.49) | -2.83 (-3.19 to -2.48) |
| Saint Kitts and Nevis | 819 (737 to 889) | 2161.75 (1956.54 to 2343.47) | 628 (535 to 711) | 1147.15 (991.43 to 1277.25) | -0.23 (-0.36 to -0.10) | -1.93 (-2.11 to -1.76) |
| Saint Lucia | 1364 (1266 to 1447) | 1768.93 (1647.83 to 1869.67) | 1799 (1537 to 2071) | 784.31 (668.99 to 903.25) | 0.32 (0.12 to 0.54) | -3.25 (-3.63 to -2.87) |
| Saint Vincent and the Grenadines | 842 (770 to 912) | 1225.28 (1122.21 to 1324.69) | 1000 (897 to 1104) | 761.51 (685.53 to 837.92) | 0.19 (0.05 to 0.34) | -1.43 (-1.64 to -1.23) |
| Samoa | 759 (652 to 894) | 1011.58 (859.88 to 1203.37) | 1115 (941 to 1299) | 863.82 (732.74 to 999.58) | 0.47 (0.23 to 0.77) | -0.58 (-0.64 to -0.51) |
| San Marino | 281 (236 to 324) | 749.76 (630.55 to 867.58) | 251 (190 to 325) | 269.78 (207.37 to 344.25) | -0.11 (-0.31 to 0.14) | -2.94 (-3.21 to -2.67) |
| Sao Tome and Principe | 804 (682 to 937) | 1294.43 (1106.82 to 1497.02) | 1424 (1191 to 1719) | 1447.23 (1237 to 1706.5) | 0.77 (0.50 to 1.06) | 0.45 (0.34 to 0.56) |
| Saudi Arabia | 97789 (77150 to 121602) | 1779.74 (1408.96 to 2201.17) | 216063 (174938 to 268571) | 1240.87 (1040.65 to 1500.1) | 1.21 (0.66 to 1.97) | -1.33 (-1.46 to -1.21) |
| Senegal | 50702 (42305 to 61074) | 1630.49 (1362.81 to 1989.3) | 99245 (78418 to 128640) | 1439.15 (1130.8 to 1862.31) | 0.96 (0.54 to 1.42) | -0.49 (-0.54 to -0.44) |
| Serbia | 309760 (276268 to 341752) | 3588.26 (3214.86 to 3931) | 344281 (293380 to 398179) | 1967.51 (1676.14 to 2285.43) | 0.11 (-0.07 to 0.34) | -2.5 (-2.75 to -2.26) |
| Seychelles | 704 (594 to 803) | 1236.84 (1043.96 to 1410.27) | 875 (718 to 1007) | 833.14 (691.41 to 953.01) | 0.24 (0.11 to 0.39) | -1 (-1.17 to -0.82) |
| Sierra Leone | 36547 (28947 to 44972) | 1719.25 (1409.3 to 2071.76) | 55693 (42580 to 72029) | 1554.32 (1220.47 to 1966.89) | 0.52 (0.19 to 0.97) | -0.13 (-0.28 to 0.03) |
| Singapore | 22308 (20632 to 23933) | 1138.64 (1056.37 to 1220.95) | 17033 (14427 to 19670) | 205.84 (174.5 to 237.16) | -0.24 (-0.32 to -0.17) | -5.61 (-5.81 to -5.4) |
| Slovakia | 107588 (96037 to 119740) | 1826.8 (1636.9 to 2026.11) | 89377 (76107 to 104499) | 927.7 (789.44 to 1083.27) | -0.17 (-0.30 to 0.00) | -2.27 (-2.36 to -2.18) |
| Slovenia | 34896 (32561 to 37032) | 1422.14 (1324.05 to 1509.57) | 21383 (18596 to 23932) | 418.24 (365.32 to 469.6) | -0.39 (-0.45 to -0.33) | -3.99 (-4.21 to -3.77) |
| Solomon Islands | 1210 (953 to 1568) | 1131.03 (900.05 to 1452.23) | 2966 (2291 to 3884) | 1062.03 (825.77 to 1384.27) | 1.45 (0.96 to 2.11) | -0.22 (-0.34 to -0.11) |
| Somalia | 18811 (13070 to 27153) | 1010.9 (706.28 to 1471.31) | 39115 (27179 to 56839) | 821.54 (567.69 to 1146.08) | 1.08 (0.65 to 1.67) | -0.68 (-0.71 to -0.64) |
| South Africa | 169711 (142211 to 191096) | 879.6 (724.73 to 991.3) | 404688 (365293 to 443552) | 1018.96 (920.18 to 1114.11) | 1.38 (1.16 to 1.73) | 0.54 (0.1 to 0.98) |
| South Sudan | 23225 (17638 to 30182) | 999.16 (762.31 to 1298.54) | 26423 (20042 to 35182) | 840.44 (635.29 to 1105.92) | 0.14 (-0.11 to 0.48) | -0.72 (-0.9 to -0.53) |
| Spain | 547934 (500367 to 581536) | 1009.5 (920.64 to 1073.65) | 306534 (264923 to 339166) | 257.62 (225.21 to 285.66) | -0.44 (-0.48 to -0.40) | -4.39 (-4.64 to -4.14) |
| Sri Lanka | 140297 (127041 to 153509) | 1637.09 (1493.98 to 1780.14) | 267585 (199624 to 337945) | 1117.68 (830.94 to 1403.7) | 0.91 (0.38 to 1.52) | -0.69 (-0.93 to -0.46) |
| Sudan | 184698 (136595 to 238857) | 1977.53 (1446.19 to 2528.43) | 283445 (202996 to 377398) | 1526.26 (1123.15 to 1997) | 0.53 (0.16 to 1.06) | -0.95 (-0.99 to -0.9) |
| Suriname | 2339 (2107 to 2600) | 1003.99 (903.09 to 1109.42) | 4397 (3499 to 5392) | 737.09 (582.92 to 902.35) | 0.88 (0.48 to 1.37) | -0.99 (-1.25 to -0.73) |
| Sweden | 127879 (116291 to 136882) | 756.36 (687.74 to 810.29) | 73163 (62149 to 82863) | 287.33 (245.87 to 328.35) | -0.43 (-0.48 to -0.38) | -3.31 (-3.48 to -3.14) |
| Switzerland | 73861 (66781 to 78954) | 641.6 (579.84 to 686.28) | 44159 (37922 to 49457) | 202.32 (174.24 to 225.85) | -0.40 (-0.45 to -0.36) | -3.66 (-3.8 to -3.52) |
| Syrian Arab Republic | 86103 (71668 to 103015) | 1584.22 (1319.17 to 1899.4) | 133988 (104878 to 167219) | 1185.17 (947.67 to 1459) | 0.56 (0.17 to 1.14) | -1.4 (-1.61 to -1.19) |
| Taiwan (Province of China) | 154984 (143514 to 165556) | 1144.29 (1059.55 to 1224.12) | 161449 (139143 to 182768) | 383.32 (329.68 to 435.13) | 0.04 (-0.05 to 0.13) | -3.5 (-3.69 to -3.3) |
| Tajikistan | 42938 (35236 to 50399) | 1643.85 (1350.62 to 1927.38) | 63406 (51375 to 76772) | 1333.42 (1083.64 to 1597.02) | 0.48 (0.14 to 0.92) | -0.94 (-1.28 to -0.6) |
| Thailand | 283739 (240863 to 330754) | 900.86 (763.3 to 1050.57) | 615507 (499422 to 746499) | 577.11 (468.71 to 697.91) | 1.17 (0.73 to 1.76) | -1.93 (-2.12 to -1.73) |
| Timor-Leste | 2612 (2167 to 3111) | 1155.22 (960.39 to 1359.94) | 9556 (7036 to 12343) | 1245.24 (926.07 to 1613.04) | 2.66 (1.69 to 3.91) | 0.44 (0.28 to 0.61) |
| Togo | 18198 (14928 to 21806) | 1589.81 (1315.93 to 1914.27) | 49746 (36902 to 62920) | 1542.66 (1169.66 to 1940.52) | 1.73 (1.11 to 2.44) | -0.21 (-0.36 to -0.07) |
| Tokelau | 15 (12 to 18) | 1157.85 (944.13 to 1396.06) | 13 (10 to 15) | 847.29 (693.14 to 1023.91) | -0.16 (-0.33 to 0.06) | -1.16 (-1.21 to -1.11) |
| Tonga | 325 (277 to 382) | 652.03 (555.31 to 762.63) | 467 (385 to 550) | 600.92 (495.42 to 711.14) | 0.44 (0.17 to 0.78) | -0.18 (-0.27 to -0.08) |
| Trinidad and Tobago | 10599 (9962 to 11192) | 1398.7 (1310.73 to 1470.2) | 13346 (10799 to 16321) | 716.63 (581.65 to 872.32) | 0.26 (0.03 to 0.55) | -2.49 (-2.71 to -2.27) |
| Tunisia | 58996 (47340 to 72600) | 1360.11 (1092.47 to 1665.78) | 125685 (92452 to 167656) | 1025.8 (750.4 to 1367.58) | 1.13 (0.58 to 1.83) | -1.08 (-1.18 to -0.98) |
| Türkiye | 29944 (24879 to 34400) | 1633.28 (1377.38 to 1861.61) | 72322 (56423 to 90146) | 1933.32 (1522.27 to 2395.41) | 0.44 (0.17 to 0.77) | 0.3 (-0.14 to 0.74) |
| Turkmenistan | 78 (65 to 94) | 1361.85 (1146.68 to 1630) | 98 (82 to 116) | 1065.13 (893.1 to 1247.27) | 1.42 (0.96 to 1.97) | -0.83 (-0.87 to -0.78) |
| Tuvalu | 464951 (403272 to 536594) | 1490.86 (1284.37 to 1706.18) | 670470 (566003 to 797213) | 783.65 (661.83 to 924.04) | 0.26 (0.04 to 0.54) | -2.05 (-2.3 to -1.8) |
| Uganda | 54816 (42840 to 69490) | 976.37 (765.81 to 1240.89) | 93294 (74822 to 118587) | 756.98 (610.55 to 945.1) | 0.70 (0.32 to 1.21) | -1.39 (-1.64 to -1.14) |
| Ukraine | 1777604 (1682460 to 1859726) | 2593.14 (2442.04 to 2713.4) | 1154862 (915982 to 1417130) | 1462.06 (1160.17 to 1796.03) | -0.35 (-0.48 to -0.20) | -2.5 (-2.78 to -2.22) |
| United Arab Emirates | 7115 (5740 to 8785) | 1757.39 (1427.36 to 2138.12) | 27700 (22906 to 33046) | 1113.87 (924.59 to 1321.16) | 2.89 (2.20 to 3.92) | 0.02 (-0.47 to 0.51) |
| United Kingdom | 932293 (868975 to 973657) | 962.4 (894.91 to 1008.26) | 397336 (354482 to 433353) | 269.35 (240.48 to 295.88) | -0.57 (-0.60 to -0.55) | -4.38 (-4.56 to -4.2) |
| United Republic of Tanzania | 74960 (60052 to 95113) | 800.13 (644.82 to 1024.02) | 211680 (161632 to 275192) | 975.6 (747.57 to 1259.06) | 1.82 (1.04 to 2.88) | 0.59 (0.44 to 0.75) |
| United States of America | 1830268 (1644784 to 1989854) | 552.57 (496.57 to 601.36) | 2178054 (1909480 to 2407925) | 362.81 (318.36 to 404.82) | 0.19 (0.14 to 0.23) | -1.85 (-2.09 to -1.6) |
| United States Virgin Islands | 479 (400 to 559) | 698.9 (589.39 to 805.96) | 582 (477 to 700) | 327.01 (270.25 to 392.48) | 0.22 (-0.05 to 0.53) | -2.32 (-2.46 to -2.18) |
| Uruguay | 49341 (45815 to 52489) | 1260.07 (1167.56 to 1338.67) | 35404 (31882 to 37966) | 564.96 (513.64 to 606.13) | -0.28 (-0.33 to -0.23) | -2.79 (-2.95 to -2.62) |
| Uzbekistan | 155904 (143878 to 169294) | 1371.01 (1264.85 to 1484) | 297392 (259388 to 339123) | 1268.87 (1116.74 to 1438.77) | 0.91 (0.64 to 1.25) | -0.83 (-1.14 to -0.51) |
| Vanuatu | 699 (564 to 860) | 1313.15 (1065.79 to 1612.58) | 1612 (1308 to 1947) | 1097.57 (882.07 to 1314.38) | 1.31 (0.87 to 1.82) | -0.72 (-0.77 to -0.66) |
| Venezuela (Bolivarian Republic of) | 50868 (45768 to 55352) | 575.25 (517.95 to 624.23) | 125337 (99415 to 156775) | 456.48 (362.65 to 569.41) | 1.46 (0.95 to 2.26) | -1.04 (-1.3 to -0.77) |
| Viet Nam | 570624 (469285 to 703462) | 1554.33 (1279.57 to 1917.01) | 1418783 (1129765 to 1695470) | 1642.27 (1329.12 to 1944.66) | 1.49 (0.87 to 2.14) | 0.47 (0.34 to 0.6) |
| Yemen | 98059 (72605 to 129410) | 2141.79 (1591.92 to 2874.73) | 246350 (178758 to 335845) | 1945.48 (1433.7 to 2655.3) | 1.51 (0.85 to 2.40) | -0.5 (-0.58 to -0.42) |
| Zambia | 23725 (18172 to 31813) | 1018.89 (784.89 to 1368.01) | 63114 (48813 to 81719) | 1168.35 (897.84 to 1505.63) | 1.66 (1.03 to 2.43) | 0.33 (0.24 to 0.42) |
| Zimbabwe | 30325 (25679 to 35512) | 929.76 (781.86 to 1087.22) | 71195 (58621 to 86965) | 1298.65 (1081.01 to 1544.02) | 1.35 (0.87 to 2.02) | 1.6 (1.13 to 2.08) |
