# Supplementary material for: Global, regional, and national epidemiology of ischemic stroke from 1990 to 2021
Source: Eur J Neurol. 2024 Sep 17;31(12):e16481. doi: 10.1111/ene.16481 (PMC11555022; doi:10.1111/ene.16481)
Supplement: Supplementary file 8 — TABLE S7. APC of incidence, death, and DALYs for ischemic stroke in 204 countries and territories. APC, annual percent change; DALY, disability‐adjusted life year. [file ENE-31-e16481-s003.docx]

Supplementary Table 7. The APC of incidence, death, and DALYs of Ischemic Stroke in 204 countries and territories. APC = Annual percent change. DALYs = Disability-Adjusted Life Years.

| **Location** | **Measure** | **Segment Start** | **Segment End** | **APC (95%CI)** | **P** |
| --- | --- | --- | --- | --- | --- |
| Afghanistan | incidence | 1990 | 1995 | -0.32 (-0.39 to -0.26) | <0.001 |
| Afghanistan | incidence | 1995 | 2005 | 0.25 (0.23 to 0.28) | <0.001 |
| Afghanistan | incidence | 2005 | 2014 | -0.81 (-0.84 to -0.78) | <0.001 |
| Afghanistan | incidence | 2014 | 2019 | 0.15 (0.07 to 0.23) | 0.001 |
| Afghanistan | incidence | 2019 | 2021 | -0.79 (-1.06 to -0.52) | <0.001 |
| Albania | incidence | 1990 | 1994 | -2.17 (-2.46 to -1.87) | <0.001 |
| Albania | incidence | 1994 | 2000 | -1.11 (-1.32 to -0.89) | <0.001 |
| Albania | incidence | 2000 | 2011 | 0.20 (0.13 to 0.27) | <0.001 |
| Albania | incidence | 2011 | 2015 | -0.92 (-1.35 to -0.49) | <0.001 |
| Albania | incidence | 2015 | 2021 | 0.89 (0.74 to 1.04) | <0.001 |
| Algeria | incidence | 1990 | 1994 | 1.10 (0.69 to 1.52) | <0.001 |
| Algeria | incidence | 1994 | 2000 | -0.24 (-0.53 to 0.04) | 0.091 |
| Algeria | incidence | 2000 | 2004 | -2.29 (-2.96 to -1.62) | <0.001 |
| Algeria | incidence | 2004 | 2010 | -1.49 (-1.78 to -1.19) | <0.001 |
| Algeria | incidence | 2010 | 2018 | 0.70 (0.53 to 0.88) | <0.001 |
| Algeria | incidence | 2018 | 2021 | -1.68 (-2.38 to -0.98) | <0.001 |
| American Samoa | incidence | 1990 | 1996 | -0.08 (-0.19 to 0.02) | 0.11 |
| American Samoa | incidence | 1996 | 2001 | -0.79 (-0.96 to -0.61) | <0.001 |
| American Samoa | incidence | 2001 | 2010 | -1.18 (-1.24 to -1.12) | <0.001 |
| American Samoa | incidence | 2010 | 2014 | -0.74 (-1.02 to -0.47) | <0.001 |
| American Samoa | incidence | 2014 | 2021 | -0.18 (-0.25 to -0.10) | <0.001 |
| Andorra | incidence | 1990 | 1999 | -1.57 (-1.64 to -1.49) | <0.001 |
| Andorra | incidence | 1999 | 2008 | -2.07 (-2.16 to -1.98) | <0.001 |
| Andorra | incidence | 2008 | 2014 | -1.34 (-1.52 to -1.17) | <0.001 |
| Andorra | incidence | 2014 | 2018 | 0.22 (-0.18 to 0.62) | 0.27 |
| Andorra | incidence | 2018 | 2021 | 1.13 (0.71 to 1.55) | <0.001 |
| Angola | incidence | 1990 | 2001 | -0.28 (-0.35 to -0.22) | <0.001 |
| Angola | incidence | 2001 | 2010 | -0.55 (-0.64 to -0.45) | <0.001 |
| Angola | incidence | 2010 | 2018 | -0.17 (-0.28 to -0.06) | 0.004 |
| Angola | incidence | 2018 | 2021 | -0.83 (-1.25 to -0.40) | 0.001 |
| Antigua and Barbuda | incidence | 1990 | 1995 | -0.77 (-0.80 to -0.74) | <0.001 |
| Antigua and Barbuda | incidence | 1995 | 2000 | -1.08 (-1.12 to -1.04) | <0.001 |
| Antigua and Barbuda | incidence | 2000 | 2005 | -1.68 (-1.72 to -1.64) | <0.001 |
| Antigua and Barbuda | incidence | 2005 | 2009 | -0.73 (-0.80 to -0.67) | <0.001 |
| Antigua and Barbuda | incidence | 2009 | 2015 | -0.34 (-0.37 to -0.31) | <0.001 |
| Antigua and Barbuda | incidence | 2015 | 2021 | 0.12 (0.10 to 0.15) | <0.001 |
| Argentina | incidence | 1990 | 1996 | -1.02 (-1.09 to -0.95) | <0.001 |
| Argentina | incidence | 1996 | 2000 | -2.39 (-2.60 to -2.18) | <0.001 |
| Argentina | incidence | 2000 | 2005 | -3.35 (-3.47 to -3.23) | <0.001 |
| Argentina | incidence | 2005 | 2014 | -1.33 (-1.36 to -1.29) | <0.001 |
| Argentina | incidence | 2014 | 2019 | -0.04 (-0.16 to 0.08) | 0.505 |
| Argentina | incidence | 2019 | 2021 | -0.72 (-1.11 to -0.33) | 0.001 |
| Armenia | incidence | 1990 | 2001 | -1.19 (-1.27 to -1.12) | <0.001 |
| Armenia | incidence | 2001 | 2005 | -3.71 (-4.18 to -3.23) | <0.001 |
| Armenia | incidence | 2005 | 2015 | -1.51 (-1.60 to -1.43) | <0.001 |
| Armenia | incidence | 2015 | 2019 | 0.59 (0.12 to 1.06) | 0.017 |
| Armenia | incidence | 2019 | 2021 | -1.83 (-2.76 to -0.90) | 0.001 |
| Australia | incidence | 1990 | 1995 | -1.45 (-1.58 to -1.32) | <0.001 |
| Australia | incidence | 1995 | 2005 | -2.12 (-2.18 to -2.06) | <0.001 |
| Australia | incidence | 2005 | 2010 | -4.14 (-4.35 to -3.93) | <0.001 |
| Australia | incidence | 2010 | 2014 | -1.09 (-1.45 to -0.73) | <0.001 |
| Australia | incidence | 2014 | 2021 | -0.07 (-0.18 to 0.05) | 0.256 |
| Austria | incidence | 1990 | 2000 | -0.31 (-0.42 to -0.21) | <0.001 |
| Austria | incidence | 2000 | 2008 | 1.02 (0.87 to 1.18) | <0.001 |
| Austria | incidence | 2008 | 2011 | 0.03 (-1.16 to 1.24) | 0.952 |
| Austria | incidence | 2011 | 2015 | -2.54 (-3.37 to -1.71) | <0.001 |
| Austria | incidence | 2015 | 2019 | -5.58 (-6.66 to -4.48) | <0.001 |
| Austria | incidence | 2019 | 2021 | -0.56 (-3.28 to 2.24) | 0.672 |
| Azerbaijan | incidence | 1990 | 1995 | -1.44 (-1.60 to -1.29) | <0.001 |
| Azerbaijan | incidence | 1995 | 2004 | 0.50 (0.43 to 0.58) | <0.001 |
| Azerbaijan | incidence | 2004 | 2010 | 2.15 (1.99 to 2.30) | <0.001 |
| Azerbaijan | incidence | 2010 | 2015 | -0.13 (-0.33 to 0.07) | 0.184 |
| Azerbaijan | incidence | 2015 | 2019 | 0.62 (0.30 to 0.93) | 0.001 |
| Azerbaijan | incidence | 2019 | 2021 | -1.94 (-2.64 to -1.22) | <0.001 |
| Bahamas | incidence | 1990 | 1995 | -0.75 (-0.81 to -0.70) | <0.001 |
| Bahamas | incidence | 1995 | 2000 | -1.09 (-1.17 to -1.02) | <0.001 |
| Bahamas | incidence | 2000 | 2010 | -0.68 (-0.70 to -0.66) | <0.001 |
| Bahamas | incidence | 2010 | 2014 | -0.42 (-0.53 to -0.31) | <0.001 |
| Bahamas | incidence | 2014 | 2019 | -0.26 (-0.34 to -0.18) | <0.001 |
| Bahamas | incidence | 2019 | 2021 | 0.15 (-0.07 to 0.37) | 0.173 |
| Bahrain | incidence | 1990 | 2000 | -1.06 (-1.08 to -1.05) | <0.001 |
| Bahrain | incidence | 2000 | 2006 | -0.74 (-0.80 to -0.69) | <0.001 |
| Bahrain | incidence | 2006 | 2011 | -1.60 (-1.68 to -1.53) | <0.001 |
| Bahrain | incidence | 2011 | 2014 | -2.58 (-2.81 to -2.35) | <0.001 |
| Bahrain | incidence | 2014 | 2019 | 0.00 (-0.08 to 0.07) | 0.958 |
| Bahrain | incidence | 2019 | 2021 | -1.72 (-1.97 to -1.47) | <0.001 |
| Bangladesh | incidence | 1990 | 1999 | 0.39 (0.35 to 0.42) | <0.001 |
| Bangladesh | incidence | 1999 | 2005 | 1.03 (0.94 to 1.11) | <0.001 |
| Bangladesh | incidence | 2005 | 2010 | -1.40 (-1.51 to -1.28) | <0.001 |
| Bangladesh | incidence | 2010 | 2014 | -0.41 (-0.58 to -0.24) | <0.001 |
| Bangladesh | incidence | 2014 | 2019 | 0.37 (0.26 to 0.48) | <0.001 |
| Bangladesh | incidence | 2019 | 2021 | -2.04 (-2.42 to -1.66) | <0.001 |
| Barbados | incidence | 1990 | 1994 | -1.60 (-1.71 to -1.49) | <0.001 |
| Barbados | incidence | 1994 | 2000 | -1.35 (-1.42 to -1.27) | <0.001 |
| Barbados | incidence | 2000 | 2005 | -1.74 (-1.84 to -1.64) | <0.001 |
| Barbados | incidence | 2005 | 2010 | -0.61 (-0.71 to -0.52) | <0.001 |
| Barbados | incidence | 2010 | 2015 | 0.35 (0.25 to 0.46) | <0.001 |
| Barbados | incidence | 2015 | 2021 | -0.13 (-0.19 to -0.07) | <0.001 |
| Belarus | incidence | 1990 | 2005 | 0.07 (0.05 to 0.09) | <0.001 |
| Belarus | incidence | 2005 | 2010 | -1.39 (-1.54 to -1.23) | <0.001 |
| Belarus | incidence | 2010 | 2014 | -3.41 (-3.67 to -3.15) | <0.001 |
| Belarus | incidence | 2014 | 2019 | -0.24 (-0.44 to -0.05) | 0.016 |
| Belarus | incidence | 2019 | 2021 | -2.77 (-3.39 to -2.15) | <0.001 |
| Belgium | incidence | 1990 | 1995 | -3.68 (-3.89 to -3.46) | <0.001 |
| Belgium | incidence | 1995 | 1999 | -5.86 (-6.20 to -5.52) | <0.001 |
| Belgium | incidence | 1999 | 2002 | -1.97 (-2.51 to -1.42) | <0.001 |
| Belgium | incidence | 2002 | 2008 | -0.79 (-0.89 to -0.69) | <0.001 |
| Belgium | incidence | 2008 | 2021 | -0.53 (-0.56 to -0.50) | <0.001 |
| Belize | incidence | 1990 | 1992 | 1.87 (1.42 to 2.33) | <0.001 |
| Belize | incidence | 1992 | 1995 | 0.86 (0.46 to 1.27) | <0.001 |
| Belize | incidence | 1995 | 2005 | -0.26 (-0.30 to -0.23) | <0.001 |
| Belize | incidence | 2005 | 2010 | -2.16 (-2.28 to -2.03) | <0.001 |
| Belize | incidence | 2010 | 2015 | -0.61 (-0.73 to -0.48) | <0.001 |
| Belize | incidence | 2015 | 2021 | 0.07 (0.00 to 0.14) | 0.042 |
| Benin | incidence | 1990 | 2001 | -0.55 (-0.57 to -0.53) | <0.001 |
| Benin | incidence | 2001 | 2010 | -1.05 (-1.08 to -1.02) | <0.001 |
| Benin | incidence | 2010 | 2015 | -0.56 (-0.65 to -0.47) | <0.001 |
| Benin | incidence | 2015 | 2019 | 0.54 (0.40 to 0.68) | <0.001 |
| Benin | incidence | 2019 | 2021 | -0.81 (-1.11 to -0.51) | <0.001 |
| Bermuda | incidence | 1990 | 1993 | -0.87 (-1.05 to -0.68) | <0.001 |
| Bermuda | incidence | 1993 | 1996 | -1.26 (-1.64 to -0.89) | <0.001 |
| Bermuda | incidence | 1996 | 2005 | -2.10 (-2.14 to -2.06) | <0.001 |
| Bermuda | incidence | 2005 | 2009 | -2.73 (-2.92 to -2.55) | <0.001 |
| Bermuda | incidence | 2009 | 2014 | -2.02 (-2.13 to -1.90) | <0.001 |
| Bermuda | incidence | 2014 | 2021 | 0.04 (-0.02 to 0.09) | 0.158 |
| Bhutan | incidence | 1990 | 1995 | -0.14 (-0.26 to -0.02) | 0.027 |
| Bhutan | incidence | 1995 | 2006 | -0.54 (-0.58 to -0.50) | <0.001 |
| Bhutan | incidence | 2006 | 2010 | -1.04 (-1.29 to -0.79) | <0.001 |
| Bhutan | incidence | 2010 | 2015 | -0.17 (-0.32 to -0.02) | 0.028 |
| Bhutan | incidence | 2015 | 2019 | 0.78 (0.55 to 1.00) | <0.001 |
| Bhutan | incidence | 2019 | 2021 | -0.37 (-0.87 to 0.12) | 0.13 |
| Bolivia (Plurinational State of) | incidence | 1990 | 1993 | -0.51 (-0.78 to -0.24) | 0.001 |
| Bolivia (Plurinational State of) | incidence | 1993 | 1996 | -0.91 (-1.43 to -0.38) | 0.002 |
| Bolivia (Plurinational State of) | incidence | 1996 | 2000 | -1.85 (-2.11 to -1.60) | <0.001 |
| Bolivia (Plurinational State of) | incidence | 2000 | 2010 | -1.39 (-1.43 to -1.34) | <0.001 |
| Bolivia (Plurinational State of) | incidence | 2010 | 2014 | -0.32 (-0.57 to -0.06) | 0.018 |
| Bolivia (Plurinational State of) | incidence | 2014 | 2021 | 0.15 (0.08 to 0.22) | <0.001 |
| Bosnia and Herzegovina | incidence | 1990 | 1994 | -2.06 (-2.51 to -1.61) | <0.001 |
| Bosnia and Herzegovina | incidence | 1994 | 2000 | 0.59 (0.27 to 0.91) | 0.001 |
| Bosnia and Herzegovina | incidence | 2000 | 2005 | -0.96 (-1.37 to -0.54) | <0.001 |
| Bosnia and Herzegovina | incidence | 2005 | 2010 | 0.56 (0.14 to 0.98) | 0.012 |
| Bosnia and Herzegovina | incidence | 2010 | 2014 | -1.88 (-2.57 to -1.19) | <0.001 |
| Bosnia and Herzegovina | incidence | 2014 | 2021 | -0.03 (-0.22 to 0.17) | 0.767 |
| Botswana | incidence | 1990 | 2000 | 1.22 (1.08 to 1.37) | <0.001 |
| Botswana | incidence | 2000 | 2015 | -0.40 (-0.48 to -0.32) | <0.001 |
| Botswana | incidence | 2015 | 2018 | 0.34 (-1.19 to 1.89) | 0.655 |
| Botswana | incidence | 2018 | 2021 | -0.84 (-1.65 to -0.02) | 0.046 |
| Brazil | incidence | 1990 | 1996 | -1.28 (-1.37 to -1.19) | <0.001 |
| Brazil | incidence | 1996 | 2000 | -3.34 (-3.58 to -3.09) | <0.001 |
| Brazil | incidence | 2000 | 2011 | -2.37 (-2.41 to -2.33) | <0.001 |
| Brazil | incidence | 2011 | 2015 | -1.55 (-1.79 to -1.32) | <0.001 |
| Brazil | incidence | 2015 | 2019 | 0.07 (-0.16 to 0.31) | 0.524 |
| Brazil | incidence | 2019 | 2021 | -2.77 (-3.24 to -2.30) | <0.001 |
| Brunei Darussalam | incidence | 1990 | 1994 | -0.95 (-1.23 to -0.67) | <0.001 |
| Brunei Darussalam | incidence | 1994 | 1997 | -2.03 (-2.90 to -1.16) | <0.001 |
| Brunei Darussalam | incidence | 1997 | 2005 | -3.50 (-3.61 to -3.39) | <0.001 |
| Brunei Darussalam | incidence | 2005 | 2010 | -2.51 (-2.76 to -2.25) | <0.001 |
| Brunei Darussalam | incidence | 2010 | 2014 | -1.93 (-2.32 to -1.54) | <0.001 |
| Brunei Darussalam | incidence | 2014 | 2021 | 0.06 (-0.05 to 0.17) | 0.29 |
| Bulgaria | incidence | 1990 | 1996 | -0.57 (-0.64 to -0.49) | <0.001 |
| Bulgaria | incidence | 1996 | 2010 | 0.41 (0.39 to 0.43) | <0.001 |
| Bulgaria | incidence | 2010 | 2015 | -0.91 (-1.04 to -0.79) | <0.001 |
| Bulgaria | incidence | 2015 | 2019 | 0.36 (0.16 to 0.56) | 0.001 |
| Bulgaria | incidence | 2019 | 2021 | -1.22 (-1.64 to -0.80) | <0.001 |
| Burkina Faso | incidence | 1990 | 1994 | -1.02 (-1.13 to -0.92) | <0.001 |
| Burkina Faso | incidence | 1994 | 2005 | 0.22 (0.20 to 0.25) | <0.001 |
| Burkina Faso | incidence | 2005 | 2015 | -0.64 (-0.67 to -0.61) | <0.001 |
| Burkina Faso | incidence | 2015 | 2019 | 0.41 (0.27 to 0.56) | <0.001 |
| Burkina Faso | incidence | 2019 | 2021 | -0.28 (-0.59 to 0.03) | 0.077 |
| Burundi | incidence | 1990 | 1996 | -0.45 (-0.51 to -0.38) | <0.001 |
| Burundi | incidence | 1996 | 2000 | -1.22 (-1.40 to -1.03) | <0.001 |
| Burundi | incidence | 2000 | 2008 | -2.08 (-2.13 to -2.03) | <0.001 |
| Burundi | incidence | 2008 | 2014 | -1.37 (-1.45 to -1.29) | <0.001 |
| Burundi | incidence | 2014 | 2019 | 0.04 (-0.07 to 0.15) | 0.475 |
| Burundi | incidence | 2019 | 2021 | -0.53 (-0.91 to -0.14) | 0.01 |
| Côte d'Ivoire | incidence | 1990 | 1994 | -0.29 (-0.47 to -0.11) | 0.003 |
| Côte d'Ivoire | incidence | 1994 | 2001 | 0.17 (0.08 to 0.26) | 0.001 |
| Côte d'Ivoire | incidence | 2001 | 2014 | -1.25 (-1.28 to -1.22) | <0.001 |
| Côte d'Ivoire | incidence | 2014 | 2019 | 0.42 (0.26 to 0.58) | <0.001 |
| Côte d'Ivoire | incidence | 2019 | 2021 | -0.81 (-1.36 to -0.25) | 0.007 |
| Cabo Verde | incidence | 1990 | 1995 | -0.31 (-0.51 to -0.11) | 0.004 |
| Cabo Verde | incidence | 1995 | 2000 | 0.82 (0.54 to 1.09) | <0.001 |
| Cabo Verde | incidence | 2000 | 2007 | -0.40 (-0.54 to -0.26) | <0.001 |
| Cabo Verde | incidence | 2007 | 2021 | 0.63 (0.59 to 0.67) | <0.001 |
| Cambodia | incidence | 1990 | 2003 | -0.34 (-0.35 to -0.33) | <0.001 |
| Cambodia | incidence | 2003 | 2006 | -0.13 (-0.29 to 0.02) | 0.089 |
| Cambodia | incidence | 2006 | 2015 | 0.36 (0.34 to 0.37) | <0.001 |
| Cambodia | incidence | 2015 | 2018 | 0.71 (0.57 to 0.86) | <0.001 |
| Cambodia | incidence | 2018 | 2021 | 0.50 (0.42 to 0.58) | <0.001 |
| Cameroon | incidence | 1990 | 1995 | 0.53 (0.36 to 0.69) | <0.001 |
| Cameroon | incidence | 1995 | 2000 | 1.62 (1.40 to 1.83) | <0.001 |
| Cameroon | incidence | 2000 | 2006 | 0.40 (0.24 to 0.55) | <0.001 |
| Cameroon | incidence | 2006 | 2015 | -0.79 (-0.86 to -0.72) | <0.001 |
| Cameroon | incidence | 2015 | 2019 | 0.06 (-0.25 to 0.38) | 0.666 |
| Cameroon | incidence | 2019 | 2021 | -1.27 (-1.91 to -0.61) | 0.001 |
| Canada | incidence | 1990 | 1994 | -2.94 (-3.33 to -2.56) | <0.001 |
| Canada | incidence | 1994 | 2005 | -2.22 (-2.27 to -2.17) | <0.001 |
| Canada | incidence | 2005 | 2010 | 0.08 (-0.07 to 0.23) | 0.279 |
| Canada | incidence | 2010 | 2019 | -0.98 (-1.03 to -0.93) | <0.001 |
| Canada | incidence | 2019 | 2021 | -2.70 (-3.45 to -1.94) | <0.001 |
| Central African Republic | incidence | 1990 | 1994 | -0.51 (-0.71 to -0.30) | <0.001 |
| Central African Republic | incidence | 1994 | 2000 | -0.08 (-0.22 to 0.05) | 0.203 |
| Central African Republic | incidence | 2000 | 2005 | -0.51 (-0.71 to -0.32) | <0.001 |
| Central African Republic | incidence | 2005 | 2010 | 0.08 (-0.11 to 0.27) | 0.4 |
| Central African Republic | incidence | 2010 | 2015 | -0.56 (-0.74 to -0.38) | <0.001 |
| Central African Republic | incidence | 2015 | 2021 | 0.08 (-0.02 to 0.17) | 0.117 |
| Chad | incidence | 1990 | 1995 | -0.29 (-0.35 to -0.24) | <0.001 |
| Chad | incidence | 1995 | 2000 | 0.49 (0.41 to 0.56) | <0.001 |
| Chad | incidence | 2000 | 2006 | 0.00 (-0.05 to 0.05) | 0.988 |
| Chad | incidence | 2006 | 2015 | -0.47 (-0.50 to -0.45) | <0.001 |
| Chad | incidence | 2015 | 2019 | 0.26 (0.15 to 0.36) | <0.001 |
| Chad | incidence | 2019 | 2021 | -1.13 (-1.36 to -0.91) | <0.001 |
| Chile | incidence | 1990 | 1994 | -2.81 (-2.92 to -2.70) | <0.001 |
| Chile | incidence | 1994 | 2001 | -1.75 (-1.80 to -1.69) | <0.001 |
| Chile | incidence | 2001 | 2010 | -2.11 (-2.14 to -2.07) | <0.001 |
| Chile | incidence | 2010 | 2015 | -0.91 (-1.01 to -0.80) | <0.001 |
| Chile | incidence | 2015 | 2019 | 1.21 (1.03 to 1.39) | <0.001 |
| Chile | incidence | 2019 | 2021 | -2.00 (-2.37 to -1.62) | <0.001 |
| China | incidence | 1990 | 1994 | 1.66 (1.37 to 1.94) | <0.001 |
| China | incidence | 1994 | 2000 | 0.31 (0.12 to 0.49) | 0.003 |
| China | incidence | 2000 | 2007 | 1.65 (1.51 to 1.80) | <0.001 |
| China | incidence | 2007 | 2015 | 0.18 (0.07 to 0.29) | 0.003 |
| China | incidence | 2015 | 2019 | 2.44 (2.04 to 2.84) | <0.001 |
| China | incidence | 2019 | 2021 | -0.55 (-1.34 to 0.26) | 0.17 |
| Colombia | incidence | 1990 | 1996 | -1.42 (-1.50 to -1.34) | <0.001 |
| Colombia | incidence | 1996 | 2009 | -3.20 (-3.23 to -3.18) | <0.001 |
| Colombia | incidence | 2009 | 2014 | -1.57 (-1.72 to -1.43) | <0.001 |
| Colombia | incidence | 2014 | 2019 | -0.06 (-0.21 to 0.09) | 0.397 |
| Colombia | incidence | 2019 | 2021 | -1.11 (-1.59 to -0.62) | <0.001 |
| Comoros | incidence | 1990 | 1996 | -0.52 (-0.64 to -0.40) | <0.001 |
| Comoros | incidence | 1996 | 2000 | -0.89 (-1.23 to -0.56) | <0.001 |
| Comoros | incidence | 2000 | 2005 | -1.59 (-1.80 to -1.38) | <0.001 |
| Comoros | incidence | 2005 | 2009 | -0.74 (-1.08 to -0.41) | <0.001 |
| Comoros | incidence | 2009 | 2014 | -0.46 (-0.67 to -0.24) | <0.001 |
| Comoros | incidence | 2014 | 2021 | 0.13 (0.04 to 0.22) | 0.01 |
| Congo | incidence | 1990 | 1997 | -0.17 (-0.36 to 0.02) | 0.076 |
| Congo | incidence | 1997 | 2009 | -0.97 (-1.05 to -0.88) | <0.001 |
| Congo | incidence | 2009 | 2021 | -0.25 (-0.32 to -0.17) | <0.001 |
| Cook Islands | incidence | 1990 | 1995 | 0.15 (0.07 to 0.22) | 0.001 |
| Cook Islands | incidence | 1995 | 2003 | -0.88 (-0.93 to -0.84) | <0.001 |
| Cook Islands | incidence | 2003 | 2009 | -0.62 (-0.69 to -0.56) | <0.001 |
| Cook Islands | incidence | 2009 | 2015 | 0.05 (-0.02 to 0.13) | 0.132 |
| Cook Islands | incidence | 2015 | 2021 | 0.40 (0.35 to 0.46) | <0.001 |
| Costa Rica | incidence | 1990 | 1995 | -0.95 (-1.06 to -0.85) | <0.001 |
| Costa Rica | incidence | 1995 | 1998 | -1.65 (-2.09 to -1.21) | <0.001 |
| Costa Rica | incidence | 1998 | 2009 | -2.11 (-2.15 to -2.08) | <0.001 |
| Costa Rica | incidence | 2009 | 2015 | -0.33 (-0.43 to -0.22) | <0.001 |
| Costa Rica | incidence | 2015 | 2021 | 0.29 (0.21 to 0.37) | <0.001 |
| Croatia | incidence | 1990 | 1995 | -2.46 (-2.56 to -2.36) | <0.001 |
| Croatia | incidence | 1995 | 2004 | -1.78 (-1.82 to -1.74) | <0.001 |
| Croatia | incidence | 2004 | 2010 | -1.13 (-1.21 to -1.06) | <0.001 |
| Croatia | incidence | 2010 | 2015 | -1.91 (-2.01 to -1.80) | <0.001 |
| Croatia | incidence | 2015 | 2021 | -0.63 (-0.69 to -0.57) | <0.001 |
| Cuba | incidence | 1990 | 1994 | -1.33 (-1.50 to -1.15) | <0.001 |
| Cuba | incidence | 1994 | 2005 | -0.23 (-0.27 to -0.19) | <0.001 |
| Cuba | incidence | 2005 | 2015 | -0.55 (-0.60 to -0.50) | <0.001 |
| Cuba | incidence | 2015 | 2018 | 0.16 (-0.37 to 0.69) | 0.542 |
| Cuba | incidence | 2018 | 2021 | -0.34 (-0.61 to -0.07) | 0.016 |
| Cyprus | incidence | 1990 | 1993 | 1.17 (-0.70 to 3.07) | 0.204 |
| Cyprus | incidence | 1993 | 1996 | -1.82 (-5.30 to 1.78) | 0.294 |
| Cyprus | incidence | 1996 | 2001 | -7.99 (-9.03 to -6.93) | <0.001 |
| Cyprus | incidence | 2001 | 2008 | -4.32 (-4.87 to -3.76) | <0.001 |
| Cyprus | incidence | 2008 | 2014 | -1.54 (-2.18 to -0.88) | <0.001 |
| Cyprus | incidence | 2014 | 2021 | 1.73 (1.35 to 2.12) | <0.001 |
| Czechia | incidence | 1990 | 1995 | -1.95 (-2.13 to -1.76) | <0.001 |
| Czechia | incidence | 1995 | 1999 | -3.21 (-3.55 to -2.87) | <0.001 |
| Czechia | incidence | 1999 | 2010 | -2.44 (-2.48 to -2.39) | <0.001 |
| Czechia | incidence | 2010 | 2014 | -4.12 (-4.39 to -3.84) | <0.001 |
| Czechia | incidence | 2014 | 2019 | -0.67 (-0.89 to -0.45) | <0.001 |
| Czechia | incidence | 2019 | 2021 | -2.60 (-3.42 to -1.78) | <0.001 |
| Democratic People's Republic of Korea | incidence | 1990 | 2000 | 0.54 (0.52 to 0.56) | <0.001 |
| Democratic People's Republic of Korea | incidence | 2000 | 2006 | 0.23 (0.17 to 0.28) | <0.001 |
| Democratic People's Republic of Korea | incidence | 2006 | 2015 | -0.53 (-0.55 to -0.50) | <0.001 |
| Democratic People's Republic of Korea | incidence | 2015 | 2019 | 0.73 (0.60 to 0.86) | <0.001 |
| Democratic People's Republic of Korea | incidence | 2019 | 2021 | -0.67 (-0.94 to -0.40) | <0.001 |
| Democratic Republic of the Congo | incidence | 1990 | 2004 | -0.61 (-0.65 to -0.57) | <0.001 |
| Democratic Republic of the Congo | incidence | 2004 | 2015 | -0.39 (-0.45 to -0.33) | <0.001 |
| Democratic Republic of the Congo | incidence | 2015 | 2021 | 0.08 (-0.06 to 0.21) | 0.249 |
| Denmark | incidence | 1990 | 1996 | -0.90 (-0.99 to -0.81) | <0.001 |
| Denmark | incidence | 1996 | 2000 | -4.49 (-4.72 to -4.26) | <0.001 |
| Denmark | incidence | 2000 | 2005 | -2.49 (-2.64 to -2.34) | <0.001 |
| Denmark | incidence | 2005 | 2012 | -3.05 (-3.13 to -2.97) | <0.001 |
| Denmark | incidence | 2012 | 2018 | -2.46 (-2.58 to -2.34) | <0.001 |
| Denmark | incidence | 2018 | 2021 | -0.11 (-0.49 to 0.27) | 0.542 |
| Djibouti | incidence | 1990 | 1994 | -0.58 (-0.84 to -0.33) | <0.001 |
| Djibouti | incidence | 1994 | 2000 | 0.00 (-0.18 to 0.17) | 0.972 |
| Djibouti | incidence | 2000 | 2005 | -0.48 (-0.71 to -0.25) | <0.001 |
| Djibouti | incidence | 2005 | 2015 | 0.09 (0.03 to 0.16) | 0.008 |
| Djibouti | incidence | 2015 | 2021 | 0.32 (0.19 to 0.45) | <0.001 |
| Dominica | incidence | 1990 | 1994 | -0.71 (-0.75 to -0.67) | <0.001 |
| Dominica | incidence | 1994 | 2000 | -0.48 (-0.50 to -0.45) | <0.001 |
| Dominica | incidence | 2000 | 2010 | -0.27 (-0.28 to -0.26) | <0.001 |
| Dominica | incidence | 2010 | 2019 | -0.08 (-0.09 to -0.07) | <0.001 |
| Dominica | incidence | 2019 | 2021 | 0.40 (0.28 to 0.52) | <0.001 |
| Dominican Republic | incidence | 1990 | 1995 | 0.17 (0.11 to 0.24) | <0.001 |
| Dominican Republic | incidence | 1995 | 2006 | 0.78 (0.76 to 0.80) | <0.001 |
| Dominican Republic | incidence | 2006 | 2009 | 1.24 (0.96 to 1.52) | <0.001 |
| Dominican Republic | incidence | 2009 | 2012 | 0.73 (0.43 to 1.03) | <0.001 |
| Dominican Republic | incidence | 2012 | 2019 | 0.44 (0.40 to 0.49) | <0.001 |
| Dominican Republic | incidence | 2019 | 2021 | 1.00 (0.68 to 1.31) | <0.001 |
| Ecuador | incidence | 1990 | 1995 | -0.75 (-0.86 to -0.63) | <0.001 |
| Ecuador | incidence | 1995 | 1999 | -1.27 (-1.52 to -1.02) | <0.001 |
| Ecuador | incidence | 1999 | 2013 | -0.93 (-0.95 to -0.90) | <0.001 |
| Ecuador | incidence | 2013 | 2019 | -0.14 (-0.25 to -0.04) | 0.013 |
| Ecuador | incidence | 2019 | 2021 | -1.28 (-1.81 to -0.75) | <0.001 |
| Egypt | incidence | 1990 | 1995 | 0.59 (0.54 to 0.63) | <0.001 |
| Egypt | incidence | 1995 | 2000 | 1.16 (1.10 to 1.23) | <0.001 |
| Egypt | incidence | 2000 | 2010 | 0.88 (0.86 to 0.89) | <0.001 |
| Egypt | incidence | 2010 | 2015 | -0.59 (-0.65 to -0.53) | <0.001 |
| Egypt | incidence | 2015 | 2019 | 0.49 (0.40 to 0.59) | <0.001 |
| Egypt | incidence | 2019 | 2021 | -0.99 (-1.20 to -0.78) | <0.001 |
| El Salvador | incidence | 1990 | 1996 | -0.90 (-1.02 to -0.77) | <0.001 |
| El Salvador | incidence | 1996 | 2005 | -1.84 (-1.92 to -1.77) | <0.001 |
| El Salvador | incidence | 2005 | 2009 | -1.50 (-1.86 to -1.13) | <0.001 |
| El Salvador | incidence | 2009 | 2014 | -0.52 (-0.76 to -0.28) | <0.001 |
| El Salvador | incidence | 2014 | 2018 | 0.04 (-0.30 to 0.38) | 0.797 |
| El Salvador | incidence | 2018 | 2021 | 0.81 (0.47 to 1.15) | <0.001 |
| Equatorial Guinea | incidence | 1990 | 1994 | -1.39 (-1.58 to -1.20) | <0.001 |
| Equatorial Guinea | incidence | 1994 | 2001 | -0.62 (-0.72 to -0.52) | <0.001 |
| Equatorial Guinea | incidence | 2001 | 2011 | -1.22 (-1.27 to -1.17) | <0.001 |
| Equatorial Guinea | incidence | 2011 | 2021 | -0.34 (-0.39 to -0.30) | <0.001 |
| Eritrea | incidence | 1990 | 2000 | -0.36 (-0.43 to -0.29) | <0.001 |
| Eritrea | incidence | 2000 | 2009 | -1.18 (-1.28 to -1.09) | <0.001 |
| Eritrea | incidence | 2009 | 2015 | -0.36 (-0.56 to -0.16) | 0.001 |
| Eritrea | incidence | 2015 | 2018 | 0.49 (-0.38 to 1.36) | 0.252 |
| Eritrea | incidence | 2018 | 2021 | -0.49 (-0.96 to -0.01) | 0.047 |
| Estonia | incidence | 1990 | 1995 | -1.74 (-1.84 to -1.65) | <0.001 |
| Estonia | incidence | 1995 | 2003 | -2.18 (-2.23 to -2.13) | <0.001 |
| Estonia | incidence | 2003 | 2006 | -2.84 (-3.33 to -2.34) | <0.001 |
| Estonia | incidence | 2006 | 2014 | -5.72 (-5.79 to -5.65) | <0.001 |
| Estonia | incidence | 2014 | 2019 | 0.00 (-0.20 to 0.19) | 0.969 |
| Estonia | incidence | 2019 | 2021 | -2.61 (-3.31 to -1.92) | <0.001 |
| Eswatini | incidence | 1990 | 1995 | 0.53 (0.27 to 0.79) | 0.001 |
| Eswatini | incidence | 1995 | 2005 | 1.35 (1.25 to 1.45) | <0.001 |
| Eswatini | incidence | 2005 | 2010 | 0.02 (-0.30 to 0.35) | 0.878 |
| Eswatini | incidence | 2010 | 2015 | -1.01 (-1.33 to -0.70) | <0.001 |
| Eswatini | incidence | 2015 | 2018 | 0.60 (-0.37 to 1.59) | 0.208 |
| Eswatini | incidence | 2018 | 2021 | -0.79 (-1.31 to -0.27) | 0.006 |
| Ethiopia | incidence | 1990 | 1994 | -1.69 (-1.86 to -1.52) | <0.001 |
| Ethiopia | incidence | 1994 | 2000 | -1.18 (-1.29 to -1.07) | <0.001 |
| Ethiopia | incidence | 2000 | 2005 | -1.79 (-1.95 to -1.63) | <0.001 |
| Ethiopia | incidence | 2005 | 2009 | -1.46 (-1.70 to -1.20) | <0.001 |
| Ethiopia | incidence | 2009 | 2014 | -0.38 (-0.53 to -0.22) | <0.001 |
| Ethiopia | incidence | 2014 | 2021 | 0.71 (0.65 to 0.78) | <0.001 |
| Fiji | incidence | 1990 | 1995 | 0.59 (0.47 to 0.70) | <0.001 |
| Fiji | incidence | 1995 | 2001 | -0.31 (-0.42 to -0.20) | <0.001 |
| Fiji | incidence | 2001 | 2010 | -1.22 (-1.28 to -1.17) | <0.001 |
| Fiji | incidence | 2010 | 2014 | -0.98 (-1.22 to -0.73) | <0.001 |
| Fiji | incidence | 2014 | 2019 | 0.09 (-0.06 to 0.24) | 0.238 |
| Fiji | incidence | 2019 | 2021 | -0.84 (-1.34 to -0.34) | 0.003 |
| Finland | incidence | 1990 | 1995 | -0.06 (-0.17 to 0.04) | 0.221 |
| Finland | incidence | 1995 | 2000 | -1.52 (-1.64 to -1.41) | <0.001 |
| Finland | incidence | 2000 | 2006 | -0.43 (-0.51 to -0.36) | <0.001 |
| Finland | incidence | 2006 | 2010 | -2.78 (-2.97 to -2.59) | <0.001 |
| Finland | incidence | 2010 | 2018 | -3.63 (-3.69 to -3.56) | <0.001 |
| Finland | incidence | 2018 | 2021 | -2.50 (-2.82 to -2.19) | <0.001 |
| France | incidence | 1990 | 1995 | -0.34 (-0.40 to -0.28) | <0.001 |
| France | incidence | 1995 | 2000 | -0.75 (-0.83 to -0.68) | <0.001 |
| France | incidence | 2000 | 2005 | -0.25 (-0.32 to -0.18) | <0.001 |
| France | incidence | 2005 | 2014 | -1.56 (-1.58 to -1.53) | <0.001 |
| France | incidence | 2014 | 2019 | -0.26 (-0.35 to -0.18) | <0.001 |
| France | incidence | 2019 | 2021 | -1.54 (-1.85 to -1.23) | <0.001 |
| Gabon | incidence | 1990 | 2001 | 0.13 (0.10 to 0.16) | <0.001 |
| Gabon | incidence | 2001 | 2013 | -0.88 (-0.91 to -0.85) | <0.001 |
| Gabon | incidence | 2013 | 2019 | 0.07 (-0.03 to 0.17) | 0.176 |
| Gabon | incidence | 2019 | 2021 | -1.10 (-1.57 to -0.63) | <0.001 |
| Gambia | incidence | 1990 | 1994 | -0.77 (-0.86 to -0.68) | <0.001 |
| Gambia | incidence | 1994 | 2000 | 0.39 (0.33 to 0.45) | <0.001 |
| Gambia | incidence | 2000 | 2008 | -0.24 (-0.27 to -0.20) | <0.001 |
| Gambia | incidence | 2008 | 2015 | -0.42 (-0.46 to -0.38) | <0.001 |
| Gambia | incidence | 2015 | 2019 | 0.41 (0.29 to 0.53) | <0.001 |
| Gambia | incidence | 2019 | 2021 | -0.58 (-0.84 to -0.32) | <0.001 |
| Georgia | incidence | 1990 | 1994 | -1.84 (-1.95 to -1.73) | <0.001 |
| Georgia | incidence | 1994 | 2000 | -0.80 (-0.87 to -0.73) | <0.001 |
| Georgia | incidence | 2000 | 2005 | 0.61 (0.50 to 0.71) | <0.001 |
| Georgia | incidence | 2005 | 2010 | 1.58 (1.48 to 1.68) | <0.001 |
| Georgia | incidence | 2010 | 2019 | 0.69 (0.65 to 0.73) | <0.001 |
| Georgia | incidence | 2019 | 2021 | 0.13 (-0.22 to 0.48) | 0.434 |
| Germany | incidence | 1990 | 1994 | -1.98 (-2.08 to -1.89) | <0.001 |
| Germany | incidence | 1994 | 2001 | -1.66 (-1.70 to -1.61) | <0.001 |
| Germany | incidence | 2001 | 2009 | -2.47 (-2.50 to -2.44) | <0.001 |
| Germany | incidence | 2009 | 2015 | -0.77 (-0.83 to -0.72) | <0.001 |
| Germany | incidence | 2015 | 2019 | -1.72 (-1.85 to -1.59) | <0.001 |
| Germany | incidence | 2019 | 2021 | 0.72 (0.35 to 1.09) | 0.001 |
| Ghana | incidence | 1990 | 1995 | -0.15 (-0.34 to 0.05) | 0.138 |
| Ghana | incidence | 1995 | 2005 | 0.67 (0.59 to 0.74) | <0.001 |
| Ghana | incidence | 2005 | 2019 | -0.02 (-0.07 to 0.02) | 0.272 |
| Ghana | incidence | 2019 | 2021 | -1.78 (-2.59 to -0.96) | <0.001 |
| Greece | incidence | 1990 | 1995 | -0.59 (-1.08 to -0.10) | 0.021 |
| Greece | incidence | 1995 | 1999 | -2.26 (-3.22 to -1.29) | <0.001 |
| Greece | incidence | 1999 | 2006 | -1.16 (-1.50 to -0.83) | <0.001 |
| Greece | incidence | 2006 | 2010 | -5.39 (-6.37 to -4.39) | <0.001 |
| Greece | incidence | 2010 | 2014 | -2.89 (-3.94 to -1.83) | <0.001 |
| Greece | incidence | 2014 | 2021 | -0.31 (-0.62 to -0.01) | 0.043 |
| Greenland | incidence | 1990 | 1996 | -0.81 (-0.91 to -0.71) | <0.001 |
| Greenland | incidence | 1996 | 2001 | -3.05 (-3.23 to -2.86) | <0.001 |
| Greenland | incidence | 2001 | 2009 | -4.85 (-4.92 to -4.78) | <0.001 |
| Greenland | incidence | 2009 | 2014 | -2.63 (-2.80 to -2.46) | <0.001 |
| Greenland | incidence | 2014 | 2019 | 0.21 (0.03 to 0.38) | 0.023 |
| Greenland | incidence | 2019 | 2021 | -2.36 (-2.93 to -1.78) | <0.001 |
| Grenada | incidence | 1990 | 1995 | -0.80 (-0.92 to -0.69) | <0.001 |
| Grenada | incidence | 1995 | 2004 | -1.82 (-1.87 to -1.76) | <0.001 |
| Grenada | incidence | 2004 | 2010 | -0.75 (-0.87 to -0.63) | <0.001 |
| Grenada | incidence | 2010 | 2014 | 0.58 (0.33 to 0.83) | <0.001 |
| Grenada | incidence | 2014 | 2019 | 0.09 (-0.06 to 0.25) | 0.22 |
| Grenada | incidence | 2019 | 2021 | 0.87 (0.41 to 1.32) | 0.001 |
| Guam | incidence | 1990 | 1993 | 0.26 (0.03 to 0.49) | 0.029 |
| Guam | incidence | 1993 | 1996 | -0.35 (-0.81 to 0.12) | 0.136 |
| Guam | incidence | 1996 | 2001 | -1.87 (-2.01 to -1.74) | <0.001 |
| Guam | incidence | 2001 | 2009 | -1.48 (-1.53 to -1.42) | <0.001 |
| Guam | incidence | 2009 | 2021 | 0.14 (0.12 to 0.16) | <0.001 |
| Guatemala | incidence | 1990 | 1997 | -0.45 (-0.51 to -0.38) | <0.001 |
| Guatemala | incidence | 1997 | 2001 | -1.15 (-1.42 to -0.88) | <0.001 |
| Guatemala | incidence | 2001 | 2005 | -2.35 (-2.61 to -2.08) | <0.001 |
| Guatemala | incidence | 2005 | 2009 | -1.24 (-1.50 to -0.98) | <0.001 |
| Guatemala | incidence | 2009 | 2021 | -0.04 (-0.07 to -0.01) | 0.023 |
| Guinea | incidence | 1990 | 1994 | -0.36 (-0.50 to -0.22) | <0.001 |
| Guinea | incidence | 1994 | 2010 | 0.15 (0.13 to 0.17) | <0.001 |
| Guinea | incidence | 2010 | 2015 | -0.28 (-0.40 to -0.15) | <0.001 |
| Guinea | incidence | 2015 | 2019 | 0.54 (0.33 to 0.75) | <0.001 |
| Guinea | incidence | 2019 | 2021 | -0.87 (-1.27 to -0.47) | <0.001 |
| Guinea-Bissau | incidence | 1990 | 1995 | -0.20 (-0.32 to -0.07) | 0.004 |
| Guinea-Bissau | incidence | 1995 | 2001 | 0.32 (0.21 to 0.44) | <0.001 |
| Guinea-Bissau | incidence | 2001 | 2015 | -0.48 (-0.50 to -0.45) | <0.001 |
| Guinea-Bissau | incidence | 2015 | 2019 | 0.55 (0.32 to 0.78) | <0.001 |
| Guinea-Bissau | incidence | 2019 | 2021 | -1.55 (-2.06 to -1.03) | <0.001 |
| Guyana | incidence | 1990 | 2000 | -0.97 (-1.05 to -0.89) | <0.001 |
| Guyana | incidence | 2000 | 2005 | -1.95 (-2.24 to -1.65) | <0.001 |
| Guyana | incidence | 2005 | 2015 | -1.12 (-1.21 to -1.03) | <0.001 |
| Guyana | incidence | 2015 | 2018 | 0.33 (-0.65 to 1.33) | 0.487 |
| Guyana | incidence | 2018 | 2021 | -0.93 (-1.43 to -0.43) | 0.001 |
| Haiti | incidence | 1990 | 1996 | -0.56 (-0.58 to -0.54) | <0.001 |
| Haiti | incidence | 1996 | 2000 | -0.87 (-0.94 to -0.80) | <0.001 |
| Haiti | incidence | 2000 | 2009 | -0.32 (-0.34 to -0.31) | <0.001 |
| Haiti | incidence | 2009 | 2019 | -0.17 (-0.19 to -0.16) | <0.001 |
| Haiti | incidence | 2019 | 2021 | -0.01 (-0.16 to 0.13) | 0.84 |
| Honduras | incidence | 1990 | 1994 | 0.37 (0.25 to 0.49) | <0.001 |
| Honduras | incidence | 1994 | 1999 | 0.71 (0.60 to 0.82) | <0.001 |
| Honduras | incidence | 1999 | 2003 | 0.37 (0.18 to 0.55) | 0.001 |
| Honduras | incidence | 2003 | 2014 | -0.17 (-0.20 to -0.14) | <0.001 |
| Honduras | incidence | 2014 | 2021 | 0.10 (0.05 to 0.14) | <0.001 |
| Hungary | incidence | 1990 | 1996 | -1.80 (-1.87 to -1.73) | <0.001 |
| Hungary | incidence | 1996 | 2005 | -2.58 (-2.62 to -2.53) | <0.001 |
| Hungary | incidence | 2005 | 2014 | -3.34 (-3.38 to -3.30) | <0.001 |
| Hungary | incidence | 2014 | 2019 | -0.15 (-0.29 to -0.01) | 0.033 |
| Hungary | incidence | 2019 | 2021 | -1.64 (-2.11 to -1.18) | <0.001 |
| Iceland | incidence | 1990 | 1993 | -1.30 (-1.68 to -0.91) | <0.001 |
| Iceland | incidence | 1993 | 1996 | -1.99 (-2.77 to -1.20) | <0.001 |
| Iceland | incidence | 1996 | 2000 | -3.78 (-4.08 to -3.48) | <0.001 |
| Iceland | incidence | 2000 | 2011 | -2.88 (-2.93 to -2.84) | <0.001 |
| Iceland | incidence | 2011 | 2015 | -2.03 (-2.34 to -1.73) | <0.001 |
| Iceland | incidence | 2015 | 2021 | -0.89 (-1.01 to -0.76) | <0.001 |
| India | incidence | 1990 | 1995 | -0.34 (-0.42 to -0.25) | <0.001 |
| India | incidence | 1995 | 2000 | -1.59 (-1.70 to -1.48) | <0.001 |
| India | incidence | 2000 | 2009 | -0.92 (-0.96 to -0.88) | <0.001 |
| India | incidence | 2009 | 2015 | -1.39 (-1.46 to -1.32) | <0.001 |
| India | incidence | 2015 | 2019 | 1.17 (1.01 to 1.33) | <0.001 |
| India | incidence | 2019 | 2021 | 0.29 (-0.04 to 0.62) | 0.079 |
| Indonesia | incidence | 1990 | 1995 | 0.30 (0.20 to 0.39) | <0.001 |
| Indonesia | incidence | 1995 | 2000 | 0.90 (0.77 to 1.03) | <0.001 |
| Indonesia | incidence | 2000 | 2010 | 0.73 (0.70 to 0.77) | <0.001 |
| Indonesia | incidence | 2010 | 2017 | 0.33 (0.26 to 0.39) | <0.001 |
| Indonesia | incidence | 2017 | 2021 | 0.07 (-0.05 to 0.19) | 0.22 |
| Iran (Islamic Republic of) | incidence | 1990 | 1994 | -2.20 (-2.50 to -1.90) | <0.001 |
| Iran (Islamic Republic of) | incidence | 1994 | 2001 | 0.28 (0.13 to 0.44) | 0.001 |
| Iran (Islamic Republic of) | incidence | 2001 | 2005 | -0.79 (-1.24 to -0.33) | 0.002 |
| Iran (Islamic Republic of) | incidence | 2005 | 2010 | -2.74 (-3.02 to -2.46) | <0.001 |
| Iran (Islamic Republic of) | incidence | 2010 | 2015 | 0.25 (-0.02 to 0.53) | 0.069 |
| Iran (Islamic Republic of) | incidence | 2015 | 2021 | -2.13 (-2.27 to -1.99) | <0.001 |
| Iraq | incidence | 1990 | 1995 | -0.56 (-0.67 to -0.44) | <0.001 |
| Iraq | incidence | 1995 | 2001 | 0.54 (0.43 to 0.66) | <0.001 |
| Iraq | incidence | 2001 | 2013 | 0.08 (0.04 to 0.11) | <0.001 |
| Iraq | incidence | 2013 | 2019 | 0.38 (0.27 to 0.49) | <0.001 |
| Iraq | incidence | 2019 | 2021 | -0.71 (-1.23 to -0.18) | 0.012 |
| Ireland | incidence | 1990 | 1995 | -1.49 (-1.92 to -1.07) | <0.001 |
| Ireland | incidence | 1995 | 2010 | -5.82 (-5.90 to -5.74) | <0.001 |
| Ireland | incidence | 2010 | 2015 | -2.89 (-3.48 to -2.29) | <0.001 |
| Ireland | incidence | 2015 | 2019 | 3.53 (2.52 to 4.56) | <0.001 |
| Ireland | incidence | 2019 | 2021 | -0.49 (-2.57 to 1.63) | 0.63 |
| Israel | incidence | 1990 | 1995 | -0.50 (-0.63 to -0.37) | <0.001 |
| Israel | incidence | 1995 | 2005 | -2.06 (-2.09 to -2.02) | <0.001 |
| Israel | incidence | 2005 | 2010 | -1.53 (-1.65 to -1.42) | <0.001 |
| Israel | incidence | 2010 | 2015 | -3.51 (-3.65 to -3.37) | <0.001 |
| Israel | incidence | 2015 | 2019 | -2.28 (-2.54 to -2.02) | <0.001 |
| Israel | incidence | 2019 | 2021 | -8.79 (-9.49 to -8.09) | <0.001 |
| Italy | incidence | 1990 | 1994 | -4.72 (-4.99 to -4.45) | <0.001 |
| Italy | incidence | 1994 | 2002 | -3.03 (-3.12 to -2.95) | <0.001 |
| Italy | incidence | 2002 | 2010 | -3.45 (-3.52 to -3.38) | <0.001 |
| Italy | incidence | 2010 | 2014 | -2.29 (-2.53 to -2.04) | <0.001 |
| Italy | incidence | 2014 | 2021 | -0.23 (-0.30 to -0.16) | <0.001 |
| Jamaica | incidence | 1990 | 1995 | -0.55 (-0.69 to -0.42) | <0.001 |
| Jamaica | incidence | 1995 | 2000 | -1.80 (-1.98 to -1.61) | <0.001 |
| Jamaica | incidence | 2000 | 2004 | -1.24 (-1.53 to -0.95) | <0.001 |
| Jamaica | incidence | 2004 | 2008 | -0.51 (-0.81 to -0.20) | 0.003 |
| Jamaica | incidence | 2008 | 2019 | 0.05 (0.00 to 0.10) | 0.038 |
| Jamaica | incidence | 2019 | 2021 | 0.61 (0.02 to 1.19) | 0.043 |
| Japan | incidence | 1990 | 1996 | -0.72 (-1.22 to -0.21) | 0.009 |
| Japan | incidence | 1996 | 2000 | -4.48 (-5.75 to -3.21) | <0.001 |
| Japan | incidence | 2000 | 2005 | -2.13 (-2.86 to -1.40) | <0.001 |
| Japan | incidence | 2005 | 2010 | -3.23 (-3.89 to -2.57) | <0.001 |
| Japan | incidence | 2010 | 2014 | -1.88 (-2.90 to -0.85) | 0.001 |
| Japan | incidence | 2014 | 2021 | 0.67 (0.39 to 0.95) | <0.001 |
| Jordan | incidence | 1990 | 1995 | -0.25 (-0.42 to -0.07) | 0.01 |
| Jordan | incidence | 1995 | 2000 | -0.73 (-0.97 to -0.49) | <0.001 |
| Jordan | incidence | 2000 | 2005 | 1.87 (1.65 to 2.10) | <0.001 |
| Jordan | incidence | 2005 | 2014 | -2.35 (-2.42 to -2.28) | <0.001 |
| Jordan | incidence | 2014 | 2019 | -0.06 (-0.29 to 0.17) | 0.566 |
| Jordan | incidence | 2019 | 2021 | -1.33 (-2.08 to -0.56) | 0.002 |
| Kazakhstan | incidence | 1990 | 1994 | -0.13 (-0.38 to 0.12) | 0.288 |
| Kazakhstan | incidence | 1994 | 2001 | 0.68 (0.55 to 0.80) | <0.001 |
| Kazakhstan | incidence | 2001 | 2009 | -1.08 (-1.18 to -0.99) | <0.001 |
| Kazakhstan | incidence | 2009 | 2014 | -2.68 (-2.92 to -2.44) | <0.001 |
| Kazakhstan | incidence | 2014 | 2019 | 0.19 (-0.06 to 0.44) | 0.129 |
| Kazakhstan | incidence | 2019 | 2021 | -2.74 (-3.51 to -1.96) | <0.001 |
| Kenya | incidence | 1990 | 1992 | -0.80 (-1.26 to -0.34) | 0.002 |
| Kenya | incidence | 1992 | 1995 | -0.27 (-0.71 to 0.16) | 0.199 |
| Kenya | incidence | 1995 | 2000 | 0.76 (0.62 to 0.89) | <0.001 |
| Kenya | incidence | 2000 | 2003 | 0.01 (-0.42 to 0.43) | 0.97 |
| Kenya | incidence | 2003 | 2015 | -0.28 (-0.31 to -0.26) | <0.001 |
| Kenya | incidence | 2015 | 2021 | 0.24 (0.17 to 0.31) | <0.001 |
| Kiribati | incidence | 1990 | 1995 | 0.35 (0.30 to 0.41) | <0.001 |
| Kiribati | incidence | 1995 | 2001 | -0.16 (-0.21 to -0.11) | <0.001 |
| Kiribati | incidence | 2001 | 2007 | -0.40 (-0.46 to -0.35) | <0.001 |
| Kiribati | incidence | 2007 | 2015 | -0.56 (-0.59 to -0.53) | <0.001 |
| Kiribati | incidence | 2015 | 2019 | 0.06 (-0.05 to 0.16) | 0.294 |
| Kiribati | incidence | 2019 | 2021 | -0.67 (-0.90 to -0.43) | <0.001 |
| Kuwait | incidence | 1990 | 1994 | 0.53 (-0.18 to 1.24) | 0.131 |
| Kuwait | incidence | 1994 | 2003 | 1.67 (1.44 to 1.91) | <0.001 |
| Kuwait | incidence | 2003 | 2008 | -0.36 (-1.08 to 0.37) | 0.312 |
| Kuwait | incidence | 2008 | 2015 | -2.30 (-2.66 to -1.94) | <0.001 |
| Kuwait | incidence | 2015 | 2018 | -0.60 (-2.71 to 1.55) | 0.556 |
| Kuwait | incidence | 2018 | 2021 | -2.55 (-3.68 to -1.41) | <0.001 |
| Kyrgyzstan | incidence | 1990 | 2001 | -0.42 (-0.49 to -0.35) | <0.001 |
| Kyrgyzstan | incidence | 2001 | 2015 | -2.34 (-2.39 to -2.29) | <0.001 |
| Kyrgyzstan | incidence | 2015 | 2019 | 0.64 (0.13 to 1.16) | 0.017 |
| Kyrgyzstan | incidence | 2019 | 2021 | -2.79 (-3.81 to -1.76) | <0.001 |
| Lao People's Democratic Republic | incidence | 1990 | 2001 | -0.15 (-0.17 to -0.13) | <0.001 |
| Lao People's Democratic Republic | incidence | 2001 | 2006 | -0.54 (-0.62 to -0.46) | <0.001 |
| Lao People's Democratic Republic | incidence | 2006 | 2010 | -0.88 (-1.00 to -0.75) | <0.001 |
| Lao People's Democratic Republic | incidence | 2010 | 2015 | -0.32 (-0.40 to -0.25) | <0.001 |
| Lao People's Democratic Republic | incidence | 2015 | 2019 | 0.76 (0.64 to 0.88) | <0.001 |
| Lao People's Democratic Republic | incidence | 2019 | 2021 | -0.76 (-1.02 to -0.51) | <0.001 |
| Latvia | incidence | 1990 | 1996 | -1.45 (-1.63 to -1.26) | <0.001 |
| Latvia | incidence | 1996 | 1999 | -2.14 (-2.98 to -1.30) | <0.001 |
| Latvia | incidence | 1999 | 2010 | -0.75 (-0.81 to -0.68) | <0.001 |
| Latvia | incidence | 2010 | 2014 | -1.91 (-2.30 to -1.52) | <0.001 |
| Latvia | incidence | 2014 | 2021 | -1.39 (-1.52 to -1.26) | <0.001 |
| Lebanon | incidence | 1990 | 1999 | -0.87 (-0.89 to -0.85) | <0.001 |
| Lebanon | incidence | 1999 | 2009 | -0.24 (-0.26 to -0.23) | <0.001 |
| Lebanon | incidence | 2009 | 2014 | -0.61 (-0.66 to -0.55) | <0.001 |
| Lebanon | incidence | 2014 | 2019 | 0.01 (-0.05 to 0.06) | 0.857 |
| Lebanon | incidence | 2019 | 2021 | -1.38 (-1.57 to -1.19) | <0.001 |
| Lesotho | incidence | 1990 | 1996 | 0.65 (0.44 to 0.85) | <0.001 |
| Lesotho | incidence | 1996 | 2009 | 2.35 (2.29 to 2.42) | <0.001 |
| Lesotho | incidence | 2009 | 2021 | 0.07 (0.01 to 0.14) | 0.036 |
| Liberia | incidence | 1990 | 1994 | -1.62 (-1.70 to -1.55) | <0.001 |
| Liberia | incidence | 1994 | 1999 | -0.91 (-0.98 to -0.84) | <0.001 |
| Liberia | incidence | 1999 | 2005 | -0.43 (-0.48 to -0.38) | <0.001 |
| Liberia | incidence | 2005 | 2014 | -0.72 (-0.74 to -0.69) | <0.001 |
| Liberia | incidence | 2014 | 2019 | 0.13 (0.06 to 0.19) | 0.001 |
| Liberia | incidence | 2019 | 2021 | -0.92 (-1.14 to -0.70) | <0.001 |
| Libya | incidence | 1990 | 2001 | 0.38 (0.36 to 0.40) | <0.001 |
| Libya | incidence | 2001 | 2009 | 0.74 (0.69 to 0.78) | <0.001 |
| Libya | incidence | 2009 | 2014 | 0.46 (0.37 to 0.56) | <0.001 |
| Libya | incidence | 2014 | 2019 | -0.14 (-0.24 to -0.05) | 0.005 |
| Libya | incidence | 2019 | 2021 | 1.12 (0.80 to 1.44) | <0.001 |
| Lithuania | incidence | 1990 | 1995 | 0.80 (0.21 to 1.39) | 0.011 |
| Lithuania | incidence | 1995 | 2000 | -0.42 (-0.94 to 0.10) | 0.103 |
| Lithuania | incidence | 2000 | 2005 | 0.69 (0.24 to 1.13) | 0.005 |
| Lithuania | incidence | 2005 | 2010 | -0.64 (-1.08 to -0.20) | 0.008 |
| Lithuania | incidence | 2010 | 2014 | -2.21 (-3.13 to -1.28) | <0.001 |
| Lithuania | incidence | 2014 | 2021 | -4.17 (-4.58 to -3.77) | <0.001 |
| Luxembourg | incidence | 1990 | 1996 | -3.93 (-4.04 to -3.81) | <0.001 |
| Luxembourg | incidence | 1996 | 2004 | -5.38 (-5.46 to -5.31) | <0.001 |
| Luxembourg | incidence | 2004 | 2009 | -3.63 (-3.79 to -3.47) | <0.001 |
| Luxembourg | incidence | 2009 | 2015 | -1.03 (-1.13 to -0.94) | <0.001 |
| Luxembourg | incidence | 2015 | 2019 | 1.88 (1.64 to 2.13) | <0.001 |
| Luxembourg | incidence | 2019 | 2021 | -2.05 (-2.67 to -1.43) | <0.001 |
| Madagascar | incidence | 1990 | 1995 | 0.09 (-0.08 to 0.25) | 0.284 |
| Madagascar | incidence | 1995 | 2000 | 0.67 (0.45 to 0.89) | <0.001 |
| Madagascar | incidence | 2000 | 2015 | -0.33 (-0.36 to -0.30) | <0.001 |
| Madagascar | incidence | 2015 | 2019 | 0.36 (0.04 to 0.68) | 0.028 |
| Madagascar | incidence | 2019 | 2021 | -0.79 (-1.43 to -0.14) | 0.02 |
| Malawi | incidence | 1990 | 1995 | -0.13 (-0.19 to -0.08) | <0.001 |
| Malawi | incidence | 1995 | 2000 | 0.80 (0.72 to 0.87) | <0.001 |
| Malawi | incidence | 2000 | 2015 | -0.57 (-0.58 to -0.56) | <0.001 |
| Malawi | incidence | 2015 | 2019 | 0.35 (0.24 to 0.46) | <0.001 |
| Malawi | incidence | 2019 | 2021 | -0.78 (-1.01 to -0.55) | <0.001 |
| Malaysia | incidence | 1990 | 1999 | -1.41 (-1.42 to -1.40) | <0.001 |
| Malaysia | incidence | 1999 | 2004 | -0.90 (-0.95 to -0.86) | <0.001 |
| Malaysia | incidence | 2004 | 2015 | -0.38 (-0.39 to -0.37) | <0.001 |
| Malaysia | incidence | 2015 | 2019 | 0.23 (0.17 to 0.29) | <0.001 |
| Malaysia | incidence | 2019 | 2021 | -0.90 (-1.03 to -0.76) | <0.001 |
| Maldives | incidence | 1990 | 1996 | -0.92 (-1.00 to -0.83) | <0.001 |
| Maldives | incidence | 1996 | 2001 | -2.39 (-2.54 to -2.24) | <0.001 |
| Maldives | incidence | 2001 | 2010 | -3.00 (-3.05 to -2.95) | <0.001 |
| Maldives | incidence | 2010 | 2014 | -1.58 (-1.81 to -1.35) | <0.001 |
| Maldives | incidence | 2014 | 2019 | 0.33 (0.17 to 0.48) | <0.001 |
| Maldives | incidence | 2019 | 2021 | -0.69 (-1.16 to -0.22) | 0.007 |
| Mali | incidence | 1990 | 1994 | -1.07 (-1.18 to -0.95) | <0.001 |
| Mali | incidence | 1994 | 2001 | -0.21 (-0.27 to -0.15) | <0.001 |
| Mali | incidence | 2001 | 2005 | -0.54 (-0.71 to -0.38) | <0.001 |
| Mali | incidence | 2005 | 2014 | -1.14 (-1.18 to -1.11) | <0.001 |
| Mali | incidence | 2014 | 2019 | 0.43 (0.32 to 0.53) | <0.001 |
| Mali | incidence | 2019 | 2021 | -0.92 (-1.24 to -0.59) | <0.001 |
| Malta | incidence | 1990 | 1995 | -3.72 (-4.27 to -3.17) | <0.001 |
| Malta | incidence | 1995 | 1999 | -6.19 (-7.24 to -5.12) | <0.001 |
| Malta | incidence | 1999 | 2012 | -3.77 (-3.89 to -3.64) | <0.001 |
| Malta | incidence | 2012 | 2015 | -1.53 (-3.75 to 0.75) | 0.171 |
| Malta | incidence | 2015 | 2019 | 3.15 (1.97 to 4.34) | <0.001 |
| Malta | incidence | 2019 | 2021 | -2.57 (-5.06 to -0.02) | 0.048 |
| Marshall Islands | incidence | 1990 | 1998 | 0.68 (0.64 to 0.73) | <0.001 |
| Marshall Islands | incidence | 1998 | 2006 | -0.04 (-0.09 to 0.01) | 0.104 |
| Marshall Islands | incidence | 2006 | 2015 | -0.77 (-0.81 to -0.73) | <0.001 |
| Marshall Islands | incidence | 2015 | 2019 | 0.08 (-0.09 to 0.26) | 0.32 |
| Marshall Islands | incidence | 2019 | 2021 | -1.36 (-1.73 to -0.98) | <0.001 |
| Mauritania | incidence | 1990 | 1996 | -1.54 (-1.56 to -1.51) | <0.001 |
| Mauritania | incidence | 1996 | 2004 | -1.76 (-1.78 to -1.74) | <0.001 |
| Mauritania | incidence | 2004 | 2009 | -1.21 (-1.26 to -1.17) | <0.001 |
| Mauritania | incidence | 2009 | 2014 | -0.65 (-0.69 to -0.60) | <0.001 |
| Mauritania | incidence | 2014 | 2019 | 0.32 (0.27 to 0.37) | <0.001 |
| Mauritania | incidence | 2019 | 2021 | -0.64 (-0.79 to -0.49) | <0.001 |
| Mauritius | incidence | 1990 | 1996 | 0.20 (-0.04 to 0.43) | 0.093 |
| Mauritius | incidence | 1996 | 2002 | -2.20 (-2.49 to -1.91) | <0.001 |
| Mauritius | incidence | 2002 | 2010 | -5.08 (-5.25 to -4.92) | <0.001 |
| Mauritius | incidence | 2010 | 2014 | -3.22 (-3.83 to -2.60) | <0.001 |
| Mauritius | incidence | 2014 | 2019 | 0.42 (-0.02 to 0.86) | 0.061 |
| Mauritius | incidence | 2019 | 2021 | -1.18 (-2.63 to 0.28) | 0.106 |
| Mexico | incidence | 1990 | 1995 | -1.24 (-1.38 to -1.09) | <0.001 |
| Mexico | incidence | 1995 | 2000 | -2.85 (-3.04 to -2.65) | <0.001 |
| Mexico | incidence | 2000 | 2010 | -2.42 (-2.47 to -2.36) | <0.001 |
| Mexico | incidence | 2010 | 2015 | -1.03 (-1.23 to -0.84) | <0.001 |
| Mexico | incidence | 2015 | 2019 | 1.13 (0.82 to 1.45) | <0.001 |
| Mexico | incidence | 2019 | 2021 | -0.39 (-1.02 to 0.25) | 0.216 |
| Micronesia (Federated States of) | incidence | 1990 | 1996 | 0.19 (0.13 to 0.26) | <0.001 |
| Micronesia (Federated States of) | incidence | 1996 | 2011 | -0.39 (-0.41 to -0.38) | <0.001 |
| Micronesia (Federated States of) | incidence | 2011 | 2019 | -0.12 (-0.17 to -0.08) | <0.001 |
| Micronesia (Federated States of) | incidence | 2019 | 2021 | -0.74 (-1.11 to -0.37) | <0.001 |
| Monaco | incidence | 1990 | 1996 | -1.43 (-1.55 to -1.31) | <0.001 |
| Monaco | incidence | 1996 | 2005 | -3.54 (-3.61 to -3.47) | <0.001 |
| Monaco | incidence | 2005 | 2009 | -2.60 (-2.95 to -2.25) | <0.001 |
| Monaco | incidence | 2009 | 2014 | -1.58 (-1.80 to -1.36) | <0.001 |
| Monaco | incidence | 2014 | 2019 | 0.29 (0.08 to 0.51) | 0.011 |
| Monaco | incidence | 2019 | 2021 | -1.11 (-1.85 to -0.36) | 0.006 |
| Mongolia | incidence | 1990 | 1995 | -0.12 (-0.34 to 0.10) | 0.254 |
| Mongolia | incidence | 1995 | 2001 | 2.61 (2.40 to 2.81) | <0.001 |
| Mongolia | incidence | 2001 | 2006 | 0.91 (0.63 to 1.20) | <0.001 |
| Mongolia | incidence | 2006 | 2015 | -0.73 (-0.82 to -0.63) | <0.001 |
| Mongolia | incidence | 2015 | 2019 | 0.72 (0.28 to 1.16) | 0.003 |
| Mongolia | incidence | 2019 | 2021 | -1.20 (-2.13 to -0.25) | 0.017 |
| Montenegro | incidence | 1990 | 1994 | -2.15 (-2.40 to -1.91) | <0.001 |
| Montenegro | incidence | 1994 | 1999 | 0.47 (0.22 to 0.72) | 0.001 |
| Montenegro | incidence | 1999 | 2007 | 1.40 (1.30 to 1.50) | <0.001 |
| Montenegro | incidence | 2007 | 2010 | 0.79 (0.07 to 1.51) | 0.034 |
| Montenegro | incidence | 2010 | 2015 | -1.26 (-1.47 to -1.05) | <0.001 |
| Montenegro | incidence | 2015 | 2021 | 0.40 (0.28 to 0.52) | <0.001 |
| Morocco | incidence | 1990 | 1995 | 0.08 (0.04 to 0.12) | <0.001 |
| Morocco | incidence | 1995 | 2000 | 0.65 (0.60 to 0.71) | <0.001 |
| Morocco | incidence | 2000 | 2010 | 0.26 (0.25 to 0.28) | <0.001 |
| Morocco | incidence | 2010 | 2015 | -0.36 (-0.41 to -0.31) | <0.001 |
| Morocco | incidence | 2015 | 2019 | 0.84 (0.75 to 0.92) | <0.001 |
| Morocco | incidence | 2019 | 2021 | -0.57 (-0.73 to -0.40) | <0.001 |
| Mozambique | incidence | 1990 | 1995 | -0.62 (-0.78 to -0.46) | <0.001 |
| Mozambique | incidence | 1995 | 1999 | -0.11 (-0.46 to 0.24) | 0.51 |
| Mozambique | incidence | 1999 | 2005 | 0.55 (0.39 to 0.71) | <0.001 |
| Mozambique | incidence | 2005 | 2009 | 1.51 (1.17 to 1.86) | <0.001 |
| Mozambique | incidence | 2009 | 2019 | 0.67 (0.60 to 0.73) | <0.001 |
| Mozambique | incidence | 2019 | 2021 | -0.12 (-0.81 to 0.58) | 0.73 |
| Myanmar | incidence | 1990 | 2005 | 0.10 (0.06 to 0.13) | <0.001 |
| Myanmar | incidence | 2005 | 2015 | -1.34 (-1.41 to -1.27) | <0.001 |
| Myanmar | incidence | 2015 | 2019 | 0.56 (0.17 to 0.95) | 0.007 |
| Myanmar | incidence | 2019 | 2021 | -1.66 (-2.49 to -0.82) | 0.001 |
| Namibia | incidence | 1990 | 1995 | 0.44 (0.24 to 0.64) | <0.001 |
| Namibia | incidence | 1995 | 2001 | -0.08 (-0.27 to 0.10) | 0.357 |
| Namibia | incidence | 2001 | 2013 | -0.82 (-0.88 to -0.77) | <0.001 |
| Namibia | incidence | 2013 | 2021 | -0.17 (-0.26 to -0.08) | 0.001 |
| Nauru | incidence | 1990 | 1995 | 0.09 (-0.04 to 0.22) | 0.157 |
| Nauru | incidence | 1995 | 2003 | -1.23 (-1.30 to -1.16) | <0.001 |
| Nauru | incidence | 2003 | 2010 | -1.95 (-2.04 to -1.87) | <0.001 |
| Nauru | incidence | 2010 | 2014 | -0.33 (-0.58 to -0.08) | 0.015 |
| Nauru | incidence | 2014 | 2019 | 0.38 (0.22 to 0.54) | <0.001 |
| Nauru | incidence | 2019 | 2021 | -0.96 (-1.49 to -0.42) | 0.002 |
| Nepal | incidence | 1990 | 1995 | -0.60 (-0.70 to -0.50) | <0.001 |
| Nepal | incidence | 1995 | 2000 | -1.13 (-1.26 to -1.00) | <0.001 |
| Nepal | incidence | 2000 | 2004 | -0.53 (-0.75 to -0.32) | <0.001 |
| Nepal | incidence | 2004 | 2010 | -0.23 (-0.32 to -0.13) | <0.001 |
| Nepal | incidence | 2010 | 2016 | 0.00 (-0.09 to 0.09) | 0.973 |
| Nepal | incidence | 2016 | 2021 | 0.58 (0.49 to 0.67) | <0.001 |
| Netherlands | incidence | 1990 | 1996 | 0.10 (-0.02 to 0.22) | 0.105 |
| Netherlands | incidence | 1996 | 2001 | -1.52 (-1.75 to -1.30) | <0.001 |
| Netherlands | incidence | 2001 | 2005 | -4.09 (-4.44 to -3.74) | <0.001 |
| Netherlands | incidence | 2005 | 2010 | -5.44 (-5.68 to -5.20) | <0.001 |
| Netherlands | incidence | 2010 | 2014 | -2.74 (-3.16 to -2.31) | <0.001 |
| Netherlands | incidence | 2014 | 2021 | -0.85 (-0.98 to -0.73) | <0.001 |
| New Zealand | incidence | 1990 | 1995 | -2.73 (-2.80 to -2.65) | <0.001 |
| New Zealand | incidence | 1995 | 2002 | -2.19 (-2.24 to -2.14) | <0.001 |
| New Zealand | incidence | 2002 | 2009 | -1.92 (-1.96 to -1.88) | <0.001 |
| New Zealand | incidence | 2009 | 2014 | -1.69 (-1.76 to -1.61) | <0.001 |
| New Zealand | incidence | 2014 | 2018 | -0.75 (-0.88 to -0.63) | <0.001 |
| New Zealand | incidence | 2018 | 2021 | 0.55 (0.41 to 0.69) | <0.001 |
| Nicaragua | incidence | 1990 | 1993 | -1.01 (-1.18 to -0.84) | <0.001 |
| Nicaragua | incidence | 1993 | 2001 | -0.40 (-0.45 to -0.36) | <0.001 |
| Nicaragua | incidence | 2001 | 2009 | -2.48 (-2.52 to -2.44) | <0.001 |
| Nicaragua | incidence | 2009 | 2014 | -1.56 (-1.66 to -1.46) | <0.001 |
| Nicaragua | incidence | 2014 | 2019 | 0.10 (-0.01 to 0.20) | 0.064 |
| Nicaragua | incidence | 2019 | 2021 | -1.34 (-1.68 to -1.00) | <0.001 |
| Niger | incidence | 1990 | 1993 | -1.06 (-1.13 to -0.98) | <0.001 |
| Niger | incidence | 1993 | 2001 | -0.67 (-0.69 to -0.65) | <0.001 |
| Niger | incidence | 2001 | 2010 | -1.11 (-1.13 to -1.10) | <0.001 |
| Niger | incidence | 2010 | 2015 | -0.39 (-0.43 to -0.35) | <0.001 |
| Niger | incidence | 2015 | 2019 | 0.60 (0.53 to 0.66) | <0.001 |
| Niger | incidence | 2019 | 2021 | -0.17 (-0.32 to -0.03) | 0.025 |
| Nigeria | incidence | 1990 | 1994 | -0.97 (-1.04 to -0.90) | <0.001 |
| Nigeria | incidence | 1994 | 2005 | -0.06 (-0.07 to -0.04) | <0.001 |
| Nigeria | incidence | 2005 | 2014 | -0.82 (-0.84 to -0.80) | <0.001 |
| Nigeria | incidence | 2014 | 2019 | 0.38 (0.32 to 0.44) | <0.001 |
| Nigeria | incidence | 2019 | 2021 | -0.79 (-0.99 to -0.59) | <0.001 |
| Niue | incidence | 1990 | 1995 | 0.26 (0.12 to 0.40) | 0.001 |
| Niue | incidence | 1995 | 2001 | -0.17 (-0.31 to -0.04) | 0.016 |
| Niue | incidence | 2001 | 2015 | -1.05 (-1.08 to -1.02) | <0.001 |
| Niue | incidence | 2015 | 2018 | 0.32 (-0.29 to 0.94) | 0.282 |
| Niue | incidence | 2018 | 2021 | -0.72 (-1.03 to -0.41) | <0.001 |
| North Macedonia | incidence | 1990 | 2011 | 0.06 (0.03 to 0.09) | <0.001 |
| North Macedonia | incidence | 2011 | 2014 | -1.21 (-2.23 to -0.19) | 0.023 |
| North Macedonia | incidence | 2014 | 2021 | -0.13 (-0.27 to 0.01) | 0.06 |
| Northern Mariana Islands | incidence | 1990 | 1999 | -0.03 (-0.07 to 0.01) | 0.151 |
| Northern Mariana Islands | incidence | 1999 | 2010 | -0.54 (-0.57 to -0.50) | <0.001 |
| Northern Mariana Islands | incidence | 2010 | 2014 | -1.13 (-1.34 to -0.91) | <0.001 |
| Northern Mariana Islands | incidence | 2014 | 2021 | 0.29 (0.23 to 0.36) | <0.001 |
| Norway | incidence | 1990 | 1995 | -1.22 (-1.55 to -0.88) | <0.001 |
| Norway | incidence | 1995 | 2000 | -4.29 (-4.67 to -3.90) | <0.001 |
| Norway | incidence | 2000 | 2011 | -2.83 (-2.91 to -2.74) | <0.001 |
| Norway | incidence | 2011 | 2015 | -1.59 (-2.14 to -1.04) | <0.001 |
| Norway | incidence | 2015 | 2019 | 1.47 (0.89 to 2.06) | <0.001 |
| Norway | incidence | 2019 | 2021 | -0.26 (-1.44 to 0.92) | 0.642 |
| Oman | incidence | 1990 | 2000 | 0.04 (-0.02 to 0.09) | 0.176 |
| Oman | incidence | 2000 | 2006 | 0.85 (0.70 to 1.01) | <0.001 |
| Oman | incidence | 2006 | 2010 | -0.01 (-0.32 to 0.30) | 0.96 |
| Oman | incidence | 2010 | 2014 | -1.30 (-1.57 to -1.03) | <0.001 |
| Oman | incidence | 2014 | 2019 | -0.22 (-0.40 to -0.05) | 0.017 |
| Oman | incidence | 2019 | 2021 | -1.57 (-2.19 to -0.94) | <0.001 |
| Pakistan | incidence | 1990 | 1995 | 0.25 (0.16 to 0.35) | <0.001 |
| Pakistan | incidence | 1995 | 2000 | 0.66 (0.52 to 0.79) | <0.001 |
| Pakistan | incidence | 2000 | 2005 | -0.23 (-0.36 to -0.10) | 0.002 |
| Pakistan | incidence | 2005 | 2014 | -0.79 (-0.84 to -0.75) | <0.001 |
| Pakistan | incidence | 2014 | 2019 | 0.30 (0.18 to 0.42) | <0.001 |
| Pakistan | incidence | 2019 | 2021 | -0.47 (-0.87 to -0.08) | 0.022 |
| Palau | incidence | 1990 | 1994 | 0.88 (0.76 to 1.00) | <0.001 |
| Palau | incidence | 1994 | 2000 | 0.13 (0.05 to 0.21) | 0.003 |
| Palau | incidence | 2000 | 2013 | -0.43 (-0.45 to -0.41) | <0.001 |
| Palau | incidence | 2013 | 2019 | 0.03 (-0.05 to 0.10) | 0.477 |
| Palau | incidence | 2019 | 2021 | -1.29 (-1.64 to -0.94) | <0.001 |
| Palestine | incidence | 1990 | 1995 | -0.66 (-1.08 to -0.24) | 0.005 |
| Palestine | incidence | 1995 | 2001 | -1.75 (-2.12 to -1.38) | <0.001 |
| Palestine | incidence | 2001 | 2011 | 0.81 (0.67 to 0.95) | <0.001 |
| Palestine | incidence | 2011 | 2015 | 0.23 (-0.54 to 1.00) | 0.537 |
| Palestine | incidence | 2015 | 2019 | 1.31 (0.57 to 2.05) | 0.002 |
| Palestine | incidence | 2019 | 2021 | -1.59 (-3.15 to -0.01) | 0.048 |
| Panama | incidence | 1990 | 2000 | -0.84 (-0.86 to -0.82) | <0.001 |
| Panama | incidence | 2000 | 2010 | -1.92 (-1.94 to -1.90) | <0.001 |
| Panama | incidence | 2010 | 2014 | -1.16 (-1.28 to -1.04) | <0.001 |
| Panama | incidence | 2014 | 2019 | 0.13 (0.06 to 0.21) | 0.001 |
| Panama | incidence | 2019 | 2021 | -0.92 (-1.16 to -0.67) | <0.001 |
| Papua New Guinea | incidence | 1990 | 1995 | -0.20 (-0.24 to -0.15) | <0.001 |
| Papua New Guinea | incidence | 1995 | 2000 | 0.32 (0.26 to 0.39) | <0.001 |
| Papua New Guinea | incidence | 2000 | 2005 | -0.23 (-0.29 to -0.16) | <0.001 |
| Papua New Guinea | incidence | 2005 | 2015 | -0.62 (-0.64 to -0.60) | <0.001 |
| Papua New Guinea | incidence | 2015 | 2019 | 0.41 (0.32 to 0.50) | <0.001 |
| Papua New Guinea | incidence | 2019 | 2021 | -0.22 (-0.42 to -0.02) | 0.035 |
| Paraguay | incidence | 1990 | 1999 | -0.66 (-0.73 to -0.60) | <0.001 |
| Paraguay | incidence | 1999 | 2008 | -1.27 (-1.35 to -1.20) | <0.001 |
| Paraguay | incidence | 2008 | 2014 | -0.81 (-0.97 to -0.65) | <0.001 |
| Paraguay | incidence | 2014 | 2018 | 0.15 (-0.20 to 0.50) | 0.379 |
| Paraguay | incidence | 2018 | 2021 | -0.42 (-0.77 to -0.07) | 0.022 |
| Peru | incidence | 1990 | 1995 | -1.26 (-1.43 to -1.08) | <0.001 |
| Peru | incidence | 1995 | 1999 | -2.35 (-2.73 to -1.97) | <0.001 |
| Peru | incidence | 1999 | 2010 | -1.84 (-1.90 to -1.78) | <0.001 |
| Peru | incidence | 2010 | 2019 | 0.09 (0.01 to 0.18) | 0.039 |
| Peru | incidence | 2019 | 2021 | -0.74 (-1.56 to 0.09) | 0.078 |
| Philippines | incidence | 1990 | 1995 | 0.72 (0.52 to 0.93) | <0.001 |
| Philippines | incidence | 1995 | 2003 | 2.44 (2.33 to 2.55) | <0.001 |
| Philippines | incidence | 2003 | 2006 | 1.12 (0.30 to 1.94) | 0.011 |
| Philippines | incidence | 2006 | 2010 | 0.12 (-0.26 to 0.52) | 0.506 |
| Philippines | incidence | 2010 | 2014 | -0.75 (-1.12 to -0.39) | 0.001 |
| Philippines | incidence | 2014 | 2021 | 0.08 (-0.02 to 0.18) | 0.093 |
| Poland | incidence | 1990 | 1995 | -0.99 (-1.14 to -0.83) | <0.001 |
| Poland | incidence | 1995 | 2000 | -2.18 (-2.38 to -1.98) | <0.001 |
| Poland | incidence | 2000 | 2005 | -0.03 (-0.24 to 0.18) | 0.755 |
| Poland | incidence | 2005 | 2015 | -1.83 (-1.89 to -1.77) | <0.001 |
| Poland | incidence | 2015 | 2018 | 0.78 (0.15 to 1.41) | 0.019 |
| Poland | incidence | 2018 | 2021 | -0.43 (-0.76 to -0.11) | 0.012 |
| Portugal | incidence | 1990 | 1996 | -1.07 (-1.39 to -0.75) | <0.001 |
| Portugal | incidence | 1996 | 2000 | -5.34 (-6.16 to -4.51) | <0.001 |
| Portugal | incidence | 2000 | 2008 | -7.65 (-7.85 to -7.45) | <0.001 |
| Portugal | incidence | 2008 | 2013 | -4.37 (-4.91 to -3.84) | <0.001 |
| Portugal | incidence | 2013 | 2019 | -0.40 (-0.80 to 0.01) | 0.055 |
| Portugal | incidence | 2019 | 2021 | -2.86 (-4.79 to -0.89) | 0.008 |
| Puerto Rico | incidence | 1990 | 2000 | -0.92 (-0.95 to -0.88) | <0.001 |
| Puerto Rico | incidence | 2000 | 2005 | -1.67 (-1.82 to -1.52) | <0.001 |
| Puerto Rico | incidence | 2005 | 2009 | -2.35 (-2.60 to -2.10) | <0.001 |
| Puerto Rico | incidence | 2009 | 2014 | -2.01 (-2.16 to -1.86) | <0.001 |
| Puerto Rico | incidence | 2014 | 2018 | -0.25 (-0.49 to -0.01) | 0.044 |
| Puerto Rico | incidence | 2018 | 2021 | 0.64 (0.37 to 0.90) | <0.001 |
| Qatar | incidence | 1990 | 1994 | 2.60 (2.11 to 3.10) | <0.001 |
| Qatar | incidence | 1994 | 2000 | -0.71 (-1.01 to -0.40) | <0.001 |
| Qatar | incidence | 2000 | 2006 | -0.08 (-0.39 to 0.23) | 0.576 |
| Qatar | incidence | 2006 | 2011 | -2.40 (-2.81 to -1.99) | <0.001 |
| Qatar | incidence | 2011 | 2015 | -5.01 (-5.68 to -4.34) | <0.001 |
| Qatar | incidence | 2015 | 2021 | -2.94 (-3.20 to -2.68) | <0.001 |
| Republic of Korea | incidence | 1990 | 1996 | -0.33 (-0.70 to 0.04) | 0.078 |
| Republic of Korea | incidence | 1996 | 2003 | -4.08 (-4.37 to -3.79) | <0.001 |
| Republic of Korea | incidence | 2003 | 2010 | -6.11 (-6.38 to -5.83) | <0.001 |
| Republic of Korea | incidence | 2010 | 2014 | -3.40 (-4.29 to -2.50) | <0.001 |
| Republic of Korea | incidence | 2014 | 2018 | -1.17 (-2.12 to -0.22) | 0.02 |
| Republic of Korea | incidence | 2018 | 2021 | -3.08 (-4.17 to -1.98) | <0.001 |
| Republic of Moldova | incidence | 1990 | 1996 | -1.51 (-1.72 to -1.30) | <0.001 |
| Republic of Moldova | incidence | 1996 | 2010 | 0.34 (0.27 to 0.40) | <0.001 |
| Republic of Moldova | incidence | 2010 | 2014 | -1.78 (-2.39 to -1.16) | <0.001 |
| Republic of Moldova | incidence | 2014 | 2019 | -0.10 (-0.49 to 0.30) | 0.607 |
| Republic of Moldova | incidence | 2019 | 2021 | -2.24 (-3.53 to -0.94) | 0.002 |
| Romania | incidence | 1990 | 2001 | -0.58 (-0.62 to -0.54) | <0.001 |
| Romania | incidence | 2001 | 2010 | -1.60 (-1.65 to -1.54) | <0.001 |
| Romania | incidence | 2010 | 2014 | -2.59 (-2.85 to -2.34) | <0.001 |
| Romania | incidence | 2014 | 2019 | -0.16 (-0.33 to 0.01) | 0.061 |
| Romania | incidence | 2019 | 2021 | -1.33 (-1.87 to -0.79) | <0.001 |
| Russian Federation | incidence | 1990 | 1994 | -2.27 (-2.85 to -1.68) | <0.001 |
| Russian Federation | incidence | 1994 | 2003 | -0.05 (-0.24 to 0.14) | 0.582 |
| Russian Federation | incidence | 2003 | 2010 | -2.70 (-2.99 to -2.42) | <0.001 |
| Russian Federation | incidence | 2010 | 2014 | -0.50 (-1.33 to 0.33) | 0.217 |
| Russian Federation | incidence | 2014 | 2019 | 1.07 (0.53 to 1.60) | 0.001 |
| Russian Federation | incidence | 2019 | 2021 | -2.85 (-4.53 to -1.13) | 0.003 |
| Rwanda | incidence | 1990 | 1996 | -0.86 (-0.97 to -0.75) | <0.001 |
| Rwanda | incidence | 1996 | 2000 | -1.92 (-2.22 to -1.61) | <0.001 |
| Rwanda | incidence | 2000 | 2005 | -2.96 (-3.15 to -2.76) | <0.001 |
| Rwanda | incidence | 2005 | 2009 | -2.11 (-2.41 to -1.80) | <0.001 |
| Rwanda | incidence | 2009 | 2014 | -0.78 (-0.97 to -0.59) | <0.001 |
| Rwanda | incidence | 2014 | 2021 | 0.63 (0.54 to 0.71) | <0.001 |
| Saint Kitts and Nevis | incidence | 1990 | 1995 | -0.85 (-0.93 to -0.77) | <0.001 |
| Saint Kitts and Nevis | incidence | 1995 | 2000 | -2.46 (-2.57 to -2.35) | <0.001 |
| Saint Kitts and Nevis | incidence | 2000 | 2009 | -2.30 (-2.34 to -2.26) | <0.001 |
| Saint Kitts and Nevis | incidence | 2009 | 2014 | -1.09 (-1.21 to -0.97) | <0.001 |
| Saint Kitts and Nevis | incidence | 2014 | 2019 | 0.28 (0.16 to 0.40) | <0.001 |
| Saint Kitts and Nevis | incidence | 2019 | 2021 | -0.52 (-0.89 to -0.15) | 0.009 |
| Saint Lucia | incidence | 1990 | 1996 | -1.39 (-1.54 to -1.24) | <0.001 |
| Saint Lucia | incidence | 1996 | 2000 | -2.67 (-3.06 to -2.28) | <0.001 |
| Saint Lucia | incidence | 2000 | 2005 | -3.10 (-3.34 to -2.87) | <0.001 |
| Saint Lucia | incidence | 2005 | 2009 | -1.93 (-2.33 to -1.54) | <0.001 |
| Saint Lucia | incidence | 2009 | 2015 | -1.09 (-1.28 to -0.90) | <0.001 |
| Saint Lucia | incidence | 2015 | 2021 | 0.10 (-0.05 to 0.25) | 0.169 |
| Saint Vincent and the Grenadines | incidence | 1990 | 1995 | -1.32 (-1.45 to -1.18) | <0.001 |
| Saint Vincent and the Grenadines | incidence | 1995 | 2000 | -2.93 (-3.11 to -2.76) | <0.001 |
| Saint Vincent and the Grenadines | incidence | 2000 | 2004 | -1.51 (-1.79 to -1.22) | <0.001 |
| Saint Vincent and the Grenadines | incidence | 2004 | 2013 | -0.53 (-0.59 to -0.47) | <0.001 |
| Saint Vincent and the Grenadines | incidence | 2013 | 2021 | -0.18 (-0.24 to -0.11) | <0.001 |
| Samoa | incidence | 1990 | 2002 | 0.01 (-0.02 to 0.05) | 0.505 |
| Samoa | incidence | 2002 | 2008 | -0.35 (-0.48 to -0.23) | <0.001 |
| Samoa | incidence | 2008 | 2015 | -0.81 (-0.90 to -0.72) | <0.001 |
| Samoa | incidence | 2015 | 2018 | 0.09 (-0.44 to 0.62) | 0.728 |
| Samoa | incidence | 2018 | 2021 | -0.54 (-0.81 to -0.27) | 0.001 |
| San Marino | incidence | 1990 | 1996 | -1.31 (-1.38 to -1.25) | <0.001 |
| San Marino | incidence | 1996 | 2000 | -2.15 (-2.33 to -1.98) | <0.001 |
| San Marino | incidence | 2000 | 2007 | -2.63 (-2.69 to -2.57) | <0.001 |
| San Marino | incidence | 2007 | 2014 | -2.18 (-2.25 to -2.12) | <0.001 |
| San Marino | incidence | 2014 | 2021 | -0.11 (-0.16 to -0.06) | <0.001 |
| Sao Tome and Principe | incidence | 1990 | 1995 | 0.40 (0.29 to 0.51) | <0.001 |
| Sao Tome and Principe | incidence | 1995 | 2001 | 0.85 (0.75 to 0.96) | <0.001 |
| Sao Tome and Principe | incidence | 2001 | 2014 | -0.22 (-0.25 to -0.20) | <0.001 |
| Sao Tome and Principe | incidence | 2014 | 2019 | 0.24 (0.11 to 0.37) | 0.001 |
| Sao Tome and Principe | incidence | 2019 | 2021 | -1.14 (-1.58 to -0.70) | <0.001 |
| Saudi Arabia | incidence | 1990 | 2004 | 0.35 (0.30 to 0.40) | <0.001 |
| Saudi Arabia | incidence | 2004 | 2014 | -1.46 (-1.55 to -1.37) | <0.001 |
| Saudi Arabia | incidence | 2014 | 2019 | 0.11 (-0.21 to 0.42) | 0.478 |
| Saudi Arabia | incidence | 2019 | 2021 | -2.85 (-3.80 to -1.89) | <0.001 |
| Senegal | incidence | 1990 | 1994 | -1.25 (-1.44 to -1.07) | <0.001 |
| Senegal | incidence | 1994 | 2015 | -0.60 (-0.61 to -0.58) | <0.001 |
| Senegal | incidence | 2015 | 2019 | 0.29 (0.03 to 0.56) | 0.034 |
| Senegal | incidence | 2019 | 2021 | -0.62 (-1.17 to -0.06) | 0.032 |
| Serbia | incidence | 1990 | 1995 | -0.23 (-0.34 to -0.13) | <0.001 |
| Serbia | incidence | 1995 | 2000 | -2.49 (-2.62 to -2.36) | <0.001 |
| Serbia | incidence | 2000 | 2010 | -0.58 (-0.62 to -0.55) | <0.001 |
| Serbia | incidence | 2010 | 2015 | -1.30 (-1.41 to -1.19) | <0.001 |
| Serbia | incidence | 2015 | 2019 | 1.12 (0.94 to 1.30) | <0.001 |
| Serbia | incidence | 2019 | 2021 | -0.66 (-1.05 to -0.27) | 0.003 |
| Seychelles | incidence | 1990 | 1997 | -0.73 (-0.78 to -0.68) | <0.001 |
| Seychelles | incidence | 1997 | 2008 | -1.24 (-1.27 to -1.21) | <0.001 |
| Seychelles | incidence | 2008 | 2014 | -0.69 (-0.77 to -0.60) | <0.001 |
| Seychelles | incidence | 2014 | 2019 | 0.42 (0.30 to 0.54) | <0.001 |
| Seychelles | incidence | 2019 | 2021 | -0.52 (-0.91 to -0.13) | 0.013 |
| Sierra Leone | incidence | 1990 | 1994 | -0.68 (-0.76 to -0.59) | <0.001 |
| Sierra Leone | incidence | 1994 | 2006 | -0.25 (-0.27 to -0.24) | <0.001 |
| Sierra Leone | incidence | 2006 | 2015 | -0.58 (-0.61 to -0.55) | <0.001 |
| Sierra Leone | incidence | 2015 | 2019 | 0.34 (0.22 to 0.46) | <0.001 |
| Sierra Leone | incidence | 2019 | 2021 | -1.30 (-1.55 to -1.06) | <0.001 |
| Singapore | incidence | 1990 | 1996 | -1.37 (-1.52 to -1.22) | <0.001 |
| Singapore | incidence | 1996 | 2000 | -4.36 (-4.78 to -3.93) | <0.001 |
| Singapore | incidence | 2000 | 2009 | -5.90 (-5.99 to -5.80) | <0.001 |
| Singapore | incidence | 2009 | 2014 | -4.11 (-4.41 to -3.81) | <0.001 |
| Singapore | incidence | 2014 | 2019 | -0.15 (-0.48 to 0.19) | 0.367 |
| Singapore | incidence | 2019 | 2021 | -2.09 (-3.21 to -0.96) | 0.001 |
| Slovakia | incidence | 1990 | 1994 | 0.68 (0.48 to 0.89) | <0.001 |
| Slovakia | incidence | 1994 | 2000 | -0.46 (-0.57 to -0.35) | <0.001 |
| Slovakia | incidence | 2000 | 2006 | -0.21 (-0.30 to -0.12) | <0.001 |
| Slovakia | incidence | 2006 | 2010 | -1.70 (-1.90 to -1.50) | <0.001 |
| Slovakia | incidence | 2010 | 2019 | -3.40 (-3.45 to -3.35) | <0.001 |
| Slovakia | incidence | 2019 | 2021 | -1.44 (-2.10 to -0.77) | <0.001 |
| Slovenia | incidence | 1990 | 1995 | -4.66 (-4.80 to -4.52) | <0.001 |
| Slovenia | incidence | 1995 | 2000 | -5.30 (-5.46 to -5.14) | <0.001 |
| Slovenia | incidence | 2000 | 2009 | -2.80 (-2.85 to -2.76) | <0.001 |
| Slovenia | incidence | 2009 | 2015 | -1.56 (-1.65 to -1.47) | <0.001 |
| Slovenia | incidence | 2015 | 2019 | 0.79 (0.55 to 1.03) | <0.001 |
| Slovenia | incidence | 2019 | 2021 | -0.90 (-1.48 to -0.31) | 0.005 |
| Solomon Islands | incidence | 1990 | 1994 | 0.80 (0.73 to 0.87) | <0.001 |
| Solomon Islands | incidence | 1994 | 2000 | 0.07 (0.02 to 0.12) | 0.008 |
| Solomon Islands | incidence | 2000 | 2005 | -0.47 (-0.54 to -0.41) | <0.001 |
| Solomon Islands | incidence | 2005 | 2014 | 0.03 (0.01 to 0.06) | 0.004 |
| Solomon Islands | incidence | 2014 | 2019 | 0.40 (0.34 to 0.47) | <0.001 |
| Solomon Islands | incidence | 2019 | 2021 | -0.76 (-0.96 to -0.56) | <0.001 |
| Somalia | incidence | 1990 | 1994 | -0.95 (-1.03 to -0.87) | <0.001 |
| Somalia | incidence | 1994 | 2001 | -0.19 (-0.23 to -0.15) | <0.001 |
| Somalia | incidence | 2001 | 2004 | -0.72 (-0.96 to -0.47) | <0.001 |
| Somalia | incidence | 2004 | 2010 | -0.39 (-0.44 to -0.34) | <0.001 |
| Somalia | incidence | 2010 | 2015 | -0.49 (-0.55 to -0.42) | <0.001 |
| Somalia | incidence | 2015 | 2021 | 0.26 (0.22 to 0.29) | <0.001 |
| South Africa | incidence | 1990 | 2002 | 1.69 (1.53 to 1.85) | <0.001 |
| South Africa | incidence | 2002 | 2006 | 0.26 (-1.02 to 1.55) | 0.681 |
| South Africa | incidence | 2006 | 2014 | -2.41 (-2.72 to -2.09) | <0.001 |
| South Africa | incidence | 2014 | 2021 | -0.70 (-1.02 to -0.39) | <0.001 |
| South Sudan | incidence | 1990 | 1994 | -1.12 (-1.17 to -1.07) | <0.001 |
| South Sudan | incidence | 1994 | 2000 | -0.39 (-0.42 to -0.35) | <0.001 |
| South Sudan | incidence | 2000 | 2005 | -0.53 (-0.58 to -0.48) | <0.001 |
| South Sudan | incidence | 2005 | 2010 | 0.43 (0.39 to 0.48) | <0.001 |
| South Sudan | incidence | 2010 | 2014 | -0.41 (-0.49 to -0.34) | <0.001 |
| South Sudan | incidence | 2014 | 2021 | -0.26 (-0.28 to -0.24) | <0.001 |
| Spain | incidence | 1990 | 1995 | -1.67 (-2.17 to -1.18) | <0.001 |
| Spain | incidence | 1995 | 2000 | -5.54 (-6.15 to -4.92) | <0.001 |
| Spain | incidence | 2000 | 2003 | -2.61 (-4.19 to -1.01) | 0.004 |
| Spain | incidence | 2003 | 2010 | -1.71 (-1.89 to -1.53) | <0.001 |
| Spain | incidence | 2010 | 2014 | -2.82 (-3.29 to -2.35) | <0.001 |
| Spain | incidence | 2014 | 2021 | -0.39 (-0.54 to -0.24) | <0.001 |
| Sri Lanka | incidence | 1990 | 1995 | 0.38 (0.30 to 0.46) | <0.001 |
| Sri Lanka | incidence | 1995 | 2005 | -0.15 (-0.18 to -0.12) | <0.001 |
| Sri Lanka | incidence | 2005 | 2014 | -1.80 (-1.84 to -1.77) | <0.001 |
| Sri Lanka | incidence | 2014 | 2019 | 0.23 (0.13 to 0.32) | <0.001 |
| Sri Lanka | incidence | 2019 | 2021 | -2.55 (-2.88 to -2.23) | <0.001 |
| Sudan | incidence | 1990 | 1995 | 0.13 (0.07 to 0.20) | 0.001 |
| Sudan | incidence | 1995 | 2003 | 0.57 (0.53 to 0.61) | <0.001 |
| Sudan | incidence | 2003 | 2007 | 0.11 (-0.04 to 0.26) | 0.138 |
| Sudan | incidence | 2007 | 2015 | -0.42 (-0.46 to -0.38) | <0.001 |
| Sudan | incidence | 2015 | 2019 | 0.12 (-0.03 to 0.27) | 0.101 |
| Sudan | incidence | 2019 | 2021 | -0.80 (-1.09 to -0.51) | <0.001 |
| Suriname | incidence | 1990 | 1998 | 0.55 (0.51 to 0.59) | <0.001 |
| Suriname | incidence | 1998 | 2002 | 0.01 (-0.19 to 0.22) | 0.89 |
| Suriname | incidence | 2002 | 2006 | -0.46 (-0.66 to -0.26) | <0.001 |
| Suriname | incidence | 2006 | 2009 | -1.51 (-1.85 to -1.16) | <0.001 |
| Suriname | incidence | 2009 | 2014 | -1.02 (-1.13 to -0.90) | <0.001 |
| Suriname | incidence | 2014 | 2021 | -0.31 (-0.36 to -0.26) | <0.001 |
| Sweden | incidence | 1990 | 2000 | -1.35 (-1.42 to -1.29) | <0.001 |
| Sweden | incidence | 2000 | 2005 | -2.29 (-2.55 to -2.02) | <0.001 |
| Sweden | incidence | 2005 | 2010 | -3.91 (-4.17 to -3.66) | <0.001 |
| Sweden | incidence | 2010 | 2014 | -1.46 (-1.88 to -1.03) | <0.001 |
| Sweden | incidence | 2014 | 2021 | 0.58 (0.46 to 0.70) | <0.001 |
| Switzerland | incidence | 1990 | 1995 | -2.78 (-2.85 to -2.71) | <0.001 |
| Switzerland | incidence | 1995 | 2004 | -2.45 (-2.48 to -2.43) | <0.001 |
| Switzerland | incidence | 2004 | 2009 | -1.77 (-1.84 to -1.71) | <0.001 |
| Switzerland | incidence | 2009 | 2015 | -1.28 (-1.33 to -1.22) | <0.001 |
| Switzerland | incidence | 2015 | 2019 | -0.01 (-0.17 to 0.16) | 0.953 |
| Switzerland | incidence | 2019 | 2021 | -0.90 (-1.30 to -0.49) | <0.001 |
| Syrian Arab Republic | incidence | 1990 | 1993 | -0.31 (-0.44 to -0.18) | <0.001 |
| Syrian Arab Republic | incidence | 1993 | 1996 | -0.63 (-0.86 to -0.39) | <0.001 |
| Syrian Arab Republic | incidence | 1996 | 2009 | -1.40 (-1.42 to -1.39) | <0.001 |
| Syrian Arab Republic | incidence | 2009 | 2015 | 0.06 (0.01 to 0.12) | 0.027 |
| Syrian Arab Republic | incidence | 2015 | 2019 | -0.11 (-0.23 to 0.02) | 0.084 |
| Syrian Arab Republic | incidence | 2019 | 2021 | 0.88 (0.63 to 1.13) | <0.001 |
| Türkiye | incidence | 1990 | 1995 | -0.33 (-0.45 to -0.21) | <0.001 |
| Türkiye | incidence | 1995 | 2004 | -1.56 (-1.62 to -1.51) | <0.001 |
| Türkiye | incidence | 2004 | 2014 | -2.07 (-2.11 to -2.02) | <0.001 |
| Türkiye | incidence | 2014 | 2019 | 0.05 (-0.12 to 0.21) | 0.562 |
| Türkiye | incidence | 2019 | 2021 | -1.88 (-2.42 to -1.34) | <0.001 |
| Taiwan (Province of China) | incidence | 1990 | 1996 | 0.06 (-0.04 to 0.15) | 0.205 |
| Taiwan (Province of China) | incidence | 1996 | 2001 | -2.06 (-2.24 to -1.88) | <0.001 |
| Taiwan (Province of China) | incidence | 2001 | 2010 | -3.34 (-3.40 to -3.27) | <0.001 |
| Taiwan (Province of China) | incidence | 2010 | 2014 | -2.31 (-2.61 to -2.01) | <0.001 |
| Taiwan (Province of China) | incidence | 2014 | 2017 | -0.36 (-0.94 to 0.23) | 0.212 |
| Taiwan (Province of China) | incidence | 2017 | 2021 | 0.52 (0.33 to 0.71) | <0.001 |
| Tajikistan | incidence | 1990 | 1996 | -0.69 (-0.80 to -0.57) | <0.001 |
| Tajikistan | incidence | 1996 | 2004 | 0.69 (0.61 to 0.78) | <0.001 |
| Tajikistan | incidence | 2004 | 2010 | 2.22 (2.07 to 2.37) | <0.001 |
| Tajikistan | incidence | 2010 | 2015 | 0.29 (0.09 to 0.49) | 0.007 |
| Tajikistan | incidence | 2015 | 2019 | 0.97 (0.65 to 1.29) | <0.001 |
| Tajikistan | incidence | 2019 | 2021 | -0.55 (-1.19 to 0.10) | 0.093 |
| Thailand | incidence | 1990 | 1997 | -0.20 (-0.28 to -0.12) | <0.001 |
| Thailand | incidence | 1997 | 2004 | -1.37 (-1.46 to -1.27) | <0.001 |
| Thailand | incidence | 2004 | 2010 | -2.37 (-2.50 to -2.25) | <0.001 |
| Thailand | incidence | 2010 | 2014 | -1.62 (-1.89 to -1.36) | <0.001 |
| Thailand | incidence | 2014 | 2019 | 0.27 (0.10 to 0.44) | 0.004 |
| Thailand | incidence | 2019 | 2021 | -0.66 (-1.24 to -0.08) | 0.028 |
| Timor-Leste | incidence | 1990 | 1995 | -0.13 (-0.31 to 0.05) | 0.141 |
| Timor-Leste | incidence | 1995 | 2000 | 0.34 (0.10 to 0.58) | 0.008 |
| Timor-Leste | incidence | 2000 | 2006 | -0.02 (-0.18 to 0.15) | 0.823 |
| Timor-Leste | incidence | 2006 | 2015 | 1.02 (0.94 to 1.09) | <0.001 |
| Timor-Leste | incidence | 2015 | 2018 | 0.15 (-0.56 to 0.86) | 0.668 |
| Timor-Leste | incidence | 2018 | 2021 | 0.92 (0.54 to 1.30) | <0.001 |
| Togo | incidence | 1990 | 1994 | -0.54 (-0.68 to -0.39) | <0.001 |
| Togo | incidence | 1994 | 2000 | 0.13 (0.03 to 0.23) | 0.013 |
| Togo | incidence | 2000 | 2004 | -0.49 (-0.70 to -0.28) | <0.001 |
| Togo | incidence | 2004 | 2014 | -0.87 (-0.91 to -0.83) | <0.001 |
| Togo | incidence | 2014 | 2019 | 0.25 (0.11 to 0.39) | 0.002 |
| Togo | incidence | 2019 | 2021 | -1.04 (-1.47 to -0.60) | <0.001 |
| Tokelau | incidence | 1990 | 1999 | 0.07 (0.02 to 0.12) | 0.008 |
| Tokelau | incidence | 1999 | 2005 | -0.94 (-1.06 to -0.83) | <0.001 |
| Tokelau | incidence | 2005 | 2010 | -1.74 (-1.90 to -1.58) | <0.001 |
| Tokelau | incidence | 2010 | 2014 | -1.08 (-1.32 to -0.84) | <0.001 |
| Tokelau | incidence | 2014 | 2019 | 0.27 (0.13 to 0.42) | 0.001 |
| Tokelau | incidence | 2019 | 2021 | -0.49 (-0.99 to 0.02) | 0.058 |
| Tonga | incidence | 1990 | 1994 | 0.36 (0.26 to 0.46) | <0.001 |
| Tonga | incidence | 1994 | 2001 | -0.06 (-0.11 to -0.01) | 0.026 |
| Tonga | incidence | 2001 | 2005 | 0.32 (0.17 to 0.47) | <0.001 |
| Tonga | incidence | 2005 | 2014 | -0.62 (-0.65 to -0.58) | <0.001 |
| Tonga | incidence | 2014 | 2019 | 0.17 (0.08 to 0.27) | 0.002 |
| Tonga | incidence | 2019 | 2021 | -0.14 (-0.44 to 0.16) | 0.323 |
| Trinidad and Tobago | incidence | 1990 | 1996 | -0.72 (-0.77 to -0.67) | <0.001 |
| Trinidad and Tobago | incidence | 1996 | 2001 | -1.63 (-1.71 to -1.55) | <0.001 |
| Trinidad and Tobago | incidence | 2001 | 2010 | -2.45 (-2.48 to -2.42) | <0.001 |
| Trinidad and Tobago | incidence | 2010 | 2014 | -1.70 (-1.83 to -1.57) | <0.001 |
| Trinidad and Tobago | incidence | 2014 | 2019 | -0.18 (-0.27 to -0.10) | <0.001 |
| Trinidad and Tobago | incidence | 2019 | 2021 | -1.50 (-1.78 to -1.23) | <0.001 |
| Tunisia | incidence | 1990 | 1995 | 0.14 (0.02 to 0.26) | 0.028 |
| Tunisia | incidence | 1995 | 2000 | 1.05 (0.88 to 1.22) | <0.001 |
| Tunisia | incidence | 2000 | 2006 | 0.18 (0.05 to 0.30) | 0.008 |
| Tunisia | incidence | 2006 | 2015 | -0.69 (-0.75 to -0.63) | <0.001 |
| Tunisia | incidence | 2015 | 2019 | 0.29 (0.04 to 0.54) | 0.027 |
| Tunisia | incidence | 2019 | 2021 | -1.01 (-1.56 to -0.46) | 0.001 |
| Turkmenistan | incidence | 1990 | 1994 | -1.71 (-2.28 to -1.13) | <0.001 |
| Turkmenistan | incidence | 1994 | 2006 | 1.07 (0.95 to 1.20) | <0.001 |
| Turkmenistan | incidence | 2006 | 2010 | 1.90 (0.97 to 2.84) | 0.001 |
| Turkmenistan | incidence | 2010 | 2015 | -0.68 (-1.24 to -0.12) | 0.021 |
| Turkmenistan | incidence | 2015 | 2019 | 1.03 (0.14 to 1.92) | 0.026 |
| Turkmenistan | incidence | 2019 | 2021 | -0.71 (-2.44 to 1.06) | 0.404 |
| Tuvalu | incidence | 1990 | 1994 | 0.85 (0.77 to 0.94) | <0.001 |
| Tuvalu | incidence | 1994 | 2000 | 0.27 (0.21 to 0.33) | <0.001 |
| Tuvalu | incidence | 2000 | 2005 | -0.37 (-0.45 to -0.29) | <0.001 |
| Tuvalu | incidence | 2005 | 2014 | -0.69 (-0.71 to -0.66) | <0.001 |
| Tuvalu | incidence | 2014 | 2019 | 0.04 (-0.03 to 0.12) | 0.246 |
| Tuvalu | incidence | 2019 | 2021 | -0.36 (-0.60 to -0.11) | 0.008 |
| Uganda | incidence | 1990 | 1995 | 0.46 (0.32 to 0.60) | <0.001 |
| Uganda | incidence | 1995 | 2000 | 0.95 (0.76 to 1.13) | <0.001 |
| Uganda | incidence | 2000 | 2003 | -0.28 (-0.85 to 0.29) | 0.309 |
| Uganda | incidence | 2003 | 2006 | -0.62 (-1.20 to -0.04) | 0.038 |
| Uganda | incidence | 2006 | 2014 | -1.31 (-1.39 to -1.24) | <0.001 |
| Uganda | incidence | 2014 | 2021 | -0.05 (-0.12 to 0.03) | 0.226 |
| Ukraine | incidence | 1990 | 1998 | -0.41 (-0.43 to -0.38) | <0.001 |
| Ukraine | incidence | 1998 | 2001 | -0.77 (-1.00 to -0.54) | <0.001 |
| Ukraine | incidence | 2001 | 2006 | -2.13 (-2.20 to -2.06) | <0.001 |
| Ukraine | incidence | 2006 | 2009 | -2.66 (-2.88 to -2.44) | <0.001 |
| Ukraine | incidence | 2009 | 2014 | -2.20 (-2.26 to -2.13) | <0.001 |
| Ukraine | incidence | 2014 | 2021 | -0.32 (-0.35 to -0.29) | <0.001 |
| United Arab Emirates | incidence | 1990 | 2002 | 0.11 (0.05 to 0.18) | 0.002 |
| United Arab Emirates | incidence | 2002 | 2005 | -1.01 (-2.11 to 0.11) | 0.074 |
| United Arab Emirates | incidence | 2005 | 2010 | -2.79 (-3.13 to -2.45) | <0.001 |
| United Arab Emirates | incidence | 2010 | 2014 | -1.89 (-2.39 to -1.39) | <0.001 |
| United Arab Emirates | incidence | 2014 | 2019 | 0.00 (-0.32 to 0.32) | 0.998 |
| United Arab Emirates | incidence | 2019 | 2021 | -1.86 (-2.93 to -0.77) | 0.002 |
| United Kingdom | incidence | 1990 | 1997 | -1.71 (-1.79 to -1.63) | <0.001 |
| United Kingdom | incidence | 1997 | 2004 | -2.46 (-2.55 to -2.37) | <0.001 |
| United Kingdom | incidence | 2004 | 2010 | -3.42 (-3.54 to -3.30) | <0.001 |
| United Kingdom | incidence | 2010 | 2014 | -2.09 (-2.34 to -1.84) | <0.001 |
| United Kingdom | incidence | 2014 | 2019 | -1.25 (-1.40 to -1.09) | <0.001 |
| United Kingdom | incidence | 2019 | 2021 | 0.79 (0.26 to 1.32) | 0.006 |
| United Republic of Tanzania | incidence | 1990 | 1993 | -1.81 (-2.09 to -1.54) | <0.001 |
| United Republic of Tanzania | incidence | 1993 | 1996 | -0.38 (-0.89 to 0.13) | 0.132 |
| United Republic of Tanzania | incidence | 1996 | 2005 | 0.70 (0.65 to 0.76) | <0.001 |
| United Republic of Tanzania | incidence | 2005 | 2014 | 1.44 (1.39 to 1.49) | <0.001 |
| United Republic of Tanzania | incidence | 2014 | 2017 | 0.71 (0.20 to 1.23) | 0.01 |
| United Republic of Tanzania | incidence | 2017 | 2021 | 0.20 (0.03 to 0.36) | 0.026 |
| United States of America | incidence | 1990 | 1994 | -2.71 (-2.86 to -2.56) | <0.001 |
| United States of America | incidence | 1994 | 2001 | -1.12 (-1.20 to -1.05) | <0.001 |
| United States of America | incidence | 2001 | 2009 | -3.03 (-3.08 to -2.97) | <0.001 |
| United States of America | incidence | 2009 | 2012 | -0.65 (-1.06 to -0.25) | 0.003 |
| United States of America | incidence | 2012 | 2021 | 0.00 (-0.03 to 0.04) | 0.841 |
| United States Virgin Islands | incidence | 1990 | 1995 | -0.11 (-0.15 to -0.07) | <0.001 |
| United States Virgin Islands | incidence | 1995 | 2000 | -0.88 (-0.93 to -0.82) | <0.001 |
| United States Virgin Islands | incidence | 2000 | 2010 | 0.34 (0.33 to 0.36) | <0.001 |
| United States Virgin Islands | incidence | 2010 | 2019 | -0.15 (-0.17 to -0.13) | <0.001 |
| United States Virgin Islands | incidence | 2019 | 2021 | 0.41 (0.24 to 0.58) | <0.001 |
| Uruguay | incidence | 1990 | 1996 | -1.05 (-1.24 to -0.86) | <0.001 |
| Uruguay | incidence | 1996 | 2010 | -3.08 (-3.13 to -3.03) | <0.001 |
| Uruguay | incidence | 2010 | 2014 | -2.24 (-2.77 to -1.70) | <0.001 |
| Uruguay | incidence | 2014 | 2019 | 0.36 (0.03 to 0.69) | 0.036 |
| Uruguay | incidence | 2019 | 2021 | -3.41 (-4.49 to -2.33) | <0.001 |
| Uzbekistan | incidence | 1990 | 1995 | 1.02 (0.85 to 1.20) | <0.001 |
| Uzbekistan | incidence | 1995 | 2000 | 2.98 (2.75 to 3.22) | <0.001 |
| Uzbekistan | incidence | 2000 | 2009 | 0.00 (-0.07 to 0.08) | 0.911 |
| Uzbekistan | incidence | 2009 | 2015 | -1.36 (-1.52 to -1.20) | <0.001 |
| Uzbekistan | incidence | 2015 | 2019 | 0.74 (0.38 to 1.11) | 0.001 |
| Uzbekistan | incidence | 2019 | 2021 | -1.87 (-2.62 to -1.12) | <0.001 |
| Vanuatu | incidence | 1990 | 1994 | 0.56 (0.37 to 0.76) | <0.001 |
| Vanuatu | incidence | 1994 | 2000 | 0.20 (0.06 to 0.33) | 0.006 |
| Vanuatu | incidence | 2000 | 2004 | -0.40 (-0.68 to -0.11) | 0.01 |
| Vanuatu | incidence | 2004 | 2015 | -0.12 (-0.16 to -0.07) | <0.001 |
| Vanuatu | incidence | 2015 | 2018 | 0.21 (-0.32 to 0.75) | 0.414 |
| Vanuatu | incidence | 2018 | 2021 | -0.32 (-0.59 to -0.06) | 0.021 |
| Venezuela (Bolivarian Republic of) | incidence | 1990 | 2001 | -1.03 (-1.07 to -0.99) | <0.001 |
| Venezuela (Bolivarian Republic of) | incidence | 2001 | 2004 | -1.87 (-2.38 to -1.36) | <0.001 |
| Venezuela (Bolivarian Republic of) | incidence | 2004 | 2009 | -1.15 (-1.32 to -0.98) | <0.001 |
| Venezuela (Bolivarian Republic of) | incidence | 2009 | 2016 | -0.05 (-0.15 to 0.05) | 0.291 |
| Venezuela (Bolivarian Republic of) | incidence | 2016 | 2021 | -0.58 (-0.71 to -0.46) | <0.001 |
| Viet Nam | incidence | 1990 | 1994 | 0.47 (-0.21 to 1.16) | 0.165 |
| Viet Nam | incidence | 1994 | 2003 | -0.72 (-0.93 to -0.51) | <0.001 |
| Viet Nam | incidence | 2003 | 2010 | 1.05 (0.79 to 1.30) | <0.001 |
| Viet Nam | incidence | 2010 | 2016 | -0.36 (-0.67 to -0.06) | 0.023 |
| Viet Nam | incidence | 2016 | 2021 | 0.92 (0.60 to 1.25) | <0.001 |
| Yemen | incidence | 1990 | 1995 | -0.04 (-0.08 to -0.01) | 0.028 |
| Yemen | incidence | 1995 | 2000 | 0.56 (0.51 to 0.61) | <0.001 |
| Yemen | incidence | 2000 | 2008 | 0.19 (0.17 to 0.22) | <0.001 |
| Yemen | incidence | 2008 | 2011 | -0.01 (-0.16 to 0.14) | 0.85 |
| Yemen | incidence | 2011 | 2017 | -0.32 (-0.36 to -0.29) | <0.001 |
| Yemen | incidence | 2017 | 2021 | -0.55 (-0.59 to -0.50) | <0.001 |
| Zambia | incidence | 1990 | 1995 | -0.26 (-0.33 to -0.20) | <0.001 |
| Zambia | incidence | 1995 | 2000 | 0.67 (0.59 to 0.76) | <0.001 |
| Zambia | incidence | 2000 | 2005 | -0.35 (-0.44 to -0.26) | <0.001 |
| Zambia | incidence | 2005 | 2010 | 1.39 (1.30 to 1.48) | <0.001 |
| Zambia | incidence | 2010 | 2014 | 0.85 (0.71 to 0.98) | <0.001 |
| Zambia | incidence | 2014 | 2021 | 0.30 (0.26 to 0.34) | <0.001 |
| Zimbabwe | incidence | 1990 | 1995 | 0.14 (-0.23 to 0.50) | 0.454 |
| Zimbabwe | incidence | 1995 | 2000 | 1.15 (0.66 to 1.63) | <0.001 |
| Zimbabwe | incidence | 2000 | 2006 | 1.93 (1.56 to 2.31) | <0.001 |
| Zimbabwe | incidence | 2006 | 2021 | -0.08 (-0.15 to -0.01) | 0.025 |
| Afghanistan | death | 1990 | 1992 | 0.35 (-0.15 to 0.84) | 0.157 |
| Afghanistan | death | 1992 | 1996 | 1.08 (0.81 to 1.35) | <0.001 |
| Afghanistan | death | 1996 | 2005 | 0.45 (0.39 to 0.51) | <0.001 |
| Afghanistan | death | 2005 | 2013 | -1.56 (-1.62 to -1.49) | <0.001 |
| Afghanistan | death | 2013 | 2019 | -0.32 (-0.42 to -0.21) | <0.001 |
| Afghanistan | death | 2019 | 2021 | -1.45 (-1.89 to -1.01) | <0.001 |
| Albania | death | 1990 | 1994 | -5.91 (-7.37 to -4.42) | <0.001 |
| Albania | death | 1994 | 2001 | -0.48 (-1.25 to 0.29) | 0.201 |
| Albania | death | 2001 | 2004 | 2.84 (-2.09 to 8.02) | 0.243 |
| Albania | death | 2004 | 2008 | -3.89 (-6.37 to -1.34) | 0.006 |
| Albania | death | 2008 | 2017 | 2.23 (1.57 to 2.88) | <0.001 |
| Albania | death | 2017 | 2021 | -1.61 (-3.66 to 0.49) | 0.123 |
| Algeria | death | 1990 | 1992 | -4.18 (-6.03 to -2.30) | <0.001 |
| Algeria | death | 1992 | 1998 | -1.09 (-1.51 to -0.67) | <0.001 |
| Algeria | death | 1998 | 2010 | -0.49 (-0.61 to -0.36) | <0.001 |
| Algeria | death | 2010 | 2021 | -1.18 (-1.31 to -1.05) | <0.001 |
| American Samoa | death | 1990 | 1999 | 0.30 (0.07 to 0.52) | 0.011 |
| American Samoa | death | 1999 | 2004 | -3.90 (-4.59 to -3.20) | <0.001 |
| American Samoa | death | 2004 | 2021 | -0.58 (-0.68 to -0.48) | <0.001 |
| Andorra | death | 1990 | 1999 | -1.85 (-2.43 to -1.27) | <0.001 |
| Andorra | death | 1999 | 2005 | -4.51 (-5.85 to -3.14) | <0.001 |
| Andorra | death | 2005 | 2018 | -0.48 (-0.81 to -0.14) | 0.008 |
| Andorra | death | 2018 | 2021 | -8.52 (-11.45 to -5.49) | <0.001 |
| Angola | death | 1990 | 2000 | 0.04 (-0.11 to 0.18) | 0.603 |
| Angola | death | 2000 | 2015 | -0.78 (-0.86 to -0.70) | <0.001 |
| Angola | death | 2015 | 2021 | 1.47 (1.16 to 1.78) | <0.001 |
| Antigua and Barbuda | death | 1990 | 1994 | 4.89 (2.73 to 7.10) | <0.001 |
| Antigua and Barbuda | death | 1994 | 2001 | -4.56 (-5.58 to -3.52) | <0.001 |
| Antigua and Barbuda | death | 2001 | 2010 | -1.34 (-2.05 to -0.62) | 0.001 |
| Antigua and Barbuda | death | 2010 | 2014 | 1.62 (-1.88 to 5.26) | 0.348 |
| Antigua and Barbuda | death | 2014 | 2021 | -2.98 (-3.96 to -2.00) | <0.001 |
| Argentina | death | 1990 | 1996 | -5.46 (-6.24 to -4.68) | <0.001 |
| Argentina | death | 1996 | 2003 | -2.48 (-3.28 to -1.68) | <0.001 |
| Argentina | death | 2003 | 2006 | -5.72 (-10.27 to -0.95) | 0.023 |
| Argentina | death | 2006 | 2012 | -2.51 (-3.60 to -1.40) | <0.001 |
| Argentina | death | 2012 | 2016 | 0.87 (-1.71 to 3.53) | 0.486 |
| Argentina | death | 2016 | 2021 | -4.65 (-5.81 to -3.48) | <0.001 |
| Armenia | death | 1990 | 1993 | 8.96 (3.31 to 14.93) | 0.003 |
| Armenia | death | 1993 | 1999 | -4.03 (-6.01 to -2.01) | 0.001 |
| Armenia | death | 1999 | 2002 | 4.30 (-5.15 to 14.68) | 0.364 |
| Armenia | death | 2002 | 2014 | -4.76 (-5.36 to -4.16) | <0.001 |
| Armenia | death | 2014 | 2021 | 1.97 (0.12 to 3.86) | 0.038 |
| Australia | death | 1990 | 1995 | -2.02 (-3.17 to -0.85) | 0.002 |
| Australia | death | 1995 | 2004 | -5.14 (-5.75 to -4.52) | <0.001 |
| Australia | death | 2004 | 2021 | -3.91 (-4.15 to -3.67) | <0.001 |
| Austria | death | 1990 | 1994 | -5.80 (-7.07 to -4.51) | <0.001 |
| Austria | death | 1994 | 1997 | -2.89 (-7.23 to 1.66) | 0.193 |
| Austria | death | 1997 | 2002 | -7.22 (-8.66 to -5.76) | <0.001 |
| Austria | death | 2002 | 2005 | -11.36 (-16.01 to -6.45) | <0.001 |
| Austria | death | 2005 | 2015 | -4.55 (-5.07 to -4.03) | <0.001 |
| Austria | death | 2015 | 2021 | -1.27 (-2.34 to -0.18) | 0.026 |
| Azerbaijan | death | 1990 | 1994 | 2.11 (0.84 to 3.40) | 0.003 |
| Azerbaijan | death | 1994 | 1998 | -2.65 (-4.54 to -0.73) | 0.01 |
| Azerbaijan | death | 1998 | 2001 | -0.36 (-4.23 to 3.66) | 0.847 |
| Azerbaijan | death | 2001 | 2004 | 6.44 (2.00 to 11.08) | 0.007 |
| Azerbaijan | death | 2004 | 2017 | -0.69 (-0.94 to -0.44) | <0.001 |
| Azerbaijan | death | 2017 | 2021 | -4.87 (-6.19 to -3.54) | <0.001 |
| Bahamas | death | 1990 | 1995 | 0.35 (-0.97 to 1.68) | 0.583 |
| Bahamas | death | 1995 | 2001 | -3.11 (-4.19 to -2.02) | <0.001 |
| Bahamas | death | 2001 | 2004 | 5.57 (0.87 to 10.50) | 0.023 |
| Bahamas | death | 2004 | 2007 | -8.05 (-12.31 to -3.59) | 0.002 |
| Bahamas | death | 2007 | 2010 | 2.55 (-2.21 to 7.54) | 0.276 |
| Bahamas | death | 2010 | 2021 | -1.44 (-1.88 to -0.99) | <0.001 |
| Bahrain | death | 1990 | 2005 | 0.25 (-0.02 to 0.52) | 0.063 |
| Bahrain | death | 2005 | 2012 | -3.12 (-4.12 to -2.11) | <0.001 |
| Bahrain | death | 2012 | 2015 | -11.99 (-18.09 to -5.43) | 0.001 |
| Bahrain | death | 2015 | 2021 | 0.36 (-1.08 to 1.82) | 0.614 |
| Bangladesh | death | 1990 | 1997 | 0.71 (-0.13 to 1.56) | 0.09 |
| Bangladesh | death | 1997 | 2002 | 3.39 (1.51 to 5.30) | 0.001 |
| Bangladesh | death | 2002 | 2010 | -0.99 (-1.73 to -0.25) | 0.012 |
| Bangladesh | death | 2010 | 2013 | -9.27 (-14.78 to -3.41) | 0.005 |
| Bangladesh | death | 2013 | 2017 | 3.65 (0.34 to 7.07) | 0.033 |
| Bangladesh | death | 2017 | 2021 | -0.26 (-2.26 to 1.79) | 0.792 |
| Barbados | death | 1990 | 1993 | 1.13 (-1.49 to 3.82) | 0.377 |
| Barbados | death | 1993 | 1996 | -2.22 (-6.46 to 2.21) | 0.298 |
| Barbados | death | 1996 | 1999 | -6.97 (-11.29 to -2.45) | 0.005 |
| Barbados | death | 1999 | 2003 | 0.88 (-1.48 to 3.29) | 0.443 |
| Barbados | death | 2003 | 2006 | -5.49 (-9.94 to -0.81) | 0.025 |
| Barbados | death | 2006 | 2021 | -0.63 (-0.96 to -0.30) | 0.001 |
| Belarus | death | 1990 | 1993 | 5.08 (1.14 to 9.18) | 0.014 |
| Belarus | death | 1993 | 2005 | 0.04 (-0.43 to 0.52) | 0.851 |
| Belarus | death | 2005 | 2015 | -4.38 (-4.98 to -3.77) | <0.001 |
| Belarus | death | 2015 | 2021 | -0.75 (-2.45 to 0.98) | 0.375 |
| Belgium | death | 1990 | 2010 | -4.57 (-4.70 to -4.43) | <0.001 |
| Belgium | death | 2010 | 2021 | -3.20 (-3.62 to -2.77) | <0.001 |
| Belize | death | 1990 | 1995 | 1.39 (-0.49 to 3.30) | 0.138 |
| Belize | death | 1995 | 1999 | 11.69 (7.81 to 15.72) | <0.001 |
| Belize | death | 1999 | 2002 | -6.64 (-12.25 to -0.67) | 0.032 |
| Belize | death | 2002 | 2013 | -2.80 (-3.31 to -2.28) | <0.001 |
| Belize | death | 2013 | 2021 | -0.63 (-1.56 to 0.31) | 0.178 |
| Benin | death | 1990 | 2000 | -0.40 (-0.48 to -0.31) | <0.001 |
| Benin | death | 2000 | 2004 | -1.56 (-2.11 to -1.01) | <0.001 |
| Benin | death | 2004 | 2007 | -0.59 (-1.65 to 0.49) | 0.26 |
| Benin | death | 2007 | 2012 | 0.80 (0.43 to 1.16) | <0.001 |
| Benin | death | 2012 | 2015 | 0.17 (-0.93 to 1.28) | 0.75 |
| Benin | death | 2015 | 2021 | -0.80 (-0.99 to -0.62) | <0.001 |
| Bermuda | death | 1990 | 1995 | -3.32 (-4.30 to -2.33) | <0.001 |
| Bermuda | death | 1995 | 1999 | -6.09 (-8.01 to -4.13) | <0.001 |
| Bermuda | death | 1999 | 2002 | 0.11 (-4.71 to 5.18) | 0.962 |
| Bermuda | death | 2002 | 2012 | -3.91 (-4.50 to -3.32) | <0.001 |
| Bermuda | death | 2012 | 2021 | -1.42 (-2.11 to -0.73) | <0.001 |
| Bhutan | death | 1990 | 1995 | -0.49 (-0.77 to -0.20) | 0.002 |
| Bhutan | death | 1995 | 2003 | -1.19 (-1.35 to -1.04) | <0.001 |
| Bhutan | death | 2003 | 2007 | -0.61 (-1.13 to -0.08) | 0.027 |
| Bhutan | death | 2007 | 2014 | -0.13 (-0.27 to 0.01) | 0.069 |
| Bhutan | death | 2014 | 2018 | 0.13 (-0.24 to 0.49) | 0.478 |
| Bhutan | death | 2018 | 2021 | -0.44 (-0.80 to -0.07) | 0.024 |
| Bolivia (Plurinational State of) | death | 1990 | 1993 | -1.48 (-1.91 to -1.04) | <0.001 |
| Bolivia (Plurinational State of) | death | 1993 | 1999 | -2.10 (-2.29 to -1.91) | <0.001 |
| Bolivia (Plurinational State of) | death | 1999 | 2003 | -2.81 (-3.24 to -2.37) | <0.001 |
| Bolivia (Plurinational State of) | death | 2003 | 2006 | -1.79 (-2.62 to -0.95) | <0.001 |
| Bolivia (Plurinational State of) | death | 2006 | 2016 | -0.61 (-0.70 to -0.52) | <0.001 |
| Bolivia (Plurinational State of) | death | 2016 | 2021 | -1.65 (-1.88 to -1.42) | <0.001 |
| Bosnia and Herzegovina | death | 1990 | 1994 | 2.44 (0.77 to 4.14) | 0.006 |
| Bosnia and Herzegovina | death | 1994 | 2004 | -3.06 (-3.54 to -2.57) | <0.001 |
| Bosnia and Herzegovina | death | 2004 | 2021 | -0.51 (-0.67 to -0.35) | <0.001 |
| Botswana | death | 1990 | 2003 | -0.68 (-1.11 to -0.24) | 0.005 |
| Botswana | death | 2003 | 2006 | -5.30 (-13.25 to 3.37) | 0.205 |
| Botswana | death | 2006 | 2010 | 4.22 (0.47 to 8.11) | 0.03 |
| Botswana | death | 2010 | 2014 | -1.69 (-4.52 to 1.24) | 0.235 |
| Botswana | death | 2014 | 2018 | -5.31 (-8.09 to -2.45) | 0.001 |
| Botswana | death | 2018 | 2021 | -1.03 (-4.38 to 2.43) | 0.529 |
| Brazil | death | 1990 | 1993 | -2.75 (-4.01 to -1.48) | <0.001 |
| Brazil | death | 1993 | 1999 | -4.84 (-5.43 to -4.24) | <0.001 |
| Brazil | death | 1999 | 2010 | -2.44 (-2.68 to -2.20) | <0.001 |
| Brazil | death | 2010 | 2021 | -3.09 (-3.31 to -2.87) | <0.001 |
| Brunei Darussalam | death | 1990 | 2010 | -2.75 (-2.95 to -2.55) | <0.001 |
| Brunei Darussalam | death | 2010 | 2016 | 3.35 (1.64 to 5.08) | <0.001 |
| Brunei Darussalam | death | 2016 | 2021 | -7.13 (-8.81 to -5.42) | <0.001 |
| Bulgaria | death | 1990 | 1992 | -5.25 (-10.58 to 0.40) | 0.066 |
| Bulgaria | death | 1992 | 1995 | 3.34 (-2.58 to 9.62) | 0.258 |
| Bulgaria | death | 1995 | 1999 | -2.63 (-5.53 to 0.36) | 0.081 |
| Bulgaria | death | 1999 | 2006 | 1.51 (0.37 to 2.67) | 0.012 |
| Bulgaria | death | 2006 | 2021 | -1.31 (-1.70 to -0.93) | <0.001 |
| Burkina Faso | death | 1990 | 1998 | -0.30 (-0.50 to -0.10) | 0.006 |
| Burkina Faso | death | 1998 | 2002 | 2.19 (1.27 to 3.12) | <0.001 |
| Burkina Faso | death | 2002 | 2013 | 0.36 (0.22 to 0.51) | <0.001 |
| Burkina Faso | death | 2013 | 2021 | -1.07 (-1.26 to -0.87) | <0.001 |
| Burundi | death | 1990 | 1996 | 0.03 (-0.19 to 0.26) | 0.764 |
| Burundi | death | 1996 | 1999 | -2.77 (-4.06 to -1.47) | <0.001 |
| Burundi | death | 1999 | 2002 | -4.42 (-5.66 to -3.16) | <0.001 |
| Burundi | death | 2002 | 2008 | -3.44 (-3.73 to -3.16) | <0.001 |
| Burundi | death | 2008 | 2014 | -0.98 (-1.27 to -0.69) | <0.001 |
| Burundi | death | 2014 | 2021 | 0.08 (-0.10 to 0.25) | 0.376 |
| Cabo Verde | death | 1990 | 2005 | 0.10 (-0.04 to 0.24) | 0.137 |
| Cabo Verde | death | 2005 | 2008 | -3.14 (-5.68 to -0.52) | 0.022 |
| Cabo Verde | death | 2008 | 2015 | 0.93 (0.46 to 1.39) | 0.001 |
| Cabo Verde | death | 2015 | 2018 | 10.55 (7.45 to 13.75) | <0.001 |
| Cabo Verde | death | 2018 | 2021 | 0.40 (-1.11 to 1.93) | 0.588 |
| Cambodia | death | 1990 | 1996 | 0.01 (-0.09 to 0.11) | 0.895 |
| Cambodia | death | 1996 | 2000 | -0.62 (-0.90 to -0.34) | <0.001 |
| Cambodia | death | 2000 | 2006 | -1.17 (-1.31 to -1.04) | <0.001 |
| Cambodia | death | 2006 | 2011 | 0.43 (0.24 to 0.62) | <0.001 |
| Cambodia | death | 2011 | 2015 | 0.87 (0.55 to 1.19) | <0.001 |
| Cambodia | death | 2015 | 2021 | 0.42 (0.31 to 0.52) | <0.001 |
| Cameroon | death | 1990 | 1994 | 1.26 (0.96 to 1.57) | <0.001 |
| Cameroon | death | 1994 | 1999 | 3.68 (3.36 to 4.01) | <0.001 |
| Cameroon | death | 1999 | 2002 | 3.00 (2.03 to 3.98) | <0.001 |
| Cameroon | death | 2002 | 2006 | 0.24 (-0.17 to 0.66) | 0.232 |
| Cameroon | death | 2006 | 2012 | -1.02 (-1.22 to -0.83) | <0.001 |
| Cameroon | death | 2012 | 2021 | -1.84 (-1.94 to -1.75) | <0.001 |
| Canada | death | 1990 | 1996 | -0.93 (-1.43 to -0.43) | 0.001 |
| Canada | death | 1996 | 2002 | -3.67 (-4.35 to -2.99) | <0.001 |
| Canada | death | 2002 | 2005 | -6.59 (-9.75 to -3.31) | 0.001 |
| Canada | death | 2005 | 2013 | -4.27 (-4.75 to -3.78) | <0.001 |
| Canada | death | 2013 | 2021 | -1.92 (-2.36 to -1.47) | <0.001 |
| Central African Republic | death | 1990 | 1992 | -0.32 (-1.15 to 0.53) | 0.438 |
| Central African Republic | death | 1992 | 1996 | 0.76 (0.32 to 1.20) | 0.002 |
| Central African Republic | death | 1996 | 2003 | -0.21 (-0.36 to -0.07) | 0.007 |
| Central African Republic | death | 2003 | 2009 | -0.78 (-0.98 to -0.58) | <0.001 |
| Central African Republic | death | 2009 | 2021 | -0.43 (-0.49 to -0.38) | <0.001 |
| Chad | death | 1990 | 1992 | 0.39 (-0.76 to 1.55) | 0.482 |
| Chad | death | 1992 | 1999 | 1.91 (1.72 to 2.10) | <0.001 |
| Chad | death | 1999 | 2003 | 1.11 (0.54 to 1.68) | 0.001 |
| Chad | death | 2003 | 2011 | -0.08 (-0.22 to 0.06) | 0.242 |
| Chad | death | 2011 | 2014 | -0.87 (-1.81 to 0.08) | 0.07 |
| Chad | death | 2014 | 2021 | -0.14 (-0.28 to -0.01) | 0.034 |
| Chile | death | 1990 | 1992 | -3.27 (-6.31 to -0.12) | 0.043 |
| Chile | death | 1992 | 1995 | -8.57 (-11.65 to -5.39) | <0.001 |
| Chile | death | 1995 | 1999 | -0.38 (-2.22 to 1.50) | 0.669 |
| Chile | death | 1999 | 2008 | -2.37 (-2.80 to -1.93) | <0.001 |
| Chile | death | 2008 | 2011 | 1.40 (-2.76 to 5.74) | 0.489 |
| Chile | death | 2011 | 2021 | -4.27 (-4.62 to -3.91) | <0.001 |
| China | death | 1990 | 1998 | -0.03 (-0.32 to 0.26) | 0.805 |
| China | death | 1998 | 2004 | 2.91 (2.49 to 3.33) | <0.001 |
| China | death | 2004 | 2007 | -4.91 (-6.29 to -3.51) | <0.001 |
| China | death | 2007 | 2010 | 0.51 (-1.23 to 2.28) | 0.544 |
| China | death | 2010 | 2014 | -2.58 (-3.47 to -1.68) | <0.001 |
| China | death | 2014 | 2021 | -1.14 (-1.50 to -0.78) | <0.001 |
| Colombia | death | 1990 | 1995 | -2.17 (-3.10 to -1.23) | <0.001 |
| Colombia | death | 1995 | 1998 | -8.24 (-12.41 to -3.87) | 0.001 |
| Colombia | death | 1998 | 2003 | -2.80 (-4.29 to -1.28) | 0.001 |
| Colombia | death | 2003 | 2014 | -4.16 (-4.57 to -3.75) | <0.001 |
| Colombia | death | 2014 | 2021 | -0.42 (-1.35 to 0.53) | 0.367 |
| Comoros | death | 1990 | 1997 | -0.84 (-1.00 to -0.69) | <0.001 |
| Comoros | death | 1997 | 2005 | -2.89 (-3.02 to -2.75) | <0.001 |
| Comoros | death | 2005 | 2013 | -0.75 (-0.89 to -0.62) | <0.001 |
| Comoros | death | 2013 | 2021 | 0.37 (0.24 to 0.50) | <0.001 |
| Congo | death | 1990 | 1992 | -0.09 (-1.17 to 0.99) | 0.854 |
| Congo | death | 1992 | 1996 | 1.52 (0.97 to 2.08) | <0.001 |
| Congo | death | 1996 | 1999 | -0.84 (-1.91 to 0.24) | 0.118 |
| Congo | death | 1999 | 2002 | -2.07 (-3.10 to -1.04) | 0.001 |
| Congo | death | 2002 | 2011 | -1.18 (-1.29 to -1.07) | <0.001 |
| Congo | death | 2011 | 2021 | -0.44 (-0.52 to -0.36) | <0.001 |
| Cook Islands | death | 1990 | 1994 | -2.20 (-2.39 to -2.02) | <0.001 |
| Cook Islands | death | 1994 | 2000 | -1.51 (-1.63 to -1.38) | <0.001 |
| Cook Islands | death | 2000 | 2003 | -4.96 (-5.49 to -4.43) | <0.001 |
| Cook Islands | death | 2003 | 2008 | -2.91 (-3.10 to -2.73) | <0.001 |
| Cook Islands | death | 2008 | 2015 | -0.49 (-0.59 to -0.38) | <0.001 |
| Cook Islands | death | 2015 | 2021 | -1.68 (-1.80 to -1.56) | <0.001 |
| Costa Rica | death | 1990 | 1992 | 4.75 (-4.16 to 14.49) | 0.29 |
| Costa Rica | death | 1992 | 2001 | -2.99 (-3.87 to -2.11) | <0.001 |
| Costa Rica | death | 2001 | 2007 | -6.04 (-7.89 to -4.16) | <0.001 |
| Costa Rica | death | 2007 | 2021 | 0.74 (0.24 to 1.25) | 0.006 |
| Côte d'Ivoire | death | 1990 | 1995 | 1.48 (1.11 to 1.86) | <0.001 |
| Côte d'Ivoire | death | 1995 | 2004 | 0.46 (0.27 to 0.66) | <0.001 |
| Côte d'Ivoire | death | 2004 | 2015 | -0.87 (-1.01 to -0.73) | <0.001 |
| Côte d'Ivoire | death | 2015 | 2021 | -1.45 (-1.79 to -1.11) | <0.001 |
| Croatia | death | 1990 | 1997 | -1.82 (-2.70 to -0.94) | <0.001 |
| Croatia | death | 1997 | 2021 | -3.94 (-4.10 to -3.77) | <0.001 |
| Cuba | death | 1990 | 2021 | -0.81 (-0.95 to -0.68) | <0.001 |
| Cyprus | death | 1990 | 1992 | 1.34 (-4.17 to 7.17) | 0.619 |
| Cyprus | death | 1992 | 1995 | -7.31 (-12.18 to -2.19) | 0.009 |
| Cyprus | death | 1995 | 1998 | -3.16 (-8.66 to 2.68) | 0.26 |
| Cyprus | death | 1998 | 2006 | -7.89 (-8.50 to -7.29) | <0.001 |
| Cyprus | death | 2006 | 2011 | -1.23 (-2.83 to 0.40) | 0.128 |
| Cyprus | death | 2011 | 2021 | -2.83 (-3.25 to -2.41) | <0.001 |
| Czechia | death | 1990 | 1992 | -4.95 (-9.47 to -0.21) | 0.042 |
| Czechia | death | 1992 | 2003 | -1.89 (-2.29 to -1.48) | <0.001 |
| Czechia | death | 2003 | 2007 | -10.61 (-13.39 to -7.74) | <0.001 |
| Czechia | death | 2007 | 2014 | -7.89 (-8.98 to -6.79) | <0.001 |
| Czechia | death | 2014 | 2021 | -4.48 (-5.49 to -3.46) | <0.001 |
| Democratic People's Republic of Korea | death | 1990 | 1998 | 0.94 (0.90 to 0.97) | <0.001 |
| Democratic People's Republic of Korea | death | 1998 | 2003 | 0.53 (0.43 to 0.63) | <0.001 |
| Democratic People's Republic of Korea | death | 2003 | 2009 | -0.38 (-0.44 to -0.32) | <0.001 |
| Democratic People's Republic of Korea | death | 2009 | 2012 | -0.80 (-1.08 to -0.52) | <0.001 |
| Democratic People's Republic of Korea | death | 2012 | 2017 | -1.78 (-1.87 to -1.69) | <0.001 |
| Democratic People's Republic of Korea | death | 2017 | 2021 | -0.94 (-1.04 to -0.83) | <0.001 |
| Democratic Republic of the Congo | death | 1990 | 2005 | -0.44 (-0.52 to -0.35) | <0.001 |
| Democratic Republic of the Congo | death | 2005 | 2008 | 0.09 (-2.05 to 2.28) | 0.933 |
| Democratic Republic of the Congo | death | 2008 | 2016 | -0.80 (-1.11 to -0.49) | <0.001 |
| Democratic Republic of the Congo | death | 2016 | 2021 | 0.80 (0.23 to 1.37) | 0.008 |
| Denmark | death | 1990 | 1993 | 0.66 (-0.85 to 2.20) | 0.374 |
| Denmark | death | 1993 | 1999 | -3.90 (-4.61 to -3.18) | <0.001 |
| Denmark | death | 1999 | 2003 | 0.32 (-1.44 to 2.10) | 0.709 |
| Denmark | death | 2003 | 2012 | -5.24 (-5.65 to -4.83) | <0.001 |
| Denmark | death | 2012 | 2021 | -3.16 (-3.56 to -2.76) | <0.001 |
| Djibouti | death | 1990 | 1995 | 0.53 (0.34 to 0.72) | <0.001 |
| Djibouti | death | 1995 | 1999 | -0.17 (-0.55 to 0.22) | 0.373 |
| Djibouti | death | 1999 | 2002 | -0.70 (-1.47 to 0.08) | 0.073 |
| Djibouti | death | 2002 | 2005 | 0.09 (-0.68 to 0.86) | 0.812 |
| Djibouti | death | 2005 | 2013 | -0.63 (-0.74 to -0.53) | <0.001 |
| Djibouti | death | 2013 | 2021 | 0.19 (0.11 to 0.28) | <0.001 |
| Dominica | death | 1990 | 1996 | -0.61 (-0.79 to -0.43) | <0.001 |
| Dominica | death | 1996 | 2001 | -1.61 (-1.97 to -1.25) | <0.001 |
| Dominica | death | 2001 | 2010 | -0.71 (-0.84 to -0.59) | <0.001 |
| Dominica | death | 2010 | 2021 | -0.20 (-0.28 to -0.12) | <0.001 |
| Dominican Republic | death | 1990 | 1994 | -4.75 (-6.17 to -3.30) | <0.001 |
| Dominican Republic | death | 1994 | 2002 | 1.08 (0.49 to 1.68) | 0.001 |
| Dominican Republic | death | 2002 | 2005 | 5.78 (1.21 to 10.56) | 0.016 |
| Dominican Republic | death | 2005 | 2016 | -1.94 (-2.34 to -1.53) | <0.001 |
| Dominican Republic | death | 2016 | 2021 | -0.21 (-1.50 to 1.09) | 0.737 |
| Ecuador | death | 1990 | 1994 | -0.20 (-2.51 to 2.17) | 0.858 |
| Ecuador | death | 1994 | 1997 | -9.30 (-14.51 to -3.77) | 0.003 |
| Ecuador | death | 1997 | 2005 | 1.87 (0.87 to 2.87) | 0.001 |
| Ecuador | death | 2005 | 2011 | -5.47 (-7.08 to -3.82) | <0.001 |
| Ecuador | death | 2011 | 2016 | 1.36 (-1.09 to 3.87) | 0.259 |
| Ecuador | death | 2016 | 2021 | -4.40 (-6.72 to -2.02) | 0.001 |
| Egypt | death | 1990 | 1992 | -4.18 (-8.17 to -0.02) | 0.049 |
| Egypt | death | 1992 | 2000 | -0.76 (-1.24 to -0.28) | 0.004 |
| Egypt | death | 2000 | 2003 | 1.75 (-1.79 to 5.42) | 0.313 |
| Egypt | death | 2003 | 2015 | 0.17 (-0.05 to 0.38) | 0.125 |
| Egypt | death | 2015 | 2018 | -4.31 (-6.67 to -1.88) | 0.002 |
| Egypt | death | 2018 | 2021 | -2.14 (-3.46 to -0.81) | 0.004 |
| El Salvador | death | 1990 | 1994 | 0.70 (-1.57 to 3.02) | 0.531 |
| El Salvador | death | 1994 | 2002 | -4.02 (-4.89 to -3.15) | <0.001 |
| El Salvador | death | 2002 | 2012 | -1.75 (-2.40 to -1.09) | <0.001 |
| El Salvador | death | 2012 | 2015 | 4.05 (-3.40 to 12.06) | 0.276 |
| El Salvador | death | 2015 | 2021 | -1.72 (-3.21 to -0.21) | 0.028 |
| Equatorial Guinea | death | 1990 | 1996 | -0.22 (-0.70 to 0.27) | 0.362 |
| Equatorial Guinea | death | 1996 | 2002 | -2.86 (-3.43 to -2.28) | <0.001 |
| Equatorial Guinea | death | 2002 | 2013 | -1.01 (-1.27 to -0.76) | <0.001 |
| Equatorial Guinea | death | 2013 | 2021 | 1.25 (0.86 to 1.65) | <0.001 |
| Eritrea | death | 1990 | 1993 | -0.87 (-1.27 to -0.47) | <0.001 |
| Eritrea | death | 1993 | 1996 | 0.47 (-0.31 to 1.25) | 0.224 |
| Eritrea | death | 1996 | 2004 | -1.04 (-1.14 to -0.94) | <0.001 |
| Eritrea | death | 2004 | 2015 | -0.24 (-0.30 to -0.19) | <0.001 |
| Eritrea | death | 2015 | 2021 | 0.20 (0.09 to 0.31) | 0.001 |
| Estonia | death | 1990 | 1993 | 2.75 (-0.98 to 6.62) | 0.141 |
| Estonia | death | 1993 | 2005 | -4.16 (-4.68 to -3.64) | <0.001 |
| Estonia | death | 2005 | 2009 | -14.86 (-18.95 to -10.56) | <0.001 |
| Estonia | death | 2009 | 2015 | -9.69 (-12.09 to -7.23) | <0.001 |
| Estonia | death | 2015 | 2021 | 0.57 (-1.74 to 2.93) | 0.616 |
| Eswatini | death | 1990 | 1993 | -0.50 (-1.08 to 0.08) | 0.085 |
| Eswatini | death | 1993 | 1998 | 2.64 (2.27 to 3.01) | <0.001 |
| Eswatini | death | 1998 | 2002 | 4.41 (3.72 to 5.11) | <0.001 |
| Eswatini | death | 2002 | 2006 | 1.09 (0.32 to 1.86) | 0.008 |
| Eswatini | death | 2006 | 2019 | -1.29 (-1.38 to -1.20) | <0.001 |
| Eswatini | death | 2019 | 2021 | -6.66 (-8.07 to -5.23) | <0.001 |
| Ethiopia | death | 1990 | 1998 | -0.29 (-0.39 to -0.18) | <0.001 |
| Ethiopia | death | 1998 | 2003 | -1.74 (-2.02 to -1.46) | <0.001 |
| Ethiopia | death | 2003 | 2006 | -0.82 (-1.63 to 0.00) | 0.05 |
| Ethiopia | death | 2006 | 2012 | -1.62 (-1.79 to -1.45) | <0.001 |
| Ethiopia | death | 2012 | 2015 | -1.23 (-1.94 to -0.53) | 0.002 |
| Ethiopia | death | 2015 | 2021 | 0.52 (0.40 to 0.64) | <0.001 |
| Fiji | death | 1990 | 2000 | 1.89 (1.44 to 2.33) | <0.001 |
| Fiji | death | 2000 | 2007 | -3.43 (-4.16 to -2.69) | <0.001 |
| Fiji | death | 2007 | 2021 | 0.04 (-0.26 to 0.35) | 0.773 |
| Finland | death | 1990 | 1993 | -2.44 (-3.99 to -0.87) | 0.005 |
| Finland | death | 1993 | 1998 | -5.03 (-5.99 to -4.06) | <0.001 |
| Finland | death | 1998 | 2003 | -3.60 (-4.66 to -2.52) | <0.001 |
| Finland | death | 2003 | 2007 | -5.30 (-7.09 to -3.46) | <0.001 |
| Finland | death | 2007 | 2013 | -1.47 (-2.40 to -0.54) | 0.004 |
| Finland | death | 2013 | 2021 | -4.04 (-4.52 to -3.56) | <0.001 |
| France | death | 1990 | 1994 | -5.46 (-6.30 to -4.62) | <0.001 |
| France | death | 1994 | 2003 | -3.55 (-3.87 to -3.23) | <0.001 |
| France | death | 2003 | 2006 | -6.81 (-9.84 to -3.68) | <0.001 |
| France | death | 2006 | 2021 | -2.92 (-3.07 to -2.77) | <0.001 |
| Gabon | death | 1990 | 1992 | -0.99 (-2.99 to 1.05) | 0.319 |
| Gabon | death | 1992 | 1996 | 1.60 (0.60 to 2.61) | 0.003 |
| Gabon | death | 1996 | 2003 | 0.36 (0.02 to 0.70) | 0.038 |
| Gabon | death | 2003 | 2013 | -1.41 (-1.62 to -1.20) | <0.001 |
| Gabon | death | 2013 | 2021 | 0.00 (-0.25 to 0.24) | 0.994 |
| Gambia | death | 1990 | 2002 | 0.51 (0.32 to 0.70) | <0.001 |
| Gambia | death | 2002 | 2005 | 1.99 (-1.09 to 5.16) | 0.196 |
| Gambia | death | 2005 | 2012 | -0.73 (-1.24 to -0.22) | 0.007 |
| Gambia | death | 2012 | 2021 | 0.89 (0.58 to 1.20) | <0.001 |
| Georgia | death | 1990 | 1995 | -5.40 (-9.31 to -1.32) | 0.013 |
| Georgia | death | 1995 | 2001 | 6.51 (2.87 to 10.28) | 0.002 |
| Georgia | death | 2001 | 2004 | -12.83 (-23.52 to -0.65) | 0.041 |
| Georgia | death | 2004 | 2009 | -2.05 (-5.63 to 1.67) | 0.255 |
| Georgia | death | 2009 | 2015 | 8.23 (5.56 to 10.97) | <0.001 |
| Georgia | death | 2015 | 2021 | 3.23 (1.09 to 5.42) | 0.006 |
| Germany | death | 1990 | 1996 | -4.12 (-4.95 to -3.27) | <0.001 |
| Germany | death | 1996 | 2005 | -6.36 (-6.94 to -5.78) | <0.001 |
| Germany | death | 2005 | 2021 | -3.16 (-3.41 to -2.92) | <0.001 |
| Ghana | death | 1990 | 2000 | -0.18 (-0.27 to -0.09) | <0.001 |
| Ghana | death | 2000 | 2007 | 3.08 (2.90 to 3.26) | <0.001 |
| Ghana | death | 2007 | 2016 | -1.37 (-1.48 to -1.27) | <0.001 |
| Ghana | death | 2016 | 2021 | -0.38 (-0.66 to -0.11) | 0.008 |
| Greece | death | 1990 | 2004 | -2.45 (-2.70 to -2.19) | <0.001 |
| Greece | death | 2004 | 2011 | -9.61 (-10.60 to -8.62) | <0.001 |
| Greece | death | 2011 | 2021 | -2.47 (-3.05 to -1.89) | <0.001 |
| Greenland | death | 1990 | 1995 | -1.55 (-2.26 to -0.82) | <0.001 |
| Greenland | death | 1995 | 2000 | -5.22 (-6.31 to -4.12) | <0.001 |
| Greenland | death | 2000 | 2012 | -4.06 (-4.31 to -3.81) | <0.001 |
| Greenland | death | 2012 | 2021 | -1.89 (-2.30 to -1.47) | <0.001 |
| Grenada | death | 1990 | 2000 | -2.47 (-3.29 to -1.63) | <0.001 |
| Grenada | death | 2000 | 2005 | 1.86 (-0.98 to 4.78) | 0.192 |
| Grenada | death | 2005 | 2021 | -2.98 (-3.35 to -2.60) | <0.001 |
| Guam | death | 1990 | 1995 | -8.67 (-10.97 to -6.31) | <0.001 |
| Guam | death | 1995 | 1999 | -0.15 (-5.44 to 5.43) | 0.954 |
| Guam | death | 1999 | 2007 | -7.29 (-8.73 to -5.82) | <0.001 |
| Guam | death | 2007 | 2019 | -0.12 (-1.01 to 0.77) | 0.773 |
| Guam | death | 2019 | 2021 | -14.93 (-26.21 to -1.92) | 0.028 |
| Guatemala | death | 1990 | 1994 | 6.86 (3.16 to 10.69) | 0.001 |
| Guatemala | death | 1994 | 2001 | -2.54 (-3.86 to -1.21) | 0.001 |
| Guatemala | death | 2001 | 2008 | -5.24 (-6.50 to -3.95) | <0.001 |
| Guatemala | death | 2008 | 2011 | 4.72 (-4.13 to 14.38) | 0.287 |
| Guatemala | death | 2011 | 2021 | -3.06 (-3.84 to -2.26) | <0.001 |
| Guinea | death | 1990 | 1995 | 0.16 (-0.04 to 0.37) | 0.113 |
| Guinea | death | 1995 | 2005 | 1.16 (1.09 to 1.23) | <0.001 |
| Guinea | death | 2005 | 2009 | 1.69 (1.31 to 2.08) | <0.001 |
| Guinea | death | 2009 | 2015 | 0.10 (-0.07 to 0.27) | 0.226 |
| Guinea | death | 2015 | 2021 | -1.19 (-1.33 to -1.04) | <0.001 |
| Guinea-Bissau | death | 1990 | 1995 | -0.27 (-0.50 to -0.03) | 0.027 |
| Guinea-Bissau | death | 1995 | 2011 | 0.38 (0.34 to 0.42) | <0.001 |
| Guinea-Bissau | death | 2011 | 2018 | -0.20 (-0.36 to -0.04) | 0.016 |
| Guinea-Bissau | death | 2018 | 2021 | -1.38 (-1.87 to -0.88) | <0.001 |
| Guyana | death | 1990 | 1992 | -8.65 (-12.85 to -4.26) | 0.001 |
| Guyana | death | 1992 | 1997 | 0.90 (-0.35 to 2.17) | 0.147 |
| Guyana | death | 1997 | 2000 | -7.17 (-11.08 to -3.08) | 0.002 |
| Guyana | death | 2000 | 2003 | 8.65 (3.87 to 13.65) | 0.001 |
| Guyana | death | 2003 | 2013 | -2.25 (-2.62 to -1.88) | <0.001 |
| Guyana | death | 2013 | 2021 | -1.16 (-2.00 to -0.31) | 0.011 |
| Haiti | death | 1990 | 1998 | -1.55 (-1.64 to -1.46) | <0.001 |
| Haiti | death | 1998 | 2005 | -0.40 (-0.55 to -0.25) | <0.001 |
| Haiti | death | 2005 | 2021 | -0.71 (-0.76 to -0.67) | <0.001 |
| Honduras | death | 1990 | 1994 | 1.25 (0.38 to 2.12) | 0.007 |
| Honduras | death | 1994 | 1997 | 7.41 (4.65 to 10.25) | <0.001 |
| Honduras | death | 1997 | 2008 | -0.66 (-0.90 to -0.41) | <0.001 |
| Honduras | death | 2008 | 2012 | 5.48 (3.77 to 7.21) | <0.001 |
| Honduras | death | 2012 | 2021 | -1.11 (-1.39 to -0.83) | <0.001 |
| Hungary | death | 1990 | 1993 | -1.10 (-3.00 to 0.84) | 0.248 |
| Hungary | death | 1993 | 2003 | -2.93 (-3.34 to -2.52) | <0.001 |
| Hungary | death | 2003 | 2006 | -8.61 (-13.09 to -3.89) | 0.001 |
| Hungary | death | 2006 | 2016 | -4.01 (-4.52 to -3.50) | <0.001 |
| Hungary | death | 2016 | 2021 | -1.97 (-3.61 to -0.31) | 0.023 |
| Iceland | death | 1990 | 1992 | -5.20 (-10.91 to 0.88) | 0.089 |
| Iceland | death | 1992 | 1995 | 3.08 (-3.50 to 10.11) | 0.351 |
| Iceland | death | 1995 | 2021 | -3.98 (-4.13 to -3.83) | <0.001 |
| India | death | 1990 | 1996 | 1.20 (0.44 to 1.96) | 0.004 |
| India | death | 1996 | 2000 | -3.98 (-5.92 to -2.00) | 0.001 |
| India | death | 2000 | 2008 | 0.46 (-0.05 to 0.98) | 0.075 |
| India | death | 2008 | 2011 | -3.38 (-7.14 to 0.54) | 0.085 |
| India | death | 2011 | 2014 | 3.62 (-0.69 to 8.11) | 0.094 |
| India | death | 2014 | 2021 | -1.29 (-1.83 to -0.75) | <0.001 |
| Indonesia | death | 1990 | 1993 | 2.04 (1.81 to 2.28) | <0.001 |
| Indonesia | death | 1993 | 1998 | 1.51 (1.38 to 1.64) | <0.001 |
| Indonesia | death | 1998 | 2005 | 2.00 (1.93 to 2.07) | <0.001 |
| Indonesia | death | 2005 | 2010 | 1.32 (1.18 to 1.47) | <0.001 |
| Indonesia | death | 2010 | 2014 | -0.51 (-0.76 to -0.26) | 0.001 |
| Indonesia | death | 2014 | 2021 | -0.07 (-0.14 to 0.00) | 0.052 |
| Iran (Islamic Republic of) | death | 1990 | 2003 | -1.75 (-1.83 to -1.66) | <0.001 |
| Iran (Islamic Republic of) | death | 2003 | 2012 | -3.47 (-3.65 to -3.28) | <0.001 |
| Iran (Islamic Republic of) | death | 2012 | 2017 | 0.16 (-0.39 to 0.70) | 0.559 |
| Iran (Islamic Republic of) | death | 2017 | 2021 | -2.49 (-3.05 to -1.92) | <0.001 |
| Iraq | death | 1990 | 1995 | 0.32 (-0.59 to 1.24) | 0.474 |
| Iraq | death | 1995 | 2017 | -1.16 (-1.28 to -1.03) | <0.001 |
| Iraq | death | 2017 | 2021 | 6.48 (4.98 to 8.01) | <0.001 |
| Ireland | death | 1990 | 2000 | -3.61 (-3.90 to -3.31) | <0.001 |
| Ireland | death | 2000 | 2006 | -7.84 (-8.81 to -6.87) | <0.001 |
| Ireland | death | 2006 | 2018 | -2.98 (-3.36 to -2.59) | <0.001 |
| Ireland | death | 2018 | 2021 | -8.08 (-11.43 to -4.60) | <0.001 |
| Israel | death | 1990 | 1996 | -0.58 (-1.96 to 0.83) | 0.403 |
| Israel | death | 1996 | 1999 | -7.14 (-15.08 to 1.53) | 0.1 |
| Israel | death | 1999 | 2021 | -4.48 (-4.74 to -4.23) | <0.001 |
| Italy | death | 1990 | 1999 | -4.24 (-4.69 to -3.78) | <0.001 |
| Italy | death | 1999 | 2005 | -5.81 (-7.11 to -4.48) | <0.001 |
| Italy | death | 2005 | 2013 | -3.97 (-4.87 to -3.06) | <0.001 |
| Italy | death | 2013 | 2021 | -1.96 (-2.75 to -1.17) | <0.001 |
| Jamaica | death | 1990 | 2006 | -2.46 (-3.18 to -1.74) | <0.001 |
| Jamaica | death | 2006 | 2021 | 0.80 (-0.34 to 1.95) | 0.164 |
| Japan | death | 1990 | 1992 | -6.75 (-10.10 to -3.27) | 0.001 |
| Japan | death | 1992 | 1996 | -1.42 (-3.31 to 0.51) | 0.138 |
| Japan | death | 1996 | 2002 | -6.97 (-7.86 to -6.06) | <0.001 |
| Japan | death | 2002 | 2017 | -4.38 (-4.61 to -4.15) | <0.001 |
| Japan | death | 2017 | 2021 | -2.38 (-4.12 to -0.61) | 0.012 |
| Jordan | death | 1990 | 2006 | -0.88 (-1.19 to -0.57) | <0.001 |
| Jordan | death | 2006 | 2009 | -10.90 (-16.87 to -4.51) | 0.002 |
| Jordan | death | 2009 | 2021 | -2.20 (-2.68 to -1.73) | <0.001 |
| Kazakhstan | death | 1990 | 1995 | 6.41 (4.67 to 8.19) | <0.001 |
| Kazakhstan | death | 1995 | 2008 | -1.02 (-1.43 to -0.61) | <0.001 |
| Kazakhstan | death | 2008 | 2011 | 3.47 (-2.84 to 10.18) | 0.27 |
| Kazakhstan | death | 2011 | 2014 | -9.84 (-15.52 to -3.77) | 0.004 |
| Kazakhstan | death | 2014 | 2021 | -1.51 (-2.51 to -0.51) | 0.006 |
| Kenya | death | 1990 | 1999 | -0.11 (-0.21 to 0.00) | 0.048 |
| Kenya | death | 1999 | 2006 | 1.20 (1.02 to 1.39) | <0.001 |
| Kenya | death | 2006 | 2010 | 1.75 (1.18 to 2.32) | <0.001 |
| Kenya | death | 2010 | 2021 | -0.16 (-0.24 to -0.08) | <0.001 |
| Kiribati | death | 1990 | 1999 | 0.78 (0.72 to 0.83) | <0.001 |
| Kiribati | death | 1999 | 2005 | -0.29 (-0.41 to -0.18) | <0.001 |
| Kiribati | death | 2005 | 2011 | 0.39 (0.28 to 0.51) | <0.001 |
| Kiribati | death | 2011 | 2021 | -0.11 (-0.16 to -0.07) | <0.001 |
| Kuwait | death | 1990 | 1992 | -11.55 (-25.97 to 5.68) | 0.165 |
| Kuwait | death | 1992 | 2008 | 5.41 (4.69 to 6.14) | <0.001 |
| Kuwait | death | 2008 | 2011 | -18.62 (-30.84 to -4.24) | 0.016 |
| Kuwait | death | 2011 | 2018 | -5.65 (-8.41 to -2.82) | 0.001 |
| Kuwait | death | 2018 | 2021 | 5.02 (-7.34 to 19.04) | 0.422 |
| Kyrgyzstan | death | 1990 | 1993 | 7.12 (3.69 to 10.66) | <0.001 |
| Kyrgyzstan | death | 1993 | 1998 | -2.73 (-4.45 to -0.98) | 0.004 |
| Kyrgyzstan | death | 1998 | 2005 | -0.73 (-1.64 to 0.19) | 0.114 |
| Kyrgyzstan | death | 2005 | 2021 | -4.12 (-4.34 to -3.89) | <0.001 |
| Lao People's Democratic Republic | death | 1990 | 1998 | -0.87 (-0.95 to -0.79) | <0.001 |
| Lao People's Democratic Republic | death | 1998 | 2011 | -1.65 (-1.69 to -1.61) | <0.001 |
| Lao People's Democratic Republic | death | 2011 | 2015 | -0.18 (-0.53 to 0.17) | 0.301 |
| Lao People's Democratic Republic | death | 2015 | 2021 | -0.43 (-0.57 to -0.30) | <0.001 |
| Latvia | death | 1990 | 1994 | 3.76 (1.21 to 6.37) | 0.006 |
| Latvia | death | 1994 | 1999 | -4.13 (-6.47 to -1.73) | 0.002 |
| Latvia | death | 1999 | 2002 | 2.15 (-6.19 to 11.24) | 0.605 |
| Latvia | death | 2002 | 2011 | -4.59 (-5.57 to -3.59) | <0.001 |
| Latvia | death | 2011 | 2021 | -0.17 (-1.10 to 0.78) | 0.71 |
| Lebanon | death | 1990 | 1999 | -2.95 (-3.17 to -2.73) | <0.001 |
| Lebanon | death | 1999 | 2004 | -7.02 (-7.67 to -6.36) | <0.001 |
| Lebanon | death | 2004 | 2009 | -2.17 (-2.77 to -1.57) | <0.001 |
| Lebanon | death | 2009 | 2014 | -1.24 (-1.77 to -0.72) | <0.001 |
| Lebanon | death | 2014 | 2018 | -3.92 (-4.69 to -3.15) | <0.001 |
| Lebanon | death | 2018 | 2021 | -0.44 (-1.40 to 0.53) | 0.35 |
| Lesotho | death | 1990 | 1997 | -0.40 (-0.94 to 0.13) | 0.132 |
| Lesotho | death | 1997 | 2000 | 11.29 (6.90 to 15.86) | <0.001 |
| Lesotho | death | 2000 | 2005 | 5.26 (4.17 to 6.35) | <0.001 |
| Lesotho | death | 2005 | 2019 | 0.30 (0.12 to 0.48) | 0.003 |
| Lesotho | death | 2019 | 2021 | -5.94 (-9.16 to -2.61) | 0.002 |
| Liberia | death | 1990 | 1998 | 0.77 (0.60 to 0.95) | <0.001 |
| Liberia | death | 1998 | 2006 | -0.79 (-1.03 to -0.54) | <0.001 |
| Liberia | death | 2006 | 2011 | 0.93 (0.32 to 1.54) | 0.005 |
| Liberia | death | 2011 | 2018 | -1.18 (-1.52 to -0.84) | <0.001 |
| Liberia | death | 2018 | 2021 | -0.07 (-1.15 to 1.02) | 0.894 |
| Libya | death | 1990 | 1996 | -2.99 (-3.88 to -2.09) | <0.001 |
| Libya | death | 1996 | 2002 | 4.48 (3.37 to 5.60) | <0.001 |
| Libya | death | 2002 | 2007 | -0.36 (-1.66 to 0.95) | 0.569 |
| Libya | death | 2007 | 2010 | 2.34 (-1.43 to 6.27) | 0.212 |
| Libya | death | 2010 | 2021 | -0.19 (-0.51 to 0.13) | 0.235 |
| Lithuania | death | 1990 | 1994 | 5.65 (3.54 to 7.81) | <0.001 |
| Lithuania | death | 1994 | 2000 | -2.35 (-3.78 to -0.89) | 0.003 |
| Lithuania | death | 2000 | 2007 | 0.44 (-0.68 to 1.56) | 0.425 |
| Lithuania | death | 2007 | 2021 | -3.10 (-3.47 to -2.73) | <0.001 |
| Luxembourg | death | 1990 | 1994 | -7.32 (-8.39 to -6.23) | <0.001 |
| Luxembourg | death | 1994 | 2007 | -5.77 (-6.03 to -5.52) | <0.001 |
| Luxembourg | death | 2007 | 2010 | -3.02 (-8.14 to 2.39) | 0.251 |
| Luxembourg | death | 2010 | 2013 | -8.10 (-13.07 to -2.83) | 0.005 |
| Luxembourg | death | 2013 | 2021 | -4.40 (-5.08 to -3.72) | <0.001 |
| Madagascar | death | 1990 | 2004 | -0.26 (-0.37 to -0.15) | <0.001 |
| Madagascar | death | 2004 | 2011 | -0.91 (-1.38 to -0.43) | 0.001 |
| Madagascar | death | 2011 | 2021 | 0.32 (0.05 to 0.59) | 0.024 |
| Malawi | death | 1990 | 1993 | 1.25 (0.47 to 2.03) | 0.004 |
| Malawi | death | 1993 | 1998 | 2.63 (2.12 to 3.13) | <0.001 |
| Malawi | death | 1998 | 2004 | 0.48 (0.14 to 0.82) | 0.008 |
| Malawi | death | 2004 | 2010 | -0.67 (-1.00 to -0.34) | 0.001 |
| Malawi | death | 2010 | 2013 | -1.94 (-3.33 to -0.53) | 0.01 |
| Malawi | death | 2013 | 2021 | 0.35 (0.17 to 0.53) | 0.001 |
| Malaysia | death | 1990 | 2015 | -0.17 (-0.40 to 0.05) | 0.129 |
| Malaysia | death | 2015 | 2021 | -2.58 (-4.64 to -0.47) | 0.018 |
| Maldives | death | 1990 | 1997 | -1.25 (-1.81 to -0.69) | <0.001 |
| Maldives | death | 1997 | 2011 | -2.89 (-3.06 to -2.73) | <0.001 |
| Maldives | death | 2011 | 2021 | -2.28 (-2.57 to -1.99) | <0.001 |
| Mali | death | 1990 | 1996 | -0.57 (-0.84 to -0.30) | <0.001 |
| Mali | death | 1996 | 1999 | -1.77 (-3.33 to -0.19) | 0.031 |
| Mali | death | 1999 | 2005 | -0.19 (-0.55 to 0.18) | 0.294 |
| Mali | death | 2005 | 2013 | 1.05 (0.84 to 1.26) | <0.001 |
| Mali | death | 2013 | 2019 | -0.17 (-0.49 to 0.16) | 0.286 |
| Mali | death | 2019 | 2021 | -1.37 (-2.91 to 0.19) | 0.081 |
| Malta | death | 1990 | 1995 | -6.00 (-7.55 to -4.42) | <0.001 |
| Malta | death | 1995 | 2005 | -2.44 (-3.20 to -1.69) | <0.001 |
| Malta | death | 2005 | 2021 | -5.89 (-6.29 to -5.49) | <0.001 |
| Marshall Islands | death | 1990 | 1992 | 0.81 (-1.18 to 2.84) | 0.411 |
| Marshall Islands | death | 1992 | 2006 | -0.31 (-0.41 to -0.21) | <0.001 |
| Marshall Islands | death | 2006 | 2021 | -0.84 (-0.92 to -0.76) | <0.001 |
| Mauritania | death | 1990 | 1996 | -1.47 (-1.70 to -1.25) | <0.001 |
| Mauritania | death | 1996 | 1999 | -2.52 (-3.95 to -1.07) | 0.002 |
| Mauritania | death | 1999 | 2008 | -1.05 (-1.21 to -0.89) | <0.001 |
| Mauritania | death | 2008 | 2016 | -0.23 (-0.42 to -0.05) | 0.018 |
| Mauritania | death | 2016 | 2021 | 1.48 (1.14 to 1.82) | <0.001 |
| Mauritius | death | 1990 | 1996 | 1.14 (0.09 to 2.20) | 0.035 |
| Mauritius | death | 1996 | 1999 | -5.46 (-10.67 to 0.05) | 0.052 |
| Mauritius | death | 1999 | 2004 | -1.78 (-3.48 to -0.06) | 0.044 |
| Mauritius | death | 2004 | 2007 | -15.95 (-21.45 to -10.07) | <0.001 |
| Mauritius | death | 2007 | 2013 | -4.56 (-6.06 to -3.04) | <0.001 |
| Mauritius | death | 2013 | 2021 | -0.45 (-1.26 to 0.37) | 0.261 |
| Mexico | death | 1990 | 1993 | -4.34 (-5.95 to -2.70) | <0.001 |
| Mexico | death | 1993 | 1996 | 0.11 (-3.43 to 3.77) | 0.95 |
| Mexico | death | 1996 | 2000 | -5.26 (-7.02 to -3.47) | <0.001 |
| Mexico | death | 2000 | 2006 | -3.58 (-4.44 to -2.71) | <0.001 |
| Mexico | death | 2006 | 2009 | -0.19 (-4.28 to 4.07) | 0.924 |
| Mexico | death | 2009 | 2021 | -2.50 (-2.79 to -2.21) | <0.001 |
| Micronesia (Federated States of) | death | 1990 | 1994 | -0.33 (-0.41 to -0.26) | <0.001 |
| Micronesia (Federated States of) | death | 1994 | 2000 | -0.82 (-0.87 to -0.77) | <0.001 |
| Micronesia (Federated States of) | death | 2000 | 2003 | -1.42 (-1.64 to -1.20) | <0.001 |
| Micronesia (Federated States of) | death | 2003 | 2006 | -1.09 (-1.31 to -0.87) | <0.001 |
| Micronesia (Federated States of) | death | 2006 | 2018 | -0.31 (-0.33 to -0.30) | <0.001 |
| Micronesia (Federated States of) | death | 2018 | 2021 | -0.17 (-0.28 to -0.05) | 0.009 |
| Monaco | death | 1990 | 1997 | -2.31 (-2.49 to -2.14) | <0.001 |
| Monaco | death | 1997 | 2002 | -6.23 (-6.58 to -5.87) | <0.001 |
| Monaco | death | 2002 | 2008 | -2.46 (-2.70 to -2.21) | <0.001 |
| Monaco | death | 2008 | 2011 | -1.61 (-2.67 to -0.54) | 0.006 |
| Monaco | death | 2011 | 2021 | -2.35 (-2.44 to -2.25) | <0.001 |
| Mongolia | death | 1990 | 2005 | 2.06 (1.65 to 2.46) | <0.001 |
| Mongolia | death | 2005 | 2010 | -1.10 (-3.71 to 1.59) | 0.404 |
| Mongolia | death | 2010 | 2021 | -2.57 (-3.16 to -1.98) | <0.001 |
| Montenegro | death | 1990 | 1996 | -0.71 (-1.99 to 0.59) | 0.265 |
| Montenegro | death | 1996 | 1999 | 5.61 (-1.84 to 13.62) | 0.135 |
| Montenegro | death | 1999 | 2007 | -0.62 (-1.45 to 0.22) | 0.136 |
| Montenegro | death | 2007 | 2016 | 4.73 (3.94 to 5.52) | <0.001 |
| Montenegro | death | 2016 | 2021 | 1.19 (-0.45 to 2.84) | 0.145 |
| Morocco | death | 1990 | 2001 | -0.38 (-0.45 to -0.32) | <0.001 |
| Morocco | death | 2001 | 2007 | 0.85 (0.63 to 1.07) | <0.001 |
| Morocco | death | 2007 | 2010 | -0.56 (-1.53 to 0.43) | 0.248 |
| Morocco | death | 2010 | 2018 | 0.13 (-0.01 to 0.26) | 0.064 |
| Morocco | death | 2018 | 2021 | -1.21 (-1.64 to -0.77) | <0.001 |
| Mozambique | death | 1990 | 1995 | -0.09 (-0.41 to 0.24) | 0.584 |
| Mozambique | death | 1995 | 2004 | 1.13 (0.96 to 1.29) | <0.001 |
| Mozambique | death | 2004 | 2012 | 2.34 (2.12 to 2.56) | <0.001 |
| Mozambique | death | 2012 | 2021 | -0.75 (-0.93 to -0.57) | <0.001 |
| Myanmar | death | 1990 | 2004 | -0.70 (-0.73 to -0.67) | <0.001 |
| Myanmar | death | 2004 | 2007 | -1.47 (-1.96 to -0.99) | <0.001 |
| Myanmar | death | 2007 | 2013 | -2.04 (-2.15 to -1.93) | <0.001 |
| Myanmar | death | 2013 | 2017 | -1.02 (-1.28 to -0.76) | <0.001 |
| Myanmar | death | 2017 | 2021 | 0.00 (-0.20 to 0.19) | 0.973 |
| Namibia | death | 1990 | 2003 | 1.54 (1.43 to 1.65) | <0.001 |
| Namibia | death | 2003 | 2008 | -3.08 (-3.68 to -2.48) | <0.001 |
| Namibia | death | 2008 | 2012 | -1.94 (-3.00 to -0.86) | 0.001 |
| Namibia | death | 2012 | 2019 | -0.06 (-0.46 to 0.35) | 0.775 |
| Namibia | death | 2019 | 2021 | -2.01 (-4.42 to 0.45) | 0.102 |
| Nauru | death | 1990 | 1993 | 1.24 (0.93 to 1.55) | <0.001 |
| Nauru | death | 1993 | 1998 | 1.95 (1.79 to 2.12) | <0.001 |
| Nauru | death | 1998 | 2002 | 0.47 (0.22 to 0.72) | 0.001 |
| Nauru | death | 2002 | 2010 | -0.78 (-0.85 to -0.71) | <0.001 |
| Nauru | death | 2010 | 2018 | -2.06 (-2.14 to -1.99) | <0.001 |
| Nauru | death | 2018 | 2021 | -1.25 (-1.54 to -0.96) | <0.001 |
| Nepal | death | 1990 | 1994 | -1.03 (-1.33 to -0.72) | <0.001 |
| Nepal | death | 1994 | 2004 | -2.10 (-2.18 to -2.01) | <0.001 |
| Nepal | death | 2004 | 2009 | -0.31 (-0.59 to -0.04) | 0.03 |
| Nepal | death | 2009 | 2013 | 0.32 (-0.10 to 0.74) | 0.13 |
| Nepal | death | 2013 | 2021 | -0.08 (-0.18 to 0.02) | 0.109 |
| Netherlands | death | 1990 | 2002 | -1.67 (-1.87 to -1.48) | <0.001 |
| Netherlands | death | 2002 | 2007 | -7.55 (-8.63 to -6.46) | <0.001 |
| Netherlands | death | 2007 | 2011 | -4.40 (-6.35 to -2.41) | <0.001 |
| Netherlands | death | 2011 | 2015 | 1.35 (-0.84 to 3.58) | 0.214 |
| Netherlands | death | 2015 | 2021 | -2.27 (-3.02 to -1.53) | <0.001 |
| New Zealand | death | 1990 | 2002 | -2.61 (-2.89 to -2.32) | <0.001 |
| New Zealand | death | 2002 | 2009 | -4.68 (-5.56 to -3.79) | <0.001 |
| New Zealand | death | 2009 | 2021 | -1.99 (-2.35 to -1.63) | <0.001 |
| Nicaragua | death | 1990 | 2000 | -2.26 (-2.79 to -1.72) | <0.001 |
| Nicaragua | death | 2000 | 2004 | 2.20 (-1.07 to 5.59) | 0.18 |
| Nicaragua | death | 2004 | 2021 | -2.48 (-2.74 to -2.21) | <0.001 |
| Niger | death | 1990 | 1996 | -0.76 (-0.97 to -0.55) | <0.001 |
| Niger | death | 1996 | 2002 | 0.83 (0.53 to 1.12) | <0.001 |
| Niger | death | 2002 | 2008 | -0.32 (-0.62 to -0.03) | 0.032 |
| Niger | death | 2008 | 2015 | 0.60 (0.40 to 0.81) | <0.001 |
| Niger | death | 2015 | 2021 | -0.29 (-0.48 to -0.10) | 0.006 |
| Nigeria | death | 1990 | 1994 | -0.59 (-0.98 to -0.20) | 0.006 |
| Nigeria | death | 1994 | 1999 | 0.70 (0.31 to 1.09) | 0.002 |
| Nigeria | death | 1999 | 2002 | -0.28 (-1.38 to 0.84) | 0.602 |
| Nigeria | death | 2002 | 2007 | -2.56 (-2.86 to -2.26) | <0.001 |
| Nigeria | death | 2007 | 2015 | -0.81 (-0.93 to -0.70) | <0.001 |
| Nigeria | death | 2015 | 2021 | -1.11 (-1.25 to -0.97) | <0.001 |
| Niue | death | 1990 | 1992 | 1.15 (0.39 to 1.92) | 0.006 |
| Niue | death | 1992 | 1997 | -0.06 (-0.30 to 0.18) | 0.582 |
| Niue | death | 1997 | 2002 | -1.38 (-1.63 to -1.14) | <0.001 |
| Niue | death | 2002 | 2012 | -1.11 (-1.18 to -1.04) | <0.001 |
| Niue | death | 2012 | 2019 | -0.59 (-0.72 to -0.46) | <0.001 |
| Niue | death | 2019 | 2021 | 0.25 (-0.50 to 0.99) | 0.493 |
| North Macedonia | death | 1990 | 1998 | 6.01 (5.53 to 6.50) | <0.001 |
| North Macedonia | death | 1998 | 2001 | 1.71 (-2.06 to 5.62) | 0.358 |
| North Macedonia | death | 2001 | 2004 | -4.71 (-8.30 to -0.99) | 0.016 |
| North Macedonia | death | 2004 | 2007 | 3.32 (-0.63 to 7.42) | 0.095 |
| North Macedonia | death | 2007 | 2021 | -2.30 (-2.51 to -2.09) | <0.001 |
| Northern Mariana Islands | death | 1990 | 1999 | -0.16 (-1.44 to 1.14) | 0.801 |
| Northern Mariana Islands | death | 1999 | 2004 | -6.10 (-9.57 to -2.49) | 0.002 |
| Northern Mariana Islands | death | 2004 | 2021 | -0.56 (-1.00 to -0.12) | 0.015 |
| Norway | death | 1990 | 1992 | -1.21 (-3.77 to 1.41) | 0.337 |
| Norway | death | 1992 | 1998 | -3.18 (-3.80 to -2.56) | <0.001 |
| Norway | death | 1998 | 2006 | -5.04 (-5.46 to -4.62) | <0.001 |
| Norway | death | 2006 | 2011 | -2.99 (-4.08 to -1.90) | <0.001 |
| Norway | death | 2011 | 2019 | -6.77 (-7.24 to -6.30) | <0.001 |
| Norway | death | 2019 | 2021 | -0.49 (-4.48 to 3.67) | 0.803 |
| Oman | death | 1990 | 2006 | -2.41 (-2.87 to -1.95) | <0.001 |
| Oman | death | 2006 | 2009 | 5.75 (-3.44 to 15.83) | 0.215 |
| Oman | death | 2009 | 2016 | 1.40 (0.05 to 2.77) | 0.043 |
| Oman | death | 2016 | 2021 | -4.38 (-6.35 to -2.37) | <0.001 |
| Pakistan | death | 1990 | 1996 | 2.63 (2.46 to 2.80) | <0.001 |
| Pakistan | death | 1996 | 2003 | -0.35 (-0.49 to -0.20) | <0.001 |
| Pakistan | death | 2003 | 2008 | -1.25 (-1.51 to -0.99) | <0.001 |
| Pakistan | death | 2008 | 2017 | -0.13 (-0.22 to -0.04) | 0.006 |
| Pakistan | death | 2017 | 2021 | -0.63 (-0.89 to -0.36) | <0.001 |
| Palau | death | 1990 | 1995 | -1.39 (-1.88 to -0.90) | <0.001 |
| Palau | death | 1995 | 2003 | -0.64 (-0.91 to -0.36) | <0.001 |
| Palau | death | 2003 | 2013 | 0.57 (0.39 to 0.75) | <0.001 |
| Palau | death | 2013 | 2021 | -1.25 (-1.48 to -1.02) | <0.001 |
| Palestine | death | 1990 | 2000 | -0.63 (-0.74 to -0.52) | <0.001 |
| Palestine | death | 2000 | 2007 | 0.43 (0.26 to 0.60) | <0.001 |
| Palestine | death | 2007 | 2011 | -2.36 (-2.83 to -1.89) | <0.001 |
| Palestine | death | 2011 | 2014 | -5.95 (-6.91 to -4.99) | <0.001 |
| Palestine | death | 2014 | 2019 | -3.50 (-3.81 to -3.19) | <0.001 |
| Palestine | death | 2019 | 2021 | 0.27 (-1.08 to 1.64) | 0.674 |
| Panama | death | 1990 | 1994 | 2.17 (-0.36 to 4.76) | 0.089 |
| Panama | death | 1994 | 2005 | -2.01 (-2.59 to -1.42) | <0.001 |
| Panama | death | 2005 | 2011 | -3.72 (-5.49 to -1.92) | <0.001 |
| Panama | death | 2011 | 2016 | 1.95 (-0.71 to 4.68) | 0.143 |
| Panama | death | 2016 | 2021 | -3.55 (-6.00 to -1.04) | 0.009 |
| Papua New Guinea | death | 1990 | 2006 | 0.17 (0.13 to 0.20) | <0.001 |
| Papua New Guinea | death | 2006 | 2015 | -1.28 (-1.36 to -1.20) | <0.001 |
| Papua New Guinea | death | 2015 | 2021 | -0.41 (-0.56 to -0.26) | <0.001 |
| Paraguay | death | 1990 | 1996 | -1.29 (-2.29 to -0.28) | 0.015 |
| Paraguay | death | 1996 | 2002 | -3.50 (-4.84 to -2.13) | <0.001 |
| Paraguay | death | 2002 | 2019 | 0.06 (-0.17 to 0.30) | 0.58 |
| Paraguay | death | 2019 | 2021 | -4.46 (-11.99 to 3.73) | 0.262 |
| Peru | death | 1990 | 1995 | 1.66 (-0.58 to 3.95) | 0.139 |
| Peru | death | 1995 | 2007 | -4.86 (-5.47 to -4.25) | <0.001 |
| Peru | death | 2007 | 2016 | -1.52 (-2.73 to -0.30) | 0.018 |
| Peru | death | 2016 | 2019 | 8.66 (-2.66 to 21.29) | 0.13 |
| Peru | death | 2019 | 2021 | -6.02 (-18.72 to 8.67) | 0.381 |
| Philippines | death | 1990 | 1992 | -5.78 (-8.63 to -2.85) | 0.001 |
| Philippines | death | 1992 | 1996 | 1.04 (-0.40 to 2.50) | 0.146 |
| Philippines | death | 1996 | 1999 | -1.45 (-4.10 to 1.28) | 0.276 |
| Philippines | death | 1999 | 2002 | 2.21 (-0.33 to 4.81) | 0.085 |
| Philippines | death | 2002 | 2021 | -0.81 (-0.89 to -0.74) | <0.001 |
| Poland | death | 1990 | 1992 | 0.63 (-2.63 to 4.00) | 0.69 |
| Poland | death | 1992 | 1997 | -2.74 (-3.79 to -1.67) | <0.001 |
| Poland | death | 1997 | 2000 | -6.07 (-9.31 to -2.71) | 0.002 |
| Poland | death | 2000 | 2008 | -3.12 (-3.63 to -2.62) | <0.001 |
| Poland | death | 2008 | 2011 | -6.29 (-10.13 to -2.29) | 0.005 |
| Poland | death | 2011 | 2021 | -3.36 (-3.76 to -2.96) | <0.001 |
| Portugal | death | 1990 | 1999 | -4.05 (-4.33 to -3.77) | <0.001 |
| Portugal | death | 1999 | 2003 | -7.68 (-9.33 to -5.99) | <0.001 |
| Portugal | death | 2003 | 2006 | -10.31 (-13.89 to -6.58) | <0.001 |
| Portugal | death | 2006 | 2010 | -5.33 (-7.37 to -3.24) | <0.001 |
| Portugal | death | 2010 | 2015 | -7.25 (-8.65 to -5.82) | <0.001 |
| Portugal | death | 2015 | 2021 | -3.05 (-3.96 to -2.12) | <0.001 |
| Puerto Rico | death | 1990 | 1992 | -3.08 (-8.25 to 2.38) | 0.243 |
| Puerto Rico | death | 1992 | 1995 | 3.26 (-2.80 to 9.69) | 0.276 |
| Puerto Rico | death | 1995 | 2003 | -6.03 (-6.86 to -5.19) | <0.001 |
| Puerto Rico | death | 2003 | 2011 | -3.03 (-4.05 to -1.99) | <0.001 |
| Puerto Rico | death | 2011 | 2018 | -6.38 (-7.72 to -5.02) | <0.001 |
| Puerto Rico | death | 2018 | 2021 | -2.17 (-7.71 to 3.71) | 0.436 |
| Qatar | death | 1990 | 1992 | 2.50 (-6.66 to 12.57) | 0.582 |
| Qatar | death | 1992 | 1995 | -11.67 (-19.24 to -3.39) | 0.01 |
| Qatar | death | 1995 | 1998 | 12.71 (2.83 to 23.54) | 0.014 |
| Qatar | death | 1998 | 2008 | -1.61 (-2.38 to -0.84) | 0.001 |
| Qatar | death | 2008 | 2016 | -11.13 (-12.32 to -9.91) | <0.001 |
| Qatar | death | 2016 | 2021 | 3.12 (0.41 to 5.89) | 0.026 |
| Republic of Korea | death | 1990 | 2000 | -3.68 (-3.97 to -3.40) | <0.001 |
| Republic of Korea | death | 2000 | 2004 | -5.97 (-7.85 to -4.06) | <0.001 |
| Republic of Korea | death | 2004 | 2009 | -8.37 (-9.79 to -6.92) | <0.001 |
| Republic of Korea | death | 2009 | 2015 | -5.77 (-7.00 to -4.53) | <0.001 |
| Republic of Korea | death | 2015 | 2021 | -2.53 (-3.59 to -1.46) | <0.001 |
| Republic of Moldova | death | 1990 | 1994 | 3.15 (-0.12 to 6.54) | 0.058 |
| Republic of Moldova | death | 1994 | 1998 | -11.91 (-14.88 to -8.84) | <0.001 |
| Republic of Moldova | death | 1998 | 2005 | 3.97 (2.69 to 5.27) | <0.001 |
| Republic of Moldova | death | 2005 | 2015 | -1.75 (-2.42 to -1.08) | <0.001 |
| Republic of Moldova | death | 2015 | 2021 | -5.26 (-6.89 to -3.61) | <0.001 |
| Romania | death | 1990 | 1996 | 1.15 (0.51 to 1.79) | 0.001 |
| Romania | death | 1996 | 2000 | -3.28 (-4.87 to -1.67) | <0.001 |
| Romania | death | 2000 | 2003 | 0.96 (-2.75 to 4.82) | 0.598 |
| Romania | death | 2003 | 2008 | -3.90 (-5.09 to -2.69) | <0.001 |
| Romania | death | 2008 | 2021 | -3.03 (-3.28 to -2.78) | <0.001 |
| Russian Federation | death | 1990 | 1994 | 7.42 (5.10 to 9.80) | <0.001 |
| Russian Federation | death | 1994 | 1997 | -2.89 (-8.83 to 3.45) | 0.343 |
| Russian Federation | death | 1997 | 2003 | 0.99 (-0.50 to 2.50) | 0.18 |
| Russian Federation | death | 2003 | 2012 | -6.39 (-7.11 to -5.67) | <0.001 |
| Russian Federation | death | 2012 | 2021 | -2.97 (-3.82 to -2.12) | <0.001 |
| Rwanda | death | 1990 | 1995 | 0.63 (0.38 to 0.89) | <0.001 |
| Rwanda | death | 1995 | 1998 | -1.82 (-2.99 to -0.64) | 0.005 |
| Rwanda | death | 1998 | 2004 | -6.32 (-6.57 to -6.07) | <0.001 |
| Rwanda | death | 2004 | 2009 | -3.79 (-4.17 to -3.40) | <0.001 |
| Rwanda | death | 2009 | 2016 | -0.50 (-0.73 to -0.27) | <0.001 |
| Rwanda | death | 2016 | 2021 | 1.24 (0.91 to 1.57) | <0.001 |
| Saint Kitts and Nevis | death | 1990 | 1992 | -8.48 (-15.83 to -0.49) | 0.039 |
| Saint Kitts and Nevis | death | 1992 | 1995 | 4.96 (-2.56 to 13.07) | 0.19 |
| Saint Kitts and Nevis | death | 1995 | 1999 | -5.51 (-8.45 to -2.49) | 0.001 |
| Saint Kitts and Nevis | death | 1999 | 2021 | -1.54 (-1.72 to -1.37) | <0.001 |
| Saint Lucia | death | 1990 | 1992 | -0.34 (-7.28 to 7.12) | 0.922 |
| Saint Lucia | death | 1992 | 2000 | -5.00 (-5.89 to -4.09) | <0.001 |
| Saint Lucia | death | 2000 | 2003 | -2.03 (-9.09 to 5.58) | 0.572 |
| Saint Lucia | death | 2003 | 2011 | -6.47 (-7.54 to -5.39) | <0.001 |
| Saint Lucia | death | 2011 | 2021 | 1.35 (0.31 to 2.41) | 0.014 |
| Saint Vincent and the Grenadines | death | 1990 | 2003 | -2.80 (-3.17 to -2.42) | <0.001 |
| Saint Vincent and the Grenadines | death | 2003 | 2012 | 1.26 (0.51 to 2.01) | 0.002 |
| Saint Vincent and the Grenadines | death | 2012 | 2016 | -6.66 (-9.95 to -3.25) | 0.001 |
| Saint Vincent and the Grenadines | death | 2016 | 2021 | 2.19 (0.03 to 4.39) | 0.047 |
| Samoa | death | 1990 | 1996 | -0.23 (-0.30 to -0.16) | <0.001 |
| Samoa | death | 1996 | 1999 | -0.67 (-1.05 to -0.28) | 0.002 |
| Samoa | death | 1999 | 2007 | -1.51 (-1.56 to -1.46) | <0.001 |
| Samoa | death | 2007 | 2011 | -0.51 (-0.72 to -0.30) | <0.001 |
| Samoa | death | 2011 | 2016 | 0.48 (0.34 to 0.62) | <0.001 |
| Samoa | death | 2016 | 2021 | -0.73 (-0.82 to -0.64) | <0.001 |
| San Marino | death | 1990 | 1998 | -2.38 (-3.09 to -1.67) | <0.001 |
| San Marino | death | 1998 | 2009 | -5.23 (-5.74 to -4.71) | <0.001 |
| San Marino | death | 2009 | 2019 | 0.32 (-0.34 to 0.98) | 0.327 |
| San Marino | death | 2019 | 2021 | -24.23 (-32.96 to -14.37) | <0.001 |
| Sao Tome and Principe | death | 1990 | 1995 | -0.02 (-0.56 to 0.52) | 0.936 |
| Sao Tome and Principe | death | 1995 | 1998 | 2.46 (-0.10 to 5.07) | 0.059 |
| Sao Tome and Principe | death | 1998 | 2015 | 0.68 (0.58 to 0.79) | <0.001 |
| Sao Tome and Principe | death | 2015 | 2021 | -0.94 (-1.37 to -0.51) | <0.001 |
| Saudi Arabia | death | 1990 | 1996 | 0.66 (0.42 to 0.90) | <0.001 |
| Saudi Arabia | death | 1996 | 2002 | -1.24 (-1.44 to -1.04) | <0.001 |
| Saudi Arabia | death | 2002 | 2005 | -2.41 (-3.17 to -1.64) | <0.001 |
| Saudi Arabia | death | 2005 | 2008 | -0.60 (-1.42 to 0.23) | 0.143 |
| Saudi Arabia | death | 2008 | 2013 | -2.50 (-2.78 to -2.22) | <0.001 |
| Saudi Arabia | death | 2013 | 2021 | -1.67 (-1.79 to -1.55) | <0.001 |
| Senegal | death | 1990 | 1994 | 0.25 (-0.32 to 0.82) | 0.369 |
| Senegal | death | 1994 | 1999 | -0.94 (-1.47 to -0.40) | 0.002 |
| Senegal | death | 1999 | 2002 | 0.77 (-0.98 to 2.55) | 0.371 |
| Senegal | death | 2002 | 2017 | -0.45 (-0.53 to -0.36) | <0.001 |
| Senegal | death | 2017 | 2021 | 0.75 (-0.04 to 1.53) | 0.06 |
| Serbia | death | 1990 | 1997 | 2.31 (1.81 to 2.81) | <0.001 |
| Serbia | death | 1997 | 2002 | -5.60 (-6.55 to -4.64) | <0.001 |
| Serbia | death | 2002 | 2013 | -3.22 (-3.48 to -2.96) | <0.001 |
| Serbia | death | 2013 | 2021 | -1.59 (-2.08 to -1.10) | <0.001 |
| Seychelles | death | 1990 | 2001 | -2.57 (-3.24 to -1.90) | <0.001 |
| Seychelles | death | 2001 | 2006 | 2.63 (-0.55 to 5.91) | 0.101 |
| Seychelles | death | 2006 | 2021 | -1.34 (-1.78 to -0.91) | <0.001 |
| Sierra Leone | death | 1990 | 1994 | -0.89 (-1.27 to -0.50) | <0.001 |
| Sierra Leone | death | 1994 | 2000 | 0.58 (0.33 to 0.84) | <0.001 |
| Sierra Leone | death | 2000 | 2003 | 1.86 (0.67 to 3.07) | 0.005 |
| Sierra Leone | death | 2003 | 2006 | 0.52 (-0.61 to 1.66) | 0.346 |
| Sierra Leone | death | 2006 | 2010 | -0.43 (-1.03 to 0.18) | 0.152 |
| Sierra Leone | death | 2010 | 2021 | -1.17 (-1.27 to -1.08) | <0.001 |
| Singapore | death | 1990 | 1996 | -3.56 (-5.06 to -2.04) | <0.001 |
| Singapore | death | 1996 | 2013 | -5.43 (-5.85 to -5.01) | <0.001 |
| Singapore | death | 2013 | 2017 | -14.91 (-21.20 to -8.12) | <0.001 |
| Singapore | death | 2017 | 2021 | -7.15 (-12.15 to -1.86) | 0.011 |
| Slovakia | death | 1990 | 1995 | -2.56 (-3.28 to -1.84) | <0.001 |
| Slovakia | death | 1995 | 1998 | -0.59 (-3.83 to 2.75) | 0.707 |
| Slovakia | death | 1998 | 2002 | -3.71 (-5.39 to -2.00) | <0.001 |
| Slovakia | death | 2002 | 2007 | -1.44 (-2.47 to -0.41) | 0.01 |
| Slovakia | death | 2007 | 2013 | -3.34 (-4.14 to -2.55) | <0.001 |
| Slovakia | death | 2013 | 2021 | -1.61 (-2.07 to -1.14) | <0.001 |
| Slovenia | death | 1990 | 2006 | -5.09 (-5.35 to -4.82) | <0.001 |
| Slovenia | death | 2006 | 2009 | 0.04 (-8.41 to 9.27) | 0.992 |
| Slovenia | death | 2009 | 2021 | -3.46 (-4.04 to -2.88) | <0.001 |
| Solomon Islands | death | 1990 | 1993 | -0.06 (-0.92 to 0.82) | 0.897 |
| Solomon Islands | death | 1993 | 2005 | -0.93 (-1.04 to -0.81) | <0.001 |
| Solomon Islands | death | 2005 | 2021 | 0.36 (0.29 to 0.43) | <0.001 |
| Somalia | death | 1990 | 1998 | -0.31 (-0.40 to -0.22) | <0.001 |
| Somalia | death | 1998 | 2003 | -1.21 (-1.47 to -0.95) | <0.001 |
| Somalia | death | 2003 | 2010 | -0.49 (-0.62 to -0.36) | <0.001 |
| Somalia | death | 2010 | 2013 | -1.16 (-1.99 to -0.32) | 0.01 |
| Somalia | death | 2013 | 2019 | 0.01 (-0.18 to 0.20) | 0.918 |
| Somalia | death | 2019 | 2021 | -2.36 (-3.15 to -1.56) | <0.001 |
| South Africa | death | 1990 | 1994 | 1.97 (-0.77 to 4.79) | 0.15 |
| South Africa | death | 1994 | 1997 | 8.59 (1.87 to 15.76) | 0.014 |
| South Africa | death | 1997 | 2003 | 2.51 (1.67 to 3.35) | <0.001 |
| South Africa | death | 2003 | 2010 | 0.52 (-0.10 to 1.14) | 0.095 |
| South Africa | death | 2010 | 2021 | -2.15 (-2.43 to -1.87) | <0.001 |
| South Sudan | death | 1990 | 1995 | -0.72 (-0.92 to -0.52) | <0.001 |
| South Sudan | death | 1995 | 1999 | -1.71 (-2.16 to -1.27) | <0.001 |
| South Sudan | death | 1999 | 2002 | -2.39 (-3.26 to -1.50) | <0.001 |
| South Sudan | death | 2002 | 2011 | -0.82 (-0.91 to -0.72) | <0.001 |
| South Sudan | death | 2011 | 2021 | 0.83 (0.76 to 0.90) | <0.001 |
| Spain | death | 1990 | 1996 | -6.46 (-6.98 to -5.93) | <0.001 |
| Spain | death | 1996 | 2013 | -5.65 (-5.81 to -5.50) | <0.001 |
| Spain | death | 2013 | 2021 | -2.64 (-3.16 to -2.12) | <0.001 |
| Sri Lanka | death | 1990 | 1993 | -4.86 (-7.37 to -2.29) | 0.001 |
| Sri Lanka | death | 1993 | 2002 | -0.83 (-1.44 to -0.22) | 0.011 |
| Sri Lanka | death | 2002 | 2006 | 4.15 (1.52 to 6.84) | 0.004 |
| Sri Lanka | death | 2006 | 2015 | -1.11 (-1.63 to -0.59) | <0.001 |
| Sri Lanka | death | 2015 | 2021 | -3.65 (-5.79 to -1.46) | 0.003 |
| Sudan | death | 1990 | 1995 | -0.41 (-0.52 to -0.31) | <0.001 |
| Sudan | death | 1995 | 2001 | -1.02 (-1.13 to -0.90) | <0.001 |
| Sudan | death | 2001 | 2004 | -0.39 (-0.89 to 0.11) | 0.118 |
| Sudan | death | 2004 | 2012 | -1.39 (-1.45 to -1.32) | <0.001 |
| Sudan | death | 2012 | 2017 | -0.94 (-1.09 to -0.79) | <0.001 |
| Sudan | death | 2017 | 2021 | 0.08 (-0.07 to 0.23) | 0.269 |
| Suriname | death | 1990 | 1993 | 5.01 (2.20 to 7.90) | 0.001 |
| Suriname | death | 1993 | 1996 | -7.17 (-11.85 to -2.23) | 0.007 |
| Suriname | death | 1996 | 2003 | 2.99 (2.14 to 3.85) | <0.001 |
| Suriname | death | 2003 | 2011 | -3.38 (-4.05 to -2.70) | <0.001 |
| Suriname | death | 2011 | 2021 | -0.20 (-0.71 to 0.30) | 0.409 |
| Sweden | death | 1990 | 2002 | -1.55 (-1.79 to -1.31) | <0.001 |
| Sweden | death | 2002 | 2006 | -6.52 (-8.69 to -4.29) | <0.001 |
| Sweden | death | 2006 | 2009 | -0.91 (-5.61 to 4.02) | 0.699 |
| Sweden | death | 2009 | 2021 | -5.25 (-5.54 to -4.95) | <0.001 |
| Switzerland | death | 1990 | 2000 | -5.09 (-5.43 to -4.74) | <0.001 |
| Switzerland | death | 2000 | 2021 | -3.82 (-3.96 to -3.68) | <0.001 |
| Syrian Arab Republic | death | 1990 | 1992 | 4.63 (1.40 to 7.97) | 0.007 |
| Syrian Arab Republic | death | 1992 | 2002 | -0.34 (-0.57 to -0.11) | 0.006 |
| Syrian Arab Republic | death | 2002 | 2005 | -6.72 (-8.65 to -4.75) | <0.001 |
| Syrian Arab Republic | death | 2005 | 2021 | -0.30 (-0.41 to -0.20) | <0.001 |
| Taiwan (Province of China) | death | 1990 | 1993 | -7.22 (-10.30 to -4.04) | <0.001 |
| Taiwan (Province of China) | death | 1993 | 2006 | -4.43 (-4.87 to -3.99) | <0.001 |
| Taiwan (Province of China) | death | 2006 | 2009 | -9.40 (-16.95 to -1.15) | 0.028 |
| Taiwan (Province of China) | death | 2009 | 2021 | -2.12 (-2.70 to -1.53) | <0.001 |
| Tajikistan | death | 1990 | 1994 | 6.83 (5.67 to 8.01) | <0.001 |
| Tajikistan | death | 1994 | 1997 | -5.27 (-8.41 to -2.03) | 0.004 |
| Tajikistan | death | 1997 | 2001 | 0.31 (-1.42 to 2.07) | 0.708 |
| Tajikistan | death | 2001 | 2008 | 2.64 (2.06 to 3.23) | <0.001 |
| Tajikistan | death | 2008 | 2014 | -5.14 (-5.88 to -4.38) | <0.001 |
| Tajikistan | death | 2014 | 2021 | -1.89 (-2.34 to -1.43) | <0.001 |
| Thailand | death | 1990 | 1994 | 0.55 (-0.84 to 1.96) | 0.414 |
| Thailand | death | 1994 | 1998 | -2.84 (-4.82 to -0.81) | 0.009 |
| Thailand | death | 1998 | 2005 | -0.25 (-0.90 to 0.40) | 0.43 |
| Thailand | death | 2005 | 2013 | -4.94 (-5.43 to -4.45) | <0.001 |
| Thailand | death | 2013 | 2017 | -1.75 (-3.83 to 0.37) | 0.099 |
| Thailand | death | 2017 | 2021 | 1.63 (0.00 to 3.28) | 0.05 |
| Timor-Leste | death | 1990 | 1994 | -0.61 (-0.84 to -0.37) | <0.001 |
| Timor-Leste | death | 1994 | 1997 | -1.25 (-1.94 to -0.57) | 0.002 |
| Timor-Leste | death | 1997 | 2004 | -0.08 (-0.21 to 0.04) | 0.181 |
| Timor-Leste | death | 2004 | 2007 | 0.85 (0.06 to 1.64) | 0.036 |
| Timor-Leste | death | 2007 | 2014 | 1.96 (1.80 to 2.11) | <0.001 |
| Timor-Leste | death | 2014 | 2021 | 0.13 (-0.02 to 0.28) | 0.085 |
| Togo | death | 1990 | 1998 | 1.05 (0.90 to 1.19) | <0.001 |
| Togo | death | 1998 | 2009 | 0.13 (0.02 to 0.24) | 0.019 |
| Togo | death | 2009 | 2014 | -1.57 (-2.06 to -1.07) | <0.001 |
| Togo | death | 2014 | 2021 | -0.36 (-0.61 to -0.12) | 0.006 |
| Tokelau | death | 1990 | 1995 | -0.42 (-0.50 to -0.35) | <0.001 |
| Tokelau | death | 1995 | 2000 | -0.99 (-1.10 to -0.89) | <0.001 |
| Tokelau | death | 2000 | 2006 | -1.76 (-1.83 to -1.68) | <0.001 |
| Tokelau | death | 2006 | 2012 | -1.34 (-1.42 to -1.26) | <0.001 |
| Tokelau | death | 2012 | 2016 | -1.53 (-1.71 to -1.35) | <0.001 |
| Tokelau | death | 2016 | 2021 | -1.06 (-1.14 to -0.97) | <0.001 |
| Tonga | death | 1990 | 1996 | -1.35 (-1.73 to -0.96) | <0.001 |
| Tonga | death | 1996 | 1999 | 5.25 (3.14 to 7.41) | <0.001 |
| Tonga | death | 1999 | 2005 | -1.89 (-2.29 to -1.48) | <0.001 |
| Tonga | death | 2005 | 2012 | 0.77 (0.43 to 1.12) | <0.001 |
| Tonga | death | 2012 | 2021 | -0.61 (-0.82 to -0.40) | <0.001 |
| Trinidad and Tobago | death | 1990 | 2011 | -3.23 (-3.38 to -3.08) | <0.001 |
| Trinidad and Tobago | death | 2011 | 2021 | -0.02 (-0.94 to 0.90) | 0.958 |
| Tunisia | death | 1990 | 1996 | 0.61 (0.14 to 1.08) | 0.013 |
| Tunisia | death | 1996 | 1999 | -2.43 (-4.95 to 0.16) | 0.064 |
| Tunisia | death | 1999 | 2003 | 0.25 (-1.21 to 1.72) | 0.728 |
| Tunisia | death | 2003 | 2021 | -1.62 (-1.74 to -1.50) | <0.001 |
| Türkiye | death | 1990 | 1997 | -2.78 (-3.61 to -1.95) | <0.001 |
| Türkiye | death | 1997 | 2005 | -4.36 (-5.28 to -3.44) | <0.001 |
| Türkiye | death | 2005 | 2016 | 0.95 (0.46 to 1.44) | 0.001 |
| Türkiye | death | 2016 | 2021 | -1.68 (-3.17 to -0.17) | 0.031 |
| Turkmenistan | death | 1990 | 1995 | 9.98 (7.61 to 12.42) | <0.001 |
| Turkmenistan | death | 1995 | 1999 | -10.49 (-14.30 to -6.52) | <0.001 |
| Turkmenistan | death | 1999 | 2006 | 7.12 (5.57 to 8.70) | <0.001 |
| Turkmenistan | death | 2006 | 2011 | -5.11 (-7.71 to -2.44) | 0.001 |
| Turkmenistan | death | 2011 | 2021 | -0.29 (-1.26 to 0.69) | 0.543 |
| Tuvalu | death | 1990 | 1994 | -0.45 (-0.53 to -0.37) | <0.001 |
| Tuvalu | death | 1994 | 1997 | 0.05 (-0.19 to 0.28) | 0.662 |
| Tuvalu | death | 1997 | 2000 | -0.90 (-1.12 to -0.67) | <0.001 |
| Tuvalu | death | 2000 | 2005 | -1.67 (-1.75 to -1.60) | <0.001 |
| Tuvalu | death | 2005 | 2015 | -0.49 (-0.51 to -0.47) | <0.001 |
| Tuvalu | death | 2015 | 2021 | -1.06 (-1.09 to -1.02) | <0.001 |
| Uganda | death | 1990 | 1995 | 1.84 (1.63 to 2.05) | <0.001 |
| Uganda | death | 1995 | 2000 | 0.61 (0.33 to 0.89) | <0.001 |
| Uganda | death | 2000 | 2003 | -1.85 (-2.69 to -1.00) | <0.001 |
| Uganda | death | 2003 | 2011 | -3.53 (-3.64 to -3.42) | <0.001 |
| Uganda | death | 2011 | 2017 | -1.08 (-1.27 to -0.89) | <0.001 |
| Uganda | death | 2017 | 2021 | 0.35 (0.08 to 0.62) | 0.015 |
| Ukraine | death | 1990 | 1994 | 3.29 (1.64 to 4.97) | 0.001 |
| Ukraine | death | 1994 | 1998 | -3.16 (-5.51 to -0.76) | 0.013 |
| Ukraine | death | 1998 | 2002 | 0.71 (-1.89 to 3.38) | 0.575 |
| Ukraine | death | 2002 | 2016 | -4.67 (-4.93 to -4.41) | <0.001 |
| Ukraine | death | 2016 | 2021 | -0.13 (-3.19 to 3.02) | 0.93 |
| United Arab Emirates | death | 1990 | 1995 | -5.72 (-11.41 to 0.33) | 0.062 |
| United Arab Emirates | death | 1995 | 2005 | 2.67 (0.43 to 4.96) | 0.022 |
| United Arab Emirates | death | 2005 | 2008 | 12.04 (-6.37 to 34.08) | 0.197 |
| United Arab Emirates | death | 2008 | 2011 | -9.66 (-26.06 to 10.37) | 0.297 |
| United Arab Emirates | death | 2011 | 2017 | 3.89 (-0.98 to 9.00) | 0.111 |
| United Arab Emirates | death | 2017 | 2021 | -13.60 (-20.02 to -6.66) | 0.001 |
| United Kingdom | death | 1990 | 2000 | -3.60 (-3.91 to -3.30) | <0.001 |
| United Kingdom | death | 2000 | 2003 | -0.82 (-5.34 to 3.92) | 0.718 |
| United Kingdom | death | 2003 | 2012 | -7.19 (-7.72 to -6.65) | <0.001 |
| United Kingdom | death | 2012 | 2021 | -3.62 (-4.16 to -3.09) | <0.001 |
| United Republic of Tanzania | death | 1990 | 1995 | 2.83 (2.51 to 3.16) | <0.001 |
| United Republic of Tanzania | death | 1995 | 2002 | 0.10 (-0.11 to 0.32) | 0.319 |
| United Republic of Tanzania | death | 2002 | 2006 | 3.08 (2.51 to 3.66) | <0.001 |
| United Republic of Tanzania | death | 2006 | 2009 | 1.98 (0.92 to 3.06) | 0.001 |
| United Republic of Tanzania | death | 2009 | 2013 | -1.65 (-2.19 to -1.11) | <0.001 |
| United Republic of Tanzania | death | 2013 | 2021 | -0.32 (-0.46 to -0.17) | <0.001 |
| United States of America | death | 1990 | 1999 | -0.22 (-0.65 to 0.21) | 0.304 |
| United States of America | death | 1999 | 2010 | -4.69 (-5.08 to -4.30) | <0.001 |
| United States of America | death | 2010 | 2021 | 0.51 (0.12 to 0.90) | 0.013 |
| United States Virgin Islands | death | 1990 | 1997 | -2.35 (-2.75 to -1.95) | <0.001 |
| United States Virgin Islands | death | 1997 | 2003 | -0.82 (-1.43 to -0.21) | 0.012 |
| United States Virgin Islands | death | 2003 | 2009 | -4.41 (-5.05 to -3.77) | <0.001 |
| United States Virgin Islands | death | 2009 | 2019 | -1.43 (-1.73 to -1.12) | <0.001 |
| United States Virgin Islands | death | 2019 | 2021 | -9.66 (-13.30 to -5.86) | <0.001 |
| Uruguay | death | 1990 | 1994 | -3.31 (-5.01 to -1.58) | 0.001 |
| Uruguay | death | 1994 | 2004 | -1.04 (-1.57 to -0.51) | 0.001 |
| Uruguay | death | 2004 | 2013 | -4.32 (-4.97 to -3.67) | <0.001 |
| Uruguay | death | 2013 | 2021 | -2.76 (-3.49 to -2.02) | <0.001 |
| Uzbekistan | death | 1990 | 1994 | 9.01 (6.63 to 11.44) | <0.001 |
| Uzbekistan | death | 1994 | 1998 | -1.69 (-4.94 to 1.66) | 0.295 |
| Uzbekistan | death | 1998 | 2003 | 2.18 (-0.06 to 4.46) | 0.055 |
| Uzbekistan | death | 2003 | 2014 | -2.93 (-3.45 to -2.40) | <0.001 |
| Uzbekistan | death | 2014 | 2017 | 3.35 (-2.02 to 9.01) | 0.208 |
| Uzbekistan | death | 2017 | 2021 | -3.51 (-5.88 to -1.07) | 0.008 |
| Vanuatu | death | 1990 | 1999 | -0.12 (-0.20 to -0.04) | 0.004 |
| Vanuatu | death | 1999 | 2004 | -1.63 (-1.87 to -1.38) | <0.001 |
| Vanuatu | death | 2004 | 2007 | -0.15 (-0.93 to 0.64) | 0.697 |
| Vanuatu | death | 2007 | 2014 | -1.16 (-1.29 to -1.03) | <0.001 |
| Vanuatu | death | 2014 | 2021 | -0.40 (-0.51 to -0.29) | <0.001 |
| Venezuela (Bolivarian Republic of) | death | 1990 | 2011 | -1.70 (-1.92 to -1.48) | <0.001 |
| Venezuela (Bolivarian Republic of) | death | 2011 | 2021 | 2.01 (0.95 to 3.09) | 0.001 |
| Viet Nam | death | 1990 | 1997 | 0.09 (0.00 to 0.17) | 0.046 |
| Viet Nam | death | 1997 | 2000 | -0.19 (-0.67 to 0.29) | 0.414 |
| Viet Nam | death | 2000 | 2007 | 1.82 (1.74 to 1.90) | <0.001 |
| Viet Nam | death | 2007 | 2010 | 0.72 (0.26 to 1.18) | 0.005 |
| Viet Nam | death | 2010 | 2015 | -0.50 (-0.66 to -0.34) | <0.001 |
| Viet Nam | death | 2015 | 2021 | -0.89 (-0.98 to -0.80) | <0.001 |
| Yemen | death | 1990 | 1995 | -0.19 (-0.40 to 0.02) | 0.068 |
| Yemen | death | 1995 | 2000 | -0.82 (-1.09 to -0.55) | <0.001 |
| Yemen | death | 2000 | 2005 | -0.01 (-0.28 to 0.26) | 0.917 |
| Yemen | death | 2005 | 2012 | -1.13 (-1.27 to -0.98) | <0.001 |
| Yemen | death | 2012 | 2017 | 0.20 (-0.08 to 0.48) | 0.147 |
| Yemen | death | 2017 | 2021 | 1.57 (1.27 to 1.88) | <0.001 |
| Zambia | death | 1990 | 1996 | 2.00 (1.82 to 2.18) | <0.001 |
| Zambia | death | 1996 | 2005 | -0.56 (-0.67 to -0.45) | <0.001 |
| Zambia | death | 2005 | 2008 | 0.29 (-0.70 to 1.30) | 0.54 |
| Zambia | death | 2008 | 2011 | 1.96 (1.03 to 2.90) | <0.001 |
| Zambia | death | 2011 | 2019 | 0.74 (0.62 to 0.86) | <0.001 |
| Zambia | death | 2019 | 2021 | -1.74 (-2.68 to -0.79) | 0.001 |
| Zimbabwe | death | 1990 | 1996 | -0.38 (-1.32 to 0.58) | 0.42 |
| Zimbabwe | death | 1996 | 2001 | 8.53 (6.83 to 10.26) | <0.001 |
| Zimbabwe | death | 2001 | 2005 | 3.44 (0.97 to 5.98) | 0.008 |
| Zimbabwe | death | 2005 | 2021 | -0.96 (-1.15 to -0.77) | <0.001 |
| Afghanistan | DALYs | 1990 | 1992 | 0.12 (-0.67 to 0.92) | 0.753 |
| Afghanistan | DALYs | 1992 | 1996 | 1.09 (0.68 to 1.51) | <0.001 |
| Afghanistan | DALYs | 1996 | 2005 | 0.44 (0.35 to 0.53) | <0.001 |
| Afghanistan | DALYs | 2005 | 2013 | -1.70 (-1.81 to -1.60) | <0.001 |
| Afghanistan | DALYs | 2013 | 2021 | -0.49 (-0.57 to -0.41) | <0.001 |
| Albania | DALYs | 1990 | 1994 | -5.52 (-6.72 to -4.30) | <0.001 |
| Albania | DALYs | 1994 | 2001 | -0.66 (-1.28 to -0.03) | 0.042 |
| Albania | DALYs | 2001 | 2004 | 1.84 (-2.07 to 5.91) | 0.337 |
| Albania | DALYs | 2004 | 2008 | -4.02 (-6.00 to -2.00) | 0.001 |
| Albania | DALYs | 2008 | 2016 | 1.76 (1.10 to 2.43) | <0.001 |
| Albania | DALYs | 2016 | 2021 | -1.04 (-2.21 to 0.16) | 0.084 |
| Algeria | DALYs | 1990 | 1992 | -2.66 (-3.72 to -1.60) | <0.001 |
| Algeria | DALYs | 1992 | 1998 | -1.19 (-1.42 to -0.95) | <0.001 |
| Algeria | DALYs | 1998 | 2003 | -0.68 (-1.01 to -0.35) | <0.001 |
| Algeria | DALYs | 2003 | 2017 | -1.26 (-1.31 to -1.20) | <0.001 |
| Algeria | DALYs | 2017 | 2021 | -0.70 (-1.06 to -0.33) | 0.001 |
| American Samoa | DALYs | 1990 | 1999 | 0.23 (0.03 to 0.42) | 0.024 |
| American Samoa | DALYs | 1999 | 2004 | -3.48 (-4.09 to -2.87) | <0.001 |
| American Samoa | DALYs | 2004 | 2021 | -0.61 (-0.70 to -0.52) | <0.001 |
| Andorra | DALYs | 1990 | 1998 | -1.74 (-2.30 to -1.18) | <0.001 |
| Andorra | DALYs | 1998 | 2007 | -3.75 (-4.25 to -3.25) | <0.001 |
| Andorra | DALYs | 2007 | 2010 | 1.38 (-2.76 to 5.69) | 0.5 |
| Andorra | DALYs | 2010 | 2018 | -0.99 (-1.51 to -0.47) | 0.001 |
| Andorra | DALYs | 2018 | 2021 | -6.09 (-8.31 to -3.82) | <0.001 |
| Angola | DALYs | 1990 | 2000 | -0.19 (-0.33 to -0.06) | 0.007 |
| Angola | DALYs | 2000 | 2015 | -0.87 (-0.94 to -0.79) | <0.001 |
| Angola | DALYs | 2015 | 2021 | 1.07 (0.77 to 1.37) | <0.001 |
| Antigua and Barbuda | DALYs | 1990 | 1994 | 3.80 (1.79 to 5.85) | 0.001 |
| Antigua and Barbuda | DALYs | 1994 | 2000 | -4.91 (-6.13 to -3.67) | <0.001 |
| Antigua and Barbuda | DALYs | 2000 | 2011 | -1.53 (-1.99 to -1.08) | <0.001 |
| Antigua and Barbuda | DALYs | 2011 | 2014 | 1.75 (-4.49 to 8.39) | 0.572 |
| Antigua and Barbuda | DALYs | 2014 | 2021 | -2.47 (-3.36 to -1.58) | <0.001 |
| Argentina | DALYs | 1990 | 1997 | -4.50 (-4.97 to -4.02) | <0.001 |
| Argentina | DALYs | 1997 | 2003 | -2.05 (-2.80 to -1.30) | <0.001 |
| Argentina | DALYs | 2003 | 2006 | -5.18 (-8.70 to -1.52) | 0.009 |
| Argentina | DALYs | 2006 | 2012 | -2.39 (-3.23 to -1.54) | <0.001 |
| Argentina | DALYs | 2012 | 2016 | 0.36 (-1.58 to 2.34) | 0.699 |
| Argentina | DALYs | 2016 | 2021 | -3.50 (-4.38 to -2.62) | <0.001 |
| Armenia | DALYs | 1990 | 1993 | 7.61 (2.95 to 12.48) | 0.003 |
| Armenia | DALYs | 1993 | 1999 | -3.90 (-5.53 to -2.24) | <0.001 |
| Armenia | DALYs | 1999 | 2002 | 3.02 (-4.51 to 11.14) | 0.421 |
| Armenia | DALYs | 2002 | 2014 | -4.32 (-4.82 to -3.83) | <0.001 |
| Armenia | DALYs | 2014 | 2021 | 2.04 (0.62 to 3.48) | 0.007 |
| Australia | DALYs | 1990 | 1995 | -2.11 (-2.89 to -1.32) | <0.001 |
| Australia | DALYs | 1995 | 2005 | -4.84 (-5.22 to -4.45) | <0.001 |
| Australia | DALYs | 2005 | 2018 | -3.23 (-3.52 to -2.93) | <0.001 |
| Australia | DALYs | 2018 | 2021 | -1.53 (-4.20 to 1.22) | 0.258 |
| Austria | DALYs | 1990 | 1994 | -5.04 (-6.13 to -3.94) | <0.001 |
| Austria | DALYs | 1994 | 1998 | -3.18 (-5.08 to -1.25) | 0.003 |
| Austria | DALYs | 1998 | 2005 | -7.09 (-7.78 to -6.38) | <0.001 |
| Austria | DALYs | 2005 | 2011 | -3.64 (-4.84 to -2.42) | <0.001 |
| Austria | DALYs | 2011 | 2021 | -1.76 (-2.26 to -1.27) | <0.001 |
| Azerbaijan | DALYs | 1990 | 1994 | 2.54 (1.70 to 3.38) | <0.001 |
| Azerbaijan | DALYs | 1994 | 1998 | -2.01 (-3.28 to -0.71) | 0.005 |
| Azerbaijan | DALYs | 1998 | 2001 | -0.33 (-2.78 to 2.17) | 0.778 |
| Azerbaijan | DALYs | 2001 | 2004 | 4.67 (1.70 to 7.73) | 0.004 |
| Azerbaijan | DALYs | 2004 | 2017 | -1.35 (-1.51 to -1.18) | <0.001 |
| Azerbaijan | DALYs | 2017 | 2021 | -3.61 (-4.45 to -2.75) | <0.001 |
| Bahamas | DALYs | 1990 | 1997 | -1.07 (-1.60 to -0.53) | 0.001 |
| Bahamas | DALYs | 1997 | 2001 | -3.60 (-5.04 to -2.15) | <0.001 |
| Bahamas | DALYs | 2001 | 2004 | 3.99 (1.08 to 6.97) | 0.01 |
| Bahamas | DALYs | 2004 | 2007 | -5.53 (-8.37 to -2.61) | 0.001 |
| Bahamas | DALYs | 2007 | 2010 | 1.02 (-2.10 to 4.24) | 0.502 |
| Bahamas | DALYs | 2010 | 2021 | -1.33 (-1.62 to -1.03) | <0.001 |
| Bahrain | DALYs | 1990 | 2005 | -0.08 (-0.29 to 0.13) | 0.447 |
| Bahrain | DALYs | 2005 | 2012 | -4.48 (-5.27 to -3.67) | <0.001 |
| Bahrain | DALYs | 2012 | 2015 | -8.48 (-13.55 to -3.11) | 0.004 |
| Bahrain | DALYs | 2015 | 2021 | -0.23 (-1.36 to 0.92) | 0.685 |
| Bangladesh | DALYs | 1990 | 1997 | -0.26 (-1.02 to 0.51) | 0.489 |
| Bangladesh | DALYs | 1997 | 2002 | 1.90 (0.15 to 3.67) | 0.034 |
| Bangladesh | DALYs | 2002 | 2009 | -0.40 (-1.31 to 0.53) | 0.379 |
| Bangladesh | DALYs | 2009 | 2012 | -6.84 (-11.77 to -1.64) | 0.013 |
| Bangladesh | DALYs | 2012 | 2021 | 0.69 (0.13 to 1.26) | 0.018 |
| Barbados | DALYs | 1990 | 1992 | 1.69 (-3.20 to 6.83) | 0.479 |
| Barbados | DALYs | 1992 | 1996 | -1.70 (-3.74 to 0.38) | 0.101 |
| Barbados | DALYs | 1996 | 1999 | -6.59 (-10.80 to -2.19) | 0.007 |
| Barbados | DALYs | 1999 | 2003 | 0.25 (-1.85 to 2.39) | 0.807 |
| Barbados | DALYs | 2003 | 2006 | -4.53 (-8.42 to -0.47) | 0.031 |
| Barbados | DALYs | 2006 | 2021 | -0.70 (-1.02 to -0.39) | <0.001 |
| Belarus | DALYs | 1990 | 1995 | 3.55 (1.77 to 5.36) | <0.001 |
| Belarus | DALYs | 1995 | 2005 | -0.16 (-0.82 to 0.51) | 0.632 |
| Belarus | DALYs | 2005 | 2015 | -4.73 (-5.35 to -4.10) | <0.001 |
| Belarus | DALYs | 2015 | 2021 | -0.92 (-2.53 to 0.72) | 0.254 |
| Belgium | DALYs | 1990 | 2008 | -4.35 (-4.46 to -4.25) | <0.001 |
| Belgium | DALYs | 2008 | 2021 | -2.51 (-2.73 to -2.29) | <0.001 |
| Belize | DALYs | 1990 | 1994 | 0.71 (-1.48 to 2.94) | 0.506 |
| Belize | DALYs | 1994 | 2000 | 7.17 (5.92 to 8.43) | <0.001 |
| Belize | DALYs | 2000 | 2003 | -8.31 (-12.75 to -3.64) | 0.002 |
| Belize | DALYs | 2003 | 2006 | -0.35 (-5.03 to 4.56) | 0.879 |
| Belize | DALYs | 2006 | 2012 | -3.41 (-4.55 to -2.26) | <0.001 |
| Belize | DALYs | 2012 | 2021 | -0.62 (-1.23 to 0.00) | 0.05 |
| Benin | DALYs | 1990 | 2000 | -0.45 (-0.55 to -0.35) | <0.001 |
| Benin | DALYs | 2000 | 2004 | -1.86 (-2.46 to -1.26) | <0.001 |
| Benin | DALYs | 2004 | 2007 | -0.73 (-1.94 to 0.50) | 0.223 |
| Benin | DALYs | 2007 | 2015 | 0.66 (0.49 to 0.84) | <0.001 |
| Benin | DALYs | 2015 | 2018 | -1.39 (-2.61 to -0.16) | 0.03 |
| Benin | DALYs | 2018 | 2021 | -0.46 (-1.08 to 0.17) | 0.137 |
| Bermuda | DALYs | 1990 | 1995 | -3.57 (-4.35 to -2.78) | <0.001 |
| Bermuda | DALYs | 1995 | 1998 | -7.46 (-10.06 to -4.79) | <0.001 |
| Bermuda | DALYs | 1998 | 2002 | -1.23 (-2.88 to 0.45) | 0.141 |
| Bermuda | DALYs | 2002 | 2012 | -3.34 (-3.74 to -2.93) | <0.001 |
| Bermuda | DALYs | 2012 | 2021 | -1.21 (-1.71 to -0.72) | <0.001 |
| Bhutan | DALYs | 1990 | 1995 | -0.51 (-0.79 to -0.24) | 0.001 |
| Bhutan | DALYs | 1995 | 2002 | -1.36 (-1.55 to -1.16) | <0.001 |
| Bhutan | DALYs | 2002 | 2008 | -0.87 (-1.10 to -0.65) | <0.001 |
| Bhutan | DALYs | 2008 | 2021 | -0.27 (-0.31 to -0.22) | <0.001 |
| Bolivia (Plurinational State of) | DALYs | 1990 | 1993 | -1.76 (-2.13 to -1.40) | <0.001 |
| Bolivia (Plurinational State of) | DALYs | 1993 | 1999 | -2.39 (-2.55 to -2.22) | <0.001 |
| Bolivia (Plurinational State of) | DALYs | 1999 | 2004 | -3.04 (-3.26 to -2.82) | <0.001 |
| Bolivia (Plurinational State of) | DALYs | 2004 | 2007 | -1.49 (-2.17 to -0.81) | <0.001 |
| Bolivia (Plurinational State of) | DALYs | 2007 | 2015 | -0.81 (-0.91 to -0.71) | <0.001 |
| Bolivia (Plurinational State of) | DALYs | 2015 | 2021 | -1.34 (-1.48 to -1.20) | <0.001 |
| Bosnia and Herzegovina | DALYs | 1990 | 1993 | 2.89 (0.06 to 5.80) | 0.046 |
| Bosnia and Herzegovina | DALYs | 1993 | 2003 | -2.46 (-3.07 to -1.85) | <0.001 |
| Bosnia and Herzegovina | DALYs | 2003 | 2021 | -0.97 (-1.13 to -0.80) | <0.001 |
| Botswana | DALYs | 1990 | 2003 | -0.44 (-0.77 to -0.11) | 0.013 |
| Botswana | DALYs | 2003 | 2006 | -4.75 (-10.55 to 1.42) | 0.119 |
| Botswana | DALYs | 2006 | 2010 | 3.18 (0.60 to 5.83) | 0.019 |
| Botswana | DALYs | 2010 | 2014 | -1.59 (-3.60 to 0.45) | 0.117 |
| Botswana | DALYs | 2014 | 2018 | -4.74 (-6.70 to -2.74) | <0.001 |
| Botswana | DALYs | 2018 | 2021 | -0.68 (-3.14 to 1.84) | 0.57 |
| Brazil | DALYs | 1990 | 1993 | -2.37 (-3.57 to -1.16) | 0.001 |
| Brazil | DALYs | 1993 | 1999 | -4.96 (-5.49 to -4.42) | <0.001 |
| Brazil | DALYs | 1999 | 2011 | -2.60 (-2.79 to -2.41) | <0.001 |
| Brazil | DALYs | 2011 | 2014 | -3.98 (-6.94 to -0.93) | 0.014 |
| Brazil | DALYs | 2014 | 2021 | -2.00 (-2.43 to -1.57) | <0.001 |
| Brunei Darussalam | DALYs | 1990 | 2004 | -2.68 (-2.88 to -2.48) | <0.001 |
| Brunei Darussalam | DALYs | 2004 | 2007 | -5.04 (-9.25 to -0.65) | 0.027 |
| Brunei Darussalam | DALYs | 2007 | 2012 | -1.04 (-2.41 to 0.34) | 0.13 |
| Brunei Darussalam | DALYs | 2012 | 2016 | 1.96 (-0.20 to 4.17) | 0.072 |
| Brunei Darussalam | DALYs | 2016 | 2021 | -5.02 (-5.99 to -4.04) | <0.001 |
| Bulgaria | DALYs | 1990 | 2003 | -0.65 (-0.99 to -0.30) | 0.001 |
| Bulgaria | DALYs | 2003 | 2006 | 3.41 (-3.92 to 11.31) | 0.356 |
| Bulgaria | DALYs | 2006 | 2021 | -1.32 (-1.70 to -0.93) | <0.001 |
| Burkina Faso | DALYs | 1990 | 1998 | -0.22 (-0.37 to -0.08) | 0.005 |
| Burkina Faso | DALYs | 1998 | 2002 | 1.52 (0.86 to 2.19) | <0.001 |
| Burkina Faso | DALYs | 2002 | 2016 | 0.13 (0.05 to 0.20) | 0.002 |
| Burkina Faso | DALYs | 2016 | 2021 | -1.49 (-1.78 to -1.19) | <0.001 |
| Burundi | DALYs | 1990 | 1996 | -0.13 (-0.37 to 0.11) | 0.255 |
| Burundi | DALYs | 1996 | 1999 | -3.00 (-4.29 to -1.69) | <0.001 |
| Burundi | DALYs | 1999 | 2002 | -4.48 (-5.73 to -3.22) | <0.001 |
| Burundi | DALYs | 2002 | 2008 | -3.46 (-3.74 to -3.18) | <0.001 |
| Burundi | DALYs | 2008 | 2014 | -1.05 (-1.33 to -0.78) | <0.001 |
| Burundi | DALYs | 2014 | 2021 | -0.12 (-0.28 to 0.05) | 0.167 |
| Côte d'Ivoire | DALYs | 1990 | 1995 | 1.85 (1.52 to 2.18) | <0.001 |
| Côte d'Ivoire | DALYs | 1995 | 2004 | 0.31 (0.14 to 0.48) | 0.001 |
| Côte d'Ivoire | DALYs | 2004 | 2011 | -1.37 (-1.62 to -1.11) | <0.001 |
| Côte d'Ivoire | DALYs | 2011 | 2014 | 0.06 (-1.56 to 1.70) | 0.943 |
| Côte d'Ivoire | DALYs | 2014 | 2021 | -1.65 (-1.88 to -1.42) | <0.001 |
| Cabo Verde | DALYs | 1990 | 2005 | 0.05 (-0.08 to 0.17) | 0.428 |
| Cabo Verde | DALYs | 2005 | 2008 | -3.33 (-5.64 to -0.96) | 0.009 |
| Cabo Verde | DALYs | 2008 | 2016 | 0.95 (0.62 to 1.28) | <0.001 |
| Cabo Verde | DALYs | 2016 | 2019 | 9.31 (6.57 to 12.12) | <0.001 |
| Cabo Verde | DALYs | 2019 | 2021 | -2.89 (-5.55 to -0.15) | 0.04 |
| Cambodia | DALYs | 1990 | 1992 | -0.54 (-1.00 to -0.08) | 0.024 |
| Cambodia | DALYs | 1992 | 1995 | 0.21 (-0.22 to 0.64) | 0.324 |
| Cambodia | DALYs | 1995 | 2000 | -0.66 (-0.80 to -0.52) | <0.001 |
| Cambodia | DALYs | 2000 | 2006 | -1.37 (-1.47 to -1.27) | <0.001 |
| Cambodia | DALYs | 2006 | 2010 | -0.12 (-0.34 to 0.10) | 0.272 |
| Cambodia | DALYs | 2010 | 2021 | 0.32 (0.29 to 0.35) | <0.001 |
| Cameroon | DALYs | 1990 | 1995 | 1.76 (1.49 to 2.03) | <0.001 |
| Cameroon | DALYs | 1995 | 2000 | 4.09 (3.68 to 4.49) | <0.001 |
| Cameroon | DALYs | 2000 | 2003 | 2.04 (0.91 to 3.18) | 0.001 |
| Cameroon | DALYs | 2003 | 2008 | -0.34 (-0.66 to -0.01) | 0.043 |
| Cameroon | DALYs | 2008 | 2013 | -1.37 (-1.72 to -1.03) | <0.001 |
| Cameroon | DALYs | 2013 | 2021 | -2.00 (-2.13 to -1.86) | <0.001 |
| Canada | DALYs | 1990 | 1997 | -1.34 (-1.60 to -1.08) | <0.001 |
| Canada | DALYs | 1997 | 2006 | -3.98 (-4.22 to -3.74) | <0.001 |
| Canada | DALYs | 2006 | 2012 | -3.18 (-3.75 to -2.60) | <0.001 |
| Canada | DALYs | 2012 | 2021 | -1.10 (-1.36 to -0.83) | <0.001 |
| Central African Republic | DALYs | 1990 | 1998 | 0.32 (0.21 to 0.43) | <0.001 |
| Central African Republic | DALYs | 1998 | 2004 | -0.52 (-0.75 to -0.29) | <0.001 |
| Central African Republic | DALYs | 2004 | 2009 | -0.89 (-1.22 to -0.56) | <0.001 |
| Central African Republic | DALYs | 2009 | 2021 | -0.56 (-0.62 to -0.50) | <0.001 |
| Chad | DALYs | 1990 | 1992 | 0.31 (-0.95 to 1.60) | 0.606 |
| Chad | DALYs | 1992 | 1999 | 1.95 (1.74 to 2.16) | <0.001 |
| Chad | DALYs | 1999 | 2003 | 1.04 (0.40 to 1.67) | 0.003 |
| Chad | DALYs | 2003 | 2011 | -0.22 (-0.38 to -0.06) | 0.009 |
| Chad | DALYs | 2011 | 2014 | -0.87 (-1.94 to 0.23) | 0.111 |
| Chad | DALYs | 2014 | 2021 | -0.19 (-0.34 to -0.03) | 0.02 |
| Chile | DALYs | 1990 | 1992 | -3.37 (-6.15 to -0.51) | 0.024 |
| Chile | DALYs | 1992 | 1995 | -7.05 (-9.90 to -4.12) | <0.001 |
| Chile | DALYs | 1995 | 2003 | -1.70 (-2.17 to -1.24) | <0.001 |
| Chile | DALYs | 2003 | 2007 | -3.49 (-5.29 to -1.64) | 0.001 |
| Chile | DALYs | 2007 | 2011 | 0.14 (-1.73 to 2.04) | 0.879 |
| Chile | DALYs | 2011 | 2021 | -3.68 (-3.99 to -3.36) | <0.001 |
| China | DALYs | 1990 | 1998 | -0.38 (-0.57 to -0.18) | 0.001 |
| China | DALYs | 1998 | 2004 | 2.18 (1.90 to 2.47) | <0.001 |
| China | DALYs | 2004 | 2007 | -4.10 (-5.11 to -3.08) | <0.001 |
| China | DALYs | 2007 | 2010 | 0.29 (-0.88 to 1.48) | 0.604 |
| China | DALYs | 2010 | 2015 | -1.85 (-2.28 to -1.41) | <0.001 |
| China | DALYs | 2015 | 2021 | -0.88 (-1.19 to -0.57) | <0.001 |
| Colombia | DALYs | 1990 | 1995 | -2.08 (-2.84 to -1.32) | <0.001 |
| Colombia | DALYs | 1995 | 1998 | -8.05 (-11.39 to -4.59) | <0.001 |
| Colombia | DALYs | 1998 | 2015 | -3.66 (-3.81 to -3.50) | <0.001 |
| Colombia | DALYs | 2015 | 2021 | 0.73 (-0.26 to 1.72) | 0.139 |
| Comoros | DALYs | 1990 | 1997 | -0.86 (-1.07 to -0.66) | <0.001 |
| Comoros | DALYs | 1997 | 2005 | -2.81 (-2.99 to -2.63) | <0.001 |
| Comoros | DALYs | 2005 | 2012 | -0.96 (-1.17 to -0.74) | <0.001 |
| Comoros | DALYs | 2012 | 2021 | 0.16 (0.02 to 0.29) | 0.024 |
| Congo | DALYs | 1990 | 1992 | -0.20 (-1.27 to 0.89) | 0.702 |
| Congo | DALYs | 1992 | 1996 | 1.50 (0.92 to 2.08) | <0.001 |
| Congo | DALYs | 1996 | 1999 | -0.99 (-2.12 to 0.16) | 0.086 |
| Congo | DALYs | 1999 | 2002 | -2.18 (-3.26 to -1.08) | 0.001 |
| Congo | DALYs | 2002 | 2011 | -1.30 (-1.41 to -1.19) | <0.001 |
| Congo | DALYs | 2011 | 2021 | -0.51 (-0.59 to -0.43) | <0.001 |
| Cook Islands | DALYs | 1990 | 1994 | -1.89 (-2.02 to -1.77) | <0.001 |
| Cook Islands | DALYs | 1994 | 2000 | -1.34 (-1.43 to -1.26) | <0.001 |
| Cook Islands | DALYs | 2000 | 2003 | -4.04 (-4.39 to -3.70) | <0.001 |
| Cook Islands | DALYs | 2003 | 2008 | -2.27 (-2.39 to -2.16) | <0.001 |
| Cook Islands | DALYs | 2008 | 2014 | -0.21 (-0.30 to -0.12) | <0.001 |
| Cook Islands | DALYs | 2014 | 2021 | -1.08 (-1.14 to -1.02) | <0.001 |
| Costa Rica | DALYs | 1990 | 1992 | 4.04 (-3.97 to 12.72) | 0.316 |
| Costa Rica | DALYs | 1992 | 2001 | -3.00 (-3.76 to -2.23) | <0.001 |
| Costa Rica | DALYs | 2001 | 2007 | -5.33 (-6.97 to -3.67) | <0.001 |
| Costa Rica | DALYs | 2007 | 2021 | 0.42 (0.01 to 0.82) | 0.045 |
| Croatia | DALYs | 1990 | 1997 | -2.19 (-3.08 to -1.30) | <0.001 |
| Croatia | DALYs | 1997 | 2021 | -3.73 (-3.89 to -3.57) | <0.001 |
| Cuba | DALYs | 1990 | 2021 | -0.66 (-0.78 to -0.54) | <0.001 |
| Cyprus | DALYs | 1990 | 1992 | 0.43 (-4.91 to 6.07) | 0.87 |
| Cyprus | DALYs | 1992 | 1995 | -6.78 (-11.53 to -1.77) | 0.011 |
| Cyprus | DALYs | 1995 | 1998 | -2.76 (-8.14 to 2.93) | 0.314 |
| Cyprus | DALYs | 1998 | 2006 | -7.23 (-7.82 to -6.65) | <0.001 |
| Cyprus | DALYs | 2006 | 2021 | -2.92 (-3.13 to -2.71) | <0.001 |
| Czechia | DALYs | 1990 | 1992 | -5.13 (-9.01 to -1.08) | 0.016 |
| Czechia | DALYs | 1992 | 2003 | -2.57 (-2.92 to -2.23) | <0.001 |
| Czechia | DALYs | 2003 | 2007 | -9.33 (-11.70 to -6.90) | <0.001 |
| Czechia | DALYs | 2007 | 2014 | -6.54 (-7.44 to -5.63) | <0.001 |
| Czechia | DALYs | 2014 | 2021 | -3.70 (-4.58 to -2.82) | <0.001 |
| Democratic People's Republic of Korea | DALYs | 1990 | 1999 | 0.94 (0.91 to 0.97) | <0.001 |
| Democratic People's Republic of Korea | DALYs | 1999 | 2003 | 0.52 (0.36 to 0.68) | <0.001 |
| Democratic People's Republic of Korea | DALYs | 2003 | 2010 | -0.34 (-0.39 to -0.29) | <0.001 |
| Democratic People's Republic of Korea | DALYs | 2010 | 2013 | -0.92 (-1.24 to -0.61) | <0.001 |
| Democratic People's Republic of Korea | DALYs | 2013 | 2017 | -1.45 (-1.62 to -1.29) | <0.001 |
| Democratic People's Republic of Korea | DALYs | 2017 | 2021 | -0.81 (-0.93 to -0.69) | <0.001 |
| Democratic Republic of the Congo | DALYs | 1990 | 2017 | -0.58 (-0.62 to -0.54) | <0.001 |
| Democratic Republic of the Congo | DALYs | 2017 | 2021 | 0.58 (-0.23 to 1.40) | 0.152 |
| Denmark | DALYs | 1990 | 1993 | -0.09 (-1.39 to 1.23) | 0.889 |
| Denmark | DALYs | 1993 | 2000 | -3.36 (-3.82 to -2.90) | <0.001 |
| Denmark | DALYs | 2000 | 2003 | 0.32 (-2.59 to 3.31) | 0.824 |
| Denmark | DALYs | 2003 | 2012 | -5.16 (-5.51 to -4.82) | <0.001 |
| Denmark | DALYs | 2012 | 2021 | -2.88 (-3.21 to -2.54) | <0.001 |
| Djibouti | DALYs | 1990 | 1995 | 0.53 (0.32 to 0.74) | <0.001 |
| Djibouti | DALYs | 1995 | 1999 | -0.20 (-0.63 to 0.25) | 0.36 |
| Djibouti | DALYs | 1999 | 2002 | -0.70 (-1.58 to 0.18) | 0.11 |
| Djibouti | DALYs | 2002 | 2005 | 0.09 (-0.80 to 0.99) | 0.833 |
| Djibouti | DALYs | 2005 | 2013 | -0.60 (-0.72 to -0.48) | <0.001 |
| Djibouti | DALYs | 2013 | 2021 | 0.05 (-0.05 to 0.14) | 0.296 |
| Dominica | DALYs | 1990 | 1996 | -0.74 (-0.92 to -0.57) | <0.001 |
| Dominica | DALYs | 1996 | 2001 | -1.81 (-2.16 to -1.46) | <0.001 |
| Dominica | DALYs | 2001 | 2009 | -0.66 (-0.82 to -0.51) | <0.001 |
| Dominica | DALYs | 2009 | 2014 | 0.10 (-0.23 to 0.43) | 0.54 |
| Dominica | DALYs | 2014 | 2021 | -0.29 (-0.45 to -0.13) | 0.001 |
| Dominican Republic | DALYs | 1990 | 1994 | -2.74 (-3.88 to -1.58) | <0.001 |
| Dominican Republic | DALYs | 1994 | 2002 | 0.73 (0.27 to 1.19) | 0.004 |
| Dominican Republic | DALYs | 2002 | 2005 | 4.28 (0.59 to 8.10) | 0.025 |
| Dominican Republic | DALYs | 2005 | 2011 | -1.87 (-2.94 to -0.78) | 0.002 |
| Dominican Republic | DALYs | 2011 | 2021 | -0.22 (-0.55 to 0.12) | 0.192 |
| Ecuador | DALYs | 1990 | 1994 | -0.22 (-2.36 to 1.97) | 0.831 |
| Ecuador | DALYs | 1994 | 1997 | -10.47 (-14.46 to -6.30) | <0.001 |
| Ecuador | DALYs | 1997 | 2005 | 1.41 (0.58 to 2.24) | 0.002 |
| Ecuador | DALYs | 2005 | 2011 | -5.44 (-6.77 to -4.08) | <0.001 |
| Ecuador | DALYs | 2011 | 2016 | 0.80 (-1.29 to 2.93) | 0.431 |
| Ecuador | DALYs | 2016 | 2021 | -3.73 (-5.74 to -1.68) | 0.002 |
| Egypt | DALYs | 1990 | 2000 | -0.98 (-1.30 to -0.66) | <0.001 |
| Egypt | DALYs | 2000 | 2003 | 1.73 (-2.13 to 5.74) | 0.364 |
| Egypt | DALYs | 2003 | 2015 | 0.05 (-0.18 to 0.28) | 0.639 |
| Egypt | DALYs | 2015 | 2018 | -3.98 (-6.37 to -1.53) | 0.003 |
| Egypt | DALYs | 2018 | 2021 | -1.55 (-2.95 to -0.12) | 0.035 |
| El Salvador | DALYs | 1990 | 1994 | 0.28 (-1.82 to 2.42) | 0.784 |
| El Salvador | DALYs | 1994 | 2002 | -4.18 (-4.95 to -3.40) | <0.001 |
| El Salvador | DALYs | 2002 | 2012 | -1.87 (-2.46 to -1.28) | <0.001 |
| El Salvador | DALYs | 2012 | 2015 | 3.57 (-3.16 to 10.76) | 0.287 |
| El Salvador | DALYs | 2015 | 2021 | -1.22 (-2.56 to 0.15) | 0.077 |
| Equatorial Guinea | DALYs | 1990 | 1996 | -0.31 (-0.69 to 0.08) | 0.115 |
| Equatorial Guinea | DALYs | 1996 | 2002 | -3.04 (-3.52 to -2.55) | <0.001 |
| Equatorial Guinea | DALYs | 2002 | 2014 | -1.08 (-1.26 to -0.91) | <0.001 |
| Equatorial Guinea | DALYs | 2014 | 2021 | 1.32 (0.93 to 1.71) | <0.001 |
| Eritrea | DALYs | 1990 | 1993 | -1.17 (-1.53 to -0.80) | <0.001 |
| Eritrea | DALYs | 1993 | 1996 | 0.24 (-0.45 to 0.94) | 0.472 |
| Eritrea | DALYs | 1996 | 2003 | -1.27 (-1.38 to -1.16) | <0.001 |
| Eritrea | DALYs | 2003 | 2014 | -0.43 (-0.48 to -0.38) | <0.001 |
| Eritrea | DALYs | 2014 | 2021 | 0.00 (-0.08 to 0.08) | 0.922 |
| Estonia | DALYs | 1990 | 1994 | 1.47 (-0.65 to 3.63) | 0.165 |
| Estonia | DALYs | 1994 | 2005 | -4.54 (-5.07 to -4.02) | <0.001 |
| Estonia | DALYs | 2005 | 2009 | -13.49 (-17.12 to -9.71) | <0.001 |
| Estonia | DALYs | 2009 | 2015 | -8.11 (-10.20 to -5.98) | <0.001 |
| Estonia | DALYs | 2015 | 2021 | 0.27 (-1.77 to 2.36) | 0.783 |
| Eswatini | DALYs | 1990 | 1993 | -0.64 (-1.16 to -0.12) | 0.019 |
| Eswatini | DALYs | 1993 | 1998 | 2.21 (1.88 to 2.53) | <0.001 |
| Eswatini | DALYs | 1998 | 2002 | 4.77 (4.12 to 5.42) | <0.001 |
| Eswatini | DALYs | 2002 | 2006 | 1.32 (0.59 to 2.06) | 0.002 |
| Eswatini | DALYs | 2006 | 2019 | -1.53 (-1.62 to -1.44) | <0.001 |
| Eswatini | DALYs | 2019 | 2021 | -4.18 (-5.50 to -2.84) | <0.001 |
| Ethiopia | DALYs | 1990 | 1998 | -0.56 (-0.65 to -0.48) | <0.001 |
| Ethiopia | DALYs | 1998 | 2003 | -2.07 (-2.30 to -1.84) | <0.001 |
| Ethiopia | DALYs | 2003 | 2006 | -1.06 (-1.72 to -0.39) | 0.004 |
| Ethiopia | DALYs | 2006 | 2009 | -2.05 (-2.70 to -1.38) | <0.001 |
| Ethiopia | DALYs | 2009 | 2015 | -1.25 (-1.38 to -1.11) | <0.001 |
| Ethiopia | DALYs | 2015 | 2021 | 0.36 (0.26 to 0.46) | <0.001 |
| Fiji | DALYs | 1990 | 2000 | 0.90 (0.58 to 1.23) | <0.001 |
| Fiji | DALYs | 2000 | 2007 | -2.70 (-3.23 to -2.17) | <0.001 |
| Fiji | DALYs | 2007 | 2021 | -0.12 (-0.34 to 0.09) | 0.238 |
| Finland | DALYs | 1990 | 2008 | -3.91 (-4.01 to -3.82) | <0.001 |
| Finland | DALYs | 2008 | 2013 | -1.83 (-3.02 to -0.62) | 0.005 |
| Finland | DALYs | 2013 | 2021 | -3.36 (-3.79 to -2.92) | <0.001 |
| France | DALYs | 1990 | 1994 | -4.83 (-5.43 to -4.22) | <0.001 |
| France | DALYs | 1994 | 2003 | -2.84 (-3.07 to -2.60) | <0.001 |
| France | DALYs | 2003 | 2007 | -4.96 (-6.11 to -3.80) | <0.001 |
| France | DALYs | 2007 | 2021 | -2.22 (-2.34 to -2.09) | <0.001 |
| Gabon | DALYs | 1990 | 1992 | -0.92 (-2.51 to 0.70) | 0.248 |
| Gabon | DALYs | 1992 | 1996 | 1.20 (0.41 to 2.00) | 0.005 |
| Gabon | DALYs | 1996 | 2003 | 0.25 (-0.03 to 0.54) | 0.076 |
| Gabon | DALYs | 2003 | 2013 | -1.38 (-1.54 to -1.22) | <0.001 |
| Gabon | DALYs | 2013 | 2021 | -0.19 (-0.38 to 0.01) | 0.066 |
| Gambia | DALYs | 1990 | 2007 | 0.59 (0.44 to 0.74) | <0.001 |
| Gambia | DALYs | 2007 | 2011 | -1.94 (-3.86 to 0.02) | 0.052 |
| Gambia | DALYs | 2011 | 2021 | 0.81 (0.46 to 1.16) | <0.001 |
| Georgia | DALYs | 1990 | 1995 | -3.39 (-6.50 to -0.18) | 0.04 |
| Georgia | DALYs | 1995 | 2001 | 5.38 (2.71 to 8.12) | 0.001 |
| Georgia | DALYs | 2001 | 2004 | -11.10 (-19.20 to -2.18) | 0.019 |
| Georgia | DALYs | 2004 | 2009 | -0.65 (-3.43 to 2.22) | 0.635 |
| Georgia | DALYs | 2009 | 2018 | 5.04 (4.05 to 6.03) | <0.001 |
| Georgia | DALYs | 2018 | 2021 | -0.43 (-5.23 to 4.62) | 0.856 |
| Germany | DALYs | 1990 | 1997 | -3.88 (-4.27 to -3.50) | <0.001 |
| Germany | DALYs | 1997 | 2000 | -6.55 (-9.48 to -3.53) | <0.001 |
| Germany | DALYs | 2000 | 2007 | -4.31 (-4.87 to -3.75) | <0.001 |
| Germany | DALYs | 2007 | 2021 | -2.22 (-2.39 to -2.03) | <0.001 |
| Ghana | DALYs | 1990 | 1994 | -0.44 (-0.74 to -0.13) | 0.008 |
| Ghana | DALYs | 1994 | 2000 | 0.04 (-0.16 to 0.23) | 0.715 |
| Ghana | DALYs | 2000 | 2007 | 2.57 (2.41 to 2.72) | <0.001 |
| Ghana | DALYs | 2007 | 2014 | -1.24 (-1.38 to -1.10) | <0.001 |
| Ghana | DALYs | 2014 | 2021 | -0.81 (-0.94 to -0.68) | <0.001 |
| Greece | DALYs | 1990 | 1999 | -2.23 (-2.60 to -1.87) | <0.001 |
| Greece | DALYs | 1999 | 2004 | -3.76 (-5.10 to -2.41) | <0.001 |
| Greece | DALYs | 2004 | 2011 | -7.98 (-8.78 to -7.18) | <0.001 |
| Greece | DALYs | 2011 | 2021 | -2.33 (-2.77 to -1.89) | <0.001 |
| Greenland | DALYs | 1990 | 1995 | -1.64 (-2.27 to -1.00) | <0.001 |
| Greenland | DALYs | 1995 | 1999 | -4.78 (-6.21 to -3.32) | <0.001 |
| Greenland | DALYs | 1999 | 2012 | -4.00 (-4.18 to -3.83) | <0.001 |
| Greenland | DALYs | 2012 | 2021 | -2.01 (-2.34 to -1.68) | <0.001 |
| Grenada | DALYs | 1990 | 2000 | -2.92 (-3.74 to -2.09) | <0.001 |
| Grenada | DALYs | 2000 | 2005 | 0.93 (-1.72 to 3.66) | 0.478 |
| Grenada | DALYs | 2005 | 2021 | -2.97 (-3.32 to -2.63) | <0.001 |
| Guam | DALYs | 1990 | 1994 | -5.34 (-6.92 to -3.74) | <0.001 |
| Guam | DALYs | 1994 | 1999 | -1.03 (-2.69 to 0.67) | 0.218 |
| Guam | DALYs | 1999 | 2007 | -4.26 (-4.96 to -3.55) | <0.001 |
| Guam | DALYs | 2007 | 2019 | 0.20 (-0.23 to 0.63) | 0.346 |
| Guam | DALYs | 2019 | 2021 | -7.53 (-14.22 to -0.32) | 0.042 |
| Guatemala | DALYs | 1990 | 1994 | 5.38 (1.89 to 8.99) | 0.004 |
| Guatemala | DALYs | 1994 | 2002 | -3.37 (-4.23 to -2.50) | <0.001 |
| Guatemala | DALYs | 2002 | 2008 | -5.17 (-6.60 to -3.72) | <0.001 |
| Guatemala | DALYs | 2008 | 2011 | 4.30 (-2.73 to 11.84) | 0.221 |
| Guatemala | DALYs | 2011 | 2021 | -2.47 (-3.10 to -1.84) | <0.001 |
| Guinea | DALYs | 1990 | 1996 | 0.25 (0.12 to 0.39) | 0.001 |
| Guinea | DALYs | 1996 | 1999 | 1.46 (0.73 to 2.19) | 0.001 |
| Guinea | DALYs | 1999 | 2004 | 0.94 (0.71 to 1.17) | <0.001 |
| Guinea | DALYs | 2004 | 2009 | 1.51 (1.28 to 1.74) | <0.001 |
| Guinea | DALYs | 2009 | 2015 | 0.09 (-0.06 to 0.25) | 0.228 |
| Guinea | DALYs | 2015 | 2021 | -1.22 (-1.35 to -1.09) | <0.001 |
| Guinea-Bissau | DALYs | 1990 | 1995 | -0.40 (-0.62 to -0.19) | 0.001 |
| Guinea-Bissau | DALYs | 1995 | 2006 | 0.26 (0.19 to 0.32) | <0.001 |
| Guinea-Bissau | DALYs | 2006 | 2014 | -0.16 (-0.26 to -0.05) | 0.008 |
| Guinea-Bissau | DALYs | 2014 | 2019 | -0.60 (-0.88 to -0.32) | <0.001 |
| Guinea-Bissau | DALYs | 2019 | 2021 | -1.45 (-2.36 to -0.53) | 0.004 |
| Guyana | DALYs | 1990 | 1992 | -7.76 (-11.53 to -3.84) | 0.001 |
| Guyana | DALYs | 1992 | 1997 | -0.53 (-1.62 to 0.58) | 0.327 |
| Guyana | DALYs | 1997 | 2000 | -6.55 (-9.89 to -3.09) | 0.001 |
| Guyana | DALYs | 2000 | 2003 | 6.51 (2.53 to 10.64) | 0.003 |
| Guyana | DALYs | 2003 | 2021 | -1.80 (-1.97 to -1.63) | <0.001 |
| Haiti | DALYs | 1990 | 1998 | -1.79 (-1.87 to -1.70) | <0.001 |
| Haiti | DALYs | 1998 | 2005 | -0.39 (-0.55 to -0.24) | <0.001 |
| Haiti | DALYs | 2005 | 2009 | -0.92 (-1.41 to -0.42) | 0.001 |
| Haiti | DALYs | 2009 | 2021 | -0.66 (-0.73 to -0.60) | <0.001 |
| Honduras | DALYs | 1990 | 1993 | -0.74 (-1.88 to 0.41) | 0.191 |
| Honduras | DALYs | 1993 | 1996 | 5.53 (3.08 to 8.04) | <0.001 |
| Honduras | DALYs | 1996 | 2009 | -0.30 (-0.46 to -0.14) | 0.001 |
| Honduras | DALYs | 2009 | 2012 | 6.59 (3.65 to 9.61) | <0.001 |
| Honduras | DALYs | 2012 | 2021 | -1.05 (-1.29 to -0.81) | <0.001 |
| Hungary | DALYs | 1990 | 1993 | -1.20 (-2.84 to 0.46) | 0.144 |
| Hungary | DALYs | 1993 | 2003 | -2.87 (-3.21 to -2.53) | <0.001 |
| Hungary | DALYs | 2003 | 2006 | -8.05 (-11.85 to -4.09) | 0.001 |
| Hungary | DALYs | 2006 | 2016 | -3.82 (-4.22 to -3.42) | <0.001 |
| Hungary | DALYs | 2016 | 2021 | -1.50 (-2.89 to -0.10) | 0.038 |
| Iceland | DALYs | 1990 | 1992 | -4.66 (-8.92 to -0.21) | 0.041 |
| Iceland | DALYs | 1992 | 1996 | 0.44 (-1.88 to 2.82) | 0.7 |
| Iceland | DALYs | 1996 | 2004 | -4.39 (-5.05 to -3.72) | <0.001 |
| Iceland | DALYs | 2004 | 2021 | -3.40 (-3.61 to -3.19) | <0.001 |
| India | DALYs | 1990 | 1996 | 0.85 (0.02 to 1.68) | 0.046 |
| India | DALYs | 1996 | 2000 | -2.93 (-5.09 to -0.72) | 0.012 |
| India | DALYs | 2000 | 2021 | -0.46 (-0.57 to -0.34) | <0.001 |
| Indonesia | DALYs | 1990 | 1993 | 1.55 (1.34 to 1.77) | <0.001 |
| Indonesia | DALYs | 1993 | 1997 | 1.05 (0.86 to 1.25) | <0.001 |
| Indonesia | DALYs | 1997 | 2005 | 1.54 (1.48 to 1.59) | <0.001 |
| Indonesia | DALYs | 2005 | 2010 | 0.99 (0.84 to 1.14) | <0.001 |
| Indonesia | DALYs | 2010 | 2014 | -0.55 (-0.80 to -0.29) | <0.001 |
| Indonesia | DALYs | 2014 | 2021 | -0.21 (-0.29 to -0.14) | <0.001 |
| Iran (Islamic Republic of) | DALYs | 1990 | 2004 | -1.85 (-1.91 to -1.78) | <0.001 |
| Iran (Islamic Republic of) | DALYs | 2004 | 2012 | -3.51 (-3.70 to -3.32) | <0.001 |
| Iran (Islamic Republic of) | DALYs | 2012 | 2017 | -0.05 (-0.54 to 0.45) | 0.845 |
| Iran (Islamic Republic of) | DALYs | 2017 | 2021 | -2.52 (-3.02 to -2.02) | <0.001 |
| Iraq | DALYs | 1990 | 1995 | 0.41 (-0.26 to 1.08) | 0.222 |
| Iraq | DALYs | 1995 | 2017 | -1.39 (-1.48 to -1.30) | <0.001 |
| Iraq | DALYs | 2017 | 2021 | 4.38 (3.19 to 5.58) | <0.001 |
| Ireland | DALYs | 1990 | 1993 | -2.63 (-4.01 to -1.23) | 0.001 |
| Ireland | DALYs | 1993 | 2000 | -3.87 (-4.35 to -3.39) | <0.001 |
| Ireland | DALYs | 2000 | 2006 | -7.91 (-8.66 to -7.16) | <0.001 |
| Ireland | DALYs | 2006 | 2018 | -3.35 (-3.65 to -3.05) | <0.001 |
| Ireland | DALYs | 2018 | 2021 | -5.62 (-8.15 to -3.02) | <0.001 |
| Israel | DALYs | 1990 | 1996 | -0.61 (-1.59 to 0.39) | 0.214 |
| Israel | DALYs | 1996 | 1999 | -7.45 (-12.76 to -1.81) | 0.013 |
| Israel | DALYs | 1999 | 2002 | -2.35 (-8.54 to 4.26) | 0.456 |
| Israel | DALYs | 2002 | 2009 | -4.87 (-6.02 to -3.70) | <0.001 |
| Israel | DALYs | 2009 | 2021 | -3.06 (-3.56 to -2.55) | <0.001 |
| Italy | DALYs | 1990 | 1999 | -4.31 (-4.66 to -3.95) | <0.001 |
| Italy | DALYs | 1999 | 2005 | -6.11 (-7.13 to -5.08) | <0.001 |
| Italy | DALYs | 2005 | 2013 | -3.78 (-4.52 to -3.04) | <0.001 |
| Italy | DALYs | 2013 | 2021 | -1.53 (-2.16 to -0.88) | <0.001 |
| Jamaica | DALYs | 1990 | 2006 | -2.50 (-3.33 to -1.66) | <0.001 |
| Jamaica | DALYs | 2006 | 2021 | 0.90 (-0.38 to 2.20) | 0.161 |
| Japan | DALYs | 1990 | 1992 | -5.90 (-8.23 to -3.51) | <0.001 |
| Japan | DALYs | 1992 | 1995 | -0.17 (-2.78 to 2.50) | 0.891 |
| Japan | DALYs | 1995 | 2004 | -5.04 (-5.34 to -4.74) | <0.001 |
| Japan | DALYs | 2004 | 2016 | -3.44 (-3.67 to -3.21) | <0.001 |
| Japan | DALYs | 2016 | 2021 | -1.64 (-2.51 to -0.76) | 0.001 |
| Jordan | DALYs | 1990 | 2001 | -0.40 (-0.88 to 0.08) | 0.097 |
| Jordan | DALYs | 2001 | 2006 | -2.29 (-4.00 to -0.56) | 0.012 |
| Jordan | DALYs | 2006 | 2009 | -9.18 (-13.23 to -4.95) | <0.001 |
| Jordan | DALYs | 2009 | 2021 | -2.39 (-2.72 to -2.06) | <0.001 |
| Kazakhstan | DALYs | 1990 | 1995 | 6.43 (4.50 to 8.40) | <0.001 |
| Kazakhstan | DALYs | 1995 | 2011 | -0.97 (-1.28 to -0.66) | <0.001 |
| Kazakhstan | DALYs | 2011 | 2015 | -7.46 (-10.62 to -4.20) | <0.001 |
| Kazakhstan | DALYs | 2015 | 2021 | -1.33 (-2.73 to 0.08) | 0.063 |
| Kenya | DALYs | 1990 | 1999 | -0.04 (-0.14 to 0.06) | 0.385 |
| Kenya | DALYs | 1999 | 2010 | 1.12 (1.04 to 1.20) | <0.001 |
| Kenya | DALYs | 2010 | 2021 | -0.37 (-0.44 to -0.30) | <0.001 |
| Kiribati | DALYs | 1990 | 1999 | 0.47 (0.42 to 0.51) | <0.001 |
| Kiribati | DALYs | 1999 | 2005 | -0.48 (-0.58 to -0.38) | <0.001 |
| Kiribati | DALYs | 2005 | 2011 | 0.15 (0.04 to 0.25) | 0.009 |
| Kiribati | DALYs | 2011 | 2021 | -0.28 (-0.32 to -0.24) | <0.001 |
| Kuwait | DALYs | 1990 | 1992 | -10.56 (-24.42 to 5.85) | 0.183 |
| Kuwait | DALYs | 1992 | 2008 | 4.30 (3.66 to 4.93) | <0.001 |
| Kuwait | DALYs | 2008 | 2011 | -16.84 (-28.46 to -3.35) | 0.019 |
| Kuwait | DALYs | 2011 | 2021 | -3.50 (-4.91 to -2.08) | <0.001 |
| Kyrgyzstan | DALYs | 1990 | 1993 | 6.37 (2.31 to 10.59) | 0.003 |
| Kyrgyzstan | DALYs | 1993 | 2006 | -0.97 (-1.31 to -0.62) | <0.001 |
| Kyrgyzstan | DALYs | 2006 | 2013 | -4.61 (-5.53 to -3.68) | <0.001 |
| Kyrgyzstan | DALYs | 2013 | 2021 | -2.68 (-3.56 to -1.80) | <0.001 |
| Lao People's Democratic Republic | DALYs | 1990 | 1998 | -1.11 (-1.19 to -1.03) | <0.001 |
| Lao People's Democratic Republic | DALYs | 1998 | 2011 | -1.72 (-1.76 to -1.68) | <0.001 |
| Lao People's Democratic Republic | DALYs | 2011 | 2021 | -0.54 (-0.60 to -0.48) | <0.001 |
| Latvia | DALYs | 1990 | 1994 | 4.33 (1.70 to 7.02) | 0.003 |
| Latvia | DALYs | 1994 | 1999 | -4.34 (-6.70 to -1.92) | 0.002 |
| Latvia | DALYs | 1999 | 2003 | 0.70 (-3.50 to 5.08) | 0.735 |
| Latvia | DALYs | 2003 | 2011 | -4.52 (-5.70 to -3.33) | <0.001 |
| Latvia | DALYs | 2011 | 2021 | -0.71 (-1.62 to 0.22) | 0.125 |
| Lebanon | DALYs | 1990 | 1999 | -3.22 (-3.41 to -3.03) | <0.001 |
| Lebanon | DALYs | 1999 | 2004 | -6.62 (-7.15 to -6.09) | <0.001 |
| Lebanon | DALYs | 2004 | 2010 | -1.59 (-1.92 to -1.26) | <0.001 |
| Lebanon | DALYs | 2010 | 2014 | -0.85 (-1.46 to -0.23) | 0.011 |
| Lebanon | DALYs | 2014 | 2018 | -3.43 (-4.01 to -2.84) | <0.001 |
| Lebanon | DALYs | 2018 | 2021 | -0.30 (-0.99 to 0.40) | 0.377 |
| Lesotho | DALYs | 1990 | 1997 | -0.34 (-0.77 to 0.10) | 0.12 |
| Lesotho | DALYs | 1997 | 2000 | 9.59 (6.04 to 13.26) | <0.001 |
| Lesotho | DALYs | 2000 | 2005 | 5.27 (4.27 to 6.28) | <0.001 |
| Lesotho | DALYs | 2005 | 2016 | 0.57 (0.30 to 0.84) | <0.001 |
| Lesotho | DALYs | 2016 | 2021 | -1.53 (-2.29 to -0.77) | 0.001 |
| Liberia | DALYs | 1990 | 1998 | 0.54 (0.34 to 0.73) | <0.001 |
| Liberia | DALYs | 1998 | 2006 | -1.07 (-1.33 to -0.80) | <0.001 |
| Liberia | DALYs | 2006 | 2012 | 0.86 (0.38 to 1.35) | 0.001 |
| Liberia | DALYs | 2012 | 2018 | -1.40 (-1.90 to -0.90) | <0.001 |
| Liberia | DALYs | 2018 | 2021 | 0.06 (-1.13 to 1.27) | 0.915 |
| Libya | DALYs | 1990 | 1996 | -2.01 (-2.78 to -1.24) | <0.001 |
| Libya | DALYs | 1996 | 2003 | 3.74 (3.04 to 4.44) | <0.001 |
| Libya | DALYs | 2003 | 2006 | -2.15 (-5.60 to 1.42) | 0.219 |
| Libya | DALYs | 2006 | 2009 | 1.79 (-1.47 to 5.16) | 0.266 |
| Libya | DALYs | 2009 | 2021 | 0.22 (-0.03 to 0.47) | 0.077 |
| Lithuania | DALYs | 1990 | 1994 | 4.68 (2.83 to 6.56) | <0.001 |
| Lithuania | DALYs | 1994 | 2000 | -2.54 (-3.74 to -1.33) | <0.001 |
| Lithuania | DALYs | 2000 | 2004 | -0.27 (-2.98 to 2.51) | 0.835 |
| Lithuania | DALYs | 2004 | 2007 | 3.07 (-2.25 to 8.69) | 0.242 |
| Lithuania | DALYs | 2007 | 2016 | -2.71 (-3.38 to -2.04) | <0.001 |
| Lithuania | DALYs | 2016 | 2021 | -4.93 (-6.65 to -3.18) | <0.001 |
| Luxembourg | DALYs | 1990 | 1994 | -6.92 (-7.71 to -6.13) | <0.001 |
| Luxembourg | DALYs | 1994 | 2004 | -5.63 (-5.91 to -5.35) | <0.001 |
| Luxembourg | DALYs | 2004 | 2007 | -6.92 (-10.46 to -3.25) | 0.001 |
| Luxembourg | DALYs | 2007 | 2010 | -2.73 (-6.56 to 1.25) | 0.161 |
| Luxembourg | DALYs | 2010 | 2013 | -7.82 (-11.62 to -3.86) | 0.001 |
| Luxembourg | DALYs | 2013 | 2021 | -3.55 (-4.05 to -3.05) | <0.001 |
| Madagascar | DALYs | 1990 | 2004 | -0.28 (-0.39 to -0.17) | <0.001 |
| Madagascar | DALYs | 2004 | 2008 | -1.24 (-2.54 to 0.08) | 0.064 |
| Madagascar | DALYs | 2008 | 2021 | 0.00 (-0.17 to 0.17) | 1 |
| Malawi | DALYs | 1990 | 1993 | 1.30 (0.83 to 1.76) | <0.001 |
| Malawi | DALYs | 1993 | 1998 | 2.53 (2.22 to 2.83) | <0.001 |
| Malawi | DALYs | 1998 | 2004 | 0.32 (0.10 to 0.53) | 0.007 |
| Malawi | DALYs | 2004 | 2010 | -0.79 (-0.99 to -0.59) | <0.001 |
| Malawi | DALYs | 2010 | 2013 | -1.85 (-2.70 to -0.99) | <0.001 |
| Malawi | DALYs | 2013 | 2021 | 0.11 (0.00 to 0.22) | 0.049 |
| Malaysia | DALYs | 1990 | 2021 | -0.63 (-0.75 to -0.51) | <0.001 |
| Maldives | DALYs | 1990 | 1996 | -1.28 (-1.96 to -0.60) | 0.001 |
| Maldives | DALYs | 1996 | 2009 | -3.56 (-3.75 to -3.38) | <0.001 |
| Maldives | DALYs | 2009 | 2021 | -2.36 (-2.58 to -2.14) | <0.001 |
| Mali | DALYs | 1990 | 1996 | -0.60 (-0.82 to -0.38) | <0.001 |
| Mali | DALYs | 1996 | 1999 | -1.82 (-3.11 to -0.50) | 0.01 |
| Mali | DALYs | 1999 | 2005 | -0.30 (-0.60 to 0.00) | 0.051 |
| Mali | DALYs | 2005 | 2015 | 0.78 (0.66 to 0.89) | <0.001 |
| Mali | DALYs | 2015 | 2021 | -0.59 (-0.81 to -0.37) | <0.001 |
| Malta | DALYs | 1990 | 1995 | -5.52 (-6.64 to -4.40) | <0.001 |
| Malta | DALYs | 1995 | 2005 | -2.96 (-3.48 to -2.44) | <0.001 |
| Malta | DALYs | 2005 | 2017 | -6.01 (-6.45 to -5.57) | <0.001 |
| Malta | DALYs | 2017 | 2021 | -2.40 (-5.03 to 0.31) | 0.079 |
| Marshall Islands | DALYs | 1990 | 1992 | 1.30 (0.44 to 2.16) | 0.005 |
| Marshall Islands | DALYs | 1992 | 1995 | -0.96 (-1.78 to -0.13) | 0.025 |
| Marshall Islands | DALYs | 1995 | 2005 | -0.11 (-0.18 to -0.03) | 0.007 |
| Marshall Islands | DALYs | 2005 | 2021 | -0.76 (-0.79 to -0.73) | <0.001 |
| Mauritania | DALYs | 1990 | 2001 | -1.88 (-1.98 to -1.77) | <0.001 |
| Mauritania | DALYs | 2001 | 2008 | -1.23 (-1.50 to -0.96) | <0.001 |
| Mauritania | DALYs | 2008 | 2016 | -0.52 (-0.74 to -0.31) | <0.001 |
| Mauritania | DALYs | 2016 | 2021 | 1.23 (0.84 to 1.62) | <0.001 |
| Mauritius | DALYs | 1990 | 1995 | 1.08 (-0.42 to 2.60) | 0.148 |
| Mauritius | DALYs | 1995 | 2004 | -3.19 (-3.85 to -2.52) | <0.001 |
| Mauritius | DALYs | 2004 | 2007 | -15.27 (-21.21 to -8.89) | <0.001 |
| Mauritius | DALYs | 2007 | 2013 | -3.95 (-5.74 to -2.14) | <0.001 |
| Mauritius | DALYs | 2013 | 2021 | -0.21 (-1.15 to 0.75) | 0.653 |
| Mexico | DALYs | 1990 | 1993 | -3.36 (-4.91 to -1.78) | <0.001 |
| Mexico | DALYs | 1993 | 1996 | -1.09 (-4.27 to 2.19) | 0.489 |
| Mexico | DALYs | 1996 | 2006 | -3.70 (-4.02 to -3.38) | <0.001 |
| Mexico | DALYs | 2006 | 2009 | -0.10 (-3.93 to 3.89) | 0.959 |
| Mexico | DALYs | 2009 | 2021 | -2.12 (-2.40 to -1.84) | <0.001 |
| Micronesia (Federated States of) | DALYs | 1990 | 1994 | -0.40 (-0.47 to -0.33) | <0.001 |
| Micronesia (Federated States of) | DALYs | 1994 | 2000 | -0.84 (-0.89 to -0.79) | <0.001 |
| Micronesia (Federated States of) | DALYs | 2000 | 2003 | -1.29 (-1.48 to -1.09) | <0.001 |
| Micronesia (Federated States of) | DALYs | 2003 | 2006 | -1.02 (-1.21 to -0.82) | <0.001 |
| Micronesia (Federated States of) | DALYs | 2006 | 2018 | -0.32 (-0.34 to -0.31) | <0.001 |
| Micronesia (Federated States of) | DALYs | 2018 | 2021 | -0.16 (-0.26 to -0.06) | 0.004 |
| Monaco | DALYs | 1990 | 1997 | -2.34 (-2.46 to -2.22) | <0.001 |
| Monaco | DALYs | 1997 | 2002 | -5.55 (-5.80 to -5.30) | <0.001 |
| Monaco | DALYs | 2002 | 2008 | -2.51 (-2.67 to -2.35) | <0.001 |
| Monaco | DALYs | 2008 | 2011 | -1.75 (-2.42 to -1.07) | <0.001 |
| Monaco | DALYs | 2011 | 2019 | -2.32 (-2.41 to -2.22) | <0.001 |
| Monaco | DALYs | 2019 | 2021 | -1.34 (-2.21 to -0.46) | 0.006 |
| Mongolia | DALYs | 1990 | 2004 | 1.77 (1.50 to 2.05) | <0.001 |
| Mongolia | DALYs | 2004 | 2010 | -0.37 (-1.49 to 0.77) | 0.512 |
| Mongolia | DALYs | 2010 | 2021 | -2.15 (-2.52 to -1.78) | <0.001 |
| Montenegro | DALYs | 1990 | 2003 | 1.30 (1.04 to 1.56) | <0.001 |
| Montenegro | DALYs | 2003 | 2007 | -0.34 (-2.47 to 1.83) | 0.746 |
| Montenegro | DALYs | 2007 | 2017 | 2.40 (1.91 to 2.89) | <0.001 |
| Montenegro | DALYs | 2017 | 2021 | -0.11 (-1.90 to 1.73) | 0.906 |
| Morocco | DALYs | 1990 | 2000 | -0.49 (-0.58 to -0.41) | <0.001 |
| Morocco | DALYs | 2000 | 2007 | 0.59 (0.41 to 0.77) | <0.001 |
| Morocco | DALYs | 2007 | 2010 | -0.90 (-1.95 to 0.16) | 0.09 |
| Morocco | DALYs | 2010 | 2018 | -0.19 (-0.33 to -0.04) | 0.018 |
| Morocco | DALYs | 2018 | 2021 | -1.09 (-1.59 to -0.59) | <0.001 |
| Mozambique | DALYs | 1990 | 1995 | -0.06 (-0.29 to 0.17) | 0.593 |
| Mozambique | DALYs | 1995 | 2003 | 1.21 (1.06 to 1.35) | <0.001 |
| Mozambique | DALYs | 2003 | 2011 | 2.34 (2.18 to 2.50) | <0.001 |
| Mozambique | DALYs | 2011 | 2014 | 0.39 (-0.94 to 1.73) | 0.55 |
| Mozambique | DALYs | 2014 | 2021 | -1.01 (-1.21 to -0.82) | <0.001 |
| Myanmar | DALYs | 1990 | 2005 | -0.87 (-0.89 to -0.85) | <0.001 |
| Myanmar | DALYs | 2005 | 2009 | -1.88 (-2.10 to -1.66) | <0.001 |
| Myanmar | DALYs | 2009 | 2013 | -2.35 (-2.56 to -2.14) | <0.001 |
| Myanmar | DALYs | 2013 | 2018 | -1.17 (-1.31 to -1.03) | <0.001 |
| Myanmar | DALYs | 2018 | 2021 | -0.08 (-0.34 to 0.19) | 0.546 |
| Namibia | DALYs | 1990 | 1997 | 0.89 (0.69 to 1.08) | <0.001 |
| Namibia | DALYs | 1997 | 2000 | 2.25 (0.86 to 3.66) | 0.004 |
| Namibia | DALYs | 2000 | 2003 | 0.86 (-0.62 to 2.37) | 0.236 |
| Namibia | DALYs | 2003 | 2008 | -2.97 (-3.40 to -2.53) | <0.001 |
| Namibia | DALYs | 2008 | 2012 | -1.88 (-2.61 to -1.14) | <0.001 |
| Namibia | DALYs | 2012 | 2021 | -0.34 (-0.50 to -0.17) | 0.001 |
| Nauru | DALYs | 1990 | 1993 | 1.19 (0.84 to 1.55) | <0.001 |
| Nauru | DALYs | 1993 | 1998 | 2.10 (1.91 to 2.30) | <0.001 |
| Nauru | DALYs | 1998 | 2002 | 0.58 (0.28 to 0.87) | 0.001 |
| Nauru | DALYs | 2002 | 2009 | -0.77 (-0.87 to -0.66) | <0.001 |
| Nauru | DALYs | 2009 | 2018 | -2.00 (-2.07 to -1.93) | <0.001 |
| Nauru | DALYs | 2018 | 2021 | -1.21 (-1.53 to -0.88) | <0.001 |
| Nepal | DALYs | 1990 | 1994 | -0.98 (-1.32 to -0.64) | <0.001 |
| Nepal | DALYs | 1994 | 2004 | -2.24 (-2.33 to -2.14) | <0.001 |
| Nepal | DALYs | 2004 | 2021 | -0.31 (-0.35 to -0.28) | <0.001 |
| Netherlands | DALYs | 1990 | 1999 | -1.43 (-1.64 to -1.21) | <0.001 |
| Netherlands | DALYs | 1999 | 2002 | -2.55 (-4.91 to -0.13) | 0.04 |
| Netherlands | DALYs | 2002 | 2007 | -6.57 (-7.36 to -5.77) | <0.001 |
| Netherlands | DALYs | 2007 | 2011 | -4.27 (-5.65 to -2.86) | <0.001 |
| Netherlands | DALYs | 2011 | 2015 | 0.06 (-1.42 to 1.56) | 0.933 |
| Netherlands | DALYs | 2015 | 2021 | -2.35 (-2.85 to -1.85) | <0.001 |
| New Zealand | DALYs | 1990 | 2002 | -3.04 (-3.25 to -2.83) | <0.001 |
| New Zealand | DALYs | 2002 | 2009 | -4.39 (-5.07 to -3.71) | <0.001 |
| New Zealand | DALYs | 2009 | 2021 | -1.69 (-1.97 to -1.42) | <0.001 |
| Nicaragua | DALYs | 1990 | 2000 | -2.16 (-2.64 to -1.68) | <0.001 |
| Nicaragua | DALYs | 2000 | 2004 | 1.06 (-1.84 to 4.04) | 0.463 |
| Nicaragua | DALYs | 2004 | 2021 | -2.30 (-2.54 to -2.06) | <0.001 |
| Niger | DALYs | 1990 | 1996 | -0.93 (-1.11 to -0.76) | <0.001 |
| Niger | DALYs | 1996 | 2003 | 0.45 (0.27 to 0.64) | <0.001 |
| Niger | DALYs | 2003 | 2008 | -0.90 (-1.25 to -0.55) | <0.001 |
| Niger | DALYs | 2008 | 2015 | 0.48 (0.30 to 0.66) | <0.001 |
| Niger | DALYs | 2015 | 2021 | -0.31 (-0.48 to -0.14) | 0.001 |
| Nigeria | DALYs | 1990 | 1994 | -0.55 (-0.88 to -0.21) | 0.003 |
| Nigeria | DALYs | 1994 | 1999 | 0.82 (0.49 to 1.14) | <0.001 |
| Nigeria | DALYs | 1999 | 2002 | -0.25 (-1.20 to 0.70) | 0.581 |
| Nigeria | DALYs | 2002 | 2007 | -2.61 (-2.87 to -2.35) | <0.001 |
| Nigeria | DALYs | 2007 | 2015 | -0.76 (-0.86 to -0.66) | <0.001 |
| Nigeria | DALYs | 2015 | 2021 | -1.26 (-1.38 to -1.13) | <0.001 |
| Niue | DALYs | 1990 | 1992 | 1.00 (-0.05 to 2.07) | 0.061 |
| Niue | DALYs | 1992 | 1997 | -0.03 (-0.36 to 0.30) | 0.862 |
| Niue | DALYs | 1997 | 2011 | -1.12 (-1.17 to -1.06) | <0.001 |
| Niue | DALYs | 2011 | 2018 | -0.67 (-0.84 to -0.49) | <0.001 |
| Niue | DALYs | 2018 | 2021 | 0.74 (0.21 to 1.26) | 0.008 |
| North Macedonia | DALYs | 1990 | 1998 | 3.93 (3.57 to 4.29) | <0.001 |
| North Macedonia | DALYs | 1998 | 2001 | 0.48 (-2.43 to 3.47) | 0.738 |
| North Macedonia | DALYs | 2001 | 2004 | -3.34 (-6.19 to -0.40) | 0.028 |
| North Macedonia | DALYs | 2004 | 2007 | 1.44 (-1.53 to 4.51) | 0.325 |
| North Macedonia | DALYs | 2007 | 2021 | -2.24 (-2.41 to -2.08) | <0.001 |
| Northern Mariana Islands | DALYs | 1990 | 1999 | -0.18 (-0.76 to 0.40) | 0.53 |
| Northern Mariana Islands | DALYs | 1999 | 2004 | -5.18 (-6.75 to -3.59) | <0.001 |
| Northern Mariana Islands | DALYs | 2004 | 2021 | -0.50 (-0.69 to -0.30) | <0.001 |
| Norway | DALYs | 1990 | 1992 | -1.56 (-3.86 to 0.79) | 0.176 |
| Norway | DALYs | 1992 | 1998 | -3.26 (-3.79 to -2.72) | <0.001 |
| Norway | DALYs | 1998 | 2006 | -4.72 (-5.08 to -4.36) | <0.001 |
| Norway | DALYs | 2006 | 2011 | -2.90 (-3.86 to -1.93) | <0.001 |
| Norway | DALYs | 2011 | 2017 | -5.46 (-6.18 to -4.73) | <0.001 |
| Norway | DALYs | 2017 | 2021 | -2.40 (-3.64 to -1.15) | 0.001 |
| Oman | DALYs | 1990 | 2006 | -1.88 (-2.13 to -1.63) | <0.001 |
| Oman | DALYs | 2006 | 2010 | 3.65 (1.37 to 5.98) | 0.003 |
| Oman | DALYs | 2010 | 2013 | -2.99 (-7.13 to 1.33) | 0.16 |
| Oman | DALYs | 2013 | 2016 | 2.47 (-1.54 to 6.65) | 0.216 |
| Oman | DALYs | 2016 | 2021 | -5.04 (-6.05 to -4.03) | <0.001 |
| Pakistan | DALYs | 1990 | 1996 | 2.58 (2.43 to 2.74) | <0.001 |
| Pakistan | DALYs | 1996 | 2003 | -0.32 (-0.46 to -0.18) | <0.001 |
| Pakistan | DALYs | 2003 | 2008 | -1.17 (-1.42 to -0.92) | <0.001 |
| Pakistan | DALYs | 2008 | 2017 | -0.14 (-0.22 to -0.05) | 0.004 |
| Pakistan | DALYs | 2017 | 2021 | -0.73 (-0.98 to -0.49) | <0.001 |
| Palau | DALYs | 1990 | 1996 | -0.98 (-1.23 to -0.73) | <0.001 |
| Palau | DALYs | 1996 | 1999 | -0.20 (-1.61 to 1.23) | 0.772 |
| Palau | DALYs | 1999 | 2003 | -0.84 (-1.48 to -0.19) | 0.014 |
| Palau | DALYs | 2003 | 2013 | 0.26 (0.15 to 0.38) | <0.001 |
| Palau | DALYs | 2013 | 2021 | -1.17 (-1.32 to -1.02) | <0.001 |
| Palestine | DALYs | 1990 | 1999 | -0.97 (-1.28 to -0.66) | <0.001 |
| Palestine | DALYs | 1999 | 2006 | 0.03 (-0.35 to 0.40) | 0.885 |
| Palestine | DALYs | 2006 | 2010 | -1.37 (-2.33 to -0.39) | 0.009 |
| Palestine | DALYs | 2010 | 2013 | -5.15 (-7.03 to -3.24) | <0.001 |
| Palestine | DALYs | 2013 | 2018 | -3.73 (-4.32 to -3.14) | <0.001 |
| Palestine | DALYs | 2018 | 2021 | -0.77 (-2.06 to 0.52) | 0.222 |
| Panama | DALYs | 1990 | 1994 | 1.76 (-0.68 to 4.25) | 0.15 |
| Panama | DALYs | 1994 | 2011 | -2.66 (-2.90 to -2.41) | <0.001 |
| Panama | DALYs | 2011 | 2016 | 0.96 (-1.30 to 3.27) | 0.391 |
| Panama | DALYs | 2016 | 2021 | -2.74 (-4.98 to -0.45) | 0.022 |
| Papua New Guinea | DALYs | 1990 | 2006 | 0.05 (0.01 to 0.09) | 0.018 |
| Papua New Guinea | DALYs | 2006 | 2015 | -1.29 (-1.39 to -1.20) | <0.001 |
| Papua New Guinea | DALYs | 2015 | 2021 | -0.38 (-0.55 to -0.21) | <0.001 |
| Paraguay | DALYs | 1990 | 2003 | -2.05 (-2.41 to -1.70) | <0.001 |
| Paraguay | DALYs | 2003 | 2021 | -0.47 (-0.70 to -0.24) | <0.001 |
| Peru | DALYs | 1990 | 1995 | 0.93 (-0.80 to 2.68) | 0.271 |
| Peru | DALYs | 1995 | 2007 | -4.62 (-5.08 to -4.16) | <0.001 |
| Peru | DALYs | 2007 | 2010 | 0.88 (-7.15 to 9.61) | 0.824 |
| Peru | DALYs | 2010 | 2015 | -2.90 (-5.28 to -0.45) | 0.024 |
| Peru | DALYs | 2015 | 2019 | 6.02 (1.88 to 10.34) | 0.007 |
| Peru | DALYs | 2019 | 2021 | -3.81 (-13.55 to 7.02) | 0.45 |
| Philippines | DALYs | 1990 | 1992 | -2.57 (-4.89 to -0.20) | 0.035 |
| Philippines | DALYs | 1992 | 1996 | 1.02 (-0.07 to 2.11) | 0.064 |
| Philippines | DALYs | 1996 | 1999 | -1.22 (-3.27 to 0.87) | 0.233 |
| Philippines | DALYs | 1999 | 2002 | 2.49 (0.51 to 4.51) | 0.016 |
| Philippines | DALYs | 2002 | 2021 | -0.38 (-0.44 to -0.32) | <0.001 |
| Poland | DALYs | 1990 | 1992 | -0.16 (-2.60 to 2.34) | 0.891 |
| Poland | DALYs | 1992 | 1997 | -2.85 (-3.62 to -2.08) | <0.001 |
| Poland | DALYs | 1997 | 2000 | -5.42 (-7.87 to -2.92) | <0.001 |
| Poland | DALYs | 2000 | 2008 | -3.17 (-3.55 to -2.79) | <0.001 |
| Poland | DALYs | 2008 | 2014 | -4.76 (-5.48 to -4.04) | <0.001 |
| Poland | DALYs | 2014 | 2021 | -2.12 (-2.67 to -1.56) | <0.001 |
| Portugal | DALYs | 1990 | 1999 | -4.27 (-4.50 to -4.03) | <0.001 |
| Portugal | DALYs | 1999 | 2003 | -7.62 (-9.05 to -6.16) | <0.001 |
| Portugal | DALYs | 2003 | 2006 | -9.33 (-12.59 to -5.95) | <0.001 |
| Portugal | DALYs | 2006 | 2016 | -6.14 (-6.49 to -5.78) | <0.001 |
| Portugal | DALYs | 2016 | 2021 | -2.80 (-3.81 to -1.79) | <0.001 |
| Puerto Rico | DALYs | 1990 | 1995 | 1.10 (-0.34 to 2.57) | 0.129 |
| Puerto Rico | DALYs | 1995 | 2003 | -4.84 (-5.78 to -3.89) | <0.001 |
| Puerto Rico | DALYs | 2003 | 2009 | -2.62 (-4.33 to -0.88) | 0.005 |
| Puerto Rico | DALYs | 2009 | 2021 | -4.13 (-4.68 to -3.58) | <0.001 |
| Qatar | DALYs | 1990 | 1992 | 2.21 (-5.22 to 10.23) | 0.546 |
| Qatar | DALYs | 1992 | 1995 | -10.74 (-17.12 to -3.85) | 0.005 |
| Qatar | DALYs | 1995 | 1998 | 11.72 (3.83 to 20.22) | 0.006 |
| Qatar | DALYs | 1998 | 2007 | -1.77 (-2.54 to -0.99) | <0.001 |
| Qatar | DALYs | 2007 | 2016 | -8.45 (-9.27 to -7.63) | <0.001 |
| Qatar | DALYs | 2016 | 2021 | 0.33 (-1.73 to 2.42) | 0.742 |
| Republic of Korea | DALYs | 1990 | 1999 | -3.45 (-3.65 to -3.24) | <0.001 |
| Republic of Korea | DALYs | 1999 | 2004 | -5.82 (-6.50 to -5.14) | <0.001 |
| Republic of Korea | DALYs | 2004 | 2009 | -7.87 (-8.66 to -7.07) | <0.001 |
| Republic of Korea | DALYs | 2009 | 2014 | -5.23 (-6.15 to -4.30) | <0.001 |
| Republic of Korea | DALYs | 2014 | 2019 | -3.23 (-4.24 to -2.21) | <0.001 |
| Republic of Korea | DALYs | 2019 | 2021 | -0.40 (-3.95 to 3.29) | 0.818 |
| Republic of Moldova | DALYs | 1990 | 1994 | 3.81 (0.40 to 7.33) | 0.03 |
| Republic of Moldova | DALYs | 1994 | 1998 | -9.01 (-12.02 to -5.90) | <0.001 |
| Republic of Moldova | DALYs | 1998 | 2006 | 4.15 (3.14 to 5.16) | <0.001 |
| Republic of Moldova | DALYs | 2006 | 2015 | -2.03 (-2.85 to -1.20) | <0.001 |
| Republic of Moldova | DALYs | 2015 | 2021 | -4.50 (-6.10 to -2.87) | <0.001 |
| Romania | DALYs | 1990 | 1996 | 1.72 (1.07 to 2.38) | <0.001 |
| Romania | DALYs | 1996 | 2000 | -3.31 (-4.92 to -1.66) | 0.001 |
| Romania | DALYs | 2000 | 2003 | 0.70 (-2.82 to 4.35) | 0.685 |
| Romania | DALYs | 2003 | 2013 | -3.67 (-3.99 to -3.34) | <0.001 |
| Romania | DALYs | 2013 | 2021 | -2.34 (-2.86 to -1.81) | <0.001 |
| Russian Federation | DALYs | 1990 | 1994 | 7.93 (5.42 to 10.51) | <0.001 |
| Russian Federation | DALYs | 1994 | 1998 | -2.75 (-6.13 to 0.75) | 0.115 |
| Russian Federation | DALYs | 1998 | 2003 | 1.60 (-0.62 to 3.87) | 0.148 |
| Russian Federation | DALYs | 2003 | 2012 | -6.08 (-6.84 to -5.31) | <0.001 |
| Russian Federation | DALYs | 2012 | 2021 | -3.12 (-4.07 to -2.16) | <0.001 |
| Rwanda | DALYs | 1990 | 1995 | 0.58 (0.27 to 0.88) | 0.001 |
| Rwanda | DALYs | 1995 | 1998 | -2.24 (-3.63 to -0.83) | 0.004 |
| Rwanda | DALYs | 1998 | 2005 | -6.15 (-6.37 to -5.93) | <0.001 |
| Rwanda | DALYs | 2005 | 2010 | -3.13 (-3.55 to -2.70) | <0.001 |
| Rwanda | DALYs | 2010 | 2016 | -0.52 (-0.84 to -0.19) | 0.004 |
| Rwanda | DALYs | 2016 | 2021 | 0.75 (0.39 to 1.10) | <0.001 |
| Saint Kitts and Nevis | DALYs | 1990 | 1996 | -0.85 (-2.44 to 0.78) | 0.292 |
| Saint Kitts and Nevis | DALYs | 1996 | 1999 | -6.31 (-13.80 to 1.84) | 0.12 |
| Saint Kitts and Nevis | DALYs | 1999 | 2021 | -1.50 (-1.72 to -1.28) | <0.001 |
| Saint Lucia | DALYs | 1990 | 1992 | -0.58 (-6.98 to 6.27) | 0.858 |
| Saint Lucia | DALYs | 1992 | 2000 | -5.36 (-6.12 to -4.60) | <0.001 |
| Saint Lucia | DALYs | 2000 | 2003 | -1.06 (-6.81 to 5.04) | 0.712 |
| Saint Lucia | DALYs | 2003 | 2011 | -5.51 (-6.32 to -4.69) | <0.001 |
| Saint Lucia | DALYs | 2011 | 2021 | 0.77 (-0.07 to 1.60) | 0.069 |
| Saint Vincent and the Grenadines | DALYs | 1990 | 2001 | -2.79 (-3.11 to -2.46) | <0.001 |
| Saint Vincent and the Grenadines | DALYs | 2001 | 2006 | -1.54 (-2.77 to -0.29) | 0.019 |
| Saint Vincent and the Grenadines | DALYs | 2006 | 2009 | 3.45 (-0.97 to 8.07) | 0.118 |
| Saint Vincent and the Grenadines | DALYs | 2009 | 2012 | -1.57 (-5.75 to 2.80) | 0.45 |
| Saint Vincent and the Grenadines | DALYs | 2012 | 2016 | -4.46 (-6.46 to -2.42) | <0.001 |
| Saint Vincent and the Grenadines | DALYs | 2016 | 2021 | 1.07 (-0.29 to 2.44) | 0.116 |
| Samoa | DALYs | 1990 | 1997 | -0.15 (-0.19 to -0.10) | <0.001 |
| Samoa | DALYs | 1997 | 2000 | -0.69 (-1.02 to -0.36) | <0.001 |
| Samoa | DALYs | 2000 | 2006 | -1.33 (-1.40 to -1.26) | <0.001 |
| Samoa | DALYs | 2006 | 2011 | -0.56 (-0.66 to -0.45) | <0.001 |
| Samoa | DALYs | 2011 | 2016 | 0.37 (0.26 to 0.48) | <0.001 |
| Samoa | DALYs | 2016 | 2021 | -0.69 (-0.77 to -0.62) | <0.001 |
| San Marino | DALYs | 1990 | 1998 | -2.42 (-2.91 to -1.93) | <0.001 |
| San Marino | DALYs | 1998 | 2009 | -4.35 (-4.67 to -4.04) | <0.001 |
| San Marino | DALYs | 2009 | 2019 | -0.20 (-0.61 to 0.22) | 0.33 |
| San Marino | DALYs | 2019 | 2021 | -17.53 (-23.32 to -11.30) | <0.001 |
| Sao Tome and Principe | DALYs | 1990 | 1995 | 0.43 (0.09 to 0.78) | 0.016 |
| Sao Tome and Principe | DALYs | 1995 | 1998 | 2.30 (0.70 to 3.93) | 0.008 |
| Sao Tome and Principe | DALYs | 1998 | 2006 | 0.69 (0.48 to 0.90) | <0.001 |
| Sao Tome and Principe | DALYs | 2006 | 2011 | -0.24 (-0.74 to 0.27) | 0.335 |
| Sao Tome and Principe | DALYs | 2011 | 2015 | 0.63 (-0.27 to 1.55) | 0.158 |
| Sao Tome and Principe | DALYs | 2015 | 2021 | -0.77 (-1.04 to -0.49) | <0.001 |
| Saudi Arabia | DALYs | 1990 | 1996 | 0.32 (0.11 to 0.53) | 0.006 |
| Saudi Arabia | DALYs | 1996 | 2002 | -0.99 (-1.16 to -0.81) | <0.001 |
| Saudi Arabia | DALYs | 2002 | 2005 | -1.96 (-2.60 to -1.32) | <0.001 |
| Saudi Arabia | DALYs | 2005 | 2008 | -0.45 (-1.14 to 0.24) | 0.184 |
| Saudi Arabia | DALYs | 2008 | 2013 | -2.48 (-2.71 to -2.25) | <0.001 |
| Saudi Arabia | DALYs | 2013 | 2021 | -1.63 (-1.73 to -1.52) | <0.001 |
| Senegal | DALYs | 1990 | 2021 | -0.50 (-0.56 to -0.44) | <0.001 |
| Serbia | DALYs | 1990 | 1996 | 2.15 (1.16 to 3.15) | <0.001 |
| Serbia | DALYs | 1996 | 2014 | -3.34 (-3.51 to -3.17) | <0.001 |
| Serbia | DALYs | 2014 | 2021 | -1.58 (-2.49 to -0.67) | 0.002 |
| Seychelles | DALYs | 1990 | 1995 | -1.30 (-2.83 to 0.25) | 0.093 |
| Seychelles | DALYs | 1995 | 2001 | -3.13 (-4.54 to -1.71) | <0.001 |
| Seychelles | DALYs | 2001 | 2005 | 2.62 (-0.72 to 6.07) | 0.117 |
| Seychelles | DALYs | 2005 | 2014 | -1.46 (-2.15 to -0.77) | <0.001 |
| Seychelles | DALYs | 2014 | 2018 | 0.61 (-2.78 to 4.11) | 0.713 |
| Seychelles | DALYs | 2018 | 2021 | -5.29 (-8.72 to -1.73) | 0.007 |
| Sierra Leone | DALYs | 1990 | 1994 | -1.08 (-1.37 to -0.79) | <0.001 |
| Sierra Leone | DALYs | 1994 | 2000 | 0.43 (0.23 to 0.63) | <0.001 |
| Sierra Leone | DALYs | 2000 | 2003 | 1.83 (0.88 to 2.78) | 0.001 |
| Sierra Leone | DALYs | 2003 | 2006 | 0.55 (-0.34 to 1.45) | 0.209 |
| Sierra Leone | DALYs | 2006 | 2011 | -0.77 (-1.07 to -0.46) | <0.001 |
| Sierra Leone | DALYs | 2011 | 2021 | -1.17 (-1.25 to -1.08) | <0.001 |
| Singapore | DALYs | 1990 | 1996 | -3.50 (-4.38 to -2.61) | <0.001 |
| Singapore | DALYs | 1996 | 2010 | -5.62 (-5.95 to -5.30) | <0.001 |
| Singapore | DALYs | 2010 | 2013 | -2.88 (-10.24 to 5.08) | 0.446 |
| Singapore | DALYs | 2013 | 2016 | -11.69 (-19.58 to -3.04) | 0.012 |
| Singapore | DALYs | 2016 | 2021 | -4.70 (-7.03 to -2.31) | 0.001 |
| Slovakia | DALYs | 1990 | 1994 | -2.54 (-3.54 to -1.53) | <0.001 |
| Slovakia | DALYs | 1994 | 1998 | -0.76 (-2.27 to 0.77) | 0.305 |
| Slovakia | DALYs | 1998 | 2002 | -2.81 (-4.32 to -1.29) | 0.001 |
| Slovakia | DALYs | 2002 | 2007 | -1.54 (-2.51 to -0.56) | 0.005 |
| Slovakia | DALYs | 2007 | 2013 | -3.65 (-4.37 to -2.91) | <0.001 |
| Slovakia | DALYs | 2013 | 2021 | -1.64 (-2.08 to -1.21) | <0.001 |
| Slovenia | DALYs | 1990 | 1992 | -1.32 (-5.17 to 2.68) | 0.494 |
| Slovenia | DALYs | 1992 | 1997 | -6.41 (-7.66 to -5.13) | <0.001 |
| Slovenia | DALYs | 1997 | 2005 | -4.83 (-5.44 to -4.21) | <0.001 |
| Slovenia | DALYs | 2005 | 2021 | -2.96 (-3.19 to -2.73) | <0.001 |
| Solomon Islands | DALYs | 1990 | 1993 | -0.06 (-0.91 to 0.79) | 0.881 |
| Solomon Islands | DALYs | 1993 | 2003 | -0.98 (-1.13 to -0.82) | <0.001 |
| Solomon Islands | DALYs | 2003 | 2008 | -0.39 (-0.92 to 0.15) | 0.145 |
| Solomon Islands | DALYs | 2008 | 2012 | 0.87 (-0.04 to 1.79) | 0.06 |
| Solomon Islands | DALYs | 2012 | 2021 | 0.31 (0.14 to 0.48) | 0.001 |
| Somalia | DALYs | 1990 | 1998 | -0.34 (-0.42 to -0.26) | <0.001 |
| Somalia | DALYs | 1998 | 2003 | -1.27 (-1.50 to -1.05) | <0.001 |
| Somalia | DALYs | 2003 | 2010 | -0.53 (-0.65 to -0.41) | <0.001 |
| Somalia | DALYs | 2010 | 2013 | -1.02 (-1.74 to -0.29) | 0.009 |
| Somalia | DALYs | 2013 | 2019 | -0.22 (-0.38 to -0.06) | 0.01 |
| Somalia | DALYs | 2019 | 2021 | -1.39 (-2.07 to -0.70) | 0.001 |
| South Africa | DALYs | 1990 | 1994 | 1.09 (-1.27 to 3.50) | 0.349 |
| South Africa | DALYs | 1994 | 1997 | 7.14 (1.18 to 13.46) | 0.021 |
| South Africa | DALYs | 1997 | 2003 | 1.94 (1.13 to 2.76) | <0.001 |
| South Africa | DALYs | 2003 | 2009 | 0.41 (-0.36 to 1.19) | 0.276 |
| South Africa | DALYs | 2009 | 2021 | -2.04 (-2.27 to -1.80) | <0.001 |
| South Sudan | DALYs | 1990 | 1995 | -0.71 (-0.88 to -0.54) | <0.001 |
| South Sudan | DALYs | 1995 | 1999 | -1.81 (-2.19 to -1.42) | <0.001 |
| South Sudan | DALYs | 1999 | 2002 | -2.34 (-3.12 to -1.56) | <0.001 |
| South Sudan | DALYs | 2002 | 2011 | -0.79 (-0.88 to -0.71) | <0.001 |
| South Sudan | DALYs | 2011 | 2021 | 0.83 (0.77 to 0.89) | <0.001 |
| Spain | DALYs | 1990 | 1997 | -6.04 (-6.37 to -5.70) | <0.001 |
| Spain | DALYs | 1997 | 2013 | -4.84 (-4.98 to -4.71) | <0.001 |
| Spain | DALYs | 2013 | 2017 | -0.62 (-2.39 to 1.18) | 0.476 |
| Spain | DALYs | 2017 | 2021 | -2.53 (-3.63 to -1.42) | <0.001 |
| Sri Lanka | DALYs | 1990 | 1993 | -4.82 (-7.00 to -2.60) | <0.001 |
| Sri Lanka | DALYs | 1993 | 1997 | 0.65 (-1.94 to 3.30) | 0.603 |
| Sri Lanka | DALYs | 1997 | 2002 | -1.14 (-2.54 to 0.27) | 0.105 |
| Sri Lanka | DALYs | 2002 | 2006 | 2.89 (0.82 to 5.00) | 0.009 |
| Sri Lanka | DALYs | 2006 | 2017 | -1.58 (-1.93 to -1.23) | <0.001 |
| Sri Lanka | DALYs | 2017 | 2021 | -3.93 (-7.59 to -0.12) | 0.044 |
| Sudan | DALYs | 1990 | 1995 | -0.43 (-0.54 to -0.33) | <0.001 |
| Sudan | DALYs | 1995 | 2000 | -1.05 (-1.21 to -0.89) | <0.001 |
| Sudan | DALYs | 2000 | 2005 | -0.46 (-0.62 to -0.30) | <0.001 |
| Sudan | DALYs | 2005 | 2011 | -1.48 (-1.59 to -1.37) | <0.001 |
| Sudan | DALYs | 2011 | 2017 | -1.09 (-1.20 to -0.99) | <0.001 |
| Sudan | DALYs | 2017 | 2021 | -0.17 (-0.33 to -0.02) | 0.033 |
| Suriname | DALYs | 1990 | 1993 | 4.28 (1.88 to 6.74) | 0.001 |
| Suriname | DALYs | 1993 | 1996 | -6.72 (-10.69 to -2.58) | 0.003 |
| Suriname | DALYs | 1996 | 2003 | 2.99 (2.24 to 3.74) | <0.001 |
| Suriname | DALYs | 2003 | 2011 | -3.61 (-4.18 to -3.05) | <0.001 |
| Suriname | DALYs | 2011 | 2021 | -0.20 (-0.63 to 0.23) | 0.346 |
| Sweden | DALYs | 1990 | 2002 | -1.77 (-1.96 to -1.57) | <0.001 |
| Sweden | DALYs | 2002 | 2005 | -6.45 (-9.92 to -2.85) | 0.001 |
| Sweden | DALYs | 2005 | 2009 | -2.41 (-4.27 to -0.51) | 0.015 |
| Sweden | DALYs | 2009 | 2021 | -4.03 (-4.29 to -3.77) | <0.001 |
| Switzerland | DALYs | 1990 | 2000 | -4.73 (-4.94 to -4.51) | <0.001 |
| Switzerland | DALYs | 2000 | 2012 | -3.56 (-3.78 to -3.33) | <0.001 |
| Switzerland | DALYs | 2012 | 2021 | -2.66 (-3.02 to -2.31) | <0.001 |
| Syrian Arab Republic | DALYs | 1990 | 1992 | 3.25 (-0.27 to 6.89) | 0.069 |
| Syrian Arab Republic | DALYs | 1992 | 2000 | -0.39 (-0.79 to 0.00) | 0.052 |
| Syrian Arab Republic | DALYs | 2000 | 2006 | -4.44 (-4.96 to -3.92) | <0.001 |
| Syrian Arab Republic | DALYs | 2006 | 2021 | -0.30 (-0.44 to -0.17) | <0.001 |
| Türkiye | DALYs | 1990 | 1996 | -1.92 (-2.72 to -1.11) | <0.001 |
| Türkiye | DALYs | 1996 | 2005 | -4.15 (-4.65 to -3.64) | <0.001 |
| Türkiye | DALYs | 2005 | 2015 | -0.25 (-0.67 to 0.17) | 0.226 |
| Türkiye | DALYs | 2015 | 2021 | -1.81 (-2.62 to -0.99) | <0.001 |
| Taiwan (Province of China) | DALYs | 1990 | 1994 | -5.62 (-6.73 to -4.50) | <0.001 |
| Taiwan (Province of China) | DALYs | 1994 | 2006 | -3.52 (-3.82 to -3.23) | <0.001 |
| Taiwan (Province of China) | DALYs | 2006 | 2009 | -6.83 (-11.60 to -1.80) | 0.011 |
| Taiwan (Province of China) | DALYs | 2009 | 2021 | -1.71 (-2.08 to -1.35) | <0.001 |
| Tajikistan | DALYs | 1990 | 1994 | 5.06 (3.98 to 6.15) | <0.001 |
| Tajikistan | DALYs | 1994 | 1997 | -4.25 (-7.22 to -1.18) | 0.01 |
| Tajikistan | DALYs | 1997 | 2001 | 0.08 (-1.46 to 1.64) | 0.915 |
| Tajikistan | DALYs | 2001 | 2008 | 1.85 (1.31 to 2.39) | <0.001 |
| Tajikistan | DALYs | 2008 | 2014 | -4.73 (-5.42 to -4.02) | <0.001 |
| Tajikistan | DALYs | 2014 | 2021 | -1.85 (-2.27 to -1.42) | <0.001 |
| Thailand | DALYs | 1990 | 1994 | 0.71 (-0.20 to 1.62) | 0.116 |
| Thailand | DALYs | 1994 | 1997 | -2.38 (-4.86 to 0.16) | 0.064 |
| Thailand | DALYs | 1997 | 2005 | -0.77 (-1.13 to -0.41) | <0.001 |
| Thailand | DALYs | 2005 | 2013 | -3.99 (-4.33 to -3.66) | <0.001 |
| Thailand | DALYs | 2013 | 2017 | -1.13 (-2.47 to 0.24) | 0.098 |
| Thailand | DALYs | 2017 | 2021 | 0.83 (-0.19 to 1.87) | 0.104 |
| Timor-Leste | DALYs | 1990 | 1994 | -0.72 (-0.98 to -0.46) | <0.001 |
| Timor-Leste | DALYs | 1994 | 1997 | -1.32 (-2.07 to -0.55) | 0.002 |
| Timor-Leste | DALYs | 1997 | 2004 | -0.20 (-0.33 to -0.06) | 0.008 |
| Timor-Leste | DALYs | 2004 | 2008 | 0.87 (0.42 to 1.31) | 0.001 |
| Timor-Leste | DALYs | 2008 | 2013 | 2.14 (1.81 to 2.48) | <0.001 |
| Timor-Leste | DALYs | 2013 | 2021 | 0.27 (0.13 to 0.40) | 0.001 |
| Togo | DALYs | 1990 | 1998 | 1.13 (1.00 to 1.27) | <0.001 |
| Togo | DALYs | 1998 | 2009 | -0.01 (-0.11 to 0.09) | 0.823 |
| Togo | DALYs | 2009 | 2014 | -1.66 (-2.11 to -1.21) | <0.001 |
| Togo | DALYs | 2014 | 2021 | -0.49 (-0.72 to -0.27) | <0.001 |
| Tokelau | DALYs | 1990 | 1998 | -0.52 (-0.58 to -0.47) | <0.001 |
| Tokelau | DALYs | 1998 | 2007 | -1.44 (-1.50 to -1.39) | <0.001 |
| Tokelau | DALYs | 2007 | 2019 | -1.22 (-1.26 to -1.19) | <0.001 |
| Tokelau | DALYs | 2019 | 2021 | 0.51 (-0.01 to 1.02) | 0.052 |
| Tonga | DALYs | 1990 | 1996 | -0.97 (-1.35 to -0.59) | <0.001 |
| Tonga | DALYs | 1996 | 1999 | 3.65 (1.50 to 5.85) | 0.002 |
| Tonga | DALYs | 1999 | 2006 | -1.49 (-1.83 to -1.16) | <0.001 |
| Tonga | DALYs | 2006 | 2011 | 0.72 (0.05 to 1.41) | 0.038 |
| Tonga | DALYs | 2011 | 2021 | -0.57 (-0.75 to -0.38) | <0.001 |
| Trinidad and Tobago | DALYs | 1990 | 1994 | -0.61 (-2.28 to 1.08) | 0.461 |
| Trinidad and Tobago | DALYs | 1994 | 2011 | -3.43 (-3.63 to -3.23) | <0.001 |
| Trinidad and Tobago | DALYs | 2011 | 2021 | -0.16 (-0.96 to 0.66) | 0.696 |
| Tunisia | DALYs | 1990 | 1995 | 0.41 (-0.09 to 0.90) | 0.102 |
| Tunisia | DALYs | 1995 | 2005 | -0.84 (-1.05 to -0.63) | <0.001 |
| Tunisia | DALYs | 2005 | 2021 | -1.48 (-1.60 to -1.36) | <0.001 |
| Turkmenistan | DALYs | 1990 | 1995 | 8.35 (6.39 to 10.36) | <0.001 |
| Turkmenistan | DALYs | 1995 | 1999 | -9.06 (-12.30 to -5.71) | <0.001 |
| Turkmenistan | DALYs | 1999 | 2006 | 6.94 (5.64 to 8.27) | <0.001 |
| Turkmenistan | DALYs | 2006 | 2010 | -5.30 (-8.53 to -1.95) | 0.004 |
| Turkmenistan | DALYs | 2010 | 2021 | -0.49 (-1.14 to 0.16) | 0.133 |
| Tuvalu | DALYs | 1990 | 1994 | -0.65 (-0.73 to -0.57) | <0.001 |
| Tuvalu | DALYs | 1994 | 1997 | -0.06 (-0.32 to 0.20) | 0.607 |
| Tuvalu | DALYs | 1997 | 2000 | -0.98 (-1.23 to -0.73) | <0.001 |
| Tuvalu | DALYs | 2000 | 2005 | -1.65 (-1.73 to -1.58) | <0.001 |
| Tuvalu | DALYs | 2005 | 2016 | -0.50 (-0.52 to -0.48) | <0.001 |
| Tuvalu | DALYs | 2016 | 2021 | -1.04 (-1.10 to -0.98) | <0.001 |
| Uganda | DALYs | 1990 | 1995 | 1.93 (1.75 to 2.10) | <0.001 |
| Uganda | DALYs | 1995 | 2000 | 0.53 (0.29 to 0.77) | <0.001 |
| Uganda | DALYs | 2000 | 2003 | -1.81 (-2.54 to -1.08) | <0.001 |
| Uganda | DALYs | 2003 | 2011 | -3.22 (-3.31 to -3.13) | <0.001 |
| Uganda | DALYs | 2011 | 2017 | -1.05 (-1.21 to -0.89) | <0.001 |
| Uganda | DALYs | 2017 | 2021 | 0.07 (-0.15 to 0.29) | 0.505 |
| Ukraine | DALYs | 1990 | 1995 | 2.96 (1.93 to 4.00) | <0.001 |
| Ukraine | DALYs | 1995 | 1998 | -4.46 (-8.56 to -0.16) | 0.043 |
| Ukraine | DALYs | 1998 | 2003 | -0.31 (-1.76 to 1.16) | 0.664 |
| Ukraine | DALYs | 2003 | 2016 | -4.00 (-4.27 to -3.73) | <0.001 |
| Ukraine | DALYs | 2016 | 2021 | -0.74 (-3.45 to 2.06) | 0.583 |
| United Arab Emirates | DALYs | 1990 | 1995 | -4.18 (-8.42 to 0.25) | 0.062 |
| United Arab Emirates | DALYs | 1995 | 2005 | 1.27 (-0.29 to 2.85) | 0.103 |
| United Arab Emirates | DALYs | 2005 | 2008 | 7.40 (-6.50 to 23.38) | 0.29 |
| United Arab Emirates | DALYs | 2008 | 2012 | -6.05 (-12.82 to 1.24) | 0.095 |
| United Arab Emirates | DALYs | 2012 | 2017 | 4.16 (-1.04 to 9.63) | 0.11 |
| United Arab Emirates | DALYs | 2017 | 2021 | -12.72 (-17.57 to -7.58) | <0.001 |
| United Kingdom | DALYs | 1990 | 2000 | -3.69 (-3.92 to -3.46) | <0.001 |
| United Kingdom | DALYs | 2000 | 2003 | -1.69 (-5.17 to 1.91) | 0.335 |
| United Kingdom | DALYs | 2003 | 2012 | -6.46 (-6.88 to -6.03) | <0.001 |
| United Kingdom | DALYs | 2012 | 2021 | -2.82 (-3.26 to -2.38) | <0.001 |
| United Republic of Tanzania | DALYs | 1990 | 1995 | 2.46 (2.17 to 2.76) | <0.001 |
| United Republic of Tanzania | DALYs | 1995 | 1998 | 0.63 (-0.62 to 1.89) | 0.3 |
| United Republic of Tanzania | DALYs | 1998 | 2001 | -0.73 (-1.79 to 0.35) | 0.171 |
| United Republic of Tanzania | DALYs | 2001 | 2009 | 1.96 (1.82 to 2.09) | <0.001 |
| United Republic of Tanzania | DALYs | 2009 | 2012 | -1.74 (-2.72 to -0.74) | 0.002 |
| United Republic of Tanzania | DALYs | 2012 | 2021 | -0.30 (-0.41 to -0.18) | <0.001 |
| United States of America | DALYs | 1990 | 1999 | -0.24 (-0.51 to 0.02) | 0.072 |
| United States of America | DALYs | 1999 | 2010 | -3.66 (-3.91 to -3.40) | <0.001 |
| United States of America | DALYs | 2010 | 2013 | -1.21 (-4.93 to 2.66) | 0.515 |
| United States of America | DALYs | 2013 | 2017 | 1.64 (-0.23 to 3.54) | 0.082 |
| United States of America | DALYs | 2017 | 2021 | -0.43 (-1.56 to 0.71) | 0.436 |
| United States Virgin Islands | DALYs | 1990 | 1998 | -2.32 (-2.55 to -2.09) | <0.001 |
| United States Virgin Islands | DALYs | 1998 | 2001 | -0.09 (-2.02 to 1.87) | 0.919 |
| United States Virgin Islands | DALYs | 2001 | 2004 | -2.22 (-4.22 to -0.18) | 0.035 |
| United States Virgin Islands | DALYs | 2004 | 2008 | -5.27 (-6.24 to -4.30) | <0.001 |
| United States Virgin Islands | DALYs | 2008 | 2019 | -1.30 (-1.48 to -1.12) | <0.001 |
| United States Virgin Islands | DALYs | 2019 | 2021 | -7.73 (-10.36 to -5.03) | <0.001 |
| Uruguay | DALYs | 1990 | 2005 | -1.78 (-2.01 to -1.56) | <0.001 |
| Uruguay | DALYs | 2005 | 2013 | -4.32 (-5.05 to -3.58) | <0.001 |
| Uruguay | DALYs | 2013 | 2021 | -2.42 (-3.09 to -1.76) | <0.001 |
| Uzbekistan | DALYs | 1990 | 1994 | 7.27 (5.35 to 9.23) | <0.001 |
| Uzbekistan | DALYs | 1994 | 1999 | -1.17 (-2.97 to 0.66) | 0.192 |
| Uzbekistan | DALYs | 1999 | 2003 | 1.25 (-1.79 to 4.37) | 0.399 |
| Uzbekistan | DALYs | 2003 | 2013 | -2.79 (-3.34 to -2.24) | <0.001 |
| Uzbekistan | DALYs | 2013 | 2017 | 1.78 (-0.95 to 4.58) | 0.187 |
| Uzbekistan | DALYs | 2017 | 2021 | -3.47 (-5.65 to -1.24) | 0.005 |
| Vanuatu | DALYs | 1990 | 1999 | -0.09 (-0.19 to 0.01) | 0.084 |
| Vanuatu | DALYs | 1999 | 2004 | -1.71 (-2.03 to -1.38) | <0.001 |
| Vanuatu | DALYs | 2004 | 2007 | 0.08 (-0.87 to 1.04) | 0.862 |
| Vanuatu | DALYs | 2007 | 2014 | -1.00 (-1.16 to -0.84) | <0.001 |
| Vanuatu | DALYs | 2014 | 2021 | -0.31 (-0.45 to -0.18) | <0.001 |
| Venezuela (Bolivarian Republic of) | DALYs | 1990 | 2012 | -1.78 (-1.97 to -1.60) | <0.001 |
| Venezuela (Bolivarian Republic of) | DALYs | 2012 | 2021 | 1.94 (0.78 to 3.11) | 0.002 |
| Viet Nam | DALYs | 1990 | 2000 | -0.24 (-0.27 to -0.21) | <0.001 |
| Viet Nam | DALYs | 2000 | 2003 | 1.60 (1.30 to 1.89) | <0.001 |
| Viet Nam | DALYs | 2003 | 2006 | 2.04 (1.75 to 2.33) | <0.001 |
| Viet Nam | DALYs | 2006 | 2009 | 1.43 (1.12 to 1.74) | <0.001 |
| Viet Nam | DALYs | 2009 | 2014 | -0.22 (-0.32 to -0.12) | <0.001 |
| Viet Nam | DALYs | 2014 | 2021 | -0.81 (-0.86 to -0.77) | <0.001 |
| Yemen | DALYs | 1990 | 1995 | -0.28 (-0.58 to 0.01) | 0.058 |
| Yemen | DALYs | 1995 | 2001 | -0.80 (-1.07 to -0.53) | <0.001 |
| Yemen | DALYs | 2001 | 2005 | 0.34 (-0.26 to 0.94) | 0.248 |
| Yemen | DALYs | 2005 | 2012 | -1.37 (-1.57 to -1.17) | <0.001 |
| Yemen | DALYs | 2012 | 2017 | -0.07 (-0.46 to 0.32) | 0.698 |
| Yemen | DALYs | 2017 | 2021 | 1.45 (1.01 to 1.89) | <0.001 |
| Zambia | DALYs | 1990 | 1996 | 1.92 (1.73 to 2.11) | <0.001 |
| Zambia | DALYs | 1996 | 2005 | -0.56 (-0.67 to -0.44) | <0.001 |
| Zambia | DALYs | 2005 | 2008 | 0.18 (-0.81 to 1.19) | 0.7 |
| Zambia | DALYs | 2008 | 2011 | 1.47 (0.53 to 2.43) | 0.005 |
| Zambia | DALYs | 2011 | 2017 | 0.69 (0.49 to 0.90) | <0.001 |
| Zambia | DALYs | 2017 | 2021 | -0.41 (-0.72 to -0.10) | 0.014 |
| Zimbabwe | DALYs | 1990 | 1994 | -0.80 (-2.12 to 0.54) | 0.221 |
| Zimbabwe | DALYs | 1994 | 1997 | 2.34 (-1.94 to 6.81) | 0.266 |
| Zimbabwe | DALYs | 1997 | 2000 | 9.02 (4.87 to 13.33) | <0.001 |
| Zimbabwe | DALYs | 2000 | 2004 | 4.54 (2.62 to 6.49) | <0.001 |
| Zimbabwe | DALYs | 2004 | 2008 | 0.15 (-1.90 to 2.24) | 0.881 |
| Zimbabwe | DALYs | 2008 | 2021 | -0.94 (-1.15 to -0.73) | <0.001 |
